# A gene deriving from the ancestral sex chromosomes was lost from the X and retained on the Y chromosome in eutherian mammals

**DOI:** 10.1186/s12915-022-01338-8

**Published:** 2022-06-09

**Authors:** Jennifer F. Hughes, Helen Skaletsky, Peter K. Nicholls, Alexis Drake, Tatyana Pyntikova, Ting-Jan Cho, Daniel W. Bellott, David C. Page

**Affiliations:** 1 Whitehead Institute, Cambridge, MA 02142 USA; 2 Howard Hughes Medical Institute, Whitehead Institute, Cambridge, MA 02142 USA; 3 Present Address: Faculty of Life Sciences, University of Bradford, BD71DP Bradford, UK; 4 Department of Biology, Massachusetts Institute of Technology, Cambridge, MA 02142 USA

**Keywords:** Y chromosome, Sex chromosomes, Sex ratio, Trypsin-like serine protease

## Abstract

**Background:**

The mammalian X and Y chromosomes originated from a pair of ordinary autosomes. Over the past ~180 million years, the X and Y have become highly differentiated and now only recombine with each other within a short pseudoautosomal region. While the X chromosome broadly preserved its gene content, the Y chromosome lost ~92% of the genes it once shared with the X chromosome. *PRSSLY* is a Y-linked gene identified in only a few mammalian species that was thought to be acquired, not ancestral. However, *PRSSLY*’*s* presence in widely divergent species—bull and mouse—led us to further investigate its evolutionary history.

**Results:**

We discovered that *PRSSLY* is broadly conserved across eutherians and has ancient origins. *PRSSLY* homologs are found in syntenic regions on the X chromosome in marsupials and on autosomes in more distant animals, including lizards, indicating that *PRSSLY* was present on the ancestral autosomes but was lost from the X and retained on the Y in eutherian mammals. We found that across eutheria, *PRSSLY*’s expression is testis-specific, and, in mouse, it is most robustly expressed in post-meiotic germ cells. The closest paralog to *PRSSLY* is the autosomal gene *PRSS55*, which is expressed exclusively in testes, involved in sperm differentiation and migration, and essential for male fertility in mice. Outside of eutheria, in species where *PRSSLY* orthologs are not Y-linked, we find expression in a broader range of somatic tissues, suggesting that *PRSSLY* has adopted a germ-cell-specific function in eutherians. Finally, we generated *Prssly* mutant mice and found that they are fully fertile but produce offspring with a modest female-biased sex ratio compared to controls.

**Conclusions:**

*PRSSLY* appears to be the first example of a gene that derives from the mammalian ancestral sex chromosomes that was lost from the X and retained on the Y. Although the function of *PRSSLY* remains to be determined, it may influence the sex ratio by promoting the survival or propagation of Y-bearing sperm.

**Supplementary Information:**

The online version contains supplementary material available at 10.1186/s12915-022-01338-8.

## Background

The sex chromosomes of all marsupials and placental mammals share a common origin—they evolved from a pair of autosomes during the past ~180 million years, likely after the divergence of monotremes [1, 2]. After the proto-Y chromosome acquired the male-determining gene *SRY*, the sex chromosomes began to differentiate, mediated through recombination suppression, which occurred in a series of discrete steps and spread across the length of the chromosomes [3]. The X and Y followed drastically different evolutionary trajectories because the X undergoes regular meiotic recombination in females, while the Y has no recombination partner over the majority of its length [4]. Consequently, the Y lost ~92% of its ancestral genes through repeated deletion and pseudogenization events. Despite this apparent evolutionary freefall, the gene content of the Y chromosome among eutherian mammals is highly conserved, suggesting that the remaining genes perform essential functions and are under strong selective pressure [5].

We identified a widespread yet uncharacterized mammalian Y-linked gene—*PRSSLY* (protease, serine-like Y) that appears to be the first example of a gene that survived on the Y chromosome but was lost from the X chromosome. *PRSSLY* escaped notice until recently because it is not present on the first three Y chromosomes sequenced to completion—human, chimpanzee, and rhesus macaque [6–8]. *PRSSLY* was first discovered on the mouse [9] and dog [10] Y chromosomes (initially named *DYNG* in dog), and homologs were subsequently found on the Y chromosomes of bull [11] and pig [12]. *PRSSLY* appears to encode a massive protein (average size: 2212 amino acid residues; largest: 4591 residues in deer; mammalian genome average: ~400 residues). Despite its size, *PRSSLY* contains only one identifiable domain—trypsin-like serine protease*—*making it part of the large *PRSS* gene family, which consists of 27 autosomal family members in human. *PRSSLY*’s expression pattern in eutherian mammals is testis-specific, suggesting a role in sperm development. Given its distinctive species distribution (conserved in diverse mammals, but lost in some lineages), gene structure, and expression pattern, we explored the evolution and function of *PRSSLY*.

## Results

### *PRSSLY* is widely distributed across mammals and has a unique gene structure

**Fig. 1:**
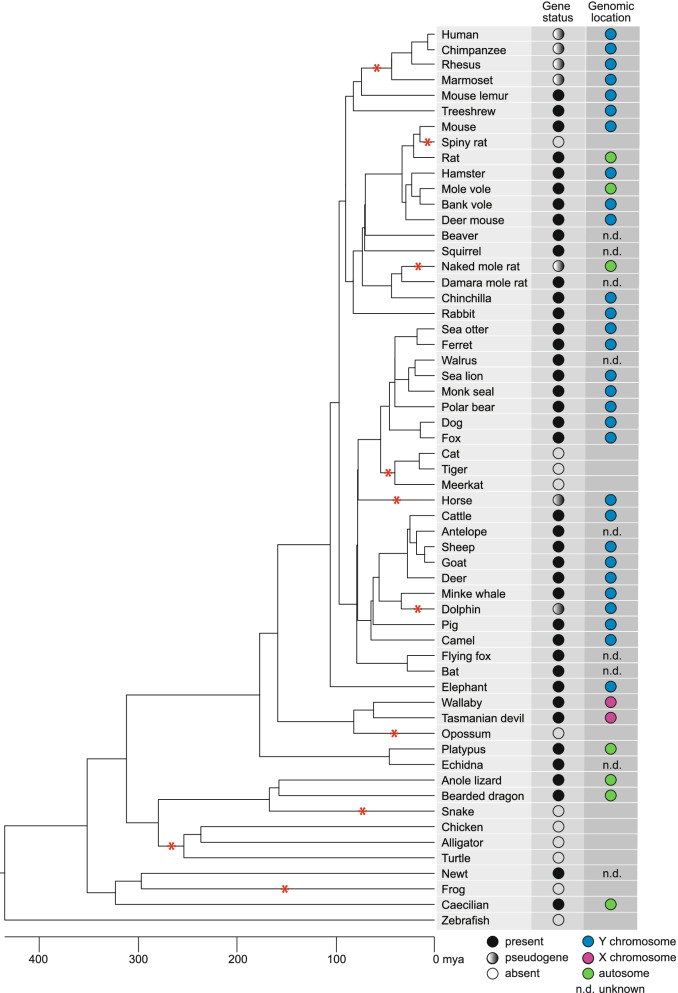
Species distribution of *PRSSLY* homologs. At left, tree diagram shows evolutionary relationships between species. Line length is proportional to time, with scale shown at bottom. Red asterisks indicate loss or pseudogenization of *PRSSLY* in a given lineage. At right, status of *PRSSLY* and chromosomal location (if known) are indicated

First, we assessed the conservation of *PRSSLY* in mammals by conducting a comprehensive survey of mammalian genomes for the gene’s presence or absence*.* The number of complete Y chromosomes available for analysis is limited, so we searched available male genomic DNA and testis RNA-seq datasets*.* In total, we examined datasets from 47 mammals and found that *PRSSLY* is present in species representing every major mammalian lineage, but it has been lost or pseudogenized repeatedly in multiple lineages: primates, felines, naked mole rat, horse, dolphin, and opossum (Fig. 1, Additional file 1). For 24 species, there is evidence of Y linkage because (i) *PRSSLY* sequence was found in confirmed Y chromosome sequence or (ii) *PRSSLY* was present in male whole-genome-shotgun sequence or RNA-seq datasets but missing from available female whole-genome-shotgun sequence (Additional file 1). The human, chimpanzee, and rhesus Y chromosomes contain loci with homology to *PRSSLY*, but these loci are pseudogenes. We found evidence that these pseudogenes are transcribed at low levels (via analysis of publicly available RNA-seq datasets), but they have severely truncated open reading frames (Additional file 2: Fig. S1). Y-linkage in mammals is not universal, however. *PRSSLY* has apparently translocated to an autosome at least three times in the rodent lineage (rat, mole vole, and naked mole rat) (Fig. 1). Phylogenetic analysis confirms that the autosomal copies in these species cluster with other *PRSSLY* homologs as expected, indicating that they are recently translocated (Additional file 2: Fig. S2). *PRSSLY* homologs are X-linked in marsupials and autosomal in monotremes (Additional file 2: Fig. S3, Additional file 1). Beyond mammals, we found *PRSSLY* homologs in species as divergent as lizards, newts, and caecilians (Fig. 1, Additional file 2: Fig. S4), so it likely arose in the tetrapod ancestor. However, this gene appears to have been lost in several major tetrapod lineages, including archelosauria (birds, crocodilians, and turtles), snakes, and frogs (Additional file 3).

**Fig. 2:**
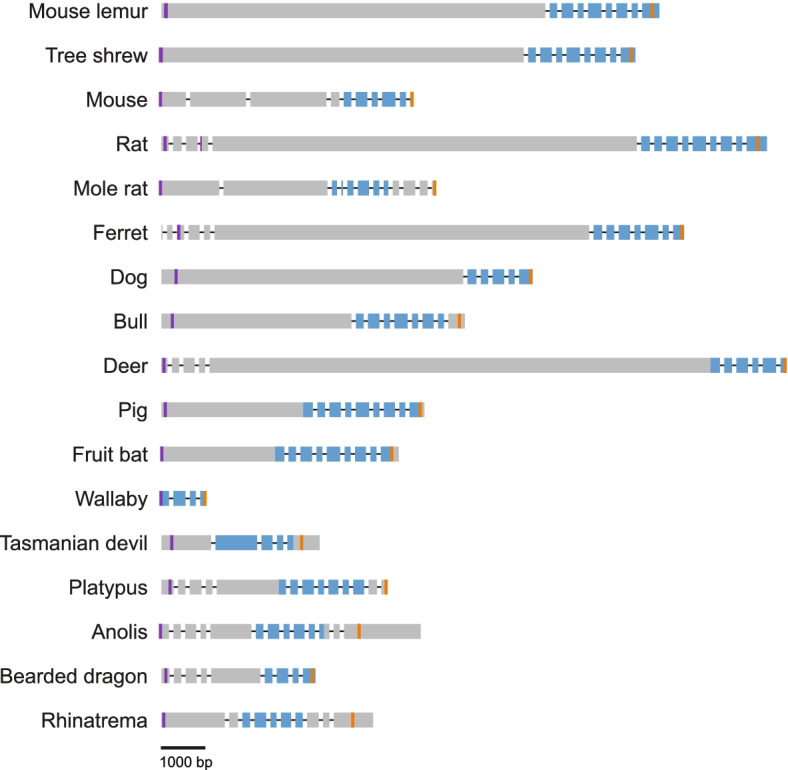
*PRSSLY* gene structure. Exons are indicated by boxes and are drawn to scale; introns are indicated by lines and are not drawn to scale. Conserved trypsin-like serine protease domains are shaded blue. Purple and orange lines indicate putative translation start sites and stop sites, respectively

The unusual gene structure of *PRSSLY* differs vastly between species (Fig. 2). The region encoding the conserved trypsin-like serine protease domain is contained within four to nine exons spanning ~1750 bp on average. However, in many species (e.g., mouse lemur, tree shrew, ferret, and deer), the entire open reading frame (ORF) spans >10 kb, with most of the ORF residing in a single exon (Fig. 2). These massive exons dwarf typical exons, which are ~300 bp on average in the human genome, and they rival the longest known coding exon, which is ~21 kb (in the gene *MUC16*) [13]. These long ORFs have no identifiable domains, only show homology between closely related species (Additional file 2: Fig. S5), and are less conserved than the trypsin-like serine protease domain. Using sequences from 27 animals, including mammals, reptiles, and amphibians, we calculated non-synonymous to synonymous substitution rate ratios (Ka/Ks) across the length of *PRSSLY* and *PRSSLY* homologs. We found that Ka/Ks is close to one within the upstream ORF and that Ka/Ks is much lower (from 0.001 to 0.31) within the conserved domain, indicating a slower rate of evolution within this domain (Additional file 2: Fig. S6, Additional files 4, 5). Furthermore, the non-synonymous substitution rate is consistently higher within the upstream ORF compared to the conserved domain (Additional file 2: Fig. S6).

### *PRSSLY* is testis-specific in eutherians but more broadly expressed in other animals

**Fig. 3:**
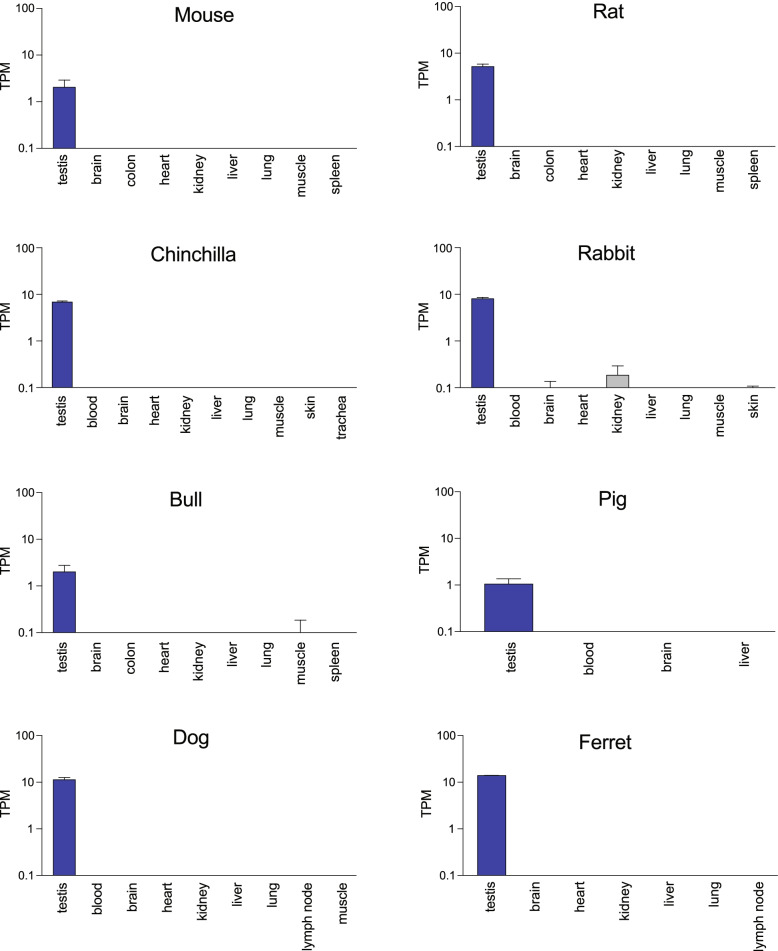
RNA-seq analysis of *PRSSLY* across tissues in eight eutherian mammals. Expression levels for *PRSSLY* were estimated in transcript per million (TPM) units. TPM values are plotted on a log10 scale. For some tissues, multiple biological replicates were analyzed for each tissue; means with standard errors are plotted. Details and source data can be found in Additional file 6

Next, we investigated the expression pattern of *PRSSLY* and its homologs across species. We examined expression in species where RNA-seq datasets from multiple tissues, including testis, were publicly available, and where the transcriptome was well annotated. In eutherian mammals, where Y linkage is nearly universal, *PRSSLY* is testis-specific (Fig. 3). Humans have 27 autosomal members of the *PRSS* gene family; about half of the family members share this testis-specific expression pattern, including *PRSSLY*’s closest relative *PRSS55* (Additional file 2: Fig. S7). We were able to refine the expression pattern of *PRSSLY* in mouse and bull. Using a germ-cell depleted mouse model [14, 15], we found that *PRSSLY* is expressed exclusively in adult germ cells (Additional file 2: Fig. S8). In bull, we analyzed previously published RNA-seq datasets generated from purified germ cells (pachytene spermatocytes and round spermatids) [16] and were able to detect transcription of bull *PRSSLY* in these samples (Additional file 2: Fig. S8), providing evidence that it is transcribed in male germ cells.

**Fig. 4:**
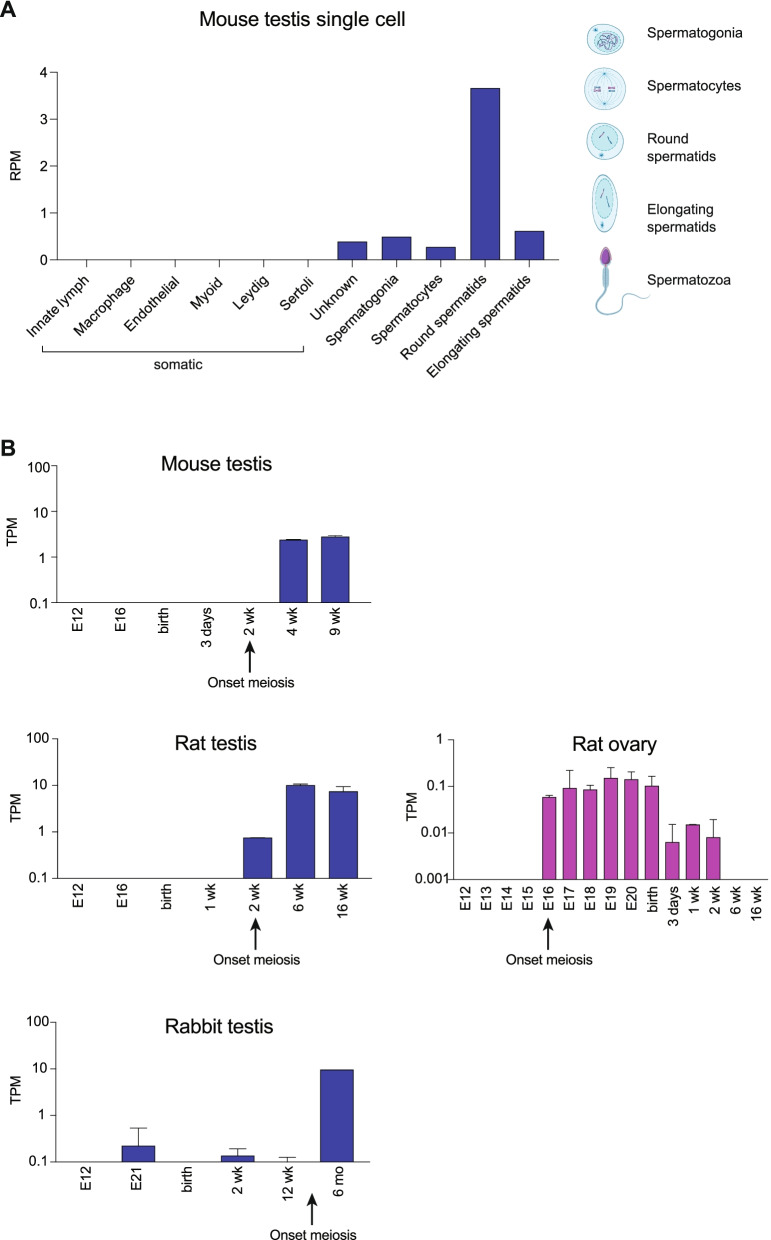
RNA-seq analysis of *PRSSLY* across development in mouse, rat, and rabbit. **A** For single-cell RNA-seq analysis in mouse, expression levels for *PRSSLY* are shown as reads per million mapped reads (RPM). At right, representative spermatogenic cells are shown (created with BioRender.com). **B** For bulk RNA-seq in mouse, rat, and rabbit across developmental timepoints, expression levels for *PRSSLY* were estimated in transcript per million (TPM) units. TPM values are plotted on a log10 scale. For some timepoints, multiple biological replicates were analyzed for each tissue; means with standard errors are plotted. Details and source data can be found in Additional file 6

Next, we examined the timing of *PRSSLY* expression in finer detail. For mouse, we examined publicly available single-cell RNA-seq datasets generated from adult whole testis [17]. Our analysis confirmed that *PRSSLY* is expressed only in germ cells and is absent in six somatic cell types included in this dataset (Fig. 4A). In germ cells, *PRSSLY* is barely detectable in pre-meiotic and meiotic cells (spermatogonia and spermatocytes, respectively), but is highly expressed in round spermatids—the developmental stage immediately following meiosis (Fig. 4A). *PRSSLY* expression is greatly reduced during the next stage of post-meiotic development (elongating spermatids).

We also examined publicly available testis bulk RNA-seq datasets spanning developmental timepoints from embryo to adult [18]. Such datasets were available for mouse, rat, and rabbit, and in all species the onset of *PRSSLY* expression correlates with the onset of meiosis (Fig. 4B). In rat, where *PRSSLY* was translocated to an autosome, we looked at available time course RNA-seq data from a variety of female tissues: ovary, brain, heart, kidney, and liver. *PRSSLY* expression was detected, at very low levels, in ovary, but was absent in all somatic tissues (Fig. 4B; Additional file 6). As in testis, the onset of *PRSSLY* expression in the rat ovary correlates with the onset of meiosis, but the functional relevance of this ovarian expression is unknown. The factor that activates *PRSSLY* at the onset of meiosis may be expressed in both males and females and conserved between mouse and rat. Since the entire *PRSSLY* gene, including introns, was translocated from the Y chromosome to chr14, the promoter was also likely translocated, accounting for the conserved expression pattern. Unfortunately, the sequence upstream of *PRSSLY* is too short to allow for comparison.

**Fig. 5:**
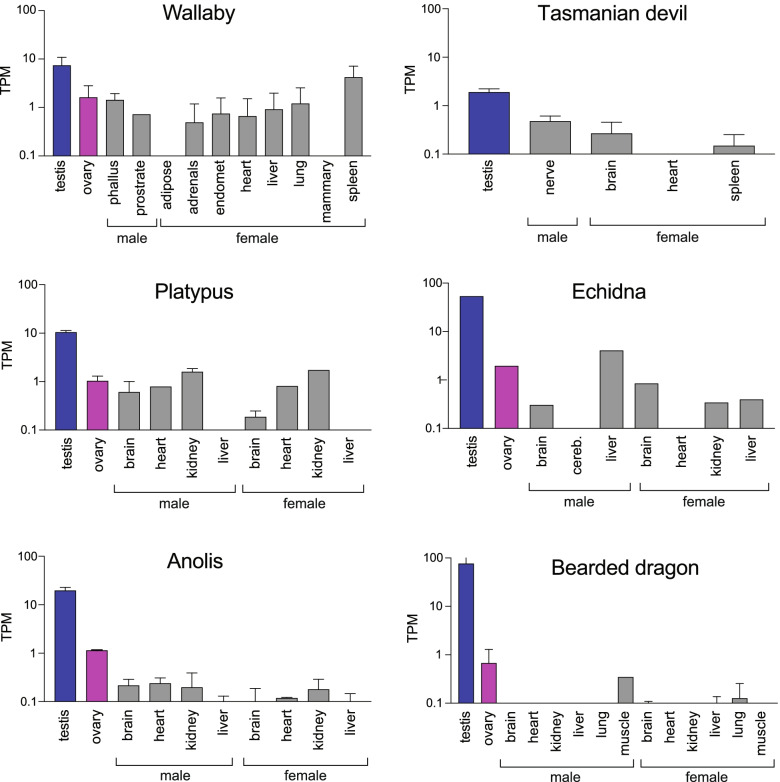
RNA-seq analysis of *PRSSLY* homologs (located on X chromosome and autosomes) across tissues in marsupials, monotremes, and lizards. Expression levels for *PRSSLY* homologs were estimated in transcript per million (TPM) units. TPM values are plotted on a log10 scale. For some tissues, multiple biological replicates were analyzed for each tissue; means with standard errors are plotted. Details and source data can be found in Additional file 6

Outside of eutherians, *PRSSLY* homologs, which are located on the X chromosome or autosomes, are more broadly expressed (Fig. 5). We examined publicly available RNA-seq data for two marsupials, two monotremes, and two lizards where multiple tissue types, including testis and ovary, were available. Non-Y-linked *PRSSLY* homologs are expressed in both males and females in both gonadal and somatic tissues. In most species, especially lizards, *PRSSLY* expression is highest in testis.

### Unique evolutionary history of *PRSSLY*

**Fig. 6:**
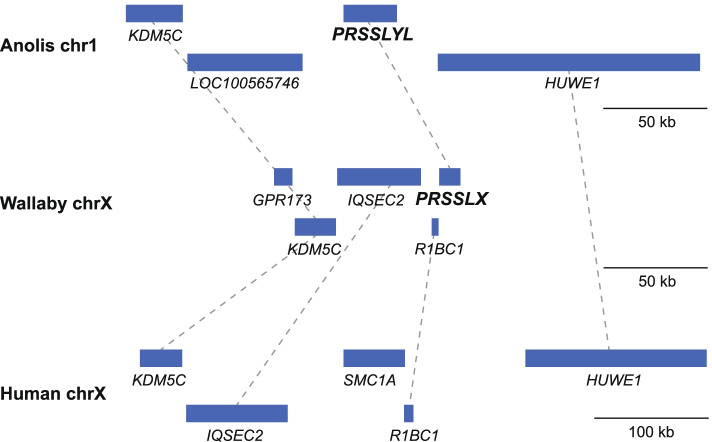
Syntenic relationships in anole lizard, wallaby, and human. Gene positions (blue boxes) in vicinity of autosomal *PRSSLYL* in anole lizard and X-linked *PRSSLX* in wallaby, as well as syntenic region on human X chromosome, which is missing a *PRSSLY* homolog, are shown. Gene positions based on genome assemblies for anole lizard chr1 (Broad AnoCar2.0/anoCar2) and human X chromosome (GRCh38/hg38). The genome assembly for wallaby does not provide sufficient X coverage, but an X-chromosome-derived BAC sequence is available (accession number CU234131) that contains *PRSSLX* and upstream genes. Dashed lines connect homologous genes

The chromosomal location and expression pattern of *PRSSLY* have evolved over time. The most parsimonious explanation for the gene’s evolutionary trajectory is supported by synteny analysis (Fig. 6). We propose that the gene originated in the tetrapod ancestor on the autosome pair that eventually became the proto-X and Y chromosomes in mammals [19]. In the ancestor of placental mammals, the X and Y chromosomes were expanded through an autosomal transposition event. *PRSSLX/Y* was located within stratum 2, which is part of the ancestral, conserved region [3]. After the placental-marsupial split, *PRSSLX/Y* was lost from the Y chromosome but retained on the X in marsupials, and lost from the X chromosome but retained on the Y in eutherian mammals. The Y-linked version in eutherians then became restricted in its expression pattern, perhaps acquiring a novel function in spermatogenesis. This evolutionary trajectory is highly unusual. While ~92% of the 636 genes once shared between the X and Y chromosomes have been lost from the eutherian Y chromosome and retained on the X chromosome [20], *PRSSLY* is the first and only example of an ancestral X-Y pair gene lost from the X chromosome and retained on the Y chromosome.

### Sex ratio of *Prssly*-knockout offspring is skewed towards females

**Fig. 7:**
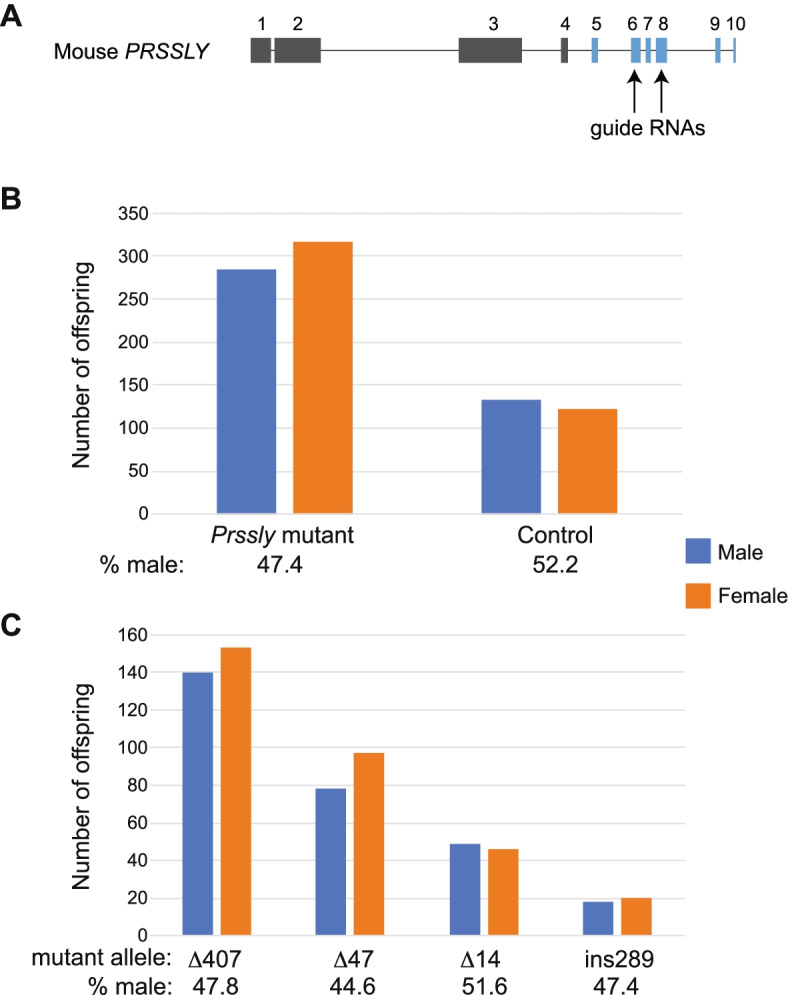
Sex ratio distortion in *PRSSLY* mutant offspring. **A** Structure of mouse *Prssly* gene. Exons are indicated by boxes, and introns by lines; both drawn to scale. Conserved trypsin-like serine protease domains are shaded blue. Arrows indicate positions of CRISPR guide RNAs. **B** and **C** Total number of male and female offspring in all four mutant lines vs. controls (**B**) and number of male and female offspring in each mutant line (**C**). Two-sided chi-square tests comparing offspring sex numbers in mutants (observed) vs. controls (expected) were performed; *p*=0.019 for all mutants vs. controls (**B**) and *p*=0.043 for Δ47 (**C**). *P* values for Δ407, Δ14, and ins289 were not significant

We explored *Prssly*’s function by generating likely loss-of-function CRISPR mutations in mice. We designed guide RNAs to target exons 6 and 8, which are part of the conserved trypsin-like serine protease domain (Fig. 7A). We obtained four founder males with various frame-disrupting mutations: (i) a 407-bp deletion between exons 6 and 8, creating a premature stop (Δ407); (ii) a 289-bp retroviral insertion into exon 6, creating a premature stop (ins289); (iii) a 14-bp deletion in exon 6, creating a premature stop (Δ14); and (iv) a 47-bp deletion, including the first 20 bp of exon 8, likely disrupting splicing (Δ47) (Additional file 2: Fig. S9). The mutations were introduced near the 3’ terminus of the gene (Additional file 2: Fig. S9), so we cannot rule out the possibility that *Prssly*’s function is partly preserved in these mutants.

Given *Prssly*’s testis-specific expression pattern in mouse, dog, and bull [9–11], we anticipated that these mutations might affect spermatogenesis. We found that *Prssly* mutants had testis weights that were within the normal range, but were significantly less than those of controls (Additional file 2: Fig. S10). However, males carrying any of the four alleles were fertile and had normal testis histology (Additional file 2: Fig. S11). A recent study, which also generated and characterized a *Prssly*-mutant mouse via CRISPR (targeting exon 5), confirmed these results: mutants were fertile with normal testis size and sperm morphology [21].

We continued breeding the mutant lines, and a clear phenotype gradually emerged: the sex ratio of the offspring of the *Prssly* mutant males was skewed towards females. We generated 95 litters and a total of 601 offspring (Additional file 7). Among these 601 offspring of *Prssly* mutant males, 47.4% were male, which is significantly lower than the 52.2% males we observed among 255 offspring of control males (Fig. 7B). If we consider each of the four mutant lines separately, the strength of the sex-ratio skewing varies (Fig. 7C). We observe no effect in the Δ14 mutant, which may indicate that *PRSSLY* is at least partially functional in this line or we have an inadequate number of offspring to detect sex ratio skewing.

We also found that the sizes of the weaned litters produced by *Prssly* mutant mice were significantly smaller (~1.5 fewer offspring per litter) than litters produced by control mice (Additional file 2: Fig. S12), so the sex-ratio skewing could be due to a sex difference in embryonic lethality or early postnatal survival rate. However, our breeding experiments were not designed to track variation in litter size (e.g. offspring were not counted immediately after birth) so we cannot conclude that there is a *Prssly-*related effect. Moreover, when each mutant is considered separately, the magnitude of the litter size difference (Additional file 2: Fig. S12) does not correlate with the magnitude of the sex-ratio skewing (Fig. 7C), suggesting that the two observations are unrelated.

## Discussion

We characterized a novel testis-specific Y-linked gene—*PRSSLY*—that is widespread in eutherian mammals and has ancient origins, dating back at least ~350 million years. *PRSSLY* is the first known example of a gene that survived on the mammalian Y chromosome but was lost from the X chromosome. The mammalian X and Y chromosomes originated from a pair of autosomes [2, 22]. Over the past ~180 million years, the X and Y chromosomes followed divergent evolutionary paths, with the Y losing ~92% of the genes it once shared with the X, while the ancestral gene content of the X remained essentially unchanged [20]. It is thus highly unusual that *PRSSLY*, which was clearly present on the ancestral autosome that gave rise to the mammalian X and Y, was lost specifically from the X chromosome in eutherian mammals. The Y copy was subsequently lost in several distinct eutherian lineages, but *PRSSLY* has survived for tens of millions of years in most lineages. In marsupials, the opposite, and more common, pattern appears, with the X copy being retained and the Y copy being lost. However, not all marsupial lineages have retained the X copy, which parallels the lineage-specific loss of the Y copy in eutherian mammals.

We probed *Prssly*’s function in mice and found that *Prssly* mutants are fertile, yet produce more female offspring than expected. In mice, *Sly – Slx/Slx1* are sex-chromosome genes that have been found to influence the sex ratio through intragenomic conflict in post-meiotic germ cells. Unlike *Prssly*, *Sly – Slx/Slx1* are not conserved outside of the *Mus* lineage. *Sly – Slx/Slx1* are also highly amplified on the sex chromosomes, with ~120 copies of *Sly* on the mouse Y long arm and ~40 copies of *Slx/Slx1* on the mouse X chromosome. Mice with a deletion encompassing two-thirds of the Y long-arm produce excess females (38% male) [23]. ShRNA-knockdowns of *Slx/Slx1* in males results in offspring sex ratio skewing towards males (60% males) [24]. A separate study showed that targeted deletion and duplication of the *Slx/Slx1* gene family skewed sex ratios towards males and females, respectively [25]. *Sly* and *Slx/Slxl1* deficiencies result in sperm head/spermatid elongation defects and sperm release defects, respectively [23, 26]. Double knock-down of *Sly* and *Slx/Slx1* rescues both the sperm defects and the skewed sex ratio [24]. We found no connection between *Prssly* and *Sly/Slx/Slx1* when we examined testis single-cell RNA-seq data [17] for evidence of correlated gene expression, so these systems appear to operate independently.

Although we do not yet know the mechanism by which *PRSSLY* affects the sex ratio in mice, *PRSSLY* likely operates directly in the male germline at or after the onset of meiosis based on its expression pattern. The function of *PRSSLY*’s closest relative—*PRSS55*—may also provide some clues. *PRSS55* is essential for male mouse fertility, playing a role in sperm motility and sperm–egg binding [27] as well as structural differentiation and energy metabolism [28]. Although *PRSSLY* is not required for fertility, it may act in a similar post-meiotic fashion to ensure the propagation of Y-bearing sperm. A full characterization of sperm morphology and sperm count in *Prssly* mutants will help elucidate this mechanism.

## Conclusions

This study uncovers a widespread mammalian Y-linked gene—*PRSSLY—* that appears to have survived on the Y chromosome but was lost from the X in eutherians, defying the trend set by >600 genes that followed the opposite evolutionary path during X-Y differentiation. In mice, *Prssly* is expressed strictly in post-meiotic male germ cells and appears to influence the sex ratio, perhaps by promoting the propagation of Y-bearing sperm. Whether *PRSSLY* plays a similar role in other species remains to be determined. If so, this discovery could open the door to the possibility of manipulating sex ratios in livestock, which would be of great interest, both biologically and commercially.

## Methods

### Identification of *PRSSLY* homologs

Using NCBI Blast suite with default parameters, we performed TBLASTN (protein sequence against translated nucleotide database) searches of NCBI’s non-redundant nucleotide database using *PRSSLY* sequences from bull and mouse as query sequences. Once more divergent *PRSSLY* sequences were identified (i.e., wallaby, lizard, and caecilian), we repeated the TBLASTN searches with the newly identified sequences as queries. To search for *PRSSLY* in species without available male genomic sequence, we scanned NCBI’s Sequence Read Archive database for available testis RNA-seq datasets and performed mapping analyses using *PRSSLY* sequence from the most closely related species (Additional file 1). To confirm that *PRSSLY* homologs were missing in certain species, we searched genomic assemblies using NCBI Blast suite with default parameters, using *PRSSLY* homolog in most closely related species as the query sequence. For species with or without closely related *PRSSLY* homologs we used BLASTN or TBLASTN, respectively. When genomic assemblies were not available, we searched short read datasets (RNA-seq or WGS) using the following pipeline: Fastq files were reformatted to fasta files; BLAST database was created using the makeblastdb function (version 2.10.1+); resulting database was searched with blastn (version 2.10.1+) (Additional file 3). We determined that *PRSSLY* is single-copy in all species with high-quality reference assemblies. For species without such assemblies, we searched for evidence of multiple *PRSSLY* copies using the following strategies but found none. First, we found no polymorphisms in *PRSSLY* RNA-seq reads. Second, we found no increased coverage of *PRSSLY* in raw genomic reads*.*

### Alignments, phylogenetic, and dot plot analyses

Nucleotide sequence alignment of conserved regions of *PRSSLY* homologs was performed using PRANK (version 121002) with default parameters [29]. Phylogenetic tree using nucleotide alignment was generated using PhyML (version 3.3) with default parameters [30]. Amino acid sequence alignments were performed using Clustalw (version 2.1) with default parameters [31]. Phylogenetic trees of *PRSS* gene family using amino acid alignment were generated using maximum likelihood in PHYLIP (version 3.66) with Jones-Taylor-Thornton model. For Ka-Ks analysis, separate alignments were generated (using Clustalw) for the conserved trypsin-like serine protease domain and the upstream ORF region. Ka/Ks calculations were performed with KaKs_Calculator (version 2.0) using codon alignments [32]. Alignment lengths of upstream ORFs were determined using FASTA (version 36.3) [33]. Dotplots were generated in MacVector (version 17.0.10) using default parameters.

### RNA-seq analysis

For each species, RNA-seq datasets were downloaded from NCBI’s Sequence Read Archive database, and transcriptomes were downloaded from Ensembl (transcriptome versions given in Additional file 6). For bulk analyses, RNA-seq reads were mapped to their respective transcriptomes using Salmon version 1.6.0 with the mapping validation option enabled [34]. For single-cell analysis, reads were mapped using Bowtie version 1.2.2 [35], and cell types were assigned as previously published [17].

### Generation of CRISPR mutations and mouse husbandry

The *Prssly* mutant mice were generated via a CRISPR/Cas9-mediated strategy on the C57BL/6J background. We designed two gRNAs, one targeting the end of exon 6 and the other targeting the start of exon 8, with the goal of producing a cut at both sites, and ideally, a deletion of the genomic DNA between these two sites. Experimental and control animals were backcrossed to C57BL/6J for an additional two generations or more. Deletions and insertions in founders and offspring were confirmed by PCR amplification and Sanger sequencing. Male offspring with edits to *Prssly* were subsequently backcrossed to C57BL/6J for two or more additional generations. The integrity of the long arm was confirmed by 18 PCR assays spanning the mouse Y (Additional file 8). The line used for controls was derived from founder littermates that did not contain CRISPR edits or mutations. Thus, controls and mutants shared the same paternal Y chromosome lineage. Litters were counted and sexed at day 5 and again prior to weaning. We genotyped males and females for the presence of the Y chromosome and found perfect correlation with observed phenotypic sex. To minimize variability between controls and mutants, all mice were maintained in the same room, were handled by the same staff, received cage changes on the same day, and received the same diet. Data collection for controls and mutants was performed in parallel. All experiments conformed to principles and guidelines approved by the Committee on Animal Care at the Massachusetts Institute of Technology.

## Supplementary Information

**Additional file 1. **Table of species distribution of *PRSSLY* homologs, including accession numbers for genomic and expression datasets used for identification of *PRSSLY.*

**Additional file 2: Fig. S1**. Structure and RNA-seq analysis of human, chimpanzee, and rhesus *PRSSLY* pseudogenes. **Fig. S2**. Phylogenetic analysis of *PRSSLY* nucleotide sequences. **Fig. S3**. Confirmation of X-linkage of *PRSSLY* in marsupials. **Fig. S4**. Phylogenetic analyses of *PRSS* family amino acid sequences. **Fig. S5**. Sequence conservation across *PRSSLY* gene sequences. **Fig. S6**. Analysis of synonymous (Ks) and non-synonymous (Ka) substitution rates across *PRSSLY.* **Fig. S7**. Expression of human *PRSS* homologs across tissues. **Fig. S8**. Gene expression analysis of *PRSSLY* in purified male germ cells and germ-cell-depleted testis. **Fig. S9**. Four CRISPR-induced mutations in mouse *PRSSLY*. **Fig. S10**. Testis weights of control and *Prssly* mutant mice. **Fig. S11**. Testis histology of control and *Prssly* mutant mice. **Fig. S12**. Litter sizes in *Prssly* mutants and controls.

**Additional file 3. **Table detailing evidence that *PRSSLY* homologs are missing in species.

**Additional file 4. **Ka-Ks analysis of *PRSSLY* and homologs.

**Additional file 5. **Sequences used for Ka-Ks analysis of *PRSSLY* and homologs.

**Additional file 6.** RNA-seq analyses including accession numbers and descriptions of RNA-seq datasets, transcriptomes used for mapping, and mapping results.

**Additional file 7. **Breeding of *PRSSLY* mutant mice.

**Additional file 8. **PCR assays across mouse Y long arm in *PRSSLY* mutant mice.

**Additional file 9. **Sequences and GenBank accession numbers for *PRSSLY* sequences.

## Data Availability

Nucleotide sequences for *PRSSLY* and non-Y-linked *PRSSLY* homologs that were assembled from genomic and/or RNA-seq data are available in the Third Party Annotation Section of the DDBJ/ENA/GenBank databases under the accession numbers TPA: BK059441-BK059443, BK059500-BK59524, and OK484381-OK484382 (see Additional File 9 for all sequences).

## Abbreviations

ORF: Open reading frame
Ka: Non-synonymous substitution rate
Ks: Synonymous substitution rate
