# Supplementary material for: A gene deriving from the ancestral sex chromosomes was lost from the X and retained on the Y chromosome in eutherian mammals
Source: BMC Biol. 2022 Jun 9;20:133. doi: 10.1186/s12915-022-01338-8 (PMC9178871; doi:10.1186/s12915-022-01338-8)

**Additional File 2: Supplementary Figures for Hughes et al., A gene deriving from the ancestral sex chromosomes was lost from the X and retained on the Y chromosome in eutherian mammals**

**Fig. S1: Structure and RNA-seq analysis of human, chimpanzee, and rhesus *PRSSLY* pseudogenes.** (A) Intact *PRSSLY* in mouse lemur is shown at top for comparison. Length of open reading frame (ORF) is indicated by black line above gene or pseudogene. For human, chimpanzee, and rhesus, the two longest ORFs are shown. Exons are indicated by boxes and are drawn to scale; introns are indicated by lines and are not drawn to scale. Conserved trypsin-like serine protease domains are shaded blue. (B) RNA-seq analysis of *PRSSLY* gene and pseudogene across tissues in mouse lemur, human, chimpanzee, and rhesus macaque. Expression levels for *PRSSLY* pseudogene were estimated in transcript per million (TPM) units. TPM values are plotted on a log10 scale. When multiple biological replicates were analyzed for each tissue, means with standard errors are plotted. Details and source data can be found in Additional File 6.

**
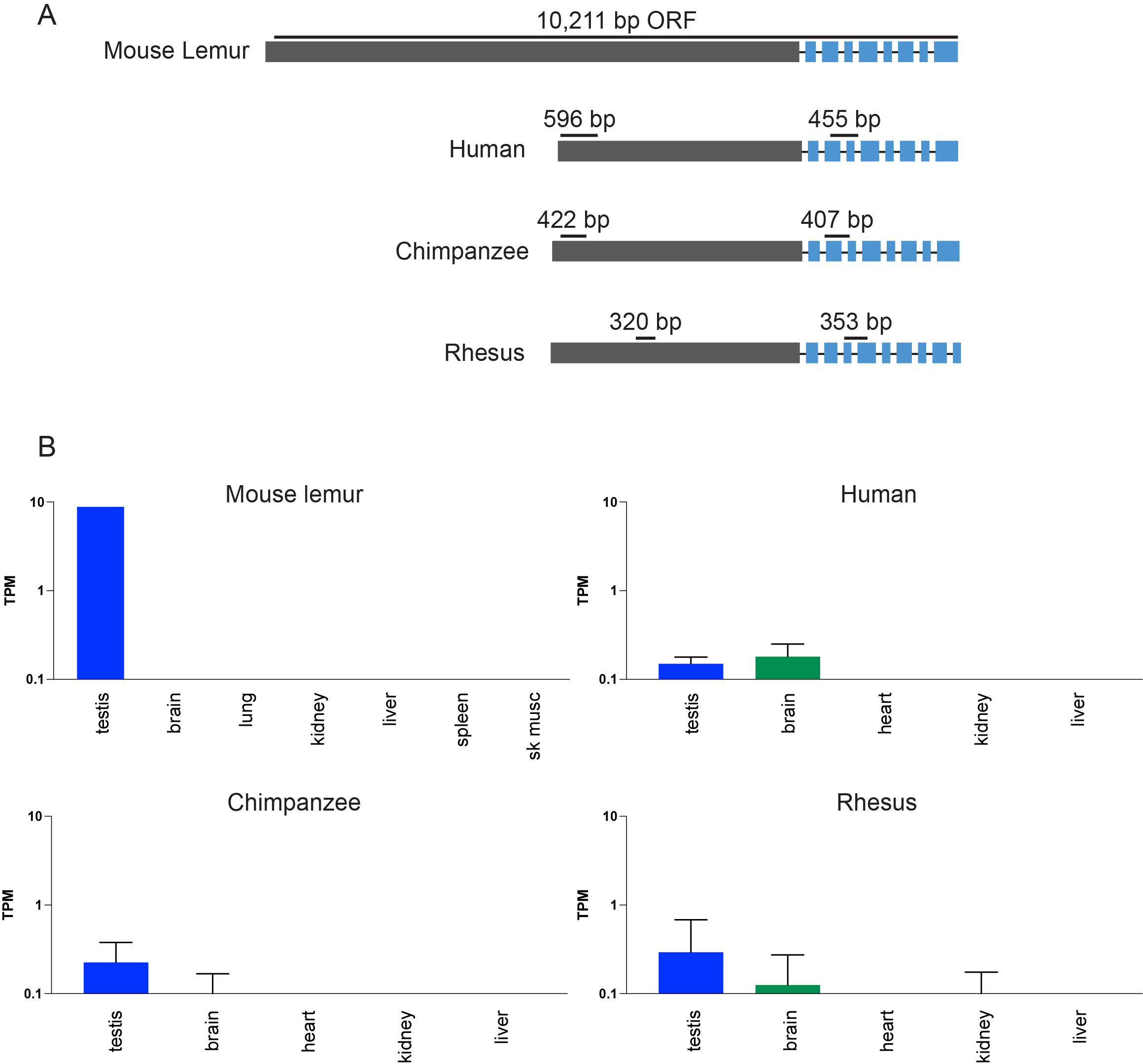
**

**Fig. S2: Phylogenetic analysis of *PRSSLY* nucleotide sequences.** Only conserved trypsin-like serine protease domains were used for alignment. Branch lengths are proportional to substitution rates. Numbers at nodes indicate support from 100 bootstrap replicates.


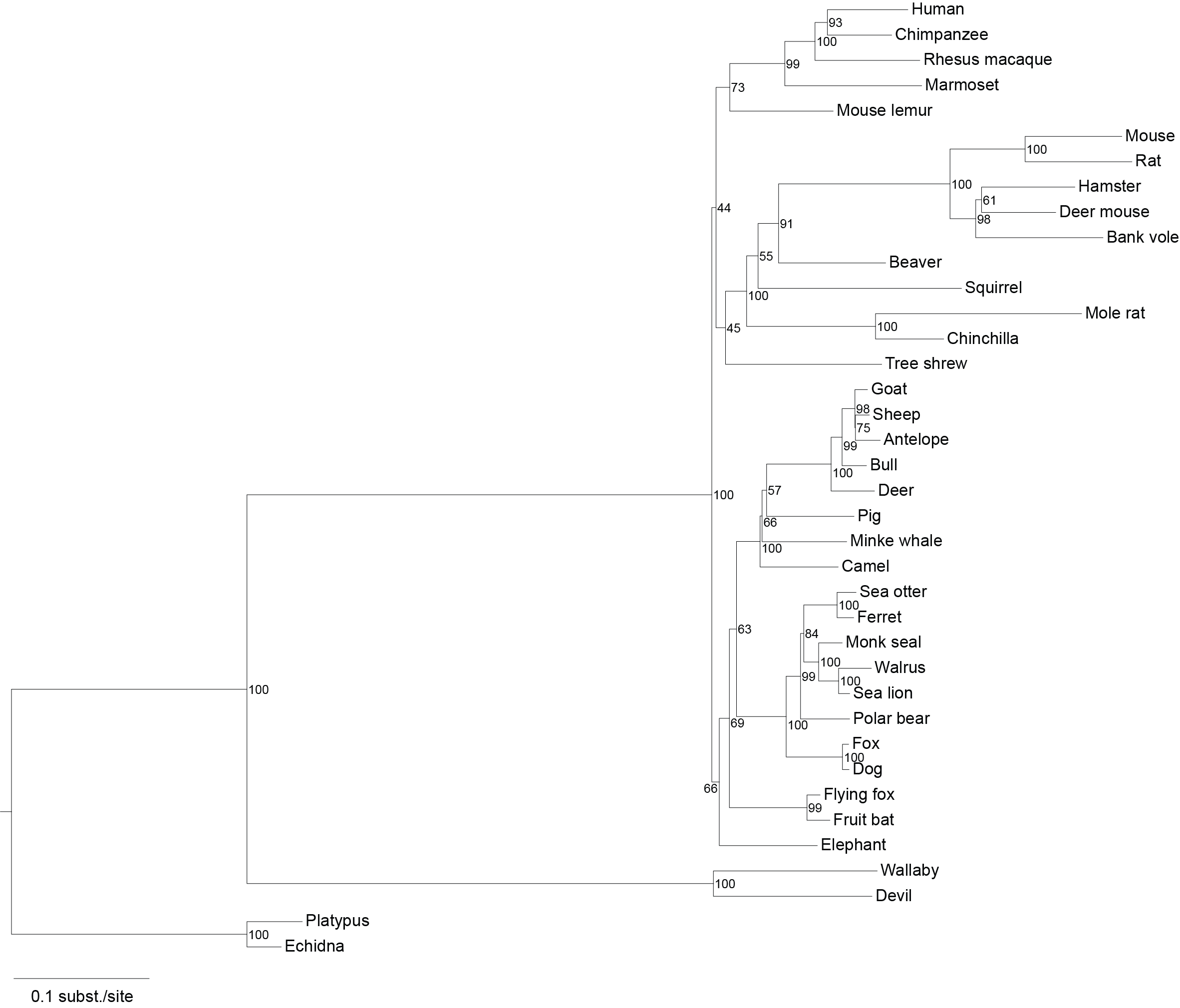


**Fig. S3: Confirmation of X-linkage of *PRSSLY* in marsupials.** *PRSSLY* homologs map to X-chromosome contigs in both the wallaby and Tasmanian devil genome assemblies. However, we wanted to confirm X-linkage using an independent method, so we performed read mapping depth coverage analysis. Separately, for wallaby and Tasmanian devil, we mapped genomic reads derived from male and female animals to the female reference genome using Bowtie version 2.3.4.1 and counted the number of reads mapping to *PRSSLY*-containing contigs, normalized by the total number of reads mapped to the genome. The number of hits in the male (XY) genome is approximately half that in female (XX), confirming X linkage.


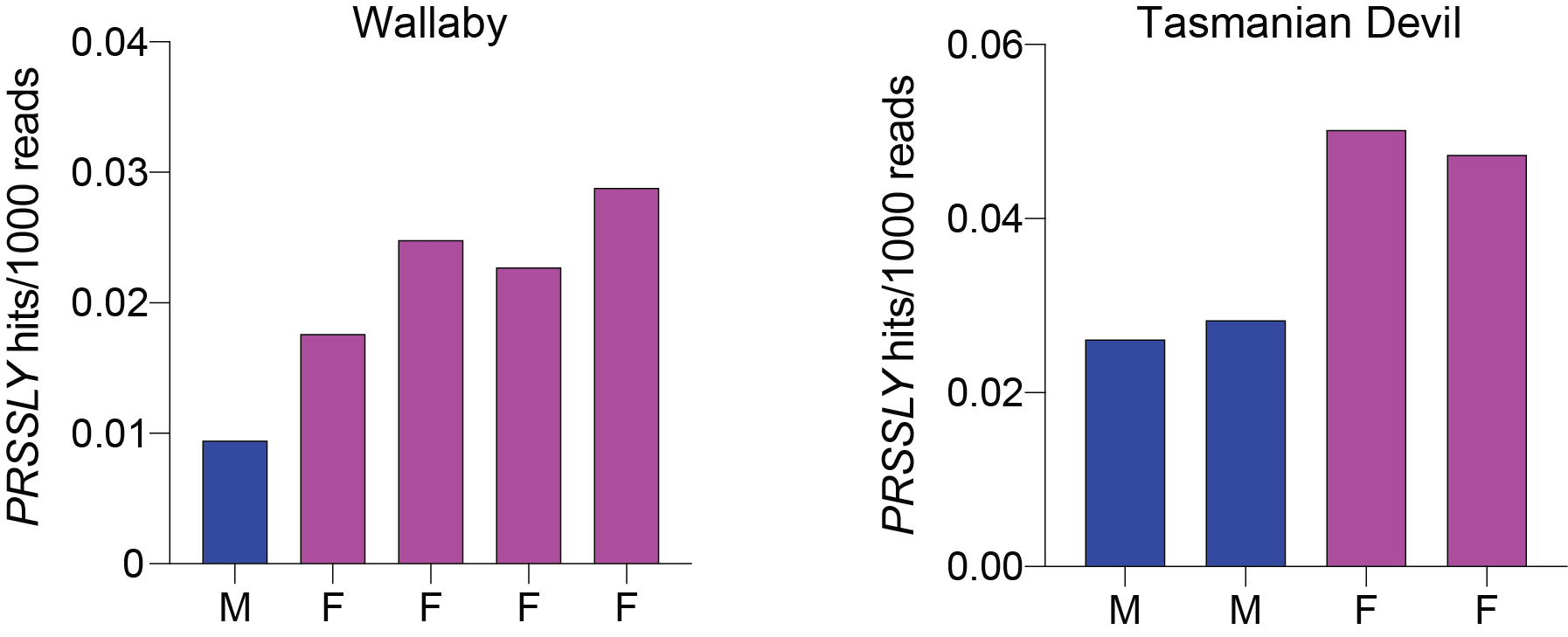


**Fig. S4: Phylogenetic analyses of *PRSS* family amino acid sequences.** Only conserved trypsin-like serine protease domains were used for alignment. Branch lengths are proportional to substitution rates. Top tree includes a subset of *PRSSLY*, *PRSS55*, and *PRSS1/2* sequences; bottom tree includes all *PRSSLY* sequences and a subset of *PRSS55* sequences. Numbers at nodes indicate support from 100 bootstrap replicates. Red arrows indicate strong support for separate clustering of *PRSSLY* in both trees.


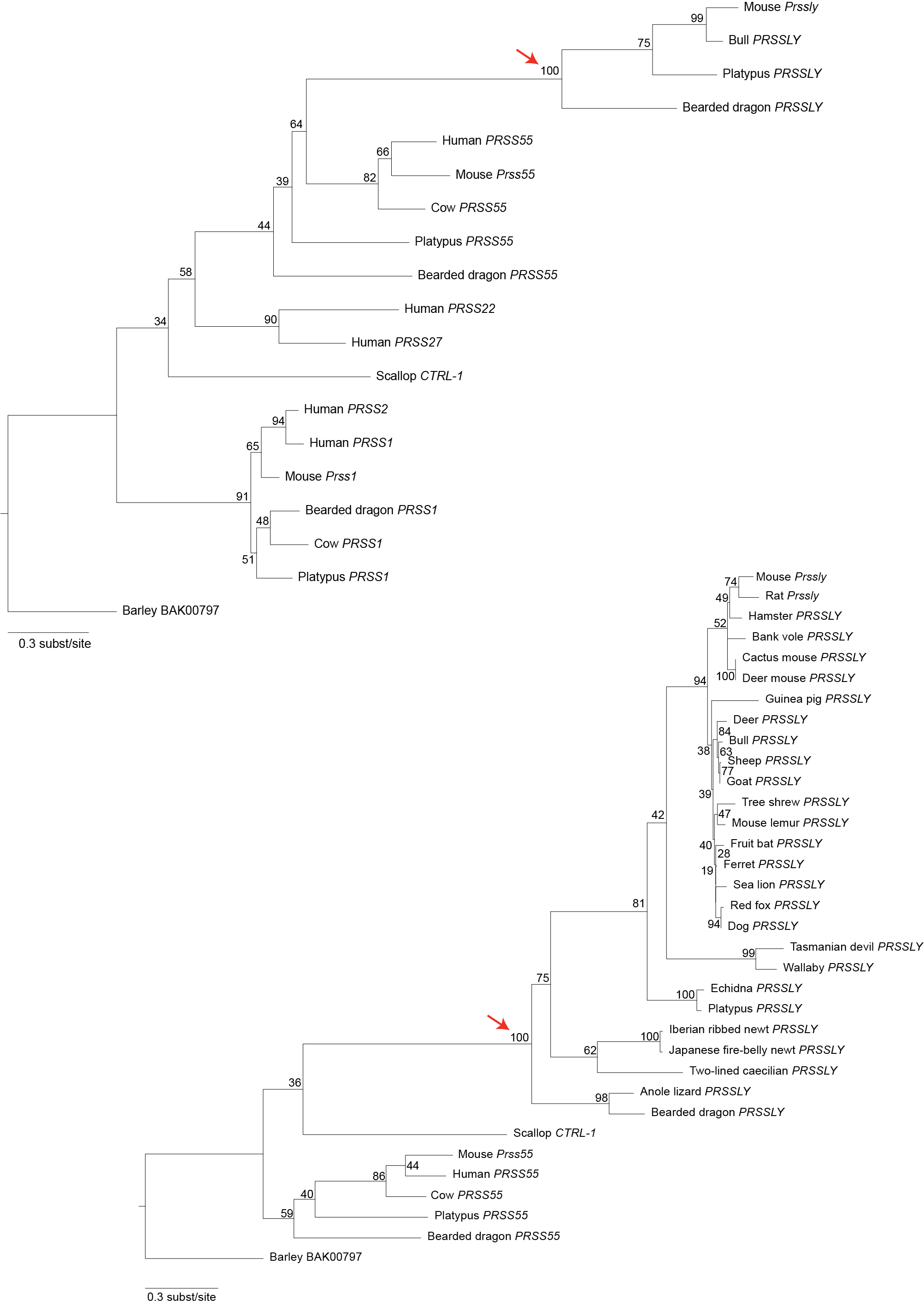


**Fig. S5: Sequence conservation across *PRSSLY* gene sequences.** Dot plot analysis of deer gene sequence (which has the longest open reading frame of all known *PRSSLY* homologs) vs. bull, pig, mouse lemur, ferret, mouse, and tree shrew. Deer is most closely related to bull and pig, which is reflected in relatively high conservation across entire gene. In comparison, conservation is mostly limited to the conserved trypsin-like serine protease domain (blue) in mouse lemur, ferret, rat, and tree shrew. Window size=30, min. % score=60, hash value=8.


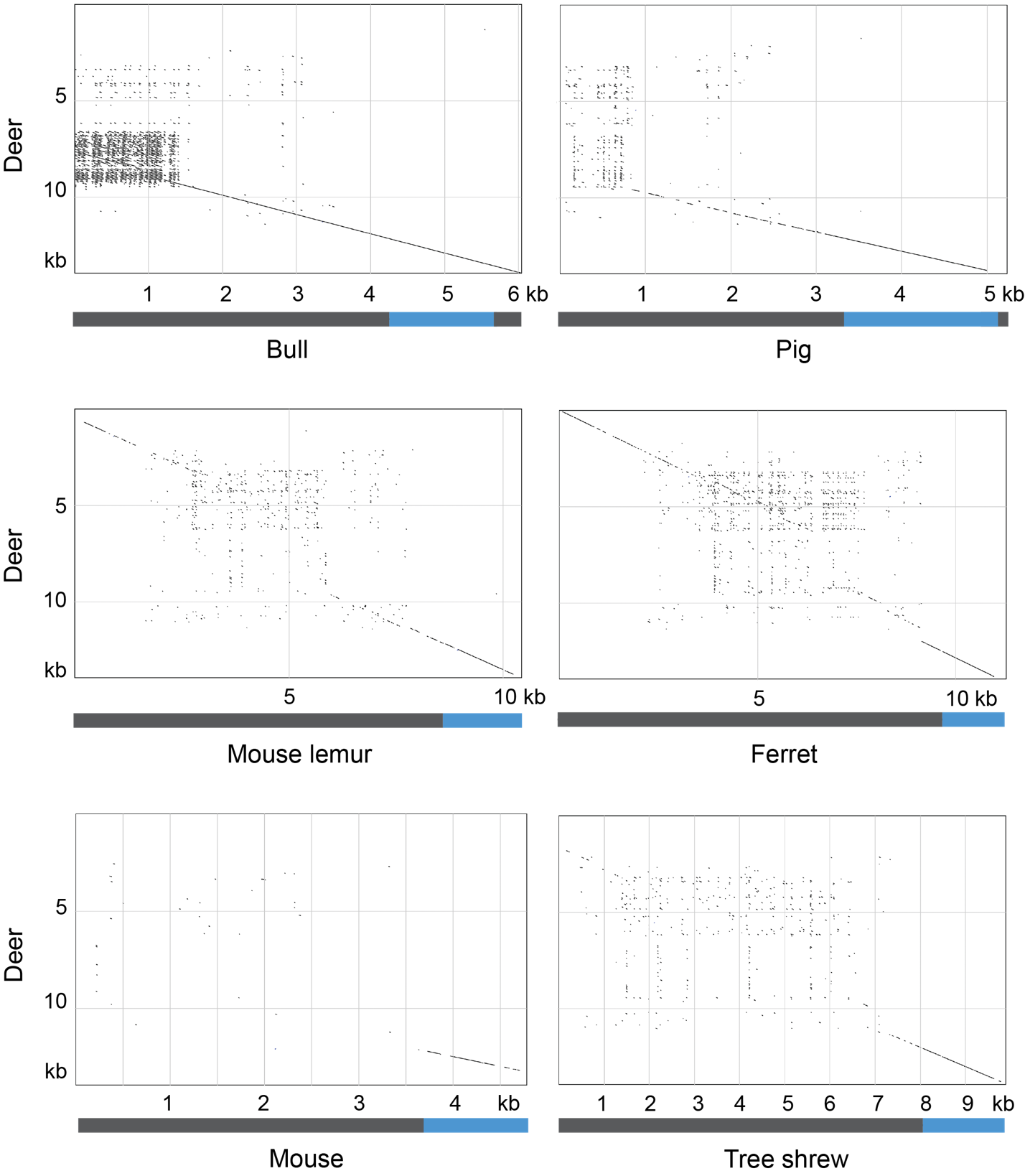


**Fig. S6: Analysis of synonymous (Ks) and non-synonymous (Ka) substitution rates across *PRSSLY.*** Separate alignments were generated for trypsin-like serine protease domain and upstream ORF using sequences of *PRSSLY* and homologs from 27 species (mouse, bull, mouse lemur, tree shrew, rat, hamster, deer mouse, cactus mouse, guinea pig, bank vole, ferret, sea lion, dog, fox, sheep, goat, deer, fruit bat, wallaby, Tasmanian devil, platypus, echidna, anole lizard, bearded dragon, caecilian, fire belly newt, and ribbed newt). For each pairwise alignment, Ka, Ks, and Ka/Ks values were calculated. All values for Ka/Ks (A) and Ka (B) are plotted on the Y axis. The X axis indicates the divergence in millions of years between the two species in each pair. Orange=trypsin-like serine protease domain; Blue=upstream ORF alignments > 1000 bp; Grey=upstream ORF alignments < 1000 bp. Short alignments within the upstream ORF should be considered unreliable, with a few exceptions in alignments between closely related species (platypus – echidna; fire-bellied newt – ribbed newt).


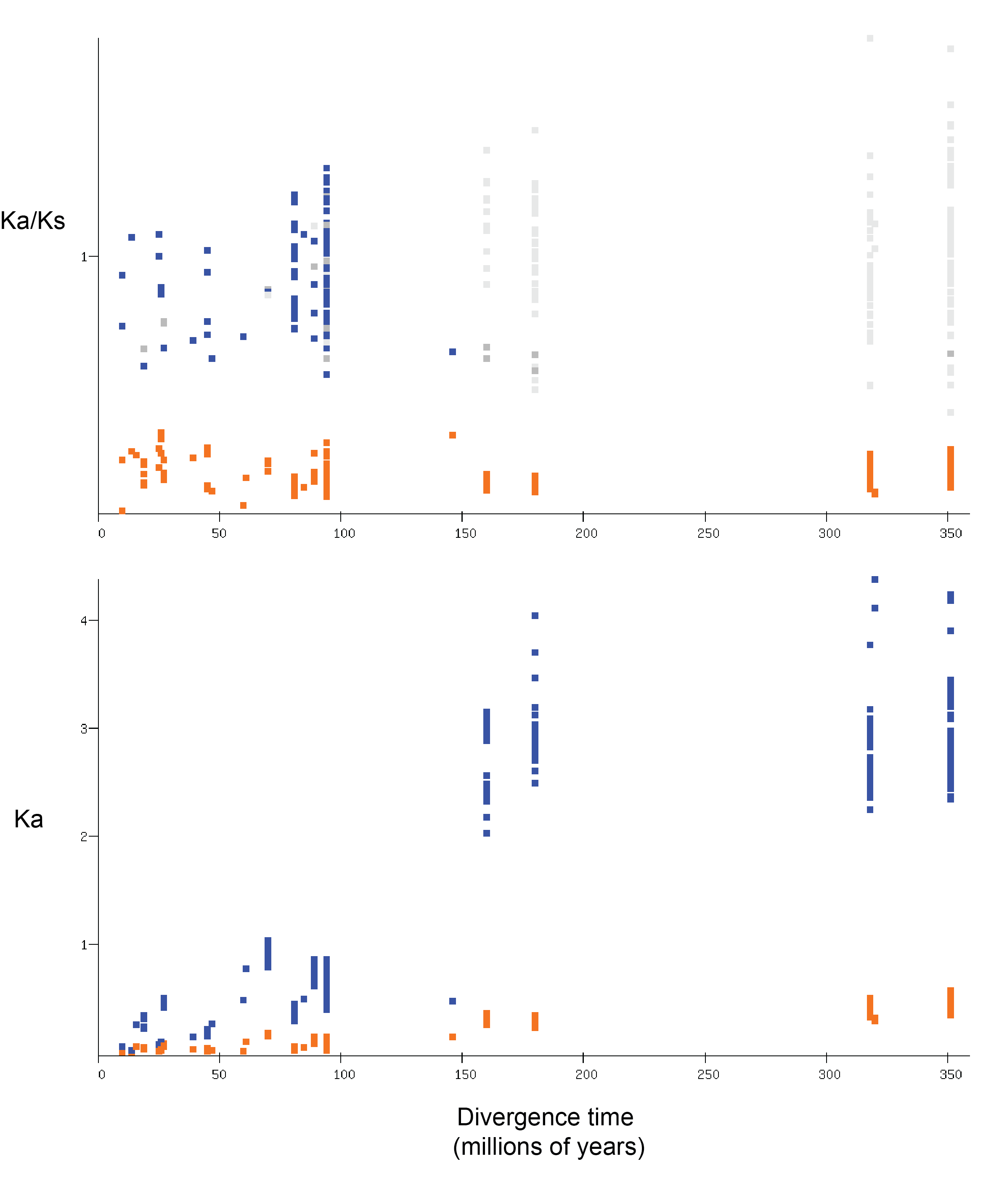


**Fig. S7: Expression of human *PRSS* homologs across tissues.** Only *PRSS* homologs with maximum tpm greater than 1 are shown. Expression levels of *PRSS33, PRSS48,* and *PRSS57* were below 1 tpm in all tissues examined. Source data can be found in Additional File 6. (A) Heat map showing tpm values relative to max tpm for each gene generated using Morpheus (<https://software.broadinstitute.org/morpheus>). (B) Graph of tpm values for each gene and tissue generated using Prism v.9.

A


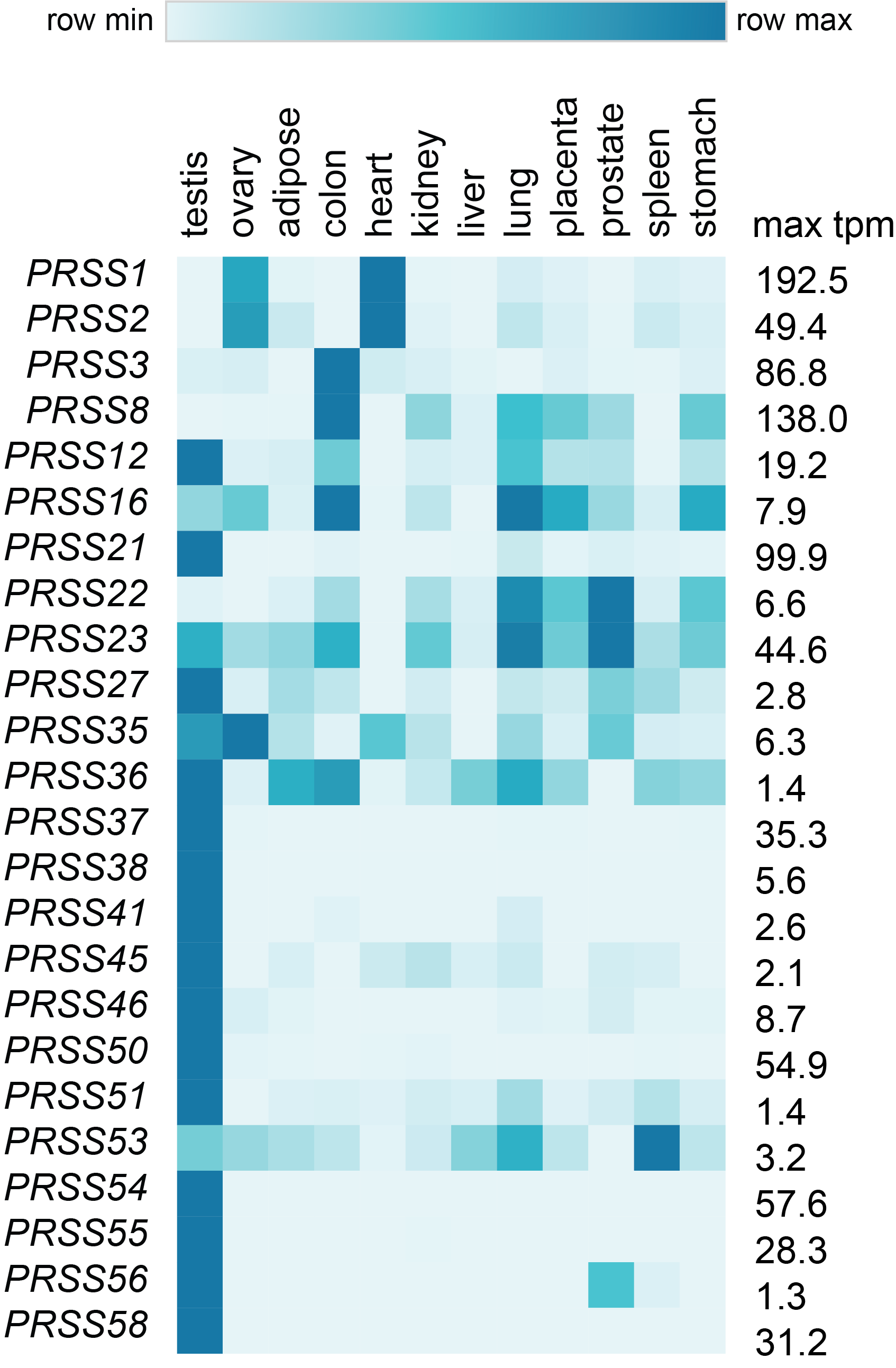


B


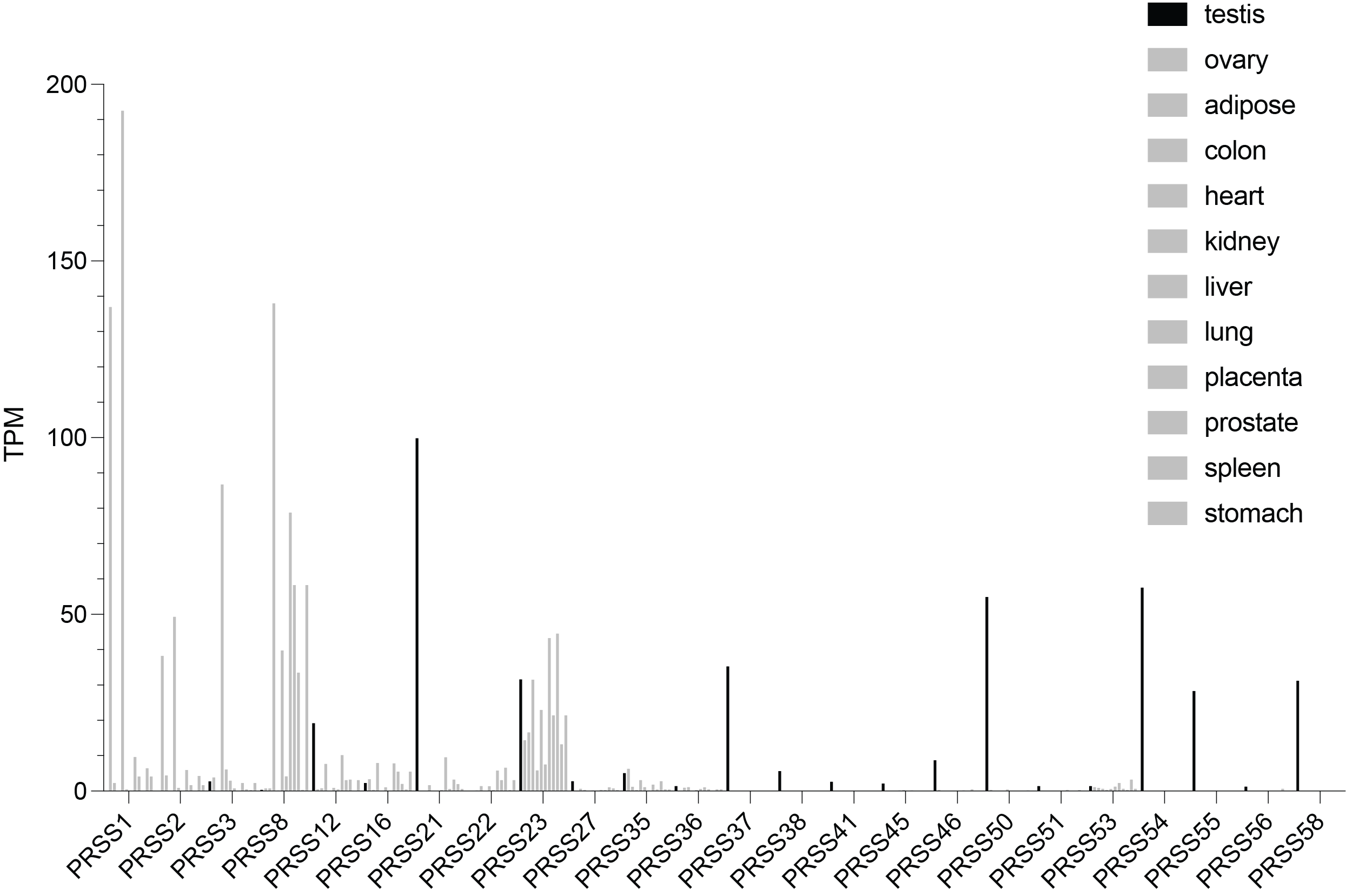


_­_

**Fig. S8: Gene expression analysis of *PRSSLY* in purified male germ cells and germ-cell-depleted testis.** Analysis includes RNA-seq datasets from whole testis and purified germ cell fractions containing pachytene spermatocytes and round spermatids for bull (A) and mouse (B). For mouse, analysis includes datasets derived from embryonic day 12.5 and adult wild-type (WT) and germ-cell-depleted (W/Wv) mice. Expression was estimated in transcript per million (TPM) units. TPM values are plotted on a log10 scale. Details and source data can be found in Additional file 6.

­­­­­­
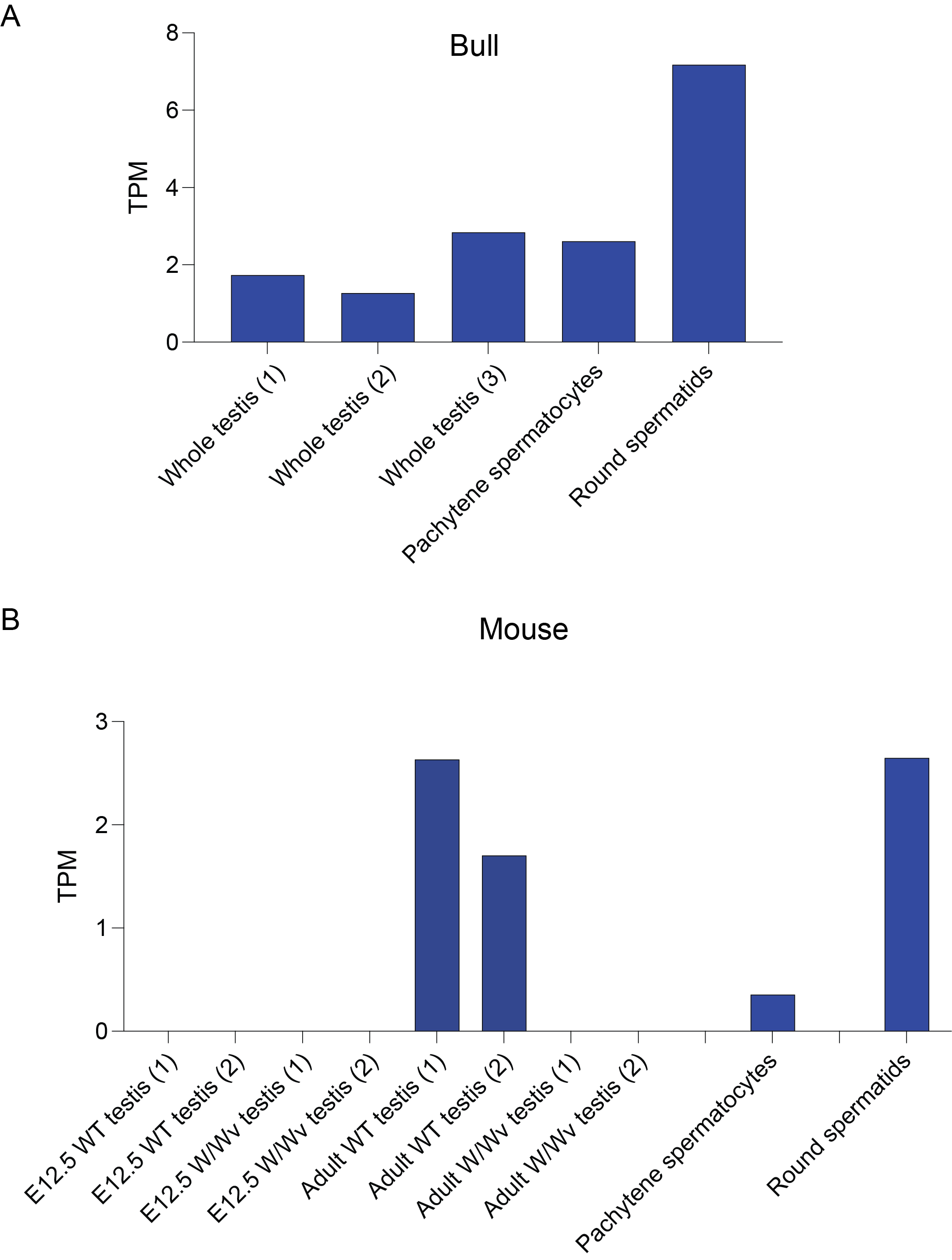


**Fig. S9: Four CRISPR-induced mutations in mouse *PRSSLY*.** Exons are indicated by boxes and are drawn to scale; introns are indicated by lines and are not drawn to scale. Conserved trypsin-like serine protease domains are shaded blue. Red asterisks indicate premature stop codons; purple asterisk indicates splice site disruption; green box indicated retroviral insertion.

**
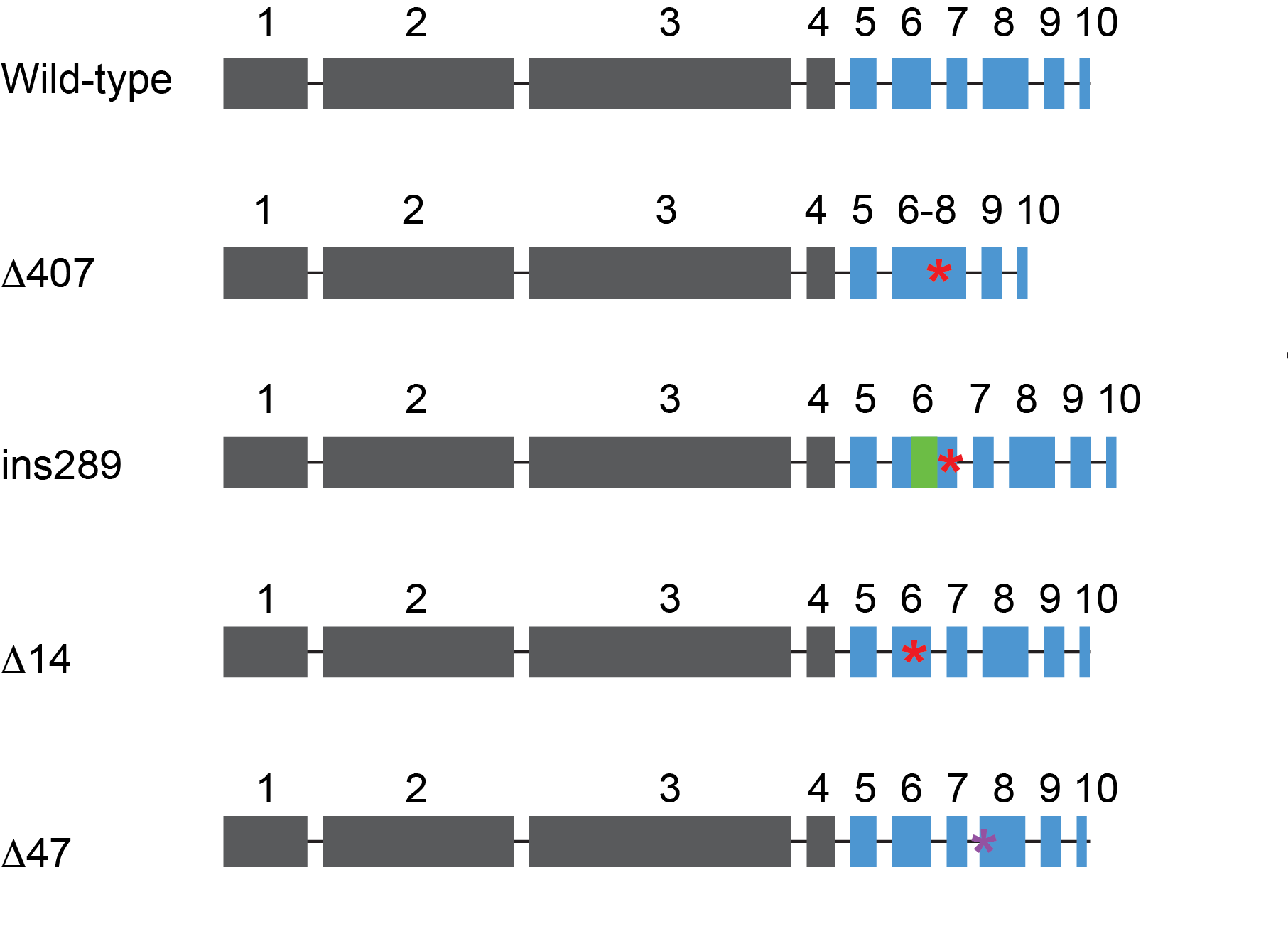
**

**Fig. S10: Testis weights of control and *Prssly* mutant mice.** Testis were collected at P60. Mean and standard deviation are plotted. Each testis is counted separately. For mutants, 3 animals are delta407 and 4 animals are delta47. P-value from unpaired t-test.


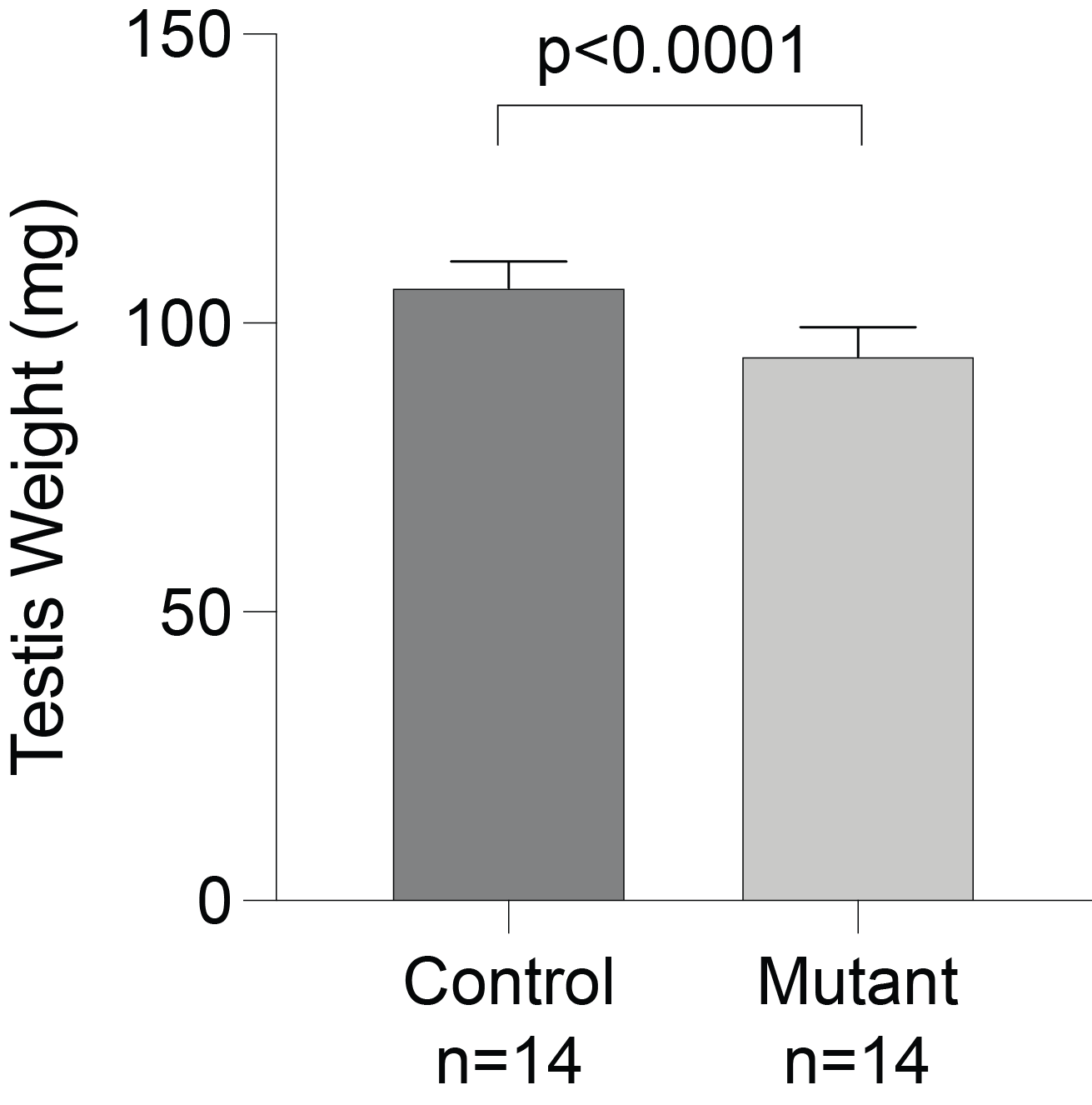


**Fig. S11: Testis histology of control and *Prssly* mutant mice.** Testis samples were collected at P60. Sections were stained with PAS and fixed with Bouin’s. Scale bar = 100μm (10X magnification) and 50μm (40X magnification)


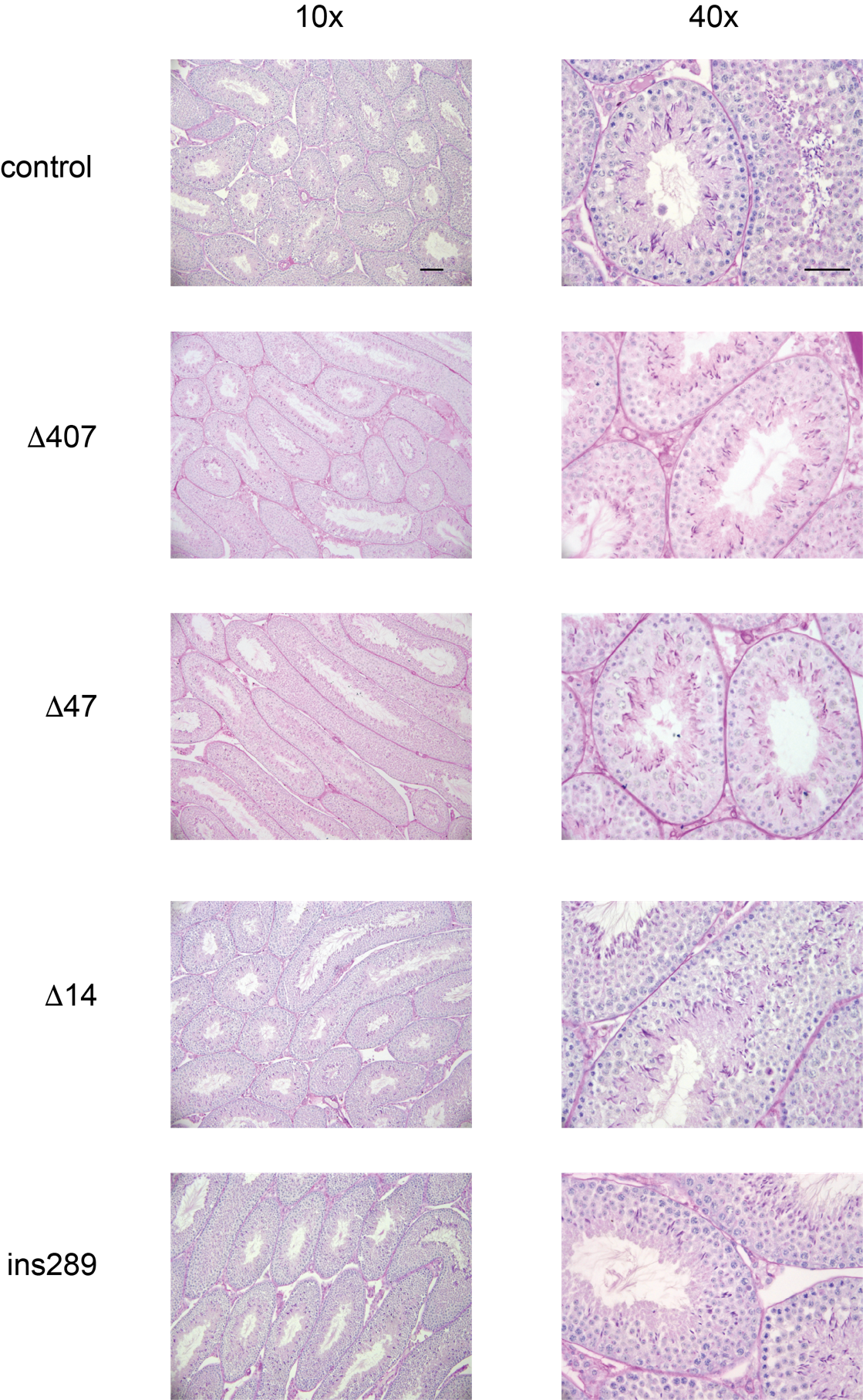


**Fig. S12: Litter sizes in *Prssly* mutants and controls.** P-values are from unpaired t-test; only statistically significant p-values are shown.


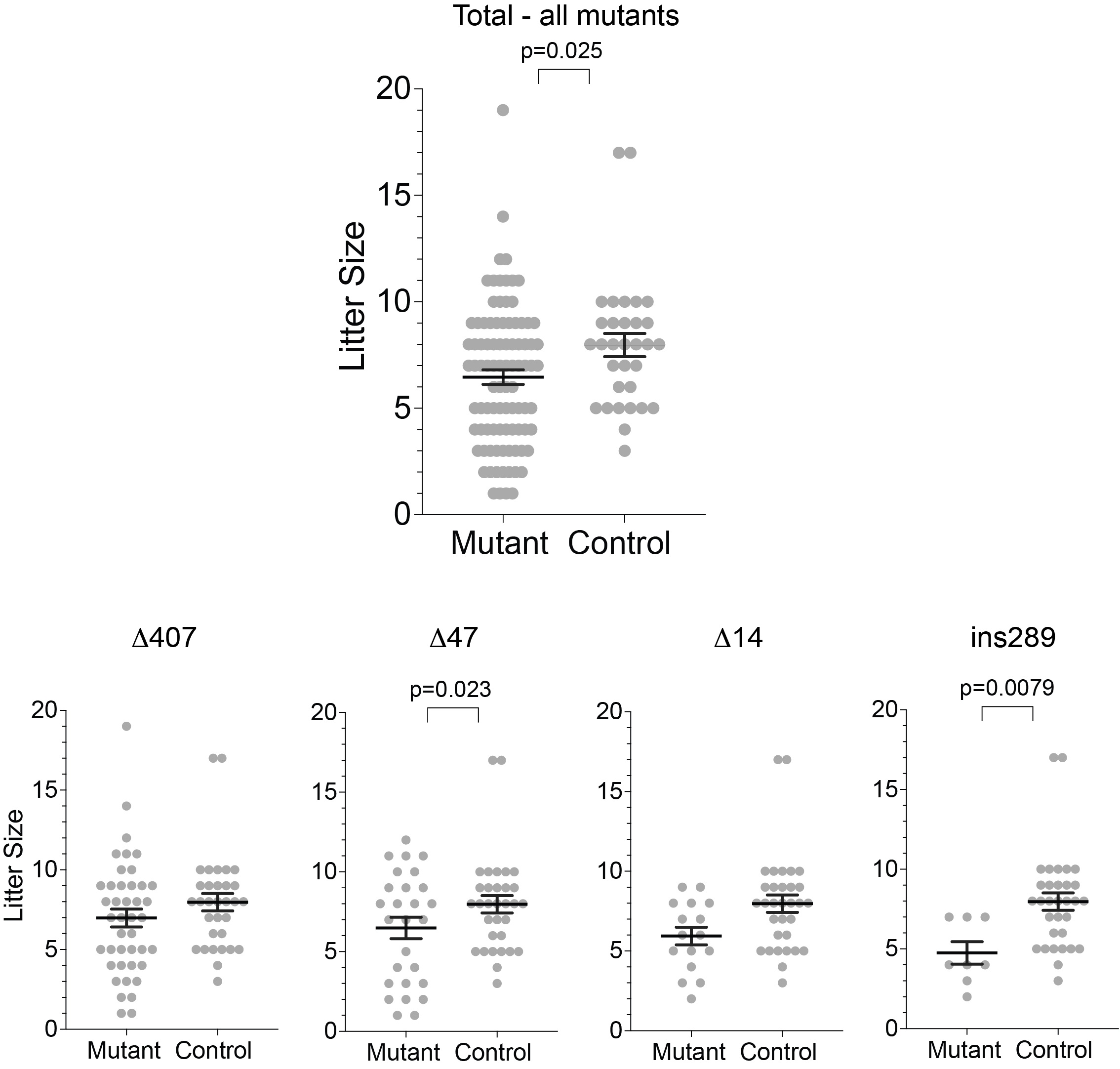

Supplement: Supplementary file 2 — Additional file 2: Fig. S1. Structure and RNA-seq analysis of human, chimpanzee, and rhesus PRSSLY pseudogenes. Fig. S2. Phylogenetic analysis of PRSSLY nucleotide sequences. Fig. S3. Confirmation of X-linkage of PRSSLY in marsupials. Fig. S4. Phylogenetic analyses of PRSS family amino acid sequences. Fig. S5. Sequence conservation across PRSSLY gene sequences. Fig. S6. Analysis of synonymous (Ks) and non-synonymous (Ka) substitution rates across PRSSLY. Fig. S7. Expression of human PRSS homologs across tissues. Fig. S8. Gene expression analysis of PRSSLY in purified male germ cells and germ-cell-depleted testis. Fig. S9. Four CRISPR-induced mutations in mouse PRSSLY. Fig. S10. Testis weights of control and Prssly mutant mice. Fig. S11. Testis histology of control and Prssly mutant mice. Fig. S12. Litter sizes in Prssly mutants and controls. [file 12915_2022_1338_MOESM2_ESM.docx]
