# Supplementary material for: A gene deriving from the ancestral sex chromosomes was lost from the X and retained on the Y chromosome in eutherian mammals
Source: BMC Biol. 2022 Jun 9;20:133. doi: 10.1186/s12915-022-01338-8 (PMC9178871; doi:10.1186/s12915-022-01338-8)
Supplement: Supplementary file 5 — Additional file 5. Sequences used for Ka-Ks analysis of PRSSLY and homologs. [file 12915_2022_1338_MOESM5_ESM.docx]

**Additional file 5: Sequences used for Ka-Ks analysis**

**Trypsin-like serine protease domain**

>MMU Mouse

GAATTTCCCTGGATAGTCTCTGTGCAACTTTCTTACTCACACTTCTGTGCAGGCTCTATACTTAATGAAAGGTGGATTCTTACATCTGCTCGATGTGCCAATTTTGTAAAACGGTCAGAAGCCCTGGCTTTAGTCCAAGTGGGGCTGGTTGATCTTCAAGACCCTACCCAAGGTAAAATCGTAGGTATCCACCGTTCCATGCCATATTTAGGTCCTAGTGGACCATTAGGACCTGGTCTAATCTTCCTGAAGGAGCCCCTTCATTTTCAACCTTTGGTGCTTCCTATTTGCCTGGAGGAGAGTCAGGAGCAAGAGAAACATATCCAGCTATATGACTGCTGGTTACCCAGTTGGTCCCTTATGAGGGGAAGTCCTGGCATCCTGCAAAAAAGGCACCTAAGCATCATGAAAGTCAGCACTTGTGCCAAATTTTGGCCTCAACTGAAAGAGTTCACTTTCTGTGTAGAAGCCAAGAAAGCTATGGGGGAATCTGGTTGTAAGGGTGATCTTGGGGCACCATTGGTATGTCATCTGCGACATAAAGATACATGGATACAGATGGGAATTTTAATTCACTTTGATGAAAACTGCAATAAGCCCTATGTCTTCAGCCATATTAGCCCTTTTATTTTGTGGCTTCAGAGAGTT

>BTA Bull

GAATTCCCTTGGATGGTTTCTGTGCAACTCTCTTTCTCCCATTTCTGTGCTGGTTCTATACTGAATGAACAGTGGATTCTCACTACAGCTAGATGTGCAAATTTCATAAAAAACTCAGAAGCATTGGCCCATGTCCAGGTGGGGCTGATTGATCTTCAAGACCCTGCTCAAGCTCAAACTGTAGGCATTCATCGTGCCATGCCCTACCTGGGCCCTAGAGGACCTTTGGGACCTGGTCTAATCTTCTTGAAGCAACCATTACATTTTCAACCCCTGGTTCTTCCTATCTGCCTGGAGGAGAACCTAGAGCAAGAGAAAAATATACAACTATATGACTGCTGGCTACCTAGTTGGTCCCTCATGAGAGGAAGTCCTGGAATTTTGCAAAAAAGGCATCTGAGCATCCTGCAAGTCATCACATGTGCCCAGTTTTGGCCCAGCCTGAATGAATTTACTTTCTGTGTGGCAGCCAAGAAAGCTATGGGGGAGGCTGGCTGTAAGGGTGACCTGGGGGCACCTCTTATATGTCATCTGCAACAAAAAGACACATGGGTGCAGGTGGGAATCTTGACTCACTTTGATGAACACTGCAGAAAGCCCTATGTCTTCAGCCAAGTGAGCCCTTTCCTTTTCTGGCTCCAGGGAGTT

>MMUR Mouse Lemur

GAATTCCCTTGGATGGTTTCCGTGCAACTCTCTTTCTCTCATTTCTGTGCTGGCTCTATACTGAATGAGCAGTGGATCCTTACTACAGCAAGATGTGCCAATTTCATAAAAAACTCAGAAGCTTTGGCTCTGGTCCAAGTTGGGCTTACTGATCTTCAAGACCCCCCTGCCCATGCTCAGACTATAGGAATACACCGTGCTATGCCTTACCTAGGCCCCAAGGGACCTCTAGGACCTGGGTTGATCTTCCTGAAGAAGCCACTACGTTTTCAACCCCTGGTACTTCCTATCTGCCTGGAGGAGAGTCTGGAACAACAGAAAAAAATACAACTGTATGATTGCTGGCTGCCCAGTTGGTCCCTCATGAGGGGAAGTCCTGGAATTCTTCAAAAAAGACATCTAAGCATTCTGCAAGTCAGCTCTTGTGCTCAATTTTGGCCTAAGCTGAATGAATTCACTTTCTGTGTGGAAGCCAAGAAAGCTATGGGGGAAGCTGGCTGTAAGGGTGACTTGGGGGCACCCCTTGTATGCCATCTACAGCAAAAGGACACATGGGTGCAGGTGGGAATCTTGAGTCACTTTGATGAACATTGCACAAAGCCCTACGTCTTCAGCCAAGTGAGCCCTTTCCTTTTCTGGCTCCAGGGAGTT

>TCH Tree shrew

GAATTTCCCTGGATGGTTTCTATACAACTCTCTTTCTCCCATTTCTGTGCTGGCTCTATATTGAATGTACAATGGATCCTTACAACAGCTAGAGGTGCCAATTTCATAAAAAACTCAGAAGCTCTGGCTCTGGTTCAAGTGGGCATTACAGATCTTCAGGACCCTGTCCAAGCTCAGACTGTAAGCATTCACCATGCTATGCCCTACTTAGGTCCCAAAGGACCTCTGGGGCCTGGACTGATATTCCTGAAGCAGCCTCTACGTTTTCAACCACTGGTACTTCCTATCTGCCTGGAAGAAAGTCTAGGGCAAGAGAGAAATATACAACTATATGACTGTTGGCTACCTAGCTGGTCCCTCATGAGAGGAAGTCCTGGAATTCTGCAGAAAAGGCATCTAAGCATCCTACAAGTCAGTGCTTGTGCCCAGTTTTGGCCCAAGCTGAATGAATTTACTTTCTGTGTGGAAGCCAAGAAGGCTATGGGGGAGGCTGGCTGTAAGGGTGACCTGGGGGCACCTTTAGTGTGCCATTTACAGCAAAAGGACATATGGGTACAGGTGGGAATTTTGAGTCACTTTGATGAACACTGCATAAAGCCTTATGTCTTCAGCCAAGTGAGCCCTTTCATTTTCTGGCTCCAGGGAGTT

>RNO Rat

GAATTTCCTTGGATGGTTTCTGTGCAACTTTCTTACTCCCACTTCTGTGCAGGCTCCATACTTAATGAAAAGTGGATCCTTACGTCTGCTCGATGTGCCAATTTTGTAAAACGGTCAGAAGCTCTGGCTTTAGTCCAAGTGGGGCTGGTTGATCTTCAGGACGCTACACAGGGTGAAATCGTAGGGATTCACCGTTCCATGCCATATTTAGGTTCCAGTGGACCATTGGGACCTGGCCTGCTCTTGTTGAAGGAGCCCCTACAGTTTCAACCTTGGGTGCTTCCTATTTGCTTGGTGGAGAGTCTGGATCAGGAGAGACATATCCAGCTGTATGACTGCTGGTTACCCAGTTGGTCCCTTATGAGGGGAAGTCCTGGTATCCTGCAAAAAAGGCACCTGAGCATCATGCAAATCAGCCCTTGTGACAAATTTTGGCCTCAACTGAATGAGTTCACTTTCTGTGTAGAAGCCAAGAAAGCTATGGGGGAATCTGGCTGCAAGGGTGATCTTGGGGCACCTTTGGTCTGTCATCTGCAACATAAAGACACGTGGGTGCAGATGGGGATTTTAATTCACTTTGATGAACAGTGCAAAAAGCCCTATGTCTTCAGCCACGTTAGTCCTTTCATTTCCTGGCTCCAGAGAGTC

>MAU Hamster

GAATTCCCCTGGATGGTTTCTGTGCAACTCTCTTATTCCCACTTCTGTGCTGGCTCTATACTTAATGAAAATTGGGTTCTTACCTCTGCTCGATGTGCCAATTTTATAAAACGATCAGAATCTATGGCTTTAGTCCAAGTTGGACTGGTTGATCTTCAAGACCCTAGCCAAGGTGAAACTGTAGGTATACATCGTTCCATGCCATACTTAGGTCCGAGTGGACCTTTAGGACCAGGCCTAATATTGCTGAATGAGCCACTGCATTTTCAACCTTTGGTGCTTCCAATTTGTCTGGAAGAAAGTCAAGAACAAGAAAGGCACATACAATTGTATGACTGCTGGTTACCCAGCTGGTCCCTCATGAGGGGAAGTCCTGGTATCCTGCAAAAAAGGCACCTCAGCATCATGCAAGTCAGTACATGTGCCAAATTTTGGCCTCAGCTGAATGAGTTCACTTTCTGTGTAGAGGCCAAGAAAGCTATGGGGGAATCTGGCTGTAAGGGTGATCTTGGGGCACCTTTAGTGTGTCGTCTAAAACAAAAAGACAAATGGGTGCAGGTTGGAATTTTAATTCACTTCGATGAACATTGCACAAAGCCCTATGTCTTCAGCCAAGTTAGCCCTTTTGTTTTCTGGATCCAGAGAGTT

>PMA Deer mouse

GAATTCCCCTGGATGGTTTCTGTGCAGCTTTCTTACTCCCACTTCTGTGCTGGCTCTATACTTAATGAAAAGTGGATCCTCACTTCTGCTCGATGTGCCAATTTCATAAAACGTTCAGAATCTCTGGCTTCAGTCCAAGTGGGGCTACTTGATCTTCAGGACCCTACGCAAGGTGAAACTGTGGGTATCCATCGTTCTATGCCATACATAGGTCCCAGGGGACCTTTAGGACCAGGCCTGATCTTCCTGAAGGAGCCACTGCATTTTCAACCTTTGGTGCTGCCTATTTGCTTGGAAGAAAGTCAAGAGCAAGAAACACATATACAACTATATGACTGCTGGTTACCAAGCTGGTCCCTTATGAGAGGAAGTCCTGGTATCCTACAAAAAAGGCACCTCAGCATCATGCAAGTCAGCACCTGTGCCAAATTTTGGCCTCAGCTAAATGACTTCACTTTCTGTGTGGAGGCCAAGAAAGCTATGGGGGAATCTGGCTGTAAGGGTGATCTTGGGGCACCTTTGGTATGTCATCTAAAACAAAAAGACACATGGGTGCAGGTGGGAATTTTAATTCACTTCGATGAACACTGCAAAAAGCCCTATGTTTTCAGCGAAGTTAGCCCTTTTGTTTTCTGGCTCCAGAGAGTT

>PER Cactus mouse

GAATTCCCCTGGATGGTTTCTGTGCAGCTTTCTTACTCCCACTTCTGTGCTGGCTCTATACTTAATGAAAAGTGGATCCTCACTTCTGCTCGATGTGCCAATTTCATAAAACGTTCAGAATCTCTGGCTTCAGTCCAAGTGGGGCTACTTGATCTTCAGGACCCTACCCAAGGTGAAACTGTGGGTATCCATCGTTCTATGCCATACATAGGTCCCAGGGGACCTTTAGGACCAGGCCTGATCTTCCTGAAGGAGCCATTGCATTTTCAACCTTTGGTGCTTCCTATTTGTTTGGAAGAAAGTCAAGAGCAAGAAACACATATACAACTATATGACTGCTGGTTACCAAGCTGGTCCCTTATGAGAGGAAGTCCTGGTATCCTGCAAAAAAGGCACCTCAGCATCATGCAAGTCAGCACCTGTGCCAAATTTTGGCCTCAGCTGAATGACTTCACTTTCTGTGTGGAGGCCAAGAAAGCTATGGGGGAATCTGGCTGTAAGGGTGATCTTGGGGCACCTTTGGTATGTCATCTAAAACAAAAAGACACATGGGTGCAGGTGGGAATTTTAATTCACTTCGATGAACACTGCAAAAAGCCCTATGTTTTCAGCGAAGTTAGCCCTTTCGTTTTCTGGCTTCAGAGAGTT

>CPO Guinea pig

GAATTTCCCTGGATGGTTTCAATACAATTGTCTTTCTCCCATTTCTGTGCTGGCTCTATTCTGAATGAACACTGGATCCTTACCTCCGCTAGATGTGCCAATTTTGTAAAAAGTTCAGAGACACTGTCCATGGTGCAAGTGGGGCTTATTGACCTCCAGGGACCAGCTCAAACTCATATTGTGGGCATTCACCGTGCCATGCCCTATATAGGTCCCTCAGGGCCCCTAGGACCCGGCCTGATCTTTCTGAAGGAGCCACTACATCTCCAACCCCTCGTGCTTCCTATTTGTCTGGAGGATAACATGGAGCAAGAGAAAAATAAGCCACTATATGATTGCTGGCTATCTAGCTGGTCCCTTCTGAGGGGAAGACCTGGAATTCTGCAAAAAAGACACCTAAGTATTCTGCAAACCAGCACTTGTGAAGAATATTGGCCCACAGCAAGTGAATTTACTTTCTGTGTGGAAGCCAAGAAAGCTACTGGGGAAGATGATTGTAAGGGTGACGTAGGGGCACCTCTAGTGTGCCGTCTACCACGAAAGAACACTTGGGTGCAGGTGGGAATATTGAGTCACTTTGATGAACATTGCATAAAGCCCTATGTCTTCATCCAAGTGAGCCCTTTCATTTTCTGGCTTCGTGGAGTA

>MGL Bank vole

GAATTCCCATGGATGGTTTCTGTGCAGCTCTCTTACTCCCACTTCTGTGCTGGTTCTATTCTTAATGAAATGTGGGTCCTCACTTCTGCCCGATGTGCCAATTTTATAAAACGGTCAGAATCTCTGGCTTTAGTCCAAGTGGGTCTGATTGATCTTCAGGATCCTACACAAGGTGAAACTGTTGGTATCCAGCGTTCTGTGCCATACTTAGGTCCCTGGGGACCTTTAGGACCAGGCCTATTCTTCCTGAAGGAGCCAATACATTTTCAACCTTTGGTGCTTCCTATTTGCCTGGAAGAAAGTCAAGAACAAGAAAGACACATACAACTATATGACTGCTGGTTACCTAGCTGGTCCCTCATGAGAGGAAGTCCTGGTATCTTGCAAAAAAGGCACCTCAGCATCATGCAAGTCAGCACCTGTTCTAAATTTTGGCCTCAACTGAATGAGTTCACTTTCTGTATAAAGGCCAAGAAAGCTATGGGGGAATCTGGCTGTAAGGGTGATCTTGGGGCACCTTTGGTTTGTCATCTAAAACAAAAAGACACATGGATGCAGGTGGGAATTTTAATTCAATTTGATGAACATTGCACAAAGCCCTATGTCTTCAGTCAAGTTAGACCTTTCGTTTTCTGGCTCCAGAGAGTT

>MPU Ferret

GAATTTCCTTGGATGGTTTCTGTGCAACTCTCTTTCTCCCATTTCTGTGCTGGTTCTATACTGAATGAACAGTGGATCCTTACTACAGCTAGATGTGCAAATTTCATAAAAAACTCAGAAGCACTGGCCCTGGTCCAAGTGGGACTTATTGATCTTCAGGAGCTTGCTCAAGCTCAAACTGTAGGCATTCATCGTGCCATGCCCTACCTAGGTCCCAAGGGACCTTTGGGTCCTGGGCTAATCTTCCTGAAACAGCCACTACATTTCCAACCACTGGTGCTTCCTATTTGCCTAGAGGAGAACCTGGAGCAAGAGAAAAATATACAGCTGTATGACTGCTGGCTACCCAGTTGGTCCCTCATGAGAGGAAGTCCTGGAATTCTGCAAAAAAGACACCTAAGCATTCTGCAAGTCAGCACATGTGCCCAGTTTTGGCCCAAGCTGAATGAATTCACTTTCTGTGTGGAAGCCAAGAAAGCTATGGGCGAGGCTGGCTGTAAGGGTGACTTAGGGGCACCTCTGGTGTGCCATCTACAACAAAAGGACACATGGGTGCAGGTGGGAATTTTGAGTCACTTCGATGAACATTGCACAAAGCCCTACGTCTTCAGCCAAGTGAGCCCGTTCCTTTTTTGGCTTCAAGGAGTT

>ZCA Sea lion

GAATTTCCTTGGATGGTTTCCGTGCAACTCTCTTTCTCCCATTTCTGTGCTGGCTCTGTACTGAATGAACAGTGGATCCTTACCTCAGCTAGATGTGCAAATTTCATAAAGACCTCAGAAGCACTAGCCCTGGTCCAAGTGGGGCTTACTGATCTTCAGGAGCCTGCTCAAGCTCAAACTGTAGGCATTCACCGTGCCATGCCCTACCTAGGTCCCAAGGGACCTTTGGGTCCTGGGCTGATCTTCCTGAAACAGCCCCTACATTTTCAACCCTTGGTGCTTCCTATCTGTCTAGAGGAGAACCTGGAGCAAGAGAAAAATATACAGCTGTATGACTGCTGGCTACCCAGTGGGTCCCTCATGAGAGGAAGTCCTGGAATTCTGCAGAAAAGGCATCTAAAAATACTGCAAGTCAGCACATGTGCCCAGTTTTGGCCCAAGCTGAATGAGTTCACTTTCTGTGTGGAAGCCAAGCAAGCTCTTGGCGAGACTGGCTGTAAGGGTGACTTAGGGGCACCtCTGGTATGCCATCTACAACAAAAGAACACATGGGTGCAGGTGGGAATTTTGAGTCACTTCGATGAACATTGCACAAAGCCCTACGTCTTCAGCCAAGTGAGCCCTTTCCTTTTTTGGCTTCAGGGAGTC

>CFA Dog

GAATTTCCCTGGATGGTTTCTGTGCAACTCTCTTTCTCCCATTTCTGTGCTGGCTCTATACTGAATGAACAGTGGATCCTTACCACAGCTAGATGTGCAAATTTCATAAAAAACTCAGAAGCACTAGCCCTGGTCCAAGTGGGGCTTGTTGATCTTCAAGAGCCTGCTCAAGCTCAAACTGTAGGCATTCACCGTGCCATGCCCTACCTAGGTCCCAAGGGACCTCTAGGCCCTGGGCTAATCTTCCTGAAGCAGCCACTACACTTTCAACCATTGGTGCTTCCTATCTGCCTGAAGGAGAATGTGGAGCAAGAGAAAAGTATACAGCTTTATGACTGCTGGCTACCCAGTTGGTCCCTCATGAGAGGAAGTCCTGGGATTCTGCAAAAAAGGCACCTAAGTATACTGCAAGTCAGCACATGTGCCCAGTTTTGGCCCAAGCTGAATGAATTCACTTTCTGTGTGGAAGCCAAGAAAGCTATGGGGGAGGCTGGCTGTAAGGGTGACTTAGGGGCACCTCTGGTATGCCATCTACATCAAAAGGATACATGGGTACAGGTGGGAATTTTGAGTCACTTCGATGAACATTGCACAAAGCCCTACGTCTTCAGTCAAGTGAGCCCTTTCCTTTTTTGGCTTCAGGGAGTT

>VUL Fox

GAATTTCCCTGGATGGTTTCTGTGCAACTCTCTTTCTCCCATTTCTGTGCTGGCTCTATACTGAATGAACAGTGGATCCTTACCACAGCTAGATGTGCAAATTTCATAAAAAACTCAGAAGCACTAGCGCTGGTCCAAGTGGGGCTTGTTGATCTTCAAGAGCCTGTTCAAGCTCAAACTGTAGGCATTCACCGTGCCATGCCCTACCTAGGTCCCAAGGGACCTCTAGGCCCTGGGCTAATCTTCCTGAAGCAGCCACTACACTTTCAACCATTGGTGCTTCCTATCTGCCTGAAGGAGAATGTGGAGCAAGAGAAAAGTATACAGCTTTATGACTGCTGGCTACCCAGTTGGTCCCTCATGAGAGGAAGTCCTGGGATTCTGCAAAAAAGGCACCTAAGTATACTGCAAGTCAGCACATGTGCCCAGTTTTGGCCCAAGCTGAATGAATTCACTTTCTGTGTGGAAGCCAAGAAAGCTATAGGGGAAGCTGGCTGTAAGGGTGACTTAGGGGCACCTCTTGTATGCCATCTACATCAAAAGGATACATGGGTACAGGTGGGAATTTTGAGTCACTTCGATGAACATTGCACAAAGCCCTACGTCTTCAGTCAAGTGAGCCCTTTCCTTTTTTGGCTTCAGGGAGTT

>OAR Sheep

GAATTCCCTTGGATGGTTTCTGTGCAACTCTCTTTCTCCCATTTTTGTGCTGGTTCTATACTGAATGAACAATGGATTCTCACTACAGCTAGGTGTGCAAATTTCATAAAAAACTCAGAAGCACTGGCCCATGTCCAGGTGGGGCTTATCGATCTTCAAGACCCTGCTCAAGCTCAAACTATAGGCATTCATCGTGCCATGCCCTACCTGGGCCCTAGAGGACCTCTGGGGCCTGGTCTAATCTTCTTGAAGCAACCATTACATTTTCAACCCCTGGTTCTTCCTATCTGCCTGGAGGAGAGCCTAGAGCAAGAGAAAAATATACAACTGTATGACTGCTGGCTACCCAGTTGGTCCCTCATGAGAGGAAGTCCTGGAATTTTGCAAAAAAGGCACCTGAGCATCCTGCAAGCCATCACATGTGCCCAGTTTTGGCCCAAACTGAATGAATTTACTTTCTGTGTGGCAGCCAAGAAAGCTATGGGGGAGGCTGGCTGTAAGGGTGACCTGGGGGCACCTCTTGTGTGTCATCTGCAACAAAAAGACACATGGGTGCAGGTGGGAATTTTGACTCACTTTGATGAACACTGCACAAAGCCCTACGTCTTCAGCCAAGTGAGCCCTTTCCTTTTCTGGCTCCAGGGAGTT

>CHI Goat

GAATTCCCTTGGATGGTTTCTGTGCAACTCTCTTTCTCCCATTTTTGTGCTGGTTCTATACTAAATGAACAATGGATTCTCACTACAGCTAGATGTGCAAATTTCATAAAAAACTCAGAAGCACTGGCCCATGTCCAGGTGGGGCTTATAGATCTTCAAGACCCTGCTCAAGCTCAAACTGTAGGCATTCATCGTGCCATGCCCTACCTGGGCCCTAGAGGACCTCTGGGACCTGGTCTAATCTTCTTGAAGCAACCATTACATTTTCAACCCCTGGTTCTTCCTATCTGCCTGGAGGAGAACCTAGAGCAAGAGAAAAATATACAACTGTATGACTGCTGGCTACCCAGTTGGTCCCTCATGAGAGGAAGTCCTGGAATTTTGCAAAAAAGGCACCTGAGCATCCTGCAAGCCATCACATGTGCCCAGTTTTGGCCCAAACTGAATGAATTTACTTTCTGTGTGGCAGCCAAGAAAGCTATGGGGGAGGCTGGCTGTAAGGGTGACCTGGGGGCACCTCTTGTGTGTCATCTGCAACAAAAAGACACATGGGTGCAGGTGGGAATTTTGACTCACTTTGATGAACACTGCACAAAGCCCTACGTCTTCAGCCAAGTGAGCCCTTTCCTTTTCTGGCTCCAGGGAGTT

>OVI Deer

GAATTCCCTTGGATGGTTTCTGTGCAACTCTCTTTCTCCCATTTCTGTGCTGGTTCTATCCTGAATGAACAGTGGATTCTCACTACAGCTAGATGTGCAAATTTCATAAAAAACTCAGAAGCAGTGGCCCATGTTCAGGTGGGGTTTATTGATTTTCAAGATCCTGCTCAAGCTCAAACTGTAGGCATTCATCGTGCCATGCCCTACCTGGGTCCTAGAGGACCGCTGGGACCTGGTCTAGTCTTCTTGAAGAAACCATTACATTTTCAACCTCTGGTTCTTCCTATCTGCCTGGAGGAGAACATAGAGCAAGAGAAAAATATACAACTATATGACTGCTGGCTACCCAGCTGGTCCCTCATGAGAGGAAGTCCTGGAATTTTGCAAAAAAGGCACCTGAGCATCCTGCAAGTCATCACATGTGCCCAGTTTTGGCCCAAACTGAATGAATTTACTTTCTGTGTGGAAGCCAAGAAAGCTATGGGGGAGGCTGGCTGTAAGGGTGATCTGGGGGCACCTCTTGTGTGTCATCTTCAACAAAAAGACATTTGGGTGCAGGTGGGAATCTTGACTCACTTTGATGAACACTGCACAAAGCCCTACGTCTTCAGCCAAGTGAGCCCTTTCCTTTACTGGCTCCAGGGAGTT

>RAE Fruit bat

GAATTCCCTTGGATGGTTTCAGTGCAACTCTCTTTCTCTCATTTCTGTGCTGGTTCTATACTGAATGAACAGTGGATCCTTACCACAGCTAGATGTGCGAATTTCATAAAAAATTCAGAAGCCTTGGCCCTGGTCCAGGTGGGACTTATTGATCTTCAGGAACCTACTCAAGCTCAGACTGTAGGCATTCACCGTGCCATGCCCTATGTAGGTCCTAAGGGACCTTTGGGACCTGGACTAATCTTCCTGAAGCAGCCACTGCATTTTCAACCCCTAGTGCTTCCTATCTGCCTGGAGGAGAGCCTGGAGCAAGAGAAAAATATACAACTGTATGACTGCTGGCTACCCAGTTGGTCTCTTATGAGGGGAAGTCTTGGAATTCTGCAGAAAAGGCACCTAAGCATCCTGAAAGTCAGCTCTTGTGCCCAGTTTTGGCCCAAGCTAAATGAATTCACTTTCTGCGTTGAAGCCAAGAAAGCTGTGGGGGAGGCTGGCTGTAAGGGTGACCTAGGGGCACCTCTGGTGTGCCATCTGCAACAAAAGGACACATGGGTGCAGGTGGGAATCTTGAGTCACTTTGATGAACATTGCACAAAGCCCTACGTCTTCAGCCAAGTGAGCCCTTTCCTTTTCTGGCTCCAGGGAGTT

>MEU Wallaby

GAGTTCCCCTGGGTGGTGTCACTGCAGTTCTCTCTGTCCCACTTCTGTTCGGGCTCCATCCTGAATGAGTGGTGGGTCCTAACCACCGCCAGCTGTGCCAACATCATACGAAATTCAGAAAGCCTGGCCCGCGTGCAGGCGGGCGTCAACAACCTTGAAGACCAGGTTCGGGCCCAACTTGTGGGTATCCACCGGGCCCTGCCATACTTTGGGATAGAAGGGCCCATGGGCTTGGGTCTCATCCTCCTCCAGGAGCCCTTGCACTTCCAGCCCAGGGTCCTGGCTGTGTGCCTAGAGGAGTCCCCGGAAAAGCCATTAGCAGAGTCTCAACTCCATCTCTATGACTGCTGGATCCCTGGCTGGACCTTGATCAAAGGGAACCTGGTCACAATGCAGAAGCAGCGACTGGATGTGGTTGAGGTTAGCAACTGTGCCCATTATTGGCCCATCAAGAACTTGAAGGCTTTCTGTGTGCAAGCCAAGAAGGTGATAGGCCAGAGCAGCTGCAAGGGAGACCTGGGAGCCCCCCTGATGTGCCGCCCGAAGCTGTGCCCAGAGGAGACCCCCTGGGTACAGATGGGTGCCCTCACTGCTTTTGATGAGACCTGCACTCGGCCCTATGTCTTCAGTCAAATCCACCCCTTCAGCTCTTGGCTCAGGGCCTC

>SHA Tasmanian devil

GAGTTCCCCTGGGTGGTCTCCTTGCAGTTCTCCCTGTCCCACTTCTGTTCGGGCTCCATCCTGAATGAATGGTGGGTCCTGACCACCGCCAGCTGCGCCAACTTCATACGGAATTCAGAAACCCTGGCCCAGGTCCAGGCCGGCGTCACCGACTTGGAAGACCAAGTTCGGGCCCAGCTCGTGGGCATCCACCGGGTCTTGCCGTACTTTGGGCTCGAAGGGCCCACGGGCTTGGGCCTGATCCTGCTCAAGGAGCCCTTGCGCTTCCAGCCCCGAGCGCTGGCCGTGTGCCTCGAGGAGCCCTCCAAGAGGCCGCCAATACAGCCTCGGCGGAACCTCTACGACTGCTGGGTCCCCGGCTGGACCCTGATCAAGGGCAACCTGGTGACTATGCAGAAGCGGCCACTGGACATGGTGGAGGTCAGCAACTGTGCCCGCTTCTGGCCCATTGAGAGCTCCATGACCTTCTGTGTGGAGGCCAAGAAGGTGACGGGCCAGAGCAGCTGCAAGGGAGACCTGGGAGCCCCGCTGATGTGCCGCTCAAAGCCACACCCAGAGGACACCCCTTGGATCCAGATGGGGGTCCTCACCGCTTTTGACGAGACCTGCACTCGGCCCTATGTCTTCAGCCGCATCCACCCCTTCAGCCTTTGGCTCAGGGCCTC

>OAN Platypus

GAGTTCCCCTGGATGGCCTCCATCCAGCTCACCCTTTATCACTTCTGTGCGGGCTCTATCCTGAACGAGTGGTGGATTCTGACCACGGCCAAATGTGCCAGTTTAATAAAGAACTCCGAGGCTCTCGCTGTTGCTCAGGTGGGTGTCGTCAATCTCCAGGATCACGTCCAAGCCCAGGTGGTAAGCATTCACCATGCAATCCATCACCACAGCCCCCAGGGGCCCGTCGGCCTAGGCCTCATCCTCTTGCAGCAGCCGTTGCACTTCCAGCCCCTGGTCCTCCCCATCTGCCTGGAGGACAGCGAGAAGCAGGAGGAACATTTGAAAATCGCGGATTGCTACCTGCCCGGCTGGAGTCTCTTGAGGGGGGGGCCTGTAGCACTCCAGAAACGTCAACTCAGCATGCTCCGTCTCAGCGCCTGCTCTCGGTTCTGGCCCAAGCTCAATGAATTCACCTTCTGTATAGAGGCGAAGAAAGTTGGCATGGCAAGATGCCAGGGAGATCTGGGAGCACCCCTAATCTGCAAAGAGAAGCAGAAAGAAGTGTGGGTGCAGGTGGGGGTGCTCAGCAACTTCGACGAGCACTGCGTGAAGCCGTATGTCTTCATTCGGATAGCCCCTTACTTGTCGTGGCTAGAAAGTGTC

>TAC Echidna

GAGTTCCCCTGGATGGCGTCCATCCAGCTCACCCTTTATCACTTCTGCGCGGGCTCGATCCTGAACGAATGGTGGATTCTGACCACGGCCAAATGTGCCAGTTTAATAAAGAACTCGGAGGCCCTGGCCGTAGCTCAGGTGGGCGTCGTCAACCTTCAGGATCACGTCCAAGCCCAGGTGGTGAGCATTCACCACGCGATCCATCACCACAGCCCGCAGGGGCCCGTCGGTCTAGGCCTCATCCTCTTGCAGCAACCGTTGCGCCTCCAGCCCCTGGTCCTACCCATCTGCCTGGAGGACAGCGAGAAGCAGGAGAAACATTTGAAAATCTCCGACTGCTACCTGCCCGGCTGGAGTCTCATGAGGGGGGGGCCTGTAGCACTCCAGAAACGTCAACTCAGCATGCTCCGTCTCAGCGTCTGCTCGCGGTTCTGGCCCAAGCTCAATGAATTCACCTTCTGTATCGCGGCAGAGAAGGCAGTAGGCAAGGCAAGATGCCAGGGAGACCTGGGAGCACCCCTGCTCTGCAAAGAGAAGCAGAAAGAAGTGTGGGTACAGGTGGGGGTGCTCAGCAACTTTGACGAGCACTGCGTGAAGCCGTATGTCTTCATTCGGATAGCCCCTTACTTGTCGTGGCTAGAAAGTGTC

>ACA Anole lizard

GAATTTCCCTGGATTGTTTCTCTCAGACTATCCATCCAACACTTTTGTGCTGGCTCCATTCTCAACCGTTGGTGGATTTTAACCACAGCCAACTGTGCCAATCTTATAAAGAATTCGGAGGCTTTGGCGCTGGTAGGGGTTGGATTGATAGATGTCACTAAGCCAACGCACTCAGTTCAGATCCGCCAGGCTCTAACACATCCCAAGTCGTTGGAACAGGGAGATCTTCACAACCTGGGACTTTTGGAACTGGAGAAACCACTGGATTTTGGGCCTCTTGTTTCACCTATTTGTATCTCAGGAAAGGCAGACATGATGGGTGATTTCAGAGACTGTTGGCTGCCAGGCTGGACCGTGCTAGAGGGAGGCCCTACTGTACTATTGAAATATCACATAGACATCTTGAACATCAGCAGCTGCAACCAACTGGAAGACAAACTCTCCAGTGCCATCTTCTGTGTCAAAGTCCAGATGGGCGAAGAAGGGATTTGCAAGGGTGATGTGGGGTCTCCACTGATCTGCCCTGACCCCAAGGGTGGGGCATGGCTTCAGCTGGGGGTGTTAAGCAGCTTTGATGAAGCCTGTTCCCGTCCCTATGTCTTCAGCAGCCTGTCTCACTACTTGCCCTGGTTGGAGAAGATC

>PVI Bearded dragon

GAGTTCCCCTGGATTGTTTCCCTAAGACTGTCCATCCAACACTTCTGTGCTGGCTCCATTCTCAACCCTTGGTGGATTTTAACCACAGCCAACTGTGCCAACTTGATAAAGAATTCAGAGACTTTGGCGCTGGTGCAGGCTGGGCAGGTAGATGCCTCCAAGGCAAGCTACTCCGTCCAGATCCGCCAAGCTCTGACTCACCCTGGTTCACTGGAGCAGCAGGATCTTCACAACTTGGGGCTTTTGGAACTGGAGGAACCACTGGAATTTGGGCCCCTTGTTGGACCCATCTGTCTCTTAGACAAGGCGGATACAATGGCTAATTTCAGAGACTGTTGGCTGCCAGGCTGGACTATGCTGGATGGAGGTCCTACTGTGCTGTTGAAGCATCATTTAGACATCCTGAACATCAGCAAGTGCAGCCAGCTTGGGGACCAGCTCCCCAATGCTACCTTTTGCATCAATGCCCAGGTGGGCCAGGAAGGAGTCTGCAAGGGTGATGTGGGCTCTCCACTGATCTGTTCTGACCCCAAGGGTGGGGCATGGCTTCAGCTGGGGGTATTAAGCAGCTTTGATGAAGCTTGCTCCCATCCCTATGTGTTCAGCAGCCTGCCCTTCTACTTGCCCTGGCTGAAGAGGGCC

>RBI Caecilian

GAGTTTCCCTGGATGGTCTCCCTGAAGTTGTCCATTTATCACTTCTGCTCGGGCTCCATCCTCAACCGATGGTGGATCCTCACTACTGCGTCCTGCACCAACATCATAAAAAACGAGGAGTCCTCTGTTCTGGTCCAGGCCGGCATGCTGAACTTCCAGCTAGACTTTCGTTCCTTTCACGTAGAGCTGGTGGTGTCCCATCAAGAATACACAGAGGACCAGGAGACACACAACCTTGGTCTCATTCTGCTGCGCGAACCTTTATTCATCAGCCCTTTGATTTCACCCATTTGCATCTCCAAGAATATAAAATTGGAGCAGCTAATGACGCCCACCAACTGCTGGATCTCTGAATGGACATCACTGCAAGGTGGCCCCAGCATCCTGCTAAAGCGCAGGGTTTCACATCTTCAGCACACGCTTTGCAGTGACTTCTGGCCCATCATCTCTGATTTCACCTTTTGCATGAAGCTGAACCCGACCAACATGACAAACTGCAAGGGAGATATTGGGGCCCCTCTGGTGTGCAAAGACTTCAACAGTTCATCCTGGCTGCAGGTCGGATTACTCAGCGACTATGACAAGACCTGTGTGAAGCCCTACGTCTTCACCAAGGTCTCCCACTACCTGTCCTGGATTGAGCAGAGC

>CPY Fire-bellied newt

GAATTCCCCTGGGTGGTATCCCTGAAGCTGTCTGTCCATCACTTCTGTGCTGGCTCCATCCTGAGCCGATGGTGGATCCTGACCACGGCCAACTGTGCCAACATCATAAAGAACGAGGAGTCGTCGGTCATGGTGCACGCGGGGATCCTGAACCTGCAGTCGGAGACCATGTCGGCGCGTGTGCAGATGGTGCTGACCCATCAGGACTACCAAGAGAACCACGAGTCACACAACCTGGGCCTCGTGCTGCTGCAGGAGCCACTGCACATGCGCTCACTGTCCTCTGCCATCTGCGTGGCTGAGAACATGTCCCAGGAGAAGCAGCTCAACCTTACCAACTGCTGGCTCCCCGGCTGGACTGTCCTGCAAGGTGGCCCCACAGTCATGGTGAAGCACCACATGGACCCGCTGCTCCGGACTGCCTGCAACAGTTTCTACAGTGGCTTCTCGGAGTTCATCTTCTGCATGACGATTGACGAGCCTCACGACACGGCATGCAAGGCAGACATTGGGTCACCGCTGGTGTGTGAAGACCCCTTCAGCAAGTCCTGGCTGCAGATTGGCATCCTGAGCGACTTTGATGTGAACTGCCGGAAGCCGTTCGTGTTCATGAAACTCTCACACTACATGAGCTGGGTGGAGAGAACC

>PWA Ribbed newt

GAATTCCCCTGGGTGGTGTCCCTGAAGCTGTCCGTCCACCACTTCTGCGCAGGATCCATCCTGAGCCGATGGTGGATCCTGACTACGGCTAACTGTGCCAACATCATAAAGAACGAGGAGTCATCGGTCATGGTGCACGCTGGGATCCTGAACCTGCAGTCCGAGACCATGTCGGCGCGGGTGCAGATGGTGCTGACCCACCAGGACTACCAGGAGAACCACGAGTCACACAACCTGGGCCTCGTGCTGCTGCAGGAGCCACTGCACATGCGGCCACTGTCCTCTGCCATCTGCGTGGCTGAGAACATGTCCCAGGAGAAGCAGCTCAACCTGACCAACTGCTGGCTGCCTGGCTGGACCGTCCTGCAAGGTGGCCCCACAGTCATGGTGAAGCACCGCATGGACCCCCTACTCCGGACCGCCTGCAACAGCTTCTACAGCGGCTTCTCGGAGTTCATCTTCTGCATGACGATCGATGAGCCTCACGACACCGCATGCAAGGCAGACATCGGGTCACCGCTGGTGTGTGAAGACCCCTACAGCAAGTCCTGGCTACAGATTGGCGTCCTGAGTGACTTTGACGTGAATTGCCGGAAGCCTTTTGTGTTCATGAAGCTCTCACACTACATGACCTGGGTGGAGAGAACC

**Upstream ORF**

>MMU Mouse

TGGATTCATTTTGATACAATAATACCATACTCATTACAGACTGAAATAGATTCTACATTTCAATCTCTGACTCAAATCTATGCAATTAGGCAATCCTCACGACCAGAATATGAAACTACAGAATTGTTTACACATTCTAATGTTGGTATAACCTATCCTTGGATTCAGCATGCGACTTCTACTGAAACTCTAAAAATCATTCCCTGGATTCATTCTGGTACAGTAATATCATACTCATTACAGACTCAAATAGATTCCTTATTTCAGTCCCTATATGAAATCTATTCAATTAGGCAACTGTCACATCCAGAATTTGATATTACAAAATATTTGGCACATCCCAATGTTGGTATAATCAAACCTTGGGCTCTGCATAAGTCATCTACTGAAACTCTAAGAATCATTCCCTGGGCCCCTTCTGATACAGTAATATCATACCCATTACAGACTGAAATAGATTCTACATTTCAGTTCATGACTCAAATCTATGCCATTAGACAATGGTCACAACAGGAATATGAAACTAAAGAATCATCGACACAGCCTAATGTTGGTATAACTTACCCTTGGATTAAGCATAAAGGTTCTACTGAAACTCTAGATATCATTTCATGGACTCATTCTAGTACATTATTATACCCATTAGAGACTCAAATAGATTTCACACTTCAGGATCTACCTCAGAATTATGCACTTAAACAATCATCACAGTCAGAATATGAAGCTACAAGATCTTTGACACAAACTAATGGTTATAAAATCAACCCTTGGATTCAACACAAAGCATCCACTGAAACTCAAAAAATAATTTTCTGGACTCACTCTGATGCAGTAATATCATATTCAATGCAGACTCTAATAGATTCATTTAGATTTTGGAACCAGTTTCAACCAAAACCAACCCAAATTTGGAATCCAACGGCAGAACAATTTGGAGAAACATCAGTTTTGACTAAAGTTGGTACAGTAACACCATCAGTTCAGTTT

>BTA Bull

GAGACTGCCCCTTTCACAGTCCCAACATGGACTCTGGCTAAAAGACCAGCTGTAAAATCCTGGAGAGAGAATGTGCCTTTCACAACTCCACCATGGACATTGGCTGAAGGTCCACCTGTAAATACCTGGAGAGAAACTCTGCCTTTCATAGGCCCACCTTGGACTCATGCTGAAAGTACAGCTATAAATAGTTGGAGAGGGACTGTGCCATTTGCAACTGCACTATGGGCTCTAGATAAAGGTCCAGCTGTAAATACCTGGAGAGAGATGATGACTTTCACAGCCCCACCATGGACACAGGCTGAAGGTCCAGCTGTAAATACATGGAGAGAGACTATTCCTTTTACAGTCCCACCATGGACTCAGGATGAAAGTCCAGGTGTAATTACCTGGGGAGCAACTGTCCCTTTCAGAGTCCCATCAAGGACACAGATTGAAAGTCCAGATATAAACACCTGGAGAGAGACTGCACCTTTCACAGCCCCAGCATGGGCACAGGCTGAACCTCCAGCTGTAAATTCCTGGAGAGAGATTATGCCTTTTTCAGTCCCACCATGGACACAGGATGAAAATCCAGGTGTAATTACCTGGGGAGAGACTGTCACTTTCAGAGCCCCACCAAGGACACAGATTGAAAGTCCAGATGTAAACACCTGGAGAGAGACTGTGCCTTTCACAGCACTACCATGGACACAGGCTGAAGGTCCAGATGTAAATACCCAAAGAGATACTGTGCCTTTCACAGGTCTACCTTGGACTCAGGCTGAAAGTACAGCTGTGCATACTGGGAGAGATATTGTACCTTTCACAGCTCCACCATGGACTCAAGATAAAGGCCCAGCTTTAAATACCCGGACAGAGACTGTACCTTTAACAGGCTCACCTTGGACTCAGGCTGAAAATCCAACTGTAAATACCTGGAGAGATAAGATGCCTTTAACAGCCCCACCCTGGTCACAGGTTGAACGTCCAGCTGTAAATACATGGAGAGAGACTGTGCCTTTCACAGCACCACCATGGACTCAGGCTGAACGTCCTGCTATAAATAGCTGGAGAGAGACTATGCCTTTCTCAGCTCTACCATGGACTCAGGCTGAAAGTCCAGCTGTAAACACCTGGAGAGACACTATGCCTTTCACAGACCCATCATGGACTCAAGATAAAAGTCCAGCTGTAAATAGCTGGAGACAGATTTTGACTTTCCCAGCCCAACCTTGGCCACAGGCTGAAAGTATAGCTGCAAATGACTGGACACGGAACGCCCCTTTTACAGCTCCACTGTGGTCACACACTGAAAGTCCAGCTGTAAATACCTGGACAGAGGCTATGCTTTTCACAGGCCCTCCATGGACTCAGGCTGAAAATCCAGCTACACATACCTGGAAAGTGAACGTGCATTACAGAGGCCCACCATGGACTCAGTCTGACTCTGCACAAGCAAACCCTTGGACATCAACTGAAAGTTTTAGAATCAGATCATGGACTCATGGAGTAAAGCAAGTTTTGAATATTTGGACAGAGCCAATAGCTTCCACAGTTACACTTCGGACTCAGGCTGAATATTCAACACTAAAATATTGGGCAGAGACTAAAGTCATTTACATAGTCACACCATTGACCCAGTGTCAGTTTCCAATAAATACTTTGACAGAATCTGTAGGATCCATAATCACACCTTGGACATCTGCTGAATCTCTAGTATTAAGTTCTTTCACACAGAATATTATTGATATAATCAAATTTTGGCCAGTGCTTAAAACTGAGTCTAAGAAAAGGTGGAATCTGCCTCAAACTGATACACTCATATTTTCACTAAATCCTCAAACTGATACTTTTGGATCCTTGAACCAAATTGAAAATCAAGAATCTCCTCTGTGGACACATCCTGAAATTGATAATGTCAATACAATGAACTTTCTTGAATCTGGAACACTCATATCACAGGTAGTATCTCTGCCCCAAGCAGCTAGACTCTGGCCCCAAACTGAAGCTGATATTAGCAAAACTTGGTTTGTATCCTCTGAAAGAATAAATTCTTGGGACCAATCAGAGTCTCAAAGAATGAGTACCTCAACCCATTTTGGAGTGGGTAGAGTAAAGCCCTTGGCCCAACATGAAACTGCTATAGTCATGTCATGGCTTCAGATTGAAACTGGTATATTCTACCCTTGGAACCAGTCTGAGGGAGACACAGTGAGGTTCTGGCCCCTTTCTGAAACTGAGGATGTAAGAGAATGGATCCAAACTGGAGCCAGTAGAGTTAACTCTTGGACTCAACCGAGAACTAGTATAGTCAGAGCTTGGCCCCAAGCTGAATCTGAACTAGTCAGACCCTGGACACAAACTAAAACTAATGCAATCACACTATTGACCCAGGCTGATACTATCAAACCTTGGTTCCAAACTCAAATTAATGCAATAAGAGAAGGAGCCCAAACTCAATCTCAAATTGTTACTAGTATCCAAACACAGTTGCAAATAGTTAACCCCTGGATTCAGCCTAAAAGTGATTCAATCAGATTTTGGACCCAGCCTTGGATCCAAGCTGAAACCCACACAGTCAGACTCTTTTATGAAATTGATATAAGAAAATCATGGGCCTCATTTGGATCTCAGTCAGTCACATTTTGGTCACTGAGTCAAAATTCAGTTAGGACCTCATTTCACTTTGAATCTCAGATGACATGTTCCTGGATCGGAAATGAATTTGATATAATCAGTCTTTGGAATCAATATGAAACTAGTTCAGTTGGATCCTGCATCAAGTCTGAAACTGGTACATGTCAACCCTGGGTCCATATTGAATCTTCTACAATCACACCATGGACCCAATATGAAACTTTAGAGATCTACCCTTCAACCCAGCCTGAGACTGATACAGCATTAAGGCATTGGTTCCAGCCCCAAATTGATCCAATTAATACTTGGAATCAGCCCGAAGTAGATACAATCAGATTCTGGACCCAAGTTGAAACAGAAACAATTTCAGTTTGGACCCAGATTGGAAGTCAAGTAGTTAAACTTTCCAACTTTTCTGAAGTTGGCATAGTTACACCTTGGCTAGAGACTGAAACTGATACAAGTAGACCCTGGATTCAGTCTGACTTTCAGTCAGTCCATCCTTGGACCCAGACTGGATTTGGTATAATTAACCCCTGGTATCAGCCAAGAGCTGCTGTAAATCAACCCTGGACATTTGTTCAAACACAGTCAATCGGACCCTGGACTAAGGTGGAAGCCAATACAATCAAATCTTGGTTTCATGTTCAAATGAAAAAAGTCAGACTGGGGATTCCTGCTGAGTCTCAAATATTGAGTTTCTGGATGCAGTCTGATGTTAGTAGAGTTAATGCTTGGATCCAACCAGAAACCCAGGCAGTCAATCCTGGGGCTCATCCTAAATCTGGTAATGTTGCATCCCTGGCTATTCCTAAGCCTGAAAGAGTCAGAATGTGGATCCAGCCTGAAACAGAAATAAGGCCTGGCATCATTTATAAAACTGATATAACCACATCATTTGCTTCTCCTGAAATTGAACCAGATGGAACAATTAGTCATTTTGATTTCTTGTCTAATCGTGTAACATTTTTAACAATAGAAACTGTTCCTTCCCTAGATGAGCATTTTGCAGCTTTGTCAACTGAAATAGCTGCAGTAGAAAGCCAAGGTCAAATAAATTCTGTCCAACCCAGTGAGATCACAAATACTCTCTTTCTTACACTTTCAAGCACATGGCTTCCTGGAGGAGCTGGTTACCTGAACTTTGCCAATAAATTGCAAATTACCAAAACAAAAGGAAGCCCTAATGTCCCATCTAGTTCTCTCAACCCACTTTTTCCATCTTTTTCCTTTCCTGTTCCTTGTTTTATCCCATTTTCATGTTCTTTGTCCCTTACTTGTTCAGTCTTTTCTTCTTGCACACTTTCTTCACCATGTACTTTTCCTTCTTGCTCAGTTCTTCCTCTTGTGGCTTTCTCTCCTGTTCTTCCCTTAGCTGCTTCTGATAGTTCTCTCCAGAAACCATCTTCCTCAGAAGTTACTGAAGACACCATTCTTTCCCATACTTTTTCATCCTTGCATGCTGCTCCAGCCACTCTTT

>MMUR Mouse lemur

aagATTTCAGTTAAGCTATTGGATCTCTCCTTCTGCCATCATCAAAATACTCGCATGACTAGAAGTAATAATTTATGTATCTGGACTCATCCACAAGAAGATTGCTGGGTTCAGCAAGGTAGTCCTGTTTTCTGTGGCTTTGGCAGTCACTGGGAACTGGTAGGCCTGGTCAGTGGGAACTCAGTGGCCTGTTATGACCCTATTCTTGTCATCAAGACAGCCCCATACATATCGTGGATGAGATGGCTTATCAAGGCATTCCAGAAGCCACTGGATCCTATTTTTTCCGTAGCCTGCAATTTTCCTCCTGGAGTAGAACATGGTCCACAAAATAGGCTTAGTCCTAGCACAGGCTTTGTCCCTTTAGCCTCTCACCGATTCTCTATACAATCATGGAGAGGAAGATTAGGCACTTCCCCACTGAACAGACAACGACGGGATCCTCCTTCAGTAAATTTTGATTCAAATAACAGAGTATTTTTTCCTGGCAATAGACAGTTATACTTTCAAAGTAGTCAGCTCTCCGTGGCTAGCAAATTCCCAATGATACAATCCTGGACCTCTCCTGTTTACAAACCATGGGATCCTTTTGCTATAGCTGAGTCCTGGAGTACCCCAGTAGCTGATACTTTTCAATCTTTGGTTCTTTCTGGACTCAAGAACTCCCAAACAGCTGCTGTATCTATACCTTGGGACTTTCCTCAGAGAAATGCGATGCAATATCAATATCAATCTTTGACTGACTCAGTAAGATCCTGGATTAAACCATTAGCTGATATAAGTGGGTTTCATACTGTACCATTATTTAATGCTGGTGATGGATCATGGGTTTTGTTTTCAAGTGACATAGATGAATCCCAATTTTCTTCTGAGATTAATGCTGTTCAGTCTCAAATTGAATCTAGTGAAGTTCCTCCTAATGTTCAACTTATAGGTAGACCATGGCTAGAAATTATTCCTGATGTTGGGACTTGGAAACAATTTGTGCCTTATAAAACAGGAACAGTGATACAGATTCAATCTAGTGAAGAGGGCATTAGAACTCAACTATACCATGTAGAGGATAGAGTGAATACTGTTGTTGATCCAGTAACCTCTAACCTACATCCATTGGTCCCTTTAATGGTTAATAAAATTGAATTATGGGCTCATTCTACACTCAGTGAAGAAGGATCCCACTATCCTACAGTGACCTATAATTTGGATCCATTGTTTCAGCCAGTCTTGAATATGTTTGGAACAGAAGAACCTATGGAAAAGACAAATGGATACTGGATTCTCTCTGAATCTAAATCAACTCAATTTTGGACTTCCTTCACACTTAATATGCCTTTCTCCCGGGCTCTGTCTTCTGATGATATGACTAGATCTTGGACTCAATACAAAGCTAATATGATCAGACCCTCAAATCAATTGAATAGAATTAATCCACTGAATAAGCATCAGGCTATTGTACTCAAACCCCAGATGATGGCTAATGATAAAACATGGCCTTTGATCTATCCTATTACAAATGTAATTGAGCCCTTGATTCACTCTAAATTTGATGCTATCAGACCCTGGACTCATCCAGAAGCAGATATATTCCAAACCTGGATCCAGCCAGAATCCCAATCAGAAAAACTATTGACTCAGCCAGAAGTTATTACAAGTAGATCATGGTCACAGACTACAACTGAAAGAATAAGACCCTGGATTCAACCTAAATTTAGACTATTCAGAACTCAGACTGAGGAAGGTAAAGGTAAACAATGGACCCAGCCAGAAGCAGTTACTGTTAGATCCTGGGCCCACACTGATATTAAAACAATCATACTCTGGAACCAGCCTGAATCTGATTCAGTCACAGATTTGTTCTTGGCTAAATTGGATCAAATGAAAACAATAATCCAACCAGACTTTAACACTGTCCATGCCTGGACCCAGCCTATAATCAGACTATGGACTTTGTCTGAAGTTGGTAGCATCCAACCATGGACAAAGCCTGAAACATTTACATTCAGGACTTGGATACAGTCTGAAGTTAATATGGTCACACCCTGGACAAAACTTGAAGCTGATGCAGTTAGACTTTGGTTCCAGACACAAGCAAATGCAGTAAGAACTTGGAGCCAACCTGAATCTCACGCCATCCTTTCCTGGTCTGAGAGTGAAGCTGACAGAGTCAGATCTTGGTTCTATACACAAACTGATTCATTCAAACCTTGGATTGAGACAGAAATTCAAACAGTTCACCCCTGGACCCAGTCTGAAGGTGATGTAGCCCAACTTTGGACTCAGACTGAGGCTGATACTGTAAAACCTTGGTTCCAGATTGAAGCAAAAACAGTCACACAATGGACAGAGAAAGTAACTCAGACAGCTCACCACTGGATACAGTATGAAAGTGAAATGGTCAGGCCCTGGAACCAACCTATGGCTGATAAATTAAGAGCTTGGATACAACATGAAGCTCATAAAATTAGACCTTGGGATAAGCTTGAAGGTGATAAAATTAAATTCTGGACCCAGTCTGAAGCTGACACATTGAGATCCTCCATTCAAGCAGACATTGATATAATTAACCCTTGGGTACAAAATGAAGATACACTAATACCGTGGATTCAGGAAGAGTCTAAGAAATTAAATCCCTGGACACAGTCTGAAACTGATCCAGTCACACTGTGGACCCATGTAGAAATTCCAGAAGTAAATCCCTGGGCACAATCTGAAACTGACACAGTCACACTGTGGACCCAGACTGAAACTCCAACAATATATCATTGGGCAGAGATGCTAGCTGATATAGTTACAACATGGACAAAGGATGAATTTCAAGGATTAAAGCCCTGGAGAGAATCTGAAACTGAAATAATTACATCATGGATTCATACTGAATCTTCAGGAGTGACTCTGTGGACACAGACTGTATTGGATACAATCATATTGTGGACTCAAGATGAATCTCCAGCTTTAATTTCCTTGACAGAGTCTGTACCTGATCCAGTCACACCGGCAACCCAAGATGTACTTCCAGAAATGAATCCCCAGGCTGGACTGCTAACAATAAATCCCTGGACAAAAATTGTGCATAATATAGTCACACCATGGTTGCAACCTAAATCTCCAGCAGTAAATACATGGATCCTGTCTGAATTGGATCCATTTTCATTGTGGACACGGGCTGATTTGTCCTGGACACAAACTGGCACTGAAACAATTACACTGTGGACCCAGTCACAGTCTCCAGCAATAAATCCATGGAATCAGCTTGAAACTGATATAATCACACCAATGACACACACTGAATCACCAATAGTAAATTCATGGACATATTCTGTATCCAATACAGTCACAATGTATACCCACACTGACTCATTAGATCCATGGACACATGATATAGTCACAATATCAATTCAGACTGAAGTAAGTTCATGGACAGAGTCTGAAATGGATATTTATGCACCTTGGACCCAGGCTAAAACTCTAGCAGTAAATGCTTGGACTCAACCTGTGGCTGATACGGTCACACCATGGACTCTGGATGAACCTCTAGCAGTGAATCCCTGGACAGAGACTGAGACTGAGATTGTCACACAATGGGATCTGGATGATTCTCTAGCAGTCAATCCATGGACACAGACAATAACTGATATAGACAAACTGTGGACCAAGGTTGAATTTCCAACACTAAACTCTTGGATACAGTCTGAAGCTCATAGTGTGACACCATGGATTCAGACAGAGTCTCCAGCAACAAATCTATGGACACGGTCTGTTTCTGATATAATCAAATCATGGACCCCAGATGAATCTCCAGCTGTACATCCCTGGGCACAGGCTATAACTTCCATAATCACAACTTTGACCAAGGCTGTATTTCCAGCAGTAAATCTATGGACAAAGTCTGTAGCCGATAACATCACAGTATGGATCCAGGCTGAACTCACATCTTTATATCCATGGTTACTGCCTTTGCCTGACACAGTCACACCAGGGCCCCGGGCTGATTCTTCAGCAATAAATCTATGGTCAAAGCCTATGTCTGACATAGTCACTTTGTCATCTCAGGCTGAAATTCCACTAATAAATTTTTTGACATATCCTTTGCCTGATACAGTCATACGATGGCCCCACGAAGAATCTTCAGGAACAATTCACTGGACTGAGCTTGTACCTGATATGTTTACACTATGGACTCAGTCTGAATTTCTAACAGTAAAGTCCTGGATTCAGTCTTTATCTAATACACACATATCATGGACCCAGGATGACTCTCCAGTGGTAAAGTACTGGATAGAGGCAATATCTGATACAGTCACACTATGGAACCAGATATCACCTCCAGTAAATCTGTGGACACAATATGAAACTCATGTGTTCACACAGTGGCCCAGATCAGAATCCTCAAAAGTAAATCCCTGGACAAAGCTTGAAACAGATACTGTTACACTATGGAACCAGGCTAATTCTGAAATCCCCAAACCCTGGACACGTGCTTTGGTTGATACAGTGACACTGTTGACCAAGGGTGTTTATCTAGCAGTAATTCCATGGAGACAGACTGAAACTGCAACAGTCACAGCATGGACCCAGGGTCTATTGCCAGCAATAAATCCCTTACCAGTGGCTATAAGTGGTACAGTTGCACTGTGGAATATGGCTGAAACTCTGTCAGTAAAGCCATGGACACACTCTGCAACAGAAATAGTCAGAGAGTGGACTCAAAATGAATCTCCCGCAGTAAATCCATTGATACAACCTATATCTGATATAGTCATTCTTTTCACCCAGATTGTTAGTTCAGCAGGAAATCATTGGACAAGGTCTGAAGCTGATGCAGTCACACCATGGACCCAGGTAAAATCTCTAGGAATAAATGATTGGAGACAGGCAGTATCTGAAACAGTCACAACATGGGCCCAGGATGAATTTTCAGAAGTAAAACTTTGGGCACATTCAGAGGCTGGTACAGTCATACTGTGGCACCACACTCTATCTCCAGTAGTAATTCCCAGGATGGAGGCTCTAGCGGATACATTCATGGCATGGACACAGACAGAATCTCCAACAATAAACCCCTGGATACATCCTTTACCTGATAAAGTCACATTGTGGACTCAGACTGAATATTTCTCAGTAAATTCTTGGAACCAGGCTGAATATCGAAAATTAAATCCCAAGTTACAGTCTGAATTTCCAACAGTAAACTTCTGGACAGAGTCTGAGTCTTCAACCCCAAATCCCTGTATACAGACTGAGAATGGTATAGTAGTCAAACCGTGGAACCATGCTGAATCCCTAGCAGTAAATTCTTGGACATATCCTATAGCTGATACAGTCACACCATGTATACAGGCTGTATCTCCATCATTAAATTCCTTTTTAGAAACTAAAAATCATACAATAAAAGCATGGACTAATATGGAATCTTTACTAGTAAACCCCTGGACAAATGCTAAAGCTTCCATAGCTATAACCTGTACCCAAGATGAATATCCAGCAGTGAAACCCTGGACACAGCCTGTATCTGATACACTTATATCATGGACCCATGATGAATTTTCATCAGTAAATCCATGGACACAGACTGTAGCTTCCACATTGTTACCATTGACTCAGACTTTGGCTTCAGGTGAAAATACCTGGAAACAACCTGAATCATTTACCCCATGGACACAGTTTGGATATCCAGCTGTGAATCTATGGACAGATGCTATAGCTTATATGGTCACACTTTGGACCCCAGCTGACTATCTAGTAGTCAACACCTGGAGAGAAGCTGAAGCTTCTTCCACAGTCACATCTTGGAAACCAACTGACTCTCCTGTAAATTTACTCTCACCTGTAAATTCCTATATACAGAGTGAAACTTACATGAACACATTTGGGACCATGCTTAAAACTGAATTTAAGAAACCTTGGGCACAGCCTGAAGCTAATATATTTAGAGTTTCATTACAGCCTCAAACTGATACTCCCCACTCTACAATTCAAATTGAAAATCAAATGTCTCTTCTGTGGACACATCCTGAAATTGAAAACATCAATACATGGACCTTGCCTGAATTTGGGACACTGATGTCCTGGATAGTCCCTGTTCGACAAGCAGCCATATCATGGCCCAACCCTAAAGCTATAATTACCAGAACTTGGTTTAAAACTCAAACAAAAAGAATAAGACCTTGGATTCAGCCAGAATTTCAAACACTCAGTCCATTTGGAGATGATAGAGTTGAGTCTTGGAGCCAACATAAAACTGCTATAGTCATATCATGGGTCCAGTCTGAAACTGGTATATTCCACCCCTGGACCCAATCTGAGGTAGACACAGTGAGACCTTGGACCGTTTCTGAAGTTGATAAAGTAAAACTATGGACCCAAAGTGAAATAGGCATAGTCAACACCTGGACCCAACCTAAAACTAATACTATCAGACCTTTGGCCCAGGCTGAATCTCAAGCAGTTAGGTCCTGGTCTCAGTCTTTACCTAATATAGTCACAGGACTGACCCAGGATGAAATGCAAGCAAGAAAACACCTGACTATACCTGCCATTAGTATTTTCAGTCCTTGGTTCCATATTCAAAGTGATACAGTAAGACAAGGGACTAAACTTGAACCTCAAACAGTCACTACATGGATCCAGCCAGAATGGCAAAGAATAAACCCCTGGAACCAGCCTAAAACTAATGTAGTCAGACCCTGGGCCCAGTCTGAAAGCGATTTTGCCCATCCCTGGATCCATGATGAAACCAATAAAGGCAGATTATGGACCCATTCTGAAATGGATAAAATAAAATCATGGGCTCAGCCTGAATCTCAAGTAATTATGACCTGGCCTAAAGCTGATACAATCACATTTTGGTCCCCAATTCAACATGATGCACTTTGGCACTGGACCAAACTTGATTCTCAAATGATACATTCCTGGACCCAGTCTGAAATTAGTATAACCAACCCTTGGCCTCTACATGAAATTGCTACAATCAGACCATGGAGTCAGTCTGAGATTGATCTCTGGATCCAGCCTGAAGCTTACAAAGTGATAGGATACTGGTTCCAGACTCAAATAAATTCAGTAAGACCCTGGAACCAAGCTGAAATGGAAACAATCCAAACTTGGACCCAGACTGTAAGACAAATAGTAAAACCCCCAAACTTGACTGAAGTTGATATAGTCACACCTGTGGTACAGACACAAAGTGATATATCTAGATCCTGGATTCAATCTGACACCAAGTCAGTCAGTCACTGGACCCAGACTGGAGCTGGTATAGTTCAGCCCTGGGGTCAGCAAAGAATTGCTACAAATCAACCCTTGACCTATCCAGAAACCCAACCAGTCAGACCCTGGATCAAGCTGGAAACTGACAAAGCTAAATCTTGGTTCCACATTCAGATGAATGAAGGCATACCATGGACCTCTTCAGAATCTCAAATATCTAGTTCTTGGATCCAGCCTGACATTGGTTTAGTTCATCCCTGGATTCAGCCTAAAACCCAAGCAGTCAGACTTTCTACTGAGTTTGAAACTTTCGTAGCCAGATCCTGGTCCCAAGTTGTATCTCAGATATTCAAACCATTGATTTTGTTTGAAGGAAGAACACTCACATCCTGGACTCTGCCTGTAACCCAAGCAGTCAGACCCTGGACCCAGCTTGAAGCTGCAATTATTGCATCATTTGCTATTTCTAAACCTGACCACATTGTAACCTGGATCCAACATGGAACAGAAATAGTAAGGCCTGGAACCCATTATAAAGCTCATATAATTGCATCATTTACTCCTACTGAAGTTGAGTCACCTGCAGAAACCCTCTTAAGCACCCATTTTGGCTTCTCATCTAAATATGTACCTTTTTTACCAGTAGAAACTATTCCTTCTCCAGATCAGTATTTTATAGCTTTGTCACTTGAGATAGCTGCTACCGAAAGCCACGATAAAATCAATTCTCTTCAACCCACCCAGCTTACAAACACTTTGAGTCTGGGAAGAGTTGGTTACAAGAACTATGACAGCAAATCACAAATTATCAAGATGAAAGAAAGCCCTGATACTCTGTCTACCTCTCTTGTCTCTCCTTCCTCATTCTTTTCCTTTACTCCTTGTTTTCTCCCATATCCATGTACATTGTACCCTTCTTGTTCAGTCCTTTCTTCTTGCACATTCCCTTCATCTTGCATTTTTCCATCATGTTCCATTTTCTCTCCTGTGGACTTCTCTCCTCTTCTCCTGCCCTTAGCCTCTTCTGACAGTCCCCTCCAGGAACTCTCTTCCTCAAAATTTACTGAAGAGACCATTCTTTCTCAGACTCTTTCTTCCATGCATGCTACA

>RNO Rat

ATGGCCCTGAACCTATCATGGACGAGGCAGTTTATTACTATAGATGAAAAACCACTGGATTGGATTTTCCCTTTGCTTTGCAATTTTTGCCCTGGAATAGAAGACAGTCTTAATAAGAACATAAGCAGTGCTGCTTTTGTCCCCCACGGATTCTCTTCTCACTCATGGAGTAGATTAATTACTTCCTCACAGAGCAGATCACGGTGGAATCCTCCTCCATTTCTCTTTGTTTCAAATAACAAAGACTCTTTTGCAGGCAGGCAGCACTTATATTTTCAAGTTGGACGTATCTTTTCAGCTAGCAAATCCTCAACATCACGACCCTGGACAATTCCTATGTTCAAAACTTTGGGTATTTATCATTCAACTGCTTCATGCAATAACCCGTTATCTAGTCATTCTGAGTGTTTTAGTCATTGTGAGACCTCGATCCCACCCAAAGCTTGTATGTCATCTAGCTTTCCCTGGAGAACTGGACTCCAGTACCAAAGTGGAAAACTTGCGGGATCAGGTAGATACCGGACTGATCTTTCAGGTGCTATAACTGGATTTCACACAATATCAATTAATTTCATTGGATCTTTGATTCCACTTCCAAAGCAAATAGATGGATTCCAAATCAGTTCTGAGATACATTCTCTTTATCCTCAAATTAAACCTCGTATATTTTCTTCTCCTGATCAGTGTGTCATTGGATCGTGGCTGGGAGTTTCTCCTGATATTAGATCTGGGGCACAGGCACAGGCTGCGCCATATGAAACTGAAACAGTGATATGGAGCCCATTTAATAAAGAATTTGATCAAACGAAAATTTATTATATTGTGGAAAAAGTTACTATTGGTGCAGAGGAAGAAAGTTATGATGTAAATCTATGGTCCCCTTTATTAGTTAGTGAAATCGAGTTAAAGTGTCCCATGCTCAGTGACTGTGGGTATCAGTACTCTTCAGCAACACCTACGGTGAATACATTGTTCAGTGGAGCTTGGTACTCAGCGAAATCCCAAATCACCTCTACAGAACCAGATGGAAAAACGATGTCTCCTGTATCTAAGACAAATCTGTTATCCTATACAATTACTATTCCTTTCTCTAAGCCTCTACCTACAAACAATCCTTTTCTGTCTTGGGCTTATTATAAACCAAATAAGATCATAGAATCAAGCCACCTGTGTACAATTAGCCTACTGAATACACATGAGGCCAGTGTAATAAAACAAAAATGGTTTGAAGTTATAACATGGCCTTTGTTCTATTCTAATATTGTTAAACCTCTCACTTATTCTAAAATGAATTTCAAGCGACAATGGATACAAAGCGATTCAGACATACCACAGCTTTGGACGTTTTCAAAAGTCAAGCCAAGGAAGTATTTGGATACCTTTAAAGCTGATAAAGCAAGGCTGTGGTTGCAGATTAAGAATAAAGAAACAGAATTCCAGAAACATCCCAAATTTCCGTTTGAGAGTCCCAGAATTGAGAGCAGAGAAGGTAAAATAAAATATTGGATGAAGGCAAGAGGAGGTGCAATTATATCATTGGACCAAACAGAAACTTGTACAATTGTACCACAGAACCGACCTGATCCTAGTGCAGTCGGATGTTGCTCTAATTCTTATCACACATTGCTGAGGATGCAACGAGATGTTAAGACTTTTACTTATCTTGAAATTGATATGGCCAGACCATTGGGCAGCCCCACTGGAAATACTGAACATTCAGAAAAGTCTATACCATTTACCGTTAGGTCTCGGATGCACGCCGAGGGTGTGAAAATCACACACTGGACAAAGTCACACACTGAAGAAAAGGTTTGGTTCCCTGTGCAATCAAGAGGGGCCCAGCATCCATGTGACTCTCAAGTGAATCATTACTTGTCTGAGCTGCAAGATGAAAAAATACGACCTTGGTATCGACCACAAGTCAATACAGTCAAAACTGTGATTAACACAGAGTTTCCAATGCTGCCTTCCTGGATCAAACAGGAAGGTGACCAATCAGGACTCTGGACTCAAAGCCAAGACGATTTTGCTAAATATTGGTTTCAAATTCGAATTAAAATGATCTCACAAGGGGCACAGCCAGTATCTCAAACAGAACACAAATGGGCAATGTTGGAAACTGAAACAGATAGACTCTGGGACCCACCTGTGGTTGACAAATTAAGAAACTGGATCCATCATGGCGTTGATACAGTGAAGACCTTAAATGATTTAGATGTTCATAAAATTAAACCACAGGTACACAAAGAGGCCAGGACATTAATAAAATGGAGGCAGACTGACTTGCTCAAAATAAAAACCCAGGCACCATATGAAGCTGACATAGCCATGTCATGGAATCATGATGGAAAACTATTAGTCCACAACTGGAAGGACTTTTTGGAAGACAGAGACACAACATGGATAAATTCTCAGCTCCAAGAACTGAATGACTGGGCACCACCTAAAATCGATAGATTCTCAAGGTGGACAAAGACAGACACACCACTCTCAAGCCTCTTGATCTCGTCTGAAATGGACACATTTGTGCAGTCAGCCGTAAGTAAATCCTCAAACATAAATCTCCTGATAGATTCTATAATTTATGCTATGCTATTGTGGACCAAAAAGAAACCAGCATCGAAGTCAAAATGTACTCATACTATACTGATTTTGTGGATGTCAACAAAAATGTTACCAGGAAGTCTCTGCACGCAAACCCTACGGGATAAGTACGTGCCATGGCTCCAGGATGAGTCTCAAGAAACAAATAACTGGATGATTTCTGAATCTGATTTAACGTCCTTGTGGCCTAAAACTCACTATCCAGAAATAAACTCAGTACCGTTTAAGGGTGAAACATTTACAATATGGGACCCAGAGGAATACCCTACACCAAATCTATGGAGAGAGATTGAAGCAGAGATAACCACTGAACTGACCCACACTGAGTCACCACTAGGAACTTCATGTGCATTTCAAGTTCCCAACATGACAGTTTTTAACCAGGCTGACCCTCCGCCAAGAATTCGTCCGCTGAGGGAGGACTATGATGAAATAACAACGTGGCTCTCATACAAAACACAACCAGTAAATCCGTGGACTCCACCAGGTAGGAATTTAGCCTCGGCTCCTCACTATGAAACGTCAGTGAACAATCTCTTCTCAGAGACAAAATATGATACAGATATTCCATGGTTTCACACTGAGTTTTTTACAGAAACCTCACTGACGGGGGCTGAAACTGTTTCAGGAGTGGGCGTGAATGACGCTGAAACTGTAAATATGTGGACACAGACTACGGATGCTTTGGAAAAATTACGGACGAAGGATAAAATCCAAATACCCACTTCCTTCACAGAGGGGAGAATGGATAGTTTCACACCATGGATTCAGATGGAGTCTCCAACATATGATAAATGGACACTCCGTGTATCCGATAAAATCACTTTATTGACTCAAGCTATGCATCCAACAGGATATCAATGGACAGAAAGCATAGCTCCCACTGCCTCAGTTTGGCTGAAAGGCAATCCTCTGTTAGTAAATCTGTGGCTACAGAAAATATCTGACAGGGCTCTATTGCCATGGCCCCAGGCCGAATACCTACCATTAAACCTGCAACCTCCAGCTGTGACTTACACAATCATACCACACCTATTTCAAGCTGAATATGCACCAATCAGTCTGTGGAGAAAACCCACGTCCGATGTCATCATCATGCCATGGAATCAGCCTGAATTATTCAATCAGAAAACATATCTTGAATCTAATACCAGAGTCCTGCCGTGGCCGGAGCCCGAACAGATACCTGAGCACTTATGGCTCCTACCAATAAGTGATACCTTTATATCACAGTGGCCATGGCACGAATCTTCTAAAATAAATATGGCATCAGCAGTAAACCCTGATGCATTGATAATGTCTATACCACGATGGCCAGAGAAGCACACACCTGAGGTGAATCTAAGGTCACTACTTGTATCTGACAGAAAGGCAGCAGCTTCGTTATGGAGTGAATATTCAGCAGGAAATGCGCTTGTATCACCTATGGATGACAGAGTCACATCTTGGTCCCAGTTTCAGGCCCTGCCAGCCTACACACAGGCGTGGACTGTATTTGATATGATGATGCAATCACATACCCACAATAAGTGTACATTAATAAACCAATGGCCACACATTTTCTCTGGTGCAATGACATCACCATGGTGCTTGGATGATTTTCAAGCAACAAGTATGTGGAGACAACCTGTGTCTGATGAATTTGTGCTATCAAGGACACCGGTTTCATCTCCAGAAGTAAATCCGTGGGGCTCTGTGATGTCTGATGCAATCACAGTCTCGTGCTCTGGATCCGAATATTTGCTAGTAGACAGGGGGACACTCCTCAGATATAATACAATTGCACCTGCATGGCTACAGCAGAGACTCCCACTGGATTTAAGAATGTCCCCCATATCTGATATAATCACGCCACATTGGTTACAGAAAGAATCTACAGATGGAAATCTAAGGCCGTGGAGTGTGTCTGATGCAGTGACACCGACTTGGACCCAGATAGAATCTCCAATATGGGGTCTGTGGGCGCTGCCACCTCCTAATCCAGGGCCTCCAAATGCATTTCCAACAAGAAATCTATTGCCAAACTACTCAGCTGACAAAATCACACAACCGTGGGATCGCACTGAAATTCCATGGATACATCATGCATCCATCACTATTCCGCCATTACGTCAGACTGAGTTTCGAGCTGAAAACCTTTGGACATCGCCTTTGTCTGACACAGTGGCACCATTATCAACCCATGTGGGTTCTTTGCCACTATACCTGAGGACACTGTTACCGTCTAACTTTATCACTGCACTGTGGACCCTCCCAGAACCTTTGCCATCGAAACTATGGACATCATCTGTATTAAATGGACTCAGACTGTCCTTGCCCCAGACTGAATCTAAAAACATAAATGCACAGGCACTGTTTGAGGCTGACCCATTAATCCTGCCATGGACTCAGTTCAAGCACAAAACACTCACTCCATGGACAGCGAAAGCAGATGATATGTTATCACAATGGATGGCGTCTGAAGTTCTTTCTGTACAATCTCAGATTCGATCTGCATATGATCCAGATCAGAGCCATCTCCAAATTCCAGCCATCAAACATTGGATGCATGCAGTGCCTGACCACGCTTTCTTAAGAAACCAAGCTTCTTCACCTGCAATGTATTTCTGGAAAAAACACGGCATTGATACATTTATGGAGTGGGCCACTTCACAATTCACAAACACAAGGCGCCCGACAGAGACTCTATCTACCATACTCAACCATGATGAAATTTCAACAACAGATTCTTGGACAAATTTTGAAACAAATACTGTCTCACAAATGTCCCAAGCTAATTCCAGGATCCTCAATCTCTGGACACACAGTTTTGCCCACTGGATGCAGTCTAATTTTGATGGAGTTACTCCATGGGCTCAAACACTATGGGCTGTAGAAAGTCCAACTATTCATATGCGTAGTACAGAACCATTGTGGAAATTGGCTCATATCAAGGTAATAAGATCTTGGAAACAATCCACGACAGAAATGGCCTTACAAGTGATAACAAAAAAGTCCACTACAGTATATTCCTTCACAAAATTTATATTAGATAATGCCTTTATTTTTGATCATACAGTCAGTCCAATAGGAAGTCGACAGACACAGACTGAAGATGATGAAGCGATATGGACACAATCCAAGTCCCTTAAAAAATATATTCAGACAAATCCTTACTCTAAAATACTCACAAAATGGGGACAGGGTGAATTTTCAGAAGACATTATTTGGGCAGAACCTGAAGCTGGCAAAGCTATGGTTTGGCAACAGAGCAAATCTAAATCTTTTATTCCAAGGATAGGAGAGACAGTTAATGTAGTAAAAGCAAGGGCAGAAACTGAATCCTCAGTCAAAACTTATTTTGCAAAATTAGTACCCAATTCATTGAGTGCACAGATACAAGCAAACTTCCTAGCAATGAATGCTGATGCACAACCTATACATGATAAATTTGTATTGGGGACAAATGCTAACTATCCATTAACAAAAAGTTGGAGAGAGGAAGGAAATAGAGCAGTCAACACCAAGGGAATCTCTGAAAATCAACCTGCAACAGAAATTGTCAACTTGTGCACAGACATGGTGTATCCAACATTCAATTCTTTGTTACAAAGAGAGAATGTAGCAACTGTATGGTCACGAGTTGAACCTCAACAAATAAAATTTCCATGGCAGAAGACAAAATTGCCAAACATAATACACTGGACACAGAGTGACTCATTATCAATATATCCATGCATGAATAATAAATTATCTAAACTCATGCCTTGGACCCTTGTTCAATATATACCAGAATATCACTTAGCAGATTCAAAAGCAATACCTTGGAGTGAGACTGAGCCTTTTCTAGCACATATATGGAAAGAGACCACATTTTCAAAAGTCATATATTGGACTCAAACTGAATCCCTGTCAGTAACCCAGTCAACACAGGATTCAGTAGTCACACCATGGATCCAGAGGGGATTTCCGCAAGAAGATATGTGGGTAGGTTATACTGCTCCTAAAATCAATCAATTGATTCAGGAGGACTATGAGTTAGAAAGTTCTCCAACAGTGACTCTATCCTCAATAGTTTTTACTTGGACACACCCTGAACATATACCATTAGTTCCATGGACAGAGTCCATGGAGCTTAGATACACAACAATAAACAAAGGGGTGTGGCCTTCATATAGTCCTATTATATTGGCCCATCCTGAATCCCCAGAAGTATTTATGCCAACAAACTCTGTTTCTGAAACTTTCACACCACTCAAATGGAACGACTTCCCAATAAAATATCACTGGGCACAGCCTTTACCTGATAAACTTATACCACTGACCAGGGTTTTATACATACCAGAAAACCTTTGGACAGAAGTCGTATATTCTACATTTGTACACATGACAAAGTTTATATCTTCATCAGAAATTATTCCTATACAGCCTACTTATTATGCAGTCACACCATGGGCTAAAGATAAGGTTTCAGAAATAATTACCTGCACATTGTCTGATTATCATGCATGCATATCATGGAACCAAAGTATGTATGTAAATTCCATAATAACAGAGGCTTCTAGTTCTGTACTTAAAAAGGCCCTCCAAACTCAGTCTTCTTTAGATTTTTTGGCACCAATAATAACCACCAAATTTACAATGTGGAGACCAAGTGAACTGCCATTATTGGAAGCTGAGTTGAAGCCCATAGCTTCCATAATTGTTGAATGGACCCAAGCTGAGGTTTCACAAAACTTAATAGCAGAAACTTCAAAAACAACCGACACATCTCTGAACTCCATTATATTTCCACCTATACTGTTTTATATGAAGGCAGAAACTGAGAAAGGAACATTTTTAACAGTGACTGAAAGTGAAGCTAAGAAAATTTTGACAGTGTCTAATATTAATACTTTAATAATTTCAGTGCATGCTCCAACTTATGTTATCCAACATTCAACACAAATTAGGTGGACATCTCTTTTGTGGGCACCTACAGAAACCCTAGACAGAAATACATGGTCTTTTTCTGAAGTGGGATCACCCTTATCCTGGAAGGTGCCTATTCCTTTAGCAGCTAGTTATGAGCTTCACCACAACCCAAAACATTGGTTTAAAACTCAAGATGAAAGAATAAAACCATGGACTCAGTCAGACTTTCAGATAATCGATGTTTCCACTCTACCGATGACTAACAAAGTAGAGTCCCATGATCGACATATAGTGGGTATGCATTCTGCTACTGATATATTTGATGCCTGGTCCCAGTCTAAAACAGAAAGTGTAAGATTCTGGAATATTTCAAAAGACAGTACATCAAGGCCATGGTTCCAAATCGAAGCTGGCATAGACAATAATCAGATGCCGCTGAAATCTACCGCAGTGAAAGTATGGCCTCAGGCTGAATCAGAAGTGTTTCGACCCTGGACCCAGAATGTATTTAATATCCCACTGTCACAGGATGAAATAGAACCAGAAACATACTTGAACATGTCCAACATTAATGCTTGGTTCCAGTTTCAAAAAATTTCAACAAGACAAAGAACTAAATTTGCCCATGAAACTGTTACTCCATGGATTTGGCCAGAATGGCAAATCACTCATTCCTGGAATGAACATGAGATAAAGGTACCAAAAACTGGGTCACATTTTAGAACAGATACATCCCAAATATTCTTAACCTATTCAAAGTCTGATGAGATTAAACTTTGGACTCAACCTGAATCGCAGCTTGTTAGGAGGTGGACTGAAGATACCATAGTCACACTTTGGTCCCTGACTAAAAATGATGCAATTAAGCCATGGTTAGGACTGGAGTATGAAACAACACAAGCTGTGGCACAGCTAAGTGATGGTGTAATCGATCCTTCCGTTCAGTGTGAGGCTTCTACTGAAACGCTACCAGTGATTTCCTGGATTCCTTTTGATGCAGTACCATACTCAGTACAGACTCAGATGGAGTCTACATTTCAATCCCCGACTCAAATCCACGCAATTAGGCAATGGTCACAACCAGAATATGAAACTACAAAATATTTGACACATTCTAATGTTGGTGTGGCCTACCCTTGGGTTCAGCATGAGGGCTCTACTAAGACTCTAGAGATCATTCCCTGGACTCGTTCTGATACACTAATATACCCATTACAGACTCAAATCGACTATGAATTTCAGTCCCCGACTCAAGCCTATTCAATTAGGCAATGGTCATACCCGAAATATGAAATTACAAAATATTTGGCACATCCTAATGTTCCTATAATCAAACCTTGGATTCAGCATGAGGACTCTACCAAAACTCAAGGAATCATTCCCTGGACTCATTCTGATATGGTAATATCATACCCATTACAGACTCAAATGGACTCTACATTTCAGTCCCTGACTCAAATTTATGCAGCTAAGCAATGGTCACAACCGAAATATGAAACTACAGAATCTTGGAGACAGCCAGGTGTTGGTATGGCCTACCCTTGGATTACGCATCAAGGTTCTACTGAAACTCTAGAAATCTTTTCATGGGCTCCTTCCAATACAGTGATATCCTCACTACAGACTCAGATAGATTCCACACTTCATTATCTACCTCAAAATTATGCCATCAGACAATGGTCACAACCAGATTACAAAACTACAAGATCAACACAAACTAATGGTCACGAAATCAGCCCTTTGATTCAGCCTCAAGGTTCCACTGCAACCCTAAAAATAGTTTTCTGGACTCACTCCGATACACTATCATACTCAATACAGACTCTAATGGATTCATTTAGGTTTTGGAACCAGTTTCAACCAAAAACACCCCAAATTTGGGCCCCAACTGCAGAACAAGCTAGAGAAACACCAGTTTTGACTGGAATTGGTACAGTAACATCACCCTTTCACTTT

>TCH Tree shrew

GATAGAGTTAATACTGGTGTTAAACCAGAAAACTATAACCTACATCCATGGGACTCTTTAAGTGTTAGTAAAATTGAAATAGAAACTCANNNTAACCACGATGGATCCCAAAACTTTATAGAAACTCATACTTTAAAACCATGGTTTTATCCAGTATTTAATATTTTTGATTCTCAAGAACCTATAGAAAATACAGATGAACACTGGATTCTCCCAGAATCTAAGTCAACTCAATTTTGGACTTCCTCTACATTTCGTATGTCTTTACTTTGGTCTCAATCTGTAAGTAACACTATTAGATCTTGGACCCAAGACAAAGTCAATAAAATCAAACCCTCACAACAAATAAATAAAATTACTTTCTTGAGTAAACATGAGGCTATTATACTAAAACCACAGGTCCAGACTGAAGCTACATGGCTGTTGATCTATTCTATTACTAATGTAAATAAGCCATATATCCACTCTAAAGTTGATATCATGAGGACCTGGATTCAATCTGAAACAGTCATAATCCAAACCTGGACCCATCCAGAAAGCCAAACAGGAAAACCCTTGTCTCAACTCAAAGCTGAGAAAGTCATTCCATGGTTACAAGCTGTATCTAAAAGAATTATACCCTGGATCCATCCTAAATTTCAAATAATCAGACCCAGAATCCAAACTGAAGAAGGTAAAGACAAAATTTTGACCTATCCAAAAGTAGGAATGATTAGATTTCGGACTCAGTCTGAAGTTGGTAGCATCCAACCATGGATAAAACTTGAAACATTTACCTTTAGATCCTCAATAGAATCTAAAGTTTATAAAGACATACACTGGACAAAACCTGAAGCTGACACCATCAGATTTTGGTTCCATACACTAACTAATATAATAATAACCTGGAGCCAACCTGAATCTCAGATGATCCTTTTCTTGTCTGAGACTAAACCTGCTAAACTTAGATCTTGGTTCCATAGACAAATAACTACATTCAAACCTTGGATAGAGACAGAATTTCAAATAACCCACCTAAAGACCCTATTTAAAGGTGATATTACCAGATCAATAATTCAGAGTGAGACTGACACTGTCAAATTTACTCAAATGGAGACAGTTACACAATGGACAGAGCAGTTAACTCAAACAGTTTACTATTGGATACACTCTATAACTGAAACAGACAGACCCTGGAACCTGTATGTGGCTGATGAATTAAGTGCTTGGAGTAAACATAACGCTTATACAATTAGGCCCTGGAATAAACATGAAAGTGATAAAGTGATATTCTGGACTCAGCTTGAAACTAGTACATTAAGATCCTGGATTCAGGCAGATGTTCGTGTAATCAACCCTTGGGTCCAACATGAAGCTAGCACATTAACAGAATGGACTCAGCATCATCTAGATTTAAATCCCTGGATACAATATGAAACTGCTCCAGTTACACTGTGGATGCAGGTGGAAACTGCAGAAGAAAATCCCTGGATACAATCCAGAACTGGCACAGTTACACTATGGACTCATGCAGAAACTACAATAGTAAATAATTGGACAGAAATGTTAACTAATACAGTCACAACACGGACAAGGACTAAATTTCAAGAACTAAATCCCTGGATACAGTCTATTAGCTCTTGGAACCAAGATAAAGTCAATATGAAAGAAATTGAGATGGCCACACTGTGGGCTCAGGCTGATTCTCTAGCAGTGAATCTACAGACACAATTTGTATCTGATACAGTCATACTGATACAGGCAGAATCTGCAGTACTAAATCCCTTCACAGTGTCTAAAATTGATACAGTCATGCTCTGGACCCAGGCTGAACTGCCAACAACAAAATCCTTGAAAAAATTTGTCAGTAATACTGTAAGATCATGGTTGCATTCTCAGTCTCCAGCAATAAACACCTGTGTTCTAACTATATTTGATCCATTCTTATGGTGGACCCAGTCTGATTCTCCAGCAGTATACCCCTATTTTCAGTCTGAAATGGATATAGCCACACTATGGACCCAGTTTAAATCTCTAGAAGCAATTCCTTATGAACAGTTTAAAACTGATACAGTTATACCAGGGACACTCATTAAAATGCCAGCAGTAAATTTATGGACACATCCTATGTCTAATACTGTCACAGTCTGTGCCCAGGCTAAATCTTTATTGAATCCTTGGACACAGTCTGGAACTGATATAATCATAACATGGCCCCAGGCTGAAATTACAGATGTGAGTTCCCACAGGTGCTCACCTGTAGCTGATACAATCATACAATGGACTCAGAATGAACATCCAGCAGTATATCCCAGGACTGAAGCAATGGCTAATGCATTTACATCACAGGCCCAGACTGATGTTTTATCAGGAAATCCTCAGGCACAATCTGATACAGTCACAGAGTGGGCCATGGCTGATTCTCCAGAAATAAATCTATGGACTCAATATGTATCTAATGCAGTCACTTTGTCATTCCTGACTGGATCATTAGTAGTACATCAGACAAAGCCTATGTCTGATAAAATCACACTGTGGCCCCAGATTGGATATCCAATAGTAAATCAACTGACACAGTATGTATCCAACACAATCATCTGGTGGCCACAGGCTAGTTTGTTAGCAGGAAATCTACAGACACACACTGTATCTGATGCGCTTTGGCCCAAAACTGGATCTCCTATAGCAAATCTATGGACACAGCATGTATTTAACACAGTAACATTGTGGCCTAAAACTGAAACTCCAGAAGTAAATCTGTGGACAAAATCTGTATCTGATACAGTCACATCTTGGCCTCACTCTAGATCTCTAATAGTAAATGTATCGACACAGAATAAATTCAACACAATCACACTATGGTCCCAGGATGAATATTCAGCAGTCAGCCTATGGAAAGAGCATGAATCCAGGACAATCACCTCTTGGTCTCAGTATGGCTCTCCAGAAATTAACCTAGAGACTCAGCTTGTATTCAGCATAATTACATTGTGGCCCAAGGATGAATCTCTAACTATAAATCGATGGACACAGAATATACTCAACACAATAACTCAAGGATCTCAGACTGGATCTCCCTTAGTAAATCTATGGACACAGCATGTATCTAACACAGTCAAGCAATGGCTTCAGGATGATTCTCCAAAAGTAAATCTATGGGCACAGCTTGTATCTGATACAGTTAAACTATGGTCTCATGATGGATCATCAAGAATAAATCTATGGATACAGCTTATGTCTTATGTAGTCACATCAACACCAAGGTCCCAGAGTGAATCTCCTACGGTAATTCCATGGATACAGCCTGTGTCTGATAAAATCACATCACAGTGGCCCCAGGCTGAATCAATAGCAATAATTTCTTGGAGAGAGCCCATCATCAATATATTCACATTGTGGTCTCAATATGAATTTCTAAAAGTAAAGCCTTGGATTAAGTCTGTACCTGATACACTCATACCATGGACTCATATTCTGTCTCCATCAACGAAGCACTGGACACAGGCTATATCCTCCATAGTCACACTATGGAACCAAACTGAGTCACCTCTAGTAAATCTCTGGACAGACTATAAAACATTCAGACAGTGGACCAAGGCTGAATCCTCCAAAACAAATCACTGGATTCAACTACTACCTGATACAGTAATATCATACCAAACTGAATTTCTGGCCATTTCATGGACAAAGCTTGAAACAGATGCTACACAGTGGACCCAGGCTAATTCTGAAATCATCAGTGCCTGGACACAGACTATAGTTGATAAAGTGACACCTTTGGTCCAGGGTATTTTTTTAGTGGTAAATCCCTGGACACAGTTTGAAACCAGTGGAGTTCCAATGTGGATAAAAGCTGTGTCTCCAATAGTAATTCCCTGGACAAAATATGAAAATGGCCCAGTCATATCATGGACCCAATCTCTATTGCCAGCAATAAGTACCTTAACAGGGGATTTGCCTGGTAGAGGTAAACTGTGGAACATGTTTGAAATTCTGTCGTTAAAGTCCTGGACACAGTCTTCAACAAAAATAATTATACAGTGGACTAAAAATGAGTCTCCTGAAGTAAATCTATTAACACAACCTGTAACAGATATAGTCATTCTTTTCACTCATGCTGTTAATTCTGCAGTAAAGCACCGGACACAGCCTGACACTAACATGATCATAACATGGACCCAGGCTAAATCTCTAGAAGTAAATGCATGGACCAAAATTATTTCTAAAACAGTCACACCATGGACTCAGATTGAATTTTCAGAAGTAAAGATTTGGCTACAGCCTGGAACTATTATAGATAGACTGTCACACCAGGATGTATATCCAACAGTAATCCCCAAAACAGAGGCTATAGCTAAGATAATCACACCATGGACAGAAATGGAATCTCCAGTACCAATTTCCTGGACACAGTCTGTAGCTGATACAGTCAAAACATGGACAAAGATAGAATTTCCAAGAGTAAATCCTCAGATGCAGTCTTTACCTAATACAGTTAGACTAACGATTCAGACTAAATCTCCAATAGTAAATACTTGGATTCAGGCCAAATTTGCAAATGTAAGCCCCAGCATACAGACCGAATTTTCAATATCAAATACCCAGATACAGCCTGTAGCTTATACAATCATACCATGTGCACAGACTGCATGTCCATTATTATCTTCTTTCCTGAAAACAGAAAATTATATAAACACTGAATGGACTCAGGAAGAATCTCCATCAGTAATTCCCTGGATGCAGATTGTAGCTACAAAAATCATACCCTGGACCCAGGCTGTGTCCCCACAAGTAAATCCTTGGACACAACCTATAACTTCCATAGTTACACAGGAAATTCAAGCTGAATATCTGGCATTACATCTCCAGCCTGTTGTTCATAAAATAAAATACTGGAGACATGTATCACCATGTCCAGTCACACCACAGTCCCCTTCTGAATCTCTATCAGAAATTACCTGGATAGAGCTAGTTTCTGATATACTCACTTCATTGACCAAGGATGAATCTTTAGTAGTAAAAGCCCAGATATTGGATGTATCTAAAACAGCAACGGCATGGACCCAGGCTCAATCTCCAACAGACATTGATTGGACATGGGCATTCACCCCATGGAGCCACATTTTATCTGTAGAAGTAATCCCTTGGGAAAAGGCCATGGCTTCCACAGTCACACAATGGACCCAGTCTGTATCTCCAGTAGTCATTCCCTGGACACAGGTTGTATCTTATATAATCACTCCATGGAAACAGACTCTATCTCCAGCAATAGTAACCAAGACACAGGCTTTGGCTTCCACTATGCCTTCAAGCTCTATCTCCAGCCATCATTTCTTGGTCATATGGACTCGGGCTCTACCTCCAGCAGTAATTCCTTGGGCACATTCTGTGGCTTCCAAATTCACACCATGGACCAATATTCAAGTTTTAGCAGCAATTCTCTGGACACAAGCTATGTCTGATACTGTAATTCGACAGATCCACTCCCTATCTCTATCTGTAAACCTCTGGACAGAAACCTTATATGATACAGTCACTCCATTGACACATGCTCTATCTCTCATTACAATTTCCTGGACACATGCTGTATCTTACACAGTCACACCATGGACCCAAGCTCTATTTCCAGCAGTTATTCCCTGGACATATGCTGTGACATCTACACTAATGACTCAAACTCCATCTATACCAGTAAATACTAGGACAAAGATTTTGACTTCTAAAATCATACCTTGGACACAGACATTATCTCAAGCAGTAAATCCTAGAAAACAGGCTAAAGCATCCACCATCAAACCATGGACCCAAGCTCTATCTTCAGTTATAAATTCCAAGACACAGAGTATAGCTTCCACATTTACAATATGGATGCAGGCTCCTACTTCTTCAATAATTCCCTGGACATATGCGGTAGTTTCCACAGTTCCATCATTGATCCAGGCTAAATCTACAGAAATCAGTCTCTGGACCCAGAATGTAGCTACTATAGTTACTACATGGGCTCTAACTCCAGCCATGACTCCCTGGGCAAATGCTGTTGTTTCCTTAGTCCCACCATGGACCCAGGCTCCAAATCTTTGGACAGTATCTATATCTGATACAGTTATATCATGGACCCAGGCTGTATCTCCTGGAGTAATTATGTCTTTAGAGCTTATATCTGATATAATTACACTGTGGATGCAATCTGAATCCCCTTCAATAATTCCCTGGACCCAGTCTGTATCTGACACACTCGCTTCATGGATACCTGATTATTCTTCAGCAGTAATGTTCTGGACAGACATGGCAGCCTCCACAATCACTCTGTGGACCCAAGATATCTCTCCAACAGTATATTTGGAGCCATATACAATAGCTTCCACAGTGTTTCCTTGGACTCAATTTCTACCTTCAACAATAAAGACTGAGGGACAATCTGTGATTGAATCAGTCACACAGTGGTCCCAGGCTGAGTTTTCAGGAGTCAACCCCTGGACAGAGGCTATAACTTCCTCAGTCACATTGTGGGCAAATACAGAGTCTCTAACAGTAATTTCTGAGACTGCAGATTCCTCAGTTACACCTTGGAAACAGACTTACTCTCCAGGTGTAAATTCCTATACACAGAGTGAAATTAATATAGAAACATTTTTAACAGAGCTGCTTAAAACTGAAATTAAGAAATTCTGGCCATTGCCTGAATCTTTGATATTAAGGGTTTCACTGCAGTTTCAAAGTGATAGTAACCAACTCTTGATTAAAACTAAAAATCAAGTATATCATCTGGGAACACATTCTGAAATTAAAAATGTTAATACATGGATTTTTCCTGAATCTGGAACCCTCATATCCTATAAAGGACCTATGTCTCAAGTAATTAGACCCTGGCCTCAGTCTGGAGCTCAAATGAACAGAATTTTGTTAACAACTCAGTCAATCATTCCCAGAACCCATACTGAAGTTTGGACCTGGACTCAACGAAGAATCATTACAAATCTACTCCTGGCCCATTCTGACACCCAGACAATGAGATCTTGGATCACACTGAAAACTGATATAGATATGTCTTGGTTTCACATTCAGCTGAATGAAAGCAGACCAATATCTGAATCTCAAATATTGAATCCCTGGATCCAGCCTGAAGTTAACATAGTCCATGCCTTGATCCAGCCTAAAACCCAAGTAGTCACACTGCAGGCTGATTCTGAAAATTTTATCATCAATTTTCTGACTCAGATTGTGTCTCGGGTAGCTGAATCATGGACTTTGCTTGAGGAAAGAGCATTGGAACCTTGGATGATACCTGCAACCCAGACTAAGGATGATAACTCATCATTTATTATTCCTGAACTTGAAAACGTTAGAACCCAGATCCAGCCTAAAACAGAAATAGCAATATCTAGAACCCATTATAAAGCTAATGTAATTGTATCATTTGTTCCTCCTGATGCCAAATCAGATGGGAAAACTCTATTGAGTCATTTTGACTCCTTGTCTAAACCTTTGTCCTTTTTACAAATAGAAACTATTGCTTCCCCAGATCAGTATTTTATAACTTTGTCAACTGATATAACTGCCATAGAAAACCAAGATAAAATAAATTTCCTACAACCTAGCCAGCACACAAACATTCTTTTTACCCTTTTAAGCACTTGGATTCCTGAAAGTGTTAGTTACCAGAATTTTGCCAGCAAATTACAAAGTATCAAAACAAAAGGACGTCATTTTGTCCCCTCTGCATTTCTCAGTTCTCTTGCTCCAGACTTTTCTCTTCTTATTTCTTATTCTGTCCCTTTCCTATGTACAGTGTTCTCTTCCTGTTCAGACTTTTCTTCTTACATATTTCCTTCATCCTGTATTTTCCCAACTTGCTCCTTCTTTTCACCTGTGGACTTCTCATATTTTCTTCTACCCTTACCCTTTTCTGATAGTTCTTTTCAGGGAATATCTTCCTCTAAATTAATTGATGAGGCCATTCTTTCTCATACTTTTACATCCCTGCATGATGCT

>PMA Deer mouse

gaatcaaaagtaacttctagatttacaacatggagattagaTAAATGGTCATTAATGCAAACTGATGTGAAGGCTATACCTTTCACAGCTATGGCATGGACAAAAACTGAGCCTCTGGGAAATGAGATGAGGTCAGAAGTTTTAACAATCAATGAGACATCATTGAACTCAAAGGAATTAACACCTGTACAATTTTATATACATACTGGTATTGATAAAGGTACATTTATAACAGTGACTGAAATTGATGCAAAGAATGTTTTAACAGTGCCTAGTGTTAATATTTTCAGAATTTCAATGCAAGCTCAAACTTATGTTATTCAACATTCAACTCAAACTGGACAAACATCTGTTCTGTTGACCCCTACAGAAACTATAGACAGAAATGCATGGTCGTTTTCTGAACTAGGATCACTCTTATCCTGGAGAATGCCTGTTCCTTTAGAAGATGATCACTGGCATCACCATGAATATTACACCAGCAGATATTGGTTTAAAACTAAAGCTGAAAATATACGACCATGGACACAGCCATATTTTCAAACAATCAATGTCTATACTCCATTAAGGGCTAGCAAAATAGAGTCCTGGGTCCAACATAAAATGGGTATAAAGTCATGGGTGAATTCTGCTACTGGTATATTTAATGACTGGACCCAGTCTGAAACAGAAAGTATAATACTTGGGAATGTTCCTAAAAATAATACAATAAAACCATCATTCCAAATGGAGGCTAGCACTAATCATATCTGGTTCCAACTGATAACTAATACAATAAGAGTATTGCCTCAGGTTGAATTTCAAGTAATTAGTTTCCTGACTCAATCTGTCTCTGATTCACTATTATCACAGTCTGAAAAAGAACCAGAAACATACTTGTACATGTCTGATAATAATGCTGTTTCATCTTGGCTCCCAATTCAAAATAGTTTAAGACAAAGAAATAAACTTGAACCTGAAACTGTTGCCCCATGGGTCCAGCAAGAATGGCATATAGCTCATCCCTGGAATAGACTTGAAACAAAGGTACTAAAAACTGGGTATAATTTTGAAGCAGATACTTCTCAAATACTCTTGACTTATTCTAAATCTGATGAAATTAAACTTTGGACTCAACTTGAATCTCCGCTGGTAAGGAGGTGGCCTGAAGATGCTATTGTCACACTTTGGCCTCTGACTAAAAGTGATGTAATTATGCCATGGTCACAACTGGAATACAAAACTATACAATATTTGGCACAGCATAATTTTGATAGAATCAACCCTTGGATTCAACATAAAGTATCTACTGAAACTCTAGAAATCATTCCCTGGACTCATTCTGATATAGTATTATCACATTCATTACATACTCAAATTGATTACACCCCTTGGCCACTGACTCATACTGATGCAACTAGGCTATGGTCAGAAGTGGAATATGAACCTACACAATCTTTGGCACAGACTAATGCTGGTTTAATCAATGCCTGGATTCATCATAACGATTGTACTGAAACTCTAGATGTCAATTCCTGCATTTATTCTCATAGACTAATATCATATTCATTACAGACTCAAATAGATTCATTTAGATTATGGAACCAGCTTCAATCTATGACAGCCCAAACTTGGAATCCAACTGTAGAACAAGTAATAAAAACAGAAGCTCCGACTGCAGTTGTTACAGTTTCACCATCGTTCCAAGTA

>PER Cactus mouse

ATGCCTGTTCCTTTAGCAGATGATCACTGGCATCACCATGAATATTACACCAGCAGAAATTGGTTTAAAACTAAAGCTGAAAATATAAGACCATGGACACAGCCATATTTTCAAACAATCAATGTCTATACTCCATTAAGGGCTAGCAAAGTAGAGTCCTGGGTCCAACATAAAATGGGTATAAAATCATGGGTGAATTCTGCTACTGGTATATTTAATGATTGGACCCAGTCTGAAACAGAAAGTATAATACTTGGGAATGTTCCTAAAAATAAGGCAATAAGACCATCATTCCAAATGGAGGCTAGCACCAATCATATCTGGTTCCAACTGATAAGTAATACAATAAGAGTATTGCCTCAGGCTGAATCTCAAGTAATTAGTTTCCTGACTCAATCTATCTCTGATATACTATTATCACAGTCTGAAAAAGAACTAGAAACATACTTGTTCATGTCTGATAATAATGCTGTTTCATCTTGGCTCTCAATTCAAAATAGTTTAAGACAAAGAAATAAGCTTGAACCTGAAACTGTTACCCCATGGATCCAGCAAAAATGGCATATAACTCATCCCTGGAATAGACTTGAAACAAAGGTATTAAAAACTGGGTATAATTTTGAAGCAGATACATCTCAAACACTCTTGACTTATTCTAAATCTGATGAAATTAAACTTCGGACTCAACTTGAATCTCCGCTGGTAAGGAGGTGGCCTGAAGATGCTATTGTCACACTTTGGCCCCTGACTAAAAGTGATGTAATTATGCCATGGTCACAACTGGAATACAAAACTACACAATATTTGGCACAGCCTAATTTTGATAGAATCAACCCTTGGATTCAACACAAAGTGTCTACTGAAACTCTAGAAATCAATCCCTGGACTCATTCTGATATAGTATTATCACATTCATTACATACTCAAATTGATTACACCCCTTGGCCACTGACTTATACTGATGCAATTAGGCTATGGTCACAAGTGGAGTATGAACCTACACAGTCTTTGGCACAGACTAATGTTGGTTTAATCAACGCCTGGATTCATCATAAAGATTGTATTGAAACTCTAGATATCAATTCCTGCATTTATTCTCATAGACTAATATCATATTTATTACAGCCTCAAATACATTCATTTACATTATGGAGCCAGCTTCAATCTATGACAGCCCAAACTTGGAATCCAACTGTAGAGCAAGTAGTAAAAACACAAGCTCTGACTGCAGTTGTTACAGTTTCACCATCATTCCAAGTA

>MGL Bank vole

ATTTTAAAAAAAACATGGAAGAATGATGCATTACTAGCACTCACTTCCTTCACAGGGATGGAAACTCATATTTTTACACCATTCATTCAAACTGAGTCTCCAACAACACATCCATGGACACAGCATGGATCTGATAGAGTCACTTTATGGAATCAACCTATATATCAAACATGGAGTCAAGGGACAGGAAGTATGCCTCCCACAGAAACAATGTGGCTGAATAGTAATTCTCCATTTGTAAATTTATGGCTTCAACAAATATCTGATAGACTTATACTGTCATGGCCCCATGTTCAATCCCCATCATTAAACCTGCAGTCACAGTCTGTAACTTATACAGCCGTATCACAGTTGATTCAAGCTGAATATGCACCATTAAGTTTGTGGAGAAAACCTGAAACTGATGTAATCATAGCACCATGGCCTCAACCTGAATTATCAAATCAGGAAACTCAGACTTTGTCTGGTACAAAATTACAGCCATGGTCTGAGCCTGAATTGATACCAAAGTATCAATGGACAATAACAGTAACTCATAGACTCATACCACAATGGTCCCAGGATTTGTCTTCTACAATAAATATGAGGTCACCACTAATATTTGATACACTCCTAATATCTACAACACCATGGCCACTGAAGCAAACACTGGAAACAAACCTAAGAACACTGCCTGTATATGAGAAAAAGACAACAGCTGGTTTCCAGAGTGAATATTCAATATCAAATACATTAATATTGTCTATGACCAATAAAGTCATATTACATTGGTCCCATTCTAAGGTTCTACCAGCTTATCTATGGAGATGGCCTATATCTGATACAATGATGCAATCCTGGACCCAAGATGAATATTCTGTAAAAAATCAATGGTCAGAGTTTTTGTTTAATGCAATTACATCATCATTGTCTTTGGATAATTACCCAGCAACAATGCTGTCTAGACAACTTATATCTGATGCATTTGTCCTGTCAAGAATACAGGTTGTATCTCCCAAAGTAAATCTATTTGGACTCCGGTTACCTGATTCAATCATACTTTCTTGGTCCCAATCTATCTACTCATCAGTAAATAGTGTCGGATACTCTGGACCTGATAAAATTGTACCATTGTGGCCAAAGGTTAAATATTCACAAACAAATAAGAAGACATCATCTGTATCTGATATAATTATGCCACATTTGTTTCATGAAGTAACTTGGGAAGGAAACAGATGGCCATGGAATTACCATGATAGAGTGACACCAACTTGGACACAGATAGGATCCCCAAAATTAAGTCTGTGGTCACTGATAGATCCTAATAGAGAGTCTTCAAATGTATCTCCTTCAAGAAATATATTATCTAGTCATTCAGCCATTATAATTGCAAAATCATGGCCCCATACCAGGTCTCCAATATTTAGTATATGGTTACTTGGGGTATCTGTTACTATTTCACTATTACATCAGACTGAGTCTACAGCTGGAAATATTTGGTCATTTCCATTATCTGATACAATCACAACAATGTTGACTCATTCTGGATCTCTGCCAGTGCACCTATGGACAGTGCTTCCATCTAATAATATTCCTTCCACATGGCCTCATCCTGAACCAGCAAAACTATGGACATTACCTATATTTAATAGAAACAAACCAGCTTTGTTCCAGACTGAATCTAAAAAACTAAATACACAAGCACTGTTTGTGTCCAATACATACATACAGTCATGGGACCTATCTAAATACCAAACACTCATTCCTGGGACAAGGATAATTGCTCATGTATTATCACGATGGACCACGTCTGGAATTCTAATTGTAAATTCTTGGACCGAGCCAGCATCTTACACAACTCAGAACTATGTTCTAACCCCAGAAGTCAAACACTGGACTCACCTAATATCTGATCCTGTTTTATTAAGGAACCAAGCCACATCACCTACAACAAAGTTTTGGAAAAAATATGGAGCTGATGCATTCACTGAGTGGGACATTTCACAATTCACAAATATAAAACATCCAACACAGTCTTTTCCTAATATACTCAAGCATGTAGAAATTTCAACAACAGATCCCTTGACAAAGGTTGAAACAGATATCTCACAGATGTTCCAACCTAATTCTAGTAACAGAAGTATCTGGTCACAAATTTTAGAAAATACAGTAACATCATTGCTTCAGAGTGTTTCTCTAATATCTATTCCTTGGACAAAATTTCAATCTAATACAATCAGAATGAAGAATCAGTCAGAGTCTTCAGTATTAACTTATTGGACACAGTCTATTTCTGATCAAGTTTTACCATGGTCTCAAACCCTGTCAGAAGCAGAAAACCCAACACTTGTTATGAGAGGTACAGAAACACCATGGAAATTTCCCAAAGTTGTGTTACTAAAATACTGGAATCAATCTACAACAAATATTATCTCTCAATGGATAACAGACAAGTCTTCTGAAATTCATGTATTCCCAACAGTTGTATTTGATAAAGTCATTCTTTTTGATCATGCAGTAAATCCATTATTAAGTCAATGGACAGAGACTGAAGCTGATGCAGTCAGAGAAGCCAAGTATTTTAACAAATATGTCCTGACAAATTCTCACCATAAAATAGTCACAAAATGGGGCCCAAGTGAATTTTCAGATAAAATGATCTGGACAGCCCCTGAAACTGGCACAGCTGTGGGATGGCAACAGGGCAAATCTACATCTTTTAGTCCAAGAACGGAATATATAGCTAAAGTAGTAAAAGTGAGAATGCAAACTGAATCCTCAGCCATAAATCATTTTGCAAAATTAGTAGTCAGTACACTGACAACACAGACTCAAGGAGAACTCACAACAAAAAATATTGATATGCAACATGTATTTGATAAAATCACATTTATAGCAGAACCTAAATATCCATTACCACACAGTTGGATCCAGAAAGAAAATGTACCATTCAATACAAAAGAAACGTCAGAAAATCATTGGGTGCAACCTGCAACTGAAACTGTCAGATTGTGCATATACACAGTGTATTCAACACTTAATTACTTGCTACAGACACAAAAATTAGTTACAGGATGGTTTCAAGCTGATCCCCAGAAAAGAAAAAATCACTGGGATAAGGCTAAATTATCAAATATCATACATTTTACGCAAAATGAATATCTATCTACATATCCATGTATAAATGATAAAATATACAAATTCATGCCTGGGACTCAAATTCAATACATACCAGAATATCACTGGTCAGAAGCTACAGATTCAAAAGTAGTGTCTTGGAGTGAGACTGACTCATTCCTAACAAATATATGGAAAGAGGCCATGTTTTCAAAAGTCATATACTGGAACCAATTTCACTCTCTATCAGTTAATCCATCAACAAGAAAAAGGGCTTCAGTACTCATTCAGAGGGAATTTCCAACAGAAGATACCTGGATAGACAGTTCTACTCCAAATGACATTCATTGGATTCAGGAAGAATCTTCATTAGAACAGTCCGAGACAGTGTCTCTAACTTCAATTGCTATTAATTGGAGACATTTTGAGCTTATACCAGTAGTTCCCTGGACAAAATCTATAGATCTTAGATATCCACTAACAAACAAATTGATGTTGTCTTCATATAATCATATCATTTGGACCCATCCCAAATCCTTACATGTAAATATACCAATGAGTCCTCTTTCTAAAACACTGACACCATTGGCATTGGATGAAAATGATTTAAAAAATCACTGGACAGAGTCTGAGTTTGAAAATCTCATAACATTGACCAAGGTTTTATCTGTATCAGAAAAGCTATTGACAGAAGATCTTTTTTATACACTTTCACCCTGGACAAAATTTTTATTTTCATCAGAAATTACTTCCATGCACTCTAATTTTGATGCAATCACACCAAGGGCCTTGGCAAAAATTTCAGCAGTAATTTCAACATGCATATTGTCTGATAATCATATATGTATATCTTGGAACCATAATGAGTTTCTAAAATCTAAACTAAAAATGTCTACTAGTTCAATAGTAAAACAGAACTTCCAAAGTGAATCTCCTTTAGATCTTTTGGAACCAAAAGTAAGTTCTAGATTTACAGCATGGAGACTAGATGAATTGTTACTACAAACTGATGTTACAATTATACTATCCACAACTGTTGCACAGACCCAAACGGAGTCTCTGGGAAGTGAAATATGGCCAGAAATTGTAACAAACAATAAGACATCATTGAACTCAACTGAATTTACACCCATATATTTTTATATGCACAACAATATTGATAAAAGCACAATTTTAACAGTGGTTGAAATTGAGGGGAAAAATGTTTTAACTGTGTCTACTATCAATATTTTTAGAATTTCAATGCAAGCACAACCTTATGCTACCAAACAATCAAGTCAAACTGCACAGACATTGGTTCCATTGGTCTCTACAGAAACTGTAGACAGAAATACATGGTCTTTTTCTGAATTAGGATCTCTCTTATCTTGGAGTGTGCCTGTTCCTTCAGCAGATATTCTCTTGTATCATACTGAATATCACATCCGTGGAAGTTGGTTTAAAACTAGTGTTGAAAGAAAAAGACCTTGGGTACAGCCATATTATCAAACAATCAGTATCTATACTTCATTAATGATTAGCACAGTAGAATCCTGTGTCCAACATAAAATGGGCAGAAAATCATGGATGAATTCTGCAACTGGACTGTTTAATGACAGAACCCAGTGTGAAACAGAAAATGTAAGACTTGGGAATGTTCTTAAAAATAATACAGTAAGGCCATGGTTCCATGAAGAAGCTACCACAGACCATACCTGGTTCCATTCGATAACTAATACAGTAATAGTATTTACCCAGACTGAATATCAAGAAATTGGACTTTGGACTGTGTCTGTCTCTGATATTCTATCATCAAAAGCTGAAATAGAACCAGAGACATCCTTGTACATTTATGACAATAATGCTGTCTCATATTGGTTCCCAATTCCAAATAGTTTAAGTCAAAGAATTAAAATCGAGCCTGGAACTGTTACCCCCTGGATTCAGAAAGAATGGCAAATAGTTCACCGATGGAATAGACTTGAAACAAAAGTGGTGAAAAGTGGGTACAATTTTGAAGCAAGTACATCTCAAATAATTTTGACCTATTCTAAATCTGGTGAAATTATACCTTGGACCCAACCTGAATCTCCACTGGTTAGGAGATGGCCTGAAGATGCTATAGTCACACTTTGGACACTGACTAAAAGTGATGAAAATATGCCATTGTCACAACTGTTACAGCATAATTTTGGTGGAATTAAACCTTGGATTCAGCATATAGCTCTTACTGAAACTCTGGAAACCATTCTCTGGACTCATTCTGATACAGTACTATCAAATGCATTGCAGACTGATGTAGATGACATACTTTGGTCATTGACTCAAGGGGATGCAATCAGGCAGTTGTCACAAGTGGAATATGAAGCTAAACAATCTTTGACAGGGCCTGATATTGATGTAATCATCCCTTGGATTCAGCATAAAGATTCTTCTGAAACTGTAGAAGTTATTCCCTGGACTCATTCTGATCCAGTGATATCAGACTCAATGCAAACTCAAATAGATTTTTGGTCCCAGAACCAAATTGATTCTATCAGGCAGTGGTCACAAGTAGAATATAAAGCTAAACAGTCTTTGGCACAGCCTAATGTTGGTATTTTCAACCCTAGCATTCAATATAAAGCGTCACCCGAATCTCTAGAAATCATTCCCTGGACTCATTCTGATATAATATCACACATATTACAGACTCAAATAGATTCCATACTGTGGACACTGACTCAAACTGATTTTGATAAACTATGGTCACAAGTGGAATATGAAAATGTACAATCTTCAGTACATCAGAATGTTGGCTTAATTCACTCATGGATTCAGCATAAAGATTCTACTGAAACTGTAGATATCTTTTCCTGGACTTATTCTCATAGAGTAATATCATACTCATTACAGACTCAAATAGATTTACTTAGATTATGGAACAAGTCTCAACCGATGACAGCCCGAATTTGGAATCCAACTTCAGAACAAATAGTAAAACCAGATGCCCTGACCGCAGTTTTTACAATTTCACCATCATTTCAGGTT

>CPO Guinea pig

TCACAAGGTTCAACAGTGATATCCTGGCCCAAGGCTGAATCTCCAATAGAGGGTCTTGGGAAAGAAGCTTTAGCTTCAGAAGTAAAGCTGTGGAACCCCATTGAATTGCCAGCAGTCAACTGGATAGATCCTATAACACAGCTTATATATCACACAGGAAACACCTGGACAAAGTCTTCATCTAACCCTACTATATTACAGAATTGTTCTGAATCTTCAGCAATCATTTCCCGGATGTACACTGTGTCTGACACTTTTACAGCATTTACTAAAAATGAATTGACAATTTTATCTGATGAAATCACACTGTGGGCCCAAGCCATGTTTGCTTCAGACAAGCCTTGGAAGAAAGCCCAATATGGCATAGGAATGCCTGTGACTCAGGCTGCATCTATATCTAAAGTTTCCTCTATGCAACCTGTGTCTGATGCAGACATACCACAGAGCCCAACTGAGTTTCTGGCCAAAGTTGGGAGGATACACTCTATATCTGATACCTTGATACAATGGATGCATTTTGGATCTCCAGGAAGCAAGATTTGGACTGAAATTACAGCTTCCACAGTTACACAGTGGAGACAAGCTAAATTTCCTATTTTCAGGTTTTGGGAACCAACTGTTACTCCTACAACCACCATGTGGAAACCAAGTGCATCTCCAGCCATTAACTTTGGTTCAAGGGCTTCAGCTGCCACAGTCTCCCCTTTGACACACTATGCGTCTCCAGAAATCCAGCCTTTGTCAGGACATTTAGCTTCCACTCCTAGGAACTGGAAACAGACTGACTTTTTGCGTATGAATGTTTACACACAAACTGAAACTGATGTAGCCATATTTTCTGAAATGTCTGTAAAACCTTTGATTCCACTTGGTTCTAAAGTTAATGTATTCATTCTGTCTGAACAAACTCAAACTTACACAACCCAAGGCTTGAATGAGATGGAAAATCAAGATTATATTCTGAGGGCACATAAATTTGAAGATGCTAATGCAGGGACTTTGTCTAAAGTTGGAGCACTTGTATCTGAGGCTTTGCCTGCAAGTCAATCAGCTAGAGTCTGGCCTGAAATTGACCCTCATAATAGCAGAGGTTGGTTTGAAATTGAAACAGAAAATATCCAACCATGGGCTCAAACAGATTTTCAAAACTATATCTCATTTAGGACCAGACAAATAGAACTAGGGGTCCAACATACGCTTGCTTCATTCACCTCATTGACCCAATCAGAAGTTGGTATGTTTGATCTGTGGACATCTAAAGTAGACATAACAAGGTCCCAGACCACTGCAGGGATTGAGAATTTACTGAGACCACTGATCCAAACTGAATCTGAGTCAATCAATGCCTGGATCCAGCCAACGGATAATATAATCCGACCACATACCCAAACTGATTATGAAGCAACCAGGCTCTGTACTCAGGACACATCTGATAAAGTCACAATGTCATCCCAGGTTGAAATTCAGGAACCAAAGCGCTTGATCCTACCTGACAGTTATAATGTCAGAGTTTTGTTTCAGACTCAAAAGAATACAATACAAGAAGGCCAACTTGAAGAACAAATGGTTACTCTCTTCATGGAGGCAGAAGGGCAATCTCTTGAAAGTAATATGATTGAAACTCCTTCCCAAGATAAGTGTGATATAACCCAAGTTGAGGACTACTCTGGAACAAATTCAATCAAAGGTTTGACCTCTTCTGAAAGTGATGAAATACTACTTTTGACCCAGACAGAAGGCCAAGTGATGAAAACCTGGGCAGAGGATGATTTGGTCAGAAATTTAGCCTTCACTGAACATACGACAAATAGTCATTGGTCCCTCACTGAATCTCAAGTGACACATTCGTTGGCCGGGACTAAAGTTGTTCTAACCGATCCTTGGCTTCAGTACAAAGCTGGGATGATCAGCCATTTGGCCCAGTCTGAAGCATCAGAAAGTATTCACTTGACCCAGCCTGGAGCCGAAAGAGAATACTGGCTCCAGTCTCAAATAGATTCAGTCGGATCCTGGAACCAGCCTGAAAGTAAACCAATTCTAAGCTGGACCCCAACTGGAAAAGAAATAATAAAAGGGTCGAACTGGGAGGAGGTTGAGGTTGAGGGAGTGACACCTTGGCTTCAGACTCAAGACAGTTCTACAGTTCGGATTCAGCCTGACTCCAAAACAGTGAGTCCTGAAATGCAGAGTAAACTTGGTGTAACCCAGACACAGGAAAGTGATGCTGCAAATCATCCTGATACTCAAGCAATCAAACCCTGGCAGAAACATGAATTTAGTAAGGCCAGCCCTTGGTTCCACATTCAAATGAGTACAGTACTGCAAGGCTCT

>MPU Ferret

ATGAATCCAGTCAAAGGAGTTATAGGAGTGGTCCTTCTGCAGTACCCTATTAGAGGGAAAAAAATACCACTTTATCAGACTCACAACATCTTCTGGAAGAGCTGTAATAACTGCCAGTACAGACACTGCAGGGTGTACCAATATCAGAAGCACAATAACTTTGGAACCAGTATCAAAACGCTGTCAGTTAAACTGCTGGACCTCTCACTTTGCCACCATCAACACATCCACCTGACCAAAAGTAACAATTTATGCATCTGGAGCCAGCCACAAGAAGACTGCTGGGTACAGCAGGGTAGTCCTGTTCTCTGCCTTTTTGGCAAACACTGGAAACTGGTAGGCTTGGTCAGTGAATCCTCAATGGCCTGTTATGACCCTGTTCTTGTCATCAAGACAGCTCCATATTTATCTTGGATGAACTGGCTTATCAAGACATCCCAGAAGACACTGGATCCTATTTTTTCTCTACTCTGCAGTTTTACTCCAGGGTTAGAACATGGTCCACTAGACAGGCTCAGCCTGAACAGGGAAACTGCCATTTTGACCTCCCCTGGATTCTCTCTACAGTCATGGAAGAGAACATTAGGCACTTTCCCACTGAACAGACAGCGCCGGAATCCTCCTCCCATATTTTTTCATTCAAATAATCAAGACTATTTTCCAGGCAGTAGAGAGTTACATCTTCAAACTAATCAGCTCTCCTCACCTAGCCACTTCCCAATGATACAATCTTGGACATCTCTTGTTACTAAACAGTGGGATCCTTCTGATATGTCGGAACCCTGGAATACCCCAACAGTTGATACTTCTGAAATTTTGGTTATTTCTGGAACACCAAAGCCTCCAACACCTGATAAATCTATACCCTGGGGCCTTCTTCAGAAAAATACAATGAACTATCAAAACCAAACTATTACCAATTCAGTAAATTCTTGGGTTAATCCTTTAGCTGGTAAAATTGGGCTTCATACACTGCCATTAGTTAATTCTGCTATATCCAGTGTTTTGTTTTCAAATGGCATAGATGGATCCCAACTTCTTTCTGGGGTTAATACCGTACAGTCTCAAGTTCAATCTAGTAGGTTTCCTTTTCATGGTCAACCTCTAGCTAGACCCTGGCTGGAAATTACCCCTGACATTGCACCTTGGAAACATATTGTACCTGATAAAACAGAAACAGTGATGCAAATCCAATCTACTGAAGAGAATGTTGGAAGCCAAATACACCATGTAGTTGATAGAGTTCACACTGCTTTTAAACCAGTAACTTACAACTTGAATGGATGGGTTCCTTTAACAGCTAATAAAAATGAATTCTGGACCCATTCCACACTAAATGCAGATGGATCTCTGTATCCTACAGTAACTCTTACCTTGGAACCTTGGTTCAAGTCAGTCTTGAATTTAGGTGGATCCCAAGAACTTATAGAAAAGACCAATGAATACTGGAGTCTTCCTGAATCTAAGTCAGCTCAATTATGGACTTCCTCAGCACTTAATATTCCTTTTACCTGGATTCCATCTTCAAGCAATAATATTAAATCTTGGGAAAAATATAAGACCAGTATTATCAAAGCATCAAGTCAAATGGATAGAATAAGTCCATTGATTGAACATGATGAGTCTATTGTTTTCAAACCCCAGATTCAAACTGCAGATACAACATGGTTTTTGATACCTACTATTACAAATGTAATTAAGCCTTTTGTTCAGCCTAAAGCTGTTACAATTAGACCCTTGACTAAATCTGAAACTGACATAGTCCAAACCTGGACCCAGCCAGAAACCCAAACAAGAAAACAACTGACTCAGCTGAAAGCAGATAAAATCAGACCATGGTTACAGACTAAAGCTGAAAAAATCAGATCCTGGATTCAGCCTAAGTTTCAAATATTCAGAGCTAGCGACCAGAATGAAAATGGTAAAGACAAATATTGGACCCCACCAGAAGCAGATATAATTAGATCCATGACCCAAACTGAAATTAAAACAGTCAGACTTGAGAACAAGCCTAAAGCTGGTATGGCCAGATCTTGGTTATGGACTAGGTCTAATCAAATGAGAACAAGTTCCCAGCCAGACTTTCAAACACTTTACCCTTGGACTCAGCCTGAAGTTGACATAATAGGACCATGGACTCAGTCTGAAGCTGGTAGCATCCAACCATGGATGAAACCTGAAGCATCAACATTGAAAATCTGGACACAATCTAAAGTTAATACAATTACACCCTGGACAAAGCCTGAAGATGATGCAGCTAGACTTTGGTTGCAAATACAAACAGATACAGTCAGAACATGGAGCCAACCTGAATCTCAAACAACCATTTCCTGGTCTGAGCTTGAAGCTGATAGAGTCAGATCTTGGTTTCAAACTCTAATGCATACATTAAAACCTTGGATTGAGACAGAATTTCAGACAACTCACTTCTGGACCCAACCTGAAGGTGACATAGCCGGGTTTTGGACTAAATCTGAGGCTAACAATGTCAGACATTGGTTCCAGACTCAAGTGGACACCGCCACAATATGGACAGAGTCAGTATCCCAAGCATCCCAGCCATGGATACAGTCCAAAACAGAAATAGTCCGGCCATGGAACCAGCCTGTGGCAGACAAGTTAAGAGCCTGGATACATCAGGACATTTATACTGTAAGGCCCTGGGATAAGCTTGAAGGTGATAAAATTAGATTCTGGACACAGTCTGAATCTGACACAAATCCTTGGATTCAGCCATATGTAGGTATAATCAATTCTGGGACACAAAATGAAGGGGATACATCGACACCATGGGCATGGGTGCAGGCTGAGACTCCAGAAGTTAATCCTTGGATACGGACTGAAACTGAAACAGTTATAGTCTCAACCCAAGGAGAAGCTCCAGCAATTAATCATAGGACAGAGGTTTTACCTGATACTACATGGGCAAAGACTGAATTTCCAGGACAAAAGCTCTTGACATATCCTTTGTCTCATACAGCTACACAGTTGACTCAGCCTGAGCTGCCAGTGGCCAGTCTCTGGACACAACCTATAGATGATACAGTCACTCAGTGGAACCATAATGAACCTTCAGAAATAAATTCTTCAACAAAGACTATAGTTGATACAGCCATGAGGACACAGGCTGAATCTCCAGCAGTGAATCCCAGGATACAGTCTGAAAATGATATAGTCACATCATGGACCCCAGATGAATCCCTAGTCATAAATCCTTGGACAAACTCTGTAGGTGATACAGTCACACAGTGGGTCCAGAGTGAACCTCAAGAAGTACTTCCTTGGGTAAAAATTGCAGCTGATACACCATGGATGCAGGTGGAGTCCCCAGCACTAAACCCCTGGATGCAGTCTGAAAGTGATATAGTCACACCATGGGTACAGGTTGAATCCTCAGCACTAAACCCCTGGATGCAGTCTGAAAGTGATATAGTCAAGCCATGGACACAGGTTGAGTCCCCAGCAGTAAATCCTTGGGTACTGTCTGAAACTAACACTGTCCTACTATGGACTCAGGCTGAATCTCCAGCAGTCAATCCCTGGATAGATTCTATAGCTGGGACAGGCACTCAGTGGACCCAAACTGAATCTTTAGCAGTAAATTCTTGGACACAGCCTGTAGCTGATACAGTCGCAGTGTGGACTCAGGATGAGCCTCCATTATTACATTCCTGGAAAAAGTCTAAAACCCATACACTTATAACATGGACTCTGACAGAATCTTTAGCAGGAAATTTCTGGAGACCATATGAAACTGATAGTACCACATTGTGGACCAACAAGGGAAATCTAGAAATAAATCCTTGGGCACAATCTGAAACTGATGCAGTCACAGCTTGGACCCAGGCAGAAACTCCTGTAATAAATTCGTGGCCACAACCTATAGCTGAAATAATTTCATCATGGACTCTGGCTGATTCTCCAGCAGTAAATCCCTGGGCAGAGACTGTGTCTGATAGTGTTATACTGTCGACCCAGGTTGAATCTCCAGTAGTAAATCCATGGTCACAATCTATAGCTGATGTGGACACACTGCAGACCCTGACTGATTCCATAGCATTAAATCCCTGGACAGAGCCTGTAACCAATACTGTTATACAGTGGACCCAGAGTGAACCTCCAGCAGTAATTCAGTGGACAAAAACTGTATCCAGTACAGTCACATCATTTACTGGGGATGAATTTCCTGCATTAGAGACCTGGATAGACCCTTTGGCTGATATCACATTGTGGACCCAGACTGAAACTCTAGTAGTAAATCACTGGACAGAGACTATAGCTTCCACTCTCACATCATGGACCCAGACAAAATCACCAACAATAAATTCACTGACAGGGGTTGTAGCTGCCACAGTAATACCATGGAACCAGGCTGAATCTGCAGGAGTAAATCCCTGGATGGATGCTGTAGCTTTCACAGTATCACTGTTGACTCATGATCAGTCTCCATCAGTGAAAACCTGGACAGAGGCTGTAGCTTTCACAATCACACCACTAACTTATGCTGAATCTCTGGCAATAAAGTCTATGACAAAGGGAGTATCTGATGCAATCATACTGTGGAATCAAGCTGAATCACCTCTAGTATATCCTTGGACACAGTCTGAAACTGATGCAATCATACGGTGGACTCAGGGTGAATCTTTAAAAATAAATCCTTGGACAGAACCTGACACGATCACACCATGGACTCATGGTGAGTCCCTAGCAGTAAATCCTTGGACAGATGCTTCAGGTGACACCGTAACACCAATGAACCTAGTTGAATATCCTTCAGTACATCCCGGGACAACATTTGAAATGGGTAATGTCACATTCGGGACCCAGGCAAATTCTGAAATCATAAATCTCTTGACACAGACCATAGGGGTTGATATAGTCACACTGTGGACCCAGACTGAATCTCCTGCAGTGAATCCCTGGACACAGTCTGAAACTAACATAGTCACACCATGGACCCAGGCTGAATTTGCTACAGTAAATCTCTGGACCCAAGCCAAAACTAATCTAGTCACACTGAAGACCCATGGTAGATACCCAGCATTAAATCCCTGGGCAGATCCAGAAACTAACACATTTAGATCATGGTCTCAGGTTGAGTCTTCAAGAGTAAATCCATGGACACAGTCTGAAACTGACACAGTCACACCATGGACCCCGCCTGAATCTGCTACTATAAATCCCTGGACACAACCTGAAAATGTTATAGTCACACTGTTGATCCAGGCTGAATCTCTTGCAATAAATCCCTGGACACAGCCTGAAAATAACTTAGTCACACCATGGACCCAGAATGAATTGCCAGAAGAAAACACCTGGACAGAGGCTTTTTCTGAAACATTCTTACCATGGACTATGGGATTTTTTCCAACCATAAATCCCTGGATAGATACTGTATCTGTTAAAGTCATACCAGGAACCAAATATCAATTTTCAGAAGTAAAACTTTGGACACTGGAGTTGTCCAGTACTTTGGACACTGAGATAGGTACAGTGAAAATGTGGACTCAGTCAGAATCTCCACCCTTAATTCCCTGGACAGAAGCTATAGCTTCCATAGTCCCATTGTGGACGCAGACTGAACATCTAGCTGTAAATCAAAATCCTAGAGCTGATAGAGTCACAAAATGGACACAGACTAAATCTCCATCAGTAAATACTTGGACTCAGGTTCAGTTTCCAGCCATAAATCCATGGTCACAGTCTGAATCACCAGAAGTAAATATCTTGACAGAGTACAAATCTCCAGCCCTAAACCCATCAGTTGAGGGGAGTATAGGTACATTGTGGACTCAGACTGAATCTCCATTAGTAAATCCTTGGATAGAGCCTGTGGCGTCCATAGTCATTCCATGGACACAAGCTGAATATCCAGCAGTAAGTTCATTTACACAGACTGTAGCTGATACAATTATATTGTGGGCTCAAGCTGAATCTCTAGAATTAAATCCCTGGACAAAATCTATAGCAGATACAGTCACAGTGTGGGTCCTGGATGAATCTTCAAGAACAAAACCTTGGACACAGATGTTAGCTTCACCAGACAAAATTTGGACCCAAGCCAAAATAGTAAATCCATGGTCTACTTTTGGAATATTTCCACAATTCACCCAAATTCAATCTGTACTGGTTAAAACTGGAACACAATTTGAGAGTGACACAGTCATAGCATTAACTCAGGGTCAGGCTCTTGCTCTGAATCCCTGGACAGAGATTGTGACTTCCAGAGACATTCTATGGACACAAGCTGTACATCCAGCAGTAAATCCCTGGACAGAGTCTAGTGCATCCAGATTCATTCCATGGACGCAGGCTGTGCCTCCAGCAGTAAATCCCTGGACTGTCACTTCTACCCAAGCTGAACCTCCAGAAGTAAATCCCTGGACAGAGGTTGTCACTTTTAGAGTCACTCCATGGACCCAAGCTATACCTATAGCAATAAATATCTGGACAGAAGCTTTCACTTCTAGAATCATTCCATGGACGCAAGCTGTACCTCCAGCAGTAAATCCCTGGAGAGAAGCTATCACTTCCAGAGTCACTCCATGGAATGAAATTGTACCTCCATCAATCAATCCCTGGACAGAGGCCATCACTTCCAGAATCACTCCATGGACCCAAGTTGTACCTCCAGCAGTAAATCCCTGGACAGAGGCCATCACTTCCAGAATCACTCCATGGACCCAAGTTGTACCTCCAGCAGTAAGTCCCTGGACAGAAGCCATCACTTCCAGTGTCACTCCATGGACCCAAACTCTACCTCCAGCAGTCAATCCCTGGACAGAGGCCATCACTTCCAGAGTCACTCCATGGACCCAAGTTGTACCTCCAGCAGTAAATCTCTGGACAGAGGCCATCACTTCCAGAATCACTTCATGGACCCAAGCTGTACATCCAGCAAAATATACCTGGACAGAGGCTGGGGCATCCAGATTCATTCCATGGACCCAAGCTGTATCCCGAGCATTAAATCCCTGGATAGAGGCTATCACTTCCAGAGTTTCATCATGGACCCAAGCTGTAACTCCGGCAGTAAATCCCTGGACAGAGGCTGGTACATCTAGAGTCACATCATGGAACCAAGGTATGTTTCCAGTAATAAATCCGTGGACAGTGACGCTTGTTTCTACAGTCACACCATGGACCCAGGTTTCCTATCCACTAAATCCTTTGACAGAGACTAAAGCTTCTACAGTGAGCATATGGGCTCAGAATGAATATTCATTAATAAAAACCTGGACACATTCTGCTGTTTCCACGGTTACATCCTGGACTTGGGCTGAATATCAAGCAGTAAATTCTTATACACCAAGTATAGCTGATATGGTCATATTTTGGACAAGGCTTATATCTGAGTCTAAGGAATCCTGGATACTGCCTGAAGCTAGTATATTCAGTATTTCATTGCATCCTCAAAATGATACTACTCAATCCTTGATTCAAAGTGAAAATCAAACATCTTTTCTATCAACACATCCTGGAATTAATAATATCAATATATGGACTTTACTTGAATTTGAAACACAGGTATCATGGATAGTGCCTTTGTCTCAAGAAGCCAGACTCTCGCCCCTATCTGAAACTGATATTAGCATATATTGGTTTAAAACTGAAACAGAGAGAGTAAGAACCTGGGACCACTCAGAATTTCAAACAGTGAGTACTTTGACACAGTTTGAAATTGGGGGATTTGAGCCCTTGGCCAAACATGAAATTCCTGCAGTCATATCATGGGTTCCAACTAAAATTGGTGTATTCCCCCTCTGGAATAAGTCTGAAAGAGACAAAATAAGAACCTGGACCCTTTCTGGAGATGATGTCTTGCCATCATGGAGTCAGGTTGAAGCTAGTATATTCAGCCTCTGGAGCCAGTCTAAAAGTAATACAATCACACCCTGGATCCAGGCTGAGACTCAGTCAGTCAGTATCTGGACTGAAGGAAATTCAGGCACATTTTGGCACCTGAGTCAAAATAATGTAGTTAAGCCCTGGTCCCACCTTGAATCTGAAATGACATTTTCCAGGATTCAAAATGGTATAAATAGTTCTTGGACTCAGTATGAAACTAGTACTGTCAGATCTTGGACCAAGCTTGAAATTAGTACAGTGCAACCTTGGAATCAATTTGAAACTGTTACAATTAGATCATGGACCCAGTCTGAAAATGTAGAAATATACCCCCTTACCCAGAAAGAAGCTGGTACAGTAATAAGACATTGGTTCCAGACTCAAATGGATTCAATAAAACCTCGGAACCAGTCTGAAACTAATACAATTAGATCATGGACTCAGACTGGAACTGAAACAATCCAAATTTGGACCCAGACAGAAAGGCAAATAGTAAAACCTCCAACTTTATCTGAGATTGATACAATTACATCTTGGTTACAGACTCAGAGTGATACAAATAGACCCTGGATTAAATCTGACTCCTTGTCTGCCAACCCCTGGAGTCAGGCTGAAGTTGGTACAAATCACCCCTGGACTCAGCAGAGAGGTACTGTGAATCAACCCTGGACCTACTCCGAAATCCAGAAAGTCAGACCTTGGATGAAGCTAGAAGCTGATGCACTTAAATCTTGGTTCTACATGCAAATGAATAAAATCAGACCATGGACCAATTCCGAATCTCAGATCTTCAGCTCCAGGTTGCAGCCTGAAGTTGGTATGGTTCACCCTTGGATCCATCCTGAAACTCAAGCAGTCAGATCCTGGGCCCAACCTGAA

>ZCA Sea lion

ATGGATCCAGTCAAAGGAGACATAGGAGTCATCCTGCTACAGTACCCTATCAGGGTGGGGGGAATACCACTTTATCACACTTATAACATCTTCTGGAAGAGCTGTTATAACTGCCAGTACAGACACTGCAGGGTATACCAATATCAGAACCACAATAACTTTGGAACCAGTATCAAAAAGCTGTCAGTTAAGCTGCTGGACCTCTCATTCTGCCACCATCAATATATCCACCTAACTAAAAGTAACAACTTATGCATCTGGAGTCAGCCACAAGGAGACTGCTGGGTACAGCAGGGTAGTCCTGTTCTCTGCCTCTGTGGCAACCACTGGGAACTGGTAGGCCTGGTCAGTGAATCCTCAATGGCCTGTTATGACCCTGTTCTTGTCATCAAGACAGCCCCATATTTATCTTGGGTGAAATGGCTTATCAAGACATCTCAGAAGCCACTGGATCCTATTTGTTCCCTACCCTGCAATTTTACTCCTGGGGTAGAACATCGTCCACAAGACAGGCTTAGCCTGAGCAGGGGTACTGCCATTTTGACCTCCCACAGACTCTCTGTACAGTCACGGAAGAGAAGATTAGGCACTTTCCCACTGAACAGACAGCACCGGAATCCTCCTCCAGTATTTTTTCATTTAAATAATAGAGACTCTTTTCCTGGCAGTAGACAGTTACACCTTCAAGCTAGTCAGCTCTCCTCAACTAGCAAATTCCCAATGATACAATCTTGGACGTCTCTTGTTACCAAACAGCGGGATCCTTCTGATATTTCTGAGCCCTGGAATACCCCAATAGCTGGTACTTCTGAAACTTTGGTTCTTTCTGGACCCCCAAAGACTCCAACACCTGATAAATCTATACCCTGGGGCCTTCCTCAGAAAGATACAATGAAATATCAATACCAAACGATGACCAATTCAGTAAACTCCTGGGTTAATCCTTTAGCTGGTATCATTGGGCTTCATACTCTACCATTAGTTAGTTCTGCTATATCCTGGGTTTTGTTTTCAAGTGGCATAGATGGATCCCACCTTCTTTCTGGGGTTAATACCGTACAGTTTCAAGTTCAGTCTAGTAAGGTTCCTTTCCATGGTCAACTTCTAGCTAGACCCTGGCTAGAAATTACCCCTGACATTGGACCTTGGACACATTCTGTACCTGATAAAACTGGAACAGTGATGCAGATACAATCAAGTGAAGAGAATATTGGAAGCCAAATACACCATGTAGTCGATAGAGTTCACACTGCTTTTAAACCAGTAACTTATAACTTGAATGCATGGGTTCCTTTAACAGTTAATAAAAATGAATTCTGGACTCATTCCACGCTAAATGCAGATGGATCTCAGTATCCTACATTAACTCTTACGTTGGAACCCTGGTTTCAGTCAGTCTTAAATTTAGATGGATCCCAAGAACTTACAGAAAAGACTAATGAATACTGGATTCTTCCTGAATCTAAGTCAGTTCAATTGTGGACTTCTTCAGCACTTAATATGCCTTTCACTTGGATACCATCTGCAAGCAACACTATTAAGTCTTGGGCACAATATAATACCAGTCTGAGCAAAGCCTCAAGTCAAATGGAGAGAATAAGTCCATGGAGTAAACGTGAATCTTTTATGGTCAAACCCCAGATTCAAACTGCGGATACAACCTGGTTTTTGACACATATTATTACAAATGTAATTAAGCCTTTTTTTCAGTCTAAAGCTGATACAACCAGGCCCTGGAGTAAACCTGAAGCTGCCATAACCCAAACCTGGACCCAGCCAGAAACCCAGGCAAGAAAACCACTGACTCAGCTGAAAGCAGAAATCAGACGATGGTTACAGATTAAAACTGAAAGAATCAGACGCTGGATTCAGCCTAAGTTTCAAATAGTCAGACCTAGAGCCCAGACTGAAAATGGTAAAGACAAACATTGGACCCAGTCAGAAGCACATATAATTAGATCCGTGACACACACTGAAATTGAAACCTTCAGACTTGGGAACAAGCCTAAAGCTGGTACAGCCAGATCCTGGTTTTGGACTCAGTCTAATCAAATGAGAGCAAGGTCTCAGCCAGACTTGCAAACACTCTACCCTTGGACTCAGCCTGAAGTTGACATAGTGAGACCATGGACTCAGTCAAAAGCTAGTAGCATCCAACCATGGATGAAGCCTGAAGCATTGACATTCAGAATCTGGAGGCAGTTTAAAGTTAATACAATTACGCCCTGGACAGGGCCTGAAGCTGATATAGCTAGACTTTGGTTGCAAATACAAACCAATACATTCAGAACATGGAACCAACTTGAATCTCAGACAACCATTTCCTGGTCTGAGCTTGAAGCTGATAGAGTCAGACCCTGGTTGTATACTCAAATGCATACATTCAAACCTTGGATTGAGACAGAATTTCAAACAGCCCATTCCTGGACCCAACCTGAAGGTGATATACCCAGGCTTAGGACTAAATCTGAGGCTGACAATGTCAGACATTGGTTCCAGACTCAAGTGGAGACACCCACAATGTGGACAGAGCCAGTATCCCAAGTAGCCCACCACTGCATACAGTCCAAAACAGAAATAGTTAGGCCCTGGAACCAACCTGTGGCAGACAAGCTAAGAGCCTGGATACAACATGACATTTACACTGTAAGGCCCTGGGATGAGCTTGAAGGTGATAAGGTTAGATTCTGGACACAATCTGAATCTGACATAAGACGTTGGATTCAGCCAGATGTGGGTATAATCAATCCCAGGGCACAAAATGAAGCTGATACATCAACACCATGGGCATGGGGACAGGCTGAGACTCCAGAAGTTAATCCCTGGGCACAGTCTGAAACCGAAGCAGTTATACTCTCAAAACAGGGAGAAGCTTCAGCAATTAGTCACCAGACAGATATTTTAGCTGATACTGTCACAACATGGGCAAAGACTGAATTTCCAGGACAAAGGCTCTTGATGTATCCTTTGTCTCGTGTAGTCACACAGTGGACTCAGCCTGAACTGCCAGTGGCAAATCTCTGGACACAACCTGAAAATGTTACAGTCACACCTTGGATCCAGGATGAATCTCTTGCAGTAAATACCTGGACACAGACTGAAAATAACACAGTCATACCATGGACTCAGAATGAATCCCCAGAAGAAAATACCTGGACAGAGGCTGTTTCTGAAACAGCCATACCATGGACCATGGGATTTTTTCCAACCATGAAGCCCTGGATAGAGACTAAATTTGATAAAGTCACACCAGGCACCAAATCTCAATTTTCAGAAGTAAAACTTTGGACACAACAGTTGCCCAAAACATTGGACACTGAAACTGGTACCGTCAAAAGGTGGACTCAGTCTGAATCTCCACCCTTAATTCCCAGGATAGAAGCTATAGCTTCCATAGTCCCATTATGGACCCAGGCTGAATCTCTAGCTGTAAATCAGTGGACACAACCTATAGCTTATAGAGTCACAGAATGGACACAGACTAAATCTCCATCAGTAAATACTTGGACTCAGGTTCAATTTCTGGCAGTAAATCCATGGACACAGTCTGAATCAGAGTACAAATCTCCAGCCCTAAAACAGTCAGTTGAGCCTGAGGCTAGTGTAGTCATATTGTGGACTCAGACTGAATCTCCAGAAGTAAATCCTTGGATAGAGCCTGTAGCTTCCTTAGTCATGCCATGGACACAAGCTGAATATCCAGCAGTAAGTTCATTGACACAGGCTGTAGCTGATACAATTATCCTGTGGCCTCAGGCCGAATCCCAAGCATTAAATCCCTGGACAAAGTCTGTAGCAGATACAGTCACAGTGTGGGCCCTGGATGAATCTTCAAGAGCAAAACCTTGGACACAGATATTAACTTCATCAGATATATTTTGGACCCAAGCCAAAACAGTAATTCCTTGGTCTACTTTTGAAATATTCCCACAATTCACCCTTATTCAATCTGTAGTGGTTAAAACTTGGACACAATTTGAGAGTGACACAGTCACAGCCCTAACTCAGGGTCAGGCTCTTGCTGTGAATCCCTGGACAGAGATTATCACTTCCAGAGTCACACCATGGATCCAAGCTGAATTTCCAGCAGTAAATCCCTGGACAGAGGCTGTTGCTTCCAGAGTCACATCATGGACCCAAGCTATATCTGTAGCAGTAAATCCAGGGACAGAAGCTGTCACTTTGAGAGACATACCATGGATCCAAGTAGTATTTCCAGTAGCAGTAAATACCTGGACAGAGGCTGTCACTTCCAGAGTCTTTCCAGGGACCCAAGCTGTACTTCCAGTAGTAAATCCCTGGACAGGCACTGGTGCATCCAGAGTCATTCCATGGACACAAGGTGTACCTCCAGCAATAAATCCCTGGACAGAGGCTGGTGCATCCAGAGTCACATCATGGAGCAAAGCTCTTCCTCCAGCAATAAATCCCTGGACAGAGGCTGGTACATCCAGAGTCACATCATGGACCCAAGCTGTATCTCCAGAAGGAAATCCCTGGACAGACGCCGGTGCATCCAGAGTCATGCCATGGACCCAAGCTGCACCTCCAGGAATAAATCCCTGGACAGAAGCTGATGCATCCAAAGTCACTCCATGGACCCAAGCTATCCCTTCAGAAGTAAATCCCTGGACAGAGGCTGGTGCATCCAGAGTCACTCCATGGACCCAAGCTGTACCTCCAGCAGTAAATCCCTGGACAGACACTGATACATCCAGAGTCACATCATGGACTCAAGCTGTACCTCCAGCAGTAAATCCCTGGACAGAGGCTCTTGCTTCCAGAGTCATGTCATGGAACCAAGGTGTGTTTCCAGCAGTAAATCCCTGGACAGTGACACTTGTTTCTACCGTCACAACATGGACCCAGGCCTCCTCTCTTCTAAATCCTTTGACAGACACTAAAGCTTCTACAGTGAAAATATGGACTCATAATGAATATTCATTAGTAAAATCCTGGACACATTCTGCAATGTCCACAATTATATCCTGGACTCAGTCTGAATATCAAGCAGTAAATTCTTATATACCGAGTGTAACTGATACGGTCATATTTTGGACTGTACTTATATCTGAGTCTAAGAAATCATGGATACTGCCTGAAGCTGGTGTATTCAGTATTTCATTGCATCCTCAAAGTGATTCTACTCAATCCTTGATTCAAGGAGAAAATCAAGCATCTCTTCTGTCGACACATCCTGGAATTAATACTGTTAGTACATGGCCTTTGCCAGAATTTGAAACACTGGTATCATGGAGAGTGCCTTTGTCACAAGCAGCCACACTCTTACCCCTATCTGAAACTGATATTAGCCGATATTGGTTTAAAACTGAAACAGAAAGAGTAAGTACCTCGGCCTGCTCAAAACTTCAAACAGTGAGAACTTTGACCCAGTTTGAAACTGATAGATTTGAGCCCTTGGCCCAACATGAAACTCCTACAGTCATATCATGGATTCCAACAAAAACTGGTATATCCCACCCCTGGAATAAGTCTGAAAAAGACAAAGTAAGAACCTGGACCCTTTCTGGAGGTGATGCCTTGCGACCATGGATTCACAGTGAAGCTAGTATATTCAACCTCTGGATCCAGTCTAAAAGCAGTACAGTCACACCCTGGACCAAGCCTGGGTCTCAGTCAGTCAGTATCTGGACTGAAAGAAATACAGGCACATTTTGGTACCTGACTCAAAATGATGCAGTTAGGCCTTGGTCCCAGCTTGAATCTCAAATGACATCTTCTGGGACCCAAAATGGTATAAGTAGCTCTTGGACTCAGTATGAAACTAGTACAGTCAGATCCTGGACCAAGCTTGAAATCAGTACAGTGCAACCCTGGATTCAGGTTGAAACTGCTACAATTAGATCACGGACCCAGTCTGAAAATATAGAAATATACCCCCTGACCCAGTTAGAAGCTGGTACAGTAATAAGACACTGGTTCCAGACTAAACTGGATTCAATAAAACCTTGGAACCAGCCTGAAGCCAATACAATTAGATCATGGACTCAACCTGAAACTGAAACAATCCAAATTTGGACCCAGACAGAGGGCAAAGTAGTAAAACCTCCAACTTCATTGGAACTTGATACAATTACATCTTGGTTACAGACTCAAAGTGATACATTTCAACCCTGGATTAAATCTGACTCCCAATCTGTCAGTCCCTGGAGTCAGGCTGACGGTATAAATCACCCCTGGATTCAGCAAAGAGGTACTGTGAATCAACCCTACTCAGAAATCCAAACAGTCAGACCCTGGATGAAGCTAGATGCTGATGCACTTAGATCTTGGTTCTACATTCCAATGAATAAAGTCAGACCATCGACCAATTCCGAATCTCAGGTCTTCAGCTCCGGGTTGCAGCCTGAAGTTGGTATGTTTCACCCTTGGATCCATCCTGAAACCCAAGCAGTGAGATCCTGGGCCCACCCTGAA

>CFA Dog

CTTCTGGTCACATGGCAGACCCAGGGTAGATATCCAGCATTAAATCCATGGATACAGTCAGAAGCTGACACATTTACATCATGGTCTCAGGCTGAGTCTCCAGCAGTAAATCCACAGGCACAGACTGAAAGTGACACAATCACAACATGGACCCAGGCTAAATCTCCTGCAGCATATCTCTGGACACAGCCTGAAAATGTCACGGTCACACTGTGGATCCAGGCTGAATCTCTTGTAGTAAATCCCTGGACACAGCATGAAAATAACACAGGCACACAATGGACCCAGAATCAATCTTCAGAAGAAAACACCTGGGCAGAGGCTGTTTCTGAAACTGTCATACCATGGAAAATGGGATTTTTTCCAAATATGAAACCGTGGATAGAGATGATATCTGATATAGTTACTAAATCTCAATTTTCTGAAGTAAAATTTTGTACAGTGCCAGGGTCCAAATTGGACACTGATGTAAGTACAGTAAAAAAGTGGACTCGGTCTGAATCTCCACCCTTAATTCCCTGGACAGAACCTCTAGCTTCCATAGTACCACTATGGACTGAATCTCTAGCTATAAATCACTGGACGCAGCCTATACCTGATACAATAACAAAATGGACACAGACTGAATCTCCATCAGTAAATACCATTCAATTTCCAGCAGTAAATCCATGGACACAGTCTGAATTACCAAGAGTACATTTCTTGACAGAGTACAAATCTCCAGCCCTAAAACCATCAATTGAGCCCGAGGCTAGTATAGTTACATTGTTGACTCAGACTGAATCTCCAGCAGTAAATCCATGGATAGAGCCTGTTGCTTCCATAGTCATGCCATGGATACAAGCTGAACACCCAACAATAACCCCAATGACACAGGCTGTAGCTGAAACAATTATCCTGTGGACTCAGAATGAAAATCTAGCATTAAATCCCTGGACAAAGTCTGTTGCAGATACAGTCACACTTTTGACCCTGGATGAATATTCAAGAGCAAAACTTTGGACACCTATATTAGATTCCTCAAATATATTTTGGGCCCAAGCCAAAAATTCAGCAATAAATCCTTGGTCTGATTTTGAAATACCCTCACCATTGACCCAATTACAATCTGTAGTGGTTAAACCATGGACACAATTTGAGAGTGACACAGTCACACCATTAATGAAGGTTCAGGATCTTGCTGTAAATCCCTGGTCAGAGATTATTGCTTCCAGAGTCACAACACAGACTCAGTCTGTATCTCCAGCAGTAAACCCCTGGATGGAAGTTGATGCAACCAGACTCCCATCATGGACCCAAACTGCAACTCCGGCAGTAACAACCTGGACAGAGGCTGATGTATCCAGAGTACTGCCATGGACCCAGTCTCTACCTCCAGTAGTAAATCCATGGACAGAGGTTTATGCATCCAGAATCACACCATGGACCCAAGCAGTAGCTCCAACAGTAAATCCCCAGACAGAGACTGTCAGTTCCAGATTCATGCCATGGACCCAGGCTGTCCCTCCAGCAGTAAATCTGTGGACAGAAACTAATGCATCCAGAGCTATACCATGGACACAGGATGTATCTCCATCAGTAAATCCCTGGACAGAGGCTGTCAGTTCCAGATTCATGGCATGGATTCCAACTGTACCTCTAGCAGTAAATTCCTGGACAGAGGCTGTTGGCTCCAGATTCATGCCATGGACCCAAGCTGTACCTCCAGCAGGAAATCCCTGGACAGAGACTGTTGGTTCTAGATTCATGCAATGGACCCAAGATATACCTCCAGCAGTAAATCCAGGGACAGAGGTTGTTGGTTCCAGATTCATGACATGGACTCAACCTGTAATTCCAGCAATAAATCCAGGGACAGAGATTGTTGGTTCCAGATTAATGACATGGACCCAAGCTGTACCTCCAGCAGGAAATCCCTGGACAGAGACTGTTGGTTCTAGATTCATGCAATGGACCCAAGATATACTTCCAGCAGTAAATTCCTGGGCAGAGGCTATTAGTTCCAGATTCATGACATGGACTCAACCTGTAATTCCAGCAGTAAATCCAGGGACAGAGGTTGTTGGTTCCAGATTCATGACATGGATTCAATCTCTAATTACAGCAGTAAATCCCTGGACAGAGGCTGATACATCCAGATTCATGCCATGGACCCAAGCTGTACCTCCAGGAGTAAATCCCTGGGCAGAGGTAAATGCATCCAGAGTCCTGCCATGGACCCAAGCTGTCTTTCTGGAAGGAAATCCCTGGACAGAGGCTGATGCATCCAGAGTCCCAGCATGGATGCATGTTGTACCTCTAGCAGTAAATCCATGGACAGAGGCTGTCAGTTCAAGAGACATGCTATGGAACCAACCTGTACTTCCAGCAGTAAAACCCCAGACAGAGGCTGTAACTTCCAGAGTTTCACCATGGACACAAGCTATAACTCCACCAGTAAATACCTGGACAGAGGCTGTTGTATCCAAAGTCATAACAGGGAGTCAACCCGTACTTCCAGCTGGAAATCCCTGGACAGAGGATGATGTATCCAGAGTCATGCCATGGACCAAAGCTTTACATCCAGCAGGAAATCAGGCTGTTGGTTCCAGATTCATGCCATTGACCCGATATATACCTTCAGCATTAACTCCATGGACAGAGGCTGTCACTTTCAGAGTCTCACCATGGACCCAAATTGTACCTTTAGCAGTAAATCCCTGGACAGAGACAATAACTTCAACAGGCATGCCATGGACCCAACCTGTACCTCCAGCAGTAATTCCCTGGGCAGAAGCTGTACCTCCCAGAGTCCCGCCATGGACCAGAGCTGTATTTCCAGCAATAAATCCCTGGACAGAGGCTGATGCATCCAAGGTCATGCCATGGACCCAGTCTGAACCTCCAACATTAAATCCCTGGACAGATATTTATGCATCCAGAGTCATGCCATGGAATCCACCTGTACCTCCAGCAGTAAATCCCTGGACAGAGGCTGTTGGTTCCAGATTCATGCCATGGACCCAGGCTGTCCTTCCAGTAGTAAATCACTGGTCAGAGGCTGATGTATCCAGAGTTCCAGCATGGACCCAAGCTGTACTTCTAACTCTAAATCTCTGGACAGAGGCGGTGGGTTCCAGATTCATATCATGGACTCAAACAGCATCTCCAACAGTAAATCCCTGGACAGAGGCTGTTGGTTCCAGATTCATGCCATGGACCCAACCAGTAACTCCAGCAGTAACTCCCTGGACAGAGGCTGATGCATCCAGAGTCTTGCCACGGACCTTATCTGTACCTCTGGCTGTGAATCTCTTGACAAATGTTTCTGGTTCCAGATTCATGCCATGGACTCATCCTGTAACTCCAGCAGTAAATCTCTGGAAAGAGGATTTCAGTTCCAGATTGATGCCATGGAACCAAGCTGTACCTCCAGTGGTAAATCCCTGGACAGAGTCTGTCACTTCCAGAATCACACCATGGACTGAACCTGTAGCTCCAATAGTAAATCCTTGGTCAGAGGCTGTCGGTTCCAGATTCATGCCAAGGACCCAAACTGTACCTCCAGCAGGAAATCCCTGGATAGATGCTGGCACTTCCAGAGTCACTCCATGGACCCAAGCTGTACCAGCCACAGGATATCCTTGGACAGACACTGATGCATCCAGAATCACACCATTGAACCAAGCTGTAACTCTAGGAGTAAATCCCTGGGCAGAGCCTATTGCTTCCAAAGTCATACCATGGACACAAGGTATATCTTCAGCAGTAAATCCTTGGGCAGAGGATGTTCCTTCCAGAGTCATGCCATGGACCCAATCTGTACATCCAGGAGTAATTCCCTGGACAGAGACTGATGCATTCAGATTCATATGGAACCAAATTGTACCTCCACTAGTAAATTCTTGGTCAGAGGCTGATGTATCCAGAGTCATGCCATGGACCCAAGCTGTACCTCCAACAATAAATCCCTGGACAGAGGCTGATTCATCCAGAGGCATGCCATGGGCCCAATTGGTACCTCCAGCAGTAAATCCCTGGACAGAGGCTGATGCATCCAGAGTCATATCATGGACCCAAGTTATACCTCCTGCAGGAAATCCCTGGACAGAGGCTGTTAGTTCCAGATTCATGCCATGGACTCAACCTATACCTCAAGCAGTAAATTTCTGGACAGAAGTTGGTGCATCTAGGGTCCCACCATGGTCCCAACCTGTACCTCCAACAGTAAATCCTTGGACAGAGGGTGCTGCATCCAGAATCACTCCATGGAATCAAGGTGTAACTCTAGCAGTAAATCCCTGGACAGAGGCCATTGGTTCCAGATTCATGTCACTGACCCAACTTGTGCCTTCAGCAGTAAATGCCTGGACAGATACTGATATATCTAGATTCATGTCATGGATTCAACCTGTACCTACCTTAGTAAATCCCTGGATAGAAGCTGTTGGTTCCAAATTCGTGCCATGGACCCAACCAGTACATCCAACAGTAAATCCCTGGTCAGAGGCTGATGCATCCAGATTCATGCCATGGACCCAGCTTGTACCTCTAACAGTAAATCCCTGGTCAGAGGCTGATGCATCCAGATTCACGCCATGGACCCAACCTGTACCTCCAGCAGTAAACACCTGGACATATATGAATGTATCTAGAATCATTCCATGGTCCCAAGATGTGCCTTCAGCAGTTAATCCCTGGACAGATGCTGTTGCTTCCAGATTCAATCCATGGACCCAAACTGTATCTCCAGCAGTTAATCCCTGGACAGAGGCTATTGGTTCCAGATTCATGCCATGGACCCAACTTGTACCTCTACCAGCAAATCACTGGTCGGAGACTGATGCATCCAGAGTCATGGCATGGACTCAACCTTTATTTCTAGCAGTAAATCTCTGGAGAGAGACTGATGCATCCAGATTCACACCAAGGACCCAACCTGTACCTCCAGCAGTAAATCCCTGGACAGATCCCAATGGATCTAGAGTTATGCAATGGACCCAACCTCTACTTCCTGTAGCAAATACATGGACAGAAGCTGATGCATCCAGAGTCATGCCATGGACCCAACCTTTACCTCTAGCAGTAAGTCCCTGGACAGAGACTATTGGTTCCAGATTCACTCCATGGACCCAACCTTTACCTCCAGTAGTAAATCCTTGGACAGAGGCTGATGCATCTAGAGTCACACCATGGATTAAACCTTTACTTATAGGAGTAAATCCCTGGACAGATCCTGATGTATCCAGAGTCACACCATGGACTTATCCTTTACTTCTTGCAGTAAATCCTTGGAGAGAAACTGGTACATCCAGATTCATGCCATGGACCCTACCTGCACCTCTAACAGTAAATCCCTGGTCAGAAATTGATGCATCCAGAGTTATGCCATGGACCCAACCTATACCTCCAGCAATAAATCCCTGGATAGTGACAGTTGTTTTTACCTTAACACCATGGACCCTGGATGCCTCTCTCCTAAATCCTTTGGCAGAGACTAAGGCTTCCTCAGTGGGAATATGGACTCAGAACAAATATTCAGTAATTAAAACTTGGACAAAATCTGAAGTTTCCACACTTACATCCTGGACAGATACTCAGACAGAATATCAAGCAGTAAATTCTTATATATCTTATATATCAAGTGTAACTGACACAGTCACATTTTGGACAATGCCAAAATATGAATCTAAGGAAACTTTGATACTGCCTGAAGCTCATATATTTAGTATATCATTGCATCCTCAAAGTGATACTCAATCCTTGATCCAAGTAAAAAATATAGCATCTCTTCTGTCAATATACCCTGGAATTAATAGTGTCAATACATGGTCTTTGCCTAAATTTGAAACAGTGGCATCATGGATAGTGCCTTTGCCTCAAGCAGCCAGATTTGTGCCCCTACCTGAAGCTGATATTAATAGAAATTGGTTTAAAATGGAAACAGAAACATTAAGAAATTGGACCCATTCAGAATCTCAAAGAGTGAGCACTTTGGCACAGTATGAAGCTAGTATGAAGCCCTTGGCCCAACATGAAAATTCTACAGTCATATCATGGATTCCAACTGAAACTGGTATATTTCACCCCAGGAATAAATCTGAAAGGGACAAAGTAAGAACCTGGACCCTTTCTGAAGGTAATGCTTTGCGACCATGGATTCAGATTGAAGCTAGCATTTTCAACCTCTGGACTCAGTCTAAAAGTAGTACAGTCACATCCTGGACCCAGCCTGAGTTTCAGGCAGTCTTTACCAGGACTGAAGGACTTATAGGTACATTTTGGTCCATGAATAAAAATGATGCAGTTGGGCCTTGGTCCCAGCTTGAATCTCAAATGACATCTTTCTGGACCCAAAGTGACATAACCAGCTCTTGGACTCAGTATAAAACTAGTACATTCATATCCAGGACCAAGCTTGAAATCAGTACACTGCAACCCTGGATTCAGTTTGAAACTGCTGCAATTAAATCATGGATTCAGTCTGAAAATGTAGAAATATACCCTTTGACCCAGACAAAAGCTGATACAGTAATAAGACCTTGGTTGCAAATTCAAATAGATTCAATACAACCTTGGAATCAACCTGAAACTAATACAATTAGACCATGGACTCAACTTGAAACTGAAGTAATCCAAATTTGGACCCCCACAGAAAATCAAGTAGTAAAACCTCCAACCTTACCTGAAATTGATACAATTACATCTTGGTTACAGACTCAAAGTGATACAACTAGACCCTGGATTAAATCTGACTCCCAGTCTGTCAGTTCCTGGGGTCATGCTGACATAGGCATAGTTCAGGATTGGATTCAGCAA

>VUL Fox

ATGATGAGATCTCCATTTTGCTTCTACCAGTTTTGGGGGTTGTTTTTTCACTGCTGTGGTGTTCCCAATTCTCAAAAGCAGTGGTTAAGCTCATTGACCTCAACGATTGCCCCATTGACAGCGTCCAGCAACATCCCATGGTTAGTGTCTATGGCTGAAACCTGCCAGGGCATTATTCTGAGTCAGTGGTGGATCCTCTCCACAGTCAGCTGTCTGAGTAAACTGAAGCATTTGCACTCTGACATTTCAGGAGTCCTTGCCCAAGAAGATATCTTACTTGGCCATAAAATATGCCTGCACCCCAGTTTTGATCCACAAGTTAGAACAGATCCAGTCAAAGGATACATAGGGGTGATGCTCCTGCAGTACCCTATCAGGCGGGAAGAAATATCTCTTTATTCCACTTATAACATTTTCTGGAAGAGCTGTTATAACTGCCAATTCAGACACTGCAGAGTATACCAAGATCAGAACCACAATAACTTCGGAACCAATATCAAGAAGCTGTCAGTTAAGCTGCTGGACCTCTCATTCTGCCACCATCAACATATTCAGCTGACTAAAAGTAACAATTTGTGCATCTGGAGTCAGCCACAAGAAGACTGCTGGGTACAGCAGGGTAGTCCTGTTCTCTGCCTTTTTGGCAACCACTGGGAATTAGTAGGCCTGGTCAGTGAGTCCTCAATGGCCTGTTATGACCCTATTTTTGTCATCAAGACAGCCCCATATGTATCTTGGATGAGATGGCTTATCAAGGCAACCCAGAAGCCATTGGATCCTATTTTTCCCCCACCCTGCAGTTTTACTCCTGGGGTAGAACATGTTCCACAAGACAGGCTTAGCCTGAAAAGGGGCACTGCCATTTTGACTTCCCATGGATTTTCTATACAGTCATGGAAGAGAAGATTAGGTACTTTCCCACTAAATAGACAGCGCCGGAATCCTCCTCCAGTATTTTCGAATTCAAATAATAGAGAGGCTTTTCCTGGCAGTACACAGTTACACCTGCAAACTAGTCAGCTCTCCTCAACAAGACTGATACTATCTTGGACCTCTCCTCTTATCAAACAACAGGATCCTTCTTATATATCTGAGCCCTGGAATACCCCAAGAGCTGGTACTTCTGAAACTTGGGTACTTTCTGGACCCCCAAAGTCTCCAGCATCTGATAAATTTATACCCTGGGACCTTCCTCAGAAAGATACAATGAAATATCAATACCAAACTATAACCAATTCAGTAAACTGGGTTAATCCTTTAGCTGTTATAATTGGGATTCACACTCTAACATTAGTTAATTCTGCTATACCCTGGGTTTTGTTTCCAAGTGGCATACATGGATCCCAACTTCTTTCTGGGATTAATATTGTACAGTCTCAAGTTCAGTCTAGTAAGTTTCCTTTCCATGACCAACTTCTATCTAGAACCAGACTAGATATTACCTCTGACATCGCACCTGGGATACACTCTGCACCTGATAAAACAGAAACACTGAAGCAGATGCAGTCTAGTGAAGAGAATATTGGAAGCCAAATACACCATGTAGTTAATATAATTCACACTACTTTTAAACCAGTAACTTTTAATTTGCATGCATGGGTCCCTTTAAGAGTTAGTAAAAATGAATTCTGGACGCATTCCACACTAAATGCTGATGTATCTCAGTATCCTACAGTAACTCTTACCTTTGAACCATGGTTTCACTCAGTCTTAAATATGGATGGATTCCAAGAACTTACAGAAAAGACTAATAAATACTGGATTCTCCCTGACTCTAAGTCAGCTCAATTGTGGACTTCTTTAGCACTTAATATGCCCTTTACTTGGGTTCCATCTACAAGTAACACTATTAAGTCTTGGGCACAATATAAGAGCAGTCTGATCAAACCCTCGAGTCAAATAGAGAAAATAACTCCACTGAGTAAGCGTGAATCTATTATGGTTAAACCCCATATTCAAACTGCAGCTGCAACCTGGTTTTTGATACGTACTATTACAAATATAATTGAGCCTTTCATTGAGTCTAAAGCTGATACAGTCAGACCCTGGACTCAGCCTGGAGCTAACATAGTCCAAACCTGGACCCAGCCAGACACTCAAGCAAGAAAATCACTGACTCAGCTGAAAGTGAATATAAGTAGACCACGGATACAGACTAAAACTGAAAGAATCAGACCCTGGATTCAGCATAAGTTTCAAATACTCAAACCTCGAGCCCAGACTGAAAATAGTAAAGACAAACTTCAAACTCAGTCAGAAGCATATATAATTAGATCCATGATCCACACTGAAATTAAAACAGTCAGATTTTGGAACAAACCTAAAGTTGATACAGCCATATCATGGTTAAGGACTAGATCTAATCAAATAAAAGCAAGATCCCAGTCAGACTCTCAAACACGTTACCCTTGGACTCAGCCTGAAGCCGGCATAGTGAGACCATGGACTCAGTCTGAAGCTGACAGTATCCAGCAAGGGATGAAGCCTGAAGGATCAACATTCAGAATCTGGACACGGTCTAAAATTAATACAGTTACACCCTGGACAGGGCCGGAAGCTGATGCAGCTAGACTTTGGTTGCAAATACAAACCAATACAATCAGAACTTGGAGCCAACCTGAATTTCAAACAACCATTTCCTGGTCTGAGCTTGAAACTGATAGAGTCAGATCTATCAGTCAAATACATACATTCAAACCTTGGAGTGAGACAGAAATTCAAACATCCCACTCCTGGACTCAATCTGAAGGTGATATAGCCAGACTCTGGACTAAATCTGAGGCTGACAATGTCAGACATTGGTTCCAGACTCAAGTGGAGACAACCACAATGTGGACAGAGCCAGTAACCCAAACAATCCACCACTGGATACAGTCTAAAACAGAAATAGTCAGGCCCTGGAGCCAACCTGCGGCAGATAAGCTCAGAGCTTGGATACAACATGAAATTTATGCTGTCAGGCCCTGGGATGAGCTTGAAGGTAATAAAGTTAGATTCTGGACCCAGTCTGAATCTGACACAAGACCTTGGATTCAGCCAGATGTGGGTATAATAAATCCCGGGGCACAAAATGTTGCTGATACATCAACACCATGGGCATGGCCACAGGCTGAGACTCCAGAAGTTAATACATGGGCACAGTCTGAAACTGAAACAGTTATACTCTCGACCCAAGGAGAAGGTCCAGCAATAAATCATTGGACAGAGATGTTAGCTGATACTGTCACAACATGGGAAAAGGCTGAATTTTCAGGACAAAAGCTCTTGGAACATCCTTTGTCTCATACAGTCACACAGTGGACTCAGCCTAAACTGCTAGCTGCAAATCTCTGGATGCAGCCTGAAGATGATTTAATCACACAGTGGACCCAGGGCGAATCTTCAGAAATAAATTCCTCAACAAAAATTATAGCTGACACAGCCATGTGGACACAAGCTGAATCTCCAGCAATGAAGCCCTGGATACAGTCTGATAATGATACAGACATATTCTGGACCCAGGATGAGTCCCTAGAAATAAATCCTTGGACAAAGTCTGTGGCTGATACAGTCACACAATGGGCCCAGGGTGAACCTTCAGAAGTACTTCCTTGGGTGAAAATTGTAGATGATACACCATGGATGCAGCCTGAATTCCCAGAAGTAAACCCCTGGACACAGCCTGAAAATGATACAGTCATACTATGGATACAGGGTGACTCTCCAGAAGTAAACCCCTGGACACAGTCTGAAAGTGAAACAGTCATACCATGGATACAGGGTGAATCTCCAGAAGTAAACCCCTGGACACAGTCTGAAAGTGATATAGTCATACCATGGATACAAGGTGAATCTCCAGAAGTAATCCCCTGGAGACAGTCTGAGAGTGATACAGTCATACCACTGATGCAGGCTGAGTCTCCAGAAGTAAATCCCTGGATACAACCTGAAACTAACAGAGTCCTACTGTGGACTCAGGCTGAATCTCCAACAGTAAATCCTTGGATAGATGCTGTAGCTGGGACAGGCACACAGGGGACCCAAGCTAAATCTCTAGATGCAAATTTTTGGCCACAGCCTGTAGGTGATACACTCACACTGTGTACTCAGGATGTGTCTCCATCATTACATCTGTGGAAAAAGTCTGAAACTCATACACTTACAACATGGACTCTGACAGAATCTTTAGTAGAAAATTCCTGGAGACAGTATGAAATTTATGGTGTCTCAACATGGACCAAAAAAGGAAATCTAGAAGTAAACCCCTGTGTACAATTTGAGACTGACACAGTCACAATTTGGACCCAGGAAGAAACTCCAGGAGTAAATTTGTGGAAACAACCTACAGCTGATATAGTTCCACCATGGACTCTGACTGAATCTACAGAAGTAAATCCCTGGAGAGACACTGTATCCAATAGTGTTACACAGGGTGTGGAGGCTGACTCTCCAGCAATAAATCCCTGGACAGAGACTGTACCTGATAGTGTTATACAGTGGGCTGAGGCTGACTCTCCAGCAGTAAATCGCTGGACAGAGACTATACCTGATAGTGTTATACAGTGGGCTGAGGCTGACTCTCCAGCAGAAAATCCTTGGACAGACACTGTACTTGATAGTGTTATACAGTGGGCTGAGGCTGACACTCCAGCAGTAAATCCTTGGACAGAAACTGTACTTGATAGTCTTATACAGTGGGCTGAGGCTGACACTCCAGCAGTAAATCCCTGGACAGACACGGTATCTGATAGTGTTATACAGTGGGGTGAAGCTGACCCTCCAGCAATAAATCCTTGGACAGAGACTGTACCTGATAATGTTATACAATGGGCTGAGGCTGAATCTCCAGCAGTAAATCCTCAGACACAATCTATAGCTGATGTAGACATACTGTGGACCCTGGCTGATTCTACACAATTAAACTCCTGGACAGAGCCTGTATCTGATAGGGTAATACAGTGGACCCAGAGTGAACCTCCAACAGTACCTCAGTGGACAGAAGCTGTTTCAGATACAGTCACATCATTTACTAATGAAGAATTTCCTGAAGTAGAGACCTGGACAGACCCTTTTGATGATGTCACACTGTGGTCTCAGACTGAAACTCTAGTATTAAAACCCTGGACAGAGACAGTAGCTTCAACGGTTGCAACATTGACCCAGACAGAATCACCAGCAATAAATCCAATGACAGAAGCTGTATCTACCACAGTAATACCTTGGAATCAGGCTGAATCTACAACAATAAATCCCTGGATAGATGCTAGAGATTTACTTTTAACACTGTTGACACAAGGTCAGTCTTCACTAGTGAAAACCTGGACGGAGGCTTTAGCTTTCATAATCACACCACTGACTCAGGCTGAATCTCTAGCAATAAAGTCTGTGACTCAGGGTATATTTGATACAATCATACTGTGGAACCAAGCTGAATCACCTTTAGTACGTCCTTGGACACATTCTGAAACTGAAACACAATGGACTCAGGGTGAATCTCTAAAAGTAATTCCTTGGACACAATCTGTAGCTGTCACAGTCACACCATGGAATAGTGGTGAGTCCCCAGCAGTAAATCTCTGGACAGATGCTATAGGTAACACAGTAACAACAATGAATCTTGATGAAGAAATTGGTGCTGTCACACTCTGGACCCAGGCAAATTCTGATATCATAAATCTCTTGTCACAGACTATAGTTGATATCGTCACACTGTGGACCCAGGATGAATCTCTTGCAGTGAATCCCAGAACACATTTTGAAAGTGATACAGTCACAGCATTTACTCAGGATGAATCTGCTACAATAAATCCCTGGATCCAGCACAAAACTAATCTGGTCACATGGCAGACCCAGGGTAGATATCCAGCATTAAATCCATGGATACAGTCAGAAGCTGACACATTTACATCATGGTCTCAGGCTGAGTCTCCAGCAGTAAATCCATGGGCACAGACTGAAAGTGACACAATCACAACATGGACCCAGGCTGAATCTCCTGCAGCATATCTCTGGACACAGCCTGAAAATGTCACGGTCACACTGCGGATCCAGGCTGAATCTCTTGTAGTAAATCCCTGGACACAGCATGAAAATAACACAGGCACACCATGGACCCAGAATCAATCTTCAGAAGAAAACACCTGGGAAGAGGCTGTTTCTGAAACTGTCATACCATGGACAATGGGATTTTTTCCAAACATGAAACCATGGATAGAGATGATATCTGATATAGTTACACCTGGCACTAAATCTCAATTTTCTGAAGTAAAATTTTGGACAGTGCCAGGGTCCAAATTGGACACTGATGTAAGTACAGTAAAAAAGTGGACTCAGTCTGAATCTCCACCCTTAATTCCCTGGACAGAACCTATAGCTTCCATAGTACCACTATGGACTGAATCTCTAGCTGTAAATCACTGGACTCAGCCTATTCCTGATACAATAACAAAATGGACACAGACTGAATCTCCATCAGTAAATACCATTCAATTTCCAGCAGTAAATCCATGGACACAGTCTGAATTACCAGCAGTACATTTCTTGACAGAGTACAAATCTCCAGCCCTAAAACCATCAATTGAGCCTGAAGCTAGTATAGTTACATTGTTGACTCAGACTGAATCTCCAGCAGTAAATCCATGGATAGAGCCTGTTGCTTCCATAGTCATTCCATGGATACAAGCTGAACATCCAACAATAACCCCAATGACACAGGCTGTAGCTGAAACAATTATCCTGTGGACTCAGAATGAAAATCTAGCATTAAATCCCTGGACAAAGTCTGTTGCAGATACAGTCACATTTTTGACCCTGGATGAATATTCAAGAGCAAAACTTTGGACACCTATATTAGATTCCTCAAATATATTTTGGGCCCAAGCCAAAAATTCAGCAATAAATCCTTGGTCTGATTTTGAAATATCACCATTGACCCAATTACAATCTGTAGTGGTTAAACCATGGACACAATTTGAGAGTGACACAGTCACACCATTAATGCAGGTTCAGGATCTTGCTGTAAATCCCTGGTCAGAGATTATTGCTTCCAGAGTCACAACACAGACTCAGTCTGTATCTCCAGCAGTAAACCCCTGGATGGAAGTTGATGAAACCAGACTCCCATCATGGACCAAAACTGCAGCTCCGGCAGTAACAACCTGGACAGAGGCTGATGTATCCAGAGTACTGCCATGGACCCAGTCTCTACCTCCAGTAGTAAATCCATGGACAGAGGTTTATGCATCCAGAATCACACCATGGACCCAAGCAGTAGCTCCAACAGTAAATCCCCGGACAGAGACTGTCAGTTCCAGATTCATGCCATGGACCCAGGCTGTCCCTCCAGCAGTAAATCTGTGGACAGAAACTAATGCATCCAGAGTTATACCATGGACACAGGATATATCTCCATCAGTAAATCCCTGGACAGAGGCTGTTGGTTCCAGATTCATGGCATGGATTCCAACTGTACCTCTAGCAGTAAATCCCTGGACAGAGGCTGTTGGTTCCAGATTCATGCCATGGACCCAAGCTGTACCTCCAGCAGGAAATCCCTGGACAGAGGCTGTTGGTTCTAGATTCATGCAATGGACCCAAGATATACCTCCAGCAGTAAATTCCTGGGCAGAGGCTATTGGTTCCAGATTCATGACATGGACTCAACCTGTAATTCCAGCAGGAAATCCAGGGACAGAGGTTGTTGGTTCCAGATTCATGCCATGGACCCAAGCTGTACCTCCAGCAGGAAATCCCTGGACAGAGGCTGTTGGTTCTAGATTCATGCAATGGACCCAAGATATACCTCCAGCAGTAAATTCCTGGGCAGAGGCTATTGGTTCCAGATTCATGACATTGACTCAACCTGTAATTCCAGCAGTAAAACCAGGGACAGAGGTTGTTGGTTCCAGATTCATGACATGGATTCAATCTCTAATTACAGCAGTAAATCCCTGGACAGAGGCTGATACATCCAGATTCATGTCATGGACCCAAGCTGTACCTCCAGGAGTAAATCCCTGGGCAGAGGTAAATGCATCCAGAGTCCTGCCATGGACCCAAGCCGTCTTTCTGGAAGGAAATCCCTGGACAGAGGCTGATGCATTCAGAGTCCCAGCATGGATGCATGTAAATCCATGGACAGAGGCTGTCAGTTCAAGAGACATGCTATGGGACCAACCTGTACTTCCCGCAGTAAAACCACAGACAGAGGCTGTAACTTCCAGAGTCTCACCATGGACACAAGCTATAACTCCATCAGTAAATACCTGGACAGAGGCTATTGTATCCAGAGTCATAACAGGGAGTCAACCTATACTTCCAGCAGGAAATCCCTGGACAGAGGATGATGTATCCAGAGTCATGCCATGGACCAAAGCTTTACATCCAGCAGGAAATCACTGGACACAGGCTGTTGGTTCCAGATTCATGCCATTGACCCAATCTATACCTTTAGCATTAACTCCATGGACAGAGGCTGTCACTTTCAGAGTCTCACCATGGACCCAAATTGTACCTTTAGCAGTAAATCCCTGGACAGAGACCATAACTTCAACAGGCATGCCATGGACCCAACCTGTACCTCCAGCAGTAATTCCCTGGGCAGAAGCTGTACCTCCCAGAGTCCCGCCATGGACCAGAGCTGTATTTCCAGCAATAAATCCCTGGACAGAGGCTGTGGGTTCCAGATTCATATCATGGACTCAAACAGCATCTCCAATAGTAAATCCCTGGAAAGAGGCTGTTGGTTCCAGATTCATGCCATGGATCCAACCAGTAACTCCAGCAGTAACTCCCTGGACAGAGGCTGATGCATCCAGAGTCTTGCCACGGACCTTATCTGTACCTCTGGCTGTGAATCTCTTGACAAATGTTTCTGGTTCCAGATTCATGCCATGGACTCATCCTGGAACTCCAGCAGTAAATCTCTGGAAAGAGGCTTTCAGTTCCAGATTCATGCCATGGAACCAAGCTGTACCTCCAGTGGTAAATCCCTGGACAGAGTCTGTCACTTCCAGAATCACACCATGGACCAAACCTGTAGCTCCAGTAGTAAATCCTTGGTCAGAGGCTGTTGGTTCCAGATTCATGCCATGGACCCAAACTGTACCTCCAGCAGGAAATCCCTGGGTAGATGCTGGCACTTCCAGAGTCACTCCATGGACCCAAGCTGTACCAGCAACAGGATATCCTTGGACAGACACTGATGCATCCAGAATCACACCATTGAACCAAGCTGTAACTCTAGGACTAAATCCCTGGGCAGAGCCTATTGCGTCCAAAGTCATACCATGGACACAAGGTATATCTTCAGCAGTAAATCCGTGGGCAGAGGATATTCCTTCCAGAGTCATGCCATGGACCCAAGCTGTACTTCCAACAATAAATCCCTGGACAGAGGCTGATTCATCCAGAGGCATGCCATGGGCCCAATTGGTACCTCCAGCAGTAAATCCCTGGACAGAGGCTGATTCTTCCAGAGTCATATCATGGACCCAAGTTATACCTCCTGCAGGAAATCCCTGGACAGAGGCTGTTAGTTCCAGATTCATGCCATGGACTCAACCTATACCTCAAGCAGTAAATTCCTGGACAGAAGTTTATGCATCTAGGGTCCCACCATGGTCCCAACCTGTACCTCCAACAGTAAATCCTTGGACAGAGGGTGGTGCATCCAGAATCACTCCATGGAATCAAGGTGTAACTCTAGCAGTAAATCCCTGGACAGAGGCCATTGGTTCCAGATTCATGTCACTGACCCAACTTGTGCCTTCAGCAGTAAATGCCTGGACAGATACTGATATATCTAGATTCATGCCATGGAATCAACCTGTACCTACTTTAGTAAATCCCTGGATAGAAGCTGTTGGTTCCAGATTCGTGCCATGGACCCAACTGGTACTTCCAACAGTAAATCCCTGGTCAGAGGCTGATGTATCCAGATTCATGCCATGGATCCAACCTGTACATCCAACAGTAAAACCCTGGTCAGAGGCTGATGCATCCAGATTCATGCCATGGACCCAGCTTGTACCTCTAACAATAAATCCCTGGTCAGAGGCTGATGCATCCAGATTCACGCCAGGGACCCAACCTATACCTCCAGCAGTAAACCCCTGGACATATATGAATGTATCTAGAATCACTCCATGGTCCCTAGATGTGCCTTCATCAGTTAATCCCTGGACAGATGCTGTTGCTTCCAGATTCAATCCATGGACCCAAACTGTATCTCCAGCAGTTAATCCCTGGACAGAGGCTATTGGTTCCAGATTCATGCCATGGACCCAACTTGTACCTCTACCAGAAAATTACTGGTCAGAGACTGATGCATCCAGAGTCATGGCATGGACTCAACCTTTATTTCTAGCAGTAAATCTCTGGAGAGAGACTGATGCATCCAGATTCACACCAAGGACCCAACCTGTACCTCCAGCAGTAAATCCCTGGACAGATCCCGATAGATCTAGAGTTATGCAATGGACCCAACCTCTACTTCCTGTAGCAAATACATGGACAGAAGCTGATGCATCCAGAGTCATGCCATGGACCCAAACTTTACCTCTAGCAGTAAGTCCCTGGACAGAGACTATTGGTTCCAGATTCACACCATGGACCCAACCTTTACCTCCAGTAGTAAATCCTTGGACAGAGGCTGATGCATCTAGAGTCACACCATGGATTAAACCTTTACTTATAGCAGTAAATCCCTGGACAGATCCTGATGTATCCAGAGTCACACCATGGACTTATCCTTTACTTCTTGCAGTAAATCCTTGGAGAGAAACTGGTGCATCCAGATTCATGCCATGGACCCAACCTACACCTCTAACAATAAATCCCTGGTCAGAGGCTTATGCATCCAGATTCATGCAATGGACCCTACCTGCACCTCTAACAGTAAATCCCTGGTCAGAAATTGATGCATCCAGAGTTATGCCATGGACCCAACCTGTACCTCCAGCAGTAAATCCCTGGATAGTGACAGTTGTTTATACTTTAACACCATGGACCCTGGATGCCTCTCTACTAAATCCTTTGACAGAGACTAAGGCTTCCTCAGTGGGAATATGGACTCAGAACAAATATTCAGTAGTTAAAACTTGGACAAAATCTGAAGTTTCCACACTTACATCCTGGACAGATACTCAGACAGAATATCAAGCAGTAAATTCTTATATATCTTATATATCAAGTGTAACAGACACAGTCACATTTTGGACAATGCCAAAATATGAATCTAAGGAAACTTGGATACTGCCTGAAGCTCATATATTTAGTATATCATTGCATCCTCAAAGTGATACTCAATCCTTGTTCCAAGTAAAAAATATAGCATCTCTTCTGTCAATATATCCTGGAATTAATAGTGTCAATACATGGTCTTTGCCTAAATTTGAAACAGTGGCATCGTGGATAGTGCCTTTGTCTCAAGCAGCCAGATTTGTGCCCCTACCTAAAGCTGATATTAATAGAAATTGGTTTAAAATGGAAACAGAAACATTAAGAAATTGGACCCATTCAGAATCTCAAAGAGTAAGCACTTTGTCACAGTATGAAGCTAGTATGAAGCCCTTGGCCCAACATGAAAATTCTACAGTCATATCATGGATTCCAACTAAAACTGGTATATTCCAACCCTGGAATAAATCTGAAAGGGACAAAGTAAGAACCTGGACCCTTTCTGAAGGTAATGCTTTGCGACCATGGATTCAGATTGAAGCTAGCATTTTCAACCTCTGGACTCAGTCTAAAAGTAGTACAGTCACATCCTGGACACAGCCTGAGTTTCAGGCAGTCTTTACCAGGACTGAAGGACGTATAGATACATTTTGGTCCATGAATAAAAATGATGCAGTTAGGCCCTGGTCCCAGCTTGAATCTCAAATGACATCTTTCTGGACCCAAAGTAACATAACCAGCTCTTGGACTCAGTATAAAACTAGTACATTCATATCCAGGACCAAGCTTGAAATCAGTACACTGCAACCCTGGATTCAGTTTGAAACTGCTGCAATTAAATCATGGATTCAGTCTGAAAATGTAGAAATATACCCTTTGACCCAGCCAGAAGCTGATACAGTAATAAGACCTTGGTTGCAAATTCAAATAGATTCAATACAACCTTGGAATCAACCTGAAACTAATACAATTAGACCATGGACTCAACTTGAAACTGAAGTAATCCAAATTTGGACCCCCACAGAAAAGCAAGTAGTAAAACCTCCAACCTTACCTGAAATTGATACAATTACCTCTTGGTTACAGACTCAAAGTGATACAACTAGACCCTGGATTAAATCTGACTCCCAGTCTGTCAATTCCTGGGGTCATGCTGACATAGGTATAGTTCACGATTGGATTCAGCAAAGAGGTACTGCAAATCAACCCTGGACCTACCCTGAACCTACCCAAACAGTCAGACCCTGGGTGAAGCTAGAAGCTGATACACTTATATCTTGGTTGCACATTCAAATAAATAAAGTCAGACCATGGACCAACTCAGAAGATCAGATATTTAGCTTCAGGTTGCAGCCTGAAGTTGGTATGGTTCACCCTTGGATCAATCCTGAAACCAAAACAGTCAGATCCTGGGCCCACTCTGAAACTGATTTTATTGCATCCTTTGCTATAGATGAGCCTAACGAAGTCAGAACATGGATCCATACTGAAGTAGAGATAACATCTGACTGGCATGATAGCATTATATCATTTGTTCCTTCTGAAATTGAGCCAGATGGAGCAACTTTCTTAACTAGTGATTTCAATTCCTGGTCTAAACATATACCTTTTTTACCAATAGAATTAATTCTTTCCCCAGATCATTATTTTATAGGTTTCTCAACTGAGATAGCAACAACAGAAAGCCAAAATGAAATTAATTCTTTCCAATTGAGTGAGCTCACAAACATTTCCTTTCTTAAACTTCTGAGTACATGGCTTCCTGAAAGACTTGATTACCCGAACTTTGGCAATAAATTACAAGTTATCAAAACAAATGGAAGCCCTGATGTACCATCTACTTCTCTTTACCCCATCTCTCCATCATTTTCCTTTCTTGTTCCTTGCTCTCTCTCATCCCCATGTGCATTGTCCCACTCATGTTTGGTCATTTCTTCTTGCATATTCCCTTTATATTGTACTTTCCCTTCTTGCTCCATTCTTTCTCCAGGAGTCTTCTCTTCTATTCTCTTGCCCTTAGACTCTTCTTCTGACAGCTCTGACAAGGAACTGTCTTCCCTAAAATTTACTGAAGAGACCATAATTTCTCATACTTTTTCATATCTGCTTGCTGCTC

>OAR Sheep

CAGGATGACAGTCCAGGTGTAATTACCTGGGGAGAGACTGTTCCTTTCAGAACCACATCAAGGACACCGATTGAAAGTCCAGATATAAACACCTGGAGAGAGACTGTGCCTTTCACAGCCTCACAATGGACTCAGGCTGAACCTCCAGCTATAAATTCTTGGAGAGAGATGATACCTTTTTCCATCCCACCATGGACACAGGATGGAAGTCCAGGTGTAATTACCTGGGGAGAGACTGTCCCTTTCAGAGCCCCACCAAGGACGCAAATTGAAAGTCCAGATGTAAACACCTGGAGAGAGATTGTGCCTTTCACAGCACTACCATGGACACAGGCTGAAGGTCCAGATGTAAATACCCAGAGAGATACTGTGCCTTTTACAGGTCGGACTCAGGCTGAAAGTACAGCTGTGCATACTGGGAGAGATACTGTGCCTTTCACAGCTCCACCATGGACTCAAGATAAAGGCCCAGATGTAAATAACAGCAGTGAGGTGCTGAGTTTCACAGGACCATCATTGGCACATGCTAAAGGTCCAGCTTTAAATAACTGGAGGGAGACTGTACCTATAATAGGCTCATCCTGGACTCAGGCTGAAAATCCAACTGTAAATGCCTGGAGAGAGAATATGCCTTTAACAGCCCCACCCTGGTCACAGGTTGAACATCCAGCTATAAATACATGGAGAGAAACTGTGCCTTTCACAGAGCCACCATGGACTCAGGCTGAATATCCTGCTGTAAACACCTGGAGAGAAACTGTGCCTTTCTCAGCTCTACCATGGACTCAGGCTGAAAGTCCAGCTGTAAACACCTGGAGAGAGGCTATGCCTTTCACAGACCCATCATGGATTCAAGAGAAAAGTCTAACTGTAAATAGCTGGAGACAGATTTTTACTTTCCCAGCCCAACCATGGCCACAGACTGAAAGTACAGTGGAAAACGACTGGATATGGAATGCCCCTTTTACAGCTCCACCATGGTCACACACTGAAAGTTCAGCTGTAAATACCTGGACTGAGCCTATGCTTTTTATAGCCCCTCCATGGACTGAGGCTGAAAATCCAGCTACAAATACCTGGAAAGTGAATATGCATTACAGAGACACACTATGGCCTCAGTCTGACTTTGCACCAGCAAACCCTTGGACATCAACTGAAAGTTTCAGAATCACCTCATGGACTCATACTGTAAAGCAAGTTTTAAATATTTGGACAGAGCCAATAGCTTCCACAGCCACACTGTGGACTCAGGCTGAATATTCAACACCAACATATTGGACAGAGATTAAGGCCATTTATATAGTCACACCATTGACCCAGTATCAGTTTTCAATAAATACTTTGACAAAATCTGTAGGAGCCATAATCACACTTTGGACGTCTGCTGAATCTCTGTCATTAAGTTCTTTCACACAGAATAGTATCGATACAATCGAATTTTGGCCAATGCTTAAAACTGAGTCTAAGAAAAGGTGGAATCTGCCTCAAACTAGTACATTCATGTTTTCACTAAATCCTCAAATTGATACTTTTGGATCCTTGAACCAAATTGAAAATCAAGAATCTCCTCGGTGGGCCCATCCTGAGATTGATAATGCCAATACAATGGCCTTTCTTGAATTTGGAACACTCATATCACAGGTAGTACCTTTGCCCCAAGCAGCTAGACTCTGGCCCCAAACTGAAGCTGCTAATAGCAAAATTTGGTTTGTATCCTCTGAAAGAATAAATTCCTGGGACCAATCAGAGTCTCAAAGAATGAGTACCTCAATCCATTTTGGAGTGGGTAGAGTGAAGCCCCTGGCCCAACATGAAACTGCTATAGTCATGTCATGGCTTCAGATTGAAACTGGTATATTCCACCCTTGGAACAAGTCTGAAGGAGGCACAGGGAGGTTCTGGCCCCTTTCTGAAACTGAGGATATAAGAGAATGGATCCAAACTGGAGCCAGTACAGTTAACTCTTGGACTCAACTGAGAACTAATATAGTCAGAGCTTGTCCCCAAGCTGAATCTGAATTAGTCAGACCCTGGACACAAGCTAAAACTAATGCAATCACACTATTGACCCAGACTGATGCTATCAAACCTTGGTTCCAAACTAAAATTAATTCACTAAGAGAAGGGACCCAAACTCAATCTCAAATTGTTACTACTTGGATCCAAACACAGTTGCAAATATTTCACCCCTGGATTCAGCCTAAAAGTGATTCAGTCAGATTTTGGACCCAGCCTTGGATCCAAGCTGAAACCCACACAGTCAGACTCTATTATGAAATTGATGTAAGAAAATCATGGGCTTCATCTGAATCTCAGTCAGTCACATTTTGGTCACTGAGTCAAAATTCAGTTAGGACCTCATTTCACTTTGAATCTCAGATGACATGTTCCTGGGTCCGAAATGAATTTGATATAATCAGTCCTTGGAATCAATATGAAACTAGTTCTGTTGGATCCTGGATCCAGTCTGAAACTGGTACATGTCAACCCTGGTTCCATATTGAATCTTCTACAATCACACCATGGACCCAATATGAAACTTTAGAGATCTCCCCTTCAACCCATCCTGAGACTGATACAGCAATAAGGCATTTGTTCCAGCCCCAAATTGATCCAATTAGTACTTGGAATCAGCCTGAGGTAGATACAATCAGATTCTGGACCCAAGTTGAAACAGAAACAATTCCAATTTGGATCCAGATTGGAAGTCAAGTAGTTAAACCTCCCAACTTTTCTGAAGTTGGTATAGTTACACCTTGGCTAAAGACTGAAACTGATGCAATTATTCCCTGGATTCAGTCTGACTTTCAGTCAATCCATCCTTCGACCCAGACTGGATTTGGTATAATTGACCCCTGGTTTCAGCCAAGAGCTTCTGTAAATCAACCCTGGACCTTTGTTCAAACACAGTCAATCAGACCTTGGATTAAAGTGGAATCCAATACAATCAAATCTTGGTTTCATGTTCCAATGAAAAAAGTCAGACTGGGGATTCCTTCTGAGTCTCAAATATTGAGTTTCTGGTTGCAGTCTGATGTTAGTAGAGTTAATGCTTGGATCCAACCAGAAACCCAGGCAGTCAATCCTGGGGCTCATCCTAAAACTGGTAATGTTGCATCCCTGACTATTCCTAAGCCTGAAAGAGTCAGAATGTGGATCCAGCCTGAAAGAGAAATGAGACCTGGCATCATTTATAAAACTAATATAACCACATCATTTGCTTCTGAAATTGAACCAGATGGAACAATTAGTCATTTTGATTCCTGGGCTAACCATGTAACATTTTTACCAATAGAAACTGTTCCTTCCCTAGATGAGCATTTTGCAGCTTTGTCAACTGAAATAGCTGCAGTAGAAAGCCAAGGTCAAATAAATCCTGTCCAACCCAGTGAAATCACAAATATTATCTTTCTTACAGTTTCAAGCACACAGCTTCCTGGAGGAGCTGGTTACCTGAACTTTGGCAACAAATTACAAATTACCAATTCAAAAGGAAGTCCTAATGTCCCATCTAGTTCTCTCAACCCACTTTTTCCATCTTTTTCCTTTATTGTTCCTTGTTTTTTCCCATTTTCATGTTCTTTGTCCCTTACTTGTTCAGTCTTTTCTTCTTGCACATTTTCTTCACCATGTACTTTTCCTTCTTGCTCAGTTCTTCCTATTGTGGGTCTCTCTCCTGTTCCTCCCTTAGCTGCTTCTGATAGTTCTCTCCAGAAACCATCTTCCTCGAAAGTTATTGAAGACACCATTCTTTCCCATACTTTTTCATCCTTTCATGCTGCT

>CHI Goat

ATTGAAAGTCCAGATATAAACACCTGGAGAGAGACTGTGCCTTTCATAGCACTACCATGGACACAGGCTGAAGGTCCAGATGTAAATACCCAGAGAGATACTTTGCCTTTTACAGGTCTACGTTGGACTCAGGCTGAAAGTACAGCTGTGCATACTGGGAGAGATATTGTGCCTTTCACAGCCCCATCATGGACACAGGCTGAACCTCCAGCTGTAAATTCCTGGAGAGAGATTATGCCTTTTTCAGTCCCATCATGGACACAGGATGGAAATCCAGGTGTAATTACCTGGGGAGAAACTGTCCCTTTCAGAATCCCACCAAAGTCACAGATTGAAAGTCCAGATGTAAACACCTGGAGAGAGACTATGCCTTTCATAGCACTACCATGGACACAGGCTGAAGGTCCAGATGTAAATACCCAGAGAGATACTGTGCCTTTTGCAGGTCTACACTGGACTCAGGCTGAAAGTACAGCTGTGCATACTGGGAGAGATATTGTGCCTTTCACAGCCCCATCATGGACTCAAGATAAAGGCCCAGATGTAAATAACTGGAGTGAGATGCTGAGTTTCACAGGACCATCATGGGTACATGCTAAAGGTCCAGCTTTAAATACCTGGACAGAGACTGTCTGGAGGGAGACTGTACCTTTAACAGTCTCACCTTGGACTCAGACTGAAAATCCAACTGTAAATACCTGGAGAGAGAATATGCTTTTAACAGCCCTACCCTGGTCACAGGTTGAATATCCAGCTGTAAATACATGGAGAGAAACCGTACATTTCACAGAGCCACCATGGAATCAGGCTGAATATCCTGCTGTAAACACCTGGAGAGAAACTGTGCCTTTCTCAGCTCTACAATGGACTCAGGCTGAAAGTCCAGCTGTAAACACCTGGAGAGAGGCTATGCCTTTCACAGACCCATCATGGATTCAAGATAAAAGTCCAACTGTAAATAGCTGGAGACGGATTTTTACTTTCCCAGCCCAACCATGGCCACAGACTGAAAGTACAGTGGAAAACGACTTGATATGGAATGCCCCTTTTACAGCTCCACCGTGGTCACACACTGAAAGTCCAGCTGTAAATACCTGGACAGAGCCTATGCTTTTTATCGCCCCTCCATGGACTCAGGCTGAAAATCCATCTACAAATACCTGGAAAGTGAATATGCATTACAGAGACCCACTATGGCCTCAGTCTGACTTTGCACCAGCAAACCCTTGGACATCAACTGAAAGTTTTAGAATCACATCATGGACTCTTACAGGAAAGCAAGTTTTAAATATTTGGACAGAGCCAATAGCTTCCACAGCCACACTGTGGACTCAGGCTGAATATTCAACACCAAAATATTGGACAGAGACTAAGGCCATTTATATAGTCACATCATTGACCCAGTGTCAGTTTCCAATAAATACTTTGACAGAATCTGTAGGAGCCATAATCACACTTTGTACATCTGCTGAATCTCTATCATTAAGTTCTTTCACACAGAATATTATTGATACAATTGAATTTTGGCCAATGGTTAAAACTGAGTCTAAGAAAAGGTGGAATCTGCCTCAAACTAGTACATTCATGTTTTCACTAAATCCTCAAACTGATACTTTTGGATCCTTGAACCAAATTGAAAATCAAGAATCTCCTCTGTGGACCCATCCTGAAATTGATAATGTCAATACAATGGCGTTTCTTGAATTTGGAACACTCATATCACAGGTAGTACCTTTGCCCCAAGCAGCTAGATTCTGGCCCCAAACTGAAGCTGATACTAGCAAAATTTGGTTTGTCTCCTCTGAAAGAATAAATTCCTGGGACCAATCAGAGTCTCAAAGAATGAGTACCTCAATCCATTTTGGAGTGGGTAGAGTGAAGCCCCTCGCCCAACATGAAACTGCTACAGTCATGTCATGGCTTCAGATTGAAACTGGTATATTCCACCCTTGGAACAAGTCTGAAGGAGGCACAGGGAGGTTCTGGCCCCTTTCTGAAACTGAGGATGTAAGAGAATGGATCCAAACTGGAGCCGGTACAGTTAACTCTTGGACTCAACTGAGAACTAATATAGTCAGAGCTTGGCCCCAAGCTGAATCTGAACTAGTCAGACCCTGGACACAAACTAAAACGAATGCAATCACACTATTGACCCAGACTGATGCTATCAAACCTTGGTTCCAAACTAAAATTAATGCACTAAGAGAAGGGACCCAAACTCAATCTCAAATTGTTACTACTTGGATCCAAACACAGTTGCAAATATTTCACCCCTGGATTCAGCCTAAAAGTGATTCAGTCAGATTTTGGACCCAGCCTTGGATCCAAGCTGAAACCCACACAGTCAGACTCTATTATGAAATTACTATAAGAAAATCATGGGCCTCATCTGAATCTCAGTCAGTCACATTTTCATCACTGAGTCAAAATTCAGTTAAGAACTCATTTCACTTTGAATCTCAGATGACATGTTCCTGGGTCCGAAATGAATTTGATATAATCAGTCCTTGGAATCAATATGAAACTAGTTCTGTTGGATCCTGGTTCCAGTCTGAAACTGGTACCTGTCAACCCTGGCTCCATATTGAATCTTCTACAATCACACCATGGACCCAATATGAAACATTAGAGATCTCCCCTTCAACCAATCCTGAGACTGATACAGCAATAAGGAATTTGTTCCAGCCCCAAATTGATCTAATTAGTACTTGGAATCAGCCTGAAGTAGACACAATCAGATTCTGGACCCAAGTTGAAACAGAAACAATTCCAATGTGGACCCAGATTGGAACTCAAGTAGTTAAACCTCTCAACTTTTCTGAAGTTGGTATAGTTACACCTTGGCTAAAGACTGAAACTGATGCAAGTAGACCCTGGATTCAGTCTGACTTTCAGTCAATCCATCCGTGGAGCCAGATTGGATTTGGTATAATTGCCCCCTGGTCTCAGCCAGGAGCTTCTGTAAATCAACCCTGGACCTTTGTTCAAACACAGTCAATCAGACCTTGGATTACAGTGGAATCCAATAGAATCAAATATTGGTTTCATGTTCCAATGAAAAAAGTCAGACTGAGGATTCCTTCTGAGTCTCAAATATTGAGTTTCTGGATGCAGTCTGATGTTAGTAGAGTTAATGCTTGGATCCAACCAGAAACCCAGGCAGTCAATCCTGGGGCTCATCCTAAAACTGGCAATGTTGCATCCCTGACTATTCCTAACCCTGAAAGAGTCAGAATGTGGATCCAGCCTGAAACAGAAATAAGACTTGGCATCATTTATAAAACTAATATAGCCACATCATTTGCTTCTGAAATTGAACCAGATGGAACAATTAGTTATTTTGATTCGTGGTCTATCCATGTAACATTTTTACCAATAGAAACTGTTACTTCCCTAGATGAGCATTTTGCAGCTTTGTCAACTGAAATAGCTGCAGTAGAAAGCCAAGGTCAAATAAATTCTGTCCAACCCAGTGAGATCACAAATATTCTCTTTCTTACAATTGCAAGCACACAGCTTCCTGGAGGATTTGGTTACCTGAACTTTGGCAACAAATTACAAATTACCAATTCAAAAGGAAGCCCTAATGTCCCATATAGTTCTCTCAACCCACTTTTTCCGTCTTTTTCCTTTCCTGTTCCTTGTTTTTTCCCATTTTCATGTTCTTTGTCCCTTACTTGTTCAGTCTTTTCTTCTTGCACATTTTCTTCACCATGTACTTTTCCTTCTTGCTCAGTTCTTCCTATTGTGGGTTTCTCTCCTGTTCCTCCCTTAGCTGCTTCTGATAGTTCTCTCCAGAAACCATCTTCCTCAAAAGTTATTGAAGACACCATTCTTTCCCATACTTTTTCATCCTTTCATGCTGCT

>OVI Deer

ATGAAAGGTCCACTTTGCCTCTTTCAGTTTTGGGGGTTGTTTTTTCACTGCTATGGTGTACCCAATTCTCAAAAGCAGTGGTTAAACTCATTGACCCCAACGATTGCCCCATTGTCAGCATCCAACAATATCCCATGGCTAGTGTCCATGGCTGAGACCTGCCAGGGCATTATTCTGAGTCGGTGGTGGATCCTCTCCACAGCCAGCTGTCTGAGTAAACTGAAACAGTTAAACTCTGACATTTCAGGAGTCTTTGACCAAGAAGATGTCTTACAAGGCCACAAAGTATGCCTACTCCCTAGTTTTGATCCAGAAACTGGAAAAGATCCAGTCAAAGCAGATATAGGGATAGTACTCCTTCAATATCCTATCAGGAAGAAAGAAATACCACTTTCTCACACTTATAACATCTTCTGGAAGACCTGTTATAACTGCCAATACAGAGACTGCAGGGTGTACCAATATCAGAATCATGGTAACTTTGAAAGCAATATCAAGAAATTGTCAGTTAAGCTGCTGGACCTCTCATTTTGCCACCATCAACACATTCACCTGACTAAAAGTAACAATTTATGCATCTGGAGTCAGTCAGAAGAAGACTGCTGGGTACAGCAGGGTAGTCCTGTTTTCTGTCTCTTTGGCAACCACTGGGAACTGGTAGGCCTGGTCAGTGAGTCCTCAATGGCCTGTTATGACCCAATTCTTGTCATCAAGACAGCCCCCTACCTACCTTGGATGAGACAGCTTATCAAGGCATCCCAGAAGTCACTGGATCCTATTTTTTCTCTACCCTGCAGTTTTTCTTCTAGGGTAGAACATGATAATCAATATAGGCTTATCCCAAAAAATGCTTCTGCATTTCTGTCCTCCCATGGATTCTCTATACAGTTATGGAGGGGAAAGTTTGGCAGTTCCCCACTGAACAGACAGCGCCGGAATCCTCCTCCAGTATTTTTTGGTCCAAATGACAGAGATTCTTTTCCTGGAAGTTCACAGTTATACCTTCAAAGCAGTCAGATCTCCTCAACTAGCAATTCCCTAATGGTAAAATCTTGGACCTCTCCTCTTGTTAAATCATGGGATCCTTTTCATACAGCTGTGTCCTGGAATACTGCAGGAACTGATATTTCTGATCCTTCAGTTCTTTATCAACCTCAAAGCACTCCAACACCAGATATAAGTACACCCTGGAACCTTCCTCAGAGAGATGTAGTGAAATATAAATATAGAAATATGACAGATTCATCAAGATACTGGGTTGAATATTTAGGTGGTATAATTGGGCTTTATTCTCCACCATTAAATAATGCTGATGGATCACATGTTCTGTTTTCAAGTGATGTAGAAGGATCCCAATTTCATTCTGAGATAAAAAATGCTCAGTCTCAAGTTCAATATGGCAGGGTTCCTTTCCAAGGTCAATTTCTAGGGAGACCCTGGCTGCGTATTGCATCTGGTGTTAGACCTTGGACATATAATATGGCTGATAAAACAGAAATAGTGATACAGATTCAACCTGCTGAGGGGAGTTTTAGAACTCAAATACATCATGTTGCTGATAGAGTCAATACTGCTATTCAATCTGTAACTTATAACATGAATCCATGGATACCTTTGATTATTAATAAGATTGGATTCTGGACTCATTCCATACTAAATGAAGAGGGATCTCAATATCCTACAGCAACTCCTACCTCAGAACCCTGGTTTCAGCCTGTCCTAAACATAGTTAAATCCCAAGGACCTATAGAAAAGACATATGAACAAATGATTTTCCCTGAACCTAAGCCAACTCAATTTTGGACTTCCTCAACTCTTAATATGCTCCTTAATTGGGCTTCATCTGCAAGTAATACTAATAGGCCTGTGGCCCAGTATAAGGCCAGTGCAATCACATCCTTAAGTCAAATGGATAGAATGAATCCAGTGAGTAAGCACAGTGATTTTACAGTCAAAACCCAGATGCAGAAGGAAGATACAACATGGCTTTTGATTCATACAGTTGCTAGTATAATTGATCCTTTAACCCACACTGAAGTTGATACAACTGCACCACGGACTCAACCTGAAGTTGACATAGTCCAAACTTGGACCCAGTCAGAAACTCAATCAGGAAGCCCTTTGACTGTGCTGAAAGCTGATACCACCAAACTGTGGTTACAGACTAAGGGAAGAAGAAGACACCAGATTCAGCCTAAATTCCAAATTCTCAGACCTAGAACTCAGACTGAAGAAGGTGAATATAAACCTTGGATCCAGTCAGAAGCAGATGCTGTCAGATCCTTGACCCACTCAGAAATGGAAACACTCAGACCCTGGGACCAACCTGAAACTGGTACTGCCAGATCTTTGTTCTTGACAAAATCTGACCAAATAATACTGAACACTCAGGCAGCCTTTAAAACACCCCACATATGGACTCAGCCTGAAGTTGACACAGTCAGGCCACAGATTCAGTCTGAAGCTGGCAGAATGCAACAATGGACAAAGCCAGAAAAATCTCCTTTAATACTCACAACAGAGTCTAAAGTTAATACAATTACACCATGGACAAGGCCTGAAGTTGATGCTATGGGGCTTTGGTTGCAAACACAAACTGATATAATCAGGACCTGGACCCAAACTGAAACTCAAACAATAATTTCTTGGTTTGAACCAGAAGCTATTATAGTCAGACCTCTGTTGAATACTCAAACTGATACATTTAAACCTTGGATTGAGACAGAAATAGAAACACCCCATTACTGGGCCCAGTCTGAAGGTAATATAGACAGATCATGGACAGAGATTGAAGCTGAAAATGTCAAACCCTGGTTCTGGACTCGAATGGAAATAGCCACTTTTTGGACAGAGCCAGTAATCAAAACATCCCACCGCTGGATGCAGTCCAAAAAGGAAATACTCAAGCCCTGGGACCAATTTTATGAGGTAGAAACCTGGACACAACATGACACTAAAACACTCAAGTCCTGGAATGAGATTGAAAGTGATAAAGATAGATTTTGGACTCAATCAGAAGTTGACACATTGAGACCCTGGATTCAGTCAGATATTGCTATCAACAACCATGGAGCGGAAAATGAAGCTGGTACATTAATGCTTTGGCCTCAGGCTGAATATCCAGAAGTAAATCTCTGGACACAATCTGAAACTGATGCAGTCTCATTATCAACCCAGAGTAAAATTCCAGCAGTAAATCACTGGACAGAAATAGCTGATACCATCATACCACAAGCAAAAGCTAAATTTCCAGGAGTAAAGCCCTTGACACATCTGGCATCTAATACAGTAACACCATGGACTCAGGCTGAATCTATAGTAGTAAATCTCTGGACTCAGCCTATAACTGATGCAGTCACACAGTGGACTCCTGGTGAGTTTTCAGAGACAAATCCCTGGACAAAAACTACAATTGATAGAGTCAAGTCATGGACCCAGGATGAATCTCTACTGGCAAATCCCTGGATGCAATTTAACACTAATACAGTTATACCACGGACACAGACTGACTCATTCATAGTAAATTCTTGGTTACAGTCTATACCTCATACCATTATACAATGGACCCACAGTGAACCTTTAGATATAGCTCCCTGGAAAAAAGCTGTCACTGATGCAACACCTTGGTCTCAGGCAGAGTCATCAGCAGTAAATCCCTGGAGAGAAAGTGCAACTGGGACAGCTACACAGTGGACCCAAGGAGAATCTCCAGTAGTAAATTTATGGGCTCATCCTGTGGTTAATGTAGTCACACTGTACACTCAGGCTGATTCCCCATTGTTACATTTCTGGACAAAGTCTGAAATAAATATAGTCACAACATGGACCCCAACAGAATCTTCAGCCATAAATTCCTGGAGACAGTCTGAAACTGACAGTATCTCAACATGGACCAATAAAACAAATCCAGAAATAAACAGTTGGTTATATTATGAAACTGATACAATGACAGTTTGGACCCAGCCAGAAAGTCCAGGAATAAATACTTGGACACCAGCTATTGCTTATGCAGCTCCACCATGGACTCAGGCTGATTTTTTAGCTGCAAATCCCTGGACAGAGGTTGAAAGTGATACAATCACACCATGGACTCAGGTTGAACCTTTAATGAATCCTCGGTCAGAGAGTGTAGCTTCCACAGTCATACCATGGACCAAGGCTGAATCTCTAGCAGTAAATTCATGGATACAATTTGCAGCTGATGTAGATACACTATGGACCCAGACTGATTCTCTAGCAGTAAATCCCTGGATATATCCTGCATCTGATGATGTTATACAGTGGATTCAAACTGAACTTCCAACAGAATATCAATGGATTCAGAATATGTCTGATATAGTAACATCAATTACTCAGGCTGAATTTCCTTCAGTAGAGACTTGGACAGATCCTCTGGCAGATATAGTAACAAGGTGGAATCAGACTGAATCTCCAATAATAAATCCATGGTCAGAAGCTGTAGTTTCTACAATGACACCATGGACTCAAGTTGAATATCCAGAACTAAATACCTGGATAAATTCTATAGCTTCCACAGTCACACAGATGACTCAGCCTAAATACCCAGTAATGAAGCCTCAGAGACAGCCCATATCTGGTACAGCCACACCATTGAACCAAACAGAATCACCTATAGTAAGTCCCAGGACACAACATGAAACTGATGTAATCACACAGAGGACTCAGACTGAATCTGAAAGAATTAATCCTTGGATACAAGTTGTAACAGACACAATCATACGCTGGACTCAGGCTGAATCTCCAGCAGTCAATTCCTGGACAGAGGCTGTTGCAGATACAGTAATACCATTGAATCAGACTGAATATCCTTCAGTAAATCCCTGGGCAATGCTTGCAACTGATACTTTTACACTTTGTACCCAGGCAAATTCTTCCATCATCAATTTTTTGACACAGTTCATAGTTCATACACTCACACCCTCCACCCAGACTATGCCTGTAGCAGTAAATCCCTTGTCACAGTTTGAAACAGATAGAGTCACACAATGGATCCAAGCTCAATTTGCTTCAGTAAACCCCTGGACTCCATCTGAAATCAACCCAATTATAGCATGGACCTATGCTAGATATTCAACCTTAAACCCCTGGACACAATTTGAAGCTGACATATTCACATTATGGACCCAGGCTGAGTCTCCAGCAATAAATATAGCTTTATCTAGGTCAGTGAAGCCATGGATCCAGGTTGAATCTCCTGCAACAGGTCCCTTAACAACAGCCATAGCTGGTGAAATCACACCATGGATCAAGGCTATATCTCCTGCAGTTAATCCCTGGACAAAATCTAAATATGACATAGTTCCACTGTTGATCCAGGATATAACTCCAGCAGTAATTCCCTCAACAGTCTCTGTAATTGATACAATCACCCTGTGGGACATGGCTAAAATTATATTACCAAGGCCTTGGCCACAGCCTGTGACAGAAACAGTCACACACTGGATCCAAAGTGAGTTTTCCTTGGTATATAACTTCACACGACCTATAACTGATGTAACCACATTGTTTACTCATGTTTTTACTCCAGAAGTAAATCCCTGGTCAAGGCCTAAAGCTGATACTGTCAAACAATGGATCGAGGTTAAATCTCCAAAGGAAAAGGCCTGGAAAGTGGCAGGTTCTGAAACACTCACACCATTGACCACAGGATTCTCTACAACAGTTAAAGTCTGGATAGACACTGTGTCTGATATATTCACACCAGGAACCAAGTACACATTTTCAAAATTAAACCTTGGAATAATGCCCGAGGATGGTATCATTGAACCATGGACTCAGTCTGAATCTCTATCAGGAATTCCCTGGACACAACCCGCAGCTGATTCAGATACAAAGCAGATACAGACTAAATACCCATTAGTAAATCTGTGGACTCAGGCTCAGTTCCCAGCAGTAAATCCCTGGACACAGTCCCAGTCACTACCGATAAATATTTTTACAAAATCCAAAACTCTAGGCATAAATCTATCAATACAACCTGAAACTACTACAGTCACGTTATGGAGTCAGGTTGACTCTCCACCAGTAAATCACTGGATAAAACCTGTATCTTCCACAGTCATACCATGGACACAGGCTGAATATTCAGCAGTACATCCATTGATGTGGTCAGCAGCGAATGCAGTCACATGGTGGACCCAAAGTGAATCTCCTGCATTGTATTCCTGGACAAAGTCTACAGTAGATACAGCCACAAAGTGGGCTATGGGTGCATCTCCAGTGTCAAATTACCAGACACAACGATTAGCTTACACAATCCCATTTGGGGTCAAATATGGAACATCAAGGATAAATCCTTGGATGCATTCTAATTTTGAGCCATTTCCATCAACACAAGTAGAATCTTCAGCAAATAAACATGGGATACATATAGAAAATTATAGAGTTATAACAATGCCCCAAATTGAACCAAGTAAAGTGAATCTCAGGCCAGAGCCTATACATATAGGAAGAAAATGGACTAAATCTCAGTTTTCCATAATTCGTTTGACAGGGACTGTGTCTTTCACAACCCGATCATGGACAAAGGCTGAAAGTTCAACTGCAAATACATGGAGAGAGACTGAGTCTTTAACATCACCAACATTGACTCAAGCTATAAAGCTATCCTTTAAAGAGGATTTTATAGGCCTTTTTATAGGCCCAACATGGACACAGGCTGAAAATACAGATGTAAATACCTCAAGAAAAGCTGTTTCTTTTACAGCCCCACCATGGACACAGGCTGAAAGTCCAGATGTAAATATCTGGAGAGAGACTGTATCTTTCACAGATCCCCCATGGACTCAAGTTAAAGGTCCAGCTATAAACAACTATAGAGAGATGTTGACTTTCACAGCCCCACTGTGGACACAGGATGAGGATCCAGCTGTAAATACCTGGAGGGTAACTGTGCTTTTAACAGGCCCACCTTGGACTCAGGCTGAAAGTACAGCTGTAAATGCTTTAAGAGAGACTGTGTCTTTCATAGTCCCACCGTGGATACAGGCTGAAGGTCCAACTTTAAATAACTGGAGGGAGACTGTGCCTTTCACAGGTCCACCTTGGACTCAGGCTGAAAGTACAGCTATGCATATTTGGAGAGAGACTGTGCCTTTCACAGCCCCACCATGGACTTCAGACAAAGGTTCAGCTATAAACAACTGGAGAGAGATGTTGCCTTTCACAGTCCCACTGTGGACACAGGCTGAGGGTCCAGCTGTAAATACCTGGAGTGAGACTGTCCCTTTTACAGTCCCAACATGGACTCTGGCTGAAAGGCCAGCTGTAAATTCCTGGAGAGAGAGTGTGCCTTTTACAGTCCCACCATGGACTCAGGATGAAAGTCCAGGTGTAATTACCTGGGGAGAGACTGTCCCATTCAATGCCCCACCAAGGACACAGTTTGAAAGCCCAGATGTAAACACCTGGAGAGAGACTGTGCTTTTCACAGCCCCTTCATGGACTTGGACAGAACGTCCAGCTGTAAATTCCAGGAGAGAGACTGTGCCTTCTACAGTCCTATCTTGGACTCAGGATGAAAGTCCAGATGTAAACACCTGGAGAGAGACTGCCCCTTTCACAGTCCCAACATGGACTCTGGCTGAAAGACCAGTTGTAAAATCCTGGAGAGAGAATGTACCATTCACAACCCCACCATGGACACAGAGTAAAGGTCCAAATGTAAATACCTGGAGAGAAACTCTGCCTTTCACAGGTCCACCCTGGACTCATGCTGAAAGTGCAGCTATAAATAGTTGGAGAGAGACTGTGCCTTTCCCAGTCCCACTATGGACTCTAGATAAAGGCCCAGCTGTAAATAACTGGAAAGATGTGTTGACTGCCACAGCGCCACCATGTAAACAGTCTGAAGGTCCAGTTGTAAATTCCTGGAGAGAAACTGTGCCTTTTACAGTCCCACAACGGACTCAGGATGAAAGTCCAGGTGCAATTACCTGGGGAGAGACTGTAACACTCAGAGCCCCACCAAGAACAGACATTGAAAGTCCAGATGTAAACACCTGGAGAGAGACTGTGCCTTTCCCAGCCCTACCATGGACACAGGCTGAAGGTCCAGCTGTAAATTCCTGGAGAAAGATTATGCCTTTTATAGTCCCACTGTGCACACAGGATGAAAGTCCAGGTTTAATTACCTGGGGAGAGACTGTCCCTTTGAGAGCCCCACCAAGGACACAGGCAGAAGGTCCAGATGTAAATACACAGAGAGTTACTGTGCCTTTTACAGGTCATCCTTGGGCTCAGGCTGAAAGTGCAGCTGTGCATACTGGGAGAGATACTGTGCCTTTCACAGCCTCACCATGGACTCAAGATGAAGACCCAGATGTAAATAGCTTCAGTGAGATGTTGAGATTCTCAGCACCATCATGGGCCCAGGCTAAAGGTTCAGCTTTAAATAATTGGAGGGAGATTTTACCTTTAATAAGCTTACCTTGGACTCAGGATGAAAATCCAGCTGTAAATACATGGAGAGAGACTGTGCCATTCATAGAGCCACCATGGACTCAGGCTGAACGTTCTGTTGCAAATAGCTGGGGAGAGACTGTGCCTTTCTCAGCTCAGCTATATATTCAGTCTGAAAGTCTAGCTGTTAAGAACTGGAGAGAGATGTTTCCTTTCACAGCCCCACCATGGACTCAGGATAAAAGTCCAGCTGTAAACACCTGGAGAGAGATGTTCCCTTTCACAGCTGCACCATGGACTCAGGATGAAAGTCCAGCCATAAATACCTGGAAAGAAACTGTGCCTTTCTCAGATTTACCATGGACTCAGGCTGAAAGGCCAGCTGTAAACACCTGGAGAGATACTGTGCCTTTCACATCCCCACCATGGACTCAGGCAGAAAGTCCAGCTGTAAACACCTGGAGAGAGATGTCTCCTTTCACAGCTGCACCATGGACTCAGGATGAAAGTCCAGCTGTAAACACCTGGAGAGAAACTGTGCCTTTCTCAGCTCTACCATGGACTCAGGATGAAAGTCCACCTGTAAACACCTGGAGAGAAACTGTGCTTTTCTCAGCTCTACCATGGACTCAGGCTGAAAGTCCAGTTGTAAATGCATGGACATGGAATGCCCCTTTTACAGCTCCACCATGGTCACACACTGAAAGTCCAGCTGTAAATACCTGGACAGAGGCCTGGATTTTCACAGCCCCTCCATGGATTCAGGCTGAAAATCCAGCTACAAATACCTGGAAAGTGAATGTGCATTACAAAGGGCCACCATGGACTCAGTCTGACTCTGCACCAGCAAACCCTTGGACATCAACTGAAAGTTTCACAATCGCATCATGGACTCATGGTGTAAAGCAAGTTTTAAATATTTGGACAGAGCCAATAGTTTCCACAGTCACACTATGGACTCAGGCTGAATATTCAACACCAAAATATTGGACAGATACAAAGGTCATTTACATAGTCACACAATTGACCCAGTGTCAGTTTCCAATAAATACTTTGACAGAATCTATAGGAGGCATAATCACACCTTGGACATCTGCTGAATCTCTAGCATTAAGTTCTTTCACACAAAATATTGTTGATACAGTCAAATTTTGGCCAGTGCTTAAAACTGAATCTAAGAAAAGGTGGAATCTGCCTCAAAGTGGTACACTCATGTTTTCACTAAATCCTCAAATTGATACTTTTGGATCCTTGAACCAAATTGAAAACCAAGACTCTCCTCTGTGGACACATCCTGAAATTGATAATGTCAATACAATGACCTTTCTTGAACCTGGAACACTCATATCACAGGTAGTACCTTTGTCCCAAGCAGCTAGATTCTGGCCCCAAACTGAAGCTGATATTAGCAAAACTTGGTTTGTATCCTCTGAAAAAATAAATTCCTGTGCCCAATCAGAGTCTCAAAGAATGAGTACCTCAACCCATTTTGGAGTGGGTAGAGTGAAGCCCTTGGCCCAACATGAAACTTCTAGAGTCATGTCATTTCTTCAGGTTGAAATTGGTATAGTCCGCCCTTGGAACCAGCCTGAGGGAGATACAGTGAGGTTCTGGCCACTTTCTGAAACTGAGGATGTAAGAAAATGGATCCAAACTGGAGCCAGTATAGTCAACTCTTGGACTCAGCCGAGAAGTAGTATAGTCAGAGCTTGGACCCAAGTTGAATCTGAACTAGTCAGACCTTGGACACAATCTAAAACTAATGCAATTACACTATTGACCCAGGCTGATACTTTCACACCTTGGTTCCAAACTCAAATTAATGCAATAAGAGAAGGGCCCCAAACTCAATCTCAAATAGTTACTACTTGGATCCAAACACAGTTGAAAATAGTTCATCCCTGGATTCAACCTAAAAGTGATTCAATCAGAATTTGGATCCAGCCTTGGATTCAAGCTGAAACCCACACAGTCGGACTCTATTATGAAAGTGATATAAGAAAATCATTAACCTCATCTGAATCTCAGGCAGTCACATTTTGGTCACTGAATCAAAATTCAGTTATGACTTCATTTAACTTGGAATCTCAGATAACATGTTCCTGGGTCCAAAATGAATTTAACACAATCAGTCCTTGGAATCAATATGAAACTAATTCTGTTGGATCCTGGATCAAGTCTGAAACTGGTACATGTCAACCCTGGCTCCATACTGAATCTTCTACAATCTTACCATGGACCCAATATGAAACTTTAGAGATCTACCCTTCAAACCAACCTGAGACTGATACATCAATAGCGCACTTGTTCCAGCCCGAAATCGATCCAATTAATACTTGGAATCAGCCTGAAGTAGATACAATCAGATTCTGGACCCAAGTTGCAACAGAAATAATTCCAGTTTGGACCCAGATTGGAAATCAGGTGCTTGAACCTCCCAACTTTTCTGAAGCTGGCATAGACATACCTTGGTTACAAACTAAAACTGATGTAAGTAGACCCTGGATTCAACCTGACTTTCAGTCAGTCCATCCTTGGACCCAGTCTGGATTTGATATAATTAACCTCTGGTCTCACCCAAAAGCTGCTGTAAATCAACCCTGGACATATGTTCAAACACAGGCAATCGGACCCAGGATCAAGGTGGAAGCCAATACAATCAAATCATTGTTTCATGTCCAAGTGAAAAAAGTCAGACTGGGGATTCCTTCTGAGTCTCAAATATTGAGTTTCTGGATGCAGCCTGACATTAGTAGAGTTAATGCTTGGATCCAACCAGCAACCCAGGCAGTCAATCCTGCGTTTCACCCTAAAACTGGTAATGTTGCATCCCTGGCTATTCATAAACCTGAAACAGTCAGAATATGGATCCAGCCTGAAACAGAAGTAAGGCCTGGCATCATTTATAAAGCTGATATAATCACATCATTTGCTTCTCCTGAAGTTGAACAAGATGCAGCAATTAGTCACTTTGATTCCTGGTCTAACTATGTAACATTTTTACCAATAGAAACTGTTCCTTCCCTACATGAGTATTTTGCAGCTTTGTCAACTGAAATAGCTGCAGTAGAAAGTCAAGGTCAAACAAATTCTGTCCAACCCAGTGAAATCACAAATATTCTCTATCTTACACTTTCAAGCACACGACTTCCTGGAGGAGCTGGTTACCTGAACTTTGGCAATGAATTACAAATTACCAAAACAAAAGGAAGCCCTGATGTCCCATCTAGTTCTCTCAACCCACTTTATCCATCTTTTTCCTTTCCTGTTCCTTGTTTTTTCCCACCTTCATGTTCTTTGCCCCTTACCTGTTCAGTCTTTTCTTCTTGCACATTTTCTTCACCCTGTACTTTTCTTTCTTGCTCAATTTGTCCTCTTATGGCCTTCTCTCCTGTTCTTCCCTTACCTGCTTCTGACAGTTCTTTCCAGAAACCATCTTCCTCAAAATTTACTGTAGACACCATTCTTTCCCATACTTTTTCATCCTTGCATGCTGCT

>RAE Fruit bat

ATGTTCTGGATGCAATTTGATTCTCTATTAAATTCATACACACAGAGTCTAAATGATACAGTCACATTGTGGACAATGATTAAAACTGAGTCTAAGCAACCTTGGCGAGTACCTGAACCTAGTATATTTAGTATTTCATTGCATCCACAAATTGATACTTCTCGATCCTTGATTACAGTTGAGAATCAAGCTTCCTTTCTTTGGACACATCCTAAGACTGATAATATCAACTCATGGGCCTTGTCTAAATTTGGAACATTTATATCCTGGGTAGTGCCCTTGCCTCAAGCAGCCAGACTCTGGCTCCCCTCTGAAGCTGATATTAGCAGATCTTGGTTGAAGACTGAAACAACAAGAGTAAGACCCTGGGGCCAATCAGAATCTCAAACAGTGAGTACCTTGACTCAGTTTGGACTTGGTAGAGTGGATCTCTTGGCTCAACATGAAACTGGAACAACCTTATCATGGATTCAGACTAAAACTGGTGTATTTCATCCAGGGAACCGGTCTAAAGGAGACACAGTGGGACCTTGGACCTTTTCTGAAGCAGATATAGTAAGACCATGGATCAAAAATGAAGCTGGTGTAATCAGTCTCTGGACTCAACCTAAAAGTAGTACTTTCAGACCTTGGGCCCAGCCTAAATCTGAAGCAGTGAGGCCCTGGACACAATCTGAAGCCACTATGTTTACATTATTGTCCCCATCTGAAATGCAAGCAGCGAAACCCTTGAATGTGACTGACATTAATATGGTCAGATCTTGGTTCCAGACTCAGTTTGGTGCAATAAAACAAGGCACTCAACCTCAATCTCAAATAGCTACTCCCAGGATCCAACCAAAATGGCAAAGAGCCCACCCATGGATCCAGGACAAAACTAATACAGTCAGATTTTGGCCTCATCCTGAAGGTGAGTTAGCCCAACTCTGGACTCAGACTGAAACCAACACAGTCAGACTCTGGACCCATCATGAAAGTGATACAGTAAAACCATGGACCCAGCCTGAGCTACAGTTTGTCAGTTCCTGGACTGATGTGGATACAGTCACATTTTGGTACCCAATGCAAAATGGTACAATTAGGCCCCAGTACCCATCTGAATCTCAAAAACCGTGTTCCTGGACACAAAATGAAGTTGATATAATCATCCCTTGGACTCAGCATGAGAGCAATTCAATCAGATCTTGGACTAAGCTTGAAACTGCTACAATCCACCCCTGGATCCACCTTGAAACTGCTGCAATCAGACCATGGACCTTGGCTGAAACTTTAAAAATCTACCCCTCAACCCGGGTGGATACTAATACAATAATAAGACATTGGTTTCAGATTCAAATAGATTCAGTCAAACCTTGGAATCTACAGGAAGCTACTACAGCTAAACCCAGTACCCAGCCTGAAACTGAAACACTCCATGTTTGGTTGCAGACTGCAAAACAACTAGTAAAACCTATAACTTTATCTGAAGTTGATACAATTACTCCTTCATTACAGACTCAAAGTAATACAAACAGAGCTTGGATTCAACCTGACTCTCAGACAGACAGTCCCTTAACTCAGGCTAACGTTTCTGTAGGTCACCCCTGGACTCAGCAAGGAGCTGTTAAAAATCAACCCTGGATGTATGCCAAAACTCAAGCAGTCAAACCCTGGGTCAATATGGAAGCAAATACAGTCAGATCTTTGTTCCACATTCAAATGAGTAAAGATATACCATGGACCCATTCACAATTTCAAGTATTCAGTCCACGGATCTACCCTGAAGTTGGTATAGTTGAGTCTTGGATACAGTCTGAAACCCAAGAAGGCAGGCCCTGGGCCCACCCTGAAACTGACATTATTGCATCCTCTGCTATACTTACACCTGACAAAGTCAAAACACAGACTCCACAAGAAAAAGAAAGAAAGCCTGACAGCCATTATAAAGCTGATATAATTACATCATTTGTTCCTCTTGAAGTTGAACTGGATAGAGCAACTCCATTAACTAGTCAGTTTGGCTCCTGGTCTAAACCTGTACCTTTTTTACAAACAGAAACTATTCTTTCCACAGATCAGTATTTTATAATAGCTTTGTCAACTGAGATACCTGCAGCAAAAAGTCAAGATCAAATCAATTCTCTCCTACCCAGTGAGCTTACAAACATTCTCCCTCTTACCCTTTCAAGCACCTGGCTTCCTGGAAGAGTCGATTATCTGAACTTTGGCAGTAATTTACAAATCACCAAAACGAAAGAAAGCCCTTATGTTCCATCTAGTTTTCTTAGCCCTCTTTCTCCATCTTTTTCCTTTCTTGTTCCTTGTTCTCTGCCAACTCCATGTGCATTGTCCCCTTCCTGTTCATTGTTTTTTTCTTGCACATTACCTTCATCCTGTATTTCCCCTTATTGCTCCATTCTTTCTCCTATGGCCTTCTCTCCTGTCCTTTTGACCTTAGCCTCTTCTGATAATTCTCCCCAGACACTATCTGGGGAGACCTCAAAATTTACCAAAGAGATCTTTCTTTCGCACGGTTTGTCATCCCTGCGTGCTGCT

>MEU Wallaby

ATGAAGGCTGTTTTGGGCCTCTGGGGGCTAAGCCTGGTTCTCTCCTGTGTTGCTGGCTTCTCCAGCCCTCAGAAGTCAAAGCAGGACTGGGAAACCCTAAGAAGGCCAAGAAACAACCAGGTCGTGGGCAGCCATGAGAAGGTGCCGTGGCTGGTATCTGTGGCTGGGGTCTGCCAGGGGGTGCTCCTGAACCACTGGTGGGTCCTCTCCACTGCCAGCTGTCTGAAAAATGTAAATCCAGGCCTCCTGCCTATGAGGGGCTCAGGAACCTGGCTCAACGGCTCCTGGATCTGCCTGCACCCCAACTTTGTGCCCCCAGCCAGGGCCATTGCAGCCAAGAGTGACCTGGGCATGGTCCTCCTTCATCAGCCAGCCAGACTGGTTGGGGGTCAAGTCCAGTTGGCCCGAGACACTAGCAAGGCCAAAAAAACCTGCCCCTTCTGTGGACATCAGCCATGTGAGGTCTATCAGCATCAGAATCCCCGAGAGCCTGGACTCACACCAGCCAAGAGGATGATGGTGAAGTTCCTGGGGTCCTGGGCCTGCCGTCACTTGGGAGTCCAACTTCCTGAGCCTGAAAGTCTTTGCATTTGGAGCGATTCCTGGCCAGGCCCAGACTGTTGGGTGCAGCCTGGCAGCCCGGTCCTCTGCCATTTTGGAAACCGCTGGGAGCTTGTGGGGCTGGTCAGTGCGCCCACAAAGATCTGCTATGTCCCCGTCCTCGCTGTCCGCATAACTCCATACCTCAGCTGGATGGATGCTGTGGTCCAGGCTACCCAGAAGCAGCCTCTGACATTCCCCTGCAGCCATCCAGACAGCAGTCCCATCGCCAGCTTGGGGTTGCAGGTCACTCCCCGGCTGTCCTCTTCCCAAGAAGAAATCAGCCGCGTACACAATGCCCCTGGGCTTGGGGGTGGCGGGAAGGATAAGCAGCTTTCTCTGGCTGTACGATCGCCCTCAGAGGTCAAGCTTCCTGGAGCTGCCACTTGGCTCCTCCATGATGAGACAGCTGAGTTCCAGGCACTCCCTCCCACTGGAGAGCCCCAGGTGCAGATGTCCCAAGATCAGCCTGGGCCTTTTCAGCCTGTCCATGAGCCAGCCCTAGTTCAGACCCACTCAGTTCCCAACAAAGCCAGATCCCAGCCTCAGCAGGGTGAGCACGTGCCTGGACCAAAGCCTCAATCAGCTTACTTGGGCCTCAGGAAAGCTCAGAACGCGGTCAGACCAAAGACTCTCCAGAGTGCCAATGAAGGTCAGACTCACCAGGCTGAGAAGCCAGCCAGGCCCCAGGCTCATCCTAAAGCCAATCCACTTGGGCCCTCAAATGTCCCCAAGACAGCCCTTGCTGGACTCTGGCCTCCTCAGTGGGTGGCTGACACCATGAGTCCCTGGGCTCTCTCTCAAACTTTATTGGCATCCAGATCACAGCCTCATCTGCTGACTGAAACAGGCAGATTCCCCAGGGTTTCTAAGGCACATGCAGCCAGGCCCCAAACTCATCCAGCTGCTGGATCTCCTGGTCAGTCAGCAAGTGATATTATTGGACCCTGGGTTCTTGCCAAAGCAAATCCAGCTGAAGTAGAGGCTCCCCCAGAAGCCAGATCCCAGGCCCAGCCAGCACCTAAGAGAGCCAGGCCCTGGATGGTCCCTAAACCAGAAGCACAAGAGATAGCTGGGCCCTGGGTTCCATCTGAGGCAGAAGCAGCTGGTCTTCAGCCTCAGCGTGCGCAGGAGCCTGCTAAAGCCCAGACTCAGCGGGAGAGCAATCCAGCCCATGTTGCCTCACCTGTGCCCCAGCCAGTGGCTGATGCAGCTGGGGTCTGGGCTGTGCCAACGCCAGCTGTGGGTGCCATCATGCCCTGGGCCGGGCCTGTGACTGGGAGAGCCTCCAAAGCTATTATCCCATGGGCTGGCCCCGCTCCAGCCAGAGCCCTGGCACCCTCCATCCTGCCTTCACCAGCTCCATTGTCCTCAGGCTCCCTCACCTACCCAGGCCCTTTCCTCTCCCCCAGATTTTTCCCTTCATCCCAGTCATTGTCCCCAGACCCTTCTCAGACTCACCTTGCTCCCTTCCCTGGAGATATGCAGGCATTGGCCTGGGGCCCCTCAAGACCATCAATGCCTCCATCATGGCCTGGCCCTTCCTTGTCATCTTCCTCTGCCCCC

>OAN Platypus

ATGAAGTTCCCGCATCTGGAGGTTATGGGGGGTCCCGGCCCAAGAAGCAGCTTTCAGGGGAGCCAAGTCTGCCTGCACCCCAGTTTTAACCCCCAATCCTGGGAAGGGCCAGCAGTAGCTGCCCTAGGACTACTCCTCCTGAAGGAGCCCACAGCAATGCATAGGGACAAGGTGTGGCTCTCCAGGACTCCAGGCAACCTCCAAAGGAAGTGCCTTCATTGCAAGCAAAGGCAGTGTCAGGTCTACCAGCGCCAGAAAACAGCTTCAGATAATCTGCGACATCCGGGTGAGGAGATCACGATGATCCCCGTGAGGCTGCTGCATTCCTCGGCGTGCCACCGATACGGAGCCCGTGTGGAGGAGGACAAAAACCTCTGCATCAAGAGCAGAGACCATCATAAGTCTGACTGCCAGATGCAGCCGGGCAGCCCACTCCTCTGCCTCTTCGGGAGCCGCTGGGAGCTCGTAGGCCTCGTCGACGCAAGCCCCGGGAGCTGCTACGGCCCGACCCTCTCCATCAGGACCGCTCCCTATTCCTCCTGGCTGAGGCAGCACGTCAAAGCAGCCACACCGCTCCACTGCAGCCCTTACCCGCTGATGGAGCCGGCCACCCCCGGAGACTCCACTCTCAACTTCTCAGAACTAGCCTCCAAGTTCCAGCCGACGCACCCTTGGCAATTTAGGAATGGAAACACGGACATCTCACAAGTGACCTGGCTTCCCCGACGTTCCCCTCCGACGCCGTCGGCTCCTCCGTCCCCTCTGCCTACTGATGCCCCAGCTTCGTCTCTCCTCAGGCCCGGGGGCGGCCTTTCTTTTCTTCCAGCCAGCCACCTGCCCTCTGGCGACAGTTTGCCCATGGTAACCAACTGGGCTCCCCTTGTCACGCAGACAGCAGGAGCCAGGTCTCCCTTCACCGGCTCGACGGTCGTACACTCGGTTCTGTCCACGGGTCAGGCGGCTGCACTTTGGACTCCCTCTGGCGCGCAGCCAGTCAGATCTTCGGTGACCTTTCCCTCACGGTCAGGTGAACCGTGGACTTCGGCTAGACCCCCGGTTTCTATCCCTGCCCTGGGGCTGGCCCCGCTGGCTTCCCGTGCCCCTGGAATGGCCGAACTCTCCCCACCCTCAGCAACCCAGATCGTCGCACCGCCCTTGGCCGGCTGGACTCCTGCCAAGGCTCCAACGATGGGAACCCCTCTGGCCTCGACAGCCAAGACCCCCCAGTACCACTTCCCTAAAGTCTGGCCCGCTGCGCATGCTTCCTACGGATTCCATGCAGACGTCCAAGCCCCAAGCAGCAGACCTATTTTGCCCTGGACAATCCGCCCTGGCGGAACATGGCCTACTGAGCCCGGGACATCCGACGTGGCTTTCCCCCAGCCCCCTCCCAGCCCCTTCGTTACGGAGAGGTCCGGGGTTATTTCCCGTCTCCTGCCTTCCGAAAGTCCCCTGTATAATATGTGGACCAGGGGTTTGGCTAAGCCTAAAGCCTACTCTGGTGACTACTTTCACGTGGCCTCCTCTTTCCCCACGGCTAAGCCCAGGACCCTGGGTGACCTGCCTGCCTTTCCCCCACCTTTTAGCACTCCCGAGCCCCATTCCTTTCCTAGGCTTCTCCAGCAGGTGATGAAGCCAACTCCTCAAGATGCCATTTCTTATGGCAGTTCCTTTCTAAGGGAGCCGGGCTCCTCCCTGAAACCCCTCGTTTTGGTCACCCCTCGAACCAGACCCCTGTCTCCCGTGCACTTCCCATGGGTCTCGGAAGTTGGACCGGC

>TAC Echidna

ATGAGACCCCTCCCTTGCCTCTGCCATCTTCTGTGGTTGGTGTTCTGTTGCGGTGGCTCCTTTACAGTCTACAACGTCCACGAGCAACGGCCGAGCCGGCCTGCCCCGCGGAAGTCCCAAGGTACCAGAGAGTTTCCGTGGCTGGTGACAGTGGCTGGGACTTGCCAGGGAATAAGCCTCGGCCGCTGGTGGATCCTGACCGCCACCAGCTGCCTGCTGAAGACGAAGTCCCCGCGTCTGGAGGTTATGGGAGGTCCTGGCCCAAGAAGCAGCTTACAGGGAAGCCAAGTTTGCCTGCATCCCAGTTTTAACCCCCAATCCTGGGAAGGGCCAGCAGTAGCTGACCTAGGACTAATCCTCCTGAAGGAGCCCGCAGAACTGCCTAGGGACGAGCTGTGGCTCTCCAGGATTCCAGGCAACCTCCAGAGGAATTGCCTTCATTGCAAGGAAAGGCAGTGTCAGGTGTACCAGCGCCAGGAAACAACTTCAGATAATCTGCGAAATCGGGGTGAGGCGGTCACGACGATCCCCGTGAGGCTGCTGCGTTCCTCGGCGTGCCGCCGACATGGGGTCCGTATGGAGGAGGACAAAAGTCTCTGCATCAAGAGCAGAGACTATCAAAAGTCCGACTGCCAGATGCAGCCGGGCAGCCCAGTCCTCTGCCTCTTTGGGAGCCGCTGGGAGCTGGTTGGCCTCATCGGGGCCAGCCCAGAGAGCTGCTACAGCCCAACCCTCTCCATCAGGACCGCTCCCTATTCCTCCTGGCTGAGGCAGCACGTCAAGGCAGCCGCCCCCCTCCCCTGCAGCCCTCACCCGCCAATGGAGCCAGCCACCCCTGGAGACTCCCCTCTTAGCTCCTCCTCGGAACCGGCCTCCACGTTCCAGCCGACGCACCCTTGGCAATTGGGAAATGAAAACCCGGACGTCTCTCAAGTCCCCCGCCGTTCCCGTCCAGGGCCACCGGCTCCTCCGACCCCTGGGCCTACGGATGCCCCAGCTTCGTCTTTCCTCAAGCCCGGGGGCGGCCTTTCTTTCCTTCCGGCCAACCACCTGCCCTCTGGTGACAGTTCGCCCATGGTAACGAACTGGGCTCCCCTCGTCGGCCAGACAGTAGGAGCCAGGTCTCCCTTCAGCGGCCCGACAGTCGTACGCTCAGTTCTCCCCACGGATCAGGCGGCCGCACTTTGGACTCCATCTGGAGGGCAGCCAGTCAGATCTTTGGTTACCTTTCCAGCGCAGTCAGGTGAACCGTGGACTTCGGCTAGACCCCCGGTTCCTGTCACTGCCCTGAGGCTGGCACCGTTGGCTTCGCGTGCCCCTGGAATGGCCGTACTCTCCACACCCTCAGCAGCCCAGATCATCGCACCGCCCTTGGCCAGCTGGACTCCCGCTAGGGCTACAACAATGGAACCCCTTCTGGCCTCAACAGCCGTGACCCCCCGATACCACTTCCCCAAAGTCTGGCCTCCTGCACCCACTTTCTACAGACTCCAAGCTAACGTCCACGGTCCAAGCAGCAGACCCATTTCGCCCTGGACGATCCACCCTGGGGGAGCACAACCTACCGGGGCCGGGTCATCCACTGTGGCTTTCCCCCAGCCCTCTCCCAGCCCCTTCGGTACGGAGACGTCGTCCAGGATTATTTCCCGTCTCCTGCCTTCTGAAAGTCCCCTGTCCAATATGTGGACCGGGGGTTTGGCCAAGCCTACGGTCTACTCTGATGACTACTTTCACGTGGCCTCCTCTTTCCCCACGGCTAAGCCCAAGACCTTGGGTGACCTACCCGCCTTTCCCCCAGTCTTTAGCACTCCTGATCCCCATTCCTTTCCTAGGCTTCCCCAGCAGGTGATGAGGCCCACTCCTCTCTATGGGCCAGATGCCCTTTCTTACGGCAGTTCCTTTCCCAGGGAGCCGGACTCCTCCCTGAAGCCGTCCTTTTTGGTCACCCCCCAAACCAGACCCTTGTCTCCCGTGGACTTCCCGTGGATCTCTGAAGTTGGGCCAGC

>ACA Anole lizard

GGCTTTCGACAACAGAGGCCCAACTTGGACAGGCTCCCAGAAGAGAGTGAAGAACCAGAGGCTCCATGGATAGTGAACATTTTTGGTAATGGGAATCGCTGCCAAGGAGTTGTCCTGAGCAGCTGGTGGGTCCTGACAGCAGCCAACTGCTTCCTGTCGATGATGCCAAGTCATGTAGAGTTGACTGGGGCAAGTGGGCGGACTGCCACTGAGACGGTGAGCCAGTTTGTCCCACACAAGGGCTTCAGTTCCTGGGATGAACAACCTAATAATGACTTGGGACTCGTTTTGCTTGGCCAACCACTTGATCTAGCAAGGGAAGATGTGTGGCCAGCTTGTATCCCCAGTGACGACACAGCTTCCAATACACGGGAAGAATGTAAGATATTTGAACGAAATGAGAAAGGATCCATACAGGAAACTAAAGTGAATGGTTTGGAGACATCGGAATGTGCTATTATTTGGCCTGACACAAAAAAAGAATTGAATTTGTGTGTTGCAAGAAATATGTCAAATGGCACATATTGCACGGTGCCTGTTGGCAGTCCTGTGGTCTGTCATAACCCAGGCAATGGGAAATGGGAAGCCATGGCCATCGTGACCCAGAGCTTGCTGAACTGCACAGCTCCTATCCTGGCCGCCCAGCTTCTAACTCATCTACAGTGGTTGAGGACAGAAGGAGCAGCAGAGGACACTGAGGAACCGTCTCCTGTACCAGAACAAACATTGACATCATCATATTTCACATCTGAACCAATTCTGCAAGCATTAATAGTTCAGCACATCACTGAGGAACCTTCAACGCCAGCAAAGCCTCTTTCCACAGCCAATAATGTAATTCTTATAGCACACTCCACAGCCCAGAAATTACCATATGAAACACCAATAAAAATGCAGTATGTTCCCCAAACTACAAATCCACTTACAGCTACTAGCCAGTCATATACAACAGCCAAAGAAACATCTGCAGTTCCCTCCACAACAAATCAGCAAACATTAGCAGCAGCACAACAATCATTTGTAACATCCACGGCAGCACAACAGTCATCTGTAACATCCACGGCAGCAGAACAGCCATCTACAACACAACAATCAACATCAATAGTCTCTTCTACACCCAAACAAGCAACTGTAAAAACCCCAACCTTCAAACCACCACATGTAATAATAATACCTGCTACCACACCAAGGACAACATCCCCCCTGCCAACAACTGATGTTAATCGAGGTGCACCTGTTAGCTTTGTTGTTGCGGGTT

>PVI Bearded dragon

ATGGAGCTTCTCCTTGGGCTCTGTGCTCTTTTCCAACTATTTATCGTCTCCTCCGGCCTCTGTGGCTTTCGTCACCTGCTGCCCAAGCTGGCTGAAGCCATGCCAAAAAAGGGTGCAACTGTGGAAGCACCGTGGTTAGTGAACATCTATGGCAATGGCCAGAGGTGCCAAGGAGTGGTCCTGAGCAGCTGGTGGGTGCTGACCGCAGCCAACTGCTTCCTGTCAATGTCGCCGAGCCATGTAGAATTGACCGGGGCTCATGGGAGTATTAACACTCAGTCGGTGAGCCAGTTCATGCCACACAAAGGCTTCAGTACCTGGGATACAGAGCCTAACAATGACCTGGGACTCCTTCTGCTTAGCCAGCCACTTGATTTAAGAACGAAAGATATGTGGCCAGCTTGTGTCCCCAAAGAAGAAAAAGCTGACAACACACAAGAGGAGTGTAGGATTTTTGAACGAAGCCAGGACGAATCGACACTGAAAGAAATTGAAGTGGAGGCTTTGATGACATCAAAATGTGTCAAACGCTGGCCTGGAACAACAGAAAAACTGAACTTGTGTGTTGCAAGAAAGAACTCTGACCAAACTGATTGCAGGGTGCCCATAGGCAGTCCTGTGATCTGTCATAACCCGGACAACAAGCACTGGGAAGTAATGGGCATGGTGAGTCGGAGCTTGCGTAAGTGCACTGCTCCTATCTTGGCTTCCAAAGTTTTAACACATCTGGAGTGGTTGAAGAAAGAAAAAGCAGTAGAGGATCCTCTCAATCCACGACTTGATGATAACAACCAGTCATCTGCTCCAGAAGAAGAAGAATTGTCTCCAACCATTGGACAAGCTGTAACATTCCCAACAGGATTGATCTATAAACCAAGATCCCAGGAACCTTCAGCAGCTTTAGAAGTGCCAGTATCACTCCCAGATGCAGAACCAACACCTGTACATCCCAGAAAAGCGACAAAGCCTTCCTCGACTGCCAATAATGTTATTTTGGTAGCATCTCCAACCATCCAGCGATCATCACGCATTGCTCCCACAAGAACCAAAACATCAAGGCGAAAAACATCAGCTTCAGTACCCTCCACATCTGAACAGCAGTCAATTGTACAGTCCACAGAAACACAGCAGTCATCACCTATAGTCTCTTCCACAGCCGAAGAAACATCTGTAGCACCCACAACAGTCGGAAAATCTACAGCAGAACAACAGACATCAGCTGTTGCCTCTTCAACAAACAACCAAACACCTACAACATCTACAACACTTCTAACATCTACAACATCACAACAGACCTCCACAACAGAACAGCAGCCATCCACAACACAGCAGCCATCAATAGTCTCTTCCACAACAGAACAGCAGCCATCCACAA

>RBI Caecilian

ATGAAATACCATTTTCTTATTGCCATCAATTTGGTGTTTCAGTGTTTAAGCTACAGCAATGCACTGAGCCAGGAGAGATGTTGTAAAGAGGAGACAGATCCGGGCGCTCTTCCACCACTTCTAAAGTTTGTTAGCTGGGATCTAAGCTTTCTGTCATCAGATCCTGCTACTCGAAAAAGGCGTTCCGTATTAGCCAGTGCACCTCTAGCTGAGCAAAAACACAACAATAGAGACCAGCATCACTATAAAGCCAATTCTTACTCATCCAGGTTACGACATTTGTTCTCCTCAAGATGGCCACATGGCCATGGAAGGCCACATCAAGCTCCTGTTAGATCACCTAGGACAAGACACCATGGCCATCCTCATGCCTTAGCAAAGCCATCCGATTCAATTCAGCCTCTGGTTGTACATCCACCAGCTGGTCCAAGTCAGGCACTTCTTTTACTATCACCAGCTGGCCCAGGTCAGCCTCTGCACGTATTGCCACTGAGAATGACAGATCCACCAGAGTTGCGGAAACCTTCAAGCCAAAATCTTCCTCTCACCTCATTGCAGCCAACAAGCCAGAGTCAGTCTCTCACCTCACTACAATCCTCCAACCAAAGTGGACCACTATCCTTACTGCAGTCGTCCAGCCAAGCTCAGCCTCTCACCTCACTGCAACCCTCCAACCAAAATGGACCACTATCCTCACTGCAGTCACCCAGCCAAGGTCAACCTCTCACCTCACTGCAATCCTCCAACCAAGGTGGACCACTATCCTCACTGCAGTCACCCAGCCAAGGTCAACCTCTCACCTCACTGCAATCCTCCAACCAAAGTGGACCACTATCCTCACTGCCGTCACCCATCCAAGGTCAGCCTCTCCCCTCACTGAAATCTTCCAACCAAAGTGGACCACTATCCTCACTGCAGCTGCCCAGCCAAGGTCAGCTTCTCTCTTCACTGCACTCTTCCAACCAAAGTGGACCACTATCTTCACTGCAGCTGCCCAGCCAAGGTCAGCCTCGCCCCTCACTGAAATCTTCCAACTTAAGTGGACTACTATCCCCACTGCAGCTGCCCAGTCAAGGTCAGCCTCTCTTCTCACTGCAGCCATCCAGCCAAGGTCAGCCTCTCACCTCACTGCAATCCTCCAACGAAAGTGGACCACTATCCTCACTGCAACCATCCAGCCAAGGTCAGCTCTCACTGCAGACATCCCGCCAAGGTCAGATTCTCCTCTCGCTACAGCCATCCAGCCAAGGTCAGTTTCTCCTTTCGCTGCAGCCGTCCAGCCAAGGTCAGCTTCTCCTCTCGCTGCAGCCCTCCAACACAGACCAGCCTCTTGTGCTGAAACTTCCTGACACAGGTTTGCAGCAA

>CPY Fire-bellied newt

GCCTTCCTTCCCTCCAGCGCCGAGAACCAATGGCTGCTTCCTGGATGCAGCCCCAGCAGAGGCCCTGGGAGAAGGATGAGACTGCCGCTGCTCCTGCTGCACATCCTGGGACTGCAGCGCTGCGCGGGCTACTGTGGCATCCGTAACCACCGGGGCAGGGCGTACCCAGCAGCTTCGGACATCCCCTGGCTGGTCTCAGTGGCTGGAAACGGGCAGATCTGTGAAGGCGCTATCATCGACAACTGGTGGATAATAACAGCTGCCAGCTGCCTTGTCATGACGAAGCAGGGCCGAGTTTCAGGAAGCAGAGTCCTAAGTGGCGTGCCGGGCATGCAGGTGAACCGAGCCATCATCCACCTGGAATACACCTCCGGCCCTGGGGACACATCTATGTTTGACATCGGCCTCATTCTCCTGAGGGAGCCGCTGGCCTTCCACCAGAACCTCTGGCCAGCCTGCTGGCCTGCAGAGGACTACAACATGCTGGGAGCCACACACGCCTGCTGGATCTTGGGAGTGCGGGCCATTGATGAAGACCCACGGTACTGGAGTGGGGGTGTCCACAGGATCCAGGTGCAGTTGGTGGAGCCATCAGAATGCCTCTGGTACTGGCCTGACATAACTCGACAAGACCTGTGTGCCAGGAATAAAGCGAGGACGAAAGGACGCTGCCAGATCCGCCGTGGCAGCCCACTCGTTTGCTTTGACATTTTCCACACCAAATGGGTCCTGGTTGGTTTAGTAGGACGGGCGTTGAAAGACTGCCAAGTGCCTGCCCTCGCCACCAGAACATCAGTCTTCACGGAGTGGGTCACTCGAGAGACAAAGACAGCTGGACATCCATTCTACCCTACAACTTTCATGCTGACTTCTAAACACCGAACTCAGCGGAAATTCTCTGGTGCTGAGGAGAGGGTGGCACAGGGTGTGCATCTCTTTGCAGCTGTACGTTGTACATATGGAGACCGACCCTCCGAGGCTCGCCTTTGGTCGGGATGGCCTCTGGATGTTAACCAACTGCCCCGATCTAAGAGGACCAAGTTACACAGAGGCAGGCTCTTACACTCGCTTGTGAAACCTATTTTTAAGAAGCTCCATTTGTTAGCTGTCCACCTAGACTTTAGACGGTGGTTTTCAGCCATGCTCACTTCCCAGGGTAGCGCAAGGAGGCAAGGGATTCCTGTGGTCAATTATCTTCTGAATCAAAAGCCCACAAACACTACCACAGTGCTGCCCTTACCTCCAGACATATACATTACACCAGCGTCTGTTGAGGCAACACTACCTTCTCAAGTAGCACATTCTCGCTACTCTCCACCATCCGTGTGGGTAAAACCTACCAGACCCACGCGAGTCTCGCTTGTAAGAAGGAGGCGTTCTGCAGTGCTGAGCTTGGAAAGCCAGGTGGGCAGCACCCAGCCAAGGTTGTCAGCACAAGCACCTCAGAATCTACTCACCCTTCCAACCGCGACTTATTTTTTATCCACAGATTCTAACACAATACCTACAATAAGCCTTGCCACTCTCATGCCACGTTCGGCAACAGGCGCAAGCAGCAGTGTAGCTGTTCTGCTGAACCCCACTTATTTGGTACAGCTCACCAAATCCTCAATACTGGGCAAAGAAATAACATTCTTTAACCATCCCACATCTGAAATCACAGCTTCTAGCACCACCAACAGCAGCACACAGAACACCACCACCACCATAAGCACCACAGTTTCCACTGTAAACACATCCACCACCACCACCACAATCAAAATGTCAACCCAAATACAATCAGTTTGGATTAAGACTCGATGGCCAATTCTTTCTACCTTAACAGTTGGCACACCCATCTTGGCAATCTTGCCAACATTCTTAAATAATCGATCTGCTTTGGTATTGTCAACCATCCAACCAGCTTCAGGCAACTCACTGCTTGCGTCAGCAAAACAACCTTCTCAGGTCCTGCCAGCGTCTGCATTACTGGCACACTCTGCAGGCCCTCGACCGTCCCCTAGATTGCTGCCCTACACCAAGCCACCTTCCACTAGCAAGCAAATCCCTCCATATGTGTTCCCCGATCCTGCAATGCCGCCTTTCATCATCATGCCGCCTAATGCAAACCCTCCATATATCACTGGTATGCCACCTTCTGCAAATCCTCTATTTGGCAAAGACATGCCACTTCTTGTATATCCTCCATTTGGCAAAAACATGCCACTTTTTACGAACCCTTTATTTGGCAAACGTTTACCATACCCTGTATATCCTCCGTTCGGTAGAGGCATGCCACCTCCTGTACACCCTCTGTTTGACCAAGGGATGCCACCTCAAGCATATGTTCCATTTGACCGAGGCATGTCACCTCCAGTACCCTCTCCATCAGACCAGGGTATGCCCTCTCTTGTATACCATCTACCAGACCAAGGCATGCAGCTTCTTGTACATCCTTCACCTGACCACCACATACCACCTCCTCCATCTGACCGAGGCATGCCACTTCCTGTATACCATCCACCGGACCAAGGCATTCAGCTTCTTGTACATCCTTCACCTGACCACCACATACCACCTCCTCCATCTGATCGAGGCATGCCACCTCCTGTATACCATCCACCAGACCAAGGCATGCAGTTTCTCGTACAACCTTCACCTGACTACCGCATACCTCCTCCTTCATCTGACCGAGGCATGCCACCTCCTGTATACCCTCTATTTGACAGGGGCATGCCGCCTCCTCTTTACCCTCCGTTTGATAGAGCTGTGCCACTTTCTGTATTCCCTCCACATGTCCACCACATACCTCCTCCTATATACCCTTTATTTGACAGAGGTATGCCACCTTCTGCATACCCTCCACATGACCACCACATACCACCTTCTATCTACCCTCCATTTGACAGAAGCTCGCCACTTCCTGTACACCCTCCTCTTGAAAGAGGCGTGCCACCTCCCATAAAAGCTCCATACGGCCCTGAAACGCTAACTACTGTATTCCATCCAAATGACCGCAGCATTCCACCTCCTACCTATTCTCAATCCGAGGGAAGCACACAACCATCTGCCTACTTGCAGCATGTCCAAACTGTACCTTTCCCTCCAAATAGACCGTTAATGCCATTTCCCTCATATATGATGCCTGGGCCATTCCTGCCCCTTATGAATGGTGTGTCACAGCTGCCCTATCTCTTGCCACTGCAATCATATCTGAGTGTCCCAGTGCTGGCCCCCACTTCTGTGTCCCCAGCAGCATCCTCTTATGTCGTAGGACCTCAGACTAAGAGGTTTTTTCGAGGCACCAAGAGATTGGTTGTTGCACCTGCAACTGTAGCTGTCTCTGCAAATCCATCTCTGCCCATAACATGGAGCAGACCTCCCTTGATCTTGGCCACCCTTCCCATGACCTTGGGCAGACGTTCTACAACCAGTTCTATCATGACCTCTGCCATCCCTCCTGTGACATGGGTCATTCCTCCTGCTACCTGGCCAAACCCTATCCCTTTTGTGAACTGGGCCAACCCTACTGCATTTTCAGTCAGCTCTCTTGCAACCTTAGCCAGCCCTTCCGTGACAGGGACTATCCCTTTGACGACCAGGCCTCCGGAGACCTTAGCTAGTGCCCCCTTGACCTGGATCATCCCACCTATGAATCTGGCCAGCCCCGCTGGTACCGGGGCAAGCCAGCCCCTAATGTGGACAATACCCCTCATAACCTGGAAAAGCCCTCCTGTAATCATGGATAGCACTCCTGCAATGCAAGTCAGCCCCCCTACAAATCAAACTATAACAATGGCAAACATGGCCAGACCTCTCCCTAACATGATCAGACTCCCCACAATCTTGGTCAACCCTCCTGTGATCAGAGTGGGCAATCCTTCCATTTCTGCCAGTGCACCAGAGGTCAATCCAAGCCCCCTCGTGAATTGGACACCTTCCCCAATGAGCATTGCTACCACCTGGATCAACATGCCCACAACTGAGACCAGCACCACCACAACTTTAACCAGCTCACCTGTGACCAGCATCCCCACAAACGTCCCCATCTTTGAAGTGACCAGTATCCCCACGACCCAGTCCCCCTCTGGTCAGACCAGCATCATCAATACAGAGAGCCGTTCAGATTCAACAGAGTTCCCGACTATCTTGACAAACTCTGATGCACCAGGGATATCCACGATCTGGATTACTTCTGATTCATCAGGAGTTCCCATCATCTGGGCTGGATCTGATGCATCAGGAATTCCCATCTTCTGGACCAGCTCTGATTCAACAGGAATTTCCACAATCTGGACCATCTCAGAAACAACCGGAATCCCCTCAGTCTGGACCACCTCTAATTCTACAGGAATCACCACCATCTGGACTAGCTCAGAAACAACCAGCGTCCCATTGGTCTCGACCAACTCTGAAACAACAGGCGTCTCAACAATCCCAACCAGCTCTGAAACAACAGAAATCCCCATGACCTCGACCTCCTCCAGTTCAGCTGGAAAGCCCACCATTCAGATCAGCTCTGAA

>PWA Ribbed newt

ATGAGACTGATGCTGCTGCTGCTCATCCTGGGACTGCACCGCTGCCAGGCCTACTGCGGCATCCGTAACCACCGGGGAAGGGCTTTCTCCGGCAGGAAGTACCCAGCAGCTTCGGACATCCCCTGGCTGGTCTCAGTGGCTGGAAACGGGCAGATCTGTGAAGGCTCCATCATTGACAAGTGGTGGATAATAACAGCTGCCAGCTGCCTTGTCATGACGAAGCAGAGTCGAGTGTCGGGAAGCAGAATCCTAAGTGGCGTGCCGGGCATGCAGGTGGACCGCGCCATCATCCACCTGGAATACACCTCCGGCCCTGGGGACAGAGCATCAATGTTTGACATTGGCCTCATTCTTCTGAGGGAGCCGCTGGCCTTCCACCAGAACTTATGGCCAGCCTGTTGGCCTGCAGATGACCACAACCTGCTGGGAGCCACACACACCTGCTGGATCCTGGGAGTACAGGCCCTAGACGAAGAGCCATGGCGCTGGAGTGGGGGTGTCAGCCGCATCCAGGTGCAGCTGGTGGAGCCGTCGGAATGCCTCTGGTACTGGCCTGACATAACTCAACAGGACCTGTGTGCCAGGAATAAAGCAACGACTAGAGGGCACTGCCAGATCCGTCGTGGAAGCCCACTCGTTTGCTTTGACATTTTCCACACCAAATGGTTCTTAGTTGGTTTAGTAGGGCGGGCGTTGAAAGACTGCCACGGGCCTGCCCTAGCCACCAGAGCATCAACCTTCACAGAGTGGATCACCCGAGAGACAAAAACAGCAGGACATCCATTCTACCCTACAACTCTTATGCTGGCTTCTAAACACCGAACTCAGCTGAAGTTCTCTGGTGCTGAGGAATGGGTGTCACAGGGTGTGAATCTCTTTTCAGCTGCACGTTGTACATATCCTGGAAGCAGATCCTGCAAGGCGCGCCTTTGGTCGGGATGGCCTCTGGATGGTGACCAACTGCCCCGATCTAAGAGGACCAAGTTACAAAGAGGCAGACTCTTGAGCCCACTTATGAAACTTATTTTTAACAAGCTCCGTTTGTTAGCTGTCCGCCTAGACTTCAGACGGTACAATTCAGCCATGTTCACTTCTGAGGGTAGTGCAAGGCGGCAAGGGATTCCTGTGGTCAATTCTCTGCTGAACCAAAAGACCAGAAAAACGGCCACAGTGCTGCCATTATCTACACACACACACATTACTCCAGCGCCCATTGAGGCAACCCCGCCTTCTAAAGTACCACGTTCTCCCCACCCTCCACCATCTGTGTGGGTAAAACCTTCCAGGTTCATGCGAGTCTCGCTTCTTAGAAGGAGGCGTTCTGCAGTGCTAACCTTTGAAAGCCAGGTGGGCAGCACCAAGCAAAGGATACCACCACAAACATCTCAGAATCTACACACCCTTCCAGCCAGGACTTACTTTTTATCCACAGAATCTAACACGATCTCTACGATAAGCCTTTCCACTCTAATGCCACCTTCTGCGGCAGGCACAAGCAGCAGTGCAAGTGTTCTGCTGAACCCCACTCTTTCAGAAGAGCTAATCAAATCTTCAGTACTGGGTGAAAAAATAACATTCATGAGCAATCCCACATCTGAAATCACAGCTGCTAGCACCACCAACAGCAGCACACACAGCCCCACCACCATCAGCACCACAGTTTCCACCAAAACCACCTCCATCAAAACGTCACCCCAAATACAATCAGTTTGGATTAAGACTCGATGGCCAATTCTTTCTACCTTAACAGTTGGCACCCCCATCTTGGCAAACTTGCCAACCTTTTTATATAATCGATCTGCTCTGGTAGTGTCAACCATCCGACCAGCCTCCAGCAACCAGCTGCTTGCTTCAGCAAAGCAACCTTCCCAGGTCTTACCAGCGTCTGCATTACTGGCACATTCTGCAGGCCCTCCGCCTTCTCCTAGATTGCTGCCCTACACCAAGCCACCTTCCACTAGCATGCACATCCCTCCATATGTGTTGCCCAATCCTGTAATGCCACCATTCATCATCATGCCACCTAATGCATACCCCCCGTATATCACTGGCATGCCACCTTCTGCATCTCCTCCATTTGGCAGAAACATGCCTCTTCTTGTATATTCTCCGTTTGGCAAAAACAAGCCACTTTTTACAAACCCTTTGTCAGGCAAAAGTTTACCACACCCCGGATATCTTGAGTATGGTAGAGCCATCCCACCTCCTATACACCCTCCGTCTGACCAAAGGATGCCACCTCCCGAAGTCTTTCTATCTGACCAAGGCATGCCGCCTCCAGGATATATTCCATCTGACCGAGGCATGTCACCTTTAGCACACTCGCCATCTGACCAAGGTATGCCACCACCAGTGCACTCACCATCTGACCAAGGCATGTCACCTCCAGGAAACCATCCACCAGACCGAGGCATACAAACTCTTGTACACCTTTCACTTGACCACCACATACAACCTCCTTCATCTGACCGAGGCATGCCACCTCCTGTATACCCTCCACCAGACCTAGGAATACAACCTCTTGTACACCCGTCACCTGACCACCACATACCACCATCTTCATCTGAGCAAGGCATGCCACCTCCTGTATACCCTCCACCAGACCGAGGAATACAACCTCTTGTACACCCGTCACCTGACCACCACATACCACCATCTTCATCTGAGCAAGGCATGCCACCTCCTGTATACCCTCCACCAGACAGAGGAATACAACCTCTTGTACACCCGTCACCTGACCACCACATACCACCATCTTCATATGAGCAAGGCATGCCACCTCCTGAATACCCTCCATCTGACAGAGGCATGCAATTTCCTGTATACCTTCCATTAGACAGAGGTGTGCCACTTCCTGTATACCCTCAATTAGACAGAGGTGTGCCACATCCTGTATACCCTCCATTAGACAGAGGTGTGCCACTTCCTGTATACCCTCAATTAGACAGAGGTGTGCCACATCCTGTATACCCTCCATTAGACAGAGGTGTGCCACTTCCTGTATACCCTCCATTAGACATAGGTGTGCCACATCCTGTATACCCTCCATTCGACAGAGGTGTGCCACTTGCTGTGGACTCTCCACATTTCCACCACATACCTTCTCCTACATACCCTCCATTTGACAGAAGCATGCCACTTCCAGTACACCCTCCTCTTGAAAAAGGCATGCCACCTCCTGTGAAAGCTCCATATGGCTCTGGAATACCAAATATTGTATTCCATCCAGATGACCATGGTATTCCGCCTCCTGGCTATTCTCAGTTTGAGGAAAACACACCTCCATCAGCCTACTTGCAGCATGTCCAAACTATAACTTTTCCAGCAAACAGACAGTCTGTATCATTTGCCTCATATATGATGCCTGGGTCTTTCCAGCCCCTTATGTACGAAGTGTCACAGCTGCCCTATCTCAAGCCGCTGCAATCATATCTGAGGGTCCCAGGAGTGGGCCCGCCTTCTGTATCCCCAGCAGCATCCCCTTCTGGTGTAGGACCTCAGACTAACAGGTTTTTTCGAGGTGCCAAGAGATCTGTTATTGCAACTGCAACTGTAGCTGCCTCTGCAAATCCGTCTTCGCCCATGACCTGGACCAGACCTCCCTTGATCCTGGCCAGCCTACCGATGACCCTGGGCAGACGTTCTACAACCAGTTCTGCCACGATCTCTGCCATCCCTCCGGTGACATGGCTCATTCCTCCTGCTACCTGGCCAAACCCTATCCCTTTTTTTAACTGGGCCAACCCTACTGCATTTTCAGTCAGCCCTCCTGCAACCTTAGCTAGCCCTTCTGTGACTATCCCTTTGACAACCTGGGCTGGTCTGCCGGTAACCTCGACCAGGCCTACTGAGAGCTTAGCTAGTGCGCCCTTGACCTGGATCATCCCTCCTGTGACTTTGGCCAACCCAGCTGCGACCTGGGCAGGCCATCCCATAATGTGGACATTTCCTCTCATCACCTGGAAAAGCCCTTCTGTAATCTTGGGCGGCACTCCTGGGACCCAAGCAAGCCTTCCTCCGAATCAAACTAGAACTTTGGCCAACATGGCCAGCCCTCCCCCTAACATGGTCAGATTCCCTGCAATATTGGTCAGTCCTCCTGTGATCACAGTGGGCAATCCTTACACTTTTGACAGTGCCACAGAGGTCAGTCCAAGCCCCACTGTGAATAGCACACCCTCTCCAATGCGCATTGCTACCACCTGGATCAATGTGCCCACAACTGAGACCAGCACCACCACAACTCTACCCAGCTCGGCTGTGACCAGCATCCCCACAATCTTAACCGTTTCTGAAGAGACCAGCATCCCCACAACCCAGCCCTCCTCTGGTCCAACCAGCGGCATTGTCACAGGGATCAATTCAGATTCAACAGAATTCCCGACAGTCTTGACAAACTCTGATTCACCAGGGATGTCCACAATCTGGATTAGCTCCGATTCATCAGGATTTCCCGTCCTCTGGGCTGGCTCTGATTCATCAGGAGTGTCCACCATCTGGACCAGCTCAGAAACAACTGCAGTCCCCTCGAGCTGGACCAGCTCTGAACCTACAGGAATCCCCACTGCCTTGACCAGATTGGAAACAACCAGCATCCCATGGATCTCAACCAACCCTGCTTCAACAGACATCTCAACAATGCTGACCAGCCCTGAAACGACAGGAATCCCCGAGATCTCAACCTCCTCCGATTCAACTGTGAGGCCCACCACCCAGATCACCTCTGAA
