# Supplementary material for: A gene deriving from the ancestral sex chromosomes was lost from the X and retained on the Y chromosome in eutherian mammals
Source: BMC Biol. 2022 Jun 9;20:133. doi: 10.1186/s12915-022-01338-8 (PMC9178871; doi:10.1186/s12915-022-01338-8)
Supplement: Supplementary file 9 — Additional file 9. Sequences and GenBank accession numbers for PRSSLY sequences. [file 12915_2022_1338_MOESM9_ESM.docx]

**Additional file 9: Sequences for cDNAs of *PRSSLY* homologs**

GenBank accession numbers given for sequences we assembled from RNA-seq. If no accession number is given, cDNA sequences were assembled from genomic sequence. Accession numbers for source sequences (RNA-seq and genomic) are given in Additional file 3.

>Anole, Anolis carolinensis (BK059517)

gtGGCTTTCGACAACAGAGGCCCAACTTGGACAGGCTCCCAGAAGAGAGTGAAGAACCAGAGGCTCCATGGATAGTGAACATTTTTGGTAATGGGAATCGCTGCCAAGGAGTTGTCCTGAGCAGCTGGTGGGTCCTGACAGCAGCCAACTGCTTCCTGTCGATGATGCCAAGTCATGTAGAGTTGACTGGGGCAAGTGGGCGGACTGCCACTGAGACGGTGAGCCAGTTTGTCCCACACAAGGGCTTCAGTTCCTGGGATGAACAACCTAATAATGACTTGGGACTCGTTTTGCTTGGCCAACCACTTGATCTAGCAAGGGAAGATGTGTGGCCAGCTTGTATCCCCAGTGACGACACAGCTTCCAATACACGGGAAGAATGTAAGATATTTGAACGAAATGAGAAAGGATCCATACAGGAAACTAAAGTGAATGGTTTGGAGACATCGGAATGTGCTATTATTTGGCCTGACACAAAAAAAGAATTGAATTTGTGTGTTGCAAGAAATATGTCAAATGGCACATATTGCACGGTGCCTGTTGGCAGTCCTGTGGTCTGTCATAACCCAGGCAATGGGAAATGGGAAGCCATGGCCATCGTGACCCAGAGCTTGCTGAACTGCACAGCTCCTATCCTGGCCGCCCAGCTTCTAACTCATCTACAGTGGTTGAGGACAGAAGGAGCAGCAGAGGACACTGAGGAACCGTCTCCTGTACCAGAACAAACATTGACATCATCATATTTCACATCTGAACCAATTCTGCAAGCATTAATAGTTCAGCACATCACTGAGGAACCTTCAACGCCAGCAAAGCCTCTTTCCACAGCCAATAATGTAATTCTTATAGCACACTCCACAGCCCAGAAATTACCATATGAAACACCAATAAAAATGCAGTATGTTCCCCAAACTACAAATCCACTTACAGCTACTAGCCAGTCATATACAACAGCCAAAGAAACATCTGCAGTTCCCTCCACAACAAATCAGCAAACATTAGCAGCAGCACAACAATCATTTGTAACATCCACGGCAGCACAACAGTCATCTGTAACATCCACGGCAGCAGAACAGCCATCTACAACACAACAATCAACATCAATAGTCTCTTCTACACCCAAACAAGCAACTGTAAAAACCCCAACCTTCAAACCACCACATGTAATAATAATACCTGCTACCACACCAAGGACAACATCCCCCCTGCCAACAACTGATGTTAATCGAGGTGCACCTGTTAGCTTTGTTGTTGCGGGTTCCCGGTCCCCCAGGAAAAAGAGAGAAGAAAGCCAACCCACCGATCCTGCACTGGCGTTAAGTATCCCACCCGTAATTCTCCATTTGGGACTCCCGGTCCAGGTGAAGCTAAAGCAGTGTGAGATTGGACTGGCATGGAATAGCGACTCTCATACCTATCAACTCAGCAAGATGGCAATTCTGATAGATCGCAAAGTCGGGTGTGGGCTGCGGCCTGGATTTGTGCCACAGTGTCCGAGCTGCTCTGAGGCTGAAATGGGTGAATTTCCCTGGATTGTTTCTCTCAGACTATCCATCCAACACTTTTGTGCTGGCTCCATTCTCAACCGTTGGTGGATTTTAACCACAGCCAACTGTGCCAATCTTATAAAGAATTCGGAGGCTTTGGCGCTGGTAGGGGTTGGATTGATAGATGTCACTAAGCCAACGCACTCAGTTCAGATCCGCCAGGCTCTAACACATCCCAAGTCGTTGGAACAGGGAGATCTTCACAACCTGGGACTTTTGGAACTGGAGAAACCACTGGATTTTGGGCCTCTTGTTTCACCTATTTGTATCTCAGGAAAGGCAGACATGATGGGTGATTTCAGAGACTGTTGGCTGCCAGGCTGGACCGTGCTAGAGGGAGGCCCTACTGTACTATTGAAATATCACATAGACATCTTGAACATCAGCAGCTGCAACCAACTGGAAGACAAACTCTCCAGTGCCATCTTCTGTGTCAAAGTCCAGATGGGCGAAGAAGGGATTTGCAAGGGTGATGTGGGGTCTCCACTGATCTGCCCTGACCCCAAGGGTGGGGCATGGCTTCAGCTGGGGGTGTTAAGCAGCTTTGATGAAGCCTGTTCCCGTCCCTATGTCTTCAGCAGCCTGTCTCACTACTTGCCCTGGTTGGAGAAGATCACAAAGATTGAAGGGCATCGGTACAACCTTTCAGTTCCTTGGGAACGGCTTGGCCCAGCTAGGAACCTTAGGCTGCTGCGGGAACCAGAGACTTTGGTTGGATGGATCTCAGCACACTCATCTTTGCCCTGGCAAGCGCTCGTTGCCACCTGTGCGAATGTGACCTGCGGGGGCTCCATCCTGAGCCGCTACTGGGTGCTGACTACAGCACAGTGCATGAGAGAAGCGGATCCAGAAGGTACTGCTGTTTTTGTGGGGTTGATGCACCCCAAAGGCCACATCAAGGGGGTCAGTGTGGCTGGGATTTACATCTCTAATAACTCCAGTGTAGCTCTACTGCTTCTCCAGAGACCTATCACCTTTAACAAGTACATCACTCGTATGGCTTCCTCCCCGACAGCATCTTGGGATAGCTGTAAAGTGATGGGCCTGCAGATACTACAATCTGGTGAGATTCAGGCTAACCCCAGTGCATACCAAGTAAAAGTGCTACTACCTTCAGATTGTGCCAAGGAATATCCTGGGGTCGAACCAGCCATGTTTTGTATCGTAAATGATAACTCTAGCTACCTGCCTGCTGAAGCTGTAGGTGAAGGTGCAGCCCTGCTGTGTCGTTTGGAATCAAAAAGCACAAGTTGGAGGCAGGTTGGCTTTGTGAGTGCACCCCTTCCAGGATACCAGGCGGTTATTCTGTCCTCTTGCATTTCCTCCTATGCTGACTGGATAGAAGAAACATCAAAAAGAGCCAAGCACCTAGTACATCTGTCCCATGGAAGTGGACCCTGTAGGCCAAATGTTTGGCTCCTCCTCCTAATTCTGTCCTTATTGGAAGGAACAGAGTTGGGTTGAATGAAGGAACGAGATGGCAATTCCACACTTGGAACCTTCTTTGCGGAATGCTCTTCCAACACATTCCTTTGAGTGTGCCCTCAAAGAGATTGGATGAAGGGCTCGCTCAGCATCAATGGTGGTTTGAGTGCATCGATTTTTATTTCTGCATGTGGCCACATTCTCATCTGCGAAGGTACTACAGGGCTCCTTCACAGGAAACTGCCAAGCAAAAGGCAGCCCTGACTGCCCCCAGCACTAGTCCACTGGGATTCAGCCAAGATGAGCTTTTCTATGCAATGTCATCAATCCTAAGGATCGTGAGGTGGTCTATCATGAAGACTGGGGGCTGCTGTGGGTTTTTATATCAACAGTTGCTTCAATGAATGAACTAAATACAATTATGTCAAATGAGATGAAGACTGACAGAGGAGATAAAGCTCAACCCCGAGGAAGAAGAAATGAATGAATTAATGAATGAATAAATAAATAAACCATGGCTGAAGTTCTAGAGAATTTTCCATCCATTTGCTGAAGACTTTGGATATAGTTGTGTTCTGTGGCCAGGTTCTCCCATTACAACTCCCAAGCATCTGGGTTGTGTGTAGAACACGCATGTGCAGCTGGTTCCCAATATATAAACATGCAAGATAACTTCAAACTATGAACAAATACTTATTTTTTGGGAAAACTGAATTCTCTCAAAGAGGGCCCAAGGTTGGGATTTGAGATCCCATGGAATTAGCTATAAAGGGAAGGGAAGTTTGGGAATGAAAACAAGTTGGGGTGGTGGGGAGGATTGCTTCCATGATCTCTGGATCTGTTGGTAGTCTGTTGCAGGTCAGATTTGCTCAGTCAGTTGGGGACTCGTTCTTCCAAGCAGTTGAGACCATCTCTTTTTCTCATTCCTGAAAACACGGTTGAAAGTGTGGCTGGTGGTTAGAGAAAATAAGTCTTCCCATTTTACTCCACGTCGGTTTTAGAACAAAGGAAACGCAGGTGCAGAGACATGCTGCAGACGGAAGGGCAAAGGAATACCTGGCATTCCACAGTCAGGGCCTCCGGGGCTGGAAGTTACTGTCTGTGATTTACCTTCTTTCTGAACGGTGCCATGTAATTTTAGGTTCCTTCCTCTGTACCCCGCCAACTTCCAGCTTATAGTCTTGAGTTTCAAGGCTTTGTTTTGCACACACATTTGTCTCATTTTTTGATTGTGGCTTTGTTCAGGAAACCCAAACATATACCAGGTTTGGAGAGGAAGAGATGGGAAATGGTGCCCTCACACATCAGGGTTACTTTGTGTGGCAACAGAACCTAACCGTTGAGATTGTGAAAATCCTGGCCCCATCAGGGCAAGGGCCAAGTGATGCCTCTATGTGATTCAAGTGGCGTTGTAAATAAAAGGAAAAAATTGGTGCTTCCTGACATATAGTTTGTTGTGCAATCATACTACCTCATGATTTTATAGGAAATAAACC

>Antelope, Pantholops hodgsonii

NTCCCAACATGGACTCTGGCTGAAAGACCAGCTGTAAATTCCTGGAGAGAGACTGTGTCCTTTACAGTCCTACCATGGATTCAGGATGAAAGTCCAGGTGTAATTACCTGGGGTGAGACTGTCCCATTCAGAGCCCCACCAAGAGCAGAGATTAAAAGTCAAGATGTAAACACCTGGAGAGAGACTGCTCCTTTCACATTCCCAACATGGACTCTGGCTAAAAGACCAGCTGGAAAATCCTGGAGAGTGAATGTGCCTTTCACAGCCCCACCATGGACACTGGCTGAAGGTTCATCTGTAAATACCTGGAGAGAAACTTTGCCTTTCACAGGCCCACCCTGGACTCATGATGAAGGTACAGCTATAAATAGTTGGAGAGGGACTGTGCCATTCATAGCTGCACTATGGGCTGTAGATAAAGGTTCAGCTGTAAATACCTGGAGAGAGATGATGACTTTCACAGCCCCACCATGGCCCCAGGCTGAAGTTCCAGCTGTAAGTACATGGAGAGTGACTATGCCTTTTACAGTCCCTCCATGGACTCAGGGTGAAAGTCCAGGTGTAATTACCTGGGGAGAGACTTTTCCTTTCAGAACCCGATCAAGGACACAGATAGAAAGTCCAGATATAAACACCTGGAGAGAGACTTTGCCTTTCACAGCCTCACAATGGACACAGGATGAAGCTCCAGCTGTAAATTCCTGGAGAGAGATTATGCCTTTTTCAGTCCCACCATGGACACAGGATGGAAGTCCAGGTGTAATTACCTGGGGAGAGACTCTTCCTTTCAGAACCCCATCAAGGACACAGATTGAAAGTCCAGATATAAACACCTGGAGAGAGAATCTGCCTTTCACAGTCCCATCATGGACACAGACTGAACCTCCAGCTGTAAATTCCTGGAGAGAGATTATGCCTTTTTCAGTTCCACCATGGACACAGGATGGAAGTCCAGGTGTAATTACCTGGGGAGAGACTGTCCCTTTCAGAATCCCACCAAGGACACTGATTGAAAGTCCAGATGTAAATACCTGGAGAAAGACTGTGCCTTTCACAGCACTACCATGGACACAGGCTGAAGGTCCAGATGTAAATACCCAGAGAGATACTGTGCCTTTTACAGGTCTACATTGGACTCAGGCTGAAAGTACAGCTGTGCATACTAGGAGAGATACTGTGCCTTTCACAGCTCCACCATGGACTCAAGATAAAGGCTCAGATGTAAATAACCGGAGTGAGATGCTGAGTTTCACAGGACCGTCATGGGCACAGGCTAAAGGTCCAGCTTTAAATACCTGGAGGGAGACTGTACCTTCAACAGGCTCACCTTGGACTCAGGCTGAAAATCCAACTGTAAATACCTGGAGAGAGAATATGCCTTTAACAGCCCCACCCTGGTCACAGGTTGAACATCCAGCTGTAAATACATGGAGAGAGACCGTGCCTTTCACAGAGTCACCATGGACTCAGGCTGAACATCTTGCTGTAACCACTTGGAGAGAAACTGTGTCTTTCTCAGCTCTACCATGGACTCAGGCTGAAAGTCCAGCTGTAAACACCTGGAGAGAGGCTATGCCTTTCACAGCCCCATCATGGATTCAAGATAAAAGTCCAACTGTAAATAGCTGGAGACAGATTTTTACTTTCCCAGCCCAACCATGGCCACAGACTGAAAGTACAGTGGAAAACGACTGGATATGGAATACCCCTTTTACAGCTCCACCGTGGTCACACACTGAAAGTCCAGCTGTAAATACATGGACAGAGCATATGCTTTTTATAGCCCCTCCATGGACTCAGGCTGAAAATCCAGTTACAAATAGCTGGAAAGTAAATATGCATTACAGAGACCCACTATGGCCTCAGTCTGACTTTGCACCAGCAAACCCTTGGACATCAACTGAAAGTTTCAGAATCACATCATGGACTCATACAGTAAAGCAAGTTTTAAATATTTGGACAGAGACAATAGCTTCCACTGCCACACTGTGGACTCAGGCTGAATATTCAACACCAAAATATTGGACAGAGACTAAGGCCATTTATATATTCACACCATTGACCGAGTGTCAGTTTCCAATAAATACTTTGACAGAATCTGTAGGAGCCATAAACACACTTTGGACATCTGCTGAATCTCTATCATTAAGTTCTTTCACACAGAATATTGTCGATACAATCAAATTTTGGCTAATGCTTAAAAGCGAGTCTAAGAAAAGGTGGAATCTGCCTCAAACATTCATGTTTTCAGTAAATCCTCAAATTGATACTTTTGGATCCTTGAACCAAACTGAAAATCAAGAATCTCCTCTGTGCACCCATCCTGAAATTGATAATGTCAATACAATGGCCTTTCTTGAATTTGGAACACTCATATTACAGGTAGTACCTTTGCCCCAAGCAGCTAGACTCTGGCCCCAAACTGAAGCTGATATTAGCAAAATTTGGTTTGTATCCTCTGAAAGAATAAATTCCTGGGACCAATCAGAGTCTCAAAGAATGAGTACCTCAATCCATTTTGGAGTGGATAGAGTGAAGCCCCTGGCCCAACATGAAACTGCTATAGTCATGTCATGGCTTCAGATTGAAACTGGTATATTCCACCCTTGGAACAAGTCTGAAGGAGGCACAGGGAGGTTCTGGCCCCTTTCTGAAACTGAGGATGTAAGAGAATGGATCCAAACTGGAGCCAGTATAGTTAACTCTTGGACTCGACTGAGAACTAATATAGTCAGAGCTTGGCCCCAAGCTGAATCTGAACTAGTCAGACCCTGGACACAAGCTAAAACTAATGCAATCACACTATTGACCCAAACTGATGTTATCAAACCTTGGTTCCAAACTAAAATTAATGCACTAAGAGAAGGGACCCAAACTCAATCTCAAATTTTTACTACTTGGATCCAAACACAGTTGCAAATATTTCACCCCTGGATTCAGACTAAAAGTGATTCAGTCAGATTTTGGACCCAGCCTTGGATCCAAGCTGAAACCCACACAGTCAGACTCTATTATGAAATTGATATAAGAAAATCATGGGCCTCATCTGAATCTCAGTCAGTCTCATTTTGGTCACTGAGTCAAAATTCAGTTAGGACCTCATTTCACTTTGAATCTCAGATGACATGTTCCTGGGTCCGAAATGAATTTGATATAATCAGTCCTTGGAATCAATATGAAACTAGTTCTGTTGTATCCTGGATCCAGTCTGAAACTAGTGCATGTCAACCCTCGCTCCACATTGAATCTACTACAATCACACCATGGACCCAGTATGAAACATTAGAGACCTCCCCTTCAACCCAGCCTGAGACTGATACAGCAATAAGGCATTTGTTCCAGCCCCAGATTGATCCAATTAGTACTTGGAATCAGCCTGAGGTAGATACAATCAGATTCTGGACCCAAGTTGAAACAGAAACAATTCCAATTTGGACCCAGATTGGAAGTCAAGTAGTTAAACATCCCAACTTTTCTGAAGTTGGTATAGTTACACCTTGGCTAAAGACTGAAACTGATGCAAGTAGACCCTGGATTCAGTCTGACTTTCAGTCAATCCATTCTTGGACCCAGACTGGATTTGGTATAATTGACCCCTGGTCTAAGCCAAGAGCTTCTGTAAATCAACCCTGGACCTTTGTTCAAACACAGTCAATCATACCTTGGATTAAAGTGGAATCCAATACAATCAAATCTTGGTTTCATGTTCCAGTGAAAAAAGTCAGACTGGGGATTCCTTCTGTGTCTCAAATATTGAGTTTCTGGATGAATTCTGATGTTAGTAGAGTCAATGCTTGGATCCAAACAGAAACCCAGGCAGTCAATCCTGGGGCTCATCCTAAAACTGCTAATGTTGCATCCCTGACTATTCCTAACCCTGAAAGAGTCAGAATGTGGATCCAGCCTGAAATAGAAATAAGGCCTGGCATCATTTATAAAACTAATATAACCACATCATTTGCTTCTGAAATTGAACGAGATAGAACAAGTAGTCATTTTGATTCCTGGTCTAACCATGTAACATTTTTACCAATAGAAACAGTTCCTTCCCTAGATGAGCATTTCGCAGCTTTGTCAACTGAAATAGCTGCAGTAGAAAGCCAAGCTGCTTCTGATAGTTCTCTTCAGAAACTATCTTCCTCAAAAGTTATTGAAGACACCATTCTTTCCCATACTTTTTTATCCTTTCATGCTGCTCCAGCCACTCTTTTAACAAAGCAACCATCTCTGGTGCCTGGATTTCAATTGGGAACCAAGTCTAATCAGCCTGAACAAGATCTTCCTAAGTATTCTGAACTCAATATTTCCCTTGCTGAGTGTCGCTTGGGTGTGGTCTGGAAAGAGAGTCTCCAGGCTTTCTCGCTCTTCAAGACAGCTGTTATTTCTCATGAAATCACAGGCAAAAAAAACTCAGAAGCACTGGCCCATGTCCAGGTGGGGCTTATCGATCTTCAAGACCCTGCTCAAGCTCAAACTGTAGGTATTCATCGTGCCATGCCCTACCTGGGCCCTAGAGGACCTCTGGGACCTGGTCTAATCTTCTTGAAGCAACCATTACATTTCCAACCCCTGGTTCTTCCTATCTGCCTTGAGGAGAATCTAGAGCAAGAGAAAAATATACAACTGTATGACTGCTGGCTACCCAGTTGGTCCCTCATGAGAGGAAGTCCTGGAATTTTGCAAAAAAGGCACCTGAGCATCCTGCAAGCCATCACATGTGCCCAGTTTTGGCCCAAATTGAATGAATTTACTTTCTGCGTGGCAGCCAAGAAAGCTATGGGGGAGGCTGGCTGTAAGGTGATGGATGAGTTGAAGGCACAAAAGGAAGGGAGAAAGAGAGTCTCTACCTCTATGAATGCCTCAGCTTTTACTTCCACACCTGCTTCTGTCCGGCCACATTTCATCTCTCTGCCACAGCCTCAGACTTTGGCAGATCGAATTTCTCTGAGGTATGCCATGCCTTGGCAGGCCATGATCATCAGTTGTGGCAGTCAAATTTGCAGTGGTTCCATTGTTAGCAGCTCTTGGGTACTCACTGCGGCCCACTGTGTCAGAAATATGAATCCTGAAGACACAGCTGTAATATTGGGCCTGAGGCCCCCTGGAGCACCTCTGAGAGTTGTTAAGATCTCTACCATTCTTCTGCATGAAAGATTTCGATATGTGAGTAGAGCAGCAAGAAACGATCTAGCATTGCTGCTCCTTCAAGAGGTCCAGACTCCCATTCAGATTTTAGCACCGCTAGGTCATCTGAAGAACCTGAACAGCTCAGAATGCTGGCTGTCTGGGCCACGAATTCTTAAGCCAGGAGAGACAGATGAAAATCCAGAAATATTACAGATGCGGGTGATAGGAGCTTCAAGCTGTGCCCACCTTTACCCTGATATAGGCAGTTCTATTGTGTGCTTCATTACACAAGACAAAGATGCTGACACAAATGTGGAACCAGTGAGTCCAGGCAGCGCTGTCATGTGCAGACCAATGTCTAGGAATGGAAGCTGGAGACAGATAGGCCTCACTAGTCTGAAGGCACTGGCTACCATTGTAAGCCCCCACTTCTCATGGATATTATCCACTTCATCAAAGGCAGGACATCCATTAAGCCATGCACTCATGCCTTGGATGGAAAAGCCTAAGTCCTCCAGTCTCGTAAAACAGCCAACCACCCTGCCATTTTATTCAACAATAATTGTTATACTACAAAGGCTTTCATAACTCATTGCAAAAATAAGGCATGGCTAATCTATGCAAACTATTTATAATAAAAATGTTAAACAACGTTAAAAAAAATTAAGACCCTCTGCAACCTGGGA

>Bank Vole, Myodes glareolus (BK059524)

CACACATTGCTAAATACCTTAATAAATGTTAATTCTCTCAACCTCTTTACTTATCTTCAAGTTGATTTACCTAGTTCTTGGATGGAGTCCATTGGAAATATTGAACACAGGAAAAAAACTGAACTATTTTTACTTAGGCCTTGGGTACATTCTCAAAGTCATAACATCATACATCAGACAAAACCTGAAGATAATAATGTAGCTTTGTTTCAGGCACAAACAAGAAGGATCTGGAGCCATTATGAATCTCCAGTAAACCATTACTTGTCTAAGATGAAAGATGAAAAAATGTTACCTATGCATCAATCACAAATGGATACATTCAAAACTTGGATTCAGAGAAAGTTACAGATAACACCGTCTTGGATGAAGCATGAAGATGATAAAGCTATATTTTTTACTCAAATTCAAGAAACTAATGATAAACAATCATTTCANNNNNNNNNNNNNNNCTTAAAACAGACACAGCCATTAACTCAATTATCTCAGAAATGGATATTATCTGCACCTGAACTACATAGTCTCTGGAAACCAGCTATGACTGATAAATTAAGAAACTCTGTATATCATGAATTTGATACAGTTAAGTCACTGAATGAGTTAAATGTTGGTATAATTAAACCAAGAGTATACAAGGAAGCCAGACCATTAAAACAGTGGATGCAGGCTGAGTATGTAAAAGTAAATCCACACAAATCATCTAAAGCTGACATAGTCATGGTTTGGAGTCAAGCTAAAACTCCAGTAGTAACTGACTGGTTGAATTTATTAGAAGATACAGCCACAACATCAATAAATTCTCAATTTCAAAAACTAAAGGACTTGACACAATCTAAAAATAATAGAGCCACAAGGTGGATCCAAACTGAAGCACCAATACCAAGTCCCTGGATGTTGCCTGGGGTGTATACTCTTACTTCATGGACTACAGGTGAATCATCAAACATAATCCCCTTGGCAGAACATTTAATTTATACTGTGCAATTGTGGACACAAAATAAACCAACAAAAAACTCCAGAAGTATAACTGACACACTCACTTATTGGTCCCAAACAAAAATATCAGCAACAAGTCTCAGAAGGAAACCTCTAAGAAGTAAAACCAAGCCAAGGCCAACGACCCAGCCTAATGGAAGAAATAGCTTGGGACTTTCTGAATCTTATTTAACTTCATTGTGGACTAAAGATCATTTTCCAACATTAAATTATTCAGTAGAGTTTCAGAGTAAAATAGGTACAGTTTGGGTCCCAGATGCATATTCTACACCAAATCTATGGAAACAAATTGAAGTAGATAGGACACAATTGACTTATACTGAATTGCCACTGGAAACTTTGTGGACACATCAAGTACCTGATACAGCAATTTTTAATTTTACTAATTCTCCATCAATTAATTTGATGAATCCTGACTATGATGTAATAACAACATGGTCCAAGGCTAAAACACAATCAATAAATCCTTGGAATCCATCAGGTACAGTCACACCCTGGACTGTGTATGGAGCTTTAGTAAACAATGTGTGGTCACAAACTAAATCTGATATGAACAGTCCACAGTTTCAGAATGCGTTTACTGAAAGAAGTCTATGGACTCGGGCTAAAGTTATCTCAGAACTAGCAATTCAAACTGAGTCTCCAACAACACATCCATGGACACAGCATGGATCTGATAGAGTCACTTTATGGAATCAACCTATATATCAAACATGGAGTCAAGGGACAGGAAGTATGCCTCCCACAGAAACAATGTGGCTGAATAGTAATTCTCCATTTGTAAATTTATGGCTTCAACAAATATCTGATAGACTTATACTGTCATGGCCCCATGTTCAATCCCCATCATTAAACCTGCAGTCACAGTCTGTAACTTATACAGCCGTATCACAGTTGATTCAAGCTGAATATGCACCATTAAGTTTGTGGAGAAAACCTGAAACTGATGTAATCATAGCACCATGGCCTCAACCTGAATTATCAAATCAGGAAACTCAGACTTTGTCTGGTACAAAATTACAGCCATGGTCTGAGCCTGAATTGATACCAAAGTATCAATGGACAATAACAGTAACTCATAGACTCATACCACAATGGTCCCAGGATTTGTCTTCTACAATAAATATGAGGTCACCACTAATATTTGATACACTCCTAATATCTACAACACCATGGCCACTGAAGCAAACACTGGAAACAAACCTAAGAACACTGCCTGTATATGAGAAAAAGACAACAGCTGGTTTCCAGAGTGAATATTCAATATCAAATACATTAATATTGTCTATGACCAATAAAGTCATATTACATTGGTCCCATTCTAAGGTTCTACCAGCTTATCTATGGAGATGGCCTATATCTGATACAATGATGCAATCCTGGACCCAAGATGAATATTCTGTAAAAAATCAATGGTCAGAGTTTTTGTTTAATGCAATTACATCATCATTGTCTTTGGATAATTACCCAGCAACAATGCTGTCTAGACAACTTATATCTGATGCATTTGTCCTGTCAAGAATACAGGTTGTATCTCCCAAAGTAAATCTATTTGGACTCCGGTTACCTGATTCAATCATACTTTCTTGGTCCCAATCTATCTACTCATCAGTAAATAGTGTCGGATACTCTGGACCTGATAAAATTGTACCATTGTGGCCAAAGGTTAAATATTCACAAACAAATAAGAAGACATCATCTGTATCTGATATAATTATGCCACATTTGTTTCATGAAGTAACTTGGGAAGGAAACAGATGGCCATGGAATTACCATGATAGAGTGACACCAACTTGGACACAGATAGGATCCCCAAAATTAAGTCTGTGGTCACTGATAGATCCTAATAGAGAGTCTTCAAATGTATCTCCTTCAAGAAATATATTATCTAGTCATTCAGCCATTATAATTGCAAAATCATGGCCCCATACCAGGTCTCCAATATTTAGTATATGGTTACTTGGGGTATCTGTTACTATTTCACTATTACATCAGACTGAGTCTACAGCTGGAAATATTTGGTCATTTCCATTATCTGATACAATCACAACAATGTTGACTCATTCTGGATCTCTGCCAGTGCACCTATGGACAGTGCTTCCATCTAATAATATTCCTTCCACATGGCCTCATCCTGAACCAGCAAAACTATGGACATTACCTATATTTAATAGAAACAAACCAGCTTTGTTCCAGACTGAATCTAAAAAACTAAATACACAAGCACTGTTTGTGTCCAATACATACATACAGTCATGGGACCTATCTAAATACCAAACACTCATTCCTGGGACAAGGATAATTGCTCATGTATTATCACGATGGACCACGTCTGGAATTCTAATTGTAAATTCTTGGACCGAGCCAGCATCTTACACAACTCAGAACTATGTTCTAACCCCAGAAGTCAAACACTGGACTCACCTAATATCTGATCCTGTTTTATTAAGGAACCAAGCCACATCACCTACAACAAAGTTTTGGAAAAAATATGGAGCTGATGCATTCACTGAGTGGGACATTTCACAATTCACAAATATAAAACATCCAACACAGTCTTTTCCTAATATACTCAAGCATGTAGAAATTTCAACAACAGATCCCTTGACAAAGGTTGAAACAGATATCTCACAGATGTTCCAACCTAATTCTAGTAACAGAAGTATCTGGTCACAAATTTTAGAAAATACAGTAACATCATTGCTTCAGAGTGTTTCTCTAATATCTATTCCTTGGACAAAATTTCAATCTAATACAATCAGAATGAAGAATCAGTCAGAGTCTTCAGTATTAACTTATTGGACACAGTCTATTTCTGATCAAGTTTTACCATGGTCTCAAACCCTGTCAGAAGCAGAAAACCCAACACTTGTTATGAGAGGTACAGAAACACCATGGAAATTTCCCAAAGTTGTGTTACTAAAATACTGGAATCAATCTACAACAAATATTATCTCTCAATGGATAACAGACAAGTCTTCTGAAATTCATGTATTCCCAACAGTTGTATTTGATAAAGTCATTCTTTTTGATCATGCAGTAAATCCATTATTAAGTCAATGGACAGAGACTGAAGCTGATGCAGTCAGAGAAGCCAAGTATTTTAACAAATATGTCCTGACAAATTCTCACCATAAAATAGTCACAAAATGGGGCCCAAGTGAATTTTCAGATAAAATGATCTGGACAGCCCCTGAAACTGGCACAGCTGTGGGATGGCAACAGGGCAAATCTACATCTTTTAGTCCAAGAACGGAATATATAGCTAAAGTAGTAAAAGTGAGAATGCAAACTGAATCCTCAGCCATAAATCATTTTGCAAAATTAGTAGTCAGTACACTGACAACACAGACTCAAGGAGAACTCACAACAAAAAATATTGATATGCAACATGTATTTGATAAAATCACATTTATAGCAGAACCTAAATATCCATTACCACACAGTTGGATCCAGAAAGAAAATGTACCATTCAATACAAAAGAAACGTCAGAAAATCATTGGGTGCAACCTGCAACTGAAACTGTCAGATTGTGCATATACACAGTGTATTCAACACTTAATTACTTGCTACAGACACAAAAATTAGTTACAGGATGGTTTCAAGCTGATCCCCAGAAAAGAAAAAATCACTGGGATAAGGCTAAATTATCAAATATCATACATTTTACGCAAAATGAATATCTATCTACATATCCATGTATAAATGATAAAATATACAAATTCATGCCTGGGACTCAAATTCAATACATACCAGAATATCACTGGTCAGAAGCTACAGATTCAAAAGTAGTGTCTTGGAGTGAGACTGACTCATTCCTAACAAATATATGGAAAGAGGCCATGTTTTCAAAAGTCATATACTGGAACCAATTTCACTCTCTATCAGTTAATCCATCAACAAGAAAAAGGGCTTCAGTACTCATTCAGAGGGAATTTCCAACAGAAGATACCTGGATAGACAGTTCTACTCCAAATGACATTCATTGGATTCAGGAAGAATCTTCATTAGAACAGTCCGAGACAGTGTCTCTAACTTCAATTGCTATTAATTGGAGACATTTTGAGCTTATACCAGTAGTTCCCTGGACAAAATCTATAGATCTTAGATATCCACTAACAAACAAATTGATGTTGTCTTCATATAATCATATCATTTGGACCCATCCCAAATCCTTACATGTAAATATACCAATGAGTCCTCTTTCTAAAACACTGACACCATTGGCATTGGATGAAAATGATTTAAAAAATCACTGGACAGAGTCTGAGTTTGAAAATCTCATAACATTGACCAAGGTTTTATCTGTATCAGAAAAGCTATTGACAGAAGATCTTTTTTATACACTTTCACCCTGGACAAAATTTTTATTTTCATCAGAAATTACTTCCATGCACTCTAATTTTGATGCAATCACACCAAGGGCCTTGGCAAAAATTTCAGCAGTAATTTCAACATGCATATTGTCTGATAATCATATATGTATATCTTGGAACCATAATGAGTTTCTAAAATCTAAACTAAAAATGTCTACTAGTTCAATAGTAAAACAGAACTTCCAAAGTGAATCTCCTTTAGATCTTTTGGAACCAAAAGTAAGTTCTAGATTTACAGCATGGAGACTAGATGAATTGTTACTACAAACTGATGTTACAATTATACTATCCACAACTGTTGCACAGACCCAAACGGAGTCTCTGGGAAGTGAAATATGGCCAGAAATTGTAACAAACAATAAGACATCATTGAACTCAACTGAATTTACACCCATATATTTTTATATGCACAACAATATTGATAAAAGCACAATTTTAACAGTGGTTGAAATTGAGGGGAAAAATGTTTTAACTGTGTCTACTATCAATATTTTTAGAATTTCAATGCAAGCACAACCTTATGCTACCAAACAATCAAGTCAAACTGCACAGACATTGGTTCCATTGGTCTCTACAGAAACTGTAGACAGAAATACATGGTCTTTTTCTGAATTAGGATCTCTCTTATCTTGGAGTGTGCCTGTTCCTTCAGCAGATATTCTCTTGTATCATACTGAATATCACATCCGTGGAAGTTGGTTTAAAACTAGTGTTGAAAGAAAAAGACCTTGGGTACAGCCATATTATCAAACAATCAGTATCTATACTTCATTAATGATTAGCACAGTAGAATCCTGTGTCCAACATAAAATGGGCAGAAAATCATGGATGAATTCTGCAACTGGACTGTTTAATGACAGAACCCAGTGTGAAACAGAAAATGTAAGACTTGGGAATGTTCTTAAAAATAATACAGTAAGGCCATGGTTCCATGAAGAAGCTACCACAGACCATACCTGGTTCCATTCGATAACTAATACAGTAATAGTATTTACCCAGACTGAATATCAAGAAATTGGACTTTGGACTGTGTCTGTCTCTGATATTCTATCATCAAAAGCTGAAATAGAACCAGAGACATCCTTGTACATTTATGACAATAATGCTGTCTCATATTGGTTCCCAATTCCAAATAGTTTAAGTCAAAGAATTAAAATCGAGCCTGGAACTGTTACCCCCTGGATTCAGAAAGAATGGCAAATAGTTCACCGATGGAATAGACTTGAAACAAAAGTGGTGAAAAGTGGGTACAATTTTGAAGCAAGTACATCTCAAATAATTTTGACCTATTCTAAATCTGGTGAAATTATACCTTGGACCCAACCTGAATCTCCACTGGTTAGGAGATGGCCTGAAGATGCTATAGTCACACTTTGGACACTGACTAAAAGTGATGAAAATATGCCATTGTCACAACTGTTACAGCATAATTTTGGTGGAATTAAACCTTGGATTCAGCATATAGCTCTTACTGAAACTCTGGAAACCATTCTCTGGACTCATTCTGATACAGTACTATCAAATGCATTGCAGACTGATGTAGATGACATACTTTGGTCATTGACTCAAGGGGATGCAATCAGGCAGTTGTCACAAGTGGAATATGAAGCTAAACAATCTTTGACAGGGCCTGATATTGATGTAATCATCCCTTGGATTCAGCATAAAGATTCTTCTGAAACTGTAGAAGTTATTCCCTGGACTCATTCTGATCCAGTGATATCAGACTCAATGCAAACTCAAATAGATTTTTGGTCCCAGAACCAAATTGATTCTATCAGGCAGTGGTCACAAGTAGAATATAAAGCTAAACAGTCTTTGGCACAGCCTAATGTTGGTATTTTCAACCCTAGCATTCAATATAAAGCGTCACCCGAATCTCTAGAAATCATTCCCTGGACTCATTCTGATATAATATCACACATATTACAGACTCAAATAGATTCCATACTGTGGACACTGACTCAAACTGATTTTGATAAACTATGGTCACAAGTGGAATATGAAAATGTACAATCTTCAGTACATCAGAATGTTGGCTTAATTCACTCATGGATTCAGCATAAAGATTCTACTGAAACTGTAGATATCTTTTCCTGGACTTATTCTCATAGAGTAATATCATACTCATTACAGACTCAAATAGATTTACTTAGATTATGGAACAAGTCTCAACCGATGACAGCCCGAATTTGGAATCCAACTTCAGAACAAATAGTAAAACCAGATGCCCTGACCGCAGTTTTTACAATTTCACCATCATTTCAGGTTCAAGAAATGCTTTTAAGTGAAGAACCTACCCCGATATTGCCACCTCAATCTGATCTACAAGATAAGCATTCACTGGAACCATTATCAACAACAGAATCAAAACTCAACATTTCCTTGTCTGAATGTCACCTGAGTATGATATGGAATGATAATCTCCAAACACTGTGGCTCTTCAAGACAGCTGTTGTTTCTCATGACTCCACAGAATGTGGTATACGCCCTGGCCTTGTTCCCCACTGTCCCGACTGTTGGGAAGCTGAAATAGGTGAATTCCCATGGATGGTTTCTGTGCAGCTCTCTTACTCCCACTTCTGTGCTGGTTCTATTCTTAATGAAATGTGGGTCCTCACTTCTGCCCGATGTGCCAATTTTATAAAACGGTCAGAATCTCTGGCTTTAGTCCAAGTGGGTCTGATTGATCTTCAGGATCCTACACAAGGTGAAACTGTTGGTATCCAGCGTTCTGTGCCATACTTAGGTCCCTGGGGACCTTTAGGACCAGGCCTATTCTTCCTGAAGGAGCCAATACATTTTCAACCTTTGGTGCTTCCTATTTGCCTGGAAGAAAGTCAAGAACAAGAAAGACACATACAACTATATGACTGCTGGTTACCTAGCTGGTCCCTCATGAGAGGAAGTCCTGGTATCTTGCAAAAAAGGCACCTCAGCATCATGCAAGTCAGCACCTGTTCTAAATTTTGGCCTCAACTGAATGAGTTCACTTTCTGTATAAAGGCCAAGAAAGCTATGGGGGAATCTGGCTGTAAGGGTGATCTTGGGGCACCTTTGGTTTGTCATCTAAAACAAAAAGACACATGGATGCAGGTGGGAATTTTAATTCAATTTGATGAACATTGCACAAAGCCCTATGTCTTCAGTCAAGTTAGACCTTTCGTTTTCTGGCTCCAGAGAGTTACACAGCCCAGTCATGCTCCCTGGTCTAATCAAAGAACTGTGATTACCTCTCTTTCCAATACCCTGTCAGTATCTAACAGGAAAGATTTAACTTTTACGTCACCTTCTGCTACTGTTCATTCACACTTCATCTCTCTGCCACACCCTCAAGCTTTAGCAGATCATATTTCTGTGCAATATTCTATGCCATGGCAAGCTATAATCTTCGGTTGTGGCAATCAGATCTGCAGTGGCTCCATTATTAGTAGCTACTGGGTTCTTACTGCTGCCCACTGTGTGAGAAACATGAATCCTGAAGACACTCTTGTGATATTGGGCCTTAGAAATCCCAGAAAACCTCTGAGAGTTGTTAAGGTGACTACTATTTTACTGCATGAAAGATTCCGGTTAGTAACTCAGGCTGCAAGAAATGACCTAGCTCTGGTGCTTCTTCGAGAAGGCCAGAGTTCTATTCATATAATAGCACCCTTAGGCAGCATGAAGAATTTGAACATTTCAGAATGCTGGCTTTCTGGACCCCAAATTCTTAAACAAGGATATGTATTTGAAAATCTAGATATGTTACAGATACAAGTGATGGGAGCATCCAAATGTGCCTATCTCTATCCAGACATAGGAAGTTCTACGGTTTGCTACAATCCACAAGCTGGTGTTCCTGTAATAAGCATAGAGTCAGTGAGTCCTGGAAGTGCTGTTATATGCAGACCACTATATGGAAATCGCAAATGGAGACAAATAGGTTTCACTAGTCTCAAGAATCTAGCTACCATAGTTGCTCCACACTTTTCCTGGATCTTGTCCTCAACAGCAAAGTCAGGCTATCCCTTAGATCCGGCATTCATTCCTTGGGTAGAAAATTCCAATTCCTCTAGACTTGTGAAACATCCAACCACATTGCTACTTTCCATCATAATGATTATTTCAGCGAAAAGGGTTTTTATTTTGTAATGCACTGATTACATCACTGTGTTCAAACCTGCTGAACAAATATACCAGAAAATTTAGAAAAAAGTTCTGACTTAATGTTTTATAGAACCTCCCAAAATATTGTATTAATAAA

>Bearded Dragon, Pogona vitticeps (BK059518)

GTTCCATCCCAGACGCCTTCAGCACTGACTTTATGCTCTGTTCCAGTGGGGCCTGTGGGTTAGCCTGTGTCATCTCATCTCGTCTATGATGCCCCTTCATGACTCTTTGGCCTCCCTCTGCCACAGAGAGGAAGGATGGAGCTTCTCCTTGGGCTCTGTGCTCTTTTCCAACTATTTATCGTCTCCTCCGGCCTCTGTGGCTTTCGTCACCTGCTGCCCAAGCTGGCTGAAGCCATGCCAAAAAAGGGTGCAACTGTGGAAGCACCGTGGTTAGTGAACATCTATGGCAATGGCCAGAGGTGCCAAGGAGTGGTCCTGAGCAGCTGGTGGGTGCTGACCGCAGCCAACTGCTTCCTGTCAATGTCGCCGAGCCATGTAGAATTGACCGGGGCTCATGGGAGTATTAACACTCAGTCGGTGAGCCAGTTCATGCCACACAAAGGCTTCAGTACCTGGGATACAGAGCCTAACAATGACCTGGGACTCCTTCTGCTTAGCCAGCCACTTGATTTAAGAACGAAAGATATGTGGCCAGCTTGTGTCCCCAAAGAAGAAAAAGCTGACAACACACAAGAGGAGTGTAGGATTTTTGAACGAAGCCAGGACGAATCGACACTGAAAGAAATTGAAGTGGAGGCTTTGATGACATCAAAATGTGTCAAACGCTGGCCTGGAACAACAGAAAAACTGAACTTGTGTGTTGCAAGAAAGAACTCTGACCAAACTGATTGCAGGGTGCCCATAGGCAGTCCTGTGATCTGTCATAACCCGGACAACAAGCACTGGGAAGTAATGGGCATGGTGAGTCGGAGCTTGCGTAAGTGCACTGCTCCTATCTTGGCTTCCAAAGTTTTAACACATCTGGAGTGGTTGAAGAAAGAAAAAGCAGTAGAGGATCCTCTCAATCCACGACTTGATGATAACAACCAGTCATCTGCTCCAGAAGAAGAAGAATTGTCTCCAACCATTGGACAAGCTGTAACATTCCCAACAGGATTGATCTATAAACCAAGATCCCAGGAACCTTCAGCAGCTTTAGAAGTGCCAGTATCACTCCCAGATGCAGAACCAACACCTGTACATCCCAGAAAAGCGACAAAGCCTTCCTCGACTGCCAATAATGTTATTTTGGTAGCATCTCCAACCATCCAGCGATCATCACGCATTGCTCCCACAAGAACCAAAACATCAAGGCGAAAAACATCAGCTTCAGTACCCTCCACATCTGAACAGCAGTCAATTGTACAGTCCACAGAAACACAGCAGTCATCACCTATAGTCTCTTCCACAGCCGAAGAAACATCTGTAGCACCCACAACAGTCGGAAAATCTACAGCAGAACAACAGACATCAGCTGTTGCCTCTTCAACAAACAACCAAACACCTACAACATCTACAACACTTCTAACATCTACAACATCACAACAGACCTCCACAACAGAACAGCAGCCATCCACAACACAGCAGCCATCAATAGTCTCTTCCACAACAGAACAGCAGCCATCCACAACACAGCAGCCATCAATAGTCTCTTCCACAACTAAACAAACAACGGCAGGACCAACAACAAAACGCCCACCCCAAACTTCCCCAACATTCAGACCACCGCATATAATAATAATACCTGCTACCACCCCAAAGCCTTTGCCACCTCCAGATCATGTTCATTACATTGTTGTAGATCCTCAGTTCCCCAAGAACAAACGTGAAGAAAGCCCATCTCCTGACCCTTCTGTAGCATTACGTGGCCCATCTAGTATGGAGGTGTCCTTTATTCCCCACTTTGGACTCCCTGTGCAGGTGAAGCTCCACCAGTGTGAGATGGGATTGGCCTGGAATAGCAACTCCCATACATACCAGCTTAACAAGATGGCAGTTCTGATAGATCGCAAAATTGGATGTGGGCTGCGGCCTGGTTTTGTGCCGAAATGCCCCAGCTGCTCTGAGGCAGAGATGGGTGAGTTCCCCTGGATTGTTTCCCTAAGACTGTCCATCCAACACTTCTGTGCTGGCTCCATTCTCAACCCTTGGTGGATTTTAACCACAGCCAACTGTGCCAACTTGATAAAGAATTCAGAGACTTTGGCGCTGGTGCAGGCTGGGCAGGTAGATGCCTCCAAGGCAAGCTACTCCGTCCAGATCCGCCAAGCTCTGACTCACCCTGGTTCACTGGAGCAGCAGGATCTTCACAACTTGGGGCTTTTGGAACTGGAGGAACCACTGGAATTTGGGCCCCTTGTTGGACCCATCTGTCTCTTAGACAAGGCGGATACAATGGCTAATTTCAGAGACTGTTGGCTGCCAGGCTGGACTATGCTGGATGGAGGTCCTACTGTGCTGTTGAAGCATCATTTAGACATCCTGAACATCAGCAAGTGCAGCCAGCTTGGGGACCAGCTCCCCAATGCTACCTTTTGCATCAATGCCCAGGTGGGCCAGGAAGGAGTCTGCAAGGGTGATGTGGGCTCTCCACTGATCTGTTCTGACCCCAAGGGTGGGGCATGGCTTCAGCTGGGGGTATTAAGCAGCTTTGATGAAGCTTGCTCCCATCCCTATGTGTTCAGCAGCCTGCCCTTCTACTTGCCCTGGCTGAAGAGGGCCACAAAGGCTGCTGGGCATCGTTACAACCTCTTTGTCCCTTGGGAACGGCTGGGTGCTGCAAATAATCTTCGTCTGCTAAGGCAACCAGAGACTATGATTGCACAGATCTCTGCACAGTTGTCCATGCCCTGGCAAGTACTCATTGCCACATGTGAGAATCAGAGCTGTGGAGGCTCCATCCTGAACCGTTACTGGGTACTGACAACAGCGCAGTGTGTTCGGGAGGCGGACCCAGGAAGCACAGCTGTCTTTGTGGGGTTGACCCACCCCAAGGGCTATGTCAAGGGCATCCCTGTGGCTGGGATTTATCCCTATGAGAATGGCTCCTCACAGTACAGTCTCTCCGATGACTACAGCTTGGCTCTGCTGCTCCTCCAGAAACCCATCACCTTTGGCAAACACATCACTCGTATGACCTTCACCCCAAAGGAGTCCTGGGATAGCTGCAAAGTGATGGGTTTGCAAATGCTACAGCCTGGAGAAGTTCGATTCAACCCCAGTGCATACCAAGTCAAGGTGCTGATACCCTCAGACTGTGCCAAAGAACATCCTGGAGTCAACCCAGGCGTGTATTGTGTTGTGAGGGATAACTCGAGCTACCTCCCTGCGGGAGCTGTAGGTGAGGGCGCGGCCTTGCTGTGCCACTTGGAAACCAAAAGCACCAAGTGGAGCCAGGTTGGTTTGGTGAGTGAGCCATTTCCAGGATCCCAGACCGTCGTTCTGTCCTCCAGCATTGCCTCCTATGTGGACTGGATAGAAAAAACATCCAAGCAAGCCAAGCATCTCTTCGTCATGCCCCACACAAGCGCAGCCCGTAAACCAAGTAGCTGGTTACTCCTCCTGCTTCTGTCTTTGTTTGGTGGAGCAGAGCTGGGCTGAACAGAGAAGCAAAGAAGATGGCGACATCACCCCAAAACCTTCTACATAGAACGCTGGC

>Beaver, Castor fiber (BK059501)

cacactaaagaCACAGACTAAATCTCCATTAATAAGTACTTTGAACCAAGAAGAAACTATAACTGTCAAATCCAAGATACAGACTGAATCTCCAAAAAGTAAATATCCAATCAGAATTTAAGACTCCATTTATATCTCCTTGGGTTGAGTCTGAGCCTGGGGAAGGCACACCATGCATTCATGTGGAAGATCAATGGAGAGATCCTGTAACGGAAATAGTCAAATTGTGCAGACAAGTTATTTCTACAACATTTAATTCCTTCTTAAGGAGGAACATTTACACCCTGGAACCAGTCTGAAACTCAACCAGCCACACATGGACCAAGCCTCAAGTTAATATAACATTCTTTTCTCTTCCTGTATCTGATAAATTCAGAACGTGGATCCAACCTAAAACAAAACTACTGCATTACAAAGCTGACATAATTGTATCACTGATTTCTCCTGAGACTGGAACGATTGGAAAAACTCTGCTAATTGATCATTTGGATAACAAGTCTAAGCTTGTAACATTTTTACCTGTTGAGACTATTTCTACCGCACATCAGTATTTTATAATTTTGTTAACAGAGATAAGTACCATAGAAAGAAAAGTTAAAAGCAGTTATCTCCAGCCAAGCCAGCTCACAAGCATTTTCCTTCTTACCCTGTCAAGCAAGTGGTTTCCTAGTATAATTGGTCACAAGAATTTTGGCAGCATATTAGAAATTATTGACACAAAAGGAAGCCTTGATGTCCTTTCTGTCTCTCTTAGTTATCTTTCCCCAGGCTTTTCCTTCCTTGTTTCTTGTTCTCTTACATATCCATGTACATTGTTCCCTCCCTGTTTAGTCTTTTCTTCTTGTCCTTATCTTTCACACTGTGTTTTCCCATCTTGCTTCAACTTTTCTTCTCTGGCCTTCTCTCCTGTGCTTTTGCCCTCAACCTCTTCTGATAGTCAGCTCCAGGAACTGTCTTTCTCAAAGTTTATTGAAGATACTATTTTTTCTCACACTTTCTCATCCCTGCATGCTCCTCCAGCAATAAGTTTAACAAAAGAGTCTCCCCTGATGCCTGGATATCTATCTGGACCCAACTATAAGCATCAGTCTGGACAACAGCCTCTCAATATTTTCCTGGCTGACTGTCGCCTAGGTATGATTTGGAAAGACAATCTCCAGGCTCTCTGGCTCTTCAAGACAGCTGCTGTTTCTCATGAGACCACAGAGTGTGGAGTACACCCTGGCCTTGTCTCCCATTGTCCCAATTGCTGGGAGGCAGATACAGGTGAATTTCCTTGGATGGTTTCTGTGCGTCTCTCTTTCTCCCATTTTTGTGCTGGCTCTATTTTGAATGAACAGTGGATTCTTACCTCAGCAACATGTGCCAATTTCATATAAGTCTGGAAAAAGCTCAGAAGCTCGGGCCTTGGTCCAAGTGGGTCTTATTGATCTACAGGATCCTACCCAAGCTCAGACTGTTGGTATTCATCGTGCCCTGCCCTACTTAGGACCCAAAGGACCTCTTGGACCTGGGCTAATCTTTCTGCAGCAGCCACTACATTTTCAACCCCTGGTTCTTCCTATTTGTCTGGAGGAAAGTTTGGAGCAAGAGAAGAATATACAACTGTATGACTGCTGGTTACCCAGCTGGTCCCTCATGAGAGGAAGTCCTGGAATTTTGCAAAAAAGGCACCTAAACATCCTGCAAGCCAGCACTTGTGCCCAGTTTTGGCCCAAGCTGAATGAATTTACTTTCTGTGTGGAGGCCAAGAAAGCTATGGGGGAGGCTGGCTGTAAGGGTGACTTGGGGGCACCTTTGGTGTGCCATATGCAACAAAAGGATACATGGGTACAGGTGGGAATCTTGAGTCACTTTGATGAACATTGCACAAAGCCCTATGTCTTCAGCCAAGTGAGCCCTTTCATTTTCTGGATCCAGGGAGTTACACGGCTCAGCCATGCACCATGGTCCCAACAAGGACCCATAACTACCTCTGCTTCCATCTCCCTTTCAGTCTCTCCTGCTAGGAATGCTTCAGTTTTAACCTCTACAACTGCTTCTATTCGACCCCACTTCATTTCTCTGCCACAACCTCAGACTTTGGCAGATCGTATTTCTCTGCGATATACTTTGCCTTGGCAGGCTATGATCATCAGTTGTGGCAGTCAAATCTGCAGTGGCTCCATAATTAGCAGCTCTTGGGTTCTCACTGCTGCCCATTGTGTCAGGAACATGAATCCAGAAGACACTATAGTGATACTGGGCCTTAGGCATCCTGGGGCACCTCTGAGAGTTGTTAAAGTAACTACTATCCTACTTCATGAGAGATTCCAGTTGGTGAGTGGAGCAGCAAGAAATGATCTAGCTTTGGTACTCCTTCAAGAGGGCCAAAATTCCATTCAGATGTTGGCACCATTGGGGCACTTGAAGTATCTAAATAGCTCAGAATGTTGGCTTTCTGGGCCAAGAATTCTTAAACCAGGAGAGACAGATGAGAACCCAGAAATATTACAGATGCAGGTTATGGGAGCTTCAAGCTGTGCCTACCTCTACCTAGACATAGGCAGTTCTATTGTTTGCTTCATTACTCAGGCCAAAGGCTATGACACAAATATGGAGCCAGTGAGTCCAGGCAGTGCTGTTATGTGCAGACCAATATCTGGCAATGGCAAATGGAGACAGATAGGCTTCACCAGTCTCAAATCTCTAGCTACCATAGTGAGCCCACACTTCTCCTGGATTTTAACCACTTTAGCAAAAGCAGGCCATCCCCTAAACCAGGACCTTATGCCTTGGATAGAAAAACCTAAGTCCTCTGGTTTCCTTAAATATCCATCCACACTGCTACTTTCCTCAGTAATTATATTTGAAGCACAGATGTTTTTGTAGCCTAGTGACTAGAACAGGTAGTATACATCTGTTCATACTATGATAGCCAAGATACCTGAAGAATAAAGAGAAAATATTGATTTATGCCCTataagctattcc

>Bull, Bos taurus (KC120771, BK059506)

AGAGACTGCCCCTTTCACAGTCCCAACATGGACTCTGGCTAAAAGACCAGCTGTAAAATCCTGGAGAGAGAATGTGCCTTTCACAACTCCACCATGGACATTGGCTGAAGGTCCACCTGTAAATACCTGGAGAGAAACTCTGCCTTTCATAGGCCCACCTTGGACTCATGCTGAAAGTACAGCTATAAATAGTTGGAGAGGGACTGTGCCATTTGCAACTGCACTATGGGCTCTAGATAAAGGTCCAGCTGTAAATACCTGGAGAGAGATGATGACTTTCACAGCCCCACCATGGACACAGGCTGAAGGTCCAGCTGTAAATACATGGAGAGAGACTATTCCTTTTACAGTCCCACCATGGACTCAGGATGAAAGTCCAGGTGTAATTACCTGGGGAGCAACTGTCCCTTTCAGAGTCCCATCAAGGACACAGATTGAAAGTCCAGATATAAACACCTGGAGAGAGACTGCACCTTTCACAGCCCCAGCATGGGCACAGGCTGAACCTCCAGCTGTAAATTCCTGGAGAGAGATTATGCCTTTTTCAGTCCCACCATGGACACAGGATGAAAATCCAGGTGTAATTACCTGGGGAGAGACTGTCACTTTCAGAGCCCCACCAAGGACACAGATTGAAAGTCCAGATGTAAACACCTGGAGAGAGACTGTGCCTTTCACAGCACTACCATGGACACAGGCTGAAGGTCCAGATGTAAATACCCAAAGAGATACTGTGCCTTTCACAGGTCTACCTTGGACTCAGGCTGAAAGTACAGCTGTGCATACTGGGAGAGATATTGTACCTTTCACAGCTCCACCATGGACTCAAGATAAAGGCCCAGCTTTAAATACCCGGACAGAGACTGTACCTTTAACAGGCTCACCTTGGACTCAGGCTGAAAATCCAACTGTAAATACCTGGAGAGATAAGATGCCTTTAACAGCCCCACCCTGGTCACAGGTTGAACGTCCAGCTGTAAATACATGGAGAGAGACTGTGCCTTTCACAGCACCACCATGGACTCAGGCTGAACGTCCTGCTATAAATAGCTGGAGAGAGACTATGCCTTTCTCAGCTCTACCATGGACTCAGGCTGAAAGTCCAGCTGTAAACACCTGGAGAGACACTATGCCTTTCACAGACCCATCATGGACTCAAGATAAAAGTCCAGCTGTAAATAGCTGGAGACAGATTTTGACTTTCCCAGCCCAACCTTGGCCACAGGCTGAAAGTATAGCTGCAAATGACTGGACACGGAACGCCCCTTTTACAGCTCCACTGTGGTCACACACTGAAAGTCCAGCTGTAAATACCTGGACAGAGGCTATGCTTTTCACAGGCCCTCCATGGACTCAGGCTGAAAATCCAGCTACACATACCTGGAAAGTGAACGTGCATTACAGAGGCCCACCATGGACTCAGTCTGACTCTGCACAAGCAAACCCTTGGACATCAACTGAAAGTTTTAGAATCAGATCATGGACTCATGGAGTAAAGCAAGTTTTGAATATTTGGACAGAGCCAATAGCTTCCACAGTTACACTTCGGACTCAGGCTGAATATTCAACACTAAAATATTGGGCAGAGACTAAAGTCATTTACATAGTCACACCATTGACCCAGTGTCAGTTTCCAATAAATACTTTGACAGAATCTGTAGGATCCATAATCACACCTTGGACATCTGCTGAATCTCTAGTATTAAGTTCTTTCACACAGAATATTATTGATATAATCAAATTTTGGCCAGTGCTTAAAACTGAGTCTAAGAAAAGGTGGAATCTGCCTCAAACTGATACACTCATATTTTCACTAAATCCTCAAACTGATACTTTTGGATCCTTGAACCAAATTGAAAATCAAGAATCTCCTCTGTGGACACATCCTGAAATTGATAATGTCAATACAATGAACTTTCTTGAATCTGGAACACTCATATCACAGGTAGTATCTCTGCCCCAAGCAGCTAGACTCTGGCCCCAAACTGAAGCTGATATTAGCAAAACTTGGTTTGTATCCTCTGAAAGAATAAATTCTTGGGACCAATCAGAGTCTCAAAGAATGAGTACCTCAACCCATTTTGGAGTGGGTAGAGTAAAGCCCTTGGCCCAACATGAAACTGCTATAGTCATGTCATGGCTTCAGATTGAAACTGGTATATTCTACCCTTGGAACCAGTCTGAGGGAGACACAGTGAGGTTCTGGCCCCTTTCTGAAACTGAGGATGTAAGAGAATGGATCCAAACTGGAGCCAGTAGAGTTAACTCTTGGACTCAACCGAGAACTAGTATAGTCAGAGCTTGGCCCCAAGCTGAATCTGAACTAGTCAGACCCTGGACACAAACTAAAACTAATGCAATCACACTATTGACCCAGGCTGATACTATCAAACCTTGGTTCCAAACTCAAATTAATGCAATAAGAGAAGGAGCCCAAACTCAATCTCAAATTGTTACTAGTATCCAAACACAGTTGCAAATAGTTAACCCCTGGATTCAGCCTAAAAGTGATTCAATCAGATTTTGGACCCAGCCTTGGATCCAAGCTGAAACCCACACAGTCAGACTCTTTTATGAAATTGATATAAGAAAATCATGGGCCTCATTTGGATCTCAGTCAGTCACATTTTGGTCACTGAGTCAAAATTCAGTTAGGACCTCATTTCACTTTGAATCTCAGATGACATGTTCCTGGATCGGAAATGAATTTGATATAATCAGTCTTTGGAATCAATATGAAACTAGTTCAGTTGGATCCTGCATCAAGTCTGAAACTGGTACATGTCAACCCTGGGTCCATATTGAATCTTCTACAATCACACCATGGACCCAATATGAAACTTTAGAGATCTACCCTTCAACCCAGCCTGAGACTGATACAGCATTAAGGCATTGGTTCCAGCCCCAAATTGATCCAATTAATACTTGGAATCAGCCCGAAGTAGATACAATCAGATTCTGGACCCAAGTTGAAACAGAAACAATTTCAGTTTGGACCCAGATTGGAAGTCAAGTAGTTAAACTTTCCAACTTTTCTGAAGTTGGCATAGTTACACCTTGGCTAGAGACTGAAACTGATACAAGTAGACCCTGGATTCAGTCTGACTTTCAGTCAGTCCATCCTTGGACCCAGACTGGATTTGGTATAATTAACCCCTGGTATCAGCCAAGAGCTGCTGTAAATCAACCCTGGACATTTGTTCAAACACAGTCAATCGGACCCTGGACTAAGGTGGAAGCCAATACAATCAAATCTTGGTTTCATGTTCAAATGAAAAAAGTCAGACTGGGGATTCCTGCTGAGTCTCAAATATTGAGTTTCTGGATGCAGTCTGATGTTAGTAGAGTTAATGCTTGGATCCAACCAGAAACCCAGGCAGTCAATCCTGGGGCTCATCCTAAATCTGGTAATGTTGCATCCCTGGCTATTCCTAAGCCTGAAAGAGTCAGAATGTGGATCCAGCCTGAAACAGAAATAAGGCCTGGCATCATTTATAAAACTGATATAACCACATCATTTGCTTCTCCTGAAATTGAACCAGATGGAACAATTAGTCATTTTGATTTCTTGTCTAATCGTGTAACATTTTTAACAATAGAAACTGTTCCTTCCCTAGATGAGCATTTTGCAGCTTTGTCAACTGAAATAGCTGCAGTAGAAAGCCAAGGTCAAATAAATTCTGTCCAACCCAGTGAGATCACAAATACTCTCTTTCTTACACTTTCAAGCACATGGCTTCCTGGAGGAGCTGGTTACCTGAACTTTGCCAATAAATTGCAAATTACCAAAACAAAAGGAAGCCCTAATGTCCCATCTAGTTCTCTCAACCCACTTTTTCCATCTTTTTCCTTTCCTGTTCCTTGTTTTATCCCATTTTCATGTTCTTTGTCCCTTACTTGTTCAGTCTTTTCTTCTTGCACACTTTCTTCACCATGTACTTTTCCTTCTTGCTCAGTTCTTCCTCTTGTGGCTTTCTCTCCTGTTCTTCCCTTAGCTGCTTCTGATAGTTCTCTCCAGAAACCATCTTCCTCAGAAGTTACTGAAGACACCATTCTTTCCCATACTTTTTCATCCTTGCATGCTGCTCCAGCCACTCTTTTAACAAAGCAACCATCTCTGATGCCTGGATTTCAATCTGAAACCAAGTCTAATCGGCCTGAACAAGATCTTCCTAAGTATTCTGAACTCAATGTTTCCCTTGCTGAGTGTCGCCTGGATGTGGTCTGGAAAGAGAGTCTCCAGGCTTTCTCGCTCTTCAAGACAGCTGTTATTTCTCATGAAATCACAGAGTGTGGATTACGCCCTGGCCTTGTTCCACACTGCCCCAACTGCTGGGAGGCTGAAGTGGGTGAATTCCCTTGGATGGTTTCTGTGCAACTCTCTTTCTCCCATTTCTGTGCTGGTTCTATACTGAATGAACAGTGGATTCTCACTACAGCTAGATGTGCAAATTTCATAAAAAACTCAGAAGCATTGGCCCATGTCCAGGTGGGGCTGATTGATCTTCAAGACCCTGCTCAAGCTCAAACTGTAGGCATTCATCGTGCCATGCCCTACCTGGGCCCTAGAGGACCTTTGGGACCTGGTCTAATCTTCTTGAAGCAACCATTACATTTTCAACCCCTGGTTCTTCCTATCTGCCTGGAGGAGAACCTAGAGCAAGAGAAAAATATACAACTATATGACTGCTGGCTACCTAGTTGGTCCCTCATGAGAGGAAGTCCTGGAATTTTGCAAAAAAGGCATCTGAGCATCCTGCAAGTCATCACATGTGCCCAGTTTTGGCCCAGCCTGAATGAATTTACTTTCTGTGTGGCAGCCAAGAAAGCTATGGGGGAGGCTGGCTGTAAGGGTGACCTGGGGGCACCTCTTATATGTCATCTGCAACAAAAAGACACATGGGTGCAGGTGGGAATCTTGACTCACTTTGATGAACACTGCAGAAAGCCCTATGTCTTCAGCCAAGTGAGCCCTTTCCTTTTCTGGCTCCAGGGAGTTACACGACCCAGCCAAGCACCCTGGTCCAAGCAAGGGCCCATGACCACCTCTGCTTCCATCTCCCTTTCAGTCTCCACCTCTATGAATGCCTCAGCTTTTACCTCCACACCTGCTTCTGTCCGGCCACATTTCATCTCTCTGCCACAGCCTCAGACTTTGGCAGATCGAATTTCTCTGAGATATGCCATGCCTTGGCAGGCCATGATCATCAGTTGTGGCAGTCAAATTTGCAGTGGTTCCATTGTTAGCAGCTCTTGGGTACTCACCGCGGCTCACTGTGTCAGGAATATGAATCCTGAAGACACAGCTGTAATATTGGGCCTGAGGCACCCTGGGGCACCTCTGAGAGTTGTTAAGATCTCTACCATTCTTCTGCATGAAAGATTTCGATTGGTGAGTAGGGCAGCAAGAAACGATCTAGCATTGCTGCTCCTTCAAGAGGTCCAGACTCCCATTCAGCTTTTAGCACCGTTGGGTCATCTGAAGAACCTGAACAGCTCAGAATGCTGGCTGTCTGGGCCAAGAATTCTTAAGCCAGGAGAGACAGATGAAAATCCAGAAATATTACAGATGCAGGTGATAGGAGCTTCAAGCTGTGCCCACCTATACCCTGATATAGGTAGTTCCATTGTCTGCTTCATTACACAAGACAAAGATTCTGACACAAGTGTGGAACCAGTGAGTCCAGGCAGTGCTGTCATGTGCAGACCAATCTCTAGGAATGGAAGCTGGAGACAGATAGGCCTCACTAGTCTGAAGGCACTGGCTACCATTGTGAGCCCCCACTTCTCATGGATATTATCCACTTCATCAAAAGCAGGGCATCCATTAAGCCATGAACTAATGCCTTGGATGGAAAAGCCTAAGTCCTCTAGTCTCATAAAACAGCCAGCCACCCTGCCATTTTATTCAATAATAATTGTTATACTACAAAAGCTTTCATAACTCACTGTGAAAATAAGGCAGGGCTAATCTATTCAAACTATTTATAATAAAAATTTTAAACAACATTAAAGAAAATTAAGACCCTATGCAACCTAGGAG

> Two-lined Caecilian, Rhinatrema bivittatum (BK059519)

TCTCTCTCCTGGTTTCAGGTAGAACCTTATTACTGTCCCACCTGAGACTTCAGCCGGCGTTCACCTAACAGAAGGATGAAATACCATTTTCTTATTGCCATCAATTTGGTGTTTCAGTGTTTAAGCTACAGCAATGCACTGAGCCAGGAGAGATGTTGTAAAGAGGAGACAGATCCGGGCGCTCTTCCACCACTTCTAAAGTTTGTTAGCTGGGATCTAAGCTTTCTGTCATCAGATCCTGCTACTCGAAAAAGGCGTTCCGTATTAGCCAGTGCACCTCTAGCTGAGCAAAAACACAACAATAGAGACCAGCATCACTATAAAGCCAATTCTTACTCATCCAGGTTACGACATTTGTTCTCCTCAAGATGGCCACATGGCCATGGAAGGCCACATCAAGCTCCTGTTAGATCACCTAGGACAAGACACCATGGCCATCCTCATGCCTTAGCAAAGCCATCCGATTCAATTCAGCCTCTGGTTGTACATCCACCAGCTGGTCCAAGTCAGGCACTTCTTTTACTATCACCAGCTGGCCCAGGTCAGCCTCTGCACGTATTGCCACTGAGAATGACAGATCCACCAGAGTTGCGGAAACCTTCAAGCCAAAATCTTCCTCTCACCTCATTGCAGCCAACAAGCCAGAGTCAGTCTCTCACCTCACTACAATCCTCCAACCAAAGTGGACCACTATCCTTACTGCAGTCGTCCAGCCAAGCTCAGCCTCTCACCTCACTGCAACCCTCCAACCAAAATGGACCACTATCCTCACTGCAGTCACCCAGCCAAGGTCAACCTCTCACCTCACTGCAATCCTCCAACCAAGGTGGACCACTATCCTCACTGCAGTCACCCAGCCAAGGTCAACCTCTCACCTCACTGCAATCCTCCAACCAAAGTGGACCACTATCCTCACTGCCGTCACCCATCCAAGGTCAGCCTCTCCCCTCACTGAAATCTTCCAACCAAAGTGGACCACTATCCTCACTGCAGCTGCCCAGCCAAGGTCAGCTTCTCTCTTCACTGCACTCTTCCAACCAAAGTGGACCACTATCTTCACTGCAGCTGCCCAGCCAAGGTCAGCCTCGCCCCTCACTGAAATCTTCCAACTTAAGTGGACTACTATCCCCACTGCAGCTGCCCAGTCAAGGTCAGCCTCTCTTCTCACTGCAGCCATCCAGCCAAGGTCAGCCTCTCACCTCACTGCAATCCTCCAACGAAAGTGGACCACTATCCTCACTGCAACCATCCAGCCAAGGTCAGCTCTCACTGCAGACATCCCGCCAAGGTCAGATTCTCCTCTCGCTACAGCCATCCAGCCAAGGTCAGTTTCTCCTTTCGCTGCAGCCGTCCAGCCAAGGTCAGCTTCTCCTCTCGCTGCAGCCCTCCAACACAGACCAGCCTCTTGTGCTGAAACTTCCTGACACAGGTTTGCAGCAACCATCTCCATCCTTTGTCACACCCGTACCCCCAGAAAATCGCACTTCTTCCTTCATGAATCCATCCCGCCTGGTGCGCCGGGGAGCTGTCATCCTTTCTCACTGTGGAGCCGACATGGAATGGAATGTTGGTAGCCAAGCTTTCCGTCTCTCCAAATTCTCCATACAGCGGAAGAGTTACCAAGCATGTGGCCAGCGCACGGCCTTCAAGCCCAAGCTCACCCCAGCAAACAACGAGGAAGCTGAAAAGGGAGAGTTTCCCTGGATGGTCTCCCTGAAGTTGTCCATTTATCACTTCTGCTCGGGCTCCATCCTCAACCGATGGTGGATCCTCACTACTGCGTCCTGCACCAACATCATAAAAAACGAGGAGTCCTCTGTTCTGGTCCAGGCCGGCATGCTGAACTTCCAGCTAGACTTTCGTTCCTTTCACGTAGAGCTGGTGGTGTCCCATCAAGAATACACAGAGGACCAGGAGACACACAACCTTGGTCTCATTCTGCTGCGCGAACCTTTATTCATCAGCCCTTTGATTTCACCCATTTGCATCTCCAAGAATATAAAATTGGAGCAGCTAATGACGCCCACCAACTGCTGGATCTCTGAATGGACATCACTGCAAGGTGGCCCCAGCATCCTGCTAAAGCGCAGGGTTTCACATCTTCAGCACACGCTTTGCAGTGACTTCTGGCCCATCATCTCTGATTTCACCTTTTGCATGAAGCTGAACCCGACCAACATGACAAACTGCAAGGGAGATATTGGGGCCCCTCTGGTGTGCAAAGACTTCAACAGTTCATCCTGGCTGCAGGTCGGATTACTCAGCGACTATGACAAGACCTGTGTGAAGCCCTACGTCTTCACCAAGGTCTCCCACTACCTGTCCTGGATTGAGCAGAGCACCCAGGCAGCCGGCAAACCCATCAGCCGGACAAAGTCTACATCTGCTGGGCGTCTTCAGAAGAAATGGCTGCGGGAGAAGGGCGATTTGCATACCGCTGAAAAAGACTCCATGTACCAAACAAAATCTTTTCGTATCTTTGCGCCATGGCAAACCCTTATCATCACCTGCCAGAACAAGATCTGCAATGGAGCCATCCTGGACAAGTACTGGATTGTCACCACAGCTGGCTGTGTGCAGAACATGGATCCGGATGATACTGCCGTCTATATGGGTCTTAACAGGCCAGAGCACATTGGTGACGTTATCAGAGCTGACCGTATCTTTCCACATGATGGTCATGATGAGAGTGTCAGTGTGGGAAATGACATTGCTCTGATCCTGCTAGAGGGTCCCATCTTGTTTTGGAAGCACGCCAGACCCCTGACCATGGCACGCGACCTGAACCTCGACATCAGCAGCATGGACACGTGTGGGATAGCAGGACTGCGCTGGTTAGAATCAGGAAAGGAATCATCTTCCACCATAAACTTGAAGAAGATACAGGTGCCAGTGAAAAATTCTGAAGTCTGTCCTGAGGATGAAGCCCTGATACAAAACGTAGCCTTCTGCATTGAGGAGGTCAGCACCCACCGCCAGCTACTGATGATCCAAGAGGGCAGCGCCATCCTCTGCACAAGCAAACAGGACTCAAACTGGACGCTGGTTGGCATTCTCAGCAAAGTATTGGATGAGTCCCCCATGCCTGCCTTAATCACCAGGCTCGCTGCCCACATCGACTGGATGAACAACGTGAGCAAAGCAGCCGGGAGACCGCTGGAGCTGCCGCCCACCACCCTGTCCCAGAAGGATCTCGAAGTGTCCACTTCCCGGGCCCACACTGGCCTTGTCCTGCTGATCGTTCTGCTCTCCTGCTTTGTTCTGCTCATCATCATCATCGGTGTGGGTGCTTTTGTACTGCACAAATTTTTTCCTAAATTCTTGACAGATTTAAAGTCAAAGTTGAAGTTGAAGTTCAAGTCAATCCTTAAACCCAAGCCTGAACCCAAGCCTGAACCCAAGCCTGAACCCATTCCTCTCACCCCCACTCCCTCAAAAAAAGCTAGAAAAGCAACCCATTTCTCTACTCCCTAAATATATATATATATATATATACACACACATACATACAATACATTATTGAAAGTGGGTAACATTGAGCAAGCAGTTGATAGGGAAACTAGGGAAACAATTGTAATATTCTGCTCTCATAATAGGCAGTTTCCTGATACCTACAAGTATTTTCAGTTACCACAAAATATAAGAACCCAGTGCAGATGCTCAGTATTAGAGAATTATAGTCACCTGAGAGGGAATGAATTGTGTGCCAGATAAAGCCAAACTGACCAATACAATTCAGCATTTTCCTTCTTATTAATTTAATGTATGTGGGAGATACATCTAAGCCATGATATTTTGAACTGGATTTCAGGATGTTTTCCATTGATTAGTTCATGAGTATTCTCTTTCAAGCAATAAAATAGTTTTCATACTTAAAAAAAA

>Camel, Camelus ferus (BK059510)

CAAAAACTCAGAGGCCCTGGCCCTGGTCCAGGTGGGGCTGATTGATCTTCAGGACCCTGCCCAGGCTCAAACTGTAGGCATTCATCGTGCCGTGCCCTACATAGGCCCCAAGGGACCTTTGGGACCTGGACTAATCTTCCTGGAACAACCACTACATTTTCAACCCCTGGTGCTTCCTATCTGCCTGGAGGAGAGCCTGGAGCAAGAGAAAAACATACAACTGTATGACTGCTGGCTACCCAGTTGGTCCCTTATGAGAGGAAGTCCTGGAATCCTGCAAAAAAGGCACCTAAGCATCCTGCAAGCCAGCACATGTGCCCAGTTTTGGCCCAGACTGAATGAATTTACTTTCTGCGTCGAAGCCAAGAAAGCTATGGGGGAGGCTGGCTGTAAGGGTGACCTGGGGGCACCTCTGGTGTGCCATCTGCAACAAAAGGACACATGGGTGCAGGTGGGAATCTTGAGTCACTTTGATGAGCACTGCACAAAGCCCTACGTCTTCAGCCAAGTGAGCCCTTTCCTTTTCTGGCTCCAAGGAGTTACACGACCCAGCCATGCACCCTGGTCCAAGCAAGGGGCAATGACTACCTCTGCTTCCATCTCCCTTTCGGCCGCTACCTCTACAAACGCGTCGGCTTTTACCTCCACTCCTGCTTCTATCCGGCCGCACTTCATCTCTCTACCACAGCCTCAGACTTTGGCAGGTCAGATTTCCCTGAGATACGCCATGCCTTGGCAGGCTTTGATCATCAGCTGTGGCAGTCAAGTTTGTAGTGGTTCCATAGTAAGCAGCTCTTGGGTACTCACTGCTGCCCACTGTGTCAGGAACATGAATCCTGAAGACACTGCTGTGATACTGGGCCTGAGGCACCCTGGGACACCTCTGAGAGTTGTTAAAGTGTCTACCATCCTGCTGCATGAGAGATTCCGGTTGGTGAGTGGGGCAGCAAAAAATGATCTCGCATTGCTTCTCCTTCAAGAGGTCCAGACTCCCCTTCAGCTTTTGGCACCCTTGGGCCATATGAAGACCCTGAATAGCTCAGAATGCTGGCTGTCTGGGCCACGAATTCTTAAACCAGGAGAGGCAGATGAAAATCCAGAAATATTGCAGATGCAAGTGATGGGAGCTTCAGGCTGTGCCCACCTCTACCCTGACATAGGCAGTTCTATTATTTGCTTCATCACACAGGACAAAGACTCAGAAACAAACGTGGAACCAGTGAGTCCAGGCAGTGCTGTTATGTGCAGACAAATGTCTGGGAATGGCAGCTGGAGACAGATAGGCCTCACCAGTCTGAAGGCACTGGCTACAACTGTGAGCCCCCACTTCTCCTGGATATTATCAACTTCAGCAAAAGCAGGGCATCCCCTAAACCAGGCACTGATGCCTTGGGTGCAAAAACCCAAGTCCTGTAGTCTCCTAAAACAGCCAACGACACTGACACTTTCTTCAGTGGTAATTATTGCAGTACAAAGTCTTTTGTAACTCCGTGGCTATGGAGGGGCATGCTAATCTGTTCACACTGTGATTAAAAAAAGGTATTAAAACAATTAAATGAAAAGATTCATTTAAGCCCTTGCAGAATCTGAGTACATAGTCATGTACTTAAATAAAGACATGCTGTATCCCTAACGACTGCTTTCTTTTTACATCCTAAAGGGAAAACAAAACAAAACAAAACAAAACAAAATCCAACTTTGCTTAGCTCTTTTCAAATTTAAGAGGACTCACATCTATACATTTTAACATTCAAATGTTACCAAGTCCAAGCTTCTACTGCTTGACACACTACAGGACAAGAAATTGGGAGACAAGG

>Chinchilla, Chinchilla lanigera

CAGGCTTCATCTAACCCTATCATATTATGGACCTGTTCTGAATCTTCAGCAGTCATTGACTGGATATATCCTGTAACTAAAATATTTACAGCACTGACTCAGAATGAACTGCTAATATTACCTGATAAAGTCACGTTGTGGACACAAGCTACATTCACATCAGACAAGACTTGGAAGAAAGCTGAATCTTTCTCAGGAATATCTTGGACTCAGGCTGTGTCTACATCATTAATTTCCTCCATACAGCCTATGTCTGATGTATATATACCACAGACCCTGGGTGAGTTTCTGGCAGCAGTTACATGGATACAGCCTATGCCAAATACCCTGATACAATGGGTGCAGTTTGAATCTCCGGGAGCCAAGGTTTGGACTGATATTACAACTTCCACAGTAACACAGTGGAGCCATACTGAATTTCCAGTTTTAAATCTATGGACACCAACTGTTTTATCCACAACCACCATGAGGAGACTAAATGAACTTCTAGCAGTGAACCTTGGGTCAATGGCTACAACAAGCATAATCTCCACATGGACCCAGTATGAGTCTGCAGCAGTCCAGACTTTGTCAATACCTTTAGGTTCTACAGCCACAAACTGGAAACAGATGGACTCTTCATATATGGATTCTTATAAACAGTTTGAAACTGATATAACTATATTTTCTAAAATGCTTACAGAAACCTTGACGCTGCCTGAAGTTAATATATCCATACTGTCTATGCAAACTCAAACTAATACAGCCCAACACATAAATCAGATTGAAATTCAAGAATATCTTCTGAGGACAGATCAACTTGAAAATGGGTATACGTGGACTGTGCCTGAGGCTGGAGCACTTCTATACCAGACATTGCCTGTACATCAAGCAGCCACATCCTGGCCACAACTGGATTCTCATATTAGCAGAGATTGGTTTAAAATTCAAGCAGAAAGTATTCAACCATGGGCTCAGTTAGAATTTCAAAATGTCACCTCATTTAGACCTAGAAAAATAGAGCTCTGGGTCCACAATACATTTCCTGCAGTCACATCATTTATCCAGTCAGAAATTGGTATATCTGATCTCTGCATATCTAAAGTAGATATAACAAGGTCTTGGACAATTTCAGACACTGATATAGTAAAATCAACAATCCAAACTGAATCTCATTCCATTAATGACTGGATCCAGCCAAAAACTAATACAATCAGACCATTTACCCAAATTGAACATGAAGCAGTCAGGTTCCATACTGAGGATGTATCTGATGTAGCCACACTATTTTCCCAGATAGAAATGCAAGAAACAAAACAGCTGACCCTACCTGATATTCATAATGTCAGAGTTTGGTTCCAGACTCAAAATAATGCAATACAAGGAACTCAACCTGAAGCTCAAATGGTTTCTCCCTGGATCCAGGCAAAATGGCACATAGCATACACCTGGCACCACCCTGAAACTAATGTGATCAGAACTCCATCTCAGACTAAAAGTGATATAGCCCAACTCATGATCTATTCCCAAAGAAATACATTCAAAATCTGGACCTATTCTGAAAGTGATGAAATGAAACTTCTGACCCATGCTGAATGCCAAGAAATTAAGACCTGGCCTGAAGCTGATTTGGTCACACCTCCAATGGCAATGAGGCCTTGGTCCCTGGTAAAATCTCAAGTGACACATTCTTTGGCCCAAACTGAAGTTGGTATAACCAACCCTTGGATTGAGCACAAAGATGGGATGTCCAGGCAGTGGTCCCTGTCTGAAACTATAGAAATTCTTCATTGGACCTGGCCTGGTACTGTAATAGAGTACTGGCTCAAACCTCAGATAGATCCAATCAGATACAAGAACCAGCCTGAAACTAATATAATTGGAATTTTGACCCAGACTGTGAAACAAGCAGTAAAAATGCCAAACTTGGCTGAGGTTGATGGAGTGACACCTTCATTACATGCTCAAGGCAATACTTCTATACATTGGATTCAGCCTGATTCTAAAACAGTGAGTCTTGGGATCCAGAGCAAACTTGGTTTAATTGGGACCCAGACTCAGCAAAGTGATGTTACAAATCACCCTGAAACCCAAGCAATCAAACCCTGGCTAAAGCCTGAAGCTGATACAGTCAGATATTTGTTCCATATTCAAATGAATAAAGATTTGCCATGGATTTATTCAGATGATCAAATATTCAGTTCCTTAATGCAACCTGAAGTCATAACATATGCCTGGACCCAATCCAAATCCCAAGCAATCAGACCCAACCTAAAATTAATATGGTCTGAAACTTTCACAGTCAGATCTTGGTCTGATATATTTCAAGTCACTCTACCACAGACTTTACTAATAGGAAGAACACTCACACCCTGGATTCAGTCTGAAAACCAACTAGACAGATCCTGGACCCAACATGAAGCTGAAGTTTCAACAAAACAAGCCATGCCTGGATCTCAGTTTGAACCCGACTCTAAGTATCAGCCTGGACCACAGCCTCTCAAGCATTCAGAACTGAAGGTTTCTTTGGCTGAATGTCACCTGGGTATAATCTGGCAAGAGAATCTCCCGGCTCTCTGGCTTGTCAAAACACCTGTTATTTCTCATGCTGTCACAGAGTGTGGATTACGCCCAGGCTTTGTTCCTCACTGTCCCAACTGCTGGAAGGCAGAAATGGGTGAATTCCCCTGGATGGTTTCTATACAATTGTCTTTCTCCCATTTCTGTGCTGGCTCAATTCTGAATGAATACTGGATCCTTACTTCTGCTAGATGTGCCAATTTCATAAAAAATTCAGAGGCCCTGGCCTTGGTACAAGTGGGGCTTGTTGATCTTCAGGACCCAGCTCAAGCTCATATTGTAGGCATTCATCGTGCCATGCCCTACATAGGTCCCAAAGGACCTCTAGGACCTGGTCTGATATTTCTGAAGGAGCCACTACATCTTCAACCCCTGGTGCTTCCTGTTTGTCTGGAGGAAAGTCTGGAGCAAGAGAAAAATAAACAATTATATGACTGCTGGCTACCCAGCTGGTCCCTTATGAGGGGAAGACCTAGAATTCTACAAAAAAGACACTTAAGTATACTGCAAGCCAGCACTTGTGCACGATTTTGGCCAAAAATAAATGAATATACTTTCTGTGTGGAAGCCAAAAGAGCTATTAGGGAGGATGGCTGTAAGGGTGATCTAGGGGCACCTCTAGTGTGTCGTCTACCACAAAAGAATACCTGGGTGCAGGTGGGAATATTGAGTCACTTTGATGAACATTGCAAAAAGCCCTATGTCTTCAGCCAAGTGAGCCCTTTCATCTTCTGGCTTCACGGAGTAACACGGCCTAGCCATGCACCCTGGTCTCAGCAACGGTCCACGACTACCTTTGCTTCTACAACCCCTTTAGTCTCTACCTCTACCAATGCCTCAGCTTTTACTTCCAATTCAGCTTCTATTCAGCCACACTTCAACTTTCTACCACAGCCTCAGAGGAAGACCCAAGACAATGGTAGCAAAGAAGCTTTGACAGATCACATTTCCCTTCATTATACTATGCCTTGGCAGGCCATTATCATAAGCTGTGGCACTCATCAAATCTGCAGTGGTTCCCTTATTAGCAGCTCTTGGATTCTCACTGTTGCCCACTGTGTCAGAAACATGAATCCTGAAGACACTGTTGTAATACTGGGCTTTAAACATCCTGGGGATCCTCTGAGAGTTGTCAAAGTGTCTAATATTCTACTGCATGAGAGATTCCGATTGGTGAGTGGGACAGCAAAAAATGACCTAGCACTGTTGCTCCTTCAAAAAGCCCAGACATCCATTCAGATATTAGCACCTTTGGGCCATATGAAGAATCTAAGTGACTCGGAATGCTGGCTGTCTGGACCACAAATCCTTAAACCAGGACAGACAGAAGAAAATCCAGAAATGTTACAGATTCAGGTGATGACAGCTTCATCCTGTGCCTATGTATACCCTGACATAGGTGGTTCTATTGTTTGCTTCATTTCTAAGGCCAAAGGTGCTGACACAAATATGGAGCTAGTAAGTCCAGGAAGTGCTGTTATGTGCAGAGAGAAATCTGCAAATGGCAAATGGATACACATAGGATTCACCAGTGTCAAGGCTCTAGCTACAGTTGTGAGTCCACATTTCTCCTGGATTATATCTACTTCAGCAAAAGCTGGTCATCCACTAAATCAGGCCATTATGCCTTGGATGGAAAAGCCTAAGTCCTCAAGTCTCCTCAAAGAGTCAAGTACATTGCCACTTTTTTCAATACTGATTGTTGCAGCCCAAAGAATTTTGTAGTCCAGTACCTACAATTGGAAGTGCTAGTCTGCTTACCCTGAGAGATAAGATAATGAAAATCACCAAAGAACAAAAGATTGACTTGTGTCCTATATTATATACTTCCTATGTTACGTAGTATTCAATAAATGGAATTTTCCTAAAAA

>Deer, Odocoileus virginianus (BK059509)

ACTCTATAATCTTAGTCTCAGCCTTGAGAGCTAGTCTATTAGAATCCTGTTCTTGTGTTCACATCTATGTGAACAAATATGAAAGGTCCACTTTGCCTCTTTCAGTTTTGGGGGTTGTTTTTTCACTGCTATGGTGTACCCAATTCTCAAAAGCAGTGGTTAAACTCATTGACCCCAACGATTGCCCCATTGTCAGCATCCAACAATATCCCATGGCTAGTGTCCATGGCTGAGACCTGCCAGGGCATTATTCTGAGTCGGTGGTGGATCCTCTCCACAGCCAGCTGTCTGAGTAAACTGAAACAGTTAAACTCTGACATTTCAGGAGTCTTTGACCAAGAAGATGTCTTACAAGGCCACAAAGTATGCCTACTCCCTAGTTTTGATCCAGAAACTGGAAAAGATCCAGTCAAAGCAGATATAGGGATAGTACTCCTTCAATATCCTATCAGGAAGAAAGAAATACCACTTTCTCACACTTATAACATCTTCTGGAAGACCTGTTATAACTGCCAATACAGAGACTGCAGGGTGTACCAATATCAGAATCATGGTAACTTTGAAAGCAATATCAAGAAATTGTCAGTTAAGCTGCTGGACCTCTCATTTTGCCACCATCAACACATTCACCTGACTAAAAGTAACAATTTATGCATCTGGAGTCAGTCAGAAGAAGACTGCTGGGTACAGCAGGGTAGTCCTGTTTTCTGTCTCTTTGGCAACCACTGGGAACTGGTAGGCCTGGTCAGTGAGTCCTCAATGGCCTGTTATGACCCAATTCTTGTCATCAAGACAGCCCCCTACCTACCTTGGATGAGACAGCTTATCAAGGCATCCCAGAAGTCACTGGATCCTATTTTTTCTCTACCCTGCAGTTTTTCTTCTAGGGTAGAACATGATAATCAATATAGGCTTATCCCAAAAAATGCTTCTGCATTTCTGTCCTCCCATGGATTCTCTATACAGTTATGGAGGGGAAAGTTTGGCAGTTCCCCACTGAACAGACAGCGCCGGAATCCTCCTCCAGTATTTTTTGGTCCAAATGACAGAGATTCTTTTCCTGGAAGTTCACAGTTATACCTTCAAAGCAGTCAGATCTCCTCAACTAGCAATTCCCTAATGGTAAAATCTTGGACCTCTCCTCTTGTTAAATCATGGGATCCTTTTCATACAGCTGTGTCCTGGAATACTGCAGGAACTGATATTTCTGATCCTTCAGTTCTTTATCAACCTCAAAGCACTCCAACACCAGATATAAGTACACCCTGGAACCTTCCTCAGAGAGATGTAGTGAAATATAAATATAGAAATATGACAGATTCATCAAGATACTGGGTTGAATATTTAGGTGGTATAATTGGGCTTTATTCTCCACCATTAAATAATGCTGATGGATCACATGTTCTGTTTTCAAGTGATGTAGAAGGATCCCAATTTCATTCTGAGATAAAAAATGCTCAGTCTCAAGTTCAATATGGCAGGGTTCCTTTCCAAGGTCAATTTCTAGGGAGACCCTGGCTGCGTATTGCATCTGGTGTTAGACCTTGGACATATAATATGGCTGATAAAACAGAAATAGTGATACAGATTCAACCTGCTGAGGGGAGTTTTAGAACTCAAATACATCATGTTGCTGATAGAGTCAATACTGCTATTCAATCTGTAACTTATAACATGAATCCATGGATACCTTTGATTATTAATAAGATTGGATTCTGGACTCATTCCATACTAAATGAAGAGGGATCTCAATATCCTACAGCAACTCCTACCTCAGAACCCTGGTTTCAGCCTGTCCTAAACATAGTTAAATCCCAAGGACCTATAGAAAAGACATATGAACAAATGATTTTCCCTGAACCTAAGCCAACTCAATTTTGGACTTCCTCAACTCTTAATATGCTCCTTAATTGGGCTTCATCTGCAAGTAATACTAATAGGCCTGTGGCCCAGTATAAGGCCAGTGCAATCACATCCTTAAGTCAAATGGATAGAATGAATCCAGTGAGTAAGCACAGTGATTTTACAGTCAAAACCCAGATGCAGAAGGAAGATACAACATGGCTTTTGATTCATACAGTTGCTAGTATAATTGATCCTTTAACCCACACTGAAGTTGATACAACTGCACCACGGACTCAACCTGAAGTTGACATAGTCCAAACTTGGACCCAGTCAGAAACTCAATCAGGAAGCCCTTTGACTGTGCTGAAAGCTGATACCACCAAACTGTGGTTACAGACTAAGGGAAGAAGAAGACACCAGATTCAGCCTAAATTCCAAATTCTCAGACCTAGAACTCAGACTGAAGAAGGTGAATATAAACCTTGGATCCAGTCAGAAGCAGATGCTGTCAGATCCTTGACCCACTCAGAAATGGAAACACTCAGACCCTGGGACCAACCTGAAACTGGTACTGCCAGATCTTTGTTCTTGACAAAATCTGACCAAATAATACTGAACACTCAGGCAGCCTTTAAAACACCCCACATATGGACTCAGCCTGAAGTTGACACAGTCAGGCCACAGATTCAGTCTGAAGCTGGCAGAATGCAACAATGGACAAAGCCAGAAAAATCTCCTTTAATACTCACAACAGAGTCTAAAGTTAATACAATTACACCATGGACAAGGCCTGAAGTTGATGCTATGGGGCTTTGGTTGCAAACACAAACTGATATAATCAGGACCTGGACCCAAACTGAAACTCAAACAATAATTTCTTGGTTTGAACCAGAAGCTATTATAGTCAGACCTCTGTTGAATACTCAAACTGATACATTTAAACCTTGGATTGAGACAGAAATAGAAACACCCCATTACTGGGCCCAGTCTGAAGGTAATATAGACAGATCATGGACAGAGATTGAAGCTGAAAATGTCAAACCCTGGTTCTGGACTCGAATGGAAATAGCCACTTTTTGGACAGAGCCAGTAATCAAAACATCCCACCGCTGGATGCAGTCCAAAAAGGAAATACTCAAGCCCTGGGACCAATTTTATGAGGTAGAAACCTGGACACAACATGACACTAAAACACTCAAGTCCTGGAATGAGATTGAAAGTGATAAAGATAGATTTTGGACTCAATCAGAAGTTGACACATTGAGACCCTGGATTCAGTCAGATATTGCTATCAACAACCATGGAGCGGAAAATGAAGCTGGTACATTAATGCTTTGGCCTCAGGCTGAATATCCAGAAGTAAATCTCTGGACACAATCTGAAACTGATGCAGTCTCATTATCAACCCAGAGTAAAATTCCAGCAGTAAATCACTGGACAGAAATAGCTGATACCATCATACCACAAGCAAAAGCTAAATTTCCAGGAGTAAAGCCCTTGACACATCTGGCATCTAATACAGTAACACCATGGACTCAGGCTGAATCTATAGTAGTAAATCTCTGGACTCAGCCTATAACTGATGCAGTCACACAGTGGACTCCTGGTGAGTTTTCAGAGACAAATCCCTGGACAAAAACTACAATTGATAGAGTCAAGTCATGGACCCAGGATGAATCTCTACTGGCAAATCCCTGGATGCAATTTAACACTAATACAGTTATACCACGGACACAGACTGACTCATTCATAGTAAATTCTTGGTTACAGTCTATACCTCATACCATTATACAATGGACCCACAGTGAACCTTTAGATATAGCTCCCTGGAAAAAAGCTGTCACTGATGCAACACCTTGGTCTCAGGCAGAGTCATCAGCAGTAAATCCCTGGAGAGAAAGTGCAACTGGGACAGCTACACAGTGGACCCAAGGAGAATCTCCAGTAGTAAATTTATGGGCTCATCCTGTGGTTAATGTAGTCACACTGTACACTCAGGCTGATTCCCCATTGTTACATTTCTGGACAAAGTCTGAAATAAATATAGTCACAACATGGACCCCAACAGAATCTTCAGCCATAAATTCCTGGAGACAGTCTGAAACTGACAGTATCTCAACATGGACCAATAAAACAAATCCAGAAATAAACAGTTGGTTATATTATGAAACTGATACAATGACAGTTTGGACCCAGCCAGAAAGTCCAGGAATAAATACTTGGACACCAGCTATTGCTTATGCAGCTCCACCATGGACTCAGGCTGATTTTTTAGCTGCAAATCCCTGGACAGAGGTTGAAAGTGATACAATCACACCATGGACTCAGGTTGAACCTTTAATGAATCCTCGGTCAGAGAGTGTAGCTTCCACAGTCATACCATGGACCAAGGCTGAATCTCTAGCAGTAAATTCATGGATACAATTTGCAGCTGATGTAGATACACTATGGACCCAGACTGATTCTCTAGCAGTAAATCCCTGGATATATCCTGCATCTGATGATGTTATACAGTGGATTCAAACTGAACTTCCAACAGAATATCAATGGATTCAGAATATGTCTGATATAGTAACATCAATTACTCAGGCTGAATTTCCTTCAGTAGAGACTTGGACAGATCCTCTGGCAGATATAGTAACAAGGTGGAATCAGACTGAATCTCCAATAATAAATCCATGGTCAGAAGCTGTAGTTTCTACAATGACACCATGGACTCAAGTTGAATATCCAGAACTAAATACCTGGATAAATTCTATAGCTTCCACAGTCACACAGATGACTCAGCCTAAATACCCAGTAATGAAGCCTCAGAGACAGCCCATATCTGGTACAGCCACACCATTGAACCAAACAGAATCACCTATAGTAAGTCCCAGGACACAACATGAAACTGATGTAATCACACAGAGGACTCAGACTGAATCTGAAAGAATTAATCCTTGGATACAAGTTGTAACAGACACAATCATACGCTGGACTCAGGCTGAATCTCCAGCAGTCAATTCCTGGACAGAGGCTGTTGCAGATACAGTAATACCATTGAATCAGACTGAATATCCTTCAGTAAATCCCTGGGCAATGCTTGCAACTGATACTTTTACACTTTGTACCCAGGCAAATTCTTCCATCATCAATTTTTTGACACAGTTCATAGTTCATACACTCACACCCTCCACCCAGACTATGCCTGTAGCAGTAAATCCCTTGTCACAGTTTGAAACAGATAGAGTCACACAATGGATCCAAGCTCAATTTGCTTCAGTAAACCCCTGGACTCCATCTGAAATCAACCCAATTATAGCATGGACCTATGCTAGATATTCAACCTTAAACCCCTGGACACAATTTGAAGCTGACATATTCACATTATGGACCCAGGCTGAGTCTCCAGCAATAAATATAGCTTTATCTAGGTCAGTGAAGCCATGGATCCAGGTTGAATCTCCTGCAACAGGTCCCTTAACAACAGCCATAGCTGGTGAAATCACACCATGGATCAAGGCTATATCTCCTGCAGTTAATCCCTGGACAAAATCTAAATATGACATAGTTCCACTGTTGATCCAGGATATAACTCCAGCAGTAATTCCCTCAACAGTCTCTGTAATTGATACAATCACCCTGTGGGACATGGCTAAAATTATATTACCAAGGCCTTGGCCACAGCCTGTGACAGAAACAGTCACACACTGGATCCAAAGTGAGTTTTCCTTGGTATATAACTTCACACGACCTATAACTGATGTAACCACATTGTTTACTCATGTTTTTACTCCAGAAGTAAATCCCTGGTCAAGGCCTAAAGCTGATACTGTCAAACAATGGATCGAGGTTAAATCTCCAAAGGAAAAGGCCTGGAAAGTGGCAGGTTCTGAAACACTCACACCATTGACCACAGGATTCTCTACAACAGTTAAAGTCTGGATAGACACTGTGTCTGATATATTCACACCAGGAACCAAGTACACATTTTCAAAATTAAACCTTGGAATAATGCCCGAGGATGGTATCATTGAACCATGGACTCAGTCTGAATCTCTATCAGGAATTCCCTGGACACAACCCGCAGCTGATTCAGATACAAAGCAGATACAGACTAAATACCCATTAGTAAATCTGTGGACTCAGGCTCAGTTCCCAGCAGTAAATCCCTGGACACAGTCCCAGTCACTACCGATAAATATTTTTACAAAATCCAAAACTCTAGGCATAAATCTATCAATACAACCTGAAACTACTACAGTCACGTTATGGAGTCAGGTTGACTCTCCACCAGTAAATCACTGGATAAAACCTGTATCTTCCACAGTCATACCATGGACACAGGCTGAATATTCAGCAGTACATCCATTGATGTGGTCAGCAGCGAATGCAGTCACATGGTGGACCCAAAGTGAATCTCCTGCATTGTATTCCTGGACAAAGTCTACAGTAGATACAGCCACAAAGTGGGCTATGGGTGCATCTCCAGTGTCAAATTACCAGACACAACGATTAGCTTACACAATCCCATTTGGGGTCAAATATGGAACATCAAGGATAAATCCTTGGATGCATTCTAATTTTGAGCCATTTCCATCAACACAAGTAGAATCTTCAGCAAATAAACATGGGATACATATAGAAAATTATAGAGTTATAACAATGCCCCAAATTGAACCAAGTAAAGTGAATCTCAGGCCAGAGCCTATACATATAGGAAGAAAATGGACTAAATCTCAGTTTTCCATAATTCGTTTGACAGGGACTGTGTCTTTCACAACCCGATCATGGACAAAGGCTGAAAGTTCAACTGCAAATACATGGAGAGAGACTGAGTCTTTAACATCACCAACATTGACTCAAGCTATAAAGCTATCCTTTAAAGAGGATTTTATAGGCCTTTTTATAGGCCCAACATGGACACAGGCTGAAAATACAGATGTAAATACCTCAAGAAAAGCTGTTTCTTTTACAGCCCCACCATGGACACAGGCTGAAAGTCCAGATGTAAATATCTGGAGAGAGACTGTATCTTTCACAGATCCCCCATGGACTCAAGTTAAAGGTCCAGCTATAAACAACTATAGAGAGATGTTGACTTTCACAGCCCCACTGTGGACACAGGATGAGGATCCAGCTGTAAATACCTGGAGGGTAACTGTGCTTTTAACAGGCCCACCTTGGACTCAGGCTGAAAGTACAGCTGTAAATGCTTTAAGAGAGACTGTGTCTTTCATAGTCCCACCGTGGATACAGGCTGAAGGTCCAACTTTAAATAACTGGAGGGAGACTGTGCCTTTCACAGGTCCACCTTGGACTCAGGCTGAAAGTACAGCTATGCATATTTGGAGAGAGACTGTGCCTTTCACAGCCCCACCATGGACTTCAGACAAAGGTTCAGCTATAAACAACTGGAGAGAGATGTTGCCTTTCACAGTCCCACTGTGGACACAGGCTGAGGGTCCAGCTGTAAATACCTGGAGTGAGACTGTCCCTTTTACAGTCCCAACATGGACTCTGGCTGAAAGGCCAGCTGTAAATTCCTGGAGAGAGAGTGTGCCTTTTACAGTCCCACCATGGACTCAGGATGAAAGTCCAGGTGTAATTACCTGGGGAGAGACTGTCCCATTCAATGCCCCACCAAGGACACAGTTTGAAAGCCCAGATGTAAACACCTGGAGAGAGACTGTGCTTTTCACAGCCCCTTCATGGACTTGGACAGAACGTCCAGCTGTAAATTCCAGGAGAGAGACTGTGCCTTCTACAGTCCTATCTTGGACTCAGGATGAAAGTCCAGATGTAAACACCTGGAGAGAGACTGCCCCTTTCACAGTCCCAACATGGACTCTGGCTGAAAGACCAGTTGTAAAATCCTGGAGAGAGAATGTACCATTCACAACCCCACCATGGACACAGAGTAAAGGTCCAAATGTAAATACCTGGAGAGAAACTCTGCCTTTCACAGGTCCACCCTGGACTCATGCTGAAAGTGCAGCTATAAATAGTTGGAGAGAGACTGTGCCTTTCCCAGTCCCACTATGGACTCTAGATAAAGGCCCAGCTGTAAATAACTGGAAAGATGTGTTGACTGCCACAGCGCCACCATGTAAACAGTCTGAAGGTCCAGTTGTAAATTCCTGGAGAGAAACTGTGCCTTTTACAGTCCCACAACGGACTCAGGATGAAAGTCCAGGTGCAATTACCTGGGGAGAGACTGTAACACTCAGAGCCCCACCAAGAACAGACATTGAAAGTCCAGATGTAAACACCTGGAGAGAGACTGTGCCTTTCCCAGCCCTACCATGGACACAGGCTGAAGGTCCAGCTGTAAATTCCTGGAGAAAGATTATGCCTTTTATAGTCCCACTGTGCACACAGGATGAAAGTCCAGGTTTAATTACCTGGGGAGAGACTGTCCCTTTGAGAGCCCCACCAAGGACACAGGCAGAAGGTCCAGATGTAAATACACAGAGAGTTACTGTGCCTTTTACAGGTCATCCTTGGGCTCAGGCTGAAAGTGCAGCTGTGCATACTGGGAGAGATACTGTGCCTTTCACAGCCTCACCATGGACTCAAGATGAAGACCCAGATGTAAATAGCTTCAGTGAGATGTTGAGATTCTCAGCACCATCATGGGCCCAGGCTAAAGGTTCAGCTTTAAATAATTGGAGGGAGATTTTACCTTTAATAAGCTTACCTTGGACTCAGGATGAAAATCCAGCTGTAAATACATGGAGAGAGACTGTGCCATTCATAGAGCCACCATGGACTCAGGCTGAACGTTCTGTTGCAAATAGCTGGGGAGAGACTGTGCCTTTCTCAGCTCAGCTATATATTCAGTCTGAAAGTCTAGCTGTTAAGAACTGGAGAGAGATGTTTCCTTTCACAGCCCCACCATGGACTCAGGATAAAAGTCCAGCTGTAAACACCTGGAGAGAGATGTTCCCTTTCACAGCTGCACCATGGACTCAGGATGAAAGTCCAGCCATAAATACCTGGAAAGAAACTGTGCCTTTCTCAGATTTACCATGGACTCAGGCTGAAAGGCCAGCTGTAAACACCTGGAGAGATACTGTGCCTTTCACATCCCCACCATGGACTCAGGCAGAAAGTCCAGCTGTAAACACCTGGAGAGAGATGTCTCCTTTCACAGCTGCACCATGGACTCAGGATGAAAGTCCAGCTGTAAACACCTGGAGAGAAACTGTGCCTTTCTCAGCTCTACCATGGACTCAGGATGAAAGTCCACCTGTAAACACCTGGAGAGAAACTGTGCTTTTCTCAGCTCTACCATGGACTCAGGCTGAAAGTCCAGTTGTAAATGCATGGACATGGAATGCCCCTTTTACAGCTCCACCATGGTCACACACTGAAAGTCCAGCTGTAAATACCTGGACAGAGGCCTGGATTTTCACAGCCCCTCCATGGATTCAGGCTGAAAATCCAGCTACAAATACCTGGAAAGTGAATGTGCATTACAAAGGGCCACCATGGACTCAGTCTGACTCTGCACCAGCAAACCCTTGGACATCAACTGAAAGTTTCACAATCGCATCATGGACTCATGGTGTAAAGCAAGTTTTAAATATTTGGACAGAGCCAATAGTTTCCACAGTCACACTATGGACTCAGGCTGAATATTCAACACCAAAATATTGGACAGATACAAAGGTCATTTACATAGTCACACAATTGACCCAGTGTCAGTTTCCAATAAATACTTTGACAGAATCTATAGGAGGCATAATCACACCTTGGACATCTGCTGAATCTCTAGCATTAAGTTCTTTCACACAAAATATTGTTGATACAGTCAAATTTTGGCCAGTGCTTAAAACTGAATCTAAGAAAAGGTGGAATCTGCCTCAAAGTGGTACACTCATGTTTTCACTAAATCCTCAAATTGATACTTTTGGATCCTTGAACCAAATTGAAAACCAAGACTCTCCTCTGTGGACACATCCTGAAATTGATAATGTCAATACAATGACCTTTCTTGAACCTGGAACACTCATATCACAGGTAGTACCTTTGTCCCAAGCAGCTAGATTCTGGCCCCAAACTGAAGCTGATATTAGCAAAACTTGGTTTGTATCCTCTGAAAAAATAAATTCCTGTGCCCAATCAGAGTCTCAAAGAATGAGTACCTCAACCCATTTTGGAGTGGGTAGAGTGAAGCCCTTGGCCCAACATGAAACTTCTAGAGTCATGTCATTTCTTCAGGTTGAAATTGGTATAGTCCGCCCTTGGAACCAGCCTGAGGGAGATACAGTGAGGTTCTGGCCACTTTCTGAAACTGAGGATGTAAGAAAATGGATCCAAACTGGAGCCAGTATAGTCAACTCTTGGACTCAGCCGAGAAGTAGTATAGTCAGAGCTTGGACCCAAGTTGAATCTGAACTAGTCAGACCTTGGACACAATCTAAAACTAATGCAATTACACTATTGACCCAGGCTGATACTTTCACACCTTGGTTCCAAACTCAAATTAATGCAATAAGAGAAGGGCCCCAAACTCAATCTCAAATAGTTACTACTTGGATCCAAACACAGTTGAAAATAGTTCATCCCTGGATTCAACCTAAAAGTGATTCAATCAGAATTTGGATCCAGCCTTGGATTCAAGCTGAAACCCACACAGTCGGACTCTATTATGAAAGTGATATAAGAAAATCATTAACCTCATCTGAATCTCAGGCAGTCACATTTTGGTCACTGAATCAAAATTCAGTTATGACTTCATTTAACTTGGAATCTCAGATAACATGTTCCTGGGTCCAAAATGAATTTAACACAATCAGTCCTTGGAATCAATATGAAACTAATTCTGTTGGATCCTGGATCAAGTCTGAAACTGGTACATGTCAACCCTGGCTCCATACTGAATCTTCTACAATCTTACCATGGACCCAATATGAAACTTTAGAGATCTACCCTTCAAACCAACCTGAGACTGATACATCAATAGCGCACTTGTTCCAGCCCGAAATCGATCCAATTAATACTTGGAATCAGCCTGAAGTAGATACAATCAGATTCTGGACCCAAGTTGCAACAGAAATAATTCCAGTTTGGACCCAGATTGGAAATCAGGTGCTTGAACCTCCCAACTTTTCTGAAGCTGGCATAGACATACCTTGGTTACAAACTAAAACTGATGTAAGTAGACCCTGGATTCAACCTGACTTTCAGTCAGTCCATCCTTGGACCCAGTCTGGATTTGATATAATTAACCTCTGGTCTCACCCAAAAGCTGCTGTAAATCAACCCTGGACATATGTTCAAACACAGGCAATCGGACCCAGGATCAAGGTGGAAGCCAATACAATCAAATCATTGTTTCATGTCCAAGTGAAAAAAGTCAGACTGGGGATTCCTTCTGAGTCTCAAATATTGAGTTTCTGGATGCAGCCTGACATTAGTAGAGTTAATGCTTGGATCCAACCAGCAACCCAGGCAGTCAATCCTGCGTTTCACCCTAAAACTGGTAATGTTGCATCCCTGGCTATTCATAAACCTGAAACAGTCAGAATATGGATCCAGCCTGAAACAGAAGTAAGGCCTGGCATCATTTATAAAGCTGATATAATCACATCATTTGCTTCTCCTGAAGTTGAACAAGATGCAGCAATTAGTCACTTTGATTCCTGGTCTAACTATGTAACATTTTTACCAATAGAAACTGTTCCTTCCCTACATGAGTATTTTGCAGCTTTGTCAACTGAAATAGCTGCAGTAGAAAGTCAAGGTCAAACAAATTCTGTCCAACCCAGTGAAATCACAAATATTCTCTATCTTACACTTTCAAGCACACGACTTCCTGGAGGAGCTGGTTACCTGAACTTTGGCAATGAATTACAAATTACCAAAACAAAAGGAAGCCCTGATGTCCCATCTAGTTCTCTCAACCCACTTTATCCATCTTTTTCCTTTCCTGTTCCTTGTTTTTTCCCACCTTCATGTTCTTTGCCCCTTACCTGTTCAGTCTTTTCTTCTTGCACATTTTCTTCACCCTGTACTTTTCTTTCTTGCTCAATTTGTCCTCTTATGGCCTTCTCTCCTGTTCTTCCCTTACCTGCTTCTGACAGTTCTTTCCAGAAACCATCTTCCTCAAAATTTACTGTAGACACCATTCTTTCCCATACTTTTTCATCCTTGCATGCTGCTCCAGCCAATCTTTTAACAAAGCAACCATCTCTGATGCCTGGATCTCAATCTGGAACCAAGTCTAATCAGCCTGAACAAGATCTTCTTAAGTATTCTGAACTCAATATTTCCCTTGCTGAGTGTCGTCTGGGTGTGGTCTGGAAAGAGAGTCTCCAGGCTTTCTCACTCTTCAAGACAGCTGTTATTTCTCATGAAGTTACAGAGTGTGGATTACGCCCTGGCCTTGTTCCACACTGTCCCAACTGCTGGGAGGCTGAAGTGGGTGAATTCCCTTGGATGGTTTCTGTGCAACTCTCTTTCTCCCATTTCTGTGCTGGTTCTATCCTGAATGAACAGTGGATTCTCACTACAGCTAGATGTGCAAATTTCATAAAAAACTCAGAAGCAGTGGCCCATGTTCAGGTGGGGTTTATTGATTTTCAAGATCCTGCTCAAGCTCAAACTGTAGGCATTCATCGTGCCATGCCCTACCTGGGTCCTAGAGGACCGCTGGGACCTGGTCTAGTCTTCTTGAAGAAACCATTACATTTTCAACCTCTGGTTCTTCCTATCTGCCTGGAGGAGAACATAGAGCAAGAGAAAAATATACAACTATATGACTGCTGGCTACCCAGCTGGTCCCTCATGAGAGGAAGTCCTGGAATTTTGCAAAAAAGGCACCTGAGCATCCTGCAAGTCATCACATGTGCCCAGTTTTGGCCCAAACTGAATGAATTTACTTTCTGTGTGGAAGCCAAGAAAGCTATGGGGGAGGCTGGCTGTAAGGGTGATCTGGGGGCACCTCTTGTGTGTCATCTTCAACAAAAAGACATTTGGGTGCAGGTGGGAATCTTGACTCACTTTGATGAACACTGCACAAAGCCCTACGTCTTCAGCCAAGTGAGCCCTTTCCTTTACTGGCTCCAGGGAGTTACACGACCCAGCCATGCACCCTGGTCTAAGCAAGGGCCTATGACCACCTCTGCTTCCATCTCCCTTTCAGTCTCTACCTCTATGAATGCCTCAGCTTTTACCTCCACACCTGCTTCTGTCTGGCCACATTTCATCTCTCTACCACAGCCTCAGACTTTGGCAGATCAAATTTCTCTGAGATATGCCATGCCTTGGCAGGCCATGATCATCAGTTGCAGTAGTCAAATTTGCAGTGGTTCCATTGTTAGCAGCTCTTGGGTACTCACTGCGGCCCACTGTGTCAGGAATATGAAACCAGAGGACACAGCTGTAATATTAGGCCCGAGGCACCCTGGGGCACCTCTAAGAATTGTTAAGGTCTCTACCATTCTTCTGCATGAGAGATTCCGATTAGTGAGTGGGGCAGCAAGAAATGATCTAGCATTGCTGCTCCTTCAAGAGGTCCAGACTCCCATTCAGCTTTTAGCACCATTGGGTCATCTGAAGAATCTGAACAGCTCAGAATGTTGGCTCTCTGGGCCACGAATTCTTAAGCCAGGAGAAACAGATGAAAATCCAGAAATGTTACAGATGCAGGTGATAGAAGCTTCAAATTGTGCCCACCTTTACCCTGATATAGGCAGTTCTATTGTGTGCTTCATTACACAAGACAAAGACTCTGACACAAGTGTGGAACCAGTGAGTCCAGGCAGTGCTGTCATGTGCAGACCAATGTCTAGGAATGGAAGCTGGAGACAGATAGGCCTCACTAGTTTGAAGGCACTGGCTACCATTGTAAGCCCCCACTTCTCATGGATATTATCCACTTCAGCAAAGGCAGGGCATCCATTAAACCATGCACTCATGCCTTGGATGGAAAAGCCTAAGTCCTCTAGTCTCATAAAACAGGCAACCACCCTGCCATTTTCTTCAATAATAATTTTTATACTGCAAAGAATTTTATAACTCACTACAAATATAAGGCAGGGCTAATCTATTCAAACTATAAGTAATAAAAATGTTAAACAACATTAAAGAAAATTAAGACCCTGTATAACCTAG

>Tasmanian Devil, Sarcophilus harrisii (BK059513)

TGACCCCCCGGCGAGCAGCTTACACTGTGACTCCCTGGGCTCGCTCTCACATCCCACGGCCATCTGGATCCCAGCCTCATCTGGCAGCTGATGCGGGCAAACCCCAAACGCCGGCAACTGTCAGATCTCAGACTCACTCGGCAACTGATGTTCTCGGAGCCTGGCTTCTTCCGTTCCAGACGGTTCCGGCTGTTCTCAAGTCCAACACATTTGGAGCCCGGGATCAGCTGGCATATGCTCAAACCGGACCCTGGATGGCCCCCGACTCAGAAGTGGCCCCAGAAACAGTGGGGCCTTGGGTTCTCCAGACGCCAGCAGCTGGCCAACTGCCCGGGTGGGCTCCTGGTGGGGCTGGGCCCTGGGCTCTGGCTGACACAGAAGTACCTGGTCCTTGGCCTCAGCACGCAGCTGAGCCCCAGTCAGAGGGCAGTCCAGGAGCCCGCCTTGCTGCACCCCTGCCCCAGCCAGTGGCTGAAGCAGCTGGGTTTTGGACCGTTCCGGCCCCAATCGTCAAAGCCATCGTCCCCTGGCCTGGACCCGTGAGCGAGATGGCCTCTGAAGCCATCGTCCCCTGGGCCGGCCCAGCTCCCGCTATGGTACAGGGACTAGATCTGAGGAACTTGCCAGGAACTGCCAGCGGTGACCGCCTTCCGCCTTCCCTGGCTCTGTTCTCCGGCTCCCTCACCTACCCGGGCCCATTCCTCTCCCCAGTGTTGCCCCCTTCGTCCCAGTCAGTGCCCCCAGCCCCTGCTCACTCACAGCCTCCTCTCTTTTCCGGAGCTCTACAGGCATTGGCCTGGGGAACCTCGGGCCCATCGGTGCCCAAATCCCAGCCCGATGGCGACTCCTCAGGGGCCGGCCCTTCCAGCACTGAGGGCCGACTGGCCCCTTCGTCCTTTCCAACCTCCTCTGCTCCCTCCCCCTTTCCCTCCTCGGCCCGTCTCTCCTCTCCGGCGGAACCCTCTAGGTCCAGGAGCCCTGTGCCCGACCACCATCGGATGCAGCAGGAGCAACCCCTCAGGGTCACCTTGGACCAGTGTCGCTTGGGCCTGGGCTGGAGGAGCAGTTTCCAAGCCTATTGGCTCTTCCAGACGGCCTTGTCCTCTGAGCAGGAGCTAGACGAGTGCGGCATGCGTCCGGGCTTCGCGGCTCGGTGCCCCACCTGCTGGGAGGCCGAGGAAGGCGAGTTCCCCTGGGTGGTCTCCTTGCAGTTCTCCCTGTCCCACTTCTGTTCGGGCTCCATCCTGAATGAATGGTGGGTCCTGACCACCGCCAGCTGCGCCAACTTCATACGGAATTCAGAAACCCTGGCCCAGGTCCAGGCCGGCGTCACCGACTTGGAAGACCAAGTTCGGGCCCAGCTCGTGGGCATCCACCGGGTCTTGCCGTACTTTGGGCTCGAAGGGCCCACGGGCTTGGGCCTGATCCTGCTCAAGGAGCCCTTGCGCTTCCAGCCCCGAGCGCTGGCCGTGTGCCTCGAGGAGCCCTCCAAGAGGCCGCCAATACAGCCTCGGCGGAACCTCTACGACTGCTGGGTCCCCGGCTGGACCCTGATNNNNNGCAACCTGGTGACTATGCAGAAGCGGCCACTGGACATGGTGGAGGTCAGCAACTGTGCCCGCTTCTGGCCCATTGAGAGCTCCATGACCTTCTGTGTGGAGGCCAAGAAGGTGACGGGCCAGAGCAGCTGCAAGGGAGACCTGGGAGCCCCGCTGATGTGCCGCTCAAAGCCACACCCAGAGGACACCCCTTGGATCCAGATGGGGGTCCTCACCGCTTTTGACGAGACCTGCACTCGGCCCTATGTCTTCAGCCGCATCCACCCCTTCAGCCTTTGGCTCAGGGCCTCCACGAAATCTCAGCACCCCCCCTGGGTCCGGACTGCCCCCAGGCCCACCCTGTCTTCCCTGCTCCAACCTGAAGCCCTGGTGAATCGGATTTCTCTGCGCTTTGCCATGCCCTGGCAAGCACTGATCGTGACCTGTGACAGCAGACTTTGTGGCGGATCCATCCTCAGCCCTTCTTGGATCCTCACCTCAGCCCACTGTATCCGGCACATGAGGACAGAGAATATGGCGGTATTTCTGGGGCTGCCCCAGCCCGGGGGCAACATGACCGTCGCCCGCGTCTCCAGCGGGGTCCTGCATGAGCGCTACCAGTTTGCGGATGGGGTCCCCTGGAATGACCTGGCCCTCCTCCTCCTGCAGAAGCCCCTGGGTTCCAGCCAGCTATTGGCCCCCATCAGCCACGTGGATGATGTGAACAAAGCTGAGTGCTGGGTCACAGGGCCCCGAGAACTCCGAGAGGGTGAAACAGACCAGAGCCCACAAGCCCTGCACGTTCAGGTGAAGGATGCTCGGTCCTGTGCCCGCCACCTCCCTGGCAGCATCAGGAACTCGGTGCTATGCCTGGCTCCCTGGGGGCCAGAATACCAAGTGGCTTTGGATCTCATGGGTCCAGGCAGTGCCCTGTTGTGCCACTCACGGGGTAAGAATGGAACCTGGAGGCAGACTGGGCTCACCAGCATCAGATCTCTTACCTTCCTCCTGGCTCCTTACTTCCCATGGATCTCCAAGACTACTTCTGCCCAGGCCGACCACATCTGGCTCAATCAGTCAGTGGGCAAGCCCGTGTCTGCCACGTCTGCAGCAGTGGCAGAGCCCAGGGGCCTTCTGGGCCTCACCTTACTCTTGACCCTATGGCTGCAGTTCCTGAGAGCATAGGACCGTTTCCCCACACCGACTACTGCACCCATCCAGCCGTACCCCTGCAGCCTCACCCTGGTCCAAGCCTCCATGGGGTTCAGCCTCTCTACAGCCCAAGGGGAGAAGACCATTCCCCTGTCCTCCCTTATCTTCTCCTTCCAATATCTATGAGAGGCTCCTTCTACCAAGAGAGCAAGATCAAGGCTGTACCAGAGACATACCAGGAGGGAACCCCGAGTGTATTTTGATGACCCCCTTTCCCCTACCAAAAATAAAGCCACACCCTCTGTGAAACATCTGATTTCTTGTGTGCTGGTTCCAAGGTCACATTGAGCAATTTCTGGTGCCCAAGAACTCGGTCTGTGAAGGGCAGAGAGCCACACAAACACACCCATCACCCTCAATAACAGCCCTCCACAGTCATCAAAAACCTTTTCGAGGCTAAATTGAACAGCCTCTTCTC

>Dog, Canis lupus familiaris (BK059504)

AACTTCTGGTCACATGGCAGACCCAGGGTAGATATCCAGCATTAAATCCATGGATACAGTCAGAAGCTGACACATTTACATCATGGTCTCAGGCTGAGTCTCCAGCAGTAAATCCACAGGCACAGACTGAAAGTGACACAATCACAACATGGACCCAGGCTAAATCTCCTGCAGCATATCTCTGGACACAGCCTGAAAATGTCACGGTCACACTGTGGATCCAGGCTGAATCTCTTGTAGTAAATCCCTGGACACAGCATGAAAATAACACAGGCACACAATGGACCCAGAATCAATCTTCAGAAGAAAACACCTGGGCAGAGGCTGTTTCTGAAACTGTCATACCATGGAAAATGGGATTTTTTCCAAATATGAAACCGTGGATAGAGATGATATCTGATATAGTTACTAAATCTCAATTTTCTGAAGTAAAATTTTGTACAGTGCCAGGGTCCAAATTGGACACTGATGTAAGTACAGTAAAAAAGTGGACTCGGTCTGAATCTCCACCCTTAATTCCCTGGACAGAACCTCTAGCTTCCATAGTACCACTATGGACTGAATCTCTAGCTATAAATCACTGGACGCAGCCTATACCTGATACAATAACAAAATGGACACAGACTGAATCTCCATCAGTAAATACCATTCAATTTCCAGCAGTAAATCCATGGACACAGTCTGAATTACCAAGAGTACATTTCTTGACAGAGTACAAATCTCCAGCCCTAAAACCATCAATTGAGCCCGAGGCTAGTATAGTTACATTGTTGACTCAGACTGAATCTCCAGCAGTAAATCCATGGATAGAGCCTGTTGCTTCCATAGTCATGCCATGGATACAAGCTGAACACCCAACAATAACCCCAATGACACAGGCTGTAGCTGAAACAATTATCCTGTGGACTCAGAATGAAAATCTAGCATTAAATCCCTGGACAAAGTCTGTTGCAGATACAGTCACACTTTTGACCCTGGATGAATATTCAAGAGCAAAACTTTGGACACCTATATTAGATTCCTCAAATATATTTTGGGCCCAAGCCAAAAATTCAGCAATAAATCCTTGGTCTGATTTTGAAATACCCTCACCATTGACCCAATTACAATCTGTAGTGGTTAAACCATGGACACAATTTGAGAGTGACACAGTCACACCATTAATGAAGGTTCAGGATCTTGCTGTAAATCCCTGGTCAGAGATTATTGCTTCCAGAGTCACAACACAGACTCAGTCTGTATCTCCAGCAGTAAACCCCTGGATGGAAGTTGATGCAACCAGACTCCCATCATGGACCCAAACTGCAACTCCGGCAGTAACAACCTGGACAGAGGCTGATGTATCCAGAGTACTGCCATGGACCCAGTCTCTACCTCCAGTAGTAAATCCATGGACAGAGGTTTATGCATCCAGAATCACACCATGGACCCAAGCAGTAGCTCCAACAGTAAATCCCCAGACAGAGACTGTCAGTTCCAGATTCATGCCATGGACCCAGGCTGTCCCTCCAGCAGTAAATCTGTGGACAGAAACTAATGCATCCAGAGCTATACCATGGACACAGGATGTATCTCCATCAGTAAATCCCTGGACAGAGGCTGTCAGTTCCAGATTCATGGCATGGATTCCAACTGTACCTCTAGCAGTAAATTCCTGGACAGAGGCTGTTGGCTCCAGATTCATGCCATGGACCCAAGCTGTACCTCCAGCAGGAAATCCCTGGACAGAGACTGTTGGTTCTAGATTCATGCAATGGACCCAAGATATACCTCCAGCAGTAAATCCAGGGACAGAGGTTGTTGGTTCCAGATTCATGACATGGACTCAACCTGTAATTCCAGCAATAAATCCAGGGACAGAGATTGTTGGTTCCAGATTAATGACATGGACCCAAGCTGTACCTCCAGCAGGAAATCCCTGGACAGAGACTGTTGGTTCTAGATTCATGCAATGGACCCAAGATATACTTCCAGCAGTAAATTCCTGGGCAGAGGCTATTAGTTCCAGATTCATGACATGGACTCAACCTGTAATTCCAGCAGTAAATCCAGGGACAGAGGTTGTTGGTTCCAGATTCATGACATGGATTCAATCTCTAATTACAGCAGTAAATCCCTGGACAGAGGCTGATACATCCAGATTCATGCCATGGACCCAAGCTGTACCTCCAGGAGTAAATCCCTGGGCAGAGGTAAATGCATCCAGAGTCCTGCCATGGACCCAAGCTGTCTTTCTGGAAGGAAATCCCTGGACAGAGGCTGATGCATCCAGAGTCCCAGCATGGATGCATGTTGTACCTCTAGCAGTAAATCCATGGACAGAGGCTGTCAGTTCAAGAGACATGCTATGGAACCAACCTGTACTTCCAGCAGTAAAACCCCAGACAGAGGCTGTAACTTCCAGAGTTTCACCATGGACACAAGCTATAACTCCACCAGTAAATACCTGGACAGAGGCTGTTGTATCCAAAGTCATAACAGGGAGTCAACCCGTACTTCCAGCTGGAAATCCCTGGACAGAGGATGATGTATCCAGAGTCATGCCATGGACCAAAGCTTTACATCCAGCAGGAAATCAGGCTGTTGGTTCCAGATTCATGCCATTGACCCGATATATACCTTCAGCATTAACTCCATGGACAGAGGCTGTCACTTTCAGAGTCTCACCATGGACCCAAATTGTACCTTTAGCAGTAAATCCCTGGACAGAGACAATAACTTCAACAGGCATGCCATGGACCCAACCTGTACCTCCAGCAGTAATTCCCTGGGCAGAAGCTGTACCTCCCAGAGTCCCGCCATGGACCAGAGCTGTATTTCCAGCAATAAATCCCTGGACAGAGGCTGATGCATCCAAGGTCATGCCATGGACCCAGTCTGAACCTCCAACATTAAATCCCTGGACAGATATTTATGCATCCAGAGTCATGCCATGGAATCCACCTGTACCTCCAGCAGTAAATCCCTGGACAGAGGCTGTTGGTTCCAGATTCATGCCATGGACCCAGGCTGTCCTTCCAGTAGTAAATCACTGGTCAGAGGCTGATGTATCCAGAGTTCCAGCATGGACCCAAGCTGTACTTCTAACTCTAAATCTCTGGACAGAGGCGGTGGGTTCCAGATTCATATCATGGACTCAAACAGCATCTCCAACAGTAAATCCCTGGACAGAGGCTGTTGGTTCCAGATTCATGCCATGGACCCAACCAGTAACTCCAGCAGTAACTCCCTGGACAGAGGCTGATGCATCCAGAGTCTTGCCACGGACCTTATCTGTACCTCTGGCTGTGAATCTCTTGACAAATGTTTCTGGTTCCAGATTCATGCCATGGACTCATCCTGTAACTCCAGCAGTAAATCTCTGGAAAGAGGATTTCAGTTCCAGATTGATGCCATGGAACCAAGCTGTACCTCCAGTGGTAAATCCCTGGACAGAGTCTGTCACTTCCAGAATCACACCATGGACTGAACCTGTAGCTCCAATAGTAAATCCTTGGTCAGAGGCTGTCGGTTCCAGATTCATGCCAAGGACCCAAACTGTACCTCCAGCAGGAAATCCCTGGATAGATGCTGGCACTTCCAGAGTCACTCCATGGACCCAAGCTGTACCAGCCACAGGATATCCTTGGACAGACACTGATGCATCCAGAATCACACCATTGAACCAAGCTGTAACTCTAGGAGTAAATCCCTGGGCAGAGCCTATTGCTTCCAAAGTCATACCATGGACACAAGGTATATCTTCAGCAGTAAATCCTTGGGCAGAGGATGTTCCTTCCAGAGTCATGCCATGGACCCAATCTGTACATCCAGGAGTAATTCCCTGGACAGAGACTGATGCATTCAGATTCATATGGAACCAAATTGTACCTCCACTAGTAAATTCTTGGTCAGAGGCTGATGTATCCAGAGTCATGCCATGGACCCAAGCTGTACCTCCAACAATAAATCCCTGGACAGAGGCTGATTCATCCAGAGGCATGCCATGGGCCCAATTGGTACCTCCAGCAGTAAATCCCTGGACAGAGGCTGATGCATCCAGAGTCATATCATGGACCCAAGTTATACCTCCTGCAGGAAATCCCTGGACAGAGGCTGTTAGTTCCAGATTCATGCCATGGACTCAACCTATACCTCAAGCAGTAAATTTCTGGACAGAAGTTGGTGCATCTAGGGTCCCACCATGGTCCCAACCTGTACCTCCAACAGTAAATCCTTGGACAGAGGGTGCTGCATCCAGAATCACTCCATGGAATCAAGGTGTAACTCTAGCAGTAAATCCCTGGACAGAGGCCATTGGTTCCAGATTCATGTCACTGACCCAACTTGTGCCTTCAGCAGTAAATGCCTGGACAGATACTGATATATCTAGATTCATGTCATGGATTCAACCTGTACCTACCTTAGTAAATCCCTGGATAGAAGCTGTTGGTTCCAAATTCGTGCCATGGACCCAACCAGTACATCCAACAGTAAATCCCTGGTCAGAGGCTGATGCATCCAGATTCATGCCATGGACCCAGCTTGTACCTCTAACAGTAAATCCCTGGTCAGAGGCTGATGCATCCAGATTCACGCCATGGACCCAACCTGTACCTCCAGCAGTAAACACCTGGACATATATGAATGTATCTAGAATCATTCCATGGTCCCAAGATGTGCCTTCAGCAGTTAATCCCTGGACAGATGCTGTTGCTTCCAGATTCAATCCATGGACCCAAACTGTATCTCCAGCAGTTAATCCCTGGACAGAGGCTATTGGTTCCAGATTCATGCCATGGACCCAACTTGTACCTCTACCAGCAAATCACTGGTCGGAGACTGATGCATCCAGAGTCATGGCATGGACTCAACCTTTATTTCTAGCAGTAAATCTCTGGAGAGAGACTGATGCATCCAGATTCACACCAAGGACCCAACCTGTACCTCCAGCAGTAAATCCCTGGACAGATCCCAATGGATCTAGAGTTATGCAATGGACCCAACCTCTACTTCCTGTAGCAAATACATGGACAGAAGCTGATGCATCCAGAGTCATGCCATGGACCCAACCTTTACCTCTAGCAGTAAGTCCCTGGACAGAGACTATTGGTTCCAGATTCACTCCATGGACCCAACCTTTACCTCCAGTAGTAAATCCTTGGACAGAGGCTGATGCATCTAGAGTCACACCATGGATTAAACCTTTACTTATAGGAGTAAATCCCTGGACAGATCCTGATGTATCCAGAGTCACACCATGGACTTATCCTTTACTTCTTGCAGTAAATCCTTGGAGAGAAACTGGTACATCCAGATTCATGCCATGGACCCTACCTGCACCTCTAACAGTAAATCCCTGGTCAGAAATTGATGCATCCAGAGTTATGCCATGGACCCAACCTATACCTCCAGCAATAAATCCCTGGATAGTGACAGTTGTTTTTACCTTAACACCATGGACCCTGGATGCCTCTCTCCTAAATCCTTTGGCAGAGACTAAGGCTTCCTCAGTGGGAATATGGACTCAGAACAAATATTCAGTAATTAAAACTTGGACAAAATCTGAAGTTTCCACACTTACATCCTGGACAGATACTCAGACAGAATATCAAGCAGTAAATTCTTATATATCTTATATATCAAGTGTAACTGACACAGTCACATTTTGGACAATGCCAAAATATGAATCTAAGGAAACTTTGATACTGCCTGAAGCTCATATATTTAGTATATCATTGCATCCTCAAAGTGATACTCAATCCTTGATCCAAGTAAAAAATATAGCATCTCTTCTGTCAATATACCCTGGAATTAATAGTGTCAATACATGGTCTTTGCCTAAATTTGAAACAGTGGCATCATGGATAGTGCCTTTGCCTCAAGCAGCCAGATTTGTGCCCCTACCTGAAGCTGATATTAATAGAAATTGGTTTAAAATGGAAACAGAAACATTAAGAAATTGGACCCATTCAGAATCTCAAAGAGTGAGCACTTTGGCACAGTATGAAGCTAGTATGAAGCCCTTGGCCCAACATGAAAATTCTACAGTCATATCATGGATTCCAACTGAAACTGGTATATTTCACCCCAGGAATAAATCTGAAAGGGACAAAGTAAGAACCTGGACCCTTTCTGAAGGTAATGCTTTGCGACCATGGATTCAGATTGAAGCTAGCATTTTCAACCTCTGGACTCAGTCTAAAAGTAGTACAGTCACATCCTGGACCCAGCCTGAGTTTCAGGCAGTCTTTACCAGGACTGAAGGACTTATAGGTACATTTTGGTCCATGAATAAAAATGATGCAGTTGGGCCTTGGTCCCAGCTTGAATCTCAAATGACATCTTTCTGGACCCAAAGTGACATAACCAGCTCTTGGACTCAGTATAAAACTAGTACATTCATATCCAGGACCAAGCTTGAAATCAGTACACTGCAACCCTGGATTCAGTTTGAAACTGCTGCAATTAAATCATGGATTCAGTCTGAAAATGTAGAAATATACCCTTTGACCCAGACAAAAGCTGATACAGTAATAAGACCTTGGTTGCAAATTCAAATAGATTCAATACAACCTTGGAATCAACCTGAAACTAATACAATTAGACCATGGACTCAACTTGAAACTGAAGTAATCCAAATTTGGACCCCCACAGAAAATCAAGTAGTAAAACCTCCAACCTTACCTGAAATTGATACAATTACATCTTGGTTACAGACTCAAAGTGATACAACTAGACCCTGGATTAAATCTGACTCCCAGTCTGTCAGTTCCTGGGGTCATGCTGACATAGGCATAGTTCAGGATTGGATTCAGCAAAGAGCCACATTTATAACAAAAGAGACTTCTCTGATGCCTGGATTTCAATCTGGAACCAAGTCTAATCAGCCTGAACAAGATCATACCAACTATTCAGAACTCAATATTTCTTTGGCTGAGTGTCACCTGGGTGTGATCTGGAAAGAGACTATCCAGGATTTCTGGCTCTTCAAGACAGCTGTTATTTCTCATGAAACCACAGAGTGTGGATTACGACCTGGCCTTGCTCCCCACTGTCCAAACTGTTGGGAAGCAGAAGTGGGTGAATTTCCCTGGATGGTTTCTGTGCAACTCTCTTTCTCCCATTTCTGTGCTGGCTCTATACTGAATGAACAGTGGATCCTTACCACAGCTAGATGTGCAAATTTCATAAAAAACTCAGAAGCACTAGCCCTGGTCCAAGTGGGGCTTGTTGATCTTCAAGAGCCTGCTCAAGCTCAAACTGTAGGCATTCACCGTGCCATGCCCTACCTAGGTCCCAAGGGACCTCTAGGCCCTGGGCTAATCTTCCTGAAGCAGCCACTACACTTTCAACCATTGGTGCTTCCTATCTGCCTGAAGGAGAATGTGGAGCAAGAGAAAAGTATACAGCTTTATGACTGCTGGCTACCCAGTTGGTCCCTCATGAGAGGAAGTCCTGGGATTCTGCAAAAAAGGCACCTAAGTATACTGCAAGTCAGCACATGTGCCCAGTTTTGGCCCAAGCTGAATGAATTCACTTTCTGTGTGGAAGCCAAGAAAGCTATGGGGGAGGCTGGCTGTAAGGGTGACTTAGGGGCACCTCTGGTATGCCATCTACATCAAAAGGATACATGGGTACAGGTGGGAATTTTGAGTCACTTCGATGAACATTGCACAAAGCCCTACGTCTTCAGTCAAGTGAGCCCTTTCCTTTTTTGGCTTCAGGGAGTTACACGGCCCAGCCATGCACCATGGTCCCAGCAAGGGGCCATGACTACTTCTGCTTCCATCTCCCTTTCAGTCTCCACTTCTACGAACATCTCGGCTTTTACTCCCACTCCTGCTTCTATTCGGCCACAGTTCATCTCTCTGCCACAGCCTCAGACTTTGGCAGATCGAATTTCTCTACAATATGCCATGCCTTGGCAGGCCATGATAATCAGTTGTGGCAGTGAAATTTGCAGTGGTTCCATTGTTAGCAGCTCTTGGGTTCTCACTGCTGCTCATTGTGTCAGGAACATGAATCCTGAAGACACTGCTGTAATAATGGGCCTAAGGCATCCTGGTGTACCTCTGAGAGTTGTTAAGGTGTCTAACATTCTACTGCATGAGAGATTTCGGATGGTGAGTGGGGCAGCAAGAAATGATCTAGCACTGCTGCTCCTTCAAGAGGTCCAGACTCCCATTCAGCTTTTAGCACCATTGGGACATCTAAAGAACTTGAATAGCTCAGAATGTTGGCTTTCTGGGCCAAGAGTTCTCAAACCAGGAGAGACAGATGAGAGTCCAGAAATGTTACAGATGCAGGTAATGGGAGCTTCAAGCTGTGCCCACCTCTATCCTGACATTGGCAGTTCTATTGTCTGCTTCATTACTCAGGAGAAAATCTCTGATAAAAATGTGGAACCAGTGACTCCAGGCAGTGCTGTTATGTGTAGACCAATATCTGGAAATGGCAGCTGGAGACAGATAGGCATCACCAGTCTAAAGGCACTTGCTACCATTGTCAGCCCACACTTCTCCTGGATATTATCCACATCAGCAAAAGCAGGTCATCCCCTAAACCAGGCCCTCATGCCTTGGGCAGAGAAGCCAAAGTCATCTAGTCTTTATGAAGAGACAACAACACTACAATTTTCTTTATTAATGATTATTACAGTCCAGAGTCTGTTGTAAACCAGTGCTTATAATGATAACTACTACTTTGGGCATTTTGTAATTTAAAAAGATAAAATATCAAATTTTAAAAATTAAAAAATATCAAATTAAAAAAAAA

>Echidna, Tachyglossus aculeatus (BK059516)

ATCTCCAGCTGCGGGTTGTCCTGGGAGACATCCTCAGAACAGAGGTGGGAGTGACCTCACTGTTGCCTCAGTGACATCAGACATTCCTGGAGACCCGTAGGAACGCAGAAGCTCTTCTGAGCTGAGGTCTCCCGTTATCCCAGAGTGGCCCACCATCCTACGATGACTTGTTGCGCCCTTCCTTGCCTGCCACCTGAGCAAGGATGAGACCCCTCCCTTGCCTCTGCCATCTTCTGTGGTTGGTGTTCTGTTGCGGTGGCTCCTTTACAGTCTACAACGTCCACGAGCAACGGCCGAGCCGGCCTGCCCCGCGGAAGTCCCAAGGTACCAGAGAGTTTCCGTGGCTGGTGACAGTGGCTGGGACTTGCCAGGGAATAAGCCTCGGCCGCTGGTGGATCCTGACCGCCACCAGCTGCCTGCTGAAGACGAAGTCCCCGCGTCTGGAGGTTATGGGAGGTCCTGGCCCAAGAAGCAGCTTACAGGGAAGCCAAGTTTGCCTGCATCCCAGTTTTAACCCCCAATCCTGGGAAGGGCCAGCAGTAGCTGACCTAGGACTAATCCTCCTGAAGGAGCCCGCAGAACTGCCTAGGGACGAGCTGTGGCTCTCCAGGATTCCAGGCAACCTCCAGAGGAATTGCCTTCATTGCAAGGAAAGGCAGTGTCAGGTGTACCAGCGCCAGGAAACAACTTCAGATAATCTGCGAAATCGGGGTGAGGCGGTCACGACGATCCCCGTGAGGCTGCTGCGTTCCTCGGCGTGCCGCCGACATGGGGTCCGTATGGAGGAGGACAAAAGTCTCTGCATCAAGAGCAGAGACTATCAAAAGTCCGACTGCCAGATGCAGCCGGGCAGCCCAGTCCTCTGCCTCTTTGGGAGCCGCTGGGAGCTGGTTGGCCTCATCGGGGCCAGCCCAGAGAGCTGCTACAGCCCAACCCTCTCCATCAGGACCGCTCCCTATTCCTCCTGGCTGAGGCAGCACGTCAAGGCAGCCGCCCCCCTCCCCTGCAGCCCTCACCCGCCAATGGAGCCAGCCACCCCTGGAGACTCCCCTCTTAGCTCCTCCTCGGAACCGGCCTCCACGTTCCAGCCGACGCACCCTTGGCAATTGGGAAATGAAAACCCGGACGTCTCTCAAGTCCCCCGCCGTTCCCGTCCAGGGCCACCGGCTCCTCCGACCCCTGGGCCTACGGATGCCCCAGCTTCGTCTTTCCTCAAGCCCGGGGGCGGCCTTTCTTTCCTTCCGGCCAACCACCTGCCCTCTGGTGACAGTTCGCCCATGGTAACGAACTGGGCTCCCCTCGTCGGCCAGACAGTAGGAGCCAGGTCTCCCTTCAGCGGCCCGACAGTCGTACGCTCAGTTCTCCCCACGGATCAGGCGGCCGCACTTTGGACTCCATCTGGAGGGCAGCCAGTCAGATCTTTGGTTACCTTTCCAGCGCAGTCAGGTGAACCGTGGACTTCGGCTAGACCCCCGGTTCCTGTCACTGCCCTGAGGCTGGCACCGTTGGCTTCGCGTGCCCCTGGAATGGCCGTACTCTCCACACCCTCAGCAGCCCAGATCATCGCACCGCCCTTGGCCAGCTGGACTCCCGCTAGGGCTACAACAATGGAACCCCTTCTGGCCTCAACAGCCGTGACCCCCCGATACCACTTCCCCAAAGTCTGGCCTCCTGCACCCACTTTCTACAGACTCCAAGCTAACGTCCACGGTCCAAGCAGCAGACCCATTTCGCCCTGGACGATCCACCCTGGGGGAGCACAACCTACCGGGGCCGGGTCATCCACTGTGGCTTTCCCCCAGCCCTCTCCCAGCCCCTTCGGTACGGAGACGTCGTCCAGGATTATTTCCCGTCTCCTGCCTTCTGAAAGTCCCCTGTCCAATATGTGGACCGGGGGTTTGGCCAAGCCTACGGTCTACTCTGATGACTACTTTCACGTGGCCTCCTCTTTCCCCACGGCTAAGCCCAAGACCTTGGGTGACCTACCCGCCTTTCCCCCAGTCTTTAGCACTCCTGATCCCCATTCCTTTCCTAGGCTTCCCCAGCAGGTGATGAGGCCCACTCCTCTCTATGGGCCAGATGCCCTTTCTTACGGCAGTTCCTTTCCCAGGGAGCCGGACTCCTCCCTGAAGCCGTCCTTTTTGGTCACCCCCCAAACCAGACCCTTGTCTCCCGTGGACTTCCCGTGGATCTCTGAAGTTGGGCCAGCATTCTATCATCACTCTCCTTTCTCAGCACCTTTCAGTCAAAACGTCCAGGCCGGTTTGTCCCCCTTATACCAGCTTCAACAGAAACAGAAGGCTGGGACCACCCTGGCCCAGTGCAGGCTAGCCCTGGCCTGGAAGATCAAGACAAGGGCCTTCAGCCTTTATCAAACCGCCGTGCCCATCAAGAAGTCCTTTGAGTGCGGAATGCGCCCAGGCTTTACCCATCGCTGCTCAGGATGCTTGGAGGCAGACAGGGGCGAGTTCCCCTGGATGGCGTCCATCCAGCTCACCCTTTATCACTTCTGCGCGGGCTCGATCCTGAACGAATGGTGGATTCTGACCACGGCCAAATGTGCCAGTTTAATAAAGAACTCGGAGGCCCTGGCCGTAGCTCAGGTGGGCGTCGTCAACCTTCAGGATCACGTCCAAGCCCAGGTGGTGAGCATTCACCACGCGATCCATCACCACAGCCCGCAGGGGCCCGTCGGTCTAGGCCTCATCCTCTTGCAGCAACCGTTGCGCCTCCAGCCCCTGGTCCTACCCATCTGCCTGGAGGACAGCGAGAAGCAGGAGAAACATTTGAAAATCTCCGACTGCTACCTGCCCGGCTGGAGTCTCATGAGGGGGGGGCCTGTAGCACTCCAGAAACGTCAACTCAGCATGCTCCGTCTCAGCGTCTGCTCGCGGTTCTGGCCCAAGCTCAATGAATTCACCTTCTGTATCGCGGCAGAGAAGGCAGTAGGCAAGGCAAGATGCCAGGGAGACCTGGGAGCACCCCTGCTCTGCAAAGAGAAGCAGAAAGAAGTGTGGGTACAGGTGGGGGTGCTCAGCAACTTTGACGAGCACTGCGTGAAGCCGTATGTCTTCATTCGGATAGCCCCTTACTTGTCGTGGCTAGAAAGTGTCACTCAGGATGACCCCCATGCTCCCTGGGGGGCTCAGACTGACACCTCCCTCCTCGTCTCCCTGCCGCACCCACGGACCCTCGTGAACCGAATCTCCGTGAAATTTGCCATGCCGTGGCAGGCCTTGATCGTTACGTGCGGCAGCCAGATCTGTGGCGGCTCACTCCTGAATAACTCCTGGGTGCTGACCACTGCCGACTGTGTCAGAAATATGAAGCCAGAGAACACGGCTGTGTTTCTGGGCCTGGACCAGCACGGCTCCTCCTTGCGAGTCATCCGAGTTGCCAACATCTTCCTGCACGACGATTATTTCTCCAACAGCCCCAGCAACAACCTGGCCCTGGTCCTCCTTCGGGGCCCCATCACGGGCGGCCAGTCCTTCGCACCCATAAGGCGCCGGTGGACCAGGAACGATGGGGATGAGTGCTGGTTCTCGGGACCCCGGGTCCTCAAACCAGGAGAGGCCGAAGGGTTCCCCAAGATGTTCCAGGTGCGGTTACTGAATGACTCTTCCTGCTCGGAATTCTACCAGGAACCCAATTCTGTCCTCTGCGTTGTTCCTAAACGGTCAAACCTCCCTGCGGCAAAGGTGACCCTTGTTTCGTGTATTTCAGGTGAGTTCAGGTGGTGCCTTGCTGTGCAGGCTGGGAGCAGCTAATGGTAGTTGGGCCCAGACCGGCATCGTGGGACCGTCCTACTTCTCCACCAACATCATTCCATTCTTGCGCTGGATGGAACTGACCGCCGCTCAGGCCGGCCGGCCCATTACCTTCTCTAAAGCCGCTTCCTCCACAGTGTTTAGCTCCTGGGTCCCTCGGGCCCAGCTCATTCTGCCCTTGCTGCTAGGGACAGTCATGGGGCTTGGTTACTTTTCCCAGTTACTTTTCCCACCCTGACGCCTACTGTCTCACCCAGCTAGAGCCTCTGAGGAGGCGGCAAACTGGCAAGACCAGCGCACACTGGGGAGCTGGTAGTGTTGAACTGGTCGGGGGGAGGGCCCGTGTCACCCGGAGAGCCTGGGACAGCAGTGCGAAGGATGGGGGAGAAAAAAACCTTTTCTACTTTCCTGGTTTGACC

>Elephant, Loxodonta africana

AGTGTGGATTACGTCCCGGCCTTGTCCCTCACTGTCCCAACTGCTGGGAGGCAGAAGTGGGTGAATTCCCATGGATGGTTTCTGTGCAACTGTCTTCCTCCCATTTCTGTGCTGGCTCTATATTGAATGAACAGTGGATCCTTACCACACCTAGATGTGCCAATTNNNNNAGTCTGGAGCAAAAGAATAACATACAACTATATGACTGCTGGTTACCCAGCTGGTCCCTCATGAGGGCTTGTGCCCAATTTTGGCCCAAACTGAATGAATTCACCTTCTGCGTGGAAGCCAAGAAAGCTATGGGGGAGGCTGGCTGTAAGGGTGACCTTGGGGCCCCCCTGGTGTGCCATCTACAACAAAAGGACACATGGGTGCAGGTGGGAATCTTGAGTCANNNNNNNNNNNNNNNNNNNNNNNNNNNNNNNNNNNNNNNNNNNNNNNNNNNNNNNNNNNNNNNNNNNNNNNNNNNNNNNNNNNNNNNNNNNNNNNNNNNNNNNNNNNNNNNNNNNNNNNNNNNNNNTTTTACCGCCACTCCTGCTTCTCTCCGGCCACACTTCATCTCCCTGCCACAGCCTCAGACTTTGGCAGGTAGGATTTCTCTTCGTTATGCCATGCCTTGGCAGGCCATGATCATCAGCTGTGGCAGTCAAGTTTGTAGTGGTTCCATTGTTAGTAACTCTTGGGTTCTTACTGCTGCACACTGTGTCAGGAACATGAATCCTAAAGATACTGCTGTGATACTGGGCCTGAGGCACCCTGGGGCACCTCTAAGGGTTGTTAAGGTGTCTACCATTCTGCTACATGAAAGATTTCGGTTGGTAAGTGGAGCAGCAAGAAATGACCTAGCATTGGTACTCCTTCAAGAGGTCCAGACTCCCGTTCAACTCCTAGCACCCTTGGGTCATCTGAAGAACCTGANNNNNNNNNNNNNNNNNNNNNNNNNNNNNNNNNNNNNNNNNNNNNNNNNNNNAGATGAGAACCCAGAAATGTTACAGATGCAAGTGAAGGGAGCTTCAAGCTGTGCCCACCTCTACCCAGACATAGGCACTTCTATTGTGTGTTTCATTACTCGGCCACAGGCTCTGATAAAAATGTGNNNNNNNNNNNNNNNNNNNAGTGCTGTTATGTGCAGACCAATATCTGGCAATGGCAACTGGAGACAAATAGGCCTTACCAGTCTCAAGGCACTGGCTACTATTGTGAGCCCACATTTCTCCTGGATCTTATCCGCTTCAGCAAAGGCAGGCCATCCCCTAAATCAGGCCCGCATGCCTTGGGTGGAAAAGCCCAAGTCCTCTAGCCTCCTTA

>Ferret, Mustela putorius furo (BK059503)

ATACCTGCAAATCATCCTAACCAGAAAGTCTCAACCCTGGCAGCTACTCAAGTAGAACACTGTTTTGCATTCACATCCACTTGAGCCAACATGAGATCTCCACTTTGCCTCTACCAGTTTTGGGGACTGTTTTTTTCACTGCTGTGGTGTACCCAATTCTCAAAAGCAGTGGTTAAGCTCATTGACCCCAACGTTTGCCCCATTGACAGCATCCAGAAACAACCCATGGCTAGTGTCCATGGCTGAAACCTGCCAGGGTGTTATTCTGAATCGGTGGTGGGTCCTCTCCACAGCCAGCTGTCTGAGTAAACTGAAACATTTGCACTCTGACATTTCAGGGATCACTGACCAAGAAGATTTCTTAATTGGCAAAAAAAATATGCCTGCACCCCAGTTTTGATCCACAAGTTGGAATGAATCCAGTCAAAGGAGTTATAGGAGTGGTCCTTCTGCAGTACCCTATTAGAGGGAAAAAAATACCACTTTATCAGACTCACAACATCTTCTGGAAGAGCTGTAATAACTGCCAGTACAGACACTGCAGGGTGTACCAATATCAGAAGCACAATAACTTTGGAACCAGTATCAAAACGCTGTCAGTTAAACTGCTGGACCTCTCACTTTGCCACCATCAACACATCCACCTGACCAAAAGTAACAATTTATGCATCTGGAGCCAGCCACAAGAAGACTGCTGGGTACAGCAGGGTAGTCCTGTTCTCTGCCTTTTTGGCAAACACTGGAAACTGGTAGGCTTGGTCAGTGAATCCTCAATGGCCTGTTATGACCCTGTTCTTGTCATCAAGACAGCTCCATATTTATCTTGGATGAACTGGCTTATCAAGACATCCCAGAAGACACTGGATCCTATTTTTTCTCTACTCTGCAGTTTTACTCCAGGGTTAGAACATGGTCCACTAGACAGGCTCAGCCTGAACAGGGAAACTGCCATTTTGACCTCCCCTGGATTCTCTCTACAGTCATGGAAGAGAACATTAGGCACTTTCCCACTGAACAGACAGCGCCGGAATCCTCCTCCCATATTTTTTCATTCAAATAATCAAGACTATTTTCCAGGCAGTAGAGAGTTACATCTTCAAACTAATCAGCTCTCCTCACCTAGCCACTTCCCAATGATACAATCTTGGACATCTCTTGTTACTAAACAGTGGGATCCTTCTGATATGTCGGAACCCTGGAATACCCCAACAGTTGATACTTCTGAAATTTTGGTTATTTCTGGAACACCAAAGCCTCCAACACCTGATAAATCTATACCCTGGGGCCTTCTTCAGAAAAATACAATGAACTATCAAAACCAAACTATTACCAATTCAGTAAATTCTTGGGTTAATCCTTTAGCTGGTAAAATTGGGCTTCATACACTGCCATTAGTTAATTCTGCTATATCCAGTGTTTTGTTTTCAAATGGCATAGATGGATCCCAACTTCTTTCTGGGGTTAATACCGTACAGTCTCAAGTTCAATCTAGTAGGTTTCCTTTTCATGGTCAACCTCTAGCTAGACCCTGGCTGGAAATTACCCCTGACATTGCACCTTGGAAACATATTGTACCTGATAAAACAGAAACAGTGATGCAAATCCAATCTACTGAAGAGAATGTTGGAAGCCAAATACACCATGTAGTTGATAGAGTTCACACTGCTTTTAAACCAGTAACTTACAACTTGAATGGATGGGTTCCTTTAACAGCTAATAAAAATGAATTCTGGACCCATTCCACACTAAATGCAGATGGATCTCTGTATCCTACAGTAACTCTTACCTTGGAACCTTGGTTCAAGTCAGTCTTGAATTTAGGTGGATCCCAAGAACTTATAGAAAAGACCAATGAATACTGGAGTCTTCCTGAATCTAAGTCAGCTCAATTATGGACTTCCTCAGCACTTAATATTCCTTTTACCTGGATTCCATCTTCAAGCAATAATATTAAATCTTGGGAAAAATATAAGACCAGTATTATCAAAGCATCAAGTCAAATGGATAGAATAAGTCCATTGATTGAACATGATGAGTCTATTGTTTTCAAACCCCAGATTCAAACTGCAGATACAACATGGTTTTTGATACCTACTATTACAAATGTAATTAAGCCTTTTGTTCAGCCTAAAGCTGTTACAATTAGACCCTTGACTAAATCTGAAACTGACATAGTCCAAACCTGGACCCAGCCAGAAACCCAAACAAGAAAACAACTGACTCAGCTGAAAGCAGATAAAATCAGACCATGGTTACAGACTAAAGCTGAAAAAATCAGATCCTGGATTCAGCCTAAGTTTCAAATATTCAGAGCTAGCGACCAGAATGAAAATGGTAAAGACAAATATTGGACCCCACCAGAAGCAGATATAATTAGATCCATGACCCAAACTGAAATTAAAACAGTCAGACTTGAGAACAAGCCTAAAGCTGGTATGGCCAGATCTTGGTTATGGACTAGGTCTAATCAAATGAGAACAAGTTCCCAGCCAGACTTTCAAACACTTTACCCTTGGACTCAGCCTGAAGTTGACATAATAGGACCATGGACTCAGTCTGAAGCTGGTAGCATCCAACCATGGATGAAACCTGAAGCATCAACATTGAAAATCTGGACACAATCTAAAGTTAATACAATTACACCCTGGACAAAGCCTGAAGATGATGCAGCTAGACTTTGGTTGCAAATACAAACAGATACAGTCAGAACATGGAGCCAACCTGAATCTCAAACAACCATTTCCTGGTCTGAGCTTGAAGCTGATAGAGTCAGATCTTGGTTTCAAACTCTAATGCATACATTAAAACCTTGGATTGAGACAGAATTTCAGACAACTCACTTCTGGACCCAACCTGAAGGTGACATAGCCGGGTTTTGGACTAAATCTGAGGCTAACAATGTCAGACATTGGTTCCAGACTCAAGTGGACACCGCCACAATATGGACAGAGTCAGTATCCCAAGCATCCCAGCCATGGATACAGTCCAAAACAGAAATAGTCCGGCCATGGAACCAGCCTGTGGCAGACAAGTTAAGAGCCTGGATACATCAGGACATTTATACTGTAAGGCCCTGGGATAAGCTTGAAGGTGATAAAATTAGATTCTGGACACAGTCTGAATCTGACACAAATCCTTGGATTCAGCCATATGTAGGTATAATCAATTCTGGGACACAAAATGAAGGGGATACATCGACACCATGGGCATGGGTGCAGGCTGAGACTCCAGAAGTTAATCCTTGGATACGGACTGAAACTGAAACAGTTATAGTCTCAACCCAAGGAGAAGCTCCAGCAATTAATCATAGGACAGAGGTTTTACCTGATACTACATGGGCAAAGACTGAATTTCCAGGACAAAAGCTCTTGACATATCCTTTGTCTCATACAGCTACACAGTTGACTCAGCCTGAGCTGCCAGTGGCCAGTCTCTGGACACAACCTATAGATGATACAGTCACTCAGTGGAACCATAATGAACCTTCAGAAATAAATTCTTCAACAAAGACTATAGTTGATACAGCCATGAGGACACAGGCTGAATCTCCAGCAGTGAATCCCAGGATACAGTCTGAAAATGATATAGTCACATCATGGACCCCAGATGAATCCCTAGTCATAAATCCTTGGACAAACTCTGTAGGTGATACAGTCACACAGTGGGTCCAGAGTGAACCTCAAGAAGTACTTCCTTGGGTAAAAATTGCAGCTGATACACCATGGATGCAGGTGGAGTCCCCAGCACTAAACCCCTGGATGCAGTCTGAAAGTGATATAGTCACACCATGGGTACAGGTTGAATCCTCAGCACTAAACCCCTGGATGCAGTCTGAAAGTGATATAGTCAAGCCATGGACACAGGTTGAGTCCCCAGCAGTAAATCCTTGGGTACTGTCTGAAACTAACACTGTCCTACTATGGACTCAGGCTGAATCTCCAGCAGTCAATCCCTGGATAGATTCTATAGCTGGGACAGGCACTCAGTGGACCCAAACTGAATCTTTAGCAGTAAATTCTTGGACACAGCCTGTAGCTGATACAGTCGCAGTGTGGACTCAGGATGAGCCTCCATTATTACATTCCTGGAAAAAGTCTAAAACCCATACACTTATAACATGGACTCTGACAGAATCTTTAGCAGGAAATTTCTGGAGACCATATGAAACTGATAGTACCACATTGTGGACCAACAAGGGAAATCTAGAAATAAATCCTTGGGCACAATCTGAAACTGATGCAGTCACAGCTTGGACCCAGGCAGAAACTCCTGTAATAAATTCGTGGCCACAACCTATAGCTGAAATAATTTCATCATGGACTCTGGCTGATTCTCCAGCAGTAAATCCCTGGGCAGAGACTGTGTCTGATAGTGTTATACTGTCGACCCAGGTTGAATCTCCAGTAGTAAATCCATGGTCACAATCTATAGCTGATGTGGACACACTGCAGACCCTGACTGATTCCATAGCATTAAATCCCTGGACAGAGCCTGTAACCAATACTGTTATACAGTGGACCCAGAGTGAACCTCCAGCAGTAATTCAGTGGACAAAAACTGTATCCAGTACAGTCACATCATTTACTGGGGATGAATTTCCTGCATTAGAGACCTGGATAGACCCTTTGGCTGATATCACATTGTGGACCCAGACTGAAACTCTAGTAGTAAATCACTGGACAGAGACTATAGCTTCCACTCTCACATCATGGACCCAGACAAAATCACCAACAATAAATTCACTGACAGGGGTTGTAGCTGCCACAGTAATACCATGGAACCAGGCTGAATCTGCAGGAGTAAATCCCTGGATGGATGCTGTAGCTTTCACAGTATCACTGTTGACTCATGATCAGTCTCCATCAGTGAAAACCTGGACAGAGGCTGTAGCTTTCACAATCACACCACTAACTTATGCTGAATCTCTGGCAATAAAGTCTATGACAAAGGGAGTATCTGATGCAATCATACTGTGGAATCAAGCTGAATCACCTCTAGTATATCCTTGGACACAGTCTGAAACTGATGCAATCATACGGTGGACTCAGGGTGAATCTTTAAAAATAAATCCTTGGACAGAACCTGACACGATCACACCATGGACTCATGGTGAGTCCCTAGCAGTAAATCCTTGGACAGATGCTTCAGGTGACACCGTAACACCAATGAACCTAGTTGAATATCCTTCAGTACATCCCGGGACAACATTTGAAATGGGTAATGTCACATTCGGGACCCAGGCAAATTCTGAAATCATAAATCTCTTGACACAGACCATAGGGGTTGATATAGTCACACTGTGGACCCAGACTGAATCTCCTGCAGTGAATCCCTGGACACAGTCTGAAACTAACATAGTCACACCATGGACCCAGGCTGAATTTGCTACAGTAAATCTCTGGACCCAAGCCAAAACTAATCTAGTCACACTGAAGACCCATGGTAGATACCCAGCATTAAATCCCTGGGCAGATCCAGAAACTAACACATTTAGATCATGGTCTCAGGTTGAGTCTTCAAGAGTAAATCCATGGACACAGTCTGAAACTGACACAGTCACACCATGGACCCCGCCTGAATCTGCTACTATAAATCCCTGGACACAACCTGAAAATGTTATAGTCACACTGTTGATCCAGGCTGAATCTCTTGCAATAAATCCCTGGACACAGCCTGAAAATAACTTAGTCACACCATGGACCCAGAATGAATTGCCAGAAGAAAACACCTGGACAGAGGCTTTTTCTGAAACATTCTTACCATGGACTATGGGATTTTTTCCAACCATAAATCCCTGGATAGATACTGTATCTGTTAAAGTCATACCAGGAACCAAATATCAATTTTCAGAAGTAAAACTTTGGACACTGGAGTTGTCCAGTACTTTGGACACTGAGATAGGTACAGTGAAAATGTGGACTCAGTCAGAATCTCCACCCTTAATTCCCTGGACAGAAGCTATAGCTTCCATAGTCCCATTGTGGACGCAGACTGAACATCTAGCTGTAAATCAAAATCCTAGAGCTGATAGAGTCACAAAATGGACACAGACTAAATCTCCATCAGTAAATACTTGGACTCAGGTTCAGTTTCCAGCCATAAATCCATGGTCACAGTCTGAATCACCAGAAGTAAATATCTTGACAGAGTACAAATCTCCAGCCCTAAACCCATCAGTTGAGGGGAGTATAGGTACATTGTGGACTCAGACTGAATCTCCATTAGTAAATCCTTGGATAGAGCCTGTGGCGTCCATAGTCATTCCATGGACACAAGCTGAATATCCAGCAGTAAGTTCATTTACACAGACTGTAGCTGATACAATTATATTGTGGGCTCAAGCTGAATCTCTAGAATTAAATCCCTGGACAAAATCTATAGCAGATACAGTCACAGTGTGGGTCCTGGATGAATCTTCAAGAACAAAACCTTGGACACAGATGTTAGCTTCACCAGACAAAATTTGGACCCAAGCCAAAATAGTAAATCCATGGTCTACTTTTGGAATATTTCCACAATTCACCCAAATTCAATCTGTACTGGTTAAAACTGGAACACAATTTGAGAGTGACACAGTCATAGCATTAACTCAGGGTCAGGCTCTTGCTCTGAATCCCTGGACAGAGATTGTGACTTCCAGAGACATTCTATGGACACAAGCTGTACATCCAGCAGTAAATCCCTGGACAGAGTCTAGTGCATCCAGATTCATTCCATGGACGCAGGCTGTGCCTCCAGCAGTAAATCCCTGGACTGTCACTTCTACCCAAGCTGAACCTCCAGAAGTAAATCCCTGGACAGAGGTTGTCACTTTTAGAGTCACTCCATGGACCCAAGCTATACCTATAGCAATAAATATCTGGACAGAAGCTTTCACTTCTAGAATCATTCCATGGACGCAAGCTGTACCTCCAGCAGTAAATCCCTGGAGAGAAGCTATCACTTCCAGAGTCACTCCATGGAATGAAATTGTACCTCCATCAATCAATCCCTGGACAGAGGCCATCACTTCCAGAATCACTCCATGGACCCAAGTTGTACCTCCAGCAGTAAATCCCTGGACAGAGGCCATCACTTCCAGAATCACTCCATGGACCCAAGTTGTACCTCCAGCAGTAAGTCCCTGGACAGAAGCCATCACTTCCAGTGTCACTCCATGGACCCAAACTCTACCTCCAGCAGTCAATCCCTGGACAGAGGCCATCACTTCCAGAGTCACTCCATGGACCCAAGTTGTACCTCCAGCAGTAAATCTCTGGACAGAGGCCATCACTTCCAGAATCACTTCATGGACCCAAGCTGTACATCCAGCAAAATATACCTGGACAGAGGCTGGGGCATCCAGATTCATTCCATGGACCCAAGCTGTATCCCGAGCATTAAATCCCTGGATAGAGGCTATCACTTCCAGAGTTTCATCATGGACCCAAGCTGTAACTCCGGCAGTAAATCCCTGGACAGAGGCTGGTACATCTAGAGTCACATCATGGAACCAAGGTATGTTTCCAGTAATAAATCCGTGGACAGTGACGCTTGTTTCTACAGTCACACCATGGACCCAGGTTTCCTATCCACTAAATCCTTTGACAGAGACTAAAGCTTCTACAGTGAGCATATGGGCTCAGAATGAATATTCATTAATAAAAACCTGGACACATTCTGCTGTTTCCACGGTTACATCCTGGACTTGGGCTGAATATCAAGCAGTAAATTCTTATACACCAAGTATAGCTGATATGGTCATATTTTGGACAAGGCTTATATCTGAGTCTAAGGAATCCTGGATACTGCCTGAAGCTAGTATATTCAGTATTTCATTGCATCCTCAAAATGATACTACTCAATCCTTGATTCAAAGTGAAAATCAAACATCTTTTCTATCAACACATCCTGGAATTAATAATATCAATATATGGACTTTACTTGAATTTGAAACACAGGTATCATGGATAGTGCCTTTGTCTCAAGAAGCCAGACTCTCGCCCCTATCTGAAACTGATATTAGCATATATTGGTTTAAAACTGAAACAGAGAGAGTAAGAACCTGGGACCACTCAGAATTTCAAACAGTGAGTACTTTGACACAGTTTGAAATTGGGGGATTTGAGCCCTTGGCCAAACATGAAATTCCTGCAGTCATATCATGGGTTCCAACTAAAATTGGTGTATTCCCCCTCTGGAATAAGTCTGAAAGAGACAAAATAAGAACCTGGACCCTTTCTGGAGATGATGTCTTGCCATCATGGAGTCAGGTTGAAGCTAGTATATTCAGCCTCTGGAGCCAGTCTAAAAGTAATACAATCACACCCTGGATCCAGGCTGAGACTCAGTCAGTCAGTATCTGGACTGAAGGAAATTCAGGCACATTTTGGCACCTGAGTCAAAATAATGTAGTTAAGCCCTGGTCCCACCTTGAATCTGAAATGACATTTTCCAGGATTCAAAATGGTATAAATAGTTCTTGGACTCAGTATGAAACTAGTACTGTCAGATCTTGGACCAAGCTTGAAATTAGTACAGTGCAACCTTGGAATCAATTTGAAACTGTTACAATTAGATCATGGACCCAGTCTGAAAATGTAGAAATATACCCCCTTACCCAGAAAGAAGCTGGTACAGTAATAAGACATTGGTTCCAGACTCAAATGGATTCAATAAAACCTCGGAACCAGTCTGAAACTAATACAATTAGATCATGGACTCAGACTGGAACTGAAACAATCCAAATTTGGACCCAGACAGAAAGGCAAATAGTAAAACCTCCAACTTTATCTGAGATTGATACAATTACATCTTGGTTACAGACTCAGAGTGATACAAATAGACCCTGGATTAAATCTGACTCCTTGTCTGCCAACCCCTGGAGTCAGGCTGAAGTTGGTACAAATCACCCCTGGACTCAGCAGAGAGGTACTGTGAATCAACCCTGGACCTACTCCGAAATCCAGAAAGTCAGACCTTGGATGAAGCTAGAAGCTGATGCACTTAAATCTTGGTTCTACATGCAAATGAATAAAATCAGACCATGGACCAATTCCGAATCTCAGATCTTCAGCTCCAGGTTGCAGCCTGAAGTTGGTATGGTTCACCCTTGGATCCATCCTGAAACTCAAGCAGTCAGATCCTGGGCCCAACCTGAAACTGCCACACTTTTAACAAAAGAACCTCCTCTGATGACTGGATCTCAATCTGGAACCAAGTCAAAACAGCCTGAAGAAGATCCTCTCAAGTATTCGGAATTCAATGTTTCTTTGGCTGAATGTCGCCTAAGTGTGGTTTGGAAAGAGAGTACCCAGGCTTTCTGGCTCTTCAAGACAGCTGTTATTTCTCATGAAGCCACAGAATGTGGGTTACGACCTGGCCTTGTTCCCCACTGCCCCAACTGCTGGGAAGCTGAATTGGGTGAATTTCCTTGGATGGTTTCTGTGCAACTCTCTTTCTCCCATTTCTGTGCTGGTTCTATACTGAATGAACAGTGGATCCTTACTACAGCTAGATGTGCAAATTTCATAAAAAACTCAGAAGCACTGGCCCTGGTCCAAGTGGGACTTATTGATCTTCAGGAGCTTGCTCAAGCTCAAACTGTAGGCATTCATCGTGCCATGCCCTACCTAGGTCCCAAGGGACCTTTGGGTCCTGGGCTAATCTTCCTGAAACAGCCACTACATTTCCAACCACTGGTGCTTCCTATTTGCCTAGAGGAGAACCTGGAGCAAGAGAAAAATATACAGCTGTATGACTGCTGGCTACCCAGTTGGTCCCTCATGAGAGGAAGTCCTGGAATTCTGCAAAAAAGACACCTAAGCATTCTGCAAGTCAGCACATGTGCCCAGTTTTGGCCCAAGCTGAATGAATTCACTTTCTGTGTGGAAGCCAAGAAAGCTATGGGCGAGGCTGGCTGTAAGGGTGACTTAGGGGCACCTCTGGTGTGCCATCTACAACAAAAGGACACATGGGTGCAGGTGGGAATTTTGAGTCACTTCGATGAACATTGCACAAAGCCCTACGTCTTCAGCCAAGTGAGCCCGTTCCTTTTTTGGCTTCAAGGAGTTACACGACCCAGCCATGCACCATGGTCACAGCAAGGGGCCATGACTACTTCTGCTTCCATCTCCCTTTCAGTCTCTACTTCAATGAATGCCTCGGCTTTTACTGCCACTCCTGCTTCTATTAAGCCTCAGTTCATCTCTCTGCCACAGCCTCAGACTTTAGCAGACCGAATTTCTCTACGATATACCATGCCTTGGCAAGCCATGATCATCAGCTGTGGCAATCAAATTTGCAGCGGTTCCATTGTTAGCAGCTCTTGGGTTCTCACTGCTGCCCATTGTGTCAGAAACATGAATCCTGAAGATACTGCTGTAATATTGGGCCTGAAGCACCCTGGAGCATCTCTGAGAGTTGTTAAGGTGTCTAACATTCTACTGCATGAGAGATTTCGGTTGGTGAGTGGGGCAGCAAGAAATGATCTAGCACTGCTGCTCCTTCAAGAGGTCCAGACTCCCATTCAGCTCTTAGCACCCCTGGGACATCTAAAGAACTTCAATAGCTCAGAATGCTGGCTTTCTGGGCCAAGAGTTCTCAAACCAGGAGAGACTGATGAGGATCCAGAAATGTTACAGATGCAGGTGATGGGAGCTTCAAGTTGTGCCCACCTCTACCCTGACATTGGCAGTTCTATTATCTGCTTCATTACTCAGGCGAAAGGCTTAGATACAAATGTGGAACCTGTGACTCCTGGAAGTGCTGTTATGTGCAGACCAGTGTCTGGAAATGGCAGCTGGAGACAGATAGGCCTTACCAGTCTAAAGGCACTTGCTACCATTGTCAGCCCACACTTTTCCTGGATATTATCCACATCAGCAAAAGCAGGTCATCCCCTAAACCAGGCAGTCATGCCTTGGGTGGAAAAGCCAAAGTCATCCAGTCTTCATAAACAGGCAACAATACTACCACTTTCATCATTAATGATTCTTGCAATGCAGAGTCTGTTGTAAACCAGTGGTTGTCCTGAACACTACTACTCCAGTCACATTGTGATTTAAAAAAGAGATTAGAACATAAATTAAAACAAACCAGTTGAATTAAAACCCTAGATTCTCCACAGTTATTCCTTATAAATGCATCTATCCTATTGAAATGAGAGTCTACCGAGTTTAACAATTTGAAATTTCAAAAATTTAGAACTGTCCAAAATGACATTTGTGTAATAGTGAATAAGATAATAAAAAAATAAATTTCATCAACAAACACACACATATTATATTCTCAC

>Flying Fox, Pteropus alecto

ATGACTACTTCTGCTTCCATCTCCCTTTCAGTCTCTACCTCTACGAATGCGTCAGGTGTTACCTCCACTACTGCTTCTATTCGGCCACACTTCATCTCTCTGCCACAGCCTCAGACTTTGGCAGATCGGATTTCTCTGCGGTATGCCATGCCTTGGCAGGCCATGATCATTAGCTGTGGCAGTCAAATTTGTAGTGGTTCTATTGTTAGCAACTCTTGGGTTCTCACTGCTGCCCACTGTGTCAGGAATATGAATCCTGAAGACACTGCTGTAATACTGGGCCTGAGGCACCCTGGGGCACCTCTGAGAGTTGTTAAGGTATCTACTATTCTACTGCATGAAAGATTCCGGTTGGTGAGTGGGGCAGCAAGAAATGATCTAGCATTGCTGCTCCTTCAAGAAGTCCAGACTCCCATTCAGCTCTTGGCACCTTTGGGCCATATGAAAAACCTGAACAGCTCCGAATGCTGGCTTTCTGGGCCACGAATTCTTAAACCAGGGCAGACAGATGAGAACCCAGAAATGTTACAGATGCAGGTGACAGGAGCTTCAAGCTGTGCATACCTTTACCCTGATATAGGCAGTTCTTCTGTCTGCTTTATGACTGAGGCCAAAGGCTCTGTCACAAATGTGGAGCCAGTGAGTCCAGGCAGTGCTGTTATGTGCAGACCAATATCTGGCAATGGCAGCTGGAGACAAATAGGCCTCACAAGTCTGAAGGACCTAGCTACCATTGTGAGGCCATACTTCTCCTGGATATTAACCACCTCTGCAAAAGCTGGTCATCCCATAAACCAGGCCCTCATGCCTTGGATGGAAAAGCCCAAGTCAGCTGGACTTCTAAAACAGCCAAACACACTGTCATTTTCTTGTGTAATGATTATTGTACTTCAGAGTCTTTTGTAA

>Fox, Vulpes vulpes (BK059505)

AGCTATTCAACTAGAACCCTATCTTGCATTCAAATCCACTTGAGCCATGATGAGATCTCCATTTTGCTTCTACCAGTTTTGGGGGTTGTTTTTTCACTGCTGTGGTGTTCCCAATTCTCAAAAGCAGTGGTTAAGCTCATTGACCTCAACGATTGCCCCATTGACAGCGTCCAGCAACATCCCATGGTTAGTGTCTATGGCTGAAACCTGCCAGGGCATTATTCTGAGTCAGTGGTGGATCCTCTCCACAGTCAGCTGTCTGAGTAAACTGAAGCATTTGCACTCTGACATTTCAGGAGTCCTTGCCCAAGAAGATATCTTACTTGGCCATAAAATATGCCTGCACCCCAGTTTTGATCCACAAGTTAGAACAGATCCAGTCAAAGGATACATAGGGGTGATGCTCCTGCAGTACCCTATCAGGCGGGAAGAAATATCTCTTTATTCCACTTATAACATTTTCTGGAAGAGCTGTTATAACTGCCAATTCAGACACTGCAGAGTATACCAAGATCAGAACCACAATAACTTCGGAACCAATATCAAGAAGCTGTCAGTTAAGCTGCTGGACCTCTCATTCTGCCACCATCAACATATTCAGCTGACTAAAAGTAACAATTTGTGCATCTGGAGTCAGCCACAAGAAGACTGCTGGGTACAGCAGGGTAGTCCTGTTCTCTGCCTTTTTGGCAACCACTGGGAATTAGTAGGCCTGGTCAGTGAGTCCTCAATGGCCTGTTATGACCCTATTTTTGTCATCAAGACAGCCCCATATGTATCTTGGATGAGATGGCTTATCAAGGCAACCCAGAAGCCATTGGATCCTATTTTTCCCCCACCCTGCAGTTTTACTCCTGGGGTAGAACATGTTCCACAAGACAGGCTTAGCCTGAAAAGGGGCACTGCCATTTTGACTTCCCATGGATTTTCTATACAGTCATGGAAGAGAAGATTAGGTACTTTCCCACTAAATAGACAGCGCCGGAATCCTCCTCCAGTATTTTCGAATTCAAATAATAGAGAGGCTTTTCCTGGCAGTACACAGTTACACCTGCAAACTAGTCAGCTCTCCTCAACAAGACTGATACTATCTTGGACCTCTCCTCTTATCAAACAACAGGATCCTTCTTATATATCTGAGCCCTGGAATACCCCAAGAGCTGGTACTTCTGAAACTTGGGTACTTTCTGGACCCCCAAAGTCTCCAGCATCTGATAAATTTATACCCTGGGACCTTCCTCAGAAAGATACAATGAAATATCAATACCAAACTATAACCAATTCAGTAAACTGGGTTAATCCTTTAGCTGTTATAATTGGGATTCACACTCTAACATTAGTTAATTCTGCTATACCCTGGGTTTTGTTTCCAAGTGGCATACATGGATCCCAACTTCTTTCTGGGATTAATATTGTACAGTCTCAAGTTCAGTCTAGTAAGTTTCCTTTCCATGACCAACTTCTATCTAGAACCAGACTAGATATTACCTCTGACATCGCACCTGGGATACACTCTGCACCTGATAAAACAGAAACACTGAAGCAGATGCAGTCTAGTGAAGAGAATATTGGAAGCCAAATACACCATGTAGTTAATATAATTCACACTACTTTTAAACCAGTAACTTTTAATTTGCATGCATGGGTCCCTTTAAGAGTTAGTAAAAATGAATTCTGGACGCATTCCACACTAAATGCTGATGTATCTCAGTATCCTACAGTAACTCTTACCTTTGAACCATGGTTTCACTCAGTCTTAAATATGGATGGATTCCAAGAACTTACAGAAAAGACTAATAAATACTGGATTCTCCCTGACTCTAAGTCAGCTCAATTGTGGACTTCTTTAGCACTTAATATGCCCTTTACTTGGGTTCCATCTACAAGTAACACTATTAAGTCTTGGGCACAATATAAGAGCAGTCTGATCAAACCCTCGAGTCAAATAGAGAAAATAACTCCACTGAGTAAGCGTGAATCTATTATGGTTAAACCCCATATTCAAACTGCAGCTGCAACCTGGTTTTTGATACGTACTATTACAAATATAATTGAGCCTTTCATTGAGTCTAAAGCTGATACAGTCAGACCCTGGACTCAGCCTGGAGCTAACATAGTCCAAACCTGGACCCAGCCAGACACTCAAGCAAGAAAATCACTGACTCAGCTGAAAGTGAATATAAGTAGACCACGGATACAGACTAAAACTGAAAGAATCAGACCCTGGATTCAGCATAAGTTTCAAATACTCAAACCTCGAGCCCAGACTGAAAATAGTAAAGACAAACTTCAAACTCAGTCAGAAGCATATATAATTAGATCCATGATCCACACTGAAATTAAAACAGTCAGATTTTGGAACAAACCTAAAGTTGATACAGCCATATCATGGTTAAGGACTAGATCTAATCAAATAAAAGCAAGATCCCAGTCAGACTCTCAAACACGTTACCCTTGGACTCAGCCTGAAGCCGGCATAGTGAGACCATGGACTCAGTCTGAAGCTGACAGTATCCAGCAAGGGATGAAGCCTGAAGGATCAACATTCAGAATCTGGACACGGTCTAAAATTAATACAGTTACACCCTGGACAGGGCCGGAAGCTGATGCAGCTAGACTTTGGTTGCAAATACAAACCAATACAATCAGAACTTGGAGCCAACCTGAATTTCAAACAACCATTTCCTGGTCTGAGCTTGAAACTGATAGAGTCAGATCTATCAGTCAAATACATACATTCAAACCTTGGAGTGAGACAGAAATTCAAACATCCCACTCCTGGACTCAATCTGAAGGTGATATAGCCAGACTCTGGACTAAATCTGAGGCTGACAATGTCAGACATTGGTTCCAGACTCAAGTGGAGACAACCACAATGTGGACAGAGCCAGTAACCCAAACAATCCACCACTGGATACAGTCTAAAACAGAAATAGTCAGGCCCTGGAGCCAACCTGCGGCAGATAAGCTCAGAGCTTGGATACAACATGAAATTTATGCTGTCAGGCCCTGGGATGAGCTTGAAGGTAATAAAGTTAGATTCTGGACCCAGTCTGAATCTGACACAAGACCTTGGATTCAGCCAGATGTGGGTATAATAAATCCCGGGGCACAAAATGTTGCTGATACATCAACACCATGGGCATGGCCACAGGCTGAGACTCCAGAAGTTAATACATGGGCACAGTCTGAAACTGAAACAGTTATACTCTCGACCCAAGGAGAAGGTCCAGCAATAAATCATTGGACAGAGATGTTAGCTGATACTGTCACAACATGGGAAAAGGCTGAATTTTCAGGACAAAAGCTCTTGGAACATCCTTTGTCTCATACAGTCACACAGTGGACTCAGCCTAAACTGCTAGCTGCAAATCTCTGGATGCAGCCTGAAGATGATTTAATCACACAGTGGACCCAGGGCGAATCTTCAGAAATAAATTCCTCAACAAAAATTATAGCTGACACAGCCATGTGGACACAAGCTGAATCTCCAGCAATGAAGCCCTGGATACAGTCTGATAATGATACAGACATATTCTGGACCCAGGATGAGTCCCTAGAAATAAATCCTTGGACAAAGTCTGTGGCTGATACAGTCACACAATGGGCCCAGGGTGAACCTTCAGAAGTACTTCCTTGGGTGAAAATTGTAGATGATACACCATGGATGCAGCCTGAATTCCCAGAAGTAAACCCCTGGACACAGCCTGAAAATGATACAGTCATACTATGGATACAGGGTGACTCTCCAGAAGTAAACCCCTGGACACAGTCTGAAAGTGAAACAGTCATACCATGGATACAGGGTGAATCTCCAGAAGTAAACCCCTGGACACAGTCTGAAAGTGATATAGTCATACCATGGATACAAGGTGAATCTCCAGAAGTAATCCCCTGGAGACAGTCTGAGAGTGATACAGTCATACCACTGATGCAGGCTGAGTCTCCAGAAGTAAATCCCTGGATACAACCTGAAACTAACAGAGTCCTACTGTGGACTCAGGCTGAATCTCCAACAGTAAATCCTTGGATAGATGCTGTAGCTGGGACAGGCACACAGGGGACCCAAGCTAAATCTCTAGATGCAAATTTTTGGCCACAGCCTGTAGGTGATACACTCACACTGTGTACTCAGGATGTGTCTCCATCATTACATCTGTGGAAAAAGTCTGAAACTCATACACTTACAACATGGACTCTGACAGAATCTTTAGTAGAAAATTCCTGGAGACAGTATGAAATTTATGGTGTCTCAACATGGACCAAAAAAGGAAATCTAGAAGTAAACCCCTGTGTACAATTTGAGACTGACACAGTCACAATTTGGACCCAGGAAGAAACTCCAGGAGTAAATTTGTGGAAACAACCTACAGCTGATATAGTTCCACCATGGACTCTGACTGAATCTACAGAAGTAAATCCCTGGAGAGACACTGTATCCAATAGTGTTACACAGGGTGTGGAGGCTGACTCTCCAGCAATAAATCCCTGGACAGAGACTGTACCTGATAGTGTTATACAGTGGGCTGAGGCTGACTCTCCAGCAGTAAATCGCTGGACAGAGACTATACCTGATAGTGTTATACAGTGGGCTGAGGCTGACTCTCCAGCAGAAAATCCTTGGACAGACACTGTACTTGATAGTGTTATACAGTGGGCTGAGGCTGACACTCCAGCAGTAAATCCTTGGACAGAAACTGTACTTGATAGTCTTATACAGTGGGCTGAGGCTGACACTCCAGCAGTAAATCCCTGGACAGACACGGTATCTGATAGTGTTATACAGTGGGGTGAAGCTGACCCTCCAGCAATAAATCCTTGGACAGAGACTGTACCTGATAATGTTATACAATGGGCTGAGGCTGAATCTCCAGCAGTAAATCCTCAGACACAATCTATAGCTGATGTAGACATACTGTGGACCCTGGCTGATTCTACACAATTAAACTCCTGGACAGAGCCTGTATCTGATAGGGTAATACAGTGGACCCAGAGTGAACCTCCAACAGTACCTCAGTGGACAGAAGCTGTTTCAGATACAGTCACATCATTTACTAATGAAGAATTTCCTGAAGTAGAGACCTGGACAGACCCTTTTGATGATGTCACACTGTGGTCTCAGACTGAAACTCTAGTATTAAAACCCTGGACAGAGACAGTAGCTTCAACGGTTGCAACATTGACCCAGACAGAATCACCAGCAATAAATCCAATGACAGAAGCTGTATCTACCACAGTAATACCTTGGAATCAGGCTGAATCTACAACAATAAATCCCTGGATAGATGCTAGAGATTTACTTTTAACACTGTTGACACAAGGTCAGTCTTCACTAGTGAAAACCTGGACGGAGGCTTTAGCTTTCATAATCACACCACTGACTCAGGCTGAATCTCTAGCAATAAAGTCTGTGACTCAGGGTATATTTGATACAATCATACTGTGGAACCAAGCTGAATCACCTTTAGTACGTCCTTGGACACATTCTGAAACTGAAACACAATGGACTCAGGGTGAATCTCTAAAAGTAATTCCTTGGACACAATCTGTAGCTGTCACAGTCACACCATGGAATAGTGGTGAGTCCCCAGCAGTAAATCTCTGGACAGATGCTATAGGTAACACAGTAACAACAATGAATCTTGATGAAGAAATTGGTGCTGTCACACTCTGGACCCAGGCAAATTCTGATATCATAAATCTCTTGTCACAGACTATAGTTGATATCGTCACACTGTGGACCCAGGATGAATCTCTTGCAGTGAATCCCAGAACACATTTTGAAAGTGATACAGTCACAGCATTTACTCAGGATGAATCTGCTACAATAAATCCCTGGATCCAGCACAAAACTAATCTGGTCACATGGCAGACCCAGGGTAGATATCCAGCATTAAATCCATGGATACAGTCAGAAGCTGACACATTTACATCATGGTCTCAGGCTGAGTCTCCAGCAGTAAATCCATGGGCACAGACTGAAAGTGACACAATCACAACATGGACCCAGGCTGAATCTCCTGCAGCATATCTCTGGACACAGCCTGAAAATGTCACGGTCACACTGCGGATCCAGGCTGAATCTCTTGTAGTAAATCCCTGGACACAGCATGAAAATAACACAGGCACACCATGGACCCAGAATCAATCTTCAGAAGAAAACACCTGGGAAGAGGCTGTTTCTGAAACTGTCATACCATGGACAATGGGATTTTTTCCAAACATGAAACCATGGATAGAGATGATATCTGATATAGTTACACCTGGCACTAAATCTCAATTTTCTGAAGTAAAATTTTGGACAGTGCCAGGGTCCAAATTGGACACTGATGTAAGTACAGTAAAAAAGTGGACTCAGTCTGAATCTCCACCCTTAATTCCCTGGACAGAACCTATAGCTTCCATAGTACCACTATGGACTGAATCTCTAGCTGTAAATCACTGGACTCAGCCTATTCCTGATACAATAACAAAATGGACACAGACTGAATCTCCATCAGTAAATACCATTCAATTTCCAGCAGTAAATCCATGGACACAGTCTGAATTACCAGCAGTACATTTCTTGACAGAGTACAAATCTCCAGCCCTAAAACCATCAATTGAGCCTGAAGCTAGTATAGTTACATTGTTGACTCAGACTGAATCTCCAGCAGTAAATCCATGGATAGAGCCTGTTGCTTCCATAGTCATTCCATGGATACAAGCTGAACATCCAACAATAACCCCAATGACACAGGCTGTAGCTGAAACAATTATCCTGTGGACTCAGAATGAAAATCTAGCATTAAATCCCTGGACAAAGTCTGTTGCAGATACAGTCACATTTTTGACCCTGGATGAATATTCAAGAGCAAAACTTTGGACACCTATATTAGATTCCTCAAATATATTTTGGGCCCAAGCCAAAAATTCAGCAATAAATCCTTGGTCTGATTTTGAAATATCACCATTGACCCAATTACAATCTGTAGTGGTTAAACCATGGACACAATTTGAGAGTGACACAGTCACACCATTAATGCAGGTTCAGGATCTTGCTGTAAATCCCTGGTCAGAGATTATTGCTTCCAGAGTCACAACACAGACTCAGTCTGTATCTCCAGCAGTAAACCCCTGGATGGAAGTTGATGAAACCAGACTCCCATCATGGACCAAAACTGCAGCTCCGGCAGTAACAACCTGGACAGAGGCTGATGTATCCAGAGTACTGCCATGGACCCAGTCTCTACCTCCAGTAGTAAATCCATGGACAGAGGTTTATGCATCCAGAATCACACCATGGACCCAAGCAGTAGCTCCAACAGTAAATCCCCGGACAGAGACTGTCAGTTCCAGATTCATGCCATGGACCCAGGCTGTCCCTCCAGCAGTAAATCTGTGGACAGAAACTAATGCATCCAGAGTTATACCATGGACACAGGATATATCTCCATCAGTAAATCCCTGGACAGAGGCTGTTGGTTCCAGATTCATGGCATGGATTCCAACTGTACCTCTAGCAGTAAATCCCTGGACAGAGGCTGTTGGTTCCAGATTCATGCCATGGACCCAAGCTGTACCTCCAGCAGGAAATCCCTGGACAGAGGCTGTTGGTTCTAGATTCATGCAATGGACCCAAGATATACCTCCAGCAGTAAATTCCTGGGCAGAGGCTATTGGTTCCAGATTCATGACATGGACTCAACCTGTAATTCCAGCAGGAAATCCAGGGACAGAGGTTGTTGGTTCCAGATTCATGCCATGGACCCAAGCTGTACCTCCAGCAGGAAATCCCTGGACAGAGGCTGTTGGTTCTAGATTCATGCAATGGACCCAAGATATACCTCCAGCAGTAAATTCCTGGGCAGAGGCTATTGGTTCCAGATTCATGACATTGACTCAACCTGTAATTCCAGCAGTAAAACCAGGGACAGAGGTTGTTGGTTCCAGATTCATGACATGGATTCAATCTCTAATTACAGCAGTAAATCCCTGGACAGAGGCTGATACATCCAGATTCATGTCATGGACCCAAGCTGTACCTCCAGGAGTAAATCCCTGGGCAGAGGTAAATGCATCCAGAGTCCTGCCATGGACCCAAGCCGTCTTTCTGGAAGGAAATCCCTGGACAGAGGCTGATGCATTCAGAGTCCCAGCATGGATGCATGTAAATCCATGGACAGAGGCTGTCAGTTCAAGAGACATGCTATGGGACCAACCTGTACTTCCCGCAGTAAAACCACAGACAGAGGCTGTAACTTCCAGAGTCTCACCATGGACACAAGCTATAACTCCATCAGTAAATACCTGGACAGAGGCTATTGTATCCAGAGTCATAACAGGGAGTCAACCTATACTTCCAGCAGGAAATCCCTGGACAGAGGATGATGTATCCAGAGTCATGCCATGGACCAAAGCTTTACATCCAGCAGGAAATCACTGGACACAGGCTGTTGGTTCCAGATTCATGCCATTGACCCAATCTATACCTTTAGCATTAACTCCATGGACAGAGGCTGTCACTTTCAGAGTCTCACCATGGACCCAAATTGTACCTTTAGCAGTAAATCCCTGGACAGAGACCATAACTTCAACAGGCATGCCATGGACCCAACCTGTACCTCCAGCAGTAATTCCCTGGGCAGAAGCTGTACCTCCCAGAGTCCCGCCATGGACCAGAGCTGTATTTCCAGCAATAAATCCCTGGACAGAGGCTGTGGGTTCCAGATTCATATCATGGACTCAAACAGCATCTCCAATAGTAAATCCCTGGAAAGAGGCTGTTGGTTCCAGATTCATGCCATGGATCCAACCAGTAACTCCAGCAGTAACTCCCTGGACAGAGGCTGATGCATCCAGAGTCTTGCCACGGACCTTATCTGTACCTCTGGCTGTGAATCTCTTGACAAATGTTTCTGGTTCCAGATTCATGCCATGGACTCATCCTGGAACTCCAGCAGTAAATCTCTGGAAAGAGGCTTTCAGTTCCAGATTCATGCCATGGAACCAAGCTGTACCTCCAGTGGTAAATCCCTGGACAGAGTCTGTCACTTCCAGAATCACACCATGGACCAAACCTGTAGCTCCAGTAGTAAATCCTTGGTCAGAGGCTGTTGGTTCCAGATTCATGCCATGGACCCAAACTGTACCTCCAGCAGGAAATCCCTGGGTAGATGCTGGCACTTCCAGAGTCACTCCATGGACCCAAGCTGTACCAGCAACAGGATATCCTTGGACAGACACTGATGCATCCAGAATCACACCATTGAACCAAGCTGTAACTCTAGGACTAAATCCCTGGGCAGAGCCTATTGCGTCCAAAGTCATACCATGGACACAAGGTATATCTTCAGCAGTAAATCCGTGGGCAGAGGATATTCCTTCCAGAGTCATGCCATGGACCCAAGCTGTACTTCCAACAATAAATCCCTGGACAGAGGCTGATTCATCCAGAGGCATGCCATGGGCCCAATTGGTACCTCCAGCAGTAAATCCCTGGACAGAGGCTGATTCTTCCAGAGTCATATCATGGACCCAAGTTATACCTCCTGCAGGAAATCCCTGGACAGAGGCTGTTAGTTCCAGATTCATGCCATGGACTCAACCTATACCTCAAGCAGTAAATTCCTGGACAGAAGTTTATGCATCTAGGGTCCCACCATGGTCCCAACCTGTACCTCCAACAGTAAATCCTTGGACAGAGGGTGGTGCATCCAGAATCACTCCATGGAATCAAGGTGTAACTCTAGCAGTAAATCCCTGGACAGAGGCCATTGGTTCCAGATTCATGTCACTGACCCAACTTGTGCCTTCAGCAGTAAATGCCTGGACAGATACTGATATATCTAGATTCATGCCATGGAATCAACCTGTACCTACTTTAGTAAATCCCTGGATAGAAGCTGTTGGTTCCAGATTCGTGCCATGGACCCAACTGGTACTTCCAACAGTAAATCCCTGGTCAGAGGCTGATGTATCCAGATTCATGCCATGGATCCAACCTGTACATCCAACAGTAAAACCCTGGTCAGAGGCTGATGCATCCAGATTCATGCCATGGACCCAGCTTGTACCTCTAACAATAAATCCCTGGTCAGAGGCTGATGCATCCAGATTCACGCCAGGGACCCAACCTATACCTCCAGCAGTAAACCCCTGGACATATATGAATGTATCTAGAATCACTCCATGGTCCCTAGATGTGCCTTCATCAGTTAATCCCTGGACAGATGCTGTTGCTTCCAGATTCAATCCATGGACCCAAACTGTATCTCCAGCAGTTAATCCCTGGACAGAGGCTATTGGTTCCAGATTCATGCCATGGACCCAACTTGTACCTCTACCAGAAAATTACTGGTCAGAGACTGATGCATCCAGAGTCATGGCATGGACTCAACCTTTATTTCTAGCAGTAAATCTCTGGAGAGAGACTGATGCATCCAGATTCACACCAAGGACCCAACCTGTACCTCCAGCAGTAAATCCCTGGACAGATCCCGATAGATCTAGAGTTATGCAATGGACCCAACCTCTACTTCCTGTAGCAAATACATGGACAGAAGCTGATGCATCCAGAGTCATGCCATGGACCCAAACTTTACCTCTAGCAGTAAGTCCCTGGACAGAGACTATTGGTTCCAGATTCACACCATGGACCCAACCTTTACCTCCAGTAGTAAATCCTTGGACAGAGGCTGATGCATCTAGAGTCACACCATGGATTAAACCTTTACTTATAGCAGTAAATCCCTGGACAGATCCTGATGTATCCAGAGTCACACCATGGACTTATCCTTTACTTCTTGCAGTAAATCCTTGGAGAGAAACTGGTGCATCCAGATTCATGCCATGGACCCAACCTACACCTCTAACAATAAATCCCTGGTCAGAGGCTTATGCATCCAGATTCATGCAATGGACCCTACCTGCACCTCTAACAGTAAATCCCTGGTCAGAAATTGATGCATCCAGAGTTATGCCATGGACCCAACCTGTACCTCCAGCAGTAAATCCCTGGATAGTGACAGTTGTTTATACTTTAACACCATGGACCCTGGATGCCTCTCTACTAAATCCTTTGACAGAGACTAAGGCTTCCTCAGTGGGAATATGGACTCAGAACAAATATTCAGTAGTTAAAACTTGGACAAAATCTGAAGTTTCCACACTTACATCCTGGACAGATACTCAGACAGAATATCAAGCAGTAAATTCTTATATATCTTATATATCAAGTGTAACAGACACAGTCACATTTTGGACAATGCCAAAATATGAATCTAAGGAAACTTGGATACTGCCTGAAGCTCATATATTTAGTATATCATTGCATCCTCAAAGTGATACTCAATCCTTGTTCCAAGTAAAAAATATAGCATCTCTTCTGTCAATATATCCTGGAATTAATAGTGTCAATACATGGTCTTTGCCTAAATTTGAAACAGTGGCATCGTGGATAGTGCCTTTGTCTCAAGCAGCCAGATTTGTGCCCCTACCTAAAGCTGATATTAATAGAAATTGGTTTAAAATGGAAACAGAAACATTAAGAAATTGGACCCATTCAGAATCTCAAAGAGTAAGCACTTTGTCACAGTATGAAGCTAGTATGAAGCCCTTGGCCCAACATGAAAATTCTACAGTCATATCATGGATTCCAACTAAAACTGGTATATTCCAACCCTGGAATAAATCTGAAAGGGACAAAGTAAGAACCTGGACCCTTTCTGAAGGTAATGCTTTGCGACCATGGATTCAGATTGAAGCTAGCATTTTCAACCTCTGGACTCAGTCTAAAAGTAGTACAGTCACATCCTGGACACAGCCTGAGTTTCAGGCAGTCTTTACCAGGACTGAAGGACGTATAGATACATTTTGGTCCATGAATAAAAATGATGCAGTTAGGCCCTGGTCCCAGCTTGAATCTCAAATGACATCTTTCTGGACCCAAAGTAACATAACCAGCTCTTGGACTCAGTATAAAACTAGTACATTCATATCCAGGACCAAGCTTGAAATCAGTACACTGCAACCCTGGATTCAGTTTGAAACTGCTGCAATTAAATCATGGATTCAGTCTGAAAATGTAGAAATATACCCTTTGACCCAGCCAGAAGCTGATACAGTAATAAGACCTTGGTTGCAAATTCAAATAGATTCAATACAACCTTGGAATCAACCTGAAACTAATACAATTAGACCATGGACTCAACTTGAAACTGAAGTAATCCAAATTTGGACCCCCACAGAAAAGCAAGTAGTAAAACCTCCAACCTTACCTGAAATTGATACAATTACCTCTTGGTTACAGACTCAAAGTGATACAACTAGACCCTGGATTAAATCTGACTCCCAGTCTGTCAATTCCTGGGGTCATGCTGACATAGGTATAGTTCACGATTGGATTCAGCAAAGAGGTACTGCAAATCAACCCTGGACCTACCCTGAACCTACCCAAACAGTCAGACCCTGGGTGAAGCTAGAAGCTGATACACTTATATCTTGGTTGCACATTCAAATAAATAAAGTCAGACCATGGACCAACTCAGAAGATCAGATATTTAGCTTCAGGTTGCAGCCTGAAGTTGGTATGGTTCACCCTTGGATCAATCCTGAAACCAAAACAGTCAGATCCTGGGCCCACTCTGAAACTGATTTTATTGCATCCTTTGCTATAGATGAGCCTAACGAAGTCAGAACATGGATCCATACTGAAGTAGAGATAACATCTGACTGGCATGATAGCATTATATCATTTGTTCCTTCTGAAATTGAGCCAGATGGAGCAACTTTCTTAACTAGTGATTTCAATTCCTGGTCTAAACATATACCTTTTTTACCAATAGAATTAATTCTTTCCCCAGATCATTATTTTATAGGTTTCTCAACTGAGATAGCAACAACAGAAAGCCAAAATGAAATTAATTCTTTCCAATTGAGTGAGCTCACAAACATTTCCTTTCTTAAACTTCTGAGTACATGGCTTCCTGAAAGACTTGATTACCCGAACTTTGGCAATAAATTACAAGTTATCAAAACAAATGGAAGCCCTGATGTACCATCTACTTCTCTTTACCCCATCTCTCCATCATTTTCCTTTCTTGTTCCTTGCTCTCTCTCATCCCCATGTGCATTGTCCCACTCATGTTTGGTCATTTCTTCTTGCATATTCCCTTTATATTGTACTTTCCCTTCTTGCTCCATTCTTTCTCCAGGAGTCTTCTCTTCTATTCTCTTGCCCTTAGACTCTTCTTCTGACAGCTCTGACAAGGAACTGTCTTCCCTAAAATTTACTGAAGAGACCATAATTTCTCATACTTTTTCATATCTGCTTGCTGCTCCAGCCACATTTATAACAAAAGAAACTTCTCTGATGCCTGGATCTCAATCTGGAACCAAGTCTAATCAGCCTGAACAAGATCATATCAACTATTCAGAACTCAATATTTCTTTGGCTGAGTGTCACCTGGGTGTGATCTGGAAAGAGACTATCCAGGCTTTCTGGCTCTTCAAGACAGCTGTTATTTCTCATGAAACCACAGAGTGTGGATTACGACCTGGCCTTGCTCCTCACTGTCCAAACTGTTGGGAAGCAGAAGTGGGTGAATTTCCCTGGATGGTTTCTGTGCAACTCTCTTTCTCCCATTTCTGTGCTGGCTCTATACTGAATGAACAGTGGATCCTTACCACAGCTAGATGTGCAAATTTCATAAAAAACTCAGAAGCACTAGCGCTGGTCCAAGTGGGGCTTGTTGATCTTCAAGAGCCTGTTCAAGCTCAAACTGTAGGCATTCACCGTGCCATGCCCTACCTAGGTCCCAAGGGACCTCTAGGCCCTGGGCTAATCTTCCTGAAGCAGCCACTACACTTTCAACCATTGGTGCTTCCTATCTGCCTGAAGGAGAATGTGGAGCAAGAGAAAAGTATACAGCTTTATGACTGCTGGCTACCCAGTTGGTCCCTCATGAGAGGAAGTCCTGGGATTCTGCAAAAAAGGCACCTAAGTATACTGCAAGTCAGCACATGTGCCCAGTTTTGGCCCAAGCTGAATGAATTCACTTTCTGTGTGGAAGCCAAGAAAGCTATAGGGGAAGCTGGCTGTAAGGGTGACTTAGGGGCACCTCTTGTATGCCATCTACATCAAAAGGATACATGGGTACAGGTGGGAATTTTGAGTCACTTCGATGAACATTGCACAAAGCCCTACGTCTTCAGTCAAGTGAGCCCTTTCCTTTTTTGGCTTCAGGGAGTTACACGGCCCAGCCATGCACCATGGTCCCAGCAAGGGGCCATGACTACTTCTGCTTCCATCTCCCTTTCAGTCTCCACTTCTACGAACATCTCGGCTTTTACTCCCACTCCTGCTTCTATTCGGCCACAGTTCATCTCTCTGCCACAGCCTCAGACTTTGGCAGATCGAATTTCTCTACGATATGCCATGCCTTGGCAGGCCATGATAATCAGTTGTGGCAGTGAAATTTGCAGTGGTTCCATTGTTAGCAGCTCTTGGGTTCTCACTGCTGCTCATTGTGTCAGGAACATGAATCCTGAAGACACTGCTGTAATAATGGGCCTGAGGCATCCTGGTGTACCTCTGAGAGTTGTTAAGGTGTCTAACATTCTACTGCATGAGAGATTTCGGATGGTGAGTGGGGCAGCAAGAAATGATCTAGCACTGCTGCTCCTTCAAGAGGTCCAGACTCCCATTCAGCTTTTAGCACCATTGGGACATCTAAAGAACTTGAATAGCTCAGAATGTTGGCTTTCTGGGCCAAGAGTTCTCAAACCAGGAGAGACAGATGAGAGTCCAGATATGTTACAGATGCAGGTAATGGGAGCTTCAAGCTGTGCCCACCTCTATCCTGACATTGGCAGTTCTATTGTCTGCTTCATTACTCAGGAGAAAATCTCTGATAAAAATGTGGAACCAGTGACTCCAGGCAGTGCTGTTATGTGTAGACCAATATCTGGAAATGGCAGCTGGAGACAGATAGGCATCACCAGTCTAAAGGCACTTGCTACCATTGTCAGCCCACACTTCTCCTGGATATTATCCACATCAGCAAAAGCAGGTCATCCCCTAAACCAGGCCCTCATGGCTTGGGCAGAGAAGCCAAAGTCATCTAGTCTTCATGAAGAGCCAACAACACTACAATTTTCATTATTAATGATTATTACAGTCCAGAGTCTGTTGTAAACCAGTGCTTATAATGACAACTATTACTTTGGGCATTTTGTAATTTAAAAAGATAAAAT

>Fruit Bat, Rousettus aegyptiacus (BK0591511)

GCTAGACTCAGTCTACAGTTTTCTCAGTCATGTTCTGGATGCAATTTGATTCTCTATTAAATTCATACACACAGAGTCTAAATGATACAGTCACATTGTGGACAATGATTAAAACTGAGTCTAAGCAACCTTGGCGAGTACCTGAACCTAGTATATTTAGTATTTCATTGCATCCACAAATTGATACTTCTCGATCCTTGATTACAGTTGAGAATCAAGCTTCCTTTCTTTGGACACATCCTAAGACTGATAATATCAACTCATGGGCCTTGTCTAAATTTGGAACATTTATATCCTGGGTAGTGCCCTTGCCTCAAGCAGCCAGACTCTGGCTCCCCTCTGAAGCTGATATTAGCAGATCTTGGTTGAAGACTGAAACAACAAGAGTAAGACCCTGGGGCCAATCAGAATCTCAAACAGTGAGTACCTTGACTCAGTTTGGACTTGGTAGAGTGGATCTCTTGGCTCAACATGAAACTGGAACAACCTTATCATGGATTCAGACTAAAACTGGTGTATTTCATCCAGGGAACCGGTCTAAAGGAGACACAGTGGGACCTTGGACCTTTTCTGAAGCAGATATAGTAAGACCATGGATCAAAAATGAAGCTGGTGTAATCAGTCTCTGGACTCAACCTAAAAGTAGTACTTTCAGACCTTGGGCCCAGCCTAAATCTGAAGCAGTGAGGCCCTGGACACAATCTGAAGCCACTATGTTTACATTATTGTCCCCATCTGAAATGCAAGCAGCGAAACCCTTGAATGTGACTGACATTAATATGGTCAGATCTTGGTTCCAGACTCAGTTTGGTGCAATAAAACAAGGCACTCAACCTCAATCTCAAATAGCTACTCCCAGGATCCAACCAAAATGGCAAAGAGCCCACCCATGGATCCAGGACAAAACTAATACAGTCAGATTTTGGCCTCATCCTGAAGGTGAGTTAGCCCAACTCTGGACTCAGACTGAAACCAACACAGTCAGACTCTGGACCCATCATGAAAGTGATACAGTAAAACCATGGACCCAGCCTGAGCTACAGTTTGTCAGTTCCTGGACTGATGTGGATACAGTCACATTTTGGTACCCAATGCAAAATGGTACAATTAGGCCCCAGTACCCATCTGAATCTCAAAAACCGTGTTCCTGGACACAAAATGAAGTTGATATAATCATCCCTTGGACTCAGCATGAGAGCAATTCAATCAGATCTTGGACTAAGCTTGAAACTGCTACAATCCACCCCTGGATCCACCTTGAAACTGCTGCAATCAGACCATGGACCTTGGCTGAAACTTTAAAAATCTACCCCTCAACCCGGGTGGATACTAATACAATAATAAGACATTGGTTTCAGATTCAAATAGATTCAGTCAAACCTTGGAATCTACAGGAAGCTACTACAGCTAAACCCAGTACCCAGCCTGAAACTGAAACACTCCATGTTTGGTTGCAGACTGCAAAACAACTAGTAAAACCTATAACTTTATCTGAAGTTGATACAATTACTCCTTCATTACAGACTCAAAGTAATACAAACAGAGCTTGGATTCAACCTGACTCTCAGACAGACAGTCCCTTAACTCAGGCTAACGTTTCTGTAGGTCACCCCTGGACTCAGCAAGGAGCTGTTAAAAATCAACCCTGGATGTATGCCAAAACTCAAGCAGTCAAACCCTGGGTCAATATGGAAGCAAATACAGTCAGATCTTTGTTCCACATTCAAATGAGTAAAGATATACCATGGACCCATTCACAATTTCAAGTATTCAGTCCACGGATCTACCCTGAAGTTGGTATAGTTGAGTCTTGGATACAGTCTGAAACCCAAGAAGGCAGGCCCTGGGCCCACCCTGAAACTGACATTATTGCATCCTCTGCTATACTTACACCTGACAAAGTCAAAACACAGACTCCACAAGAAAAAGAAAGAAAGCCTGACAGCCATTATAAAGCTGATATAATTACATCATTTGTTCCTCTTGAAGTTGAACTGGATAGAGCAACTCCATTAACTAGTCAGTTTGGCTCCTGGTCTAAACCTGTACCTTTTTTACAAACAGAAACTATTCTTTCCACAGATCAGTATTTTATAATAGCTTTGTCAACTGAGATACCTGCAGCAAAAAGTCAAGATCAAATCAATTCTCTCCTACCCAGTGAGCTTACAAACATTCTCCCTCTTACCCTTTCAAGCACCTGGCTTCCTGGAAGAGTCGATTATCTGAACTTTGGCAGTAATTTACAAATCACCAAAACGAAAGAAAGCCCTTATGTTCCATCTAGTTTTCTTAGCCCTCTTTCTCCATCTTTTTCCTTTCTTGTTCCTTGTTCTCTGCCAACTCCATGTGCATTGTCCCCTTCCTGTTCATTGTTTTTTTCTTGCACATTACCTTCATCCTGTATTTCCCCTTATTGCTCCATTCTTTCTCCTATGGCCTTCTCTCCTGTCCTTTTGACCTTAGCCTCTTCTGATAATTCTCCCCAGACACTATCTGGGGAGACCTCAAAATTTACCAAAGAGATCTTTCTTTCGCACGGTTTGTCATCCCTGCGTGCTGCTCCAATCACACTTTTAACAAAGCAACTTTATCAGATGCCTGGATCTGAATTTGAAACTAAGTCTAAACAGCCTGAACAAGAGCCTCTCAAGAATTCAGAACTCAATGTTTCCTTGGCTGAGTGTCATCTGGGTTTGGTCTGGAAAGACAGTCTTCAGGCCTTCTGGCTCTTCAAGACAGCTGTTGTTTCTCATGAAACCACAGAGTGTGGATTACGTCCTGGCCTTGTGCCTCAGTGTCCCAACTGCTGGGAGGCAGAAGTTGGTGAATTCCCTTGGATGGTTTCAGTGCAACTCTCTTTCTCTCATTTCTGTGCTGGTTCTATACTGAATGAACAGTGGATCCTTACCACAGCTAGATGTGCGAATTTCATAAAAAATTCAGAAGCCTTGGCCCTGGTCCAGGTGGGACTTATTGATCTTCAGGAACCTACTCAAGCTCAGACTGTAGGCATTCACCGTGCCATGCCCTATGTAGGTCCTAAGGGACCTTTGGGACCTGGACTAATCTTCCTGAAGCAGCCACTGCATTTTCAACCCCTAGTGCTTCCTATCTGCCTGGAGGAGAGCCTGGAGCAAGAGAAAAATATACAACTGTATGACTGCTGGCTACCCAGTTGGTCTCTTATGAGGGGAAGTCTTGGAATTCTGCAGAAAAGGCACCTAAGCATCCTGAAAGTCAGCTCTTGTGCCCAGTTTTGGCCCAAGCTAAATGAATTCACTTTCTGCGTTGAAGCCAAGAAAGCTGTGGGGGAGGCTGGCTGTAAGGGTGACCTAGGGGCACCTCTGGTGTGCCATCTGCAACAAAAGGACACATGGGTGCAGGTGGGAATCTTGAGTCACTTTGATGAACATTGCACAAAGCCCTACGTCTTCAGCCAAGTGAGCCCTTTCCTTTTCTGGCTCCAGGGAGTTACACGGCCCAGCCATGCACCCTGGTCCCAGCAAGGGCCAATGACTACTTCTGCTTCCAAATCCCTTTCAGTCTCAACCTCTACGAATGCCTCAGCTGTTACCTCCACTACCACGTCTATTCGGCCACACTTCATCTCTCTGCCACAGCCTCAAACTTTGGCAGATCGGATTTCTCTGAGGTATGCCATGCCTTGGCAGGCCATGATCATTAGCTGTGGCAGTCAAATTTGTAGTGGTTCTATTGTTAGCAGCTCTTGGGTTCTCACTGCTGCCCACTGTGTCAGGAATATGAATCCTGAAGACACTGCTGTAATACTGGGCCTGAGGCACCCTGGGGCACCTCTGAGAGTTGTTAGGGTATCTACTATTCTACTGCATGAAAGATTCCGGTTGGTGAGTGGGGCAGCAAGAAATGATCTAGCATTGCTGCTCCTTCAAGAAGTCCAGACTCCCATTCAGCTCTTAGCACCTTTGGGCCATCTGAAAAACCTGAATAGCTCTGAATGCTGGCTTTCTGGGCCACGAATTCTTAAACCAGGACAGACAGATGAGAACCCAGAAATGTTACAGATGCAGGTGACAGGAGCTTCAAGCTGTGCATACCTTTACCCTGATATAGGGAGTTCTTCTGTCTGCTTTATGACTGAGGCCAAAGGCTCTGTCACAAATGCGGAGCCAGTGAGTCCAGGCAGTGCTGTTATGTGCAGACCAATATCTGGCAATGGCAGCTGGAGACAAATAGGCCTCACAAGTCTGAAGGACCTAGCTACCATTGTGAGGCCATACTTCTCCTGGATATTAACCACCTCTGCAAAAGCTGGTCATCCCATAAATCAGGCCCTCATGCCTTGGATGGAAAAGCCGAAGTCATCTGGACTTTTAAAACAGCCAAACACACTATCACTTACTTGTGTAATGATTATTGTACTTCAGAGTCTTTTGTAACTCAGTAACTATGAAAGGCATTGCTAGTATTTTGAAAATGTGTAACATGAGACATAAGTAACACATAGTCAAAAACATTGAATTTAGGCATGAGAGGATCTTCTAGATATTTTTTAAATAAAGACATACTAGAGATCGGAAGAGCGTCGTGTAGGGAAAGAGTGTTAAGAT

>Giraffe, Giraffa camelopardalis

ATGAAAGGTCCACTTTGCCTCTGTCAGTTTTGGGGGCTGTTTTTCCACTGCTATGGTGTACCCAATTCTCAAAAGCAGTGGTTAACCTCATTGACCCCAACAATTGCCCCATTGTCAGCGTCCAACAACATCCCATGGCTAGTGTCCATGGCTGAGACCTGCCAGGGCATTATCCTGAGTCGTTGGTGGATCCTCTCCACGGCCAGCTGTCTGACTAAACTGAGACAGTTAAACTCTGACATTTCGGGAGTCTTTGACCAAGAAGATGTCTTACGAGGCCACAAAATATGCCTACTCCCTAGTTTTGATCCAGAAACTGGAAAAGATCCAGTCAAAGCAGATGTAGGGGTGGTACTCCTTCAATATCCTATCAGGAAGAAAGAAATACCACTTTCTCACACTTATAACATCTTCTGGAAGGCCTGTTATAACTGCCAATACAGACACTGCAGGGTGTACCAATATCAGAATCATGGTAACTTTGAAAGCAATATCAAGAAATTGTCAGTTAAGCTGCTGGACCTCTCATTCTGCCGCCATCAAAACATTCACCTGACTAAAAGTAACAATTTATGCATCTGGAGTCAGCCAGAAGAAGACTGCTGGGTACAGCAGGGCAGTCCTGTTTTCTGTGTCTTTGGCAACCACTTGGAACTCGTAGGCCTGGTCAGTGAGTCCTCAATGGCCTGTTATGACCCAATTCTTGTCATCAAGACAGCCCCCTACCTACCTTGGATGAGAAGACTTATCAAGGCATCCCAGAAGTCACTGGATCCTATTTTTTCTCTACCCTGCAGTTTTTCTTCTAGGGTAGAACATGGTAATCAAGATAGGCTTAGCCCAAAAAGTGCTTTTGCATTTTTGTCCTCCCATGGATTCTCTATACAGTCGTGGAGGGGGAAATTTGGCACTTTTCCACTAACCAGACAGCGCCAGAATCATCCTCCTGTATTTTTTGGTTCAAATAATAGAGACTCTTTTCCTGGAAGTTCACAGTTATACCCTCAAAGCAGTCAGATCTCCTCAGCTAGCAATTTCCTAATGGTACAATCTTGGACGTCTCCTCTTGTCAAATCATGGGATCCTTTTCATACAGCTGTGTCCTGGAATACTGCCGTAACTGATACATCTAATCCTTCAGTTCTTCATCAGCCTCGAAACACTCCAGCACCAGATGTATATACACCCTGGAACCTTCCTCAGAGAGATGCAATGAAATATAAATATAGAAATATGACAGATTCATTAAGATACTGGGTTGAATCTTTAGGTGGTATAATTGGGTTTTATACTCCACCATTAGATAATGCTGATGGATCACATATTCTGTTTTCAAGTGATGTAGATGGATACCAAGTTCATTCTGAGATAAATAATGTTCAGTCTCAAGTTCAGTATGGTATGATTCCTTCCCAAGGTCAATTTCCAGGAAGACCCTGGCTGCATATTATATCTGGTGTTGGACCTTTGACACATAATATGCCTGATAAAACAGGAATAGTGATGCAGATTCAACCTGCTGAGGAGAGTTTTAGAACTCAAACATATCATGCTGCTGATAGAGTTAATACTGTTATTCAACCAGTAGCTTATAAGATTAATCCATGGGTCCCTTTAATTGTTAATAAGATTGGATTCTGGACTCATTCCATACTAAATGAAGAGAGATCCCGATATCCGACAGTAACTCCTACATCAGAACCCTGGTTTCAGACTGTCCTAAATATAGTTAAATCCCAAGGACCTATAGAAAGGACAGAAGAACTCAGACTTCTCCCTGAATCTAAGTCAACTCAATTTTGGACTTCCTCAACTGTTAATATGCTCCTTAATTGGGCTTCATCCACAAGCAAGACTAACAGGCCTGTGGCCCAGTATATGGCCAGTACAATCACATCCTTAAGTCAAATGGATAGAATGAATCCATTGAACGAGTATGGTGCTTTTACAGTCAAAACCCAGAAGCAGACTGATGATACAACCTGGCTTTTGATTCATACAGTTACTAGTATAGTTGATCCTTTAATTCACTCTGAAGTTGATACACCATGGACTCAGCATGAAGCTGATGTAGTCCAAACTTGGACCTGGTCAAAAACCCAATCAGGAAGCCTTATGAATCTGCTGAAAGCTGATACCACCAAACCATGGTTACAGACTAAAGGAAGAAGACACTGGATTCAACCTAAACTCCAAAATCTCAGACCCAGAACTCAAACTGAAGAAGGTAAAAATAAACTTTGGATCCAGTCAGAAGCAGATGCAGTCATATCCTGGACCCACCATGAAATTGAAACACTCAGACCCTGGGACCAACCTGAAATTGGTACTGCCAGACCTTTGTTCTTGACAAAATCTGACCAAATAACACTGAGCACTCAGGCAGCCTTTAAAACACTCCACATATGGGCTCAGCCTGAAGTTGACCCAGTCAGACCATGGATTCAGTCTGAAGGTGGCACCATCCAACAACGGACAAAGCCAGAGGAATCTCCTTTCAGACTCACAACAGAGTCTAAAGTTAATACAACTACACCATGGACAAGACCTGAAGCTGATGCTATCAGAGTTTGGTTGCAAACACAAACTGATATAATCAGAACCTGGACCCAAACTGAAACTCAAACAATCATTTCTTGGTTTGAACCTGAAGCTGTTCCTGTCAGACCTCCGTGGAATACTCAAACTGATACATTTAAACCTTGGATTGAGACAGAAATAGAAACAGCCCATTCCTGGGCCCAATCTGAAGGTAATATAGACAGATCATGGACCCAGACTGAAGCTGAAAATGTCAAATGGTTCTGGACTCAAATGGAAACAGTCACATTTTGGACAGAGCCAGTAATCAAAACATCCCACAACTGGATACAATCCAAAAAGGAAATAGTCAAGCCCTGGAACCAATTTTATGAGGTTAAAACCTGGACGCAACATGATACTAAAACACTCAAGCCATGGAATGAAAACAATAAAGATAGATTTTGGACTCAGTCAGAAGCTGATACAATGAGACCCTGGATTCAGTCAGATATTGCTATCAACAACCCTGGAGCAGAAAATGAAGCAGGTGCATTCATGCTTTGGACTCAGGCTGAATATCCAGAAGTAAATCTCTGGATACAATCTGACACTGACACAGTGTCATTGTCAACCAATAGTAAAATTCCAGCAGTATATCACTGGACAGAAATACCTGATACCACCATACCACAGGCAAAGGCTAAATTTCCAGGAGTAAAGCCCTTGACACATCTGGTATCTAATACAGTAACACCACGGACTCAGACTGAATCTATAGTAGTAAACCTCTGGACTCAGCCTATGACTGATACAGTCACACAGTGGACTCCAGGTGAGTTTTCAGAAACAAATCCCTGGACAAAAACTACAATTGACAGAGTCAAGTCATGGACCCAGGCTGAATCTCTAGTAGTAAATCCCTGGGTGCCATTTGACCCTGATACAGTTACACCTTGGGCACAGACTGACTCCTTCATAATAAATTATTGGACACAGTCTATACCTCATACTATTACACAATGGACCCACAGTGAACCTTTAGATATAACTCCCTGGACAAAAGCTGTCACTGATGCAACACCTTGGTCTCAGGCTGAATCATCATCAGTAAATCCCTGGACAGATAGTGCAGCTGGGACAGTCACACAACGGACCCAAAGTGAATCTCCAGTAGTAAATTTATGGACTCAACCTGTGGTTAATGTAGTCACACTGTATACTCAGGCCGATTCCCCATTATTACATTTCTGGACAAAGTCTGAAATGGATATGGTCACTGCATGGACCTCAACAGAATCTTTAGCCATGAATTCCTGGAGACAGTCTGAAACTGACAGTATCTCAACATGGACCAATCAAATAAATCCAGACATATACAACTGGGTACCTTATGAAACTGATGTAGTCACAGTTTGGACCCAGTCAGAAAGTCCAGAAATAAATACTTGGACACAAGCTGTAGCTGATGCAGCTCCACCATGGACTCAGGCTGATTTTTTAGCTCTAAATCCCTGGACAGAAGTTGAAAGTGATACAATATCATCATGGACTCAGGTTGAATCTTTAGTGAATCCTTGGACAGAGAGTGTAGCTTCCACAGTCATACCATGGACCCAGGCTGAATCTCTAGCAGTAAACCCCTGGACACAATTCGCAGCTGATGTAGATACACTATGGACCAAGACTGATTCTCTAACAATAAATCCCTGGATAGATCCTGCATCTGATCGTGTTATACAGTGGATTCAAACTGAACTTCCAACAGAATTTCAGTGGATTCAGAATATGTCTGATATAGTGACATCAATTACCCAGGCTGACTTTCCTGCAGTAGAGACTTGGACAGATCCTCTGGTTGATATAGTAACAATGTGGAATCAGACTGAATCTTCAATAATAAATCCATGGACAGAAGCTGCAGTTTCTACAATGACACCATGGACTCAAATTGAATTTCCAGCACTAAATACCTGGATAAATTCTATAGCTTCCACAGTCACACAGATGACTCAGGCTAAATACCCAGTAGTAAAGCCTCAGAGACAGTCCATATCTGGTTCAGCCACACTATTGAACCAAATAGAATCACCTATAGTAAATCCCTGGACACAGCATGAAACTGATGTAATCACACAGTGGACTCAGACTGAATCTGAAGAAATACATTCTTGGATACAACCTGTAACTGACACAATCATACGCGGGACTCAGGCTGAATCTCCAGTCATAAATTCCTGGACAGAGGCTGTTGCAGATACAGTAAGAACACTGAATCAGATTGAATATCCTTCAGTAAATCCCTGGGAAACAATTGCAACTGATACCAGTACACTCTGGACCCAGGCAAATTCTTCTGTCATCAATCTTTTGACACAGTCCATAGTTCATACACTCACAGCCTCGATCCAGACTATGTCTGTAGCAGTAAATCCCTTGTCTCAGTTTGAAACTGATAGAGTCACACAATGGATCCAAGCTCAATTTGCTTCAGTAAATCCATGGACTCCGTCTGAAGTCAATCCAATTACAGCATGGGCCCATTCTAGATATTCAGCATTAAACTCCAGGACACAATTTGAAACTAGCACATTCACATCACGGACCCAGGCTGAGTCTCCAGTAATAAATATAGCTTTATCTAGATCAGTCAAACCATGGATCCAGGCTGAATCTCCTGCAACACGTCCCTTAACAACAGCTGTAGCTGGTGAAATGACACCATGGATCAAGGCTAAATCTCCTGCAGTAAATCCCTGGACAAAATCTAAATATGACGTAGTCTCACTGTTGATCCAGAATACAACACCAGCAGTAATTTCCTCAACAGCGTCTGTAATTGATACAATGACCCTGTTGAACATGGCCAAAATTACATTACTAAAGCCTTGGTCACAGTCTGTGACAGAAACAGTCACACATTGGATCCAAAATGAGTTTCCCCTGGTATATAACTTCACACGAGATATAACTGATGTAACCACACTGTTTACTCAGGTTTTTACGCCAGAGGTAAATCCCTGGTCAAGGCCTAAAGCTGATACAGTCACACCATGGATCCAGGTTAAATCTCCAAAGGAAAAGGCCTGGAAAGTGGCAGGTTCTGAAACACTCACACCGTCGACCATAGGATTCTCTACAGCAGTTAAAGCCCAGATAGAGACTGTGTCTGATATAGTCACACCAGGAACCAAGTACAGTTTTTCAAAAGTAAAACTTGGGAAATTGCCTGAGGCAGGTGTCACTGAACCATGGACTCAGTCTGAATCTCTAGCAGGAATTCTCTGGACAGGACCTGCAGCTGACTCAGTCACAAAGCAGATACAGACTAAATATCCATTTGTAAATCTGTGGACTCAGGCTCAATTTCCAGTAGTAAATCCCTGGACACAGTCTAAGTCACTACCAAGAAATATTTTGACAAAATCCAAAACTCAAGGTGTAAACCTATTAATACAGCCTGAGACGACTACAGTCACATTATGGAGTCAGGTTGACTCTCCATCGGTAAATCACTGGATAAAACCTGTATCTTCCATAGTCATACCATGGACACAGGCTAAATATTCAACAGTACATCCATTGATGTGGACAGCAGCAAATTCAGTCACACAGTGGACCCAAAATGAATCTTCAGCATTGTATTCCTGGACAAAGACTACAGTAGATACAGTCACAAAATGGGCTCTGGGTGCATCTCCAGTAGCAAATTACCAGACACAACGATTAGCTTACACAGTCCCATTTTGGGCCAAATATGGAACATCAAGGATAAATGCTTGGATACATTCTAATTTTGAGCCATTTCCACCATCAACACAAGTAGAATCTTTAGCAAATAAATATGGGATACATATAGAAAATTATAGAGTCATAACCATGCCCCAAGTTGAACCAAATGAAGTGAATTTCAGACCAGAGTCTATACATGTAGGGGGAAGAAAATGGACTAAAACTCAATTTTCCATAATTCATTTGACAGGGACTGTATCTTTCACAACCCCATCATGGACAAAGGCTGAAAGTTCAACTGCAAATACATGGAGAGAGACTGAGTCGTTAACATCACCAGCATTGACTCAGGCTATAAATCCAGCTATAAATACCTGGAAAGAGACTGTCTTTTTTACAGGCCCAACATGGACACAGGTTGAAAGTTCAGCTGTAAATACGTGGAGAGAGAATGTGCCTTTCACAACCCCGCCATGGACTCAGGCTGGAAGACTAGCTATAAATATCTGGAGACAGGAAGTGCCTTTCACAGGCCCACCATGGACACAGGCTGAAAGTTTGTCTATAAATACCTGGAGGGAAGCTGTGCCTTTCACAGCCCCAATATGGACTCCAGATAAAGGCACAGCTGTAAATAACTGGAGAGAGACTGTGCCTTTTACAGTCCCACTATGGACTCAGGATGAAAGTCTAGGTGTAATTACCTGGGGAGAGACTGTCCCATTCAGAGCCCTACCAAGGACACAAATTGAAAGTCCAGATGCAAACACCTGGAGAGAGACTGTTCCTTTCACATTCCCACCATCGACTCAGACAGAAAATCCAGCTACAAATACTTGGAGAAAGGAAGTGCCTTTCACAGACCCACCATGGACACAGGCTGAAAGTTCAGCTGTAAATACCTGGAGAAAGACTGTACTTTTAACAGACCAACCATGGACACAGATGGAAAGTCCAGCTGTAAATACCTGGAGTGAGACTGTTCCTTTGGCAAACCCACCATGGACTCAGATGGAAAGTCTAGCTGTAAATGCCTGGAGAGAGAATGTGCCTTTCACAGCCCCACCATGGACTCAGAGAGAAAATCCAGCTATAAATACCTCGATAAAGACTGTTCCTTTCACAGTCTCACCATGGACTCAGGCTGAAAGTCCAGTTATACTTACCTGGGGAGAGACTGTGACTTTCACAGCCCCACCATGGACACAGGCTGAAGGTCCAGATGTAAAAACCTTGAGAGAAACTGTGCCTTTAACTGGCCAACTTTGGACACAGGCTGAAAGTACTGCTCTGCAGACTTGGAGAGAGACTGTGCCTTTCACAGCTCCATCGTGGACTCAAGATAAAGGTCTAGCTGTTAATAAATGGAGAGAGATGTTGACTTTCACAGCCCCACCATGGACACAGGCTGAAGGTCCAGCTGTGAATACCTGGAGGGAGACTGTGCTTTTAACAGGCCCACCTTGGACTCAGGCTGAAAGTAGAGCTGTAAATGCGTTAAGAGAGACTGTGCCTTTCACAGCCCCACCATGGACACAGGCTGAAGGTCCAGATGTAAATAACTGGAGAGAGACTGTGCCTTTCACAGCTTCACCATGGACTCAAGTTAAAGGTCCATCTGTAAACAACTGGAGAAAGATGTTGACTTTCACAGCCCCATCGAGGACACAGGCTAAGAGTCCAGGTGTAAATACCTGGAGGGTGACTGTGCTTTTAACAGGCCCACCTTGGACTCAGGCTGAAAGTACAGCTGTAAATGCTTTAAGAGAGACTTTGTCTTTCAAAATTGCACCATGGATACAGGCTGAAGGTCCAGTTTTACATAACTGGAGGGAGATTGTGCCTTTCACAAGCCCACCTTGGACTCAGGCTGAAAATCCACCTGTAAACACCTGGAGAGAGACTGTGCCTTTCACAGCCCCAACATGGACTCAGGAGGAAAGCACAGATGTAAATACCTTGAGAGAAGCGGTGCCTTTCACAGGCTCACACTGGTCTCAAGCTGAAAGTACAGCTGTGCATACTTGGAGAGAGACGGTGCCTTTCACAGCTCCACCATGGACTCAAGATAAAGGTCCAACTGTAAACAAATGGAGAGAGATGTTGACTTTTACAGACACACTGTGGACACAGGCTGAGGGTCTAGCTGTAAATACCTGGAGTGTAACTGTGCTTTTAATGGGCCCACCTTGGACTCAGTCTGAAAGTACAGCTGTAAATACTTTAAGAGAGACTGTGTCTTTCACAGTCCCACCATGGACACAGGCTGAACGTCCAGCTGTAAACACTTGGAAAGAGACTGTTCCTTTCACAGCCGCACCATGGACACAGGCTGAAAGTCCAGCTGTAAATAACTGGAGAGAGACTGTGCCTTTCACAGCCCCATCATGGACTTGGATAGAATGGCCAGCTGTAAATTCCTGGAGAGAGACTGTGCCTTTTACAGCTTCACTATGGACTCAGGATGAAAGTCCAAGTGTAATTACCTGGGGAGAGACTGTCCCATTCAGAGCCCCACCAAGGACACAGATTGAAAGTCCAGATGTAAATACCTGGAGAGAGACTGTGCCTTTCACAGCCACACCATGGACACAGGCTGAAAGTACAGATGTAAATACCTCGAGAGAAGCTGTGACTTTTACCAGTCCACCTTGGACTCAGGCTGAAAGTCCAGCTGTAAACACCTGGAGAGAGACTTTGCCTTTCACAGCCCCACCATGGACTCAGGATGAAAGTCCATCTGTAAACACCTGGAGAAAAAATGTGCCTTTCTCAGCTCTACCATGGACTCAGGCTGAAAGTCCAGCAGTAAATACCTGGACATGGAATGCCCCTTTTACAGCTGCACCATGGTCACACACTGAAAGTCCAGCTGTAAACACCTGGACAGAGGCTATGCTTCTCACAGCCGCTCCATGGACTCAGGCTGAAAATCAAGCTACAAATACCTGGAAAGTGAATGTGCTTTACAGAGGCCCGCCACGGGCTCAGTCTGACTCTGCACCAGCAAACCCTTGGACATCTACTGAAAGTTTTAGAATTACATCATGGACTCACACAGTAAAGCCAGTTTTAAATATGCAGACAGAGCCAATACTGCGGACTGAGGCTGAATATTCAACAACAAAATATTGGACAGAGACTAAGGTCACTTACATAGTCACACCATTGACCCAGTGTCAGTTTTCAATAAATACTTTGATAGAATCTTTAGGAGCCATGATCACACCATGGAAATCTGCTGAAGCTCTAGCATTACATTCTTTCACACAAAATATTATTGATACAATCGAATTTTGGCCAATGCTTAAAACTGAGTCTAAGAAAAGGTGGCATCTGCCTCAAACTGGTACACTCATGTTTTCACTAAATCCTCAAATTGATACTTTTGGATCCTTGAACCAAATTGAAAATCAGGAATCTCCTCTGTGGACACATCCTGAAATTGATAATGCCAATACAGTGACCCTTCCTGAATCTGGAACACTCATATCGCAGGTAGTACCTTTGCCCCAAGCAGCTAGACTCTGGCCCCAAAATGAAGCTGATATTAGCAGAACTTGGTTTGTATCCTCAGAAAGAATAAATTCCTGGGCCCAATCAGAGTCTCAAAGAATGAGTACCTCAACACATTTTGGAGTGGGTAGAGTGAAGCCCTTGGCCCAACATGAGACTTCTATAGTCATGTCATGGTTTCAGATTGAAACTGGTATATTCCACCCTTGGAACCAGTCTGAGGGAGACACAGTGAGGTTCTGGCCCCTTTCTGAAACTGAGGAGGTAAGAAAATGGATCCAAACTGGAGCCGGTATAGTCAACTCTTGGACTCAAGCGAGAAGTAGTGTAGTCAGAGCTTGGATCCAAGCTGAATCTGAACTAGTCAGACCCTCGACACAATCTAAAACTAATGCAATCACACTATTGACCCAGGCTGATACTATCAAATCTTGGTTCCAAACTCAAATTAATGCAGTAAGAGAAGGGACCCAAACTCAATCTCAAATTGTTGCTACTTGGATCCAAACACAGTTGCAAACAGTTTACCCCTGGATTCAGCCTAAAAGTGATTCAATCAGAATTTGGACCCAGCCTTGGATCCGAGCTGAAACCCACACAGTCAGACTCTATTATGAAACTGATATAAGAAATTCATGGATCTCATCTGAATCTCAGTCAGTCACATTTTGGTCACTGAGTCAAAATTCAATTAGGACCTCATTTCACTTTGAATCTCAGATGACATGTTCCTGGGCCCCAAATGAATTTGATATAATCAGTCCTTGGACTCAATATGAAACTAGTTCTGTTGGATCCTGGATCAAGTCTGAAACTGGTACATGTCAACCCTGGCTCCATATCGAATCTCCTAAAATCACACTATGGACCCAATATGAAACTTTAGAGATCTACCCTTCAACCCAACCTGAGACTGATTCAGCAGTAAGGCATTGGTTCCAGCCCCAAATTGATCCAATGAATACTTGGAATCAGCCTGAAGTGGATACAATCAGATTCTGGACCCAAGCTGAAACAGAAACAATTCCAATCTGGACCCAGATTGGAAGTCAGGTAGTTAAACCTCCCAAATTATCTGAAGTTGGTATAGTCACACCTTGGTTACAGACTGAAACTGATGCAAGTAGATCCTGGATTCAATATGACTTTCAGTCAGTCCATCCTTGGCCCCAGACTGGATTTGGTATAATTAACCCCTGGTCTCAGCAAAGAGCTGTGGTAAATCAACCCTGGACATATGTTCAAACACAGGCAATCCGACCCTGGATCAATGTGGAAGCCAATACAATCAAATCTCGGTTTCATGTTCAAATGAAAAAAGTCAGACTGGGGATTCCTTCTGAGTCTCAAATATTGAGTTTCTGGATGCAGTCTGATGTTAGTAGAGCTAAAGCTTGGATCCAACCAGAAACCCAGGCAGTCAATCCTGGGGCTCACCCTAAAACTGGTAATGTTGCATCCCTGGCTATTCCTAAGCCTGAAAGAGTCAGAATGTGGATCCAGCCTGAGACAGAAATAAGGCCTGGCGTCATTTATAAAGCTGATATAATCACATCATTTGTTTCTCCTGAAATTGAACCAGATGGAACAATTAGTCACTTTGATTCCTGGTATAACCATGTAACATTTTTACCAATAGAAACTGTCCCTTCCCTAGATGAGTATTTTGCAGCTTTGTCAACTGAAATAGCTTCAGTAGAAAGCCAAGGTCAAACAAATTTTTTCCAACCCAGTGAGATCACAAATATTCTCTTTCTTACACTTTCAAGCACATGGCTTCCTAGAGGAGTTGGTTACCTGAACTTTGGCAATAAATTACAAATTACCAAAACAAAAGGAAGCCCTCATATCCCGTCTAGTTCTCTCAACCCTCTTTTTCCATCTTTTTCCTTTCCTGTTCCTTGTTTTTTCCCATCTTCATGTTCTTTGTCTCTTACCTGTTCAGTATTTTCTTCTTGCACATTTTCTTCACCCTGTACTGTTCCTTCTTGCTCAATTCTTCCTCTTGTGGCATTCTCTCCCATTCTTCCCTTAGCTGCTTCTGATAGTTCTCTCCAGGAACCATCTTTTTCAAAATTTACTGAAGACGCCGTTGTTTCCCATGCTTTTTCATCCTTACATACTGCTCCAGCCACTCTTTTAACAAAGCAACCATCTATGATGCCTGGATATCAATCTGGAACCAAATCTAATCAGCCTGAACAAGATCTTCTTAAGTATTCTGAACTCAATATTTCCCTTGCTGAGTGTCACCTGGGTGTGGTCTGGAAAGAGAGTCTCCAGGCTTTGTCGCTCTTCAAGACAGCTGTTATTTCTCATGAAATCACAGAGTGTGGATTACGTCCCGGCCTAGTTCCACACTGTCCCAACTGCTGGGAGGCCGAAGTGGGTGAATTCCCTTGGATGGTTTCTGTGCAACTCTCTTTCTCCCATTTCTGTGCTGGTTCTATACTGAATGAACAGTGGATTCTCACTACAGCTAGATGTGCAAATTTCATAAAAAACTCAGAAGCACTGGCTCATGTCCAGGTGGGGCTTATTGATCTTCAAGACCCTGCACAAGCTCAACCTGTAGGCATTCATCGTGCCATGCCCTACCTAGGCTCCAGAGGACCTTTGGGACCTGGTCTAATCTTCTTGAAGCAACCATTACATTTTCAACCCCTGGTTCTTCCTATCTGCCTGGAGGAGAACCTAGAGGAAGAGAAAAATATACAACTATATGACTGCTGGCTACCCAGTTGGTCTCTCATGAGAGGAAGTCCTGGAATTTTGCAAAAAAGGCACCTGAGCATCCTGCAAGTCATCACATGTGCCCAGTTTTGGCCCAAACTGAATGAATTTACTTTCTGTGTGGAAGCCAAGAAAGCTATGGGGGAGGCTGGCTGTAAGGGTGACCTTGGGGCACCTCTTGTGTGTCATCTACAACAAAAAGACACATGGGTGCAGGTGGGAATCTTGACTCATTTTGATGAACACTGCACAAAGCCCTACGTCTTCAGCCAAGTGAGCCCTTTCCTTTTCTGGATCCAGGGAGTTACACGACCCAGCCATGCACCCTGGTCCAAGCAAGGGCTCATGACTACCTCGGCTTCCATCTCCCTTTCAGTCTCTACCTCTATGAATGCCTCAGCTTTTACCTCCATACCTGCTTCTGTACGGCCACATTTCATCTTTCTGCCACAGCCTCAGACGTTGGCAGAACGAATTTCTCTGAGATATGCCATGCCTTGGCAGGCCATGATCATCAGTTGTGGCAGTCAGATTTGCAGTGGTTCCATTGTTAGCAGCTCTTGGATACTCACTGCAGCCCACTGTGTCAGGAATATGAATCCTGAAGACACAGCTGTAATATTGGGCCTGAGGCACCCTGGGGCACCTCTGAGAGTTGTTAAGGTCTCTACCATTCTTCCGCATGAAAGATTTCGATTGGTGAGTGGGGCAGCAAGAAACGATCTAGCATTGCTGCTCCTTCAAGAGGTCCAGACTCCCATTCAGCTTTTAGCACCCTTGGGTCATCTGAAGAACCTGAACAGCTCAGAATGCTGGCTCTCTGGGCCACGAATTCTTAAGCCAGGAGAGACAGATGAAAACCCAGAAATATTACAGATGCAGGTGATAGGTGCTTCAAGCTGTGCCCACCTTTACCCTGATATAGGCAGTTCTATTGTATGCTTCATTACTCAAGACAAAGACTCTGACACAAGTGTGGAACCAGTAAGTCCAGGCAGTGCTGTTATGTGCAGACCAAAGTCTAGGAATGGAAGCTGGAGACAGATAGGCCTCACTAGTCTGAAGGCACTGGCTACCATTGTGAGCCCCCACTTCTCATGGATATTATCCACTTCAGCAAAGGCAGGGCAGCCATTAAGCCATGCACTCATGCCTTGGATGGAAAAGCCTAAGTCCTCTAGTCTCAAAAAACAGCCAACCACCCTGCCATTTTCTTCAATAATAATTGTTTTGCTACAAAGACTT

>Goat, Capra hircus (BK059508)

CACAGATTGAAAGTCCAGATATAAACACCTGGAGAGAGACTGTGCCTTTCACAGCCTCACAATGGACAAAGGCTGAACCTCCAGCTATAAATTCCTGGAGAGAGAATATGCCTTTTTCAGTCCCACCATGGACACAGGATGGAAGTCCAGGTGTAATTACCTGGGGAGAGACTGTCACTTTCAGAGACCCACAAAGGACACAAATTGAAAGTCCAGATATAAACACCTGGAGAGAGACTGTGCCTTTCATAGCACTACCATGGACACAGGCTGAAGGTCCAGATGTAAATACCCAGAGAGATACTTTGCCTTTTACAGGTCTACGTTGGACTCAGGCTGAAAGTACAGCTGTGCATACTGGGAGAGATATTGTGCCTTTCACAGCCCCATCATGGACACAGGCTGAACCTCCAGCTGTAAATTCCTGGAGAGAGATTATGCCTTTTTCAGTCCCATCATGGACACAGGATGGAAATCCAGGTGTAATTACCTGGGGAGAAACTGTCCCTTTCAGAATCCCACCAAAGTCACAGATTGAAAGTCCAGATGTAAACACCTGGAGAGAGACTATGCCTTTCATAGCACTACCATGGACACAGGCTGAAGGTCCAGATGTAAATACCCAGAGAGATACTGTGCCTTTTGCAGGTCTACACTGGACTCAGGCTGAAAGTACAGCTGTGCATACTGGGAGAGATATTGTGCCTTTCACAGCCCCATCATGGACTCAAGATAAAGGCCCAGATGTAAATAACTGGAGTGAGATGCTGAGTTTCACAGGACCATCATGGGTACATGCTAAAGGTCCAGCTTTAAATACCTGGACAGAGACTGTCTGGAGGGAGACTGTACCTTTAACAGTCTCACCTTGGACTCAGACTGAAAATCCAACTGTAAATACCTGGAGAGAGAATATGCTTTTAACAGCCCTACCCTGGTCACAGGTTGAATATCCAGCTGTAAATACATGGAGAGAAACCGTACATTTCACAGAGCCACCATGGAATCAGGCTGAATATCCTGCTGTAAACACCTGGAGAGAAACTGTGCCTTTCTCAGCTCTACAATGGACTCAGGCTGAAAGTCCAGCTGTAAACACCTGGAGAGAGGCTATGCCTTTCACAGACCCATCATGGATTCAAGATAAAAGTCCAACTGTAAATAGCTGGAGACGGATTTTTACTTTCCCAGCCCAACCATGGCCACAGACTGAAAGTACAGTGGAAAACGACTTGATATGGAATGCCCCTTTTACAGCTCCACCGTGGTCACACACTGAAAGTCCAGCTGTAAATACCTGGACAGAGCCTATGCTTTTTATCGCCCCTCCATGGACTCAGGCTGAAAATCCATCTACAAATACCTGGAAAGTGAATATGCATTACAGAGACCCACTATGGCCTCAGTCTGACTTTGCACCAGCAAACCCTTGGACATCAACTGAAAGTTTTAGAATCACATCATGGACTCTTACAGGAAAGCAAGTTTTAAATATTTGGACAGAGCCAATAGCTTCCACAGCCACACTGTGGACTCAGGCTGAATATTCAACACCAAAATATTGGACAGAGACTAAGGCCATTTATATAGTCACATCATTGACCCAGTGTCAGTTTCCAATAAATACTTTGACAGAATCTGTAGGAGCCATAATCACACTTTGTACATCTGCTGAATCTCTATCATTAAGTTCTTTCACACAGAATATTATTGATACAATTGAATTTTGGCCAATGGTTAAAACTGAGTCTAAGAAAAGGTGGAATCTGCCTCAAACTAGTACATTCATGTTTTCACTAAATCCTCAAACTGATACTTTTGGATCCTTGAACCAAATTGAAAATCAAGAATCTCCTCTGTGGACCCATCCTGAAATTGATAATGTCAATACAATGGCGTTTCTTGAATTTGGAACACTCATATCACAGGTAGTACCTTTGCCCCAAGCAGCTAGATTCTGGCCCCAAACTGAAGCTGATACTAGCAAAATTTGGTTTGTCTCCTCTGAAAGAATAAATTCCTGGGACCAATCAGAGTCTCAAAGAATGAGTACCTCAATCCATTTTGGAGTGGGTAGAGTGAAGCCCCTCGCCCAACATGAAACTGCTACAGTCATGTCATGGCTTCAGATTGAAACTGGTATATTCCACCCTTGGAACAAGTCTGAAGGAGGCACAGGGAGGTTCTGGCCCCTTTCTGAAACTGAGGATGTAAGAGAATGGATCCAAACTGGAGCCGGTACAGTTAACTCTTGGACTCAACTGAGAACTAATATAGTCAGAGCTTGGCCCCAAGCTGAATCTGAACTAGTCAGACCCTGGACACAAACTAAAACGAATGCAATCACACTATTGACCCAGACTGATGCTATCAAACCTTGGTTCCAAACTAAAATTAATGCACTAAGAGAAGGGACCCAAACTCAATCTCAAATTGTTACTACTTGGATCCAAACACAGTTGCAAATATTTCACCCCTGGATTCAGCCTAAAAGTGATTCAGTCAGATTTTGGACCCAGCCTTGGATCCAAGCTGAAACCCACACAGTCAGACTCTATTATGAAATTACTATAAGAAAATCATGGGCCTCATCTGAATCTCAGTCAGTCACATTTTCATCACTGAGTCAAAATTCAGTTAAGAACTCATTTCACTTTGAATCTCAGATGACATGTTCCTGGGTCCGAAATGAATTTGATATAATCAGTCCTTGGAATCAATATGAAACTAGTTCTGTTGGATCCTGGTTCCAGTCTGAAACTGGTACCTGTCAACCCTGGCTCCATATTGAATCTTCTACAATCACACCATGGACCCAATATGAAACATTAGAGATCTCCCCTTCAACCAATCCTGAGACTGATACAGCAATAAGGAATTTGTTCCAGCCCCAAATTGATCTAATTAGTACTTGGAATCAGCCTGAAGTAGACACAATCAGATTCTGGACCCAAGTTGAAACAGAAACAATTCCAATGTGGACCCAGATTGGAACTCAAGTAGTTAAACCTCTCAACTTTTCTGAAGTTGGTATAGTTACACCTTGGCTAAAGACTGAAACTGATGCAAGTAGACCCTGGATTCAGTCTGACTTTCAGTCAATCCATCCGTGGAGCCAGATTGGATTTGGTATAATTGCCCCCTGGTCTCAGCCAGGAGCTTCTGTAAATCAACCCTGGACCTTTGTTCAAACACAGTCAATCAGACCTTGGATTACAGTGGAATCCAATAGAATCAAATATTGGTTTCATGTTCCAATGAAAAAAGTCAGACTGAGGATTCCTTCTGAGTCTCAAATATTGAGTTTCTGGATGCAGTCTGATGTTAGTAGAGTTAATGCTTGGATCCAACCAGAAACCCAGGCAGTCAATCCTGGGGCTCATCCTAAAACTGGCAATGTTGCATCCCTGACTATTCCTAACCCTGAAAGAGTCAGAATGTGGATCCAGCCTGAAACAGAAATAAGACTTGGCATCATTTATAAAACTAATATAGCCACATCATTTGCTTCTGAAATTGAACCAGATGGAACAATTAGTTATTTTGATTCGTGGTCTATCCATGTAACATTTTTACCAATAGAAACTGTTACTTCCCTAGATGAGCATTTTGCAGCTTTGTCAACTGAAATAGCTGCAGTAGAAAGCCAAGGTCAAATAAATTCTGTCCAACCCAGTGAGATCACAAATATTCTCTTTCTTACAATTGCAAGCACACAGCTTCCTGGAGGATTTGGTTACCTGAACTTTGGCAACAAATTACAAATTACCAATTCAAAAGGAAGCCCTAATGTCCCATATAGTTCTCTCAACCCACTTTTTCCGTCTTTTTCCTTTCCTGTTCCTTGTTTTTTCCCATTTTCATGTTCTTTGTCCCTTACTTGTTCAGTCTTTTCTTCTTGCACATTTTCTTCACCATGTACTTTTCCTTCTTGCTCAGTTCTTCCTATTGTGGGTTTCTCTCCTGTTCCTCCCTTAGCTGCTTCTGATAGTTCTCTCCAGAAACCATCTTCCTCAAAAGTTATTGAAGACACCATTCTTTCCCATACTTTTTCATCCTTTCATGCTGCTCCAGCCACTCTTTTAACAAAGCAACCATCTCTGATGCCTGGATTTCAATTGGGAACCAAGTCTAATCAGCCTGAACAAGATCTTCCTAAGTATTCTGAACTCAATATTTCCCTTGCTGAGTGTCGCCTGGGTGTGGTCTGGAAAGAGAGTCTCCAGGCTCTCTCGCTCTTCAAGACAGCTGTTATTTCTCATGAAATCACAGAGTGTGGATTGCGCCCTGGCCTTGTTCCACACTGTCCCAACTGCTGGGAGGCTGAAGTGGGTGAATTCCCTTGGATGGTTTCTGTGCAACTCTCTTTCTCCCATTTTTGTGCTGGTTCTATACTAAATGAACAATGGATTCTCACTACAGCTAGATGTGCAAATTTCATAAAAAACTCAGAAGCACTGGCCCATGTCCAGGTGGGGCTTATAGATCTTCAAGACCCTGCTCAAGCTCAAACTGTAGGCATTCATCGTGCCATGCCCTACCTGGGCCCTAGAGGACCTCTGGGACCTGGTCTAATCTTCTTGAAGCAACCATTACATTTTCAACCCCTGGTTCTTCCTATCTGCCTGGAGGAGAACCTAGAGCAAGAGAAAAATATACAACTGTATGACTGCTGGCTACCCAGTTGGTCCCTCATGAGAGGAAGTCCTGGAATTTTGCAAAAAAGGCACCTGAGCATCCTGCAAGCCATCACATGTGCCCAGTTTTGGCCCAAACTGAATGAATTTACTTTCTGTGTGGCAGCCAAGAAAGCTATGGGGGAGGCTGGCTGTAAGGGTGACCTGGGGGCACCTCTTGTGTGTCATCTGCAACAAAAAGACACATGGGTGCAGGTGGGAATTTTGACTCACTTTGATGAACACTGCACAAAGCCCTACGTCTTCAGCCAAGTGAGCCCTTTCCTTTTCTGGCTCCAGGGAGTTACACGACCTAGCCAAGCACCCTGGTCCAAGCAAGGGCCCATGACCACCTCTGCTTCCATCTCCCTTTCAGTCTCTACCTCTATGAATGCCTCAGCTTTTACTTCCACGCCTGCTTCTGTCCGGCCACATTTCATCTCTCTGCCACAGCCTCAGACTTTGGCAGATCGAATTTCTCTGAGATATGCCATGCCTTGGCAGGCCATGATCATCAGTTGTGGCAGTCAAATTTGCAGTGGTTCCATTGTTAGCAGCTCTTGGGTACTCACTGCAGCCCACTGTGTCAGGAATATGAATCCTGAAGACACAGCTGTAATATTGGGCCTGAGGCACCCTGGGGCACCTCTGCGAGTTGTTAAGATCTCTACCATTCTTCTGCATGAAAGATTTCGGTTGGTGAGTAGGGCAGCAAGAAACGATCTAGCATTGCTGCTCCTTCAAGAGGTCCAGACTCCTATTCAGATTTTAGCACCGCTAGGTCATCTGAAGAACCTGAACAGCTCAGAATGCTGGCTGTCTGGGCCACGAATTCTTAAGCCAGGAGAGACAGATGAAAATCCAGAAATATTACAGATGCAGGTGATAGGAGCATCAAGCTGTGCCCACCTTTACCCTGATATAGGTAGTTCTATTGTGTGCTTCATTACACAAGACAAAGATGCTGACACAAATGTGGAACCGGTGAGTCCAGGCAGTGCTGTCATGTGCAGACCAATGTCTAGGAATGGAAGCTGGAGACAGATAGGCCTCACTAGTCTGAAGGCACTGGCTACCATTGTGAGCCCCCACTTCTCATGGATATTATCCACTTCATCAAAGGCAGGACATCCATTAAGCCATGCACTCATGCCTTGGATGGAAAAGCCTAAGTCCTCTAGTATCATGAAACAGCCAACCACTCTGCCATTTTATTCAACAATAATTGTTATACTACAAAGGCTTTAATAACTCATTGCAAAAATAATGCAGGGCTAATCTATTCAAACTATTCATAATAAAAATGTTAACGTTAAAAAAAATAAGGCCCTAT

>Guinea Pig, Cavia porcellus (BK059522)

TCACAAGGTTCAACAGTGATATCCTGGCCCAAGGCTGAATCTCCAATAGAGGGTCTTGGGAAAGAAGCTTTAGCTTCAGAAGTAAAGCTGTGGAACCCCATTGAATTGCCAGCAGTCAACTGGATAGATCCTATAACACAGCTTATATATCACACAGGAAACACCTGGACAAAGTCTTCATCTAACCCTACTATATTACAGAATTGTTCTGAATCTTCAGCAATCATTTCCCGGATGTACACTGTGTCTGACACTTTTACAGCATTTACTAAAAATGAATTGACAATTTTATCTGATGAAATCACACTGTGGGCCCAAGCCATGTTTGCTTCAGACAAGCCTTGGAAGAAAGCCCAATATGGCATAGGAATGCCTGTGACTCAGGCTGCATCTATATCTAAAGTTTCCTCTATGCAACCTGTGTCTGATGCAGACATACCACAGAGCCCAACTGAGTTTCTGGCCAAAGTTGGGAGGATACACTCTATATCTGATACCTTGATACAATGGATGCATTTTGGATCTCCAGGAAGCAAGATTTGGACTGAAATTACAGCTTCCACAGTTACACAGTGGAGACAAGCTAAATTTCCTATTTTCAGGTTTTGGGAACCAACTGTTACTCCTACAACCACCATGTGGAAACCAAGTGCATCTCCAGCCATTAACTTTGGTTCAAGGGCTTCAGCTGCCACAGTCTCCCCTTTGACACACTATGCGTCTCCAGAAATCCAGCCTTTGTCAGGACATTTAGCTTCCACTCCTAGGAACTGGAAACAGACTGACTTTTTGCGTATGAATGTTTACACACAAACTGAAACTGATGTAGCCATATTTTCTGAAATGTCTGTAAAACCTTTGATTCCACTTGGTTCTAAAGTTAATGTATTCATTCTGTCTGAACAAACTCAAACTTACACAACCCAAGGCTTGAATGAGATGGAAAATCAAGATTATATTCTGAGGGCACATAAATTTGAAGATGCTAATGCAGGGACTTTGTCTAAAGTTGGAGCACTTGTATCTGAGGCTTTGCCTGCAAGTCAATCAGCTAGAGTCTGGCCTGAAATTGACCCTCATAATAGCAGAGGTTGGTTTGAAATTGAAACAGAAAATATCCAACCATGGGCTCAAACAGATTTTCAAAACTATATCTCATTTAGGACCAGACAAATAGAACTAGGGGTCCAACATACGCTTGCTTCATTCACCTCATTGACCCAATCAGAAGTTGGTATGTTTGATCTGTGGACATCTAAAGTAGACATAACAAGGTCCCAGACCACTGCAGGGATTGAGAATTTACTGAGACCACTGATCCAAACTGAATCTGAGTCAATCAATGCCTGGATCCAGCCAACGGATAATATAATCCGACCACATACCCAAACTGATTATGAAGCAACCAGGCTCTGTACTCAGGACACATCTGATAAAGTCACAATGTCATCCCAGGTTGAAATTCAGGAACCAAAGCGCTTGATCCTACCTGACAGTTATAATGTCAGAGTTTTGTTTCAGACTCAAAAGAATACAATACAAGAAGGCCAACTTGAAGAACAAATGGTTACTCTCTTCATGGAGGCAGAAGGGCAATCTCTTGAAAGTAATATGATTGAAACTCCTTCCCAAGATAAGTGTGATATAACCCAAGTTGAGGACTACTCTGGAACAAATTCAATCAAAGGTTTGACCTCTTCTGAAAGTGATGAAATACTACTTTTGACCCAGACAGAAGGCCAAGTGATGAAAACCTGGGCAGAGGATGATTTGGTCAGAAATTTAGCCTTCACTGAACATACGACAAATAGTCATTGGTCCCTCACTGAATCTCAAGTGACACATTCGTTGGCCGGGACTAAAGTTGTTCTAACCGATCCTTGGCTTCAGTACAAAGCTGGGATGATCAGCCATTTGGCCCAGTCTGAAGCATCAGAAAGTATTCACTTGACCCAGCCTGGAGCCGAAAGAGAATACTGGCTCCAGTCTCAAATAGATTCAGTCGGATCCTGGAACCAGCCTGAAAGTAAACCAATTCTAAGCTGGACCCCAACTGGAAAAGAAATAATAAAAGGGTCGAACTGGGAGGAGGTTGAGGTTGAGGGAGTGACACCTTGGCTTCAGACTCAAGACAGTTCTACAGTTCGGATTCAGCCTGACTCCAAAACAGTGAGTCCTGAAATGCAGAGTAAACTTGGTGTAACCCAGACACAGGAAAGTGATGCTGCAAATCATCCTGATACTCAAGCAATCAAACCCTGGCAGAAACATGAATTTAGTAAGGCCAGCCCTTGGTTCCACATTCAAATGAGTACAGTACTGCAAGGCTCTCCAGCCACAGTAACATTAAAAGGACCCATGACTGGATCTCAGTTTGAACCCGAATCTCAGTATCAGCCTGGACAACAGACTCCCAACCATTCAGAACTCAAGATTTCTCTGGCTCAGTGTCACCTAGGTTTACTGTGGCAAGAGAATCTTCAGGCTCTCTGGCTTGTCAAAACACCTGTTATCACTCATGCTGCCACAGAGTGTGGATTACGCCCAGGCTTTGTTCCTCGCTGTCCCAACTGCTGGAAGGCAGAAGTGGGTGAATTTCCCTGGATGGTTTCAATACAATTGTCTTTCTCCCATTTCTGTGCTGGCTCTATTCTGAATGAACACTGGATCCTTACCTCCGCTAGATGTGCCAATTTTGTAAAAAGTTCAGAGACACTGTCCATGGTGCAAGTGGGGCTTATTGACCTCCAGGGACCAGCTCAAACTCATATTGTGGGCATTCACCGTGCCATGCCCTATATAGGTCCCTCAGGGCCCCTAGGACCCGGCCTGATCTTTCTGAAGGAGCCACTACATCTCCAACCCCTCGTGCTTCCTATTTGTCTGGAGGATAACATGGAGCAAGAGAAAAATAAGCCACTATATGATTGCTGGCTATCTAGCTGGTCCCTTCTGAGGGGAAGACCTGGAATTCTGCAAAAAAGACACCTAAGTATTCTGCAAACCAGCACTTGTGAAGAATATTGGCCCACAGCAAGTGAATTTACTTTCTGTGTGGAAGCCAAGAAAGCTACTGGGGAAGATGATTGTAAGGGTGACGTAGGGGCACCTCTAGTGTGCCGTCTACCACGAAAGAACACTTGGGTGCAGGTGGGAATATTGAGTCACTTTGATGAACATTGCATAAAGCCCTATGTCTTCATCCAAGTGAGCCCTTTCATTTTCTGGCTTCGTGGAGTAACACGGCCCAGCCTTGCACCCTGGTCTCAGCAAGCAGCCATGACGCCCTCTGTTTCTGCCTCCCCTTCAGTCACTACTTCTATAAATGCCTCAGTTTTTACTGGCAATTCAGCTTCTGTTCAATCACACTTCATCCCTCTACTACAGCCTCAGAGAAGATCACAAGACAATGGCAGTAAAGCTTTGACAGATCGCATTTCTCTTCATTATGCGATGCCTTGGCAGGCAATTATAATAAGCTGTAGCAGTCAAATCTGCAGTGGTTCCATTATTAGCAGCTCTTGGATTCTCACTGCTGCCCACTGTGTCAGAAACATGAACCCTGAAGACACTGCTGTAATACTTGGCTTTAAGCATCCTGGGGAATCTCTGAAAGTTGCTAAGGTGTCTAATATTCTACTGCATGAGAGATTCCGGTTGGTGAGTGAGGCAGCAAAAAATGACCTGGCATTGTTGCTCCTGCAAAATCCCCAGACATCCATTCAAACATTAGCACCTTTGGGTCACATGAAGAATCTAAACAACTCAGAATGTTGGCTGTCTGGACCACAAATTCTTAAGCCAGGACAGACAGATGGAAATCCAGAAAAATTACAGATTCAGGTGATGACAGCATCAACTTGTGCCTATCTATACCCGGACATAGATGGTTCCATTGTTTGCTACATTTCTAAGGCCAAAGGTGCTGATGCAAATACGGAGTTAGTAAGTCCAGGAAGTGCTGTCATGTGTAGAGAGAAGTCTGCAAATGGCAAATGGATGCATGTAGGCTTCACCAGTGTCAAGGCTCTAGCTACAATTGTGAGTCCCCATTTCTCCTGGATTTTGTCTAGTTTGGCAAAAGCTGGCCCATCACTAAACCAGGTCATAATGCCTTGGGTTGAAAATCCCAAGTCTTCTAGTCTCCTCAAAGAGTCAAGTGCATTGCTGCTTCTTTCAATACTGATTATTGAAGCCCATATAATTCTGTAGTCCAGTAGCTACAGTTGGAAGTGCTAGTCTACTTGTCCTGAAAGATAAGAGAATGAAAATCATCACAGAACAAAGCACAACTTATGCTCTATGTTATATATTTCCTATGTTTTGTAGTCT

>Hamster, Mesocricetus auratus (OK484382)

CTGAAACTCTAGAAATCATCCCTTGGACAGATTCTCACAGATTAATATCTTATTCTTTACAAACTCAAATAGATTCAATTACATTATGGAAGCAGCCTCAACCTATGATGACAGCCCAAGTTTGGAGTACAACTCTAGAACAAGTAGTAAAAACACAAGCCCCAACTGCAGTTGGTACAGTTTCACCATTGTTTCAGGTTCAAGAAATGTTTTTAAATGAAGAACCTTCCTTTATGTCACCATATCAATCTGGACTTCAAGATAAGCATTCACCGGAACCATTATCTCCTAGGCAATCAAAACTCAATGTTTCTTTGGCTGAATGTCACCTGAGTATGATGTGGAAAGATAATATTCAAGCTCTATGGCTCTACAAGACAGCTGTTGTTTCTCATGAGATCACAGAATGTGGTTCACGCCCTGGCCTGGTTCCCCACTGTCCTAATTGCTGGGAGGCTGAAATAGGTGAATTCCCCTGGATGGTTTCTGTGCAACTCTCTTATTCCCACTTCTGTGCTGGCTCTATACTTAATGAAAATTGGGTTCTTACCTCTGCTCGATGTGCCAATTTTATAAAACGATCAGAATCTATGGCTTTAGTCCAAGTTGGACTGGTTGATCTTCAAGACCCTAGCCAAGGTGAAACTGTAGGTATACATCGTTCCATGCCATACTTAGGTCCGAGTGGACCTTTAGGACCAGGCCTAATATTGCTGAATGAGCCACTGCATTTTCAACCTTTGGTGCTTCCAATTTGTCTGGAAGAAAGTCAAGAACAAGAAAGGCACATACAATTGTATGACTGCTGGTTACCCAGCTGGTCCCTCATGAGGGGAAGTCCTGGTATCCTGCAAAAAAGGCACCTCAGCATCATGCAAGTCAGTACATGTGCCAAATTTTGGCCTCAGCTGAATGAGTTCACTTTCTGTGTAGAGGCCAAGAAAGCTATGGGGGAATCTGGCTGTAAGGGTGATCTTGGGGCACCTTTAGTGTGTCGTCTAAAACAAAAAGACAAATGGGTGCAGGTTGGAATTTTAATTCACTTCGATGAACATTGCACAAAGCCCTATGTCTTCAGCCAAGTTAGCCCTTTTGTTTTCTGGATCCAGAGAGTTACACAACCCATCCATGCACCCTGGATTCATCAAAGGCTTGTGACTACCTCACCTTTTAATTCCCTGTCAGTCTCTACCAATAAAGCCTCAACTTTTACTTCTCCAAATGCTGTAATTCATCGACACTTCATCTCCCTGCCACACCCTCAGGCATTTGCAGATCATATTGCTCTGCAATATACTATGCCTTGGCAAGCTATGATCTTCAGTTGTGACAATCAGATCTGCAGTGGCTCCATTATTAGTAGCTACTGGGTTCTTACTGCTGCTCATTGTGTCAGAAACATGAATCCTCAAGACACTGTTGTGATACTAGGCCTTAGACATCCTGGAACACCTCTGAGAGTTGTTAAGGTGACTAGTATTTTACTGCATGAAAGATTCCGGTTAGTGACTCAGGCTGCAAGAAATGATTTAGCTCTTGTTCTTCTTAAAGAAAGTCAGAGTGCTTTTCACATAGTGGCACCTTTAGGCAACATGAAGCATCTAAACACTTCAGAATGCTGGCTTTCTGGACCACAGATTCTTAAACAAGGTGAAATACTTGAAAATCCAGAAATGTTACAGATACAAGTCATGGGAGCTTCAAACTGTGCCTATCTCTATCCTGACATAGGAAGTTCTACTGTTTGCTACATTGCACAGGCCAGGGGTCCTGAAATAAATATGGAGTCAGTGAGTCCTGGAAGTGCTGTTATGTGCAGACCACTATCTGGAAATGGCAAATGGAGACAAATAGGGTTCACTAGCCTCAAGCATCTAGCTACCATAGCTAGCCCACACTTTTCTTGGATTTTGTCTACAGCAGCAAAGGCAGGTTATCCCTTAAATCAGGCCTTCAGTGCTTGGGTAGAAAATCCCAGGTCCTCTAGCCTTGTTACACAACCAAGCTCATTGCTATTTTCTATGGTAATGGCTATTGCAATGAGGAGGATATTTATTTTATAGTGTACTTATTACACCAAGGTATACAAATATGCTAAACTAAGAAATCAGAAAAATTAGGAAAATATGCTGACATGTATTATAGCACCTCCCTACATTTTGTTTTTAATAAAGAGTATGTCCCTAAACAATGATAC

>Horse, Equus caballus

GTCACAACGTGGGCAAACGCTGAATTTCCAGCACTAAAGCCCTTGACACATCTTTTGTCTGATACTGTCAGGCTGTGGACTCAGGCTGAATCTCGAGTAGTAAATCCCTGGGTGCGGCCTGAAAATAATAGAGTCATACCGTGGGCCCAGGCTGAATCCCTAGCAGGAAGTCCTTGGATACAGTCTGTAGCTGATACAGCCACACAGTGGACTCAGGGTGAGCCTTCAGAGATAATTCTCTGGGCAAAAATGGTGGCTGATACAATCACACCAAGGACGCAGTCTGAGTCCCTAGCAGTAAATCCCTGGACAGAGTCTGAAACTGATACAGTCACACTGTGGACCCAGGCTGAACCTCCAGCAGTAAATCCCTGGACAGAGGATGTAGTTTCCATAGTCACGCCACAGACCCAGGCTGATTCTCTAGCAGTAAATCCTTGGACAGAGCCTGTATCTGATAGTGTTATACAGCGGACCCAAACTGAACCTCTGGCAGTAAGTCAGTGGACACGGATTATGTCAGAATCAGTCACAGCAGTTTCCCAGCCTGAATTTCCCACAGTACGGACCTGGACAGATCCTTTGGCTGACATAATCCCACTGTGGACTCAGGCTGAATGTCCAGTAGTAAAGCCCTGGACAGAGGCTGTGACTAATATAGTAACACAATTGAGCCAGGCTGAATCTCCTTTAGTAAATCCCTGGGCAATACTTGAAACTGATACTTTCACACACTGGACTCAGGCAGATTCTGAAATCATAAATCTCCTGAAAAAGGCCATAGTTGATACAGTTAAACCATGGACACTGGGTAGATATCCAGCGTTAAATCCCTGGACACACTTTGAAACTGACACATTCACAGCACAGACTCAGGCCGAATCTCCAGCAGTGTATCTCTGGGCACAGTCTGAAAGAGACACAGTTACACCATTGACCCAGGGTGAATCTCCTGCAACAAATCCCTTAGAAATACCTGTAGATGGTACAGTCACACCATGGACCCAGGCTGAATCTCCTACAGTAAATCCCTGGATAGAGCCTGAAAGTAACACAGTCACAGTGTGGACCCAGGGTGAAACTCCACCAGTGATCTTTTGATAGAGTCTGGGGCTCCCACAGCCACACTCTGGAACCAGGGTGAATCTCCAACAGTAAATCCATGGACACAATGTACAGCTGATGTAGAAATACCGTGGACGCACGCTGAATCTCTAGCGTTAAATCCCTGGACAAAGTCGGTAGCTGACACAGCCCCACAGTGGGCCCTGGTTGAATCTCCAAGAGGAAATGCTGGGACACAGCCGTTAGCTTCCACAGTCACATTTTGGGCCCATGCTGAAACTCCAACAGTGTTTCCTTGGATACAGACTGATTTTGAAATAGTCCTACCAACAGCCCAGGTTCAATCTGCAGCAATTAAACCCTGGATGCAGTCTGAAAATGACACAGCCATACTGTGGACCCAAGGTCAATCTTCTGCAGTGAGTCCCTGGACAGAGACTGTGGCTTCCACAGTCACACCCTGGACTCAGGCTGAGGGCCCAGCAGTGGAGACCTGGACAGAGACTGTAGATGCCACAGTCGCACCCTGGACTCAGGGTAAATCTCCTGCATTAAAAACCTGGACAGAGACTGTAGTTCCCACAGTCACGTCATGGACTCAGGCTAAATCTCTAGCAGTAACGTCTTGCTCACAGAGTGTAATTGATACAGTCATATTTTGGACAATGCTTAAAACTGAATCTAAGAAACCCTGGACACTGCCTGAAACTAGCATATTCAGTATTTTATTACGTCCTCAAAGTGATACTACTCAATCCTTGATTCAAGTTGAAAATCAAGCATCTCTTCTGTGGACACATCCCAAAATTGATAATGTCGATGCAGGGACCTTGCCTGAATCTAGAACCCTCATATCATGGATAGTCGCTGTGCCTCAAGCAACCAGACTCTGGCACCACCCTGAAGGTGATATTAGCAGATCTTTGTTTGAAACTCAAACAGAAAGAATGAGACCCCTGGCCCAGTCAGAATCTCAAAGAGTGAGTACGTTGACCCAGTTTGGAGCTGGTATTGCCGAGCCCTTTGCTCAACATGAAACTGCTACAGTCACATCATGGATTCAGACTACAACTGGTATATTCCACCCATGGAACCAGTCTGAAGGAGACACAGTGAGATCCTGCACCCTTTCTAAAGCCGATGGGTTCAGACCGTGGATCCAAACAAAAGCTGGTATAGTCAACCCCTGGTCTCAAGCTAAAAGCAGTACAGTCAGACCCCGGACCCAGGCTGAATCTGAAGCAGTCAGCCTGTGGACACAGTCTGAAGCAACGAAACGCTTAACTATGCCTGGCATTAATACCGTCAGACCTTGGATCCAGACTCACGTTGGTGCAATAAGACATAGAATTCAACCTGAATCTCAAATAATTACTACCTGGATCCAGACAGAATGGCAAGTAGTTCATCTCTGGATCCATCCTGAAACTAATGCAGTCAGAGTGACTTAGCCCAACCCTGGATCCAAGCTGAAACCAACACAGTCAGACCCTGGATACATTACGAAATCGACACAGTAAAACCGTGGACCCAGCATGAATCTCAGGCAGTCAATACCTGGACTGAAGCGGATACAGTCACATTTTGGTCCCCGACTCAAAATCATGCAATTAGTCCCTGTTCCCAACTTGAATCTCAAATGATATGTTGGTGTAATCAACCCTCACACTAGATTTATAACTAGTTCAATCAGATCCTGGACCAAGCTTGAAATGGGTACAGTCCAACCCTGGATCCACCTTGAATCTGCTACAATCAGACCATGGTTCCAATCTGAAACTTTGTTAATCTACCCCTCAATCCAATCTGAGGCTGATACAGTAATAAGACGTTGGTTCCAGACTCCACTAGATTCAGCTGAACCTTGGAGCCAGCCTGAAGCTAGTACAGGTAGATCCTGGGCCCAACCTGAAATTGAAACAATGCAAATTTGGACCCAGACTGAAAGACATGTACTAAAACCTCCAGTCTTACCTGAAGTTGATACAGCTACAGCTTGGTTACAGGCTAAAAGTGATATGATTGGACCCTGGATTTGATCTAACTCTCAGGCAATCAGTCCTTGGACCCAGACTGAAGTTGGTATAGTTCACCCCTGGACTCAGCAGAGGCCTACAAATCAACCCTGGACCTACCCTGAGACCCAAGCATGTAGACCTTGGAACAAGCTGGAAGCTGATACAGTCAGACCTTGGTTCCACTTTCAAATAAGTAAAGTTAGACCGTGGACCAGAGTTTTTCATCCATTCATGCTGCTCCAGCTGCACTTTTAACAAAACTATCTTCTCTGATGCCTGGATCTCAATCTGGAACCAAGTCTAATCAGCCTGAGCAAGAGGCTGTCAAGCATTCAGAGCTCAGTGTTTCCTTAGCTGAGTGCCACCTGCGTGTCATCTGGAAAGACAGTCTCCAGGCTTTCTGGCTCTTCAAGACGGCTGTTATTTCTCATGAAACCACAGGCGAGTTCAGCATAGTCATCAATTTGAGTTATTGTCCTTGAGACCAAGTATATTACTATAAAAACACTTTCAGTGCTTGAAAATGGTTTATTATTTCACTTTAAAAATGAATAGAGGTAGTGCAGATTATTCTCAGAGAGAGTGTACAGACAACCTCTGTTATTACATGTAACTTTGTAGGATTAAAAACATATTCCAAGTCTTTCCCACAGTTGAGGATTTACAAGCATTTTCCTAAAATCTGAAAATAGGAAATCAGCATCTTCAGAGAAAGGAGAGTAGTGACCCTAACCTCACCCTGTTGCCTATGTGCATTCCTTCTGCAGAGTGTGCATTATGCCCTGGCCTTGTCCCGCACTGTCCCAGTTGCTGGGAGGCAGAATGTCCTGAGTTCAAGTCATAACCCACATTAGAATCACAGTTTTACATTTTATGAACTTAAAGCATCTTGTTTCTTAAAACTTAATTTCTCAATCTCTAGAAGAATAATAACTATCTCAAAATATTTGTTTGGGTATATAATTTTATTTCTCATTTTAACATTGTGAAGTAAAGCTATTAGTACAGACAAGACAAACAAGGGAGTAACTGTCTAAAGTCTCACACCTTGTAAGTGGGATATTTGAACTCAAATTTCAAGGTCTATATCACTCAAGTGTTAAACATTATAGATATTAGGATGTATGAGAATTTGTTTCAACTTAAGGATTTTCAACAAGGATAAGGATTGCGTCAGAGCATTTATCTTCTCATCCTGAGGGTGTCCACATATGTTGTGCGTTATGTGTTGGCAACAAGCAGAGAACGGAATATGCGTAGTACCTGAGTGCTCCCCTGGACATCATCTGATTGATGTGACCTCCAAAGTTCTTATTCACATTCAAAACTGTAATAATGCTTGCTAAATTTTCCCCCCAGAAAAAACTCAGAAGCCCTTGCCCTGGTCCAAGTGGGGCTTATTGATCTTCAGGACCCTGGCCAAGCTCAAACTGTAGGCATTCACCGTGCCTTGCCCTACCTAGGTCCCAGGGGACCTTTGGGACCTGGCCTAATCTTCCTGAAGCAACCACTACATTTTCAACCCCTGGAGCTTCCAATCTGCCTGGAGGAAAGCCTGGATCAAGAGGAAACCATACAACTGTATGACTGCTGGCTACCCAGTTGGTCCCTCATGAGGGGAAGTCCTGGAATCCTGCAAAAAAGGCACCTAAGCATCCTACAAGTCAGCACTTGCACCCAATTTTGGCCCAAACTGAATGAATTCACTTTCTGTGTAGAAGCCAAGAAAGCTATGGAGGCTTGTTATAAGGGTGACCTGGGGGCACCTCTGGTGTGCCATCTACAGCAAAAGGACACAGGGGTGCAGATGGGAATCTTGAGTCGCTTTGATGAACATTGCACAAAGCCCTACATCTTCAGCCAAGTCAGCCCTTTCCTTTTCTGGCTCCAGGCAGTTACACAGCCCAGCCATGCACCCTGGTCCCAGCAAGGGCCCATGACTACCTCTGCTTCCATCTCCCTTTCAGTCTCTGCCTCTACGAATGCCTCGGCTTTTACCTCCACTCCCACTTTTCTTCGGCCTCCCTTCATCTCTCTGCCACAGCCTCAGAGTAAGGCCCAAGAAAATGGTAGGGAGGGAGGTATAAGAAAGAAAGGGAGAGAAGAGGATGATGGAAAGCCATATTCCCACATATTACATTTCCACCTTACCAAAGCTACTTACCGTGTCTCATATTGCCATGTCAATAGCGAAGGATGCTTTTAAAATGTAAGTGAGGTCATCTCACTTCTTTGCACAAATCCTTCAGTCTTTGTCACTCAGAGAACTCAAAGGTGACTTCAGCAAACAGGATTCTAGTATCTATCTTCATCTATCCTCTTGCCATACTCCTTATCAGGGTCTTATCTTCAACAATTGCTCTTTCAATATGAGCTTTGTCTTTTGGGCTTTTTATGTTCCATCTGTTTTTCTAGAAGAGACAGATGAGACTCCAGAAATGTTACAGATGCAGGTAATGGGAGCTTCAAACTGTGCCCACTTCTACCCTGACATAGGCAGTTCTATTGTCTGCTTCATTACTCAGGCCAAAGACTCTGACACAAATGTGGATCCAGTGAGTCCGGGCAGTGCTGTTATGCGCAGACCAGTGTCTGGCAATGGCAGCTGGAGACAAACAGGCCTCACCAGTCTGAAGGCACTAGCTACCATTGTGAGACCACACTTCTCTTGGATAATGTCCACTTCAGCAAGGGCAGGCCATCCCCTAAACCAGGCCCTCATGCCTTGGATGGAAAACCCCAAGTCGTCTAGTCTCCTTAGACAGCCAACTACACTGCCACCTTCTGCACTAGTGCTTATTGCACTAGGCAGTCGTTTGTAACCCAGGAGCTATGAAAGGGAGCAATAGTCAATTCACAGTGTGATAGAAAAGAAAT

>Koala, Phascolarctos cinereus (BK059523)

GCGGAACATCCAGGGTAAGGTGAAGGGAGGGCTCTGGAGGGTCTCATCATTGGCCTAGAGCCCGAGGAGGAGCCTCATTTCTGCTTCTCTGGAGGGCCACTCATCCCTGCCCTTCTTTGCCCTGCCATGTCCTTCCCAGTGATGGAAGCCAAGATAGACGCTATTTGTGGGTCTTCCCCGCCGCATGTACATTGCACATGTACACACCCGCACACGTGCAGACAGACTTTCCATCTGCCTGCAGATGGAATTCAGAAAGCCTGGCCCGGGTCCAGGCAGGGGTCATCAACCTAGAAGACCAGGTTCGGGCCCAGCTTGTGGGCATCCACCAGGCCCTGCCATACCCTTCACTAGGAGTACACATGGGCCTGGGCCTGGTCCTCCTCCAGGAGCCCTTGCGCTTCCAGCCCAGAGCCCTGGCTGTGTGCCTTGAGGAGTTCCCAGAGAAGCAGTTACCCGAGCCTCAGCTGCATCTCTTTGACTGCTGGGTCCCTGGCTGGACCTTGATCAAGGGGAACCTGGTCACGATGCAGAAGTGGCGACTGAATGTGGTTGAGCTCAGCAACTGTGCCCAGTATTGGCCCATCAAGAACTCGATGGCCTTCTGTGTGGAGGCCAAGAAGGTGATGGGCCAGAGCAGCTGCAAGGGAGACCTGGGATCCCCATTGATGTGCCGTCCAAAGCTGCACCCGGAGGAGAGCCCCTGGGTACAGATGGGTGTCCTCACCGCTTTTGATGAGGACTGTGTTCGGCCTTATGTCTTCAGTCGCATCGGCCCCTTCGGCCTTTGGCTCAAGGCCTCCATGAGGCCCCAGCACCCCCCCTGGGCCAGGCCAATCCACAGGCCCACCCTCTCTTCCCTGCCCAAGCCCGAAGCTCTGATTGTGACCTGTGACAGCACCGTGTGCAGCGGATCGATCCTCAGCCCTTCCTGGGTCCTCACCTCAGCCCACTGCGTCCAGGACATGAGATCAGAGAACATGGCATTGTTTCTGGGGCTGCCACAGCCTGGGGGCAACATGACAGCTGCACGGGTGTCCAGTGTCATCCTGCATGAGCAGTACCAGGCAATGAATGGGGTCCCCTGGAATGATTTGGCTCTCATCCTCCTGCAGAAGCCCTTGGGCCCTGACCAGCCCCTGGCCCCCATGGGCCATGTGGAGGATGTGCACAAAGCTGAGTGCTGTCTTACAGGGGCCCGTGAACTCCAAGAGGGTGAGAGGGACCAGTACCCACGAGCCCTCCAAGTTCAGGTGAAAGATGCTTTGACCTGTGCGCACCTCTTCCCTGGCATCAAGAGCGCGGTGCTCTGCCTGGGTCCCCGGTTGCCAGAATTCCAAATGGCTTTGGATCTCATGGGCCCGGGCAGTGCCCTGTTGTGCCGATCAAGGGGCATGAATGGGACCTGGAGGCAGACTGGACTCATCAGCATCAAGTCTCTTGCCTCTCTCTTGGCCCCTTGCTTCCCATGGATCTCCAACACCTCTGCTGTCCAATCTGACCACATTTTGTTCAATCAGTCAATGAAGAGGCTTGTCCCTGTCATATCTGTGGCAGGGGCCAGGAGCCCTCTTACTCTCTTCATACTGTTGACCCTTTGGCTAGGGTCCCTCATAGCATAGGGCTCCTGCACCCATCC

>Minke Whale, Balaenoptera acutorostrata scammoni

CGATCACACCATGCATCCAGGTTGAATCTCCAGCAGTGGATCCCTGGACAAAATCTGCATATGACACAGTCACATTGTTTACCCAGAGTATAACTCCAGCAGTAATTCCCTTAACGATAGCTGTAGCTGATAGAGTCACACTGTGGAATATGGCCAAAATTATATTACTAAAGCCTTGGCCACAGTCTGTGACAGATACACAATGGGTGCAATATGAGTCTCCCCCAGTATATCCCTTTGCACAACCTATATCTGACAAAGTCACACTATTTACTCAGGCTGTTACTCCAGAAGTAAATCCTTGGTCAAGGCCTGAAGCTGATACAGTCACACCATGGATCCAGGTTGAATCTTCAGAAGAAAATGCCTGGACAGAGGCTGGTTCTGAAACATTCACACCATTGATCATGGGATTTAAACCCTGGATAGAGACTGTATCTGATATAGTCACACCAGGAACCAAGTATGATTTTTCAGAAGCAAAACTTGGGATACTGCCTGAGGCAGGTATATTTGAACTGTGGACTCAGTCTGAATCTTCAGCNNNNNNNNNNNNNNCAGGAATTCCCTGGACAGAGGCTGTACCTGTCGTAGTCACACAGTGGACCCAGGCTGATTCTCTAGCAGTAAATCCCTGGACACAACCTGCAGCTGATACAGTCACAAAGTAGACACAGACTAAATCTCCATTAGTAAATATGTGGACTCAGGCTCAATTTCCAGGGGTAAATCCCTGGACACAGTCTGAATCACTACCAGTAAATATTTTGACAGAAACCGAATCTCTAGCTCTAAATCCACCAATACAGCCTGAGGCTAGTACAGTCACATTGTGGATTCAGGGTGAATCGCCATCAGTAAATCTTTGGATAGAAGCTGTAGCTTCCAGAGTCATACCATGGACCCAGGCTGAATATTCAGCAGTAAACCCATTGACATAGGCTGTAGCTAATGCACTCACACTGTGGACCCAAGCTGAATCTCTAGCATTGCATCCCTGGCCAAAGTCTATGATGGATACAATCACAGAATGGGCCCTGGATGCATTTGCTCCAGGTGCAAATCCTCTGACACAATCCTTAGCTTCCGCAGTCCCATTTGGGGCCCAGCCTGAAACTCCAACAGTAAATCCTTGGATACAGTTTGATTTTGAAACACCCCCACCATCAACCCAGGTTCAATCTGCAGCAGTTAAACCATGGACACAGTCTGAAAATTACACACTCATACCATGGACCCAGGTTCAACCTCGTGCAGTGAATCCCTGGGCAGAGGCTGTAGCTTCTTCCGTCATACCATGGACTCAGGCTCAGTCTCCAGTAAATCCTTTGACAGAGGCTGTGCCTTTCACAGTCATACCATGGACTCATCCTGAATATTGTGCAGCAAAATCCTGGACAGAGACTGTAGCTTCCACAGTCACACTGTGGATTCAGGCTGAATTTCCAACAACAAAGACATGGACAGAGGCTATAGCTTCCACAGTCACACCATGGATTCAGGCTGAATCTCCAACAGTAAACACTTGGAGAGAGGCTGTAGCTTCCACAGTCACACCATGGACTCAGGGTGAAAGTCCAGCAGTAAATACTTGGACAGAGGGTGTAGCTTCCACAGTGGCATTGTGGACTCATGCCAAATCTCTAGCATTAAATTCTTTCACACAGAGTGTAATTGATACAGTCGTATTTTGGACAACGCTTAAAACAAAGAAACCCTGGAAACTGCCTCAAACGACGATGTTCATTATTTCACTGAATCCTCAAATTGATACTATTGGATCCTTGAACCAAGTTGAAAATCAAGGATCTCTTCTGTGGTCACATCCTGAAATTGATAATGTCAGTACATTAACCTTGCCTGAATCTGGAGCACTTATGTCATGGATAGTGCCTTTTCCCCAAACAGCCAGACTCTGGCCCCAACCTGAAGCTGATATTAGCAGACCTTGGTTTAAAACCTCAGAAAGAATAAGATCCTGGGCTCAGTCAGAATCTCAAACAATGAGTACCTCAACCCAGTTTGGAGCTGGTAGAGTCAAACCCTTGGCCCAGCACGAAACTGCTACAGTCGTGTCATGGATTCAGACTGAAACTGGTATATTCCACCCATGGAACCAATCTGAAGGAGACACAGTAAGATCCTGGAACCTTTCTAAAACAGGCTGTAAGACCATGGATCCAGACTGGAGCTGGTATAGTCAACCTCTGGGATCAGCCTAGAAGTAGTACAATCAGACCCTGAACCCAAGCTGAATCTGAAGCAGTCAGACCCTGGACACAGTCTGAAGCTAAGGCAATCACAGTGTTGACCCAGGCTGAAACACAAGCAATGAAACACTTGACATTAATACTGTCAGACCTTGGTTCCAAACTCAAAATGATGCAATAAGAGAGGGGACCCAACCTGAATCTCAAATAGTTACTACCTGGATCCAACCACAATTGCCAATAGTCCATCCCTGGATTCAGCCTGAAACTAATGCAGTCAGATTTTGGACCCATGCTGAGGGTTATTTAGCCCAACCCTGGATCCAGGCTGAAACCCACACAGTCAGACTCCAGACTCATTATGAAATTGATCCAAGAAAACCATGGACCCAGCCTGAATCTCAGTCAGTCACACTTTGGTTCCTGACTCAAAATGATGCAGTTAGGCCCTCATCCCAGTTTGAATCTCAGATGACATGTTCCAGGACCCAAAATGAATTTGGTATAATCAATCCTTGGACCCATTCTGAAACTAATTCAGTCAGATCCTAGACCAAGCTTGAAACTGGTACAGGCCAACCCTGGCTCCATCTTGACGCTGCTACAATCAGACCGTGGACCCAGTATGAAACTTTAGAAATCCATCCCTCAACCCAGCCTGAGGCTGATACAGTAGTAAGACATTGGTTCCAGACACAAATGGATTCAATCAAAATTTGGAACCAGCCTGAGGCTGATACATCTAGACTCTGGACCCAAACTGAAACAGAAACAATTCAAATTTGGACCCAGACTGGAAGCAAAGTAGTAAAGCCTCCAAGCTTATCTGAAGTTGCTATAGTCACACCTCGGTTACAGACTCAAAGTGATACACCTAGATCCTGGATTCAACCTGACTTTCAGTCAATCAGTCTCTGGACCCAGACTGGAGTTGGTATAATTTGTGCCTGGTCTCAGCAAAGAGTTGCTATAAATCAACCCTGGGCATACCCTCAAACCCAAACCCAAGTCAGACCCTGGAACATTTTGGAAGCCAATACAATCAAATCTTGGTTCCATGTTCAAATGAATAAAGTCAGACCATGGGCCCCTTCAGAATCTCAAATATTGAGTCTGGATGCAGGCTGAAGTTGGTACAGTTAATCCTTGGATCCAGCCAGAAACCCCGGCAGTTAGACCTGGGGCCCACCTGGAAACTGCTAGTGTTGCATCTTTTGCTATTCCTAAAGCTAACAAAGTCAGAACATGGGTCCAACCTGAAATAGAAATAAGGCCTGACATTCATTATAAAGCTGATATAATCACATCATTTGCTTCTCCTGAAGTTGAGCCAGATGGAACAACCCAATTAACTAGTCACTTTGACTCCTCATCTAAACATGTAACTTTTTTACCAGTAGAAACTGTTCCTTCACCAGGTGAGTATTTTACAGCTTTGTCAGCTGAAACAGCTGCAGTAGAAAGCCAAGGTCAAATCAATTCTCTCCAACCCAGTGACCTCCCAAATATTCTCTTTCTTACTCTTTCAAGCACATGGCTTTCTGGAGGAGTTGGCTACTGGAACTTTGGCAATAAATTACAAATTACCAAAACAAAAGGAAGCCTGATGTTCCATCTACTTCTCTCAGCCCCCTTTCTCCATCTTTTTCCTTTCTTGTTCCTTGTTTTTTCCCATCCTCATGTTCTTTGTCCCTTTCCTGTTCAGCCTTTTCTTCTTGCACCTTCCCTTCATCCTGTACTTTCTGTTCTTGCTCAACTCTTTCTCCTGTGGCCTTCTCTCCTGTTCTCCTTCCCTTAGCTGATTCTGATAGTTCTCTACAGGGACTATCTTTCTCAAAATTTGCTGAAGGACCATTCTTTCTCATACTTTTTCATCCCTACGTGCTGCCCCAGCCACACCTTTAACAAACCAACCTTCTGTGATGCCTGCATCTCAATCTGGAACCAAGTCTAACCAGCCTGAACAAGATCCTCTCAAGTATTCAGAACTCAATATTTCCTTGGCTGAGTTTCGCCTAAGTGTGGTCTGGAAAGAGAGCCTCCGGGCTTTCTGGCTCTTCAAGACAGCTGTTATTTCTCATGAAACCACAGAGTGTGGATTACTCCCTGGCGTTGTCCCACACTGTCCCAACTGCTGGGAGGCAGGAGTGGGTGAATTCTGTGCAACTCTCCTTCTCCCAATTCTGTGCTGGCTCTATACTGAATGAACAGTGCATCCTTACTACAGCTAGATGTGTGAATTTCATAAAAAACTCAGAGGCATTGGCCCTGGTCCAGATGGGGCTTATTGATCTTCAGAGCCCTGCCCAAGCTCAAACCATAGGTATTCATCATGGTATGCCCTACCTAGGTCCCAAAGGACTTTTGGGACCTGGGCTAATCTTCCTGAAGTAGTCGCTACGTTTTCAACCCCTGGTGCTTCCTGTCTGCATGGAGGAGAGCCTAGAGCAAGAGAAAAACATACAACTATATGACTGCTGGCTACCCAGTTGGTCCCTCATGAGAGGAAGTCCTGGAATTCTGCAAAAAAAAGGCATCTAAGCATCCTGCAGGTCAGCACATGTGCCCAGTTTTGGCCCAAACTGAATGAATTTACTTTCTGCATGGAAGCCAAGAAAGCTATGGGGGAGGCTGGCTGTAAGGGTGACCTGGGGGCACCTCTTGTGTGTCATCTACAGCAAAAGGACACATGGGTGCAGGTGGGAATCTTGAGTCACTTTGATGAACATTGCACAAAGCCATACATCTTCAGCCAAGTGAGCCCTTTCCTTTTCTGGCTCCAGGGAGTTACACAACCCAGCCATGCACCCTGGTCCAAGCAAGGGCCCATGACTATCTCTGCTTCCATCTCCCTTTCAGTCTCTACCTCTGCAAATGCCTCAGCTTTTACCTCCACTCCTGCTTCTGTCCGGCCACACTTCATCTCTCTGCCACAGCCTCAGACTTTGGCAGATCGGATTTCTCTGAGATATGCCATGCCTTGGCAGGCCATGATCATCAGCTGTGGCAGTCAAATTTGCAGTGGTTCCATTGTTAGCAGCTCTTGGGTACTCACTGCTGCCCTCTGCGTCAGGAACATGAATCCTGAAGGCACTGCTGTAATACTGGGCCTGAGGCACCCTGGGGCACCTCTGAGAGTTAAGGTATCTACCATTCTACTGCATGAAAGATTCCAGTTGGTGAGTGGGGCAGCAAGAAATGATCTAGCATTGCTGCTCCTCCAAGAGGTGGAGACTCCCATTCAGCTTTTAGCACCCACGGGCCATCCGAAGAACCTGAATAGCTCAGAATGCTGGCTGTCTGGGCCACAGATGCTTAAGCCAGGAGAGACAGATGAAAATCCAGAAATATTACAGATGCAAGTGATGGGCACTTCAAGCTGTGCCCACCTCTCCCCTGACAGGGGCGGTTCTGCTGTGTGCTTCATTACACAAGACAAACACTCTCATACAAATGTGAAACCAGTGAGCCCAGGCAGTGCGGTTATATGCAGACCAATTTCTGGGAATGGCAGCTGGAGACAGACAGGCCTCACCAGTCTGAAAGCACTGGCTACCATTGTGAGCTCCCACTTCTCATGGATATTATCCACTTCAGCAACAGCAGGGCATCCCCTAACCCAGGAATGCATGCTTTGGGTGGAAAAGCCCAAGTCCTCTAGTGTCCTAAGACAGCCAAACACACTGCCGCTCTCTTCAATAATAATTATAGCAGTACACACCCTTTTGTAAGACAGTGGCTTGGTCAGTCAGTGCTAATCTATTAGGACGATGACAAAAAGAATATCAAAACAACATTACA-AAGATTAAGGCCCTACAAAATCTAAGTA

>Mole Rat, Fukomys damarensis

ATGCTGTATCTATTAAAAGCATTGAGCCTAAACATCAAGCTGTGCATTTACAAGGACCAAGATATATCAGATATAGTTGTACTCTCATGGTCCCATACTGAATCTCCAGCAGATAGTCTGTGGATACTGCAGCTCTCCTATGTATGGGCCCAGGATAAATCCAAAGCAGAAAATCTGTGGCCCCAGCCTACATCAGATTCAGTCATACCATTGTGGCCCCAGACTGAATGTCCACCAACAAATTTACAGCCTCATCCTGTATCTCTTACAATAATATCACCTTGGCATAAGACTGAATTTCTAGCAATGAATCCATGGTTGCTCTCTATAATTGATAACATCACATCACCATGGATACAAGCTGAATGTCTACCAGTAAACCTTTGGGAATTGCTCAGCTCTAATAGAAATGCTTCACCGCTGTGGTTCTCATCTGAATCTTTTGCTGTTAACCCAAATACACTCATCACACCTGATTTGATTACATCAGTGTGGCCCAAGGTTGATTATCCAGCACTAATCCCTCGGGCAGGACTTATGGCTAGTAAACCCATAGCATTAAAGAAAGGGTCTGAATTTCTAACCAAAAAGTCTCAGATTTTGTCTGTTTCTGATATGCTCACACCTTGGCCCCCAGCTGAGTTTCCATCAGTAATGCAATGGACAGAGGCACTATCACACTATCACACTATCACACTTCAGAAGCAGGCTGGTTTGCCCAAAGTGTATCTCTGGACTCCATATGAAAGTGAAGTCTTTACAAAATGGACCAATACTCAAATCCCACAAGAAAAGTATCCACCAAATAATGAAGCAATAAAGCACTGGGCATCTTCTGAAGATGATGTGGTTTTACCGCAGACCCAAGATAAATCTCTGGAAGAAAATGTCTGGATAGATGCTGAATATGAAACAGTCATCCCATGGAAACAGGGTAGTTTTTCAGAAGTGAAGGTTAGGACACAGCCAGAAACTGGTCAAGTTATACCTAAGGAAGTAATTCCTGGGATAGAAGGTATAGCTAAAATATTCACATTGTGGATAGAGTCTGAAACTCAAGCAATAAATACTTTGACAGAACCAGTAGCTGATGGAGTTACATTACAGACACAGACAGTGTCCCTAACAATAAATTCTTGGACAGAACTTCTCAATAAAGCCACACTGAGGACACTGCCCAAAACTCCATTCATGAGCACTTGGAGACATGGTGAAAACACAGTGGTAAACCTCAGGACACTGTCGGACTCTCAAACAGTAAATATCAAGACACAGCCTGAAACTCCACCCTTAACTCGCACGGCTGAATCTTTGTCAGTAAATTCCTGGACAGACACTAAAATAGCTGAAGTCAGGACACAGGTTGAATTGCTACCACCCAAGTATCCCTTGGCCATGGCTTCAAAATTCATACCCTGGACCAAGGCTGAACTTCCAACAGTAGGGCTCAGGAAAGGAGCCATAGATTCAAAAATCAAACACTGGATTCAGGTTGAATTGACCCCAGTCTATTTCTGGAAACAACCTGTGACTGACACTGTTGCAGATTTGGCTCAGGTTATATATCCAACAATAAACACCTGGGAACAGCCTTCATCTGTCCCTGTAACATTATGGACCTGTTCTGCACCTTCAGCCATCATTACCTGGATATATCCTGTATCTGAAACATTTATCTCATTGACTCAGAATGAATCAACAACATTATCTGATAAAGTCACATTGTGGACCCAAGCTGTACTTGCTTCAGGCAAGCCATGGAAGGAATCTGAATCTTTTATAGACATACATTGGACCCAGGCTGAGTCTATATCCTTTATTTCCTCCATGCAACTTGTGTATGATACACCCACACCACAGACCCTGGCTGAGTTTCTGGCAGCAATAACATGGATGCAGCCTGTGTCTGATGCCCAGATGCCATGGACCCAGATTGAATCTCCAGGAGTCAAGACTTGGATTGAAGTTAAAGCTTCCATGGATACAGAGTGGAGGCAAGACAATTCCCCACCTTTAAATCCATGGACAGAGACTGTAACCTATGCAATGTGGAGGCTAAGAGAACCTACAAAAGGGAATCTTGGGTCAAGGGCAGCCACAGTCACCACATGGGTCCAGTATGAATCTTCAGCACTCCAGACCTGGTCAGCCGCTTCTATGGCCACAGACTTGAAACAGACTTCAAGTTTGAATTCTTGCATGCAACTTGAAACTGACATAAGAATACTTTCCAAAATTCCTATGAAATCCTGGACTCTGCCTGGAGTTAATATAGTCTCACTGTCTGTGCAACCTCACACTTATACTACCCAATACTTGAATCAGATTGAAACTCAAGAATCTCTCCTGTGGACATATCAAGTTGAAAATACTAACACGTGGACTTTGCCGGAAGTTGGAGCATTCATATTCCAGACAGTACCTATACATCAAGCAGCCAGATCCTCACTCCAATTTAACTCTCATATTAGAAGTTGGTTTAAAATTTCAGCAGAAAATATCCAACCGTGGACCGGGGCAGAATTTAACAATCTTATATCATTTAGAGCTAGAAAAATAGAGCTGTGGGCCCAACATATCACTAACTCAGTAGCATCATTTATCCAATCAGAAATTGGTAGATTTGATCTCTGGACATCTAAAATAGATATAGCCAGATCCTGGAATATTTCAGACACTGATACAATACCAGCAGTCCAAAATGAATCTGGCCCAGTCAATAACTGGATCCAGTCAAAAACTGTTATAATCAGACCATGTACCCTAATTGAATCTGACACAGTCAGGCTCTGGACTCTGGACGCAACAGACACAGTCAAACTAATTTCACAGGCTGAAATGCAAGGAAAGAAACACTTGACTGTATCTGACATTTACACTGTCAGAGTGTGGTTCCAGTCTCAAAAGGAAACAATACAAGGAACTCAACTTGAAGCTCAAAGAGTTACTCCCTGGATCCAGCTAGCATTGCAAATAGCACAGGCACAGTACCATCCAGAAACTAATGGGATCCCACCTCTGTCCAAGGTTAACGGTGATGTAGCCCAACCTGAAATCCATTTTCAAACAAACCCAGTCACACTCTGGACCTATTTTGAAAGAAATGAAATAAAACTTTCTTCCCAGGCTGAATGCCAAGGGACTAAGACCTGGCCAGCAGCTGATTTGGTCACACTTTTATGTTTAACTCAAAATAAGGCAATTAGGCCTTGGTTCCTAGTTGAACCTCAAGTGACATATCCCTGCTCCCAGGCTGAAGTTATAATGAACCCTTGGATTCATTATAAAACTGGCATGATCAGACAATGGACCCAGTTTGAAACTCTAGAAATCCTTCTTTGGACCCAGCCAGTTGCTGTAATAGAATACGGGCTCAAGACTTGGACAGGTTCATTCAGACATTGGAACTGGGCTGAAAGTGAAACCATTCAAACTCAGACCCATACTGTGAAACAAATAGCAAAAACCCTAAACTTTGCTGAGGTTGATATGGTCACAAGTTCGTTACATGTTCAAGCCAATACTTCTAAACATAGGATTCAGCCTGATTCCCAAACACTGAGTCTATGGATCCAGAGTGAAATTGGTTTAACTGGGGCCTTGGCTCAGCAAAGAGCTGTTATAAACGACCCTGATACCCAATCAGGTGATATAGGAGCTCCTCTGGTGTGCCGCCTACAACAGAAGAACACCTGGGTACAGGTGGGAATATTGATTTACTTTGATGAACATTGCCAAAAGCCCTACATCTTCAGCCAACCTCAGAGGAAGACCCAAGACAATACTAGTAAAGAAGCTTTGACAGATCGGATTTCTCTTCATTATGCTATGCCTGGGCAGGCCATTATCATAAACTGTGGCAGTCAAGTCTGCAGTGGATCCGTTATTAGCAGCTCTTGGTTTCTCACTGCTGCCCACTGTGTCAGAAACATGACTCCTGAAGATTCTGTTGTCATACTGGGCTTTAGACATCCTGGGGCACCTCTGAGAGTTGTCAAGGTGTCTAACATTCTATTGCATGAGAGATTCCGGTTGGTGAATGGGGCAGCAAAAAATGATCTAGCATTATTGCTCCTTCAAGAAGTCCAGACACCCCTTCCGATATTAGCGCCTTTGGGCCATATGAAGAATCTAAGCAACTCAGATTGTTGGCTTACTGGACCACAAATTCTTAAACCAGGACATACAGATGAAAATCCAGAAATGTTACAGATTCAGGTTATGCAAACTTCGACCTGTGCCTATCTATACCCTGATATAGGTGGTTCTGTTATTTGCTTCATTGCTAAGGCCAAAGGTGCTGACACAATTATGGAGCCAGTGAGTCCGGGCAGTGCAGCTTTGTGCAGAGATGTGACTGAAAATGGCAGATGGAGACAAGTAGGCTTTGCCAGTGTCAAGGCTCTAGCTACCATTAACATATCAGCTCTGGGCCATTCTGAAAAGCAGCAGGAATTTGAAGGGGTTTGGTCAAAGAACTGGCACAAGGTGACTTGGAATGTAACATCAAGAGATGATCCGGCGGGCGCTTCCATCACAGCCCCAGTGACAGCCAACAAGTTGGGAAGGGGCTGTGGACTAGCTGCGAAAAGTAGGAACAGCAGCCAGGAGCCGGAGAGCCGACCCTACCCGTCTTACCTGCGGTTCAGCGCGAAGAGACAGCAACGCTCACGCACTGTCGCCCAAGGCCGGGGTTTGAGAGCGGGTGGAGATGCGAGGCTGGACTGGTGGAAAGAGAAGGTGGGGTACTGGCGGTCACAACCATTGCCAACCCACAGCACCAAGGCGCTGGGCGATTCCATCCAGCTGTTCCTGCCGCAAAGCACAGGCCGGCGACTTGTGGAGAGAGCGCGGCCTGGCGGCGGCGCGGCTTACTGCTCACCTTGTGTTGCGAGTCCAACAGGTGTACTTGATGAAGCCGCTGGTCTGAGGCGCTGCTCGCAGGAGCCCCCTGCCGGCTACAAGCCTGGGCGGAGCTTCCTCCAAAAGGAAGGGACATGGGAAGGTTCTGAATGCTATGTGATTATGACCGCAGAAAATACCTGTAGGAAATCTGTGGGACGGACATCTAAGTTCAGGCTTAAGGAATGA

>Monk Seal, Neomonachus schauinslandi

GTACAGCAGGGTAGTCCTGTTCTCTGCCTCTTAGGCAACCATTGGGAACTGGTAGGCCTGGTCAGTGAATCCTCAATGGCCTGTTATAACCCTGTTCTTGTCATCAAGACAGCCCCATATTTATCTTGGATGAAACGGCTTATCAAGACATCTCAGAAGCCATTGGATCCTATTTTTTCCCTCCCCTGCAGTTTTACTCCTGGGGTAGAACATGGTCCACAAGATAGGCTTAGCCTGAACAGGGGTACTACCATTTTGACCTCCCATGGATTCTCTGTACAGTCATGGAAGAGATTAGGCACTTTCCCACTGAACAGACAGCGCCGGAATCCTCCTCCCATATTTTTTCATTTAAATAATAGAGACTCTTTTCCTGGCAGTAGACAGTTACACCTTCAAACTAGTCAGCTCTCCTCAACTAGCAAATTCCCAATGATACAATCTTGGACATCTCTTGTTACCAAACAGCGGGATCCTTCTGATATTTCTGAGCCCTGGAATACCCCAATAGCTGGTACTTCTGAAACTTTGGTTCTCTCTGGACCCCCAAAGCCTCCAACATCTGATAAATCTATATGCTGGGACCTTCCTCAGAAAGATACAATGAAATATCAATACCAAACTATGACCAATTCAGTAAACTCCTGGGTTAATCCGTTAGCTGGTATAATTGGGCTTCATACTCTACCATTAGTTAATTCTGCTATATCCTGGGTTTTGTTCTCAAGTGGCATAGATGGATCCCAACTTCTTTCTGGGGTTAATACTGTACAGTTTCAAGTTCGGTCTAGTAAGGTTCCTTTCCACGGTCAACTTCTAACTAGACCCTGGCTAGAAATTACCCCTGACATTGGACCTTGGACACATTCTGTACCTGATAAAACAGGAACAGTGATGCAGATCCAATCGAGTGAAGAGAATATTGGAAGCCAAATACACCATGTAGTTGATGGAGTTCACACTGCTTTTAAACCAGTAACTTTTAACTTGAATACATGGGTTCCTTTAACAGTTAATAAAAATGAATTCTGGACCCATTCCACACTAAATGCAGATGGATCTCAGTATCCTACAGTAATGCTTACGTTGGAACCCTGGTTTCAGTCAGTCTTAAATTTAGATGGATCCCCAGAACTTACAGAAAAGACTAATGAATACTGGATTGTCCCTGAATCTAAGTCAGCTCAATTGTGGACTTCTTCAGCACTTAATATGCCTTTTACTTGGGTTCCATCTGCAAGCAACACTATTAAGTCTTGGGCACAATATAAGACCAGTCTGAGCAAAGCCTCAAGTCAAATGGATAGAGTAAGTCCATTGAGTAAACATGAATCTTTTATGGTCAAACCCCAGATTCAAACTGCAGATACAACCTGGTTTTTGATACATACTATTACAAGTGTAATTAAGCCATTTATTCAGTCTAAAGCTGATACAACCAGGCCCTGGACTAAACCTGAAGCTAACATAATCCAAACCTGGACCCAGCCAGAAACCCAGGCAGGAAAACCATTGACTCAGCTGAAAGCAGATATAATCAGACCATGGTTACAGACTAAAACTGAAAGAATCAGACACTGGATTCAGCCTAAGTTTCAAATAGTCAGACCTGGAGTCCAGACTGAAAATGGTAAAGACAAACATTGGACCCAGCCAGAAGCAGATATAATTAGATCCATGACACACACTGAAATTGAAACAGTCAAACTTGGGAACAAGCCGAAAGCTGGTATAGCCAGATCCTGGTTTTGGACTCGGTCTAATCAAATGAGAGCAAGGTCTCAGCCAGACTTTCAAACACTCTACCCTTGGACTCAGCCTGAAGTTGACATAGTGAGACCATGGACTCAGTCCGAAGCTGGTAGCATCCAACCATCAATGAAGCCTGAAGCATTGACATTCAGAATCTGGACTCAGTCTAAAGTTAATACAATTATACCCTGGACAGGGCCTGAAGCTGATGCAGCTAGATTTTGGTTGCAAATACAAACCAATACAGTCAAAACATGGAATGAACTTGAATCTCAAACAACCATTTCCTGGTCTGAGCTTGAAGCTGATAGAGTCAGATCTTGGTTGTATACTCAAATGCATACATTCAAACCTCAGACTGAGACAGAATTTCAAACAGTCCATTCCTGGACCCAACCTGAAGGTGACATAGCCAGGCTTTGGACTAAATCTGAGGTTGACAATGTCAGACATTGGTTCCAGACTCAAGTGGAGACACCCACAACGTGGACAGAGCCAGTATCCCAAGTAGCTCACCACTGGATACAGTCCAAAACAGAAATAGTTAGGCCCTGGAACCAACCTGTGGCAGACAAGCTAAGATCCTGGATACAACATGACATTTACACTGTAAGGCCCTGGGATGAGCTTGAAGGTGATAAAGTTAGATTCTGGACACAATCTGAATCGGACACAATACCTTGGATTCAGCCAGATGTGGGTATAATCAATCCCGGGGCACAAAATGAAGCTGATACATCAATACCATGGGCATGGGTACAGGCTGAGAATCCAGAAGTTAATCCCTGGGCACAGTCTGAAACTGAAACAGTTATACTCTCAACACAGGGAGAAGCTCCAGCAATTAATCATTGGACAGATATTTTAGCTGATACTGTTACAACATGGGCAAAGACTGAATTTCCAGGAAAAAGGCTCTTGACCTATCCTTTGTCTCGTACAGTCACACAGTGGACTCAGCCTGAACTGCCGGTGGCAAATCTCTGGACACAGCCTGTAGATGATACAGTCAGTCAGTGGAACCAGAGTGAACCTTCAGAAATAAATTCCTCAACAAAAACTATAGCTGACACAGCCATCTGGACACAGTCTGAATCTCCAGCAGTGAATCCCTGGATACAGTCTGAAAATGATACAGTCATATCATGGACCCAGGCTGAGTCCCTAGCAGTAAATCCCTGGACAAAGCCTATGGCTGATACAGTCACACAATGGGCCCAGGGTGAACCTTCAGAAGTACTTCCTTGGGTGAAAATTGTAGCTGATACACCATGGATGCAGGCTGAGTCCCCAGCAGTAAATCCCTGGATACAGTCTGAAACTAACACAGTCCTACTGTGGACACAGGCTGAATCTCCAGCAGTAAATCCCTGGATAGATACTGTAGCTGGGACAGGCACACAATGGACTCAAGCTGAATCTCTAGCAGTAAATTCTTGGACCCAGCCTGTAGCTGATACAGTCACACTATGTACTCAGAATGAGTCTCCATTATTATATTCCTTGAAAAAGTCTGAAACTCATACACTTACAACATGGACTCTGACAGAATCTTTAGCAGGAAATTCCTGGAGACAGTATGAAACTGATAGTGCCCCAATGTGGACCAAAAAAGGAAATCTAGAAGTAAATCCCTGGGCACAATCTGAAACTGGTACAGTCACAACTTGGACCCAGGCAGAAACTCCAGGAGTAAATTTGTGGCCACAACCTATAGCTGAAATAGTTTCACCATGGACTGTGGCTGAATCTCCAGCAGTAAATCCCTGGACAGAGACTGAGACTGTATTTGATGGTGTTATACCATGGACCCAGGTTGAATCTCCAGCAGAAAATCCTTGGACACAGTCTATAGCTGATGTAGACCTGACTGATTCTATAGCACTAAATTCCTGGACAGAGCGTATATCTGATAGTGTTATACAGTGGACCCAGAGTGAACCTCCAGCCATAAATCAGTGGACACAAACTGTATCAGATACAGTCACATCATTTACTGAGGATGAATTTCATGCAGTAGAGACCTGGACAGACCCTTCAGCTGATGTCACATCATGGACCCAGACTGAAACTCCAGTAGTAGATCCCTGGTCAGAGACTGTAGTTTTCACTGTCACACCATGGACCCAGACAAAATCACCAGTAGTAAATCCACTGATAGAGGCTATAGCTGCCACAGTATTACCATGGAATCAAGCTGGATCTGCAGCAGTAAATCCATGGATAGATGTTGTAGGTTTCACAGTAACACTATTGACTCAGGATCAGTCTCCATCAGTAAAAACCTGGACAGAGGTTGTGGCTTTCACAATGGAAACACTGACTCAGGCTGAATCTCTAGCAATAAAGTCTGTGTCACAGGGAGTATCTGATACAATCTTACTGTGGAACCAAGCTGGATCACCTCTAGTACATCCCTGGACACAGTCTGAAACTGATGCAATCACACAGTGGACTCAGAGTGAATCTCTAAAAGTAAATCCTTGGACACAGCCTTTGGCTGACACAATCACACCGTGGACTCATGATGAGTCCCCAGCAGTAAATCCCTGGACACAATCTGAAACTGACACAATCACAGCATGGACCCAGGCTGAATCTCCTGTAGTAAATCTCTGGACACAACCTGGAAATGTTACAGTCACACTGTGGATCCAGGATGAATCTCTTGCAGTAAATACCTGGACACAGCCTGAAAATAACACAGTCACACCATGGACTCAGAATGAATCACCAGAAAAAAATACCTGGACAGAGGCTGTTTCTGAAACAGCCATGCCATGGACCATGGGATTTTTTCCAACCATGAAGCCCTGGATAGAGACTAAATCTGATACAGTCACACCAGGCACCAAATCTCAATTTTCAGAAGTAAAACTTTGGACACAGCAGTTGTCCAAAACATTGGACACTGAAACAGGTACAGTCAAAACGTGGACTCAGTCTGAATCTCCACCCTTAATTCCCAGCATAGACGCTATAACTTCCGTAGTCCCACTGTGGACCCAGGCTGAATCTCTAGCTGTAAATCAGTGGACACAACCTATAGCTTATAGAGTCACGGAATGGACACAGACTAAATCTCCATCAGTAAGTACTTGGACTCAGGTTCAATTTCTGGCAGTAAATCCCTGGACACAGTCTGAATCAGAGTACAAATCTCCAGCCCTAAAACCATCAGTTGAGCCTGAGGCTAGTGTAGTCACATTGTGGACTCAGACTGAATCTACAGAAGTAAATCTTTGGATGGAGCCTGTAGCTTCCATAGTCGTGCCATGGATACAAGCTGAATATCCTGCAGTAAGTTCATTGACACAGGCTGTAGCTGATACAATTATACTGTGGCCTCAGGCTGAATCTCCAGCATTAAATCCCTGGACAGAGTCTGTAGCAGATACAGTCACAGTGTGGGCCCTGGATGAATCTTCAAGAGCAAAACCTTGGACACAGATATTAGTTTCATCAGATATATTTTGGACCCAAGCCAAAACAGTAAATCCTTGGTCTACTGTTGAAATATTCCCACAATTCACCCAAATTCAATCTGTAGTGGTTAAACCTTGGACACAATTTGAGAGTGACACAGTCACAGCACTAACTCAGAGTCAGGCTCTTGCTATGAATCCCTGGATAGAGATTATCACTTCCAGAGTCAATCCATGGACCCAAGCTGTACCTCCAGAAGCAAATCCCTGGACAGAGATTATCACTTCCAGAGTCACATCATGGACCCAAGCTGTACCTCCAGCAGTAAATCCCTGGACAGACGCTGGTGCATCCAGAGTCACATCATGGGCCCAAGCTGTACCTCCAGCAGTAAATCCCTGGACAGGGACTGGTGCATCCAGAGTCACACCATGGACCCAAGCTGTACCTCCAGCAATAAATCCCTGGACAGACAGTGGTACATCCAGAGTCACACCATGGACCCAAGCTGTACCTCCAGCCGTAAACCCCTGGACAGATGCTGGTGCATCCAGAGTCACTCCATGGACCCAAACTGTACCTCCAGCAATAAATCCCTGGACAGACAGTGGTACATCCAGAGTCACACCATGGACTCAAGCTGTACCTCCAGCAGTAAATCCCTGGATAGAGGCTCTTGCTTCCAGAGTCATGTCATGGAACCAAGGTGTGTTTCCAGCAGTAAATCCCTGGACAATGATACTTGTTTCTACAGTCACACCATGGACCCAGGCTACCTCTCCTCTAAGTCCTTTGACAGAGACTAAAGCTTCTACAGTGAGAATATGGACTCAGAATGAATATTCATTAGTAAAATCCTGGGCACATTCTGCAATTTCCACAGTTACATCTTGGACTCAGTCTGAATATCAAGCAGTAAATTCTTATATACCAAGTGTAACTGATACGGTCATATTTTGGACTGTACTTATATCTGAGTCTAAGAAATCATGGATACTGCCTGAAGCTGGTATATTCAGTATTTCATTGCATCCTCAAAGTGATTCTACTCAACCCTTGATTCAAGGAGAAAATCAAGCATCTCTTCTGTCAACACATCCTGGAATTAATAATGTTAATACATGGCCTTTGCCTGAATTTGAAACACTGATATCATGGATAGTGCCTTTGTCTCAAGCAGCCAGACTCTTACCCCTATCTGAAACTGATATTAGCAGATATTGGTTTAAAACTGAAACAGAAAGAGTAAGAACCTTGACCCACTCAGAACTTCATACAGTGAGTACTTTGACCCAGTTTGAAACTGATAGATTTGAGCCCTTGGCCCAACGTGAAACTCCTATAGTCATATCATGGATTCCAACTAAAACTGGTATATCCCACCTCTGGAATAAGTCTGAAAACTACAAAGTAAGAACCTGGACCCTTTCTGGAGGTGATGCCTTGCGACCATGGATTCAGATTGAAGCTAGTATATTCAACCTCTGGATCCAGTCTAAAAGCAGTACAGTCACACACTGGACCCAGCCTGAGTCTCAGTCAGTCAGTATCTGGACTGAAAGAAATACAGGCACATTTTGGTACCTGACTCAAAATGATGTAGTTAGGCCTTGGTCCCAGCTTGAATCTCAGATGACATCTTCTGGGACCCAAAATGGTATAAGTAGCTCTTGGACTCAGTATGAAACTAGTACAGTCAGATCCTGGACCAAGCTTGAAATCAGTACAGTGCAACCCTGGATTCAGGTTGAAACTGCTACAATTAGATCATGGACCCAGTCTGAAAATATAGAAATATACCCCCTGACCCAGCTAGAAGCTGATACAGTAATAAGACACTGGTTCCAGACTAAACTGGATTCAATAAAACCTTGGAACCAGCCTGAAGCCAATACAATTAGATCATGGACTCAGCCTGAAACTGAAACAATCCAAATTTGGACCCAGACAGAGGGGCAAGTAGTAAAACCTCCAATTTTATTGGAAGTTGATACAATTACATCTTGGTTACAGACTCAAAGTGATACAGTTCGACCCTGGATTAAATCTGACTCCCAGTCTGTCAGTCCCTGGAGTCAGGCTGATGGTATAAATCACCCCTGGATTCAGCAAAGAGGTACTGTGAATCAACCCTACTCGGAAATCCAAACAGTCAGACCCTGGATGAAGCTAGAAACTGATGCACTTAGATCTTGGTTCTACATTCAAATGAATAAAGTCAGACCATGGACCAATTCCGAATCTCAGATCTTCAGCTCCAGGTTGCAGCCTGAAGTTGGTATGGTTCACCCTTGGATCCATCCTGAAACCCAAGCAGTGAGATCCTGGGCCCAATCTGAAACTGGTATGATTACATCATTTGTTATTCATAAACATGACAAAGTCAGAACATGGATCCATACTGAAGTAGAAGTCAGACCTGACAGGCAGTATAAAGCTGATAGAATTACATCATTTGTTCCTTCTGAGCTCTGTCAAGGAACTGTCTTCCTCAAATTTACTGAAGAGCACATTCTTTCTCATACTTTTTCATATCGGCTTGCTGCTCCAGCCACCCTTTTAAGAAAAGAACCTTCTCTGATGCCTGGGTCTCAATCTGGAACCAAGTCTAATCAGCCTGAACAAGATCCTCTCAAGTATTCACAACTCAATGTTTCTTTGGCTGAGTGTCACCTGGATGTGGCCTGGAAAGAGAGTGTCCAGGCTTTCTGGCTCTTCAAGACAGCTGTTATTTCTCATGAAGCCACAGGCAAAAAAAACTCAGAAGCACTAGCTCTGGTCCAAGTGGGGCTTACTGATCTTCAGGAGCCTGCTCAAGCTCAAATTGTAGGCATTCACCGTGCCATGCCCTACCTAGGTCCCAAGGGACCTTTGGGTCCTGGGCTAATCTTCCTGAAACAGCCCCTACATTTTCAACCCTTGGTGCTTCCTATCTGCCTAGAGGAGAACCTGGAGCAAGAGAAAAATATACAGCTGTATGACTGCTGGCTACCCAGTTGGTCCCTCATGAGAGGAAGTCCTGGAATTCTGCAGAAAAGGCACCTAAGCATACTGCAAGTCAGCACATGTGCCCAGTTTTGGCCCAAGCTGAATGAATTCACTTTCTGTGTGGAAGCCAAGAAAGCTCTTGGCGAGGCTGGCTGTAAGGGTGACTTAGGGGCACCTCTGGTGTGCCATCTACAACAAAAGGACACATGGGTGCAGGTGGGAATTTTGAGTCACTTCGATGAACATTGCACAAAGCCCTACGTCTTCAGCCAAGGAGTTACACGGCCCAGCCATGCACCATGGTCCCAGCCAGGGGCCATGACTACCTCTGCTTCCACCTCCCTTTCAGTCTCTACCTCTACAAACGCCTCAGCTTTTACTGCCACTCCTGCTTCTGTTCAGCCACAGTTCATCTCTCTGCCACAGCCTCAGAGTAAGGCCCAAGAAAATGGTAGTGAGGGAGCTTTAGCAGATCGAATTTCTCTACGATATGCCATGCCTTGGCAAGCCATAATCATCAGCTGTGGCAGTCAAATTTGCAGTGGTTCCATTGTTAGCAGCTCTTGGGTTCTCACTGCTGCCCACTGTGTCAGGAACATGAATCCTGAAGACACTGCTGTAATATTAGGCCTGAAGCACCCTGGAACACCTCTGAGAGTTGTTAAGGTGTCTAACATTTTACTGCATGAGAGATTTCGGTTGGTGAGTGGGGCAGCAAGAAATGATCTAGCACTGCTGCTCCTTCAAGAGGTCCAGACTCCCATTCAGCTCTTAGCACCACTGGGACATCTGAAGAACCTCAATAGCTCAGAATGCTGGCTTTCTGGGCCAAGAGTTCTCAAACCAGGAGAGACAGATGAGAATCCAGAAATGTTACAGATGCAGGTGATGGGAGCTTCAGGCTGTGCCCACCTCTACCCTGACATTGGCAGTTCTATTATCTGCTTCATTAGTCCCGCGAAAGGCTCTGATACAAATGTGAGAGAGACAGTGAGAGAGGGAATACAAGCAGGGGGAGTGGGAGAGGGAGAAGCAGGCCCCCCGCCGAACAGGGAGCCTGATGTGGGGCTCGATCCCAGGACCCTGGGATCCTGA

>Mouse, Mus musculus (KJ780361)

ATGAAAACCATCTCACAGTGGACACAGCCAATAACTCAAGCAGAATACAAAGGAAGAATATCTGAAACTGAAAAAAATAGGCTCTGGAAACCACCTATGCTTGATGAAGTAAGAAACTGGATACATCATGAAGTTTATACAATTAAGATCTTGGATGATTTAGATGTTGGTATAATTAAACCAAGGGTACAGAAAGAAGCCAAAACATTAATAAAACGGAGGCAGGCTGAAACTCCAAATGTAAATTCCCAGATACCATATGAAATTGACATAGCCAATTTATGGAATCAAGATGAAACACCAATAACTAATGACTGGAAGGACTTATTAGAAGATACAACATGGATGAATTCGCAACTTCAAGAACTAAATGACTGGACACAATCTAAAATTGATAGATTCACAAGATGGACAAAGACAGAAACACCACTATCAAGTCTCAGGACTTTGTCTGAAATGGATACATTTACACAATCAGCCATAAGTAAATCTTCAAACATAAATCTCTTGATAGATTCTATAGTTTACACTGTGCCATCATGGACCAAAACTCACTATCCAGAAATAAATTCTCCAGTAAAGTTTAAGGGTGAAATAATTACAATATTGGCCCCTGAGGAATATCCTACACTAAATCTATGGAAACAGATTGAAGTAGACATAACCACAAAACTGACCCACATTGAATCATCGCTGGGATCTTCATTTACATTTCCAGGGTCTACTATAACCATTTTTAACCAGGTGAATTCTCCATCAGGAATGCATTCAGTGAATCCTGACTATGATGAAATAACTACATGGCTCCCATCCAAAACACTATCAATAAATCTCTGGACTTCACCAGGTAGGAATTCAATCTTGACTCATGAAGCTTCAGGAAAAACTCTCTTCTCAGAAATAAAATATACAGATATTCCATGGTTTCACACTGATGTTTTTAGAGAAATCTCATTGACACAGGTTGAAACTATCTCAGAACTGGTACTAGATAATGCTGAAACTGTAAGTGTGTGGGCACAGATTCTTGATACTTTGGAAAAATCATGGACAAAAGATAAATTCCAAATACTCACTTCCTTCAGAGGGATGAGAACTGATAGTTTTACACCATTGATTCAGACAGAGTCTCCAACATCTAATACATGGCCACTCCATGTATCTGATAAAATCAATTTATGGACTCAAGCTATACATCCAACAGGATATCAGTTGACAGAAAGTGTAGCTCCAACTGTCACAGTGTGGCTGAAAAGCAATCCTCTGTTAGTAAATTTATGGCTACAGGAAATATCTGATAGAGTTGTAATGCCATTGCCCCAGGTTGAATACCCACCATTAATCCTGCAGTCACTGGCTGTAACTTACACAGTCATATCACAGTTACTTCAAGCTGAATATGCACCAATTAATTTGTGGAGAAAACCTGTGTCTGATATAGTCAGAATACCATGGAATCAACCTGAATTATTTAATCAGAAAACACATCTGGTATCTAATACAAGAATGCTTCCATGGCCTGAGCCTGAACAGATACCCGAGTATTTATGGCTACTGCCAATAAGTGATACACTCATACCACAGTGGCCCCAGAATAAATCTCCTAATGTAAATATGGTGTCACCACTAAACTTTGATGCACTGATAATATCTACATCACCAGGACCACAGAAGCCCACATCTGAAATGAATCTAAAGATACTACTTGCATCTGATAAAAAGACAACAGTTTGGTCTCAGAGTGAATATTCAACAGGAAATATGCTTATATTACCTATGTATGACAGAGTCACATCTAGGTCCCACAAAGTAGAGTCCTATGAACAACATAAAATGGGTATGCATTCTGCTACTGGTGTATCTAATGACTGGATCCAATCTGAAACAGAAAGTATAAGATTCTGGAATGTTTCAAAAGACAATACATTAAGGCCACAGTTACAAATTGAAGAGAGCACAGACAATGCTTCAATCCAGCTGAAATCTAATACAGTAAAAGTGTGGTCTCAGGCTGAATCAGAAGTAATTAGGCTTTGGACTGAGTCTGTATCTAATATACCATTGTCACAAGCTAAAATAGAACCAGAAAAGTACTTGAACATGTCTGACATTAATGCTATTTTATCTTGGTTCCAGATTCAAAAACTTTCAACAAGACAAAGAACTAAACTTGAATACAAAACTGTTAACCCCTGGCTCCAGCAAGAATGGCAAATAGCACATTCCTGGAATGAACTTGAAATAAAGGTAGTAAAAACTAGGTCAGATTTTAAAACAGATGCATCCCAGATATTCTTAACCTATACAAAGTCTGATGAAATTAAACTTTGGACTCAAGCTGAATCGCAGCTTGTCAGGAGATGGCCTGAAGATGTTATATTCACACTTTGGTCCCTGACTAAGAATGATGCTATTAAGCCATGGCCAGAACTGGAGTATAAAACTACACAATCTTTGGCACATCTTAGTGTTGGTATAATCAATCCTTGGATTCAGTGTAAGCCTTCTACTGAAACTCTAAATATGATTTCCTGGATTCATTTTGATACAATAATACCATACTCATTACAGACTGAAATAGATTCTACATTTCAATCTCTGACTCAAATCTATGCAATTAGGCAATCCTCACGACCAGAATATGAAACTACAGAATTGTTTACACATTCTAATGTTGGTATAACCTATCCTTGGATTCAGCATGCGACTTCTACTGAAACTCTAAAAATCATTCCCTGGATTCATTCTGGTACAGTAATATCATACTCATTACAGACTCAAATAGATTCCTTATTTCAGTCCCTATATGAAATCTATTCAATTAGGCAACTGTCACATCCAGAATTTGATATTACAAAATATTTGGCACATCCCAATGTTGGTATAATCAAACCTTGGGCTCTGCATAAGTCATCTACTGAAACTCTAAGAATCATTCCCTGGGCCCCTTCTGATACAGTAATATCATACCCATTACAGACTGAAATAGATTCTACATTTCAGTTCATGACTCAAATCTATGCCATTAGACAATGGTCACAACAGGAATATGAAACTAAAGAATCATCGACACAGCCTAATGTTGGTATAACTTACCCTTGGATTAAGCATAAAGGTTCTACTGAAACTCTAGATATCATTTCATGGACTCATTCTAGTACATTATTATACCCATTAGAGACTCAAATAGATTTCACACTTCAGGATCTACCTCAGAATTATGCACTTAAACAATCATCACAGTCAGAATATGAAGCTACAAGATCTTTGACACAAACTAATGGTTATAAAATCAACCCTTGGATTCAACACAAAGCATCCACTGAAACTCAAAAAATAATTTTCTGGACTCACTCTGATGCAGTAATATCATATTCAATGCAGACTCTAATAGATTCATTTAGATTTTGGAACCAGTTTCAACCAAAACCAACCCAAATTTGGAATCCAACGGCAGAACAATTTGGAGAAACATCAGTTTTGACTAAAGTTGGTACAGTAACACCATCAGTTCAGTTTCAAGACACACTTTTAAGGAAAGAACCTATGTTGATGCCTCCATCTCAACCTGGCCTTCAAAATAAGCATTTAATAGAACCACAATCAAGAATCAATGTTTCATTGTCTGAATGTCATCTGAGTATGATATGGAAAGATAATCTTCAAGTTCTATGGCTCTACAAAACAGCAATAGTTGATCATAAAACCACAGAATGTGGCTTACGTCCTGGACTTGTTTCCCATTGTCCCAACTGCTGGGAAGCTGAAATAGGTGAATTTCCCTGGATAGTCTCTGTGCAACTTTCTTACTCACACTTCTGTGCAGGCTCTATACTTAATGAAAGGTGGATTCTTACATCTGCTCGATGTGCCAATTTTGTAAAACGGTCAGAAGCCCTGGCTTTAGTCCAAGTGGGGCTGGTTGATCTTCAAGACCCTACCCAAGGTAAAATCGTAGGTATCCACCGTTCCATGCCATATTTAGGTCCTAGTGGACCATTAGGACCTGGTCTAATCTTCCTGAAGGAGCCCCTTCATTTTCAACCTTTGGTGCTTCCTATTTGCCTGGAGGAGAGTCAGGAGCAAGAGAAACATATCCAGCTATATGACTGCTGGTTACCCAGTTGGTCCCTTATGAGGGGAAGTCCTGGCATCCTGCAAAAAAGGCACCTAAGCATCATGAAAGTCAGCACTTGTGCCAAATTTTGGCCTCAACTGAAAGAGTTCACTTTCTGTGTAGAAGCCAAGAAAGCTATGGGGGAATCTGGTTGTAAGGGTGATCTTGGGGCACCATTGGTATGTCATCTGCGACATAAAGATACATGGATACAGATGGGAATTTTAATTCACTTTGATGAAAACTGCAATAAGCCCTATGTCTTCAGCCATATTAGCCCTTTTATTTTGTGGCTTCAGAGAGTTACACAGCCCAGCTATGCCCCCTGGTCCAAACAAAGATCTGTGACTATTTCAGTTTCCAATTCCTTGTCAGTCTTTACCAAGAGAAAACTCTCTACTTTGAGATCTTCAACTGACTCTATTCATCCACACTACATCTCTCTGTCACAGCCTCAGGCTTTGGCAGATCATATTTCTCTGCAATATACTATGCCATGGCAAGTTCTTATTTTCAGTTGTGGCAATCAGATCTGCAGTGGCTCCATAATTAGTAGCTATTGGATTCTCACTTCTGCCTACTGTGTCAGGCACATGAATCCAAAAGATACTGTTGTGATACTGGGCCTTCGGAATCCCAGGGATACCTCTGAGAGTTGTTAA

>Mouse Lemur, Microcebus murinus (BK059441)

CCTAATTTATAAATATGTATTATGTATAAACATATATAATATATGTATCTATATGATCTATAATTCATAAAGATGTGTAATTTATATATAATTTTTACATGCACATTTTAATATATGTGTGTTATATGTCTGTCAATCTGTATCATTACTTGTATGGATATAGATATATATATATGGATTATTTATGGATTATATATGGATTATTTTTGTTTTTTCATTCAGGTTCAGCAAGGTAGTCCTGTTTTCTGTGGCTTTGGCAGTCACTGGGAACTGGTAGGCCTGGTCAGTGGGAACTCAGTGGCCTGTTATGACCCTATTCTTGTCATCAAGACAGCCCCATACATATCGTGGATGAGATGGCTTATCAAGGCATTCCAGAAGCCACTGGATCCTATTTTTTCCGTAGCCTGCAATTTTCCTCCTGGAGTAGAACATGGTCCACAAAATAGGCTTAGTCCTAGCACAGGCTTTGTCCCTTTAGCCTCTCACCGATTCTCTATACAATCATGGAGAGGAAGATTAGGCACTTCCCCACTGAACAGACAACGACGGGATCCTCCTTCAGTAAATTTTGATTCAAATAACAGAGTATTTTTTCCTGGCAATAGACAGTTATACTTTCAAAGTAGTCAGCTCTCCGTGGCTAGCAAATTCCCAATGATACAATCCTGGACCTCTCCTGTTTACAAACCATGGGATCCTTTTGCTATAGCTGAGTCCTGGAGTACCCCAGTAGCTGATACTTTTCAATCTTTGGTTCTTTCTGGACTCAAGAACTCCCAAACAGCTGCTGTATCTATACCTTGGGACTTTCCTCAGAGAAATGCGATGCAATATCAATATCAATCTTTGACTGACTCAGTAAGATCCTGGATTAAACCATTAGCTGATATAAGTGGGTTTCATACTGTACCATTATTTAATGCTGGTGATGGATCATGGGTTTTGTTTTCAAGTGACATAGATGAATCCCAATTTTCTTCTGAGATTAATGCTGTTCAGTCTCAAATTGAATCTAGTGAAGTTCCTCCTAATGTTCAACTTATAGGTAGACCATGGCTAGAAATTATTCCTGATGTTGGGACTTGGAAACAATTTGTGCCTTATAAAACAGGAACAGTGATACAGATTCAATCTAGTGAAGAGGGCATTAGAACTCAACTATACCATGTAGAGGATAGAGTGAATACTGTTGTTGATCCAGTAACCTCTAACCTACATCCATTGGTCCCTTTAATGGTTAATAAAATTGAATTATGGGCTCATTCTACACTCAGTGAAGAAGGATCCCACTATCCTACAGTGACCTATAATTTGGATCCATTGTTTCAGCCAGTCTTGAATATGTTTGGAACAGAAGAACCTATGGAAAAGACAAATGGATACTGGATTCTCTCTGAATCTAAATCAACTCAATTTTGGACTTCCTTCACACTTAATATGCCTTTCTCCCGGGCTCTGTCTTCTGATGATATGACTAGATCTTGGACTCAATACAAAGCTAATATGATCAGACCCTCAAATCAATTGAATAGAATTAATCCACTGAATAAGCATCAGGCTATTGTACTCAAACCCCAGATGATGGCTAATGATAAAACATGGCCTTTGATCTATCCTATTACAAATGTAATTGAGCCCTTGATTCACTCTAAATTTGATGCTATCAGACCCTGGACTCATCCAGAAGCAGATATATTCCAAACCTGGATCCAGCCAGAATCCCAATCAGAAAAACTATTGACTCAGCCAGAAGTTATTACAAGTAGATCATGGTCACAGACTACAACTGAAAGAATAAGACCCTGGATTCAACCTAAATTTAGACTATTCAGAACTCAGACTGAGGAAGGTAAAGGTAAACAATGGACCCAGCCAGAAGCAGTTACTGTTAGATCCTGGGCCCACACTGATATTAAAACAATCATACTCTGGAACCAGCCTGAATCTGATTCAGTCACAGATTTGTTCTTGGCTAAATTGGATCAAATGAAAACAATAATCCAACCAGACTTTAACACTGTCCATGCCTGGACCCAGCCTATAATCAGACTATGGACTTTGTCTGAAGTTGGTAGCATCCAACCATGGACAAAGCCTGAAACATTTACATTCAGGACTTGGATACAGTCTGAAGTTAATATGGTCACACCCTGGACAAAACTTGAAGCTGATGCAGTTAGACTTTGGTTCCAGACACAAGCAAATGCAGTAAGAACTTGGAGCCAACCTGAATCTCACGCCATCCTTTCCTGGTCTGAGAGTGAAGCTGACAGAGTCAGATCTTGGTTCTATACACAAACTGATTCATTCAAACCTTGGATTGAGACAGAAATTCAAACAGTTCACCCCTGGACCCAGTCTGAAGGTGATGTAGCCCAACTTTGGACTCAGACTGAGGCTGATACTGTAAAACCTTGGTTCCAGATTGAAGCAAAAACAGTCACACAATGGACAGAGAAAGTAACTCAGACAGCTCACCACTGGATACAGTATGAAAGTGAAATGGTCAGGCCCTGGAACCAACCTATGGCTGATAAATTAAGAGCTTGGATACAACATGAAGCTCATAAAATTAGACCTTGGGATAAGCTTGAAGGTGATAAAATTAAATTCTGGACCCAGTCTGAAGCTGACACATTGAGATCCTCCATTCAAGCAGACATTGATATAATTAACCCTTGGGTACAAAATGAAGATACACTAATACCGTGGATTCAGGAAGAGTCTAAGAAATTAAATCCCTGGACACAGTCTGAAACTGATCCAGTCACACTGTGGACCCATGTAGAAATTCCAGAAGTAAATCCCTGGGCACAATCTGAAACTGACACAGTCACACTGTGGACCCAGACTGAAACTCCAACAATATATCATTGGGCAGAGATGCTAGCTGATATAGTTACAACATGGACAAAGGATGAATTTCAAGGATTAAAGCCCTGGAGAGAATCTGAAACTGAAATAATTACATCATGGATTCATACTGAATCTTCAGGAGTGACTCTGTGGACACAGACTGTATTGGATACAATCATATTGTGGACTCAAGATGAATCTCCAGCTTTAATTTCCTTGACAGAGTCTGTACCTGATCCAGTCACACCGGCAACCCAAGATGTACTTCCAGAAATGAATCCCCAGGCTGGACTGCTAACAATAAATCCCTGGACAAAAATTGTGCATAATATAGTCACACCATGGTTGCAACCTAAATCTCCAGCAGTAAATACATGGATCCTGTCTGAATTGGATCCATTTTCATTGTGGACACGGGCTGATTTGTCCTGGACACAAACTGGCACTGAATCAATTACACTGTGGACCCAGTCACAGTCTCCAGCAATAAATCCATGGAATCAGCTTGAAACTGATATAATCACACCAATGACACACACTGAATCACCAATAGTAAATTCATGGACATATTCTGTATCCAATACAGTCACAATGTATACCCACACTGACTCATTAGATCCATGGACACATGATACAGTCACAATATCAATTCAGACTGAAGTAAGTTCATGGACAGAGTCTGAAATGGATATTTATGCACCTTGGACCCAGGCTAAAACTCTAGCAGTAAATGCTTGGACTCAACCTGTGGCTGATACGGTCACACCATGGACTCTGGATGAACCTCTAGCAGTGAATCCCTGGACAGAGACTGAGACTGAGATTGTCACACAATGGGATCTGGATGATTCTCTAGCAGTCAATCCATGGACACAGACAATAACTGATATAGACAAACTGTGGACCAAGGTTGAATTTCCAACACTAAACTCTTGGATACAGTCTGAAGCTCATAGTGTGACACCATGGATTCAGACAGAGTCTCCAGCAACAAATCTATGGACACGGTCTGTTTCTGATATAATCAAATCATGGACCCCAGATGAATCTCCAGCTGTACATCCCTGGGCACAGGCTATAACTTCCATAATCACAACTTTGACCAAGGCTGTATTTCCAGCAGTAAATCTATGGACAAAGTCTGTAGCCGATAACATCACAGTATGGATCCAGGCTGAACTCACATCTTTATATCCATGGTTACTGCCTTTGCCTGACACAGTCACACCAGGGCCCCGGGCTGATTCTTCAGCAATAAATCTATGGTCAAAGCCTATGTCTGACATAGTCACTTTGTCATCTCAGGCTGAAATTCCACTAATAAATTTTTTGACATATCCTTTGCCTGATACAGTCATACGATGGCCCCACGAAGAATCTTCAGGAACAATTCACTGGACTGAGCTTGTACCTGATATGTTTACACTATGGACTCAGTCTGAATTTCTAACAGTAAAGTCCTGGATTCAGTCTTTATCTAATACACACATATCATGGACCCAGGATGACTCTCCAGTGGTAAAGTACTGGATAGAGGCAATATCTGATACAGTCACACTATGGAACCAGATATCACCTCCAGTAAATCTGTGGACACAATATGAAACTCATGTGTTCACACAGTGGCCCAGATCAGAATCCTCAAAAGTAAATCCCTGGACAAAGCTTGAAACAGATACTGTTACACTATGGAACCAGGCTAATTCTGAAATCCCCAAACCCTGGACACGTGCTTTGGTTGATACAGTGACACTGTTGACCAAGGGTGTTTATCTAGCAGTAATTCCATGGAGACAGACTGAAACTGCAACAGTCACAGCATGGACCCAGGGTCTATTGCCAGCAATAAATCCCTTACCAGTGGCTATAAGTGGTACAGTTGCACTGTGGAATATGGCTGAAACTCTGTCAGTAAAGCCATGGACACACTCTGCAACAGAAATAGTCAGAGAGTGGACTCAAAATGAATCTCCCGCAGTAAATCCATTGATACAACCTATATCTGATATAGTCATTCTTTTCACCCAGATTGTTAGTTCAGCAGGAAATCATTGGACAAGGTCTGAAGCTGATGCAGTCACACCATGGACCCAGGTAAAATCTCTAGGAATAAATGATTGGAGACAGGCAGTATCTGAAACAGTCACAACATGGGCCCAGGATGAATTTTCAGAAGTAAAACTTTGGGCACATTCAGAGGCTGGTACAGTCATACTGTGGCACCACACTCTATCTCCAGTAGTAATTCCCAGGATGGAGGCTCTAGCGGATACATTCATGGCATGGACACAGACAGAATCTCCAACAATAAACCCCTGGATACATCCTTTACCTGATAAAGTCACATTGTGGACTCAGACTGAATATTTCTCAGTAAATTCTTGGAACCAGGCTGAATATCGAAAATTAAATCCCAAGTTACAGTCTGAATTTCCAACAGTAAACTTCTGGACAGAGTCTGAGTCTTCAACCCCAAATCCCTGTATACAGACTGAGAATGGTATAGTAGTCAAACCGTGGAACCATGCTGAATCCCTAGCAGTAAATTCTTGGACATATCCTATAGCTGATACAGTCACACCATGTATACAGGCTGTATCTCCATCATTAAATTCCTTTTTAGAAACTAAAAATCATACAATAAAAGCATGGACTAATATGGAATCTTTACTAGTAAACCCCTGGACAAATGCTAAAGCTTCCATAGCTATAACCTGTACCCAAGATGAATATCCAGCAGTGAAACCCTGGACACAGCCTGTATCTGATACACTTATATCATGGACCCATGATGAATTTTCATCAGTAAATCCATGGACACAGACTGTAGCTTCCACATTGTTACCATTGACTCAGACTTTGGCTTCAGGTGAAAATACCTGGAAACAACCTGAATCATTTACCCCATGGACACAGTTTGGATATCCAGCTGTGAATCTATGGACAGATGCTATAGCTTATATGGTCACACTTTGGACCCCAGCTGACTATCTAGTAGTCAACACCTGGAGAGAAGCTGAAGCTTCTTCCACAGTCACATCTTGGAAACCAACTGACTCTCCTGTAAATTTACTCTCACCTGTAAATTCCTATATACAGAGTGAAACTTACATGAACACATTTGGGACCATGCTTAAAACTGAATTTAAGAAACCTTGGGCACAGCCTGAAGCTAATATATTTAGAGTTTCATTACAGCCTCAAATTGATACTCCCCACTCTACAATTCAAATTGAAAATCAAATGTCTCTTCTGTGGACACATCCTGAAATTGAAAACATCAATACATGGACCTTGCCTGAATTTGGGACACTGATGTCCTGGATAGTCCCTGTTCGACAAGCAGCCATATCATGGCCCAACCCTAAAGCTATAATTACCAGAACTTGGTTTAAAACTCAAACAAAAAGAATAAGACCTTGGATTCAGCCAGAATTTCAAACACTCAGTCCATTTGGAGATGATAGAGTTGAGTCTTGGAGCCAACATAAAACTGCTATAGTCATATCATGGGTCCAGTCTGAAACTGGTATATTCCACCCCTGGACCCAATCTGAGGTAGACACAGTGAGACCTTGGACCGTTTCTGAAGTTGATAAAGTAAAACTATGGACCCAAAGTGAAATAGGCATAGTCAACACCTGGACCCAACCTAAAACTAATACTATCAGACCTTTGGCCCAGGCTGAATCTCAAGCAGTTAGGTCCTGGTCTCAGTCTTTACCTAATATAGTCACAGGACTGACCCAGGATGAAATGCAAGCAAGAAAACCCCTGACTATCCCTGCCATTAGTATTTTCAGTCCTTGGTTCCATATTCAAAGTGATACAGTAATACAAGGGACTAAACTTGAACCTCAAACAGTCACTACATGGATCCAGCCAGAATGGCAAAGAATAAACCCCTGGAACCAGCCTAAAACTAATGTAGTCAGACCCTGGGCCCAGTCTGAAAGCGATTTTGCCCATCCCTGGATCCATGATGAAACCAATAAAGGCAGATTATGGACCCATTCTGAAATGGATAAAATAAAATCATGGGCTCAGCCTGAATCTCAAGTAATTATGACCTGGCCTAAAGCTGATACAATCACATTTTGGTCCCCAATTCAACATGATGCACTTTGGCACTGGACCAAACTTGATTCTCAAATGATACATTCCTGGACCCAGTCTGAAATTAGTATAACCAACCCTTGGCCTCTACATGAAATTGCTACAATCAGACCATGGAGTCAGTCTGAGATTGATCTCTGGATCCAGCCTGAAGCTTACAAAGTGATAGGATACTGGTTCCAGACTCAAATAAATTCAGTAAGACCCTGGAACCAAGCTGAAATGGAAACAATCCAAACTTGGACCCAGACTGTAAGACAAATAGTAAAACCCCCAAACTTGACTGAAGTTGATATAGTCACACCTGTGGTACAGACACAAAGTGATATATCTAGATCCTGGATTCAATCTGACACCAAGTCAGTCAGTCACTGGACCCAGACTGGAGCTGGTATAGTTCAGCCCTGGGGTCAGCAAAGAATTGCTACAAATCAACCCTTGACCTATCCAGAAACCCAACCAGTCAGACCCTGGATCAAGCTGGAAACTGACAAAGCTAAATCTTGGTTCCACATTCAGATGAATGAAGGCATACCATGGACCTCTTCAGAATCTCAAATATCTAGTTCTTGGATCCAGCCTGACATTGGTTTAGTTCATCCCTGGATTCAGCCTAAAACCCAAGCAGTCAGACTTTCTACTGAGTTTGAAACTTTCGTAGCCAGATCCTGGTCCCAAGTTGTATCTCACATATTCAAACCATTGATTTTGTTTGAAGGAAGAACACTCACATCCTGGACTCTGCCTGTAACCCAAGCAGTCAGACCCTGGACCCAGCTTGAAGCTGCAATTATTGCATCATTTGCTATTTCTAAACCTGACCACATTGTAACCTGGATCCAACATGGAACAGAAATAGTAAGGCCTGGAACCCATTATAAAGCTCATATAATTGCATCATTTACTCCTACTGAAGTTGAGTCACCTGCAGAAACCCTCTTAAGCACCCATTTTGGCTTCTCATCTAAATATGTACCTTTTTTACCAGTAGAAACTATTCCTTCTCCAGATCAGTATTTTATAGCTTTGTCACTTGAGATAGCTGCTACCGAAAGCCACGATAAAATCAATTCTCTTCAACCCACCCAGCTTACAAACACTTTGAGTCTGGGAAGAGTTGGTTACAAGAACTATGACAGCAAATCACAAATTATCAAGATGAAAGAAAGCCCTGATACTCTGTCTACCTCTCTTGTCTCTCCTTCCTCATTCTTTTCCTTTACTCCTTGTTTTCTCCCATATCCATGTACATTGTACCCTTCTTGTTCAGTCCTTTCTTCTTGCACATTCCCTTCATCTTGCATTTTTCCATCATGTTCCATTTTCTCTCCTGTGGACTTCTCTCCTCTTCTCCTGCCCTTAGCCTCTTCTGACAGTCCCCTCCAGGAACTCTCTTCCTCAAAATTTACTGAAGAGACCATTCTTTCTCAGACTCTTTCTTCCATGCATGCTACACCAGCAACACTTTTAACAAAACAATCTCCTGTGATGTCTGGATCTCAGTCTGCATGGAACCATCAACCTGAACAACCATCTCTCGGGCATTCAGAAATCAATGTTTCCCTGGCTGAGTGTCACCTAGGTTTGATCTGGAAAGAGAGTCTCCAGGCTTTCTGGCTCGTCAAGACTGCTGTTATTTCTCATGAAACCACAGGCAAGTTTAGTGTACTCATCTCTTTGAAGTGTGGATTACGCCCTGGCCTTGTCCCGCACTGTCCCAACTGCTGGGAGGCAGAAATGGGTGAATTCCCTTGGATGGTTTCCGTGCAACTCTCTTTCTCTCATTTCTGTGCTGGCTCTATACTGAATGAGCAGTGGATCCTTACTACAGCAAGATGTGCCAATTTCATAAAAAACTCAGAAGCTTTGGCTCTGGTCCAAGTTGGGCTTACTGATCTTCAAGACCCCCCTGCCCATGCTCAGACTATAGGAATACACCGTGCTATGCCTTACCTAGGCCCCAAGGGACCTCTAGGACCTGGGTTGATCTTCCTGAAGAAGCCACTACGTTTTCAACCCCTGGTACTTCCTATCTGCCTGGAGGAGAGTCTGGAACAACAGAAAAAAATACAACTGTATGATTGCTGGCTGCCCAGTTGGTCCCTCATGAGGGGAAGTCCTGGAATTCTTCAAAAAAGACATCTAAGCATTCTGCAAGTCAGCTCTTGTGCTCAATTTTGGCCCAAGCTGAATGAATTCACTTTCTGTGTGGAAGCCAAGAAAGCTATGGGGGAAGCTGGCTGTAAGGGTGACTTGGGGGCACCCCTTGTATGCCATCTACAGCAAAAGGACACATGGGTGCAGGTGGGAATCTTGAGTCACTTTGATGAACATTGCACAAAGCCCTACGTCTTCAGCCAAGTGAGCCCTTTCCTTTTCTGGCTCCAGGGAGTTACACGGCCCAGCCATGCACCCTGGTCCCAGCGAGGGCCGGTGACTACCTCTGGTTCCATCCCCCCTTCAGTCTCTACTTCTACGAATGCCTCGGTTTTTACCTCCACACCTGCTTCTATTCGGCCACACTTCATCTCTCTGCCACAGCCTCAGACTTTGGCAGATCGGATCTCTCTCCGATATGCCATGCCTTGGCAGGCTATGATCATCAGCTGTGGAAGTCAAATCTGCAGTGGTTCCATAATCAGCAGCTCTTGGGTTCTCACTGCTGCCCACTGTGTCAGAAACATGAATCCTGAAGATACAGCTGTGATACTGGGCCTGAGGCACCCTGGAGCTCCTCTGAGAGTTGTTAAGGTGTCTACCATTCTACTCCATGAGAGATTCCGGTTGGTGAGTGGGGCAGCAAGAAATGACCTAGCATTGTTGCTCCTTAAAGAGGCTCAGACTCCTCTTCAACTCTTAGCACCCTTGGGACATCTGAAGAATCTGAATAGCTCAGAATGCTGGCTCTCAGGGCCACGAATTCTTAAACCAGGAGAGACAGATGAGAATCCAGAAATGTTACAAATGCATGTGATGGAAGCTTCAAGCTGTGCCCACCTCTACCCTGACATAGGAAGTTCTATTGTTTGCTTTGTTACTCGGGTCAAAGGCTCTGATGCAGACATGGAGCCAGTGACTCCAGGAAGTGCTGTTATGTGCAAACTGAAATCTGGCATTGGAAGTTGGAGACAGATAGGCTTCACCAGTCTCAAAACGCTAGCTACCATAGTCAGTCCACACTTCTCCTGGATCTTATCTACTTCAGCAAAAGCAGAGCATCCCCTAAACCAAGCCCACACGCCTTGGATGGAAACCCCAAAGTCCTCCAGTCTCCTTAGGCAGGCAATAGCAGTGCCACTGTCTTCGGTAATGATTATGGTATCCCAGTCTTTTGTAGCCCAGTGTCTATACTAAGTACTAGTAATCTAAATACACTATGGTGAAACACCAGAAAACAAAGAAAAAAGATTTCTTTATTCCTTATAGTATTTCTCTCAATATTCCTTCAATAAAT

>Newt, Japanese Fire-bellied, Cynops pyrrhogaster (BK059520)

CGGCCTTCCTTCCCTCCAGCGCCGAGAACCAATGGCTGCTTCCTGGATGCAGCCCCAGCAGAGGCCCTGGGAGAAGGATGAGACTGCCGCTGCTCCTGCTGCACATCCTGGGACTGCAGCGCTGCGCGGGCTACTGTGGCATCCGTAACCACCGGGGCAGGGCGTACCCAGCAGCTTCGGACATCCCCTGGCTGGTCTCAGTGGCTGGAAACGGGCAGATCTGTGAAGGCGCTATCATCGACAACTGGTGGATAATAACAGCTGCCAGCTGCCTTGTCATGACGAAGCAGGGCCGAGTTTCAGGAAGCAGAGTCCTAAGTGGCGTGCCGGGCATGCAGGTGAACCGAGCCATCATCCACCTGGAATACACCTCCGGCCCTGGGGACACATCTATGTTTGACATCGGCCTCATTCTCCTGAGGGAGCCGCTGGCCTTCCACCAGAACCTCTGGCCAGCCTGCTGGCCTGCAGAGGACTACAACATGCTGGGAGCCACACACGCCTGCTGGATCTTGGGAGTGCGGGCCATTGATGAAGACCCACGGTACTGGAGTGGGGGTGTCCACAGGATCCAGGTGCAGTTGGTGGAGCCATCAGAATGCCTCTGGTACTGGCCTGACATAACTCGACAAGACCTGTGTGCCAGGAATAAAGCGAGGACGAAAGGACGCTGCCAGATCCGCCGTGGCAGCCCACTCGTTTGCTTTGACATTTTCCACACCAAATGGGTCCTGGTTGGTTTAGTAGGACGGGCGTTGAAAGACTGCCAAGTGCCTGCCCTCGCCACCAGAACATCAGTCTTCACGGAGTGGGTCACTCGAGAGACAAAGACAGCTGGACATCCATTCTACCCTACAACTTTCATGCTGACTTCTAAACACCGAACTCAGCGGAAATTCTCTGGTGCTGAGGAGAGGGTGGCACAGGGTGTGCATCTCTTTGCAGCTGTACGTTGTACATATGGAGACCGACCCTCCGAGGCTCGCCTTTGGTCGGGATGGCCTCTGGATGTTAACCAACTGCCCCGATCTAAGAGGACCAAGTTACACAGAGGCAGGCTCTTACACTCGCTTGTGAAACCTATTTTTAAGAAGCTCCATTTGTTAGCTGTCCACCTAGACTTTAGACGGTGGTTTTCAGCCATGCTCACTTCCCAGGGTAGCGCAAGGAGGCAAGGGATTCCTGTGGTCAATTATCTTCTGAATCAAAAGCCCACAAACACTACCACAGTGCTGCCCTTACCTCCAGACATATACATTACACCAGCGTCTGTTGAGGCAACACTACCTTCTCAAGTAGCACATTCTCGCTACTCTCCACCATCCGTGTGGGTAAAACCTACCAGACCCACGCGAGTCTCGCTTGTAAGAAGGAGGCGTTCTGCAGTGCTGAGCTTGGAAAGCCAGGTGGGCAGCACCCAGCCAAGGTTGTCAGCACAAGCACCTCAGAATCTACTCACCCTTCCAACCGCGACTTATTTTTTATCCACAGATTCTAACACAATACCTACAATAAGCCTTGCCACTCTCATGCCACGTTCGGCAACAGGCGCAAGCAGCAGTGTAGCTGTTCTGCTGAACCCCACTTATTTGGTACAGCTCACCAAATCCTCAATACTGGGCAAAGAAATAACATTCTTTAACCATCCCACATCTGAAATCACAGCTTCTAGCACCACCAACAGCAGCACACAGAACACCACCACCACCATAAGCACCACAGTTTCCACTGTAAACACATCCACCACCACCACCACAATCAAAATGTCAACCCAAATACAATCAGTTTGGATTAAGACTCGATGGCCAATTCTTTCTACCTTAACAGTTGGCACACCCATCTTGGCAATCTTGCCAACATTCTTAAATAATCGATCTGCTTTGGTATTGTCAACCATCCAACCAGCTTCAGGCAACTCACTGCTTGCGTCAGCAAAACAACCTTCTCAGGTCCTGCCAGCGTCTGCATTACTGGCACACTCTGCAGGCCCTCGACCGTCCCCTAGATTGCTGCCCTACACCAAGCCACCTTCCACTAGCAAGCAAATCCCTCCATATGTGTTCCCCGATCCTGCAATGCCGCCTTTCATCATCATGCCGCCTAATGCAAACCCTCCATATATCACTGGTATGCCACCTTCTGCAAATCCTCTATTTGGCAAAGACATGCCACTTCTTGTATATCCTCCATTTGGCAAAAACATGCCACTTTTTACGAACCCTTTATTTGGCAAACGTTTACCATACCCTGTATATCCTCCGTTCGGTAGAGGCATGCCACCTCCTGTACACCCTCTGTTTGACCAAGGGATGCCACCTCAAGCATATGTTCCATTTGACCGAGGCATGTCACCTCCAGTACCCTCTCCATCAGACCAGGGTATGCCCTCTCTTGTATACCATCTACCAGACCAAGGCATGCAGCTTCTTGTACATCCTTCACCTGACCACCACATACCACCTCCTCCATCTGACCGAGGCATGCCACTTCCTGTATACCATCCACCGGACCAAGGCATTCAGCTTCTTGTACATCCTTCACCTGACCACCACATACCACCTCCTCCATCTGATCGAGGCATGCCACCTCCTGTATACCATCCACCAGACCAAGGCATGCAGTTTCTCGTACAACCTTCACCTGACTACCGCATACCTCCTCCTTCATCTGACCGAGGCATGCCACCTCCTGTATACCCTCTATTTGACAGGGGCATGCCGCCTCCTCTTTACCCTCCGTTTGATAGAGCTGTGCCACTTTCTGTATTCCCTCCACATGTCCACCACATACCTCCTCCTATATACCCTTTATTTGACAGAGGTATGCCACCTTCTGCATACCCTCCACATGACCACCACATACCACCTTCTATCTACCCTCCATTTGACAGAAGCTCGCCACTTCCTGTACACCCTCCTCTTGAAAGAGGCGTGCCACCTCCCATAAAAGCTCCATACGGCCCTGAAACGCTAACTACTGTATTCCATCCAAATGACCGCAGCATTCCACCTCCTACCTATTCTCAATCCGAGGGAAGCACACAACCATCTGCCTACTTGCAGCATGTCCAAACTGTACCTTTCCCTCCAAATAGACCGTTAATGCCATTTCCCTCATATATGATGCCTGGGCCATTCCTGCCCCTTATGAATGGTGTGTCACAGCTGCCCTATCTCTTGCCACTGCAATCATATCTGAGTGTCCCAGTGCTGGCCCCCACTTCTGTGTCCCCAGCAGCATCCTCTTATGTCGTAGGACCTCAGACTAAGAGGTTTTTTCGAGGCACCAAGAGATTGGTTGTTGCACCTGCAACTGTAGCTGTCTCTGCAAATCCATCTCTGCCCATAACATGGAGCAGACCTCCCTTGATCTTGGCCACCCTTCCCATGACCTTGGGCAGACGTTCTACAACCAGTTCTATCATGACCTCTGCCATCCCTCCTGTGACATGGGTCATTCCTCCTGCTACCTGGCCAAACCCTATCCCTTTTGTGAACTGGGCCAACCCTACTGCATTTTCAGTCAGCTCTCTTGCAACCTTAGCCAGCCCTTCCGTGACAGGGACTATCCCTTTGACGACCAGGCCTCCGGAGACCTTAGCTAGTGCCCCCTTGACCTGGATCATCCCACCTATGAATCTGGCCAGCCCCGCTGGTACCGGGGCAAGCCAGCCCCTAATGTGGACAATACCCCTCATAACCTGGAAAAGCCCTCCTGTAATCATGGATAGCACTCCTGCAATGCAAGTCAGCCCCCCTACAAATCAAACTATAACAATGGCAAACATGGCCAGACCTCTCCCTAACATGATCAGACTCCCCACAATCTTGGTCAACCCTCCTGTGATCAGAGTGGGCAATCCTTCCATTTCTGCCAGTGCACCAGAGGTCAATCCAAGCCCCCTCGTGAATTGGACACCTTCCCCAATGAGCATTGCTACCACCTGGATCAACATGCCCACAACTGAGACCAGCACCACCACAACTTTAACCAGCTCACCTGTGACCAGCATCCCCACAAACGTCCCCATCTTTGAAGTGACCAGTATCCCCACGACCCAGTCCCCCTCTGGTCAGACCAGCATCATCAATACAGAGAGCCGTTCAGATTCAACAGAGTTCCCGACTATCTTGACAAACTCTGATGCACCAGGGATATCCACGATCTGGATTACTTCTGATTCATCAGGAGTTCCCATCATCTGGGCTGGATCTGATGCATCAGGAATTCCCATCTTCTGGACCAGCTCTGATTCAACAGGAATTTCCACAATCTGGACCATCTCAGAAACAACCGGAATCCCCTCAGTCTGGACCACCTCTAATTCTACAGGAATCACCACCATCTGGACTAGCTCAGAAACAACCAGCGTCCCATTGGTCTCGACCAACTCTGAAACAACAGGCGTCTCAACAATCCCAACCAGCTCTGAAACAACAGAAATCCCCATGACCTCGACCTCCTCCAGTTCAGCTGGAAAGCCCACCATTCAGATCAGCTCTGAATCAACCAGAATCCCAACATCCTGGACCAACTCTGCCCCATCCAAGACTTATAGTGCAACTGACATGGCCCACAGTGATGATATGACAGTGATACTGTCTCACTGTGGGGTGTCCCTGGAGTGGAACATCTACACACAAGCCTTCCACCTCTCCCGTAGCACCATGCAAGCTCCAGATGATGTCATGGCGTGTGGTCAGCGGCCTTTCTATATCCCCAGTGACCCTAAAAATCTGGAGGCCCAGACTGGAGAATTCCCCTGGGTGGTATCCCTGAAGCTGTCTGTCCATCACTTCTGTGCTGGCTCCATCCTGAGCCGATGGTGGATCCTGACCACGGCCAACTGTGCCAACATCATAAAGAACGAGGAGTCGTCGGTCATGGTGCACGCGGGGATCCTGAACCTGCAGTCGGAGACCATGTCGGCGCGTGTGCAGATGGTGCTGACCCATCAGGACTACCAAGAGAACCACGAGTCACACAACCTGGGCCTCGTGCTGCTGCAGGAGCCACTGCACATGCGCTCACTGTCCTCTGCCATCTGCGTGGCTGAGAACATGTCCCAGGAGAAGCAGCTCAACCTTACCAACTGCTGGCTCCCCGGCTGGACTGTCCTGCAAGGTGGCCCCACAGTCATGGTGAAGCACCACATGGACCCGCTGCTCCGGACTGCCTGCAACAGTTTCTACAGTGGCTTCTCGGAGTTCATCTTCTGCATGACGATTGACGAGCCTCACGACACGGCATGCAAGGCAGACATTGGGTCACCGCTGGTGTGTGAAGACCCCTTCAGCAAGTCCTGGCTGCAGATTGGCATCCTGAGCGACTTTGATGTGAACTGCCGGAAGCCGTTCGTGTTCATGAAACTCTCACACTACATGAGCTGGGTGGAGAGAACCACCCGACAGGCGGGCAAGCCCTACACACCACCGAATGCTGCATGGATCAACCGCCTCCGCAAGCCTGTTCCCAAGGAGTGGGACCAGCCCAAAGCACCCTACAACCCCACTCTGAGCGCGGAGGCCTCCGAGGACCCCAACTCGCTGGATGTGAAGGCCCCCTGGCAGACCCTTGTTGTCAACTGCAACACGAGCCCCTGCAGTGGCTCCATCATCAGCGACTACTGGATCCTGACGTCAGCTAATTGTGTGCTGGACATTGAGCCTGATGACATCATGGTCTACGTTGGCCTCCGGGGTGGCGAGCACATACCTGAGGATGTCAGGGCGGACCGCATCTTCCCACACGAGCTGTACAGGCCTATGTTGAGCAGCGCCCACAACATAGCGCTCATCCTGCTGAGGGGTCCCATCATCTACAGGGATGACATTGGCGTCCTGTCCCTCTGGATGAATCTGCGCAGCAACATGTCCACTGTGGAGAACTGCGGACTGACTGGAATACGCTCCCTGCAACCAGTTGATGACGAGTCTGAGACAGGGGAGGCAGATGTGGCGGAAACTAACCTTAACGTGCAAAGTGCTACCCACTGCCCCCAGGAGCCCTCAGTGCAGAACCAGACCTCTTTCTGCATCACCGAGCCAAGCCCCGGAGTCCAGCTGGCGAAGGTCCTCGAAGGAAGCCCCATACTGTGCCAAGACCGGATCACCGCCAAGTGGTCTCAAGTGGGCACCTTGTCACATATTCTGCCAGGTGTGCCTGTGTCAGCGATGTGTACCACGGTCACATCCTATGCACACTGGATCAACAAGACATCCAAAAATGCCAAGAGACCTCTGAACTTGCCACCACTGAGTTCCGCCCGGCCCACTCGGGTCTGCTCTGCAGACCTGCCACATGCCCTCATGCTCCCGGCCCTGGCCCTCCTCTCCTTAGCGACCCAATACTCCTATTAACGCACATTTATTTCTGAAATCTTAATATACTCCTTAGTTCTGCCCTCTCCTGCGCGTGGTGTGTCGTGGGTGGATAGTAATGCATCCATGTAGGGTGGCATGGGCTGGCAGGCTCCTGCGCCCCACTCGAATGCAGGCTCTGTCCCATCATCGCGGCTGAGGGCTCACTGCGCAGGCCATTTGGGCAACGACCACTGGCAGCTAAAGTTAAAGTCTGGCACCTCATTGATAGAAGACTATGGAGAGGAAGCTGGGTGCCAGATCCGGTCCTGGGGGACCTCTTCTCACCCTCACCTGACACTCTTTTCCGTTGTCAGATCCATGCCCTTTGTTCCTGAGATCTTCTCCTGCCCCTTCTTTCATGCCCAGTGCCACCTGGCAGCTCTCCCCCCATGTATCCCAGTCATCCTAGCCACTTGAACCAGAT

>Newt, Iberian ribbed, Pleurodeles waltl (BK059521)

TGACCTCTTGTGGTCTTGTGTAGATGTTCTCAGTCGGGGGAGGGCGGCCTCTCTTCCGGAGTGTGACGTCACGGTGATGTCTGCAGGACCTGTGTGACCTCACAATGGGGCGGTGACGTCCCTCTGGGAGGCGCCGAGGACCAATGGCTGCTTCCTGGATGCAGCCCCAGCAGAGGCCCTGGCCGCCCCCTTCTTCCCTGGAGGAGGATGAGACTGATGCTGCTGCTGCTCATCCTGGGACTGCACCGCTGCCAGGCCTACTGCGGCATCCGTAACCACCGGGGAAGGGCTTTCTCCGGCAGGAAGTACCCAGCAGCTTCGGACATCCCCTGGCTGGTCTCAGTGGCTGGAAACGGGCAGATCTGTGAAGGCTCCATCATTGACAAGTGGTGGATAATAACAGCTGCCAGCTGCCTTGTCATGACGAAGCAGAGTCGAGTGTCGGGAAGCAGAATCCTAAGTGGCGTGCCGGGCATGCAGGTGGACCGCGCCATCATCCACCTGGAATACACCTCCGGCCCTGGGGACAGAGCATCAATGTTTGACATTGGCCTCATTCTTCTGAGGGAGCCGCTGGCCTTCCACCAGAACTTATGGCCAGCCTGTTGGCCTGCAGATGACCACAACCTGCTGGGAGCCACACACACCTGCTGGATCCTGGGAGTACAGGCCCTAGACGAAGAGCCATGGCGCTGGAGTGGGGGTGTCAGCCGCATCCAGGTGCAGCTGGTGGAGCCGTCGGAATGCCTCTGGTACTGGCCTGACATAACTCAACAGGACCTGTGTGCCAGGAATAAAGCAACGACTAGAGGGCACTGCCAGATCCGTCGTGGAAGCCCACTCGTTTGCTTTGACATTTTCCACACCAAATGGTTCTTAGTTGGTTTAGTAGGGCGGGCGTTGAAAGACTGCCACGGGCCTGCCCTAGCCACCAGAGCATCAACCTTCACAGAGTGGATCACCCGAGAGACAAAAACAGCAGGACATCCATTCTACCCTACAACTCTTATGCTGGCTTCTAAACACCGAACTCAGCTGAAGTTCTCTGGTGCTGAGGAATGGGTGTCACAGGGTGTGAATCTCTTTTCAGCTGCACGTTGTACATATCCTGGAAGCAGATCCTGCAAGGCGCGCCTTTGGTCGGGATGGCCTCTGGATGGTGACCAACTGCCCCGATCTAAGAGGACCAAGTTACAAAGAGGCAGACTCTTGAGCCCACTTATGAAACTTATTTTTAACAAGCTCCGTTTGTTAGCTGTCCGCCTAGACTTCAGACGGTACAATTCAGCCATGTTCACTTCTGAGGGTAGTGCAAGGCGGCAAGGGATTCCTGTGGTCAATTCTCTGCTGAACCAAAAGACCAGAAAAACGGCCACAGTGCTGCCATTATCTACACACACACACATTACTCCAGCGCCCATTGAGGCAACCCCGCCTTCTAAAGTACCACGTTCTCCCCACCCTCCACCATCTGTGTGGGTAAAACCTTCCAGGTTCATGCGAGTCTCGCTTCTTAGAAGGAGGCGTTCTGCAGTGCTAACCTTTGAAAGCCAGGTGGGCAGCACCAAGCAAAGGATACCACCACAAACATCTCAGAATCTACACACCCTTCCAGCCAGGACTTACTTTTTATCCACAGAATCTAACACGATCTCTACGATAAGCCTTTCCACTCTAATGCCACCTTCTGCGGCAGGCACAAGCAGCAGTGCAAGTGTTCTGCTGAACCCCACTCTTTCAGAAGAGCTAATCAAATCTTCAGTACTGGGTGAAAAAATAACATTCATGAGCAATCCCACATCTGAAATCACAGCTGCTAGCACCACCAACAGCAGCACACACAGCCCCACCACCATCAGCACCACAGTTTCCACCAAAACCACCTCCATCAAAACGTCACCCCAAATACAATCAGTTTGGATTAAGACTCGATGGCCAATTCTTTCTACCTTAACAGTTGGCACCCCCATCTTGGCAAACTTGCCAACCTTTTTATATAATCGATCTGCTCTGGTAGTGTCAACCATCCGACCAGCCTCCAGCAACCAGCTGCTTGCTTCAGCAAAGCAACCTTCCCAGGTCTTACCAGCGTCTGCATTACTGGCACATTCTGCAGGCCCTCCGCCTTCTCCTAGATTGCTGCCCTACACCAAGCCACCTTCCACTAGCATGCACATCCCTCCATATGTGTTGCCCAATCCTGTAATGCCACCATTCATCATCATGCCACCTAATGCATACCCCCCGTATATCACTGGCATGCCACCTTCTGCATCTCCTCCATTTGGCAGAAACATGCCTCTTCTTGTATATTCTCCGTTTGGCAAAAACAAGCCACTTTTTACAAACCCTTTGTCAGGCAAAAGTTTACCACACCCCGGATATCTTGAGTATGGTAGAGCCATCCCACCTCCTATACACCCTCCGTCTGACCAAAGGATGCCACCTCCCGAAGTCTTTCTATCTGACCAAGGCATGCCGCCTCCAGGATATATTCCATCTGACCGAGGCATGTCACCTTTAGCACACTCGCCATCTGACCAAGGTATGCCACCACCAGTGCACTCACCATCTGACCAAGGCATGTCACCTCCAGGAAACCATCCACCAGACCGAGGCATACAAACTCTTGTACACCTTTCACTTGACCACCACATACAACCTCCTTCATCTGACCGAGGCATGCCACCTCCTGTATACCCTCCACCAGACCTAGGAATACAACCTCTTGTACACCCGTCACCTGACCACCACATACCACCATCTTCATCTGAGCAAGGCATGCCACCTCCTGTATACCCTCCACCAGACCGAGGAATACAACCTCTTGTACACCCGTCACCTGACCACCACATACCACCATCTTCATCTGAGCAAGGCATGCCACCTCCTGTATACCCTCCACCAGACAGAGGAATACAACCTCTTGTACACCCGTCACCTGACCACCACATACCACCATCTTCATATGAGCAAGGCATGCCACCTCCTGAATACCCTCCATCTGACAGAGGCATGCAATTTCCTGTATACCTTCCATTAGACAGAGGTGTGCCACTTCCTGTATACCCTCAATTAGACAGAGGTGTGCCACATCCTGTATACCCTCCATTAGACAGAGGTGTGCCACTTCCTGTATACCCTCAATTAGACAGAGGTGTGCCACATCCTGTATACCCTCCATTAGACAGAGGTGTGCCACTTCCTGTATACCCTCCATTAGACATAGGTGTGCCACATCCTGTATACCCTCCATTCGACAGAGGTGTGCCACTTGCTGTGGACTCTCCACATTTCCACCACATACCTTCTCCTACATACCCTCCATTTGACAGAAGCATGCCACTTCCAGTACACCCTCCTCTTGAAAAAGGCATGCCACCTCCTGTGAAAGCTCCATATGGCTCTGGAATACCAAATATTGTATTCCATCCAGATGACCATGGTATTCCGCCTCCTGGCTATTCTCAGTTTGAGGAAAACACACCTCCATCAGCCTACTTGCAGCATGTCCAAACTATAACTTTTCCAGCAAACAGACAGTCTGTATCATTTGCCTCATATATGATGCCTGGGTCTTTCCAGCCCCTTATGTACGAAGTGTCACAGCTGCCCTATCTCAAGCCGCTGCAATCATATCTGAGGGTCCCAGGAGTGGGCCCGCCTTCTGTATCCCCAGCAGCATCCCCTTCTGGTGTAGGACCTCAGACTAACAGGTTTTTTCGAGGTGCCAAGAGATCTGTTATTGCAACTGCAACTGTAGCTGCCTCTGCAAATCCGTCTTCGCCCATGACCTGGACCAGACCTCCCTTGATCCTGGCCAGCCTACCGATGACCCTGGGCAGACGTTCTACAACCAGTTCTGCCACGATCTCTGCCATCCCTCCGGTGACATGGCTCATTCCTCCTGCTACCTGGCCAAACCCTATCCCTTTTTTTAACTGGGCCAACCCTACTGCATTTTCAGTCAGCCCTCCTGCAACCTTAGCTAGCCCTTCTGTGACTATCCCTTTGACAACCTGGGCTGGTCTGCCGGTAACCTCGACCAGGCCTACTGAGAGCTTAGCTAGTGCGCCCTTGACCTGGATCATCCCTCCTGTGACTTTGGCCAACCCAGCTGCGACCTGGGCAGGCCATCCCATAATGTGGACATTTCCTCTCATCACCTGGAAAAGCCCTTCTGTAATCTTGGGCGGCACTCCTGGGACCCAAGCAAGCCTTCCTCCGAATCAAACTAGAACTTTGGCCAACATGGCCAGCCCTCCCCCTAACATGGTCAGATTCCCTGCAATATTGGTCAGTCCTCCTGTGATCACAGTGGGCAATCCTTACACTTTTGACAGTGCCACAGAGGTCAGTCCAAGCCCCACTGTGAATAGCACACCCTCTCCAATGCGCATTGCTACCACCTGGATCAATGTGCCCACAACTGAGACCAGCACCACCACAACTCTACCCAGCTCGGCTGTGACCAGCATCCCCACAATCTTAACCGTTTCTGAAGAGACCAGCATCCCCACAACCCAGCCCTCCTCTGGTCCAACCAGCGGCATTGTCACAGGGATCAATTCAGATTCAACAGAATTCCCGACAGTCTTGACAAACTCTGATTCACCAGGGATGTCCACAATCTGGATTAGCTCCGATTCATCAGGATTTCCCGTCCTCTGGGCTGGCTCTGATTCATCAGGAGTGTCCACCATCTGGACCAGCTCAGAAACAACTGCAGTCCCCTCGAGCTGGACCAGCTCTGAACCTACAGGAATCCCCACTGCCTTGACCAGATTGGAAACAACCAGCATCCCATGGATCTCAACCAACCCTGCTTCAACAGACATCTCAACAATGCTGACCAGCCCTGAAACGACAGGAATCCCCGAGATCTCAACCTCCTCCGATTCAACTGTGAGGCCCACCACCCAGATCACCTCTGAATCAACCAGAATACCAACATCCTGGACCAGATTTGCCCCATCCAAGACCTATAGTGCAACTGACATGGCCCACGGTGATGATATGACAGTGATCCTGTCTCACTGTGGGGTGTCCCTGGAGTGGAACATCTATACACAAGCCTTCCACCTCTCTCGCAGTACCTTGCAAGCTCCGGATGATGTCATGGCGTGTGGCCAGCGGCCTTTCTATATCCTCAGTGACCCTGACAGTCTGGAGGCCCAGGCTGGAGAATTCCCCTGGGTGGTGTCCCTGAAGCTGTCCGTCCACCACTTCTGCGCAGGATCCATCCTGAGCCGATGGTGGATCCTGACTACGGCTAACTGTGCCAACATCATAAAGAACGAGGAGTCATCGGTCATGGTGCACGCTGGGATCCTGAACCTGCAGTCCGAGACCATGTCGGCGCGGGTGCAGATGGTGCTGACCCACCAGGACTACCAGGAGAACCACGAGTCACACAACCTGGGCCTCGTGCTGCTGCAGGAGCCACTGCACATGCGGCCACTGTCCTCTGCCATCTGCGTGGCTGAGAACATGTCCCAGGAGAAGCAGCTCAACCTGACCAACTGCTGGCTGCCTGGCTGGACCGTCCTGCAAGGTGGCCCCACAGTCATGGTGAAGCACCGCATGGACCCCCTACTCCGGACCGCCTGCAACAGCTTCTACAGCGGCTTCTCGGAGTTCATCTTCTGCATGACGATCGATGAGCCTCACGACACCGCATGCAAGGCAGACATCGGGTCACCGCTGGTGTGTGAAGACCCCTACAGCAAGTCCTGGCTACAGATTGGCGTCCTGAGTGACTTTGACGTGAATTGCCGGAAGCCTTTTGTGTTCATGAAGCTCTCACACTACATGACCTGGGTGGAGAGAACCACCCGACAGGCGGGCAAGCCTTACATACCACCGGATGCTCCCTGGATCAGTCCCCTCCCCAAGCCTCTTCCCAAGGAATGGGACCTGCCTAAAGCTCCCTACAATCCCACTCTGAGCGTGGAGGCCTCCGAGGACCCTAACTCACTGGATGTGAAGGCGCCCTGGCAGTCCCTGGTGGTCACCTGCGTGAGGAACCCCTGCAGCGGCTCCATCGTCAGCGAGTACTGGATACTGACCTCGGCTGCTTGTGTGCTGGACATCGAGCCAGATGACATCATGGTCTACGTTGGCCTCAGGGGAGGCGACCACATACCTGAGGATGTGAGGGCAGACCGCATCTTTCCGCATGAGCTGTACAGGCCCAAGTCGAGCAGCGCCCACAACATAGCGCTCATCCTGCTGAGGGGCCCCATCATATTCCGTGATGACATCGGCATCCTGTCCCTCTGGATGAACCTCCGCAGCAACATGTCCACCGTGGAAAACTGCGGATTGACAGGAATGCGCTCCCTGCAACCAGTTGATGATGAGTCGGAGACAGGGGCAGCGGATGTGGCGGAAACTAACCTTAATGTGCAGAGTGCCACCCACTGCCCCCAAGAGCCATCAGTGCAAAACCGCACTTCCTTCTGCATCACTGAGCCAAGCCCGGGAGTGCAGCTGGCTAAGGTCCTTGAGGGAAGCCCTATTCTGTGCCAGGACCGGGTCACCGCAAAGTGGTCCCAAGTGGGCACCTTGGCGCATATTCTGCCAGGCGTGCCCGTGTCAGCAATGTGTACCACGGTCACTGCCTATGCCCACTGGATCAACAAGACGTCCAGAAGTGCCAGAAGGCCTCTGAACTTGCCACCGCTGAGTTCAGCCCGGCCCACCCTGGCGCGCTCTGCAGACCTCCCAGATGCCCTCTTGCTTCTGGCCCTGGCCCTCCTCTCTTTAGCAACCCAAGACTCATGTTAACACATTTATTTTCATAACCTTAAAATACTCCCTAGTTCTGCCCTCTCTGGTCCATGGCGTGTCTTGGGTGGAAAGTAGTGCATCCACTTGGGGTGGCATGGGCTGGCAGGCACCTGTGCCCCCCGAATGCAGGCTCGGTCCCATTGTCGAGGCTGAGGACCCATTGCGCGGGTCCTTCGGACAACAGCAACTGGAAGCTAAAGTTGAAGTCTGGTACATCATTAACAGAAG

>Cactus mouse, Peromyscus eremicus (BK059443)

CATTAATGCAAACTGATGTGAAGGCTATACCTTTCACAGCTATGGCATGGACAAAAACTGAGCCTCTGGGAAATGAGATGAGGTCAGAAGTTTTAACAATCAATGAGACATCATTGAACTCAAAGGAATTAACACCTGTACAATTTTATATACATACTGGTATTGATAAAGGTACATTTATAACAGTGACTGAAATTGATGCAAAGAATGTTTTAACAGTGCCTAGTGTTAATATTTTCAGAATTTCAATGCAAGCTCAAACTTATGTTATTCAACATTCAACTCAAACTGGACAAACATCTGTTCTGTTGACCCCTACAGAAACTATAGACAGAAATGCATGGTCGTTTTCTGAACTAGGATCACTCTTATCCTGGAGAATGCCTGTTCCTTTAGAAGATGATCACTGGCATCACCATGAATATTACACCAGCAGATATTGGTTTAAAACTAAAGCTGAAAATATACGACCATGGACACAGCCATATTTTCAAACAATCAATGTCTATACTCCATTAAGGGCTAGCAAAATAGAGTCCTGGGTCCAACATAAAATGGGTATAAAGTCATGGGTGAATTCTGCTACTGGTATATTTAATGACTGGACCCAGTCTGAAACAGAAAGTATAATACTTGGGAATGTTCCTAAAAATAATACAATAAAACCATCATTCCAAATGGAGGCTAGCACTAATCATATCTGGTTCCAACTGATAACTAATACAATAAGAGTATTGCCTCAGGTTGAATTTCAAGTAATTAGTTTCCTGACTCAATCTGTCTCTGATTCACTATTATCACAGTCTGAAAAAGAACCAGAAACATACTTGTACATGTCTGATAATAATGCTGTTTCATCTTGGCTCCCAATTCAAAATAGTTTAAGACAAAGAAATAAACTTGAACCTGAAACTGTTGCCCCATGGGTCCAGCAAGAATGGCATATAGCTCATCCCTGGAATAGACTTGAAACAAAGGTACTAAAAACTGGGTATAATTTTGAAGCAGATACTTCTCAAATACTCTTGACTTATTCTAAATCTGATGAAATTAAACTTTGGACTCAACTTGAATCTCCGCTGGTAAGGAGGTGGCCTGAAGATGCTATTGTCACACTTTGGCCTCTGACTAAAAGTGATGTAATTATGCCATGGTCACAACTGGAATACAAAACTATACAATATTTGGCACAGCATAATTTTGATAGAATCAACCCTTGGATTCAACATAAAGTATCTACTGAAACTCTAGAAATCATTCCCTGGACTCATTCTGATATAGTATTATCACATTCATTACATACTCAAATTGATTACACCCCTTGGCCACTGACTCATACTGATGCAACTAGGCTATGGTCAGAAGTGGAATATGAACCTACACAATCTTTGGCACAGACTAATGCTGGTTTAATCAATGCCTGGATTCATCATAACGATTGTACTGAAACTCTAGATGTCAATTCCTGCATTTATTCTCATAGACTAATATCATATTCATTACAGACTCAAATAGATTCATTTAGATTATGGAACCAGCTTCAATCTATGACAGCCCAAACTTGGAATCCAACTGTAGAACAAGTAATAAAAACAGAAGCTCCGACTGCAGTTGTTACAGTTTCACCATCGTTCCAAGTACAAGGAACACTTTTAAGAGATGAACCTATTCTGGTGTCTCCATCTAAATATGTACCTCAAGATAAGCATTCACTGGAACCATTATCTCTTAGGCCATCAAAACTCAATGTTTCCTTGGCTGAATGTCACCTAAGTATGATATGGAAAGATAACCTCCAAGCTCTATGGATCTACAAGACAGCTGTTGTTTCTCATGAAACCACAGAATGTGGTTTACGCCCTGGCCTTGTTCCTTACTGTCCCAACTGCTGGGAGGCTGAAATAGGTGAATTCCCCTGGATGGTTTCTGTGCAGCTTTCTTACTCCCACTTCTGTGCTGGCTCTATACTTAATGAAAAGTGGATCCTCACTTCTGCTCGATGTGCCAATTTCATAAAACGTTCAGAATCTCTGGCTTCAGTCCAAGTGGGGCTACTTGATCTTCAGGACCCTACGCAAGGTGAAACTGTGGGTATCCATCGTTCTATGCCATACATAGGTCCCAGGGGACCTTTAGGACCAGGCCTGATCTTCCTGAAGGAGCCACTGCATTTTCAACCTTTGGTGCTGCCTATTTGCTTGGAAGAAAGTCAAGAGCAAGAAACACATATACAACTATATGACTGCTGGTTACCAAGCTGGTCCCTTATGAGAGGAAGTCCTGGTATCCTACAAAAAAGGCACCTCAGCATCATGCAAGTCAGCACCTGTGCCAAATTTTGGCCTCAGCTAAATGACTTCACTTTCTGTGTGGAGGCCAAGAAAGCTATGGGGGAATCTGGCTGTAAGGGTGATCTTGGGGCACCTTTGGTATGTCATCTAAAACAAAAAGACACATGGGTGCAGGTGGGAATTTTAATTCACTTCGATGAACACTGCAAAAAGCCCTATGTTTTCAGCGAAGTTAGCCCTTTTGTTTTCTGGCTCCAGAGAGTTACACGGCCCAGCCATGCACCCTGGTCCTTTCAAAGACCAGTGACTACCTCTCTTTTCAACTCCCTGTCAATCTCTACCCATAGGAGAGCCCCAATTTTTAGTTCTCCAACTGCTGTTATTCATCCACACTTCATCTCTCTGCCACACCCTCAAGCTTTGGCAGATCATATTTCTCTTCAATATACTATGCCTTGGCAAGCTATGATCTTCAGTTGTGGCAATCAGATCTGCAGTGGCTCCATTATTAGTAGCTACTGGGTTCTTACTGCTGCCCATTGTGTTAGAAACATGAATCCTGAAGACACTGTTGTGATACTGGGCCTTAGGCATCCTGGAACACCTCTGAGAGTTGTTAAGGTGACTGCTATTTTACTGCATGAAAGATTCCGGTTAGTGAGTCAGACTGCAAGAAATGATCTAGCTCTCGTGCTTCTTCAAGAAGTCCAGAGTTCTATACACATAGTGGCACCTTTAGGCAATGTGAAGAATCTAAACACTTCAGAATGCTGGCTTTCTGGACCACAAATTCTTAAACAAGGAGATATACTTGAAAATCCAGAAATGTTACAGATACAAGTGATGGGAGCTTCCAGTTGTGCCTATCTCTATCCAGATATAGGAAGTTCTACTGTTTGCTACATTGCACAGGCCAGGGGTCCCGAAATAAACATGGAGTCAGTGAGTCCCGGAAGTGCTGTTATGTGCAGACCACTATCAGGAAATGGCAAATGGACACAAATAGGCTTCACTAGTCTCAAGCATCTAGCCACCATAGTAAGCCCACACATTTCCTGGATCTTGTCCTCATCAGCAAAGGCAGGTTATCCCTTAAGTCATGTCCTCTCTCCTTGGGTAGAAAATCCCAATTCCTCCAGTTTTGTAAAATACCCAGATTTATTGCTACTTTCCATTGTAATGGCTATTGCACTGAAGATTGTTTTCATTTTGTAGTGTACTAACTACATCAAGTTGTACAAACCTGCTGAACTAAGATATCAGAAAAATTAGAAAAAAATCCTGATGTATGCCTTttagctcttatctacatattgttttaataaagagtatttccctaaacaatgatattttatg

>Pig, Sus scrofa (MK393871)

ctcagagaaaaatcttggctgcagtctgattttgaaaaattctcaccttCAACCCAGGTTCAATTTGTATTGGTAAAACCCTGGACACAGTCTGAAAATGATACACTCATGCCATGGACCCAGGTTCAACCTTCTGCAGTAAATGTCTGGACAAAGGCTACAGCTTTCACAGTCATACCATGGACTCAAGATGAATTTTTTGCAGAAAAGTCCTGGACAGAGACTGTGGTTTCCATAGTCACACTGTGGATTAAGGCTACATCTCCAGAAGTAAATACCTGGACAGAGGTTTCTCTTTCCACAGTCACACCAGGGTCTCAAACTGGATCTCCAGAGGTAAAACCCTGGGCAGAGGCTCTAACTTCCACAGTTATACCCACACTACTGCCTCAGGATGAAATTTCTGCAACAAAATCCTGGACAAAGAATGTGGGTGCCAGAGTCACACTATGGACTCAGCCTGAATCTCCATCTGTCCATCCCTGGGCACAGACTGTAGCTTCCACAGTCACACTCACACCATGGACTCAGGATGAATTTTCTGTAACAAAATTCTGGACAGAGACTTTTTTTTCCACAGTTCCACAGTGGACTCAGGCTCAATCTCCCTTAAATCCTTTGACAGAGACTATAGATGCCATAGTCACACCATGGATTCAGGCTGAATCTCCAGGAGTAAATCATTTGACAGAGGCTGTTTCTTCCATAGTCACACCTACACCATGGACTCAGGCTAAAGATTCAGCAATAAAACCCTGGACACAGACTGTCACACCATGGATTCACACTCAATCTCCAGAAGTAAATCCCTGGACACAGACTGCAGCTTCCACAGCTGCATTGTGGACTCATATTGAATCTCTAGCATTAAGTTATTCCGCACAGAGTATTACTGATACAGTTATATTTTGGACCATGCTTCAAACTGAATCTAAGAAACCCTGGAGTCTTTCCAAACCTAGTTTATTCACTATTTCACTGAATACTCAAAGTGATCTTACTGGATCACTGATTCATAATGAAAATCAAGCATATCTTCTGTTGACACATTCTGAAATTTATAATGTCAAGACACTGAACTTGCCTAAATCTGAAACACTCATATCATGGACAGTGCCCTTTCCTCAAGCAGTCAGTCTCTGGCCCCAATCTGAAGTTGATATTACCAGAACTTGGTTTAAAATACCAGAAAGAAAAACATCCTGGGCCCAGTCAGAATCTCTGACAATGAGTACCATGACACAGATAGGAGATGCTGGAATGAGGATGATAGCACAGCATGAACCTGCTGAAGTCACATCATGGATTCAGACTGAAAGTGGTATATTCCACCCTTGGGAGAAGTCTGAAGGAGATATAGTGAGATTCTGGACCCCTTCTGAAACTAAGCCTGTAAAACCATGGATGCAAACTGGAACTGCTTTAGTCACTCCACTGACCCAGCCTGAAATGCAAGCATTAAAATACATGAGCATGCCTGACATTAATTCTATCAGACCTTGGTTCAAGACTCAAACTGAGCCTATAAGAGAAGGCACTCAACCTGAATCTCAAATAGTTTCTACTAGGATCCAACCAGAACCACAAATCATCCATCCATGGTTCCAGCCTGAAAGTAATGCAGTCAGATTCTGGACTCAGCCTAAAGGTAATTTAGCCCAATCCTGGTTTCAAGCTAAAGCCAATATAGTAAGATTCCAGACTCAGTATGAAATTGATACAGGAAAACCATGGACCCAGCCTGAATCTCAGTCAGTCACATTTTTGTCCTTAACGCAAAATGATGCAATTAGTCCCACATCCCACTTTGAATCTCAGAAGACGTGTTCCTGGACCCAAAATGAATTTGGTATAATCAGTCTTTGGACTCAGTCTGAAACTAGTTCAGTCATATTTGGAACCAAGCTTGAAACTATTACAGGCCAACCCTGGCTTCATCTTGAATCTGCTACATGCAGACCATGGACCCTGCATGAAACCTTAACCCAGCCTGAAGCTGCTACAGTAAGACACTGGCTCCATACCCAAATGGACTTAAAAGAACCTTGGAATCAGCCTGAAGATGATGCAGTTATACACTGGACCCAGCTTGAAACAGAAACAATTTATGTTTGGACCCAGACTGAAAGAGAAGTAGTAAAACCTCCAACTTTATCTGAAGTCAATTTAGTCACATCTTGGTTACAGACTCAAAGTGATATAATTAGACCTTGGATTAAACCTGAGTTTCAGTCAGTCATTCCTTCGATCCACACTGGAGTTGATATACTTCACTTCTGGTCTCAGCAAAGAGCTGCTACAAATCAACCCAGAACATACCCCAAAACCCAGGTAGTCAGACCATGGATCAAGCTGGAGGCTGATACAATCAAATCTTGGTTCCACATTCAAAGGAATAAAGTCAGACCATGGATGCCTACAAAATCTCAAATATTGAGCCTCTGGAGGCAGCATGAAGTTGATATAGTCCATCCATGGATCCAGCCAGAAACCCTGGCAGACAGACCCTGGTCCCACTCTGAAACTGAGATTGCATTCTTGGAAAGTTATAAACCTGACCAAGTTAGAACATGGATCCAGCCTGAAATAGAAATAAGGCCTGGCATCCATTATAAAGCTGATAAAATGACATCATTTACTTCTCCTGAAGTAGAGCAAAATGAAACAACCCAATTAACCAGTCACTTTGGCTCTTGGTATAAACATAAACCTTTTATACCAACAGAAAGTATTCCTTCCCCGGATGAGTATTTTACAGCTTTGTCAACTGAGATAACTACACAAGAAATCCAAGATCAAATCAATTCTGTCCAACCCACTGAGCTCACAGATATTCTCTTTTTCACCCTTTCAAGCATGTGGTTTCCTGAAGGAGTTGGTTATCTAAAATTTGGCAGTAAATTACAAATTACCAAAACAAAAGGAAGCCCTGAGTTCCCATCTACTTCTCAGAGCCCCCTTTCTCCATCCCTTTCCTTTCCTGTTCCATGTTTTTCACCATCCCTGTGTTCCTTGTCCCTTTCCTGTTCGGTGTTTTCTTCTTGCGCATTCCCTAGACCCTGTATTTTCCCTTCTTGCTCAAGTCTTTCCTCTGTGGTATTCTCTCCTATTCTCCTTCCCTTAGCTGCTTCTGATAGTTTTCTCCAGAAACTATATTACTCAAAAGTTACTGAAGAAACCATTCTTTCTCATACTTCTCCATCCCTGAATATTGCTTCAGCCATACTTTTAACAAAGCAGCCTTCCCTGATGTCTGGATCTCAATCTGGAACCAAGTCTAATCAACCTGAACAAGATCCTCTCAAGTATTCTGAACTCAATATTTCCTTGGCTGAATGTCACCTGGGTGTGGTCTGGAAAGAGAATCTCCAAGCTTTCTGGCTATTCAAAACAGCTGTTATTTCCCATGAAACCACAGAGTGTGGATTACGCCCTGGCCTCGTCCCACATTGTCCCAACTGCTGGGAAGCAGAAGTGGGTGAATTCCCTTGGATGGTTTCTGTGCAACTGTCTTTCTCTCATTTCTGTGCTGGCTCCATACTGAATGAACAGTGGATCCTTACTACCGCTAGATGCGCAAATTTCATAAAAAACTCAGAAGCACTGGCCCTTGTCCAGGTGGGGCTTATTGATCTTCAAGACACTGCCCAAGCTCAAACAGTAGGCATTCATCGTGCCATGCCCTACCTAGGTCCTAAGGGCCCTTTGGGACCTGGGCTGATCTTCCTTAAGCAGCCATTACATTTTCAACCTCTGGTGCTTCCTATCTGCCTGGAAGAGAGTCTGGAGCAAGAGAAAAACATACAACTGTATGACTGCTGGCTACCTAGTTGGTCTCTCATGAGAGGAAGTCCTGGAATTCTGCAAAAAAGACACCTAAGCATCCTGCAAGTCAGCACGTGTGCTCAATTTTGGCCCAATTTGAGTGAATTTACTTTCTGTGTAGAAGCCAAGAAAGCTATGGGGGAAGCTGGCTGTAAGGGTGACCTGGGGGCACCTTTGGTGTGCCATCTACAACAAAAGGACACATGGGTGCAGGTGGGAATATTGAGTCATTTTGATGAACATTGCACAAAGCCCTACGTCTTCAGCCAAGTGAGCCCTTTCCTTTTCTGGCTCCAGGGAGTTACACGACCAAGCCATGCTCCCTGGTCCAAGCAAGGGCCCATGACTACTTCTGCTTCCATCTCACTTTCAGTATCTACCTCTACGAATGCTTCAGCTTTTACCTCCACTCCTGCTTCTGTCCAGCCTCACTTCATCTCACTGCCACAGCCTCAGACTCTGGCAGATCGGGTTTCTCTAAGATATGCCATGCCTTGGCAGGCCATGATTATCAGCTGTGGCAGTCAAATTTGCAGTGGTTCCATTGTTAGCAGCTCCTGGGTACTCACTGCTGCTCACTGTGTCAGGAACATGAATCCTGAAGACACTGCTGTAATACTGGGCCTGAGACACCCAGGGGCACCTCTGAGAGTTGTCAAGGTATCCACCATTTTACTGCATGAGAGATTCCGGTTGGTAAGTGGAGCAGCAAGAAATGACCTAGCATTGCTGCTGCTTCAAGAGGTACAGACTCCCATTCAGCTTTTAGCACCCTTAGGCCATCTAAAGAACTTGAATAGCTCAGAATGCTGGCTCTCAGGACCACGGATTCTAAAGCCAGGAGAGACAGATGAAAATCCAGAAATACTGCAGATGCAGGTGATAGGAGCTTCAAGCTGTGCCCACCTCTATCCAGACATAGGCAGTTCTATTGTGTGCTTCATTACACAGGACAAAGACTCCAATACAAATGTGGAACCGGTGAGCCCAGGCAGTGCTGTTATGTGCCGACCAATGTCTGGGAATGGAAGCTGGAGACAGATAGGGCTCACCAGTCTGAAAGCTCTAGCTACCATTGTGAGACCCCACTTCTCCTGGATATTATCCACTTCTGCAAAAGCAGGGCATCCCCTAAACCAAGCACTCATGCCTTGGGTAGAAAAGGCCAAATCCTCTAGTCTCTTAAAAAAGCCAACCATATTACCATCAGTAATAATTATTGCAATACAAAGCCTTTTGTAAGTTAATAGCTATAGAGGGAGGAGATAATCTGTTCACACTGTACTGGAAAGTATATTAACAATGTCATAACAAGACTGATACATTTAGATAGAACTCAGTAATTCTGAAATAAAGAAATACTCTATCTAAATGACTAATTTCTTTTAAAACACTTAAAAAACAAATTAT

>Platypus, Ornithorhynchus anatinus (BK059514, BK059515)

GAAATGGCTGCTGTGGGCGAGGTGGACCACGGAAGGCTGGGAAAGGCCCTAACCAGAAGACCACCTCTCCCCGCCCCACCGCCAAGGGGAAACAGGTCAGAACTGCAACAGAAAGTTGAATGAAGACTGAAATAAGAGAAGTGTTAATGCTTTGTTTGCCTCCTCCCTATACCCTTTACAGTCTACAACGTCCACAAGCAATGGCTGAACCGGCCTGCCCTGAGAAAGTCCAAAGGGGCAGGTACCAGTGAGCTTCCTTGGCTGGTGACAGTGGCTGGGACCTGCCAGGGAATCAGCCTTGGCCGCTGGTGGATCCTGACCACCACCAGCTGCCTGCTGAAGATGAAGTTCCCGCATCTGGAGGTTATGGGGGGTCCCGGCCCAAGAAGCAGCTTTCAGGGGAGCCAAGTCTGCCTGCACCCCAGTTTTAACCCCCAATCCTGGGAAGGGCCAGCAGTAGCTGCCCTAGGACTACTCCTCCTGAAGGAGCCCACAGCAATGCATAGGGACAAGGTGTGGCTCTCCAGGACTCCAGGCAACCTCCAAAGGAAGTGCCTTCATTGCAAGCAAAGGCAGTGTCAGGTCTACCAGCGCCAGAAAACAGCTTCAGATAATCTGCGACATCCGGGTGAGGAGATCACGATGATCCCCGTGAGGCTGCTGCATTCCTCGGCGTGCCACCGATACGGAGCCCGTGTGGAGGAGGACAAAAACCTCTGCATCAAGAGCAGAGACCATCATAAGTCTGACTGCCAGATGCAGCCGGGCAGCCCACTCCTCTGCCTCTTCGGGAGCCGCTGGGAGCTCGTAGGCCTCGTCGACGCAAGCCCCGGGAGCTGCTACGGCCCGACCCTCTCCATCAGGACCGCTCCCTATTCCTCCTGGCTGAGGCAGCACGTCAAAGCAGCCACACCGCTCCACTGCAGCCCTTACCCGCTGATGGAGCCGGCCACCCCCGGAGACTCCACTCTCAACTTCTCAGAACTAGCCTCCAAGTTCCAGCCGACGCACCCTTGGCAATTTAGGAATGGAAACACGGACATCTCACAAGTGACCTGGCTTCCCCGACGTTCCCCTCCGACGCCGTCGGCTCCTCCGTCCCCTCTGCCTACTGATGCCCCAGCTTCGTCTCTCCTCAGGCCCGGGGGCGGCCTTTCTTTTCTTCCAGCCAGCCACCTGCCCTCTGGCGACAGTTTGCCCATGGTAACCAACTGGGCTCCCCTTGTCACGCAGACAGCAGGAGCCAGGTCTCCCTTCACCGGCTCGACGGTCGTACACTCGGTTCTGTCCACGGGTCAGGCGGCTGCACTTTGGACTCCCTCTGGCGCGCAGCCAGTCAGATCTTCGGTGACCTTTCCCTCACGGTCAGGTGAACCGTGGACTTCGGCTAGACCCCCGGTTTCTATCCCTGCCCTGGGGCTGGCCCCGCTGGCTTCCCGTGCCCCTGGAATGGCCGAACTCTCCCCACCCTCAGCAACCCAGATCGTCGCACCGCCCTTGGCCGGCTGGACTCCTGCCAAGGCTCCAACGATGGGAACCCCTCTGGCCTCGACAGCCAAGACCCCCCAGTACCACTTCCCTAAAGTCTGGCCCGCTGCGCATGCTTCCTACGGATTCCATGCAGACGTCCAAGCCCCAAGCAGCAGACCTATTTTGCCCTGGACAATCCGCCCTGGCGGAACATGGCCTACTGAGCCCGGGACATCCGACGTGGCTTTCCCCCAGCCCCCTCCCAGCCCCTTCGTTACGGAGAGGTCCGGGGTTATTTCCCGTCTCCTGCCTTCCGAAAGTCCCCTGTATAATATGTGGACCAGGGGTTTGGCTAAGCCTAAAGCCTACTCTGGTGACTACTTTCACGTGGCCTCCTCTTTCCCCACGGCTAAGCCCAGGACCCTGGGTGACCTGCCTGCCTTTCCCCCACCTTTTAGCACTCCCGAGCCCCATTCCTTTCCTAGGCTTCTCCAGCAGGTGATGAAGCCAACTCCTCAAGATGCCATTTCTTATGGCAGTTCCTTTCTAAGGGAGCCGGGCTCCTCCCTGAAACCCCTCGTTTTGGTCACCCCTCGAACCAGACCCCTGTCTCCCGTGCACTTCCCATGGGTCTCGGAAGTTGGACCGGCATTCTATCATCACTCTCCTTTCTCAACGCCCTTCAGTCAAAACTTCCAGTCCGATTTGTCCCCCTTATACCAGCTTCAAGTGAAACAGAAAGCTGGGACCACCCTGGCCCAGTGCAGGCTAGCCCTGGCCTGGGAGATCAAGACAAAGGCCTTCAGCCTTTATCAAACCGCTGTGCCCATCAAGAAGTCCTTTGAGTGTGGAATGCGCCCAGGCTTTACCCACCGCTGCTCAGGTTGCTCGGAGGCAGATAGGGGCGAGTTCCCCTGGATGGCCTCCATCCAGCTCACCCTTTATCACTTCTGTGCGGGCTCTATCCTGAACGAGTGGTGGATTCTGACCACGGCCAAATGTGCCAGTTTAATAAAGAACTCCGAGGCTCTCGCTGTTGCTCAGGTGGGTGTCGTCAATCTCCAGGATCACGTCCAAGCCCAGGTGGTAAGCATTCACCATGCAATCCATCACCACAGCCCCCAGGGGCCCGTCGGCCTAGGCCTCATCCTCTTGCAGCAGCCGTTGCACTTCCAGCCCCTGGTCCTCCCCATCTGCCTGGAGGACAGCGAGAAGCAGGAGGAACATTTGAAAATCGCGGATTGCTACCTGCCCGGCTGGAGTCTCTTGAGGGGGGGGCCTGTAGCACTCCAGAAACGTCAACTCAGCATGCTCCGTCTCAGCGCCTGCTCTCGGTTCTGGCCCAAGCTCAATGAATTCACCTTCTGTATAGAGGCGAAGAAAGTTGGCATGGCAAGATGCCAGGGAGATCTGGGAGCACCCCTAATCTGCAAAGAGAAGCAGAAAGAAGTGTGGGTGCAGGTGGGGGTGCTCAGCAACTTCGACGAGCACTGCGTGAAGCCGTATGTCTTCATTCGGATAGCCCCTTACTTGTCGTGGCTAGAAAGTGTCACTCAGGATGACCCCCATGCTCCCTGGGGGGCTCAGACTGACACTTCCCTCCTCGTCTCCCTGCCGCACCCACGGACCCTCGTGAACCGAATCTCCGTGAGATTTGCCATGCCGTGGCAGGCCTTGATCGTTACGTGTGGCAGCCAGATCTGTGGCGGCTCGCTCCTGAATAACTCCTGGGTGTTGACCACCGCCGACTGTGTCAGAAATATGAAGCCAGAGAACATGGCTGTGTTTCTGGGCCTGAACCAGCACGGCTCCTCCTTGAGAGCCATCCGAGTTGCCAACATCTTCCTGCACGACGATTATTTCTCCAACAGCCCCAACAACAACCTGGCCCTGGTCCTCCTTCGGGGCCCCATCATGTCCGGCCAGGCCTTCGCCCCCATAAAGCGCCGGTGGACCAGGAACGATGGGGATGAGTGCTGGTTCTCGGGACCCCGGCTTCTCAGACCAGGAGAGGCCGAGGGCTACCCCAAGATGTTCCAAGTGCGGATACTGAATGACTCTTCCTGCTCCGAATTCTACCAGGAACCCAATACCGTCCTCTGTGTTGTTCCTAAACGGTCAAACCTCCCCAAGGCGAAGGTGAGTTCAGGCGGTGCCTTGCTGTGCAGGCTGGGAGCAGCTAATGGTAGTTGGGCCCAGACCGGCCTCGTGGGACCGTCCTACTTCTCCACCAACATCATTCCATTCTTGCGCTGGATGGAACTGACCGCCGCTGAGGCCGGCCGGCCCATTACCTTCTCTAAAGCCACTTCCTCCACAGTCTTTGGCTCCTGGGTCCCTGGGGTCCCGCTCTTTCTGCCCCTGCTGCTAGGGATGGTCGTGGGCCACGTCCCACCCCAGTTACTTTTCCCACCCTGACGCCTGCCTGTCTCACCCAGCTAGAGCCTCTGCGGAGGCAACAAACTGGCAAGACCAGCGTACCCCGGGGAGCTGGTAGTGTCGAACTGGTCGGGAGGAGGGCCTGTGAGTCACCCGGAGAGCCTGGGACAGCAGTGCGAAGG

>Polar Bear, Ursus maritimus

ATGAAACTGGACCATGCTCTCACACCATACACAAAGATAAACTCTAGATGGATAAAAGACTTCAATGTGAGACAAGAATCCATTAAAATCCTAGAGGAGAACACAGGCAGCAACCTCTTTGACATCAGCGACAGCAACTGTGACCCATCTCCAAAGGCAACGTCCAGCAACATCCCATGGCTAGTGTCCATGGCTGAAACCTGCCAGGGCATTATTCTGAGTCGGTGGTGGATCCTTTCCACGGCCAGCTGTCTGAGTAAATTGAAACATTTGCATTCTGACATTTCAGGAGTCATTGACCAAGAAGATATCTTACTTGGCCATAAAATATGCCTGCACCCCAGTTTTGCTGCACAAGGTGGAACAGATCCAGTCAAAGGAGACATAGGAGTGGTACAGCAGGGTAGTCCTGTTCTCTGCCTCTTTGGCAACCGCTGGGAACTGGTAGGCCTGGTCAGTGAGTCCTCAATGGCCTGTTACAACCCTATTCTTGTCATCAAGACAGCCCCATATTTATCTTGGATGAAATGGCTTATCAAGACATCCCAGAAGCCGTTGGATCCGATTTTTTCCATACCCTGCAGTTTTACTTCTGGTGTAGAACATGGCCCACAAGACAGGCTTACCCTGAACAGGGGCACTGCCATTTTGACCTCCCATGGATTCTCTGTTCAGACATGGAAGAGAAGATTAGGCACTTCCCCACTGAACAGACAGCGCCGGAATCCTTCTCCTGTATTTTTCCGTTCAAATAATAGAGACTCTTTTCTTGTCAGTAGACAGTCACACCTTCAAACTAGTCAGCTACCCTCAACTATCAAATTCCCAATGATACAATCTTGGACCTCTCTTGTTACCAAACAACGGGATCCTTCTGATATGTTTGAGCCCTGGAATACCCCAATAACTGATACTTCTGAAACTTTGGTTCTTTCTGGACCCCCAAAGCCTCGAATACCTGATGAATTTATACCCCAGGAACTTTCTCTGAAAGATACAATGAAATATCAATACCAGACTATGACCAATTCAGTAAACTCCTGGGTTAATCCTTTAGCTGGTATAATTGGGCTTCATACTCTACCACTAGTTAATTCTGCTATATCCTGGCTTTTGTTTTCAAGTGGCATAGATGGGTCCCAACTTCTTTCTGGGGTTAATACTGTACAGTCTCAAGTTCAGTCTAGTAAGGTTCCTTTCCATGGTCAAATTCTAGCTAAACCGTGGTTAGAAATCACCCCTGATGTTGGACCTTGGACACATTCTGTACTTGATAAAACAGGAACAGTGATGCAAATCCAATCTAGTGAAGAGAATATTGGAAGCAAAATACACCATGTAGTTGATAGAGTGCACACTTCTTTTAAAACAGTAACTTATAACTTGAATGCATGGGTTCCTTTAACAGTTAATAAAAATGAATTCTGGACTCATTCCACACTAAATGCAGATGGATCTCAGTATCCTATAGGAACTCTTACCTTAGAACCCTGGTTTCAGTCAGTCTTAAATTTAGATGGATCCCAAGAACTTGTAGAAAAGACTAATGAATACTGGATTCACCCTGAATCTAAGTCACCTCAGCTATGGACTTCTTCAGCACTTAATATGCCTCTTACTTGGGTTCCATCTGCAAGCAACACTATTAAGTCTTGGGCACAATATAAAACTAGTCTGATCAAAGTATCAAGTCAAGTGGATAGAATAAGTCCATTGAGTAAACATGAGTCTATTATGGTCAAACCCCAGATTCAAACTGAAGATACAACCTGGTTTTTGATACAAACTATTACAAATGTAATTAAGCCTTTTATTCAGTCTAATGCTGATACAGTCAGACCCTGGACTATACCTGAAGCTGACATAGTCCAAACTTGGACCCAGCAAGAAACCCAAAGAATAAACCCACTAACTCAGCTGAAATCAAATATAATCAGACCATGGTTACAGACTAAAACTGAAAGAAACAGACCCTGGATTCAACCTAAGTTTCAAATAGTCAGACCTAGAGCCCAGACTGAAAATGGTAAAGACAAACATTGGACTCAGCCAGAAGCAGATATGATTAGATCCATGACCCACACTGATATTAAAACAGTCAGGCTTGGGAACAAGCCTAAAGCTGGTACAGCCAGATCCTGGTCATGGATTAGGTCTAATCAAAGGAGAGCAAGGTCCCAGCCAGACTTTCAAACACTCTACCCTTGGACTCAGCCTGAGGTTGATATAGTGAGATCATGGATGAAGCCTGAAGCATCAATATTAAGAATCTGGACACAGTCTAAAGTTAATACAGTTACACCATGGACAGGGCCTGAAGCTGAGGCAGCTAGACTTTGGTTGCAAATTCAAACCAATACAGTCAGATCATTGAGCCAATCCGAATCTCAAACAACCATTTCCTGGTCTGAGCTTGAAGCTGATAGAGTCAGATCTTGGCTGCAGAGTCAGATGCATACATTCAAACCTTGGGTTGAGACAGAATTTCAAACAGCCCACTCCTGGACCCAACCTGAAGGTAATATAGCCAGGCTTTGGGCTAAATCTGAGACTGACAGTGTCAGACATTGGTTTCCGACTCAAGTAGAGATAGCCACAACATGGACAGAGCCAGTATCCCAAACAGCCCACCACTGGATACAGTCCAAAACAAAAATAGTCAGGCCCTGGAACCAGCCTGTGGGAGACAAGCTAAGAGCCTGGATACAACATGGCATTTATACTGTAAGGCCCTGGGATGACCTTGAAGGTGATAAAGTTAGATTCTGGATGCAATCTGAATCTGATACAAGACCTTGGATTCAGCCAGATGTGGGTATAATCAATCTTGGGACACAAAATGAAGCTGATACATCAACACCATGGGCATGGGTGCAGGATGAGAATCCAGAAGTTAATCCCTGGGCACAGTCTGAAACTGAAACAGCTATACTCTCGACCCAGGGAGAAGTTCCAGCAATAAATCTTTGGACAGAGATTTTAGTTGATACTGTCACAACATGGGCAAAGACTGAATTTCCAGGACAAAAGCTCTTGATGTATCCTTTATCTCATATGGTCACACAGTGGACTCAGCCTGAACTGACAGCGGCAAATCTCTGGACACAGCCTGTAGATGATACGGTCACTCAGTGGACCCAGGGTGAACCTTCAGAAATAAATTCCTCAACAAAAACTATAGCTGACACAGCCATGTGGACACAGGCTGACTCTCCAGCAGTGAATCCCTGGATATGGTCTGAAAATGATACAGTCATATCATGGACCCAGGATGAGTCCGTAGGAATAAATCCTTGGACACAGCCTATAGCTGATACAGTCACACAGTGGGCCCAGGGTGAACCTTCAGAAGTACTTCCTTGGGTGAAAATTATAGCTGATACACCATGGATGCAGGCTGAATCCCCAGCAATAAACCCCTGGACACAGACTGAAAGTGATATAGTCACACCATGGATGCAGGCTGAGTCTCCAGCAGTAAATCCCTGGACACAGTCTGAAACTAACACAGTCCTACTGTGGACTCAGGCTGAATCTCCAACAGTAAATCCCTGGATAGATACTGTAGCTGGGACAGGCACACAGTGGACCCACGCTGAATCTCTGGCAGTAAATTCTTGGACCCAGCCTATAGCTGATACAGTCACACTGTGTACTCAGAATGAGTCTCCATTATTACATTCCTGGAAAAAGTCTGAAACTCATACACTTACAACATGGACTCTGACAGAATCTTTAGCAGGAAATTCCTGGAAACAATATGAAACTGATAGTGCCTCAATGTGGACCAAAAAAGGAAATCTAGAAGTAAATCCCTGGACACAGTCTGAAACTGATACAACTTGGACCCAGGCAGAAACTCCAGGAGTAAATTTGTGGCCACAACCTATAACTGAAATAGTTTCACCATGGACTCTGGCTGAATCTCCAGCAGTAAACCCCTGGACAGAGACTGTATCTGATAGTGGTATACCATGGATCCAGGTTGAATCTCCAGCAGTAAATCCATGGACACAATCTATAGCTGATGTAGACACACTGTGGACTCTGGCTGATTCTATAGCAGTAAATCCCTGGACAGAGCCTATATCTGATAGTGTAATACAGTGGACCCAGAGTGAACCTCCAGCAATAAATCAGTGGACACAAACTGTATCAGATACAGTCACATCATTTACTGAGTTTGAATTTCCTGCAGTAGAAACCTGGACAGACCCATTGGCTGATGTCACATTGTGGACCCAGACTGAAACTCCAGTAGTAAATCCCTGGACAGAGACTGTAACTTCCACTGTCACACCATGGACCCAGACAAAATCGTCAGCAGTAAATCCACTGACAGAGGCTATAGTTGCCACAGTAATACCATGGAATCAGGCTGAATCTGTAGCAGTAAATCCCTGGATAGATGCTGTAGCTTTCACACTGTTGACTCAGGATCAGGCTCCATCAGTGAAACCCTGGACAGAGGCTGTAGCTTTCATAATCACACCACTGACTCTGGCTGAGTCTTTAGCAATAAAGTCTGTGAGACAGGAAGTATCTGATACAATCATATTCTGGAACCAAGCTGAATCACCTCTAGTACATCCCTGGACACAGTCTGAAACTGATGCAATCACACAGTGGACTCAGGGTGAGTCTCTAAAAGTAAATCCTTGGACACAACCTGTAGTTGACACAATCACACCATGGACTCATGTTGAGTTCCCAGAAGTAAATCCCTGGACAGATGCTGTAAGTGACACAGTACCACCAGTGAGCCTAGCTGAATCTCCTTCAGTACATCCCTGGACAACATTTGAAATTGATACAGTCACACTGTGGACCCAAGCTGAATCTCCTGCAATGAACCCCTGGACACAGTTTGAAACTGACACAGTCACACCATGGACCCAGGCTGAATCTGCTACAAGAAATCTGTGGATCCAGCCCAAAACTAATCTCGTCACACTGCAGACCCATGGTAGATACCCACCATTAAATTCCTGGGCACAGCCAGAAACTGACACATTTACATCATGGTCTCAGGTTGAGTCTCCAGCAGTCAACCCATGGACACAGTCTGAAACTGACACAATCACAACATGGACCCAAGCTGAATCTCCTGCAGTAAATCTCTGGACACAACCTGAAAATGTCACAGTCACACTGTGGATCCAGGCTGAATCCCTTGCAGTAAAACTCTGGGCACAGCCCGAAAATAACACAGTCACACCATGGACCCAGAATGAATCTCCAGAAGAAAACACCTGGACAGAGACTGTTTCTAAAACAGCCATACCATGGACCATGGGATTTTTTCCAACCATGAAGCCCTGGATAGAGACTATATCTGTTAAAGTCACACCAGCCACCAAATCTCAATTTTCAGAAGTAAAACTTTGGACACTGCAGTTGTCCAAAACATTGGACACTGAGGTAGGTACAGTCAAAATGTGGACTCAGTATGAATCTCCACCCTTAGTTCCCTGGACAGAAGCTATAGCTTCCATAGTCCCACTATGGACCCAGGTTGACACTCTAGCTGTAAATCACTGGACACAGCCTATAGCTTATAAAGTCACAAAATGGACACAGACTAAATTTCCATCAGTAAATACTTGGACTCAAGTTCAATTTCCAGCAGTAAATCCCTGGACACAATCTGAATCACCAGCTCTAAATATCTTGACAGAGTACAAATCTCCAGCCCTTAAATTATCAATTGAGCCTGAGGCTACTATAGTCACATTGTGGACTCAGATTGAATCTCCAGCAGTAAATCCTTGGATAGAGCCTGTAGCTTCCATAGTCATACCATGGACACAAGCTGAATATCCAGCAGTAAGTTCATTCACACAGGCTGTAACTGATAGAATTATACTCTGGACTCAGACAGAATCTCCAGCATTAACTCCCTGGACAAAGTCTGTAGCAGATACAGTCACAGTGTGGGCCCTGGATGAATCTTCAAGAGCAAAACTGTGGACACAGTTATTATCTTCATCAGATATATTTTGGACTCAAGCCAAAACAGTAAATCCTTGGTCTACTTTTGAAATATTCCCACCATTCACCCAAATTCAATCTGTAGTGGTTAAAACATGGACACGATTTGAGAGTGACACAGTCACAGCATTGACTCAGGGTCAGGCTCTTGCTATGAATCGCTGGACAGAGGCTGCCACTATCAGAGTCACACCATGGATCCAAGTTGTACCTCCAGCAGTAAATCCCTGGATAGAGGCTCGTGTATCCAGATTCACTCCATGGACCCAAGGTGTTCATCCAGGAGTAAATCCCTGGACAGAGGCTGGTGTATCCAGAGTCACTCCATGGACCCAAGCTCCTCCAGCAGTAAATCCCTGGACAGAGGCTGGTGCTTTCAGAGTCACTCCGTGGACCCAAGGTGTTCCTCCAGCAGTAAATCCCTGGACAGAGGCTGGCATATCCAGATTCACTCCATGGACCCAAGCTGTTCCTCCAGCAGTAAATCCCTGGACAGAGGCTGGTGCTTCCAGATTCACTCCATGGACCCAAGGTATTCCTCCAGTAGTAAATCCCTGGACAGAGGCTGGTGTATCCAGAGCCACTGCATGGACCCAGGCTGTTCCTCCAGCAGTAAATCCCTGGACAGAGGCTGATGTATCCAGATTCACTCCATGGACCCAAGGTGTTCCTCCAGCAGTAAATCCCTGGACAGAGGCTGGTGTATCCAGAGCCACTGCATGGACCCAAGGTATTCCTCCAGTAGTAAATCCCTGGACAGAGGCTGGTGTATCCAGAGTCACTCCATGGACCCAAGGTGTTCCTCCAGCAGTAAATCCTTGGACAGAGGCTGGTGCTTTCAAATTCACTCCATGGCAAGATATACCTTCTGTAGTAAATCCCTGGACAGAGGCTAGTGCTTCCAGAGTCATTCCATGGACCCAAGCTCCTCGAGCAGTAAATCCCTGGACAGAGGCTGGTGCTTCCAGAGTCATTTCACGGACCCACGCGCCTCGAGCAGTAAATCCCTGGACAGAGGCTGGTGCTTTCAGAGTCACTCCACGGACCCAAGATGTTCCTCCAGCAGTAAATCCCTGGACAGAGGCTGGTGTATCCAGATTCACTCCATGGACCCAAGCTGTTCCTCCAGCAGTAAATCCCTGGACAGAAGAAAGTGCATCCAGAATCAGTCTGTGGACCCAAGATGTTTCTCCAATAGTAAATCCTTGGACAGAGGTTGTTGCATCCAGAGTCATTCCGTGGACCCAAGGTGTTCCTCCAGCAGTAAATCCTTGGACAGAGGCTGGTGCTTTCAAATTCACTCCATGGACTCAAGCTCCTCCAGCAGTGAATCCCTGGACAGAGGCTAGTGCTTCCAGAGTCACTGCATGGACCCAATCTGTTCCTCCAGCAGTAAATCCCTGGACAGAGGCTGATATATCCAGAGTCACTCCATGGACCCAGCCTGTACCTCCAGCAGTAAATGCTTGGACAGAGGCTGTTGCTTCCAGAGTCATTCCATGGACCCAAGCTGTACCTTCTGCAGTAAATCCTTGGACAGTGATACTTGTTTCTACAGTTACACCATGGACCCAGGCTCCCTCTCCCCTAAATCCTTTGGCAGAGACTAAAGTTTCTACAGTGGGACTAGGAACTCAAAGTGATCATTCGTTAATAAAAATCTGGACATATTCTGTACTCTCCACCACTACATCCTGGACTCAGGCTGAATATCAAGCAGTAAGTTCATATATACCAAGTGTTACTAATACAGTCATATTTTGGACAATGCTTAAATCTGAGTCTAAGAAATCTTGGATACTACCTGAAGCTGGTATATTCAGTATTTCATTGCATCCTCAAAGTGATATTTCTCAATCGTTGATTCAAGGAGAAAATCAAGCATCTCTTCTGTCAACACATCCTGGAATTAATAATGTCAATACATGGACTTTGCCTGAATATGAAACACTGGTATCATGGATAGCGGCTTCGTCTCAAGCAGCCAGACTTTCACCCCTATCTGAAACTGGTATTAGCAGATATTGGTTTAAAACTGAAACAGAAAGAGTAAGAATCTTGACCCACTCAGAATTCCAAACAGTGAGTACTTTGACCCAGTTTGAAACTGGAACATTTGAGCCCTTGGCCCAACATGAAACTCATACAGTCATATCATGGATTCCAACTAAAACTGGTATATTCCACCTCTGGAATAAGTCTGAAAGAGACAGAGTAAGAAACGGGACCCTTTTTGGAGGTGATGCCTTACGACCATGGATTCAGATTGAAGCTAGTATGTTCAACCTCTGGATCCAGTCTAAAAGTAGTACAGTCACACCCTGGACCCAGCCTGAGTCTCAGTCAGTCAGTATCTGGACTGAAGGAAATACAGGCATATTTTGGTACTTGACTCAAAATGATGCAGTTAGTCCCTGGTCCCAACTTGAATCTCAAATGACATCTTCTGGGACCCAAAATGGTATAAGTAGCTCTTGGACTCCGTATGAAACTAGTAAAGTCAGATCCTGGACCAAGCTTGAAATCAGTACAGTGCAACCCTGGATTCAGGCTGAAACCGCTACAATTAGATCATGGGCCCAGTCTGAAAATGTAGGAATATACCCTGTGACCCAGTCAGAAACTGATACAATAATAGGACATTGGTTCCAGACTCAAATGGATTCAATGAAACCGTGGAACCAGCCTGAAGCAAATACAATTAGATCACGGACTCGACCTGAAACTGAAACAATCCAAATTTGGACCGAGAGAGAAAGTGAAGTAGTAAAACCTCCAACCTTATCTGAAGTTGATACAATTACATCTTGGTTACAGACTCAAAGTGATACAACTCGACTGCGGATTAAATCTGACTCCCAGTCTGTCAGTCCTTGGAGTCAGGCTGATGTTGGTATAAATCACCCCTGGACTCAGCAAAGAGGTACTGTGAATCAACCCTGGACCTACTCTGAAATCCAAACAGTCAGACCCTGGATGAAGCTAGAAGCTGATGCACTTAGATCTTGGTTCTACATTCAAATGAATAAAGTCAGACCATGGACCAATTCTGAATCTCAGATCTTCAGCTCCGGATTGCAGCCTGAAGTTGGTATGGTTCATCCTTGGATCCACCCTGAAACCCAAGCAGTCAGATCCGGGGCTTACCCTGAAACTGCGACACTTTTAACAAAAGAACCTTCTCTGATGCCTGGATCTCAGTCTGGAACCAAGTCTGATCAGCCTGAACAAGATCCTCTCAAGTATTCAGAACTCAGCGTTTCTTTGGCTGAGTGTCAACTGGGTGTGGTCTGGAAAGAGAGTGTCCAGGCTTTCTGGCTCTTCAAGACAGCTGTTATTTCTCATGAAACCACAGGCAAAAAAAACTCAGAAGCATTAGCCGTGGTCCAAGTGGGGCTTATTGATCTTCAGGAGCCTGCTCAAGCTCAAACTGTAGGCATTCACCGTGCCATGCCCTATCTAGGTCCCAAGGGACCTTTGGGTCCTGGGTTAATCTACCTGAAACAGCCACTGCATTTTCAACCCTTGGTGCTTCCTATCTGCCTAGAGGAGAACCTGGAGCAAGAGAAAAATATACAGCTGTATGACTGCTGGCTACCCAGTTGGTCCCTCATGAGAGGAAGTCCTGGGATTCTGCAAAAAAGGCACCTAAGCATACTTCAAGTCAGCACATGTGCCCAGTTTTGGCCCAAGCTGAATGAATTCACATTCTGTGTAGAAGCCAAGAAAGCTATGGGCGAGGCTGGCTGTAAGGGTGACTTAGGGGCACCTCTAGTGTGCCATCTACAACAAAAGGACACATGGGTGCAGGTGGGAATTTTGAGTCACTTCGATGAACATTGCACAAAGCCCTATGTCTTCAGCCAAGGAGTTACACGGCCCAGCCATGCACCATGGTCCCAGCACCGGGCCATGACTACCTCTGCTTCCATCTCCCTTTCAGTCTCTACCTCTATGAATGCCTCGGCTTTCACTGCTACTCCTGCCTCTATCCGACCACAATTCATCTCTCTGCTGCAGCCGCAGAGTAAGGCCCAAGAAAATGGTAGTGAGGGAGCTTTAGCAGATCGAATTTCTCTACGATATGCCATGCCTTGGCAGGCCATGATTGTCAGTTGTGGCAGTCAGATTTGCAGTGGCTCCATTGTTAGCAGCTCTTGGGTTCTCACTGCTGCCCATTGTGTCAGGAACATGAATCCTGAAGACACTGCTGTAATATTGGGCCTGAAGCACCCTGGAGCACCTCTGAGAGTTGTTAAGGTGTCTGACATTCTATTGCATGAGAGATTTCGGTTGGTGAGTGGGGCAGCAAGAAATGATCTAGCACTGCTGCTCCTTCAAGAGGTCCAAACTCCCATTCAGCTCTTAGCACCATTGGGATATCTGAAGAACCTCAATAGCTCAGAATGCTGGCTTTCTGGGCCAAGAGTTCTCAAACCAGGAACCTGTGACTCCTGGCAGTGCTGTTATGTGCAGACCAGTGTCTGGAAATGGCAGCTGGAGACAGATAGGTCTTACCAGTCTAAAGGCACTCGCTACCATAATCAGCCCACACTTCTCCTGGATATTATCCACATCGGCAAAAGCAGACCATCCCCTAAACCAGGCCCTAATGCCTTGGGCAGAGAGGCCAAAGTCATCTAG

>Rabbit, Oryctolagus cuniculus

CTCAACCTTGGTAGCCATTCTGCTACACCCACTTGAGCCAACATGAGAGCTCCAATATACCTTTACCAAATTTGGGGATTATACTTTTACTGCTGTGGTGGACCTAATTCACACAAGAAACAGTTAAACTCATGGGTCCAAATGATGGTCCCATTGTCAGCATACAGAAACATCTCATGGCTAATGTCCATGACTGGAAACTGCCAAGGCATTCTTCCAAATCAGTGGTGGAGCCTCTCCACAATTTGTTGTCTGTCCAAAATGAAGCATTTACATTTTGACAGTTCAGATGTCACTGACCAGCAAAATAGTTTACATGGCTATGAAGTCTGCCTGTATCCTAGTTTTAATCCACAAGTTGGAAGACATCAAGTCAAAGGAGATATAGGAGTGGTTCTCCTGCAGCTCCCCATCAGCGGGAAAGAAATTCTTCTTTCTAATATTTACCACATATTCTGGAAGAGCAGTTACAGGTGCCAGTACAGACACTGCTGTGTATACCAACTTCAGAATCATAATGCCTTTGTGGATAATATCAAGAAATTATCAGTTAAGCCTCTGGATCGCTCATTCTGCCATCATCCTCGTATCCATATGATGAAAACTAACAACTTATATATTATCTGGAATCAACCACAAGACAACTGCTGGGTACAGCATGGTAGTCCTGTTCTCTGTCTGTTTGGTTGTCACGGGAAACTGGTCGGCCTGGTCTGTGAGTCTTCTCCAGCATCTGACAACAACATTCTTGTCATCAACACAGCCTCATATTCATCCTGGATTAGATGGGTGATGAAAGCATTTCAGAAGAGGCTAGATACTTTTTTTTTTTTATCTTGTAGTTTTTTTCCTGAAGTAGAACGTGGGCCACAAGATGAGCTGAGACCTAACACAGCCTCCTCCACTTGGGCTCCCCATGAATCTCCTGTGCAATCCCTGAGATTAGACACTCACGCACTGAACAGACAGCGACGGAATCTTCTGCCCAAACATTTCCATTTCAATAATAGAGACTTATTTCATGATAGTAGACAGTTACACTTCCGAATTAGTCAGCTTTCCTACGCCAGCAGATTCCCCACAATACAACCCTGGATCTTTCCTGAGGCCAATCCAGGGGATCTTTTGAATCCAGCTGAATATGGGAGTATCACATTCACTGGTACTTCTGAATCTTGGGTTCATTTTGCAACCCAGAATCCACAAACACCTTGGGATTCTACTCAGAGAGATGCCAAGAAATATCCATATAAGTCTACGATGGCTTCAGTCATATCCTGGATTAAATCATCAGGTGACAGAATTGGGCTTCCAACTATGCCATTAGATAATGCTATTGGAGTCTGGGTTCTGTTTTCGAGTGACATAGACAAATCCCTGATGGCTTCTGAAATGAATCCTGTCCAGACGCATGGAAAATCTGGTATGGTTCCTTCCCAGATTCAATTTTTCACTGCATCCTGGTACAGGGAGGAAGTTACTCCAGATGTAATACACAGATCATATTCTTTATCTGATAAAACAGGAGCAATCATGCAGATTCCATCAAGTGAAAAGGGTGTTAGAACACAAATGCACCACATTGCTAATAGAGTGAATACAGTTGTTAAACTTGTAACTGATAACCTCTATCCTTGGGTCCCTTTCATCATGAATACATTTGAATTATGGACTCATTCTATGCTCACCAAGGATGAATCCCAGCATTTCACAGTAACCAATTCCATGGAATCATGGTTTCCACCAGTCTGGAAAATATTTGAATCCCAACAACCTGTAGAAAAGACAGATGGATACTGGATTTTTCCAGAATCACCTCTATTTTCCTCTACACATAGTATGTCTTTCTCTTGGGCTGAATATGCAAGTGACACAATTAGGCCTTGGATCCCAGATAAAAGCAGTGGGCTCACATTCCCAAGCCAAATTAACAGAATTGGTTTATTGAGTAAGCATGAGGCTCCCAAATACAAACATCAGTTGCAAAGCAAAGAAACAACATGGCTCTTGATCCATACTCTTGTGATTGATACCTCCAAACCCTGGAATAACCCTGAAGCAGGCAGAGTCCAAACCTGGACATATCTATATACTCATTTAGAAAGTCCGTTGATAAAGCTGAAAACCACCAGACTATTGTTACAGACTAAAACTGAAAAAATAAGACACTGGAGTCGATCCAAGTTTCAAATATGCAGATCCAGAACCCAGAATGAAGAAAGTATAAGGCAGTATGGGGCTCAGTCAGAAACTGACACAATTCCATCATGGAGCCACACTAAAACTGAAACAGTCATACCCTGGAAACAACCTGAGTTTGGTCCAGGCAGCTTGTGGTTCTGGACTAAATCTGATCAAGTAGTGAGATCTAGAACCAATATAGACTTTCAAGTTTTCCATCCTTGGATATATAGTAAAGCTGACAAATTCAGAAAATGGAATAATTCTGAAGTTGGTACTGTTAGACTATGGGTAAATCTTGAAGTATTAACTTTTACACCTTGGACACAGTTCTGGACAGAACCTGAGGCTAACGTGGCCCAACAAGTTAATAGGATAAGAATTTGGAGTCAAACTGAAGAGCCAACAATTACTTCTTGGTCTGAAAGTGAAGCTGATACAATTGGATCTTGGTTCAGTACACAAATAGATACATTCACACCTTGGAAGGAGATGGTATTCCAAACAGTCCTCCCATGGACCCAATTAGAAGGAGACATATCCAGACCTTGGATTCCAGCAGAGGGTGACGTGGTCAAGTTTTGGTTTCAGACTCAAACAGAGACACAGTTGACACAGTGGAAACAGATAGCTCATCAGTGGGTACAGTCTAAATCTGAAATAGTTAGGCCCTGGAGCCAACCTGTGACGGATAAGTTAAGAGTCTGGGATGAGCTTGAAGCTGATAGAATCAGGTTCTGGACTCAGCTTGAATCTGGCACATTAAGAACCAGGACCCAAGCAAATGTAGTTGTCATCAACCCTTGGGTACAAAATGAAGCTATATTTATACCATGGCCCAAGACTGAGTCCTCAGAATTAAATCTGATGACTCAATCAGATATGGCAGAAATCAGTTCATGGATATGGGATAAGACTCACATAGTCACCCTGGGTACTCAGACAGAAGCTTCAGAGCTAAATCCTGGGACGCTCTCTGAAACAGACACACTTGAGCTGTGGACTCAGGCTGAACGTCCTTCAGTGAACTGGTCAGACATATTAGCTGATACAGTCACCTTATGGACAAAGGATGAATTTCAAGAACTAAAACTTTGGACACAGTATGAAGTTAATACACTTACATTGTGGATCCAGGCTGAATCTCCAGCATTAATTCCCTTGGCAGTGTCTACAGCTAATTCACGGACACCATGGGCTGATGATTACCTTTCAGAAATGAATCTCTGGAGAAAAAAAGAAGGTAATACAGCCATGCCATGGTTGCTGGCTGAATCCTTAATTATGAATACCTGGACCATTTCTGAATTGTATTCAGCATCATTGTGGACTCCTGCTGATTTATCAACAGTCAATCTATGGAAACAATTGGAAACTAATATTATCACACAATGGGCCCCAAATGAATGGCCAACAGTAGATTCATGGACTCACCTGGTACCTGATATAGATACAATTTGTATCCAGGTTGAATCTTCATCATTAAATCTCTGGATACAGTCTGAAGCAGCGACATTCACATTGTGGACCCAGGCTGAAAATCAAGCAGAAAATCCCTGGTCTCTACCTGACATAGCTGAGACAATCAGACAATGGAATCCATATGCAACCCCAACAAGGAATCCTTGGCCAGAGACTCTGACTGATACAATGACATCATGGACCCAGGCTGAATTTCCCACAATAGACTCCTGGGCAGTTCCTGTGACTGAGACAATCACACTATGGCTCATGGCTGAATATGCAGCAGCAAATCCACAGAAAGGGCCTGTACCTGACACAGACACATGGTGGACCCAGACTGAATTTCTCAGGACCAATTCCTGGGTGGAGCCTATGACTGACAATGCTAAAATAAAAATTCAAGTAGAGTCTGTTGCAATCAGTTTATGGACACTGACTCTGTCTAATACAGTTATACCATGGATCCAGGCTGAATCTCCAGCTATAGAGCCAAAGAGAGAGGCTATTTCTTCCATGGCGGTACAATGGGAAGGGCCTTTATCTTCACAAGTAAATTCCTGGACAATGTCTGTAGCTGATGCTGTCAAACCATGGATTCATGGTTTTTCAGCTGTAAATCCATGGATATTTCCTTTTGCTGACATAGTCACACCATGGTCTCAGATAGACTATTTAGAAAACCATCCCTGGACACAGAAAATATCTGACACAGTCATCTTCTGGCCTCAGGCAGTTCATAGAGCCATGAATCTCTGGAACCAGACCATGTCTGGCACAGGGACACCATGGGCTCTCTCTGAATTTATAACAGGAAAACCCTGGATACATGCTATATCTGATGCACTCACACCATGGTCCCAGGCTGCATCTCCAACAGGAAGGCAAGGGATCCAGTCAGTGTCAGATATAGTTCCTCAATTAAACACAGCAAAATTGCCTGTAGCAAATCGCTGGACACATCATAAAACTGATATATTCACACAATTGGTGAATCTTGAATCTACAAAAGGAAATAACTGGATGCAGGCATCACCAGATATGGTAATAACATCAAAACAAACCATATCAGCAGCAATTAGTGCCTTGCTGGACATTGATTCTGCAAGAGAATGGATGCAGGCTGACTCAGAAAACACTAATCTCTGGACTCAGGTTGTGGTTGATACAATACCTTGGGTCCAGGATTTTCCTCTAGCAATAAAATCTCGGGCACAGTTTGAAGCTTATACAGTGATATCATGGAGCCAAGAGTTAACACCAACATTCAGTTCTATAACAGTGGCTCTAACTGATAGATTTATACTGTCGAACATGGCTGGATTTTTACATGTAAATCTCCTGACACATCCTGTACCTGGTACAGTTCTTTTTACTCAGGCTCCGAGGTCATCAATTAGTTACTGGATATACTCTAAAACAGAGATAGTTATATCATGGACTCAGCCCAAGTCTATAGGAATCAATGCCCATACAGAAACTGTTTCTACAACAATCACACCATGGGCCCAGAGTGAAATTTTTTCAGAAGTGGAAATGGGGACCCCTGCAGGAGATGTTGTAGACACAGTGTGGACCCACACTGTATTTCCATCGCTCATTCCCAAGATAGAGGTATTCAATCAAGTGGAAATTCTATCAGTATTTTCTTGGACACATTCAGTGTCTGATATGTTTACACCATGGGCCCGAACAGCATACTCAACAATGAATCCCTGGACATATCTTCTACCTACTAGGCACATACTGTGGACCCAGAATCAATCTCCACTAGTAAATATTTGGAATTGGGCTGAAATAGCCCCACACCATTTCAGGACACAGCCTAAACTGATAACAGTAAATATCTGGACAGAGTCTCAGTCTTCAGTGTTGAATTACAGAATGCAGACTGGGAACAGTGTAGAAATTCAGAAGATACAGGCTGAGTCTCTTGCAGTAAATCTTTTGACTTGGTCTGTAGCTGATACAATCATATTATATACAAATGCAGCATCTTCCTCAATGCATTCTTGGCCATACCCTGAAAATAACAGAGTTTCAGGTTGGATCCAGGCCAAATATCCACTCATAAATCTGTGGACAGAAACTATAGGTATTACTAGTAAAACTTGGTCTCAGGATGAATTTCTAGCACTGAATTCATGGAAACAGAATTTCACTTACACAGTTACACTGTGGGCACCAGCTGAATCTCCAATAATAAAAGTCTTAACACAACCTGCATCTAATGAAACTATTATACAGACCCATTTTGAATATCCAGCATCAATGACTTGGATATGGCCTGATTCTGAAAGAGTCATGCTATCAATCCAAGAAGAATATCAAGTAGTACCTCTCTGGACACAGTCTGTTTCAGACATACTCAATCAACAAGACCAACCTCTGTCTCCAGCAGTAATTACTTGGAAACAGGATATATATCCCAAACTCATACTATGGACAAAAGTTTTGTCTTCTGCAGAAATTCCAAGGACAGAGGCCACAGCTTTCATATTCACACCATGGGTCCATTCTTTTACTCCAGCAGTTACTCTCTGGACACAGGTTATAACTTCTACAGTTATACCCCAGACTCATGTTCCATCTCTGACAGTCATTCAATGGCTGGAAACTTCAGCTTCCAGCTTTCAGTTTGGAGAATGGATTCAGATTCCAACAGAGATGATAACTTCTACAATCATACTGTTGAAACATAATGAATCTACAGTGTTGAAGCTCAGGACAGAGCATGCAGCTTCCACTTTGACACCATGGACACAGGCTGTCTCTTTGGCAGTAATATCTTGGACACAGTCTACAGATTTTTCAGCCATACCACAGAGCCAGCCTCAATTACTAGTAATAAAAGCTTGGACAGAGGTGGTAGTAGCTTTGACAATGACACCATGGGCCCATGCTGTATTTCCAGCAAGCATTCTCTGGAGAGAGACTGGGGCTTCAAGAATCACACCATGGCCTCAAGCTCTATCTGAGCTAGTCAAATCCTGGGCAGAGGTCATAGTTTCCACAGGCATACCATGGGCCCAGCTTCCAAATCCAGAAACAAGTCCCTGGACACAGACTACAGCTTTCACAATTGTTCCAGAAACTCATGATCTTTCTTCAGCAGGAATATTCTGGACACAGGCTTCAGCTTCAGAAAGTATAGCAAGGACCCAGACTCAATCTATAGAAGTTTCTGGGACAGAAACAGCAACTCGCACACTCACACCATGGACTCAGGGTGTGTCTACAATTGAAAAATCTCAGACACAAATTATATCTTTCAGTGTCACTGAACGGAACCAAGCTGTATATTTAGGCAAAACTTCTCAGATGGAGGATACAACTTCCACAGTCATACCATGGAGGAAGCCTCTTTCCTCAGCTCTTCATTCCCATATACAGACAGAACTTAATACCACCCAATTTTGGACCATGCTTAAAACTGCAACTGAGAAGCACTGGGCATTGCCTGGAGCTCATATATTCAGATTTTCATGGCAGCCACAAACCTATTTGACCAATCCCTTGATTCAAGTTAAAAATCAATCACCTATTCTGTTGACATATACTGAAATTAAAAACATTAACACGTGGAATTTGCCTTTATCTGGAACACTCACATGGGTAGTATCTGCGCCTCTAGCAGGCAGAACATGGCGCCAGCCTGAAACTTACATTAGTAGAACCTGGCATAAAACTCACACGGAAATGATCACATCCTGGACACTTACAGATTATCAAGCATTTGATAATTTTATCCTGTACGCTATAAACACAGAACCCTTGGCTCAGCATGAAACTTCTACAGTCACATCATGGATTCATTTTGACACTGGTTTATTACATCAATGGACTCAGTCTGGAATAGATACAGAGAGATCCTGGGTTATTTATCAAGATGATACAGGAAGACAATGGACTCCAACAAAAGCTGGCAACATTCATTCCTGGACTCAACCTGAAACTTCCATGATCAGCCCACTAGCCCAGGCTGAATCTCAAACAGGTAGAATTTGGACACATCCTGTGATTGATATGGTTACACGATTGTCACAGGCTGATAAAAAAGCAGCAAAACTCTTGACAATCCCTCAAAATAATCATGTGATATCTTTGTTCCAGGTTCAAAATGATATAATAAAACAAGCAACTCAAAATGATTCCCCAACAGTTACCACCAAGATCCAGCCAAAATGGCCAAAAATCCATTCCTGGAGCCATCCTAAAACAGATGGCATCAGATGGTGGACGTATCCTGAAATGGAACAGTTTAAACTGTGGACCCTGTCTGACTATTCAAGAGTTAGGGAATGGCCTGAATTCACACTTGGGTCCTTGAGACAAAATGAGGCATTGACGCCTCAGTCAGAACTTGGCTCTCAAATGACCAATTTCTGGACCTGGCCTGAAGCCAGCGAATTGAAACCTTGGACTCAGTTAGATGCAGCGAGGCCCTGGAACCAACGCGAACTGGAAATCACACAAATCTGGAACCAGACTATAAAACAACTGGCAAACCTCCCAGCATTGACAAAAGTTGACACAGTGACAAATGGGTTGCAGACTCAAAGGAATACAACTGGACCATGGATTCACCCTGATTCTCAAACTGTCACATCTTGGACCAAGGATGCTGAAGTCAGTGTAGCAAATCTCTGGACTCAGGAGATATCTACCCATCAACCCTCGAATCATCATGAAATGAAAGTAGGTAGACCATGGATCAAACTGGAAGCTGACACAGCGAGATCATGCCTCTACATTCAAACACATCAAGGAAGACCATGGACCTATTCAGAATCTCAAATATTGAGTCCATGGACCCAATCTGAAACACAAACAGCTGCACTCTGGACCCAGTCTGAAATCTTCATAGTCAGATTCTGGTCCCAAATTGTACCTCCAGTATTCAAACCATGGGCTTTTCCTGAAAGAACACTTACCTCCTTGATTCATTCTGAAAACCAGACACACAGACCTTGGAACAAGTCCAACGTTCACATGATGACATCCTTTGTGATTTCCAAGCCTGAAACACACAGAACCTGGGTACAGCCTGTAACAGAAATACTAAGGTCAGAAACTTATGATAAAGCTGATAGGATTGAATCATTGACTGCTTCTAAGGCTGAGTCAGATAGAGAAATGCTACTGATGAGTCATTTTGGCTTTGGGTCTCAACTTGGACCATTTGTACCCATAGAAAGTATTCCTTCCTCAGTAGAGAATTTAATATCTTTATCAACTAAAACAGATGCTCTAGAAAACCAAGATAACAGAAAATATCTCCAATCCAGCCTGCCCACAAGCATATTCTTTACAATTTCAAGTACTTGGTCTTTTGGGAGAGATGGTTACCAGAATTTTGACAACCAATTACAAGTGATAAATACAAAGAGAAGCCCTAGAATCTCACATACCTCTTTTTATTCTCAGTATCCAGCTTATTCCTCTTTTATTTCTTGTTCTTTCCAACCTCCATGTATGTCATCCACTTCTTGTTTAGTGTTTTCTTCTTGCACATTCCCTTCATCCTGTATTTTCGCATCTTGTCCTGTGTTTTCTCCCGTGTCTCTCTCCTCTCCTCTTTTATCCTTAGCCTCTTCTAAAAGTCTTCTCCAGGAAACATCTTCATCAGAATTTACTCAAGAGACTATTCTTCTACATGCATTGTCATCCTCACATGCTTCTCCAGCAACATTGTTAACAAAACAACCTACTTGGTTGCCTGGATCTCAGTCTGGAACTGACTCTGAAGATCAGCTGGAAAATTCTGCCAAGCACTCAGTACTCAATGTTTCGTTGGCTGAATGCCGCCTTGGTGTAATTTGGAAAGAAAGTATCCAAGCATTTTGGCTCTTTAAGACAGCTGTCATTTCTCAGGAGATCACAGAGTGTGGATTACGCCCAGGGCTTGTGCCTCACTGTCCCAACTGCTGGGAGGCAGAGATGGGTGAATTCCCTTGGATGGTTTCCGTGCAACTCTCTTTCTCTCACTTCTGTGCTGGCTCCATACTGAATGCACAATGGATCCTCACAACAGCAAGATGTGCCAATTTCATAAAAAACTCAGAAGCTCTTGCACTGGTCCAAGTGGGGCTGATTGATCTGCAAGACCCTGCCCAAGCCCAAACTGTGGGCATTCACCGGGCCATGCCTTACCTTGGCCCCCATGGTCCTCTGGGGCCTGGGCTGATCTTCCTGAAGCAGCCACTGCATTTGCAACCCTTAGTGCTTCCTGTCTGCCTGGAGGAGAGCCTGGATCTAGAGAAAAACATGCAACTGCATGACTGCTGGCTGCCCAGTTGGTCCCTTATGCGGGGAAGTCCTGGCATTCTGCAAAAGAGGCACTTGAGTGTCCTGCCAATCAACACCTGTACCCAGTTTTGGCCCTTGTTAGATGAGTACAGCTTCTGTGTGGAGGCCAGGAGAGCCATGGGCGAGGCCGGATGCAAGGGTGATCTGGGGTCTCCTCTGGTGTGCCACCTGCAGCAGAGAGACACATGGGTGCAGGTGGGGATACTGAGTCAGTTTGATGAACATTGCACCAAGCCCTACGTCTTCAGCCATGTGAGCCCATTCCTCTTCTGGCTCCAGGGAGTGACTCAACCCAGCCTGGCACCCTGGGCCCAGCAGGGACCCATGACGACATCAGCATCCACCTCCCTCTCAGTCTCAGCTTCCACCTCACTCTCAGTCTCTATCCCTGAAAATGCCTCAGCTTTCACCTCCATGCCTGCTTCTGTTCAGCCACACTTCATCTCTCTGCCACAGCCTCAGACTTTGGCAGATCGGATTTCTGTACGATATGCATTCCCTTGGCAGGCCATGATTGTTGGCTGTGGCCATCAGATCTGCAGTGGTTCCATCATTGGTAGCTCTTGGATTCTCACTGCTGCTCACTGTGTCAGGAATATGAATCCAAAAGACACTGCTGTTATTCTGGGACTGAGGCACCCTGAGGGACCACTGAGAATTGTTCAGGTGTCTGCCATTCTACTCCATGAGAGATTCCAGCTGGTGGGTGGGGCAGCAAGAAATGACCTGGCATTGCTGCTGCTGCAAGAAGCTCAGACTCCCATCCAGTTCTTGGCACCTTTGGGTCATTGGAAGAATCTGAATCACTCAGAATGCCGGCTGTCTGGACCATGGATTCTTAAACCAGGAGAAACAGATGAGAATCCAAGTATCTTACAAATGCAGGTGATGGAAACATCTACTTGTGCCAATCTCTACCCTGAAATGGGCAATTCTGTTGTTTGCTTTGTGAATCAGGGCAGAAGTTCTGATACAAACGCGGATCCCATGAGCCCAGGCAGTGCTCTGATGTGCAGACCCAGATCAGGCAATGGTGAATGGAGACAAATTGGCGTCACCAGTCTTGAGACCCTGGCTACCGTCGTGAGCCCACACTTTTCCTGGATCCTATCCACAGCAGCCAAAGCAGGTCATCCTCTCAACCAAGCCCTCACACCAGGGATGGAAAGCCGCAAGTCCTCCACGCTTCTGAAACAGCCAAACATACGGCTTCTGACATGGATAATGGTGACTGCAGCCCACAGTCTACTGTAACCCAACACTAGTAGCAGATTAGGATAAATAAAGACACGACAGAT

>Rat, Rattus norvegicus (MW769785)

TCGTCATAAACCAAGACTGGCGTCTATGTCACAGACGAGTCCAGCATTCGACTTCCCGTTCTTTAAATCCCTCACTCAGTGTCTCAGCCGGCGGTCCCTCGGGTTCGAACTCCTGAAGTGAGAATGAGCGGCGTCCATTTCCTCTGCCATTCTCAGCGCTGTTTATTCACTGCCTTGGCCCATTGAACACTCAGAAGCCATGGCGACCTTTCACCCCATTGCCAACATGGCGGAGACCGTTCACTCTCCTGCAATCGGACTCATGGCCGACGTTCGACAACATCCCTTGGGTCGCAGCCATTGATGACACCTGCCAGGGAATTATCCTTAATCCATGGTGGATCCTGTCCGCAGTCACATGTCTGACTCGACTGAAACATTTCACCTCTGACCTCCCAAAAGCAATACACGAAGACGGAGTTTCTGGTGGGGATGAAATCTGCCTCCACCCTGGAATTAACCCCGGACGAGGAAAATACAAAGGTCACGACGTCATCGTTCATGGCATCGTCATCAGTCGCCTGCGGGTCCCAGTGAGGGGCAAGGAAATCTCACTTCCTCCATCATACATCATCGTCAGGAAAACCTGTTTGAACCGGCTGTGCAGTCATTGCGGAGTCTATCAATCCCAAAAGTACAGTTTGTTTGAGAACAACATAAGGAAGTTGTCAATTGAATTTCTGGATATTTCATTCTGCCATCATCAACATAACAGCGTGTCTAAAAGTAATAAACTGTGCATCTGGATTCATCAAAAAGAAGATTGCTTGATACAGCAAGGTAGCCCTGTTCTCTGTATCTTTGGCAGTCACTGGGAAGTCGTGGGCCTCATCAGCGAATCCTCAAGGACATGTGATGATACTGTTCTCATCATCAAGATGGCCCTGAACCTATCATGGACGAGGCAGTTTATTACTATAGATGAAAAACCACTGGATTGGATTTTCCCTTTGCTTTGCAATTTTTGCCCTGGAATAGAAGACAGTCTTAATAAGAACATAAGCAGTGCTGCTTTTGTCCCCCACGGATTCTCTTCTCACTCATGGAGTAGATTAATTACTTCCTCACAGAGCAGATCACGGTGGAATCCTCCTCCATTTCTCTTTGTTTCAAATAACAAAGACTCTTTTGCAGGCAGGCAGCACTTATATTTTCAAGTTGGACGTATCTTTTCAGCTAGCAAATCCTCAACATCACGACCCTGGACAATTCCTATGTTCAAAACTTTGGAGACGCTTTCAAGGGAGAAACCTACCCTGATGCCTCCACCTCAACTTGGCCCTCAAGATAAGCACTTAATGGAACCACTGTCTTTTAAGGAATCGATACTTAATGTTTCCTTGTCGGGATGTCACCTTAGTATGGTATGGAAAGATAATCTTCAAGCTCTATGGCTCTACAAAACAGCAGTTCTTTCTCATGAAACCACAGAATGTGGTTTACGCCCTGGCCTTGTCCCCCACTGTCCCAACTGCTGGGAAGCTGAAATAGGTGAATTTCCTTGGATGGTTTCTGTGCAACTTTCTTACTCCCACTTCTGTGCAGGCTCCATACTTAATGAAAAGTGGATCCTTACGTCTGCTCGATGTGCCAATTTTGTAAAACGGTCAGAAGCTCTGGCTTTAGTCCAAGTGGGGCTGGTTGATCTTCAGGACGCTACACAGGGTGAAATCGTAGGGATTCACCGTTCCATGCCATATTTAGGTTCCAGTGGACCATTGGGACCTGGCCTGCTCTTGTTGAAGGAGCCCCTACAGTTTCAACCTTGGGTGCTTCCTATTTGCTTGGTGGAGAGTCTGGATCAGGAGAGACATATCCAGCTGTATGACTGCTGGTTACCCAGTTGGTCCCTTATGAGGGGAAGTCCTGGTATCCTGCAAAAAAGGCACCTGAGCATCATGCAAATCAGCCCTTGTGACAAATTTTGGCCTCAACTGAATGAGTTCACTTTCTGTGTAGAAGCCAAGAAAGCTATGGGGGAATCTGGCTGCAAGGGTGATCTTGGGGCACCTTTGGTCTGTCATCTGCAACATAAAGACACGTGGGTGCAGATGGGGATTTTAATTCACTTTGATGAACAGTGCAAAAAGCCCTATGTCTTCAGCCACGTTAGTCCTTTCATTTCCTGGCTCCAGAGAGTCACACAGCCCAGCCACGCCCCCTGGTCCAATCAAGAACCTGTGACTATTTCAGTTTCTAATTCCGTGACAGTCTTTACCAATAGAAAAGTCTCAAAATTTACAGCTTCAGTTGAATCTACTCCACACTTCATCTCTCTGTCAGAGCCTCAGGCTTTAGGGGATCATATTTCTCTGCAATATACGATGCCATGGCAGGCTCTGATCTCCAGCTGTGGCAATCAGATCTGCAGTGGCTCCATGATTAATAGCTACTGGGTTCTCACCGCTGCCCACTGTGTCAGGCACATGAATCCCAAAGACACTGTTGTGATACTGGGCTTTCGGAATCCTGGGACAACTCTGAAAATTGTTAAGGTGACTTCTATTTTACTGAATGAAGAATTCCGGTTATCAAATCAGGGTGTGAGAAATGACCTAGCTCTTGTGAGAATTCAAGAAGGCCAGGGTTCTGTTCCCATAGTGGCGCCTTTGGGCAATATTAGGAATCTAAACACCTCAGAATGCTGGCTGTCTGGACCTCAGATTGTCAACCCAGGGGATATACTTGAAAACCCAGAAGTTCTACAGATACAATTGATGGGAGCTTCCAACTGTGCCTATCTCTACCCAGACATAGGGGGTTCTACTGTTTGCTATGCTTCACAGGCCAGGGGACCTGAAATAAATATGGAGTCAGTGAGTCCTGGAAGCACTGTTATGTGCAGGCCGATATCTGGCAATGGCAAATGGATACAAATAGGTCTCACCAGTCTCAAACATTTAGCTACTATAGTGAGCCCACACTTTTCCTGGATCTTGTCATCCACAGCAAAGGCAGGTTATCCCTTAAACCCGGATTTCAATCCTTGGGTAGAAAACCCGAAATCCTCTAGTCTTGTTAGACGTCCAACCACACCGCTATTTTACTTTGGAATGACTCTTGCAGTGATGAGGATGTTTATTTTGTAGTTAATTAAATACATTATGCAAGTCTGTTGAACTGTAAATAGGAAATCAGGAAAAAAAATCAATGAAATACTGTTATTCTCTGTAGCTTCTCCTTACATATTGTTTTAATAAACAGGA

>Tiny Cayenne Caecilian, Microcaecilia unicolor

TCTCTCTCCTGGTTTCAGGTAGAACCTTATTACTGTCCCACCTGAGACTTCAGCCGGCGTTCACCTAACAGAAGGATGAAATACCATTTTCTTATTGCCATCAATTTGGTGTTTCAGTGTTTAAGCTACAGCAATGCACTGAGCCAGGAGAGATGTTGTAAAGAGGAGACAGATCCGGGCGCTCTTCCACCACTTCTAAAGTTTGTTAGCTGGGATCTAAGCTTTCTGTCATCAGATCCTGCTACTCGAAAAAGGCGTTCCGTATTAGCCAGTGCACCTCTAGCTGAGCAAAAACACAACAATAGAGACCAGCATCACTATAAAGCCAATTCTTACTCATCCAGGTTACGACATTTGTTCTCCTCAAGATGGCCACATGGCCATGGAAGGCCACATCAAGCTCCTGTTAGATCACCTAGGACAAGACACCATGGCCATCCTCATGCCTTAGCAAAGCCATCCGATTCAATTCAGCCTCTGGTTGTACATCCACCAGCTGGTCCAAGTCAGGCACTTCTTTTACTATCACCAGCTGGCCCAGGTCAGCCTCTGCACGTATTGCCACTGAGAATGACAGATCCACCAGAGTTGCGGAAACCTTCAAGCCAAAATCTTCCTCTCACCTCATTGCAGCCAACAAGCCAGAGTCAGTCTCTCACCTCACTACAATCCTCCAACCAAAGTGGACCACTATCCTTACTGCAGTCGTCCAGCCAAGCTCAGCCTCTCACCTCACTGCAACCCTCCAACCAAAATGGACCACTATCCTCACTGCAGTCACCCAGCCAAGGTCAACCTCTCACCTCACTGCAATCCTCCAACCAAGGTGGACCACTATCCTCACTGCAGTCACCCAGCCAAGGTCAACCTCTCACCTCACTGCAATCCTCCAACCAAAGTGGACCACTATCCTCACTGCCGTCACCCATCCAAGGTCAGCCTCTCCCCTCACTGAAATCTTCCAACCAAAGTGGACCACTATCCTCACTGCAGCTGCCCAGCCAAGGTCAGCTTCTCTCTTCACTGCACTCTTCCAACCAAAGTGGACCACTATCTTCACTGCAGCTGCCCAGCCAAGGTCAGCCTCGCCCCTCACTGAAATCTTCCAACTTAAGTGGACTACTATCCCCACTGCAGCTGCCCAGTCAAGGTCAGCCTCTCTTCTCACTGCAGCCATCCAGCCAAGGTCAGCCTCTCACCTCACTGCAATCCTCCAACGAAAGTGGACCACTATCCTCACTGCAACCATCCAGCCAAGGTCAGCTCTCACTGCAGACATCCCGCCAAGGTCAGATTCTCCTCTCGCTACAGCCATCCAGCCAAGGTCAGTTTCTCCTTTCGCTGCAGCCGTCCAGCCAAGGTCAGCTTCTCCTCTCGCTGCAGCCCTCCAACACAGACCAGCCTCTTGTGCTGAAACTTCCTGACACAGGTTTGCAGCAACCATCTCCATCCTTTGTCACACCCGTACCCCCAGAAAATCGCACTTCTTCCTTCATGAATCCATCCCGCCTGGTGCGCCGGGGAGCTGTCATCCTTTCTCACTGTGGAGCCGACATGGAATGGAATGTTGGTAGCCAAGCTTTCCGTCTCTCCAAATTCTCCATACAGCGGAAGAGTTACCAAGCATGTGGCCAGCGCACGGCCTTCAAGCCCAAGCTCACCCCAGCAAACAACGAGGAAGCTGAAAAGGGAGAGTTTCCCTGGATGGTCTCCCTGAAGTTGTCCATTTATCACTTCTGCTCGGGCTCCATCCTCAACCGATGGTGGATCCTCACTACTGCGTCCTGCACCAACATCATAAAAAACGAGGAGTCCTCTGTTCTGGTCCAGGCCGGCATGCTGAACTTCCAGCTAGACTTTCGTTCCTTTCACGTAGAGCTGGTGGTGTCCCATCAAGAATACACAGAGGACCAGGAGACACACAACCTTGGTCTCATTCTGCTGCGCGAACCTTTATTCATCAGCCCTTTGATTTCACCCATTTGCATCTCCAAGAATATAAAATTGGAGCAGCTAATGACGCCCACCAACTGCTGGATCTCTGAATGGACATCACTGCAAGGTGGCCCCAGCATCCTGCTAAAGCGCAGGGTTTCACATCTTCAGCACACGCTTTGCAGTGACTTCTGGCCCATCATCTCTGATTTCACCTTTTGCATGAAGCTGAACCCGACCAACATGACAAACTGCAAGGGAGATATTGGGGCCCCTCTGGTGTGCAAAGACTTCAACAGTTCATCCTGGCTGCAGGTCGGATTACTCAGCGACTATGACAAGACCTGTGTGAAGCCCTACGTCTTCACCAAGGTCTCCCACTACCTGTCCTGGATTGAGCAGAGCACCCAGGCAGCCGGCAAACCCATCAGCCGGACAAAGTCTACATCTGCTGGGCGTCTTCAGAAGAAATGGCTGCGGGAGAAGGGCGATTTGCATACCGCTGAAAAAGACTCCATGTACCAAACAAAATCTTTTCGTATCTTTGCGCCATGGCAAACCCTTATCATCACCTGCCAGAACAAGATCTGCAATGGAGCCATCCTGGACAAGTACTGGATTGTCACCACAGCTGGCTGTGTGCAGAACATGGATCCGGATGATACTGCCGTCTATATGGGTCTTAACAGGCCAGAGCACATTGGTGACGTTATCAGAGCTGACCGTATCTTTCCACATGATGGTCATGATGAGAGTGTCAGTGTGGGAAATGACATTGCTCTGATCCTGCTAGAGGGTCCCATCTTGTTTTGGAAGCACGCCAGACCCCTGACCATGGCACGCGACCTGAACCTCGACATCAGCAGCATGGACACGTGTGGGATAGCAGGACTGCGCTGGTTAGAATCAGGAAAGGAATCATCTTCCACCATAAACTTGAAGAAGATACAGGTGCCAGTGAAAAATTCTGAAGTCTGTCCTGAGGATGAAGCCCTGATACAAAACGTAGCCTTCTGCATTGAGGAGGTCAGCACCCACCGCCAGCTACTGATGATCCAAGAGGGCAGCGCCATCCTCTGCACAAGCAAACAGGACTCAAACTGGACGCTGGTTGGCATTCTCAGCAAAGTATTGGATGAGTCCCCCATGCCTGCCTTAATCACCAGGCTCGCTGCCCACATCGACTGGATGAACAACGTGAGCAAAGCAGCCGGGAGACCGCTGGAGCTGCCGCCCACCACCCTGTCCCAGAAGGATCTCGAAGTGTCCACTTCCCGGGCCCACACTGGCCTTGTCCTGCTGATCGTTCTGCTCTCCTGCTTTGTTCTGCTCATCATCATCATCGGTGTGGGTGCTTTTGTACTGCACAAATTTTTTCCTAAATTCTTGACAGATTTAAAGTCAAAGTTGAAGTTGAAGTTCAAGTCAATCCTTAAACCCAAGCCTGAACCCAAGCCTGAACCCAAGCCTGAACCCATTCCTCTCACCCCCACTCCCTCAAAAAAAGCTAGAAAAGCAACCCATTTCTCTACTCCCTAAATATATATATATATATATATACACACACATACATACAATACATTATTGAAAGTGGGTAACATTGAGCAAGCAGTTGATAGGGAAACTAGGGAAACAATTGTAATATTCTGCTCTCATAATAGGCAGTTTCCTGATACCTACAAGTATTTTCAGTTACCACAAAATATAAGAACCCAGTGCAGATGCTCAGTATTAGAGAATTATAGTCACCTGAGAGGGAATGAATTGTGTGCCAGATAAAGCCAAACTGACCAATACAATTCAGCATTTTCCTTCTTATTAATTTAATGTATGTGGGAGATACATCTAAGCCATGATATTTTGAACTGGATTTCAGGATGTTTTCCATTGATTAGTTCATGAGTATTCTCTTTCAAGCAATAAAATAGTTTTCATACTTAAAAAAAA

>Sea Lion, Zalophus californianus (BK059503)

CCCTATCTTGCATTCACATCCGCTTGAGCCAACATGAGATCTCCACTTTGCTTCTATCAGTTTTGGGGACTGTTTTTTTCACTGCTCTGGTGTACCCAATTCTCAAAAGCAGTGGTTAAGCTCATTGACCCCAACGGTTGCCCCATTGACAGCATCCAGCAACATCCCATGGCTAGTGTCCCTGGCTGAAAACTGCCAGGGCATTATTCTGAGTCAGTGGTGGATCCTCTCCACAGCCAGCTGTCTGAATAAACTGAAACATTTGGACTCTGACATTTCAGCAGTCATTGACCAAGAAGATATCTTACTTGGCCATACAATATGCCTGCACCCCAGTTTTGGTCCACAAGTTGGAATGGATCCAGTCAAAGGAGACATAGGAGTCATCCTGCTACAGTACCCTATCAGGGTGGGGGGAATACCACTTTATCACACTTATAACATCTTCTGGAAGAGCTGTTATAACTGCCAGTACAGACACTGCAGGGTATACCAATATCAGAACCACAATAACTTTGGAACCAGTATCAAAAAGCTGTCAGTTAAGCTGCTGGACCTCTCATTCTGCCACCATCAATATATCCACCTAACTAAAAGTAACAACTTATGCATCTGGAGTCAGCCACAAGGAGACTGCTGGGTACAGCAGGGTAGTCCTGTTCTCTGCCTCTGTGGCAACCACTGGGAACTGGTAGGCCTGGTCAGTGAATCCTCAATGGCCTGTTATGACCCTGTTCTTGTCATCAAGACAGCCCCATATTTATCTTGGGTGAAATGGCTTATCAAGACATCTCAGAAGCCACTGGATCCTATTTGTTCCCTACCCTGCAATTTTACTCCTGGGGTAGAACATCGTCCACAAGACAGGCTTAGCCTGAGCAGGGGTACTGCCATTTTGACCTCCCACAGACTCTCTGTACAGTCACGGAAGAGAAGATTAGGCACTTTCCCACTGAACAGACAGCACCGGAATCCTCCTCCAGTATTTTTTCATTTAAATAATAGAGACTCTTTTCCTGGCAGTAGACAGTTACACCTTCAAGCTAGTCAGCTCTCCTCAACTAGCAAATTCCCAATGATACAATCTTGGACGTCTCTTGTTACCAAACAGCGGGATCCTTCTGATATTTCTGAGCCCTGGAATACCCCAATAGCTGGTACTTCTGAAACTTTGGTTCTTTCTGGACCCCCAAAGACTCCAACACCTGATAAATCTATACCCTGGGGCCTTCCTCAGAAAGATACAATGAAATATCAATACCAAACGATGACCAATTCAGTAAACTCCTGGGTTAATCCTTTAGCTGGTATCATTGGGCTTCATACTCTACCATTAGTTAGTTCTGCTATATCCTGGGTTTTGTTTTCAAGTGGCATAGATGGATCCCACCTTCTTTCTGGGGTTAATACCGTACAGTTTCAAGTTCAGTCTAGTAAGGTTCCTTTCCATGGTCAACTTCTAGCTAGACCCTGGCTAGAAATTACCCCTGACATTGGACCTTGGACACATTCTGTACCTGATAAAACTGGAACAGTGATGCAGATACAATCAAGTGAAGAGAATATTGGAAGCCAAATACACCATGTAGTCGATAGAGTTCACACTGCTTTTAAACCAGTAACTTATAACTTGAATGCATGGGTTCCTTTAACAGTTAATAAAAATGAATTCTGGACTCATTCCACGCTAAATGCAGATGGATCTCAGTATCCTACATTAACTCTTACGTTGGAACCCTGGTTTCAGTCAGTCTTAAATTTAGATGGATCCCAAGAACTTACAGAAAAGACTAATGAATACTGGATTCTTCCTGAATCTAAGTCAGTTCAATTGTGGACTTCTTCAGCACTTAATATGCCTTTCACTTGGATACCATCTGCAAGCAACACTATTAAGTCTTGGGCACAATATAATACCAGTCTGAGCAAAGCCTCAAGTCAAATGGAGAGAATAAGTCCATGGAGTAAACGTGAATCTTTTATGGTCAAACCCCAGATTCAAACTGCGGATACAACCTGGTTTTTGACACATATTATTACAAATGTAATTAAGCCTTTTTTTCAGTCTAAAGCTGATACAACCAGGCCCTGGAGTAAACCTGAAGCTGCCATAACCCAAACCTGGACCCAGCCAGAAACCCAGGCAAGAAAACCACTGACTCAGCTGAAAGCAGAAATCAGACGATGGTTACAGATTAAAACTGAAAGAATCAGACGCTGGATTCAGCCTAAGTTTCAAATAGTCAGACCTAGAGCCCAGACTGAAAATGGTAAAGACAAACATTGGACCCAGTCAGAAGCACATATAATTAGATCCGTGACACACACTGAAATTGAAACCTTCAGACTTGGGAACAAGCCTAAAGCTGGTACAGCCAGATCCTGGTTTTGGACTCAGTCTAATCAAATGAGAGCAAGGTCTCAGCCAGACTTGCAAACACTCTACCCTTGGACTCAGCCTGAAGTTGACATAGTGAGACCATGGACTCAGTCAAAAGCTAGTAGCATCCAACCATGGATGAAGCCTGAAGCATTGACATTCAGAATCTGGAGGCAGTTTAAAGTTAATACAATTACGCCCTGGACAGGGCCTGAAGCTGATATAGCTAGACTTTGGTTGCAAATACAAACCAATACATTCAGAACATGGAACCAACTTGAATCTCAGACAACCATTTCCTGGTCTGAGCTTGAAGCTGATAGAGTCAGACCCTGGTTGTATACTCAAATGCATACATTCAAACCTTGGATTGAGACAGAATTTCAAACAGCCCATTCCTGGACCCAACCTGAAGGTGATATACCCAGGCTTAGGACTAAATCTGAGGCTGACAATGTCAGACATTGGTTCCAGACTCAAGTGGAGACACCCACAATGTGGACAGAGCCAGTATCCCAAGTAGCCCACCACTGCATACAGTCCAAAACAGAAATAGTTAGGCCCTGGAACCAACCTGTGGCAGACAAGCTAAGAGCCTGGATACAACATGACATTTACACTGTAAGGCCCTGGGATGAGCTTGAAGGTGATAAGGTTAGATTCTGGACACAATCTGAATCTGACATAAGACGTTGGATTCAGCCAGATGTGGGTATAATCAATCCCAGGGCACAAAATGAAGCTGATACATCAACACCATGGGCATGGGGACAGGCTGAGACTCCAGAAGTTAATCCCTGGGCACAGTCTGAAACCGAAGCAGTTATACTCTCAAAACAGGGAGAAGCTTCAGCAATTAGTCACCAGACAGATATTTTAGCTGATACTGTCACAACATGGGCAAAGACTGAATTTCCAGGACAAAGGCTCTTGATGTATCCTTTGTCTCGTGTAGTCACACAGTGGACTCAGCCTGAACTGCCAGTGGCAAATCTCTGGACACAACCTGAAAATGTTACAGTCACACCTTGGATCCAGGATGAATCTCTTGCAGTAAATACCTGGACACAGACTGAAAATAACACAGTCATACCATGGACTCAGAATGAATCCCCAGAAGAAAATACCTGGACAGAGGCTGTTTCTGAAACAGCCATACCATGGACCATGGGATTTTTTCCAACCATGAAGCCCTGGATAGAGACTAAATTTGATAAAGTCACACCAGGCACCAAATCTCAATTTTCAGAAGTAAAACTTTGGACACAACAGTTGCCCAAAACATTGGACACTGAAACTGGTACCGTCAAAAGGTGGACTCAGTCTGAATCTCCACCCTTAATTCCCAGGATAGAAGCTATAGCTTCCATAGTCCCATTATGGACCCAGGCTGAATCTCTAGCTGTAAATCAGTGGACACAACCTATAGCTTATAGAGTCACAGAATGGACACAGACTAAATCTCCATCAGTAAATACTTGGACTCAGGTTCAATTTCTGGCAGTAAATCCATGGACACAGTCTGAATCAGAGTACAAATCTCCAGCCCTAAAACAGTCAGTTGAGCCTGAGGCTAGTGTAGTCATATTGTGGACTCAGACTGAATCTCCAGAAGTAAATCCTTGGATAGAGCCTGTAGCTTCCTTAGTCATGCCATGGACACAAGCTGAATATCCAGCAGTAAGTTCATTGACACAGGCTGTAGCTGATACAATTATCCTGTGGCCTCAGGCCGAATCCCAAGCATTAAATCCCTGGACAAAGTCTGTAGCAGATACAGTCACAGTGTGGGCCCTGGATGAATCTTCAAGAGCAAAACCTTGGACACAGATATTAACTTCATCAGATATATTTTGGACCCAAGCCAAAACAGTAATTCCTTGGTCTACTTTTGAAATATTCCCACAATTCACCCTTATTCAATCTGTAGTGGTTAAAACTTGGACACAATTTGAGAGTGACACAGTCACAGCCCTAACTCAGGGTCAGGCTCTTGCTGTGAATCCCTGGACAGAGATTATCACTTCCAGAGTCACACCATGGATCCAAGCTGAATTTCCAGCAGTAAATCCCTGGACAGAGGCTGTTGCTTCCAGAGTCACATCATGGACCCAAGCTATATCTGTAGCAGTAAATCCAGGGACAGAAGCTGTCACTTTGAGAGACATACCATGGATCCAAGTAGTATTTCCAGTAGCAGTAAATACCTGGACAGAGGCTGTCACTTCCAGAGTCTTTCCAGGGACCCAAGCTGTACTTCCAGTAGTAAATCCCTGGACAGGCACTGGTGCATCCAGAGTCATTCCATGGACACAAGGTGTACCTCCAGCAATAAATCCCTGGACAGAGGCTGGTGCATCCAGAGTCACATCATGGAGCAAAGCTCTTCCTCCAGCAATAAATCCCTGGACAGAGGCTGGTACATCCAGAGTCACATCATGGACCCAAGCTGTATCTCCAGAAGGAAATCCCTGGACAGACGCCGGTGCATCCAGAGTCATGCCATGGACCCAAGCTGCACCTCCAGGAATAAATCCCTGGACAGAAGCTGATGCATCCAAAGTCACTCCATGGACCCAAGCTATCCCTTCAGAAGTAAATCCCTGGACAGAGGCTGGTGCATCCAGAGTCACTCCATGGACCCAAGCTGTACCTCCAGCAGTAAATCCCTGGACAGACACTGATACATCCAGAGTCACATCATGGACTCAAGCTGTACCTCCAGCAGTAAATCCCTGGACAGAGGCTCTTGCTTCCAGAGTCATGTCATGGAACCAAGGTGTGTTTCCAGCAGTAAATCCCTGGACAGTGACACTTGTTTCTACCGTCACAACATGGACCCAGGCCTCCTCTCTTCTAAATCCTTTGACAGACACTAAAGCTTCTACAGTGAAAATATGGACTCATAATGAATATTCATTAGTAAAATCCTGGACACATTCTGCAATGTCCACAATTATATCCTGGACTCAGTCTGAATATCAAGCAGTAAATTCTTATATACCGAGTGTAACTGATACGGTCATATTTTGGACTGTACTTATATCTGAGTCTAAGAAATCATGGATACTGCCTGAAGCTGGTGTATTCAGTATTTCATTGCATCCTCAAAGTGATTCTACTCAATCCTTGATTCAAGGAGAAAATCAAGCATCTCTTCTGTCGACACATCCTGGAATTAATACTGTTAGTACATGGCCTTTGCCAGAATTTGAAACACTGGTATCATGGAGAGTGCCTTTGTCACAAGCAGCCACACTCTTACCCCTATCTGAAACTGATATTAGCCGATATTGGTTTAAAACTGAAACAGAAAGAGTAAGTACCTCGGCCTGCTCAAAACTTCAAACAGTGAGAACTTTGACCCAGTTTGAAACTGATAGATTTGAGCCCTTGGCCCAACATGAAACTCCTACAGTCATATCATGGATTCCAACAAAAACTGGTATATCCCACCCCTGGAATAAGTCTGAAAAAGACAAAGTAAGAACCTGGACCCTTTCTGGAGGTGATGCCTTGCGACCATGGATTCACAGTGAAGCTAGTATATTCAACCTCTGGATCCAGTCTAAAAGCAGTACAGTCACACCCTGGACCAAGCCTGGGTCTCAGTCAGTCAGTATCTGGACTGAAAGAAATACAGGCACATTTTGGTACCTGACTCAAAATGATGCAGTTAGGCCTTGGTCCCAGCTTGAATCTCAAATGACATCTTCTGGGACCCAAAATGGTATAAGTAGCTCTTGGACTCAGTATGAAACTAGTACAGTCAGATCCTGGACCAAGCTTGAAATCAGTACAGTGCAACCCTGGATTCAGGTTGAAACTGCTACAATTAGATCACGGACCCAGTCTGAAAATATAGAAATATACCCCCTGACCCAGTTAGAAGCTGGTACAGTAATAAGACACTGGTTCCAGACTAAACTGGATTCAATAAAACCTTGGAACCAGCCTGAAGCCAATACAATTAGATCATGGACTCAACCTGAAACTGAAACAATCCAAATTTGGACCCAGACAGAGGGCAAAGTAGTAAAACCTCCAACTTCATTGGAACTTGATACAATTACATCTTGGTTACAGACTCAAAGTGATACATTTCAACCCTGGATTAAATCTGACTCCCAATCTGTCAGTCCCTGGAGTCAGGCTGACGGTATAAATCACCCCTGGATTCAGCAAAGAGGTACTGTGAATCAACCCTACTCAGAAATCCAAACAGTCAGACCCTGGATGAAGCTAGATGCTGATGCACTTAGATCTTGGTTCTACATTCCAATGAATAAAGTCAGACCATCGACCAATTCCGAATCTCAGGTCTTCAGCTCCGGGTTGCAGCCTGAAGTTGGTATGTTTCACCCTTGGATCCATCCTGAAACCCAAGCAGTGAGATCCTGGGCCCACCCTGAAACTGCCACCCTTTTAAGAAAAGAACCTTCTCTGATGCCTGGGTCTCAATCTGGAACCAAGTCTCATCAGCCTGAACAAGATCCTCTCAAGTATTCACAACTCAACGTTTCTTTGGCTGAGTGTCACCTAGGTGTGGCCTGGAAAGAGAGTGTCCAGGCTTTCTGGCTCTTCAAGACAGCTGTTATTTCTCATGAAGCCACAGAGTGTGGATTACGACCTGGCCTTGTGCCCCACTGTCCCAACTGCTGGGAGGCAGACATGGGTGAATTTCCTTGGATGGTTTCCGTGCAACTCTCTTTCTCCCATTTCTGTGCTGGCTCTGTACTGAATGAACAGTGGATCCTTACCTCAGCTAGATGTGCAAATTTCATAAAGACCTCAGAAGCACTAGCCCTGGTCCAAGTGGGGCTTACTGATCTTCAGGAGCCTGCTCAAGCTCAAACTGTAGGCATTCACCGTGCCATGCCCTACCTAGGTCCCAAGGGACCTTTGGGTCCTGGGCTGATCTTCCTGAAACAGCCCCTACATTTTCAACCCTTGGTGCTTCCTATCTGTCTAGAGGAGAACCTGGAGCAAGAGAAAAATATACAGCTGTATGACTGCTGGCTACCCAGTGGGTCCCTCATGAGAGGAAGTCCTGGAATTCTGCAGAAAAGGCATCTAAAAATACTGCAAGTCAGCACATGTGCCCAGTTTTGGCCCAAGCTGAATGAGTTCACTTTCTGTGTGGAAGCCAAGCAAGCTCTTGGCGAGACTGGCTGTAAGGGTGACTTAGGGGCACCtCTGGTATGCCATCTACAACAAAAGAACACATGGGTGCAGGTGGGAATTTTGAGTCACTTCGATGAACATTGCACAAAGCCCTACGTCTTCAGCCAAGTGAGCCCTTTCCTTTTTTGGCTTCAGGGAGTCACACGGCCCAGCCATGCACCATGGTCCCAGCAAGGGGCCATGACTACCTCTGCTTCCATCTCCCTTTCAGTCTCTACCTCTACAAACAACTCAGCTTTTACTGCCACTCCTGCTTCTGTTCGGCCACAGTTCATCTCTCTGCCACAGCCTCAGACTTTAGCAGATCGAATTTCTCTACGATATGCCATGCCTTGGCAAGCCATGATCATCAGCTGTGGCAGTCAAATTTGCAGTGGTTCCCTTGTTAGCAGCTCTTGGGTTCTCACTGCTGCCCACTGTGTCAGGAACATGAATCCTGAAGACACTGCTGCAATACTAGGCCTGAAGCACCCTGGAGCACCTCTGAGAGTTATTAAGGTGTCTAACATTCTACTGCATGAGAGATTTCGGTTGGTGAGTGGGGCAGCAAGAAATGATCTAGCACTGCTGCTCCTTCAAGAGGTCCAGACTCCCATTCAGCTCTTAGCACCATTGGGACATCTGAAGAACCTCAGTAGCTCAGAATGCTGGCTTTCTGGGCCAAGAGTTCTCAAACCAGGAGAAACAGATGAGAATCCAGAAATGTTACAGATGCAGGTGATGGGAGCTTCAGGCTGTGCCCACCTCCACCCTGACATTGGCAGTTCTATTATCTGCTTCATTACTCAGGGGAAAGGCTCTGATACGAATGTGGAACCTGTGACTCCTGGCAGTGCTGTTATGTGCAGACCAGTGTCTGGAAATGGCAGCTGGAGACAGATAGGCTTTACCAGTCTAAAGGCACTTGCTACCATTGTCAGCCCACACTATTCCTGGATATTTTCCACATCAGCCAAAACAGGTCATCCCCTAAACCAGGCACTCATGCCTTGGGTGGAAAAGCCAAAGTCATCTAGTCTTCATAAACAGGCAACACTACCACTTTCATCATTAATGATTCTTGTAATGCAGAGTCTATTGTAAACCAGTGGTTATAATGACCAGTACCAATCTGGTCACACTGTGATAAAAAAAAAAAAAGAGATCAGAACTTCCAATTAAAACAAAACAGTTGAATTAAGACCCTAACTTCTCTGCAGTTATTCCTTAGAAAAGCATCAATTCTATCCATTAAAATGACA

>Sea Otter, Enhydra lutris kenyoni

NTACCCAGTTCTCAAAAGCAGTGGTTAAGCTCATTGACCCCAACGATTGCTCCATTGACAGCATCTAGAAATATCCCATGGCTAGTGTCCATGGCTGAAACCTGCCAGGGTGTTATTCTGAGTCGGTGGTGGGTCCTCTCCACAGCCAGCTGTCTGAGTAAACTGAAACATTTGCACTCTGACATTTCAGGGATCACTGACCAAGAAGATATCTTAATTGGCAAAAAAATATGCCTGCACCCCAGGTTTGATCCACAGGTTGGAATGAATCCAGTCAAAGGAGTTATAGGAGTGGTCCTTCTGCAGTACCCTATTAGAGGGGAAAAAATACCACTTTATCAGACTCACAACATCTTCTGGAAGAGCTGTTATAACTGCCAGTACAGACACTGCAGGGTACAGCAGGGTAGTCCTGTTCTCTGCCTTTTTGGCAAACACTGGAAACTGGTGGGCTTGGTCAGTGAATCCTCAATGGCCTGTGATGACCCTATTCTTGTCATCAAGACAGCTCCATATTTATCTTGGATGAAATGGTTTATCAAGACATCCCAGAAGACACTGGATCCTATTTTTTCTCTACTCTGCAGTTTTACTCCAGGGTTAGAACATGGTCCACAAGACAGGCTTAGCCTGAACAGGGAAACTGCCATTTTGACCTCCCATGGATTCTCTCTACAGTCATGGAAGAGAAGATTAGGCACTTTCCCACTGAACAGACAGCGCCGGAATCCTCCTCCCATATTTTTTCATTCAAATAGTAGAGACTATTTTCCAGGCAGTAGAGAGTTACATCTTCAAACTAGTCAGATCTCCTCAACTAGCCAATTCCCAATGATACAATCTTGGACCTCTCTTGTTACCAAACAATGGGATCCTTCTGATATGTCTGAACCCTGGAATACCCTGACAGTTGATACTTCTGAAATTTTGGTTATTTCTGGAACACCAAAGCCTCGAACACCTGATAAATCTATACCCTGGGGCCTTCCTCAGAAAAATACAATGAACTATCAATACCAAACTATGACCAATTCAGTAAATTCTTGGGTTAATCCTTTAGCTGGTAAAATTGGGCTTCATACACTGCCATTAGTTAATTCTGCTATATCCAGCGTTTTGTTTTCAAGTGGCATAGATGGATCCCAACTTCTTTCTAGGGTTAATACCGTACAGTCTCAAGTTCAATCTAGTAGGTTTCCTTTTCATGGTCAACTTCTAGCTAGACCCTGGCTGGAAATTACCCCTGACATTGGACCTTGGAAACATACTGTACCTGATAAAACAGAAACAGTGATGCAGATCCAATTTACTGAAGAGAATGTTGGAAGCCAAATACACCATGTAGTTGATAGAGTTCACACTGCTTTTAAACCAATAACTTACAACTTGAATGGATGGGTTCCTTTAACAGTTAATAAAAATGAATTCGGGACCCATTCCACACTAAATGCAGATGGATCTCTGTATCCTACAGTAACTCTTACCTTGGAACCTTGGTTCAAGTCAGTCTTGAATTTAGGTGGATCCCAAGAACTTATAGAAAAAACTAATGAATACTGGAGTCTCCCTGAATCTAAGTCAGCTCAATTATGGACTTCCTCAGCACTTAATATTCCTTTTACCTGGATTCCATCTTCAAGCAATAATATTAAGTCTTGGGGAAAATATAAGACCAGTATTATCAAAGCATCAAGTCAAATGGATAGAATAAGTCCATTGATTGAACATGATGAGTCTATTATGGTCAAACCCCAGATTCAAACTGCAGATACAACATGGTTTTTGATACCTACTATTACAAATGTAATTAAGCCTTTTGTTCAGTCTAAAGCTGTTACAATTAGACCCTTGACTAAGCCTGAACCTGACATAGTCCAAACCTGGACCCAGCCAGAAGCCCAAATAAGAAAACAACTGACTCAGCTGAAAGCAGATAAAATCAGACCATGGTTACAGACTAAAGCTGAAAAAATCAGATCCTGGATTCAGCCTAAGTTTCAAATAGTCAGAGCTAGAGCCCAGAATGAAAATGGTAAAGACAAATATTGGACCCAGCCAGAAGCAGATATAATTAGATCCATGACCCACACTGAAATTAAAACAATCAGACTTGAGAACAAGCCTAAAGCTGGTATGGCCAGATCCTGGTTATGGACTAGGTCTAATCAAATGAGAACAAGTTCCCAGCCAGACTTTCAAACACTCTACCCTTGGACTCAGCCTGAAGTTGACATAGTAAGACCATGGACTCAGTCTGAAGCTGGTAGCATCCAACCATGGATGAAACCTGAAGCATCAACAGTCAAAATCTGGACACAGTCTAAAGTTAATACAGTTACACCCTGGACAAGGCCTGGAGATGATGCAGCTAGACTTTGGTTGCAAATACAAACCAATACAGTCAGGACATGGAGCCAACCTGAATCTCAAACAACCATTTCCTGGTCTGAGCTTGAAGCTGATAGAGTCAGATCTTGGTTGCAAACTCTAATGCATACATTAAAACCTTGGATTGAGACAGGATTTCAGACAGCTCACTTCAGGACCCAACCTGAAGGTGACATAGCCAGATTTTGGACTAAATCTGAGACTAACAATGTCAGACATTGGTTCCAGACTCAAGTGGACACAGCCACAATATGGACAGAGACAGTATCCCAAGCAGCCCACCCATGGATACAGTCCAAAACAGAAATAGTCAGGCCGTGGAACCAGCCTGTGGCAGACAACTTAAGAGCCTGGATACAACATGACATTTATACTGTAAGGCCCTGGGATAAGCTTGAAGGTGATAAAATTAGATTCTGGACACAGTCTGAATCTGACACAAGTCCTTGGATTCAGCCAGATATAGGTATAATCAATTCTGGGACACAAAATGAAGGGGATACATCAACACCATGGGCATGGGTGCAGGCTGAGACTCCAGAAGTTAATCCTTGGGTACAGACTGAAACTGAAACAGTTATAGTCTCAACCAAGGGAGAAGCTCCAGCAATTAATCAATGGACAGAGGTTTTACCTGATGCTGCATGGGCAAAGACTGAATTTCCAGGACAAAAGCTCTTGACATATCCTTTGTCTCATACAGCTGCACAGTGGACTCAGCCTGAACTGCCAGTGGCCAGTCTCTGGACACAACCTATAGATGATACAGTCACTCAGTGGAACCATGATGAACCTTCAGAAATAAATTCCTCAACGAAGACTATAGTTGATACAGTCATGAGGACACAAGCTGAATCTCCAGCAGTGAATCCCGGGATACAGTCTGAAAATGATACAGTCATATCATGGACCCCGGATGAATCCCTAGTCATAAATCCTTGGACAAAGTCTGTAGGTGATACAGTCACACAGTGGGCCCAGGGTGAACCTTCAGAAGTACTTCCTCGGGTAAAAATTGCAGCTGATACACCATGGATGCAGGTGGAATCCCCAGCACTAAAGCCCTGGATGCAGTATGAAAGTGATATAGTCACACCATGGATACAGGTGGAATCCCCAGCACTAAAGCCCTGGATGCAGTCTGAAAGTGATATAGTCAAACCATGGATACAGGTTGAGTCCCCAGCAGTAAATCCCTGGATACAGTCTGAAACTAACACTGTCCTACTATGGAATCAGGCTGAATCTCCAGCAGTCAATCCCTGGATAGATACTATAGCTGGGACAGGCACTCAGTGGACCCAAGCTGAATCTCTAGCAGTAAATTCTTGGACACAGCCTATAGCTGATACAGTCACAGTGTGGACTCAGGATGAGCCTCCATTATTACATTCCTGGAAAAAGTCTAAAATCCATACACTTACAACATGGACTCTGACAGAATCTTTAGCAGGAAATTTCTGGAGACCATATGAAACTGATAGTACCTCATTGTGGACCAATAAGGGAAATCTAGAAGTAAATCCTTGGGCACAATCTGAAACTGATGTAGTCACAGCTTGGACCCAGGCAGAAACTCCTGGAATAAATTCATGGCTACAACCTATAGCTGAAATAATTTCATCATGGACTCTGGCTGATTCTCCAGCAGTAAATCCCTGGACAGATACTGTGTCTGATAGTGTTATACTGTCGACCCAGGTTGAATCTCCAGTAGTAAATCCATGGTCACAATCTATTGCTGATGTGGACACACTGCAGACCCTGACTGATTCCATAGCATTAAATCCCTGGACAGAGCCTGTATCCGATACTGTTATACAGTTGACCCAGAGTGAACCTCCAGCAGCAATTCAGTGGACAAAAACTGTATCCAGTATAGTTACATCATTTACTGGGGATGAATTTCCTGCAATAGAGACCTGGATAGACCCTTTGCCTGATATCACATTGTGGACCCAGACTGAAACTCTAGTAGTAAATCCCTGGACAGAGACTGTAGCTTCCACTCTCACATCATGGACCCAGACAAAATCACCAACAGTAAATTCACTGACAGAGGTTGTAGCTGCCACAGTAATACCATGGAATCAGGCTGAATCTGCAGGAGTAAATCCCTGGATGGATGCTGTAGCTTTCACAGTATCACTGTTGACTCACGATCAATCTCCATCAGTGAAAACCTGGACAGAGGCTGTACCTTTCACAATCACACCACTAACTCATGCTGAATCTCTGGCAATAGTGTCTATGACAAAGGGAGTATCTGATGCAATCTTATTGTGGAACCAAGCTGAATCACCTCTAGTATATCCTTGGACACAGTCTGAAACTGATGCAATCATACAGTGGACTCAGGGTGAATCTTTAAAAATAAATCCTTGGACAGAACCTGACACAATAACACCATGGACTCGTGGTGAGTCCCTAGCAGTAAATCCTTGGACAGATGCTGCAGGTGACACAGTAACACAAATGAACCTAGTTGAATATCCTTCAGTACATCCCTGGACAACATTTGAAATGGATAATGTCACATTCTGGACCCAGGCAAATTCTGAAATCATAAATCTATTGACACAGACTATAGTTGATATAGTCACACTGTGGACCCAGGCTGAATCTCCTGCAGTGAATCCCTGGACACAATCTGAAACTAACACAGTCACACCATGGACCCAGGCTGAATCTGCTACAATAAATCTCTGGACCCAGGCCAAAACTAATCTAGTCACACTGCAGACCCATGGTAGATATCCGGCATTAAATCCCTGGGCAGATCCAGAAACTAACACATTTAGATCATGGTCTCAGGTTGAGTCTTCAAGAGTAAATCCATGGATACAGTCTGAAACTGACACAGTCTCACCATGGACCCAGGCTGAATCTGCTACTATAAATCTCTGGACACAACCTGAAAATGTTATAGTCACACTGTGGATCCAGGCTGAATCTCTTGCAACAAATCCCTGGACACGGCCTGAAAATAACTTAGTCACACCATGGACCCAGAATGAATCACCAGAAGAAAACACCTGGACAGAGGCTTTTTCTGAAACATTCATACCATGGACTATGGGATTTTTTCCAACCATAAAGCCTTGGATAGAGACTGTATCTGTTAAAGTCATACCAGGAACCAAATATCAATTTTCAGAAGTAAAACTTTGGACACTGGAGTTGTCCAGAACATTGGACACTGAGATAGGTACGGTCAAAATGTGGACTCAGTCAGAATCTCCATCCTTAATTCCCTGGACAGAAGCTATAGCTTCCATAGTCCCACTGTGGACTCAGGCTGAACAACTAGCTGTAAATCAAAACCCTAGAGCTGATAGGGTCACAAAATGGACACAGACTAAATCTCCATCAGTAAATACTTGGACTCAGGTTCAGTTTCCAGCCATAAATCCATGGTCACAGTCTGAATCACCAGAAGTAAATATCTTAACAGAGTACAAATCTCCAGCCCTAAACCCATCAGTTGAGCCTGAGGGTAGTATAGGCACATTGTGGATTCAGACTGAATCTCCATTAGTAAATCCTTGGATAGAGCCTGTGGCTTCCATAGTCATTCCATGGACACAAGCTGAATATCCAGCAGTAAGTTCATTTACACAGGCTGTGGCTGATACAATTATACTGTGGGCTCAAGCTGAATCTCTAGAATTAAATCCCTGGACAAAGTCTATAGCAGATACAGTCACAATGTGGGTCCTGGATGAATCTTTAAGAGCAAAACCTTGGACACAGATGTTAGCTTCATCAGATAAAATTTGGACCCAAGCCAAAATAGTAAATCCATGGTCTACTTTTGGAATATTTCCACAATTCACCCAAATTCAATCTGTACTGGTTAAAACTGGAACACAATTTGAGAGTGACACAGTCACAGCATTAACTCAGGGTCAGGCTCTTGCTCTGAATCCCTGGACAGAGATTGTGACTTCCAGAGACATTCCATGGACACAAGCTGTACATCCAGCAGTAAATCCCTGGACAGAGGCTAGTGCATCCAGATTCATTTCATGGACCCAAGCTGTGCCTCCAGCAATAAATCCCTGGACTGTCACTTCCACCCAAGCTGAACCTCCAGAAGTAAATCTCTGGACAGAGGTTGTCACTTTTAGAGCCACTCCATGGACCCAAGCTATACCTATAGCAATAAATATCTGGACAGAGGCTTTCACTTCTAGAATCATTCCATGGACGCAAGCTGTACCTCCAGCAGTAAATCCCTGGAGAGAAGCTATCACTTCCAGAGTCACTCCATGGAACCAAATTGTACCTCCAGCAGTAAATCCCTGGACAGAGGCCATCACTTCCAGAATCACTCCATGGACCCAAGTTGTACCTCCAACTGTAAATCCCTGGACAGAGGCCATCACTTCCAGAGTCACTCCATGGACCCAAGTTGTACCTCCAGCAGTAAATCCCTGGACAGAAGCCATCACTTCCAGTTTCACTCCATGGACCCAAATTGTACCTCCAGCAGTCAATCCCTGGATAGAGGCCATCACTTCCAGAATCACTCCATGGACCCAAGTTGTACCTCCAGCAGTCAATCCTTGGACAGAGGCCATCACTTCCAGAATCACTCCATGGACCCAAGCTGTACATCCAGCAATAAATACCTGGACAGAGGCTAGTGTATCCAGATTCATTCCATGGACCCAAGCTGTATCTCGAGCATTAAATCCCTGGATAGAGGCTATCACTTCCAGAGTTACATCATGGACCCAAGATGTACATCCAGCAGTAAATCCCTGGACAGAGGCTGGTACATCTAGAGTCACATCATGGAACCAAGGTGTGTTCCCAGTAATAAATCCCTGGACAGTGATGCTTGTTTCTACAGTCACACCATGGACCCAGGTTTCCTATCCACTAAATCCTTTGATAGAGACTAAAGCTTCTACAGTGAGAATATGGGCTCAGAATGAATATTCATTAATAAAAACCTGGACACATTCTGCTATTTCCATGGTTACATCCTGGACTCAGACTGAATATCAAGCAGTAAATTCTTATATACCAAGTGCAGCTGATATGGTCATATTTTGGACAAGGCTTGTATCTGAGTCTAAGAAATCCTTGATACTGCCTGAAGCTAGTATATTCAGTATTTCATTGCATCCTCAAAGTGATACTACTCAATCCTTGATTCAAGTTGAAAATCAAGCATCTCTTCTGTCAACATATCCTGGAATTAATAATATCAATATATGGACTTTACTTGAATTTGAAACACAGGTATCATGGATAGTGCCTTTCTCTCAAGAAGCCAGACTCTCACCTCTATCTGAAATTGATATTAGCATATATTGGTTTAAAACTGAAACAGAGAGAGTAAGAACCTGGGCCCACTCAGAATTTCAAACAGTGAGTACTTTGACACAGTTTGAAATTGGTGGATTTGAGCCCTTGGCCAAACATGAAACTCCTGCAGTCATATCATGGGTTCCAACTAAAACTGGTGTATTCCCCCTCTGGAATAAGTCTGAAAGAGACAAAATAAGAACCTGGACCTTTTCTGGAGGTGATGTCTTGCCACCATGGAGTCAGATTGAAGCTAGTATATTCAGCCTCTGGATCCAGTCCAAAAGTAGTACAGTCACACCCTGGACCCAGGCTGAGACTCAGTCAGTCAGTATCTGGACTGAAGGAAATTCAGGCACATTTTGGTACCTGAGTCAAAATAATGTAGTTAAGCCCTGGTCCCACCTTGAATCTCAAATGACATCTTCCAGGATTCAAAATGGTATAAATAGTTCTTGGACTCAGTATGAAACTAGTACAGTCAGATCTTGGACCAAGCTTGAAATTAGTACAGTGCAACCTTGGAATCAATTTGAAACTGTTACAATTAGATCATGGACTCAGTCTGAAAATGTAGACATATACCCCCTTACCCAGATAGAAGGTGGTACAGTAATAAGACATTGGTTCCAGACTCAAATGGATTCAATAAAACCTTGGAACCAGTCTGGAACTAATACAATTAGATCATGGACCCAACTTGGAACTGAAACAATCCAAATTTGGACCCAGACAGAAAGGCAAATAGTAAAACCTCCAACTTTATCTGAGATTGATACAATTACATCTTGGTTACAGACTCAAAGTGATACAAATAGACCCTGGATTAAATCTGACTCCTTGTCTGTCAATCCCTGGAGTCAGGCTGAAGTTGGTACAAATCACCCCTGGACTCAGCAAAGAGGTACTGTGAATCAACCCTGGACCTACTCTGAAATCCAGAAAGTCAGACCTTGGATGAAGCTAGAAGCTGATGCGCTTAAATCTTGGTTCTACATGCAAATGAATAAAATCAGACCATGGACCAATTCTGAATCTCAGATCTTCAGCTCCAGGTTGCAGCCTGAAATTGGTATGGTTCACCCTTGGATCCATCCTGAAACTCAAGCAGTCAGATCCTGGGCCCAACCTGAAACTGGTATTATTGCATCCTTTGCTATTCATAAACATGACAAAATCAGAACATGGATCCATACTGAAGTAGAAATCAGACTTAAAAAGCATTATAAAGCTTATAGAATTATATCATTTGCTCCCTCTGAGGTTGAGCCAGATGGAGCAACTCTCTTAACTACCCATTTTGGTTCCTGGTCTAAACATGTACCTTTTTTTACCAATAGAAGTAATTCCTTCCCCACATCAGTATTTTATAACTTTGAGATAGCCACACTTTTAACAAAAGAACCTTCTCTGATGACTGGATCTCAATCTGGAACAAAGTCTAATCAGCCTGAAGAAGATCCTCTCAAGTATTCAGAATTCAATGTTTCTTTAGCTGAATGTCGCCTACGTGTGGTCTGGAAAGAGAGTGTCCAGGCTTTCTGGCTCTTCAAGACAGCTGTTATTTCTCATGAAGCCACAGGCAAAAAAAACTCAGAAGCACTAGCCCTGGTCCAGGTGGGACTTATTGATCTTCAGGAGCCTGCTCAAGCTCAAACTGTAGGCATTCATCGTGCCATGCCCTACCTAGGTCCCAAAGGACCTTTGGGTCCTGGGCTAATCTTCCTGAAACAGCCACTACATTTTCAACCACTGGTGCTTCCTATTTGCCTAGAGGAGAACCTGGAGCAAGAGAAAAATATACAGCTGTACGACTGCTGGTTACCCAGTTGGTCCCTCATGAGAGGAAGTCCTGGAATTCTGCAAAAAAGGCACCTAAGCATTCTGCAAGTCAGCACATGTGCCCAGTTTTGGCCCAAGCTGAATGAATTCACTTTCTGTGTGGAAGCCAAGAAAGCTATGGGCGAGGCTGGCTGTAAGGGTGACTTAGGGGCACCTCTGGTGTGCCATCTACAACAAAAGGACACATGGGTGCAGGTGGGAATTTTGAGTCACTTCGATGAACATTGCACAAAGCCCTACGTCTTCAGCCAAGTGAGCCCATTCCTTTTTTGGCTTCAAGGAGTTACACGACCCAGCCATGCACCATGGTCACAGCAAGGGACCATGACTACTTCTGCTTCCATCTCCCTTTCAGTCTCTACTTCAATGAATGCCTCGGCTTTTACTGCCACTCCTGCTTCTATTCCGCCTCAGTTCATCTCTCTGCTGCAGCCTCAGACTTTAGCAGATCGAATTTCTCTACGATATACCATGCCTTGGCAAGCCATGATCATTAGTTGTGGCAGTCAGATTTGCAGCGGTTCCATTGTTAGCAGCTCTTGGGTTCTCACTGCTGCCCATTGTGTCAGGAACATGAATCCTGAAGATACTTCTGTAATATTGGGCCTGAAGCACCCTGGAACATCTCTGAGAGTTGTTAAGGTGTCTAGCATTCTACTGCATGAGAGATTTCGGTTGGTGAGTGGGGCAGCAAGAAATGATCTAGCACTGCTGCTCCTTCAAGAGGTCCACACTCCCATTCAGCTCTTAGCACCACTGGGACATCTAAAGAACTTCAATAGCTCAGAATGCTGGCTTTCTGGGCCAAGAGTTCTCAAACCAGGAGAGACTGATGAGGATCCGGAAGTGTTACAGATGCAGGTGATGGGAGCTTCAAGTTGTGCCCACCTCTACCCTGACATTGGCAGTTCTATTATCTGCTTCATTACTCAGGCGAAAGGCTTAGATACAAATGTGGAACCTGTGACTCCTGGAAGTGCTGTTATGTGCAGACCAGTGTCTGGAAATGGCAGCTGGAGACAGATAGGCCTTACCAGTCTAAAGGCACTTGCTACCATTGTCAGCCCACACTTCTCCTGGATATTATCCACATCAGCAAAAGCAGGTCATCCCCTAAACCAGGCAGTCATGCCTTGGGTGGAAAAGCTAAAGTCATCCAGTCTTCATAAACAGGCAACAACACTACCACTTTCATCATTAATGATTCTCGCAATGCAGAATCTGTTGTAA

>Sheep, Ovis aries (BK059507)

CTCAGGATGACAGTCCAGGTGTAATTACCTGGGGAGAGACTGTTCCTTTCAGAACCACATCAAGGACACCGATTGAAAGTCCAGATATAAACACCTGGAGAGAGACTGTGCCTTTCACAGCCTCACAATGGACTCAGGCTGAACCTCCAGCTATAAATTCTTGGAGAGAGATGATACCTTTTTCCATCCCACCATGGACACAGGATGGAAGTCCAGGTGTAATTACCTGGGGAGAGACTGTCCCTTTCAGAGCCCCACCAAGGACGCAAATTGAAAGTCCAGATGTAAACACCTGGAGAGAGATTGTGCCTTTCACAGCACTACCATGGACACAGGCTGAAGGTCCAGATGTAAATACCCAGAGAGATACTGTGCCTTTTACAGGTCGGACTCAGGCTGAAAGTACAGCTGTGCATACTGGGAGAGATACTGTGCCTTTCACAGCTCCACCATGGACTCAAGATAAAGGCCCAGATGTAAATAACAGCAGTGAGGTGCTGAGTTTCACAGGACCATCATTGGCACATGCTAAAGGTCCAGCTTTAAATAACTGGAGGGAGACTGTACCTATAATAGGCTCATCCTGGACTCAGGCTGAAAATCCAACTGTAAATGCCTGGAGAGAGAATATGCCTTTAACAGCCCCACCCTGGTCACAGGTTGAACATCCAGCTATAAATACATGGAGAGAAACTGTGCCTTTCACAGAGCCACCATGGACTCAGGCTGAATATCCTGCTGTAAACACCTGGAGAGAAACTGTGCCTTTCTCAGCTCTACCATGGACTCAGGCTGAAAGTCCAGCTGTAAACACCTGGAGAGAGGCTATGCCTTTCACAGACCCATCATGGATTCAAGAGAAAAGTCTAACTGTAAATAGCTGGAGACAGATTTTTACTTTCCCAGCCCAACCATGGCCACAGACTGAAAGTACAGTGGAAAACGACTGGATATGGAATGCCCCTTTTACAGCTCCACCATGGTCACACACTGAAAGTTCAGCTGTAAATACCTGGACTGAGCCTATGCTTTTTATAGCCCCTCCATGGACTGAGGCTGAAAATCCAGCTACAAATACCTGGAAAGTGAATATGCATTACAGAGACACACTATGGCCTCAGTCTGACTTTGCACCAGCAAACCCTTGGACATCAACTGAAAGTTTCAGAATCACCTCATGGACTCATACTGTAAAGCAAGTTTTAAATATTTGGACAGAGCCAATAGCTTCCACAGCCACACTGTGGACTCAGGCTGAATATTCAACACCAACATATTGGACAGAGATTAAGGCCATTTATATAGTCACACCATTGACCCAGTATCAGTTTTCAATAAATACTTTGACAAAATCTGTAGGAGCCATAATCACACTTTGGACGTCTGCTGAATCTCTGTCATTAAGTTCTTTCACACAGAATAGTATCGATACAATCGAATTTTGGCCAATGCTTAAAACTGAGTCTAAGAAAAGGTGGAATCTGCCTCAAACTAGTACATTCATGTTTTCACTAAATCCTCAAATTGATACTTTTGGATCCTTGAACCAAATTGAAAATCAAGAATCTCCTCGGTGGGCCCATCCTGAGATTGATAATGCCAATACAATGGCCTTTCTTGAATTTGGAACACTCATATCACAGGTAGTACCTTTGCCCCAAGCAGCTAGACTCTGGCCCCAAACTGAAGCTGCTAATAGCAAAATTTGGTTTGTATCCTCTGAAAGAATAAATTCCTGGGACCAATCAGAGTCTCAAAGAATGAGTACCTCAATCCATTTTGGAGTGGGTAGAGTGAAGCCCCTGGCCCAACATGAAACTGCTATAGTCATGTCATGGCTTCAGATTGAAACTGGTATATTCCACCCTTGGAACAAGTCTGAAGGAGGCACAGGGAGGTTCTGGCCCCTTTCTGAAACTGAGGATATAAGAGAATGGATCCAAACTGGAGCCAGTACAGTTAACTCTTGGACTCAACTGAGAACTAATATAGTCAGAGCTTGTCCCCAAGCTGAATCTGAATTAGTCAGACCCTGGACACAAGCTAAAACTAATGCAATCACACTATTGACCCAGACTGATGCTATCAAACCTTGGTTCCAAACTAAAATTAATTCACTAAGAGAAGGGACCCAAACTCAATCTCAAATTGTTACTACTTGGATCCAAACACAGTTGCAAATATTTCACCCCTGGATTCAGCCTAAAAGTGATTCAGTCAGATTTTGGACCCAGCCTTGGATCCAAGCTGAAACCCACACAGTCAGACTCTATTATGAAATTGATGTAAGAAAATCATGGGCTTCATCTGAATCTCAGTCAGTCACATTTTGGTCACTGAGTCAAAATTCAGTTAGGACCTCATTTCACTTTGAATCTCAGATGACATGTTCCTGGGTCCGAAATGAATTTGATATAATCAGTCCTTGGAATCAATATGAAACTAGTTCTGTTGGATCCTGGATCCAGTCTGAAACTGGTACATGTCAACCCTGGTTCCATATTGAATCTTCTACAATCACACCATGGACCCAATATGAAACTTTAGAGATCTCCCCTTCAACCCATCCTGAGACTGATACAGCAATAAGGCATTTGTTCCAGCCCCAAATTGATCCAATTAGTACTTGGAATCAGCCTGAGGTAGATACAATCAGATTCTGGACCCAAGTTGAAACAGAAACAATTCCAATTTGGATCCAGATTGGAAGTCAAGTAGTTAAACCTCCCAACTTTTCTGAAGTTGGTATAGTTACACCTTGGCTAAAGACTGAAACTGATGCAATTATTCCCTGGATTCAGTCTGACTTTCAGTCAATCCATCCTTCGACCCAGACTGGATTTGGTATAATTGACCCCTGGTTTCAGCCAAGAGCTTCTGTAAATCAACCCTGGACCTTTGTTCAAACACAGTCAATCAGACCTTGGATTAAAGTGGAATCCAATACAATCAAATCTTGGTTTCATGTTCCAATGAAAAAAGTCAGACTGGGGATTCCTTCTGAGTCTCAAATATTGAGTTTCTGGTTGCAGTCTGATGTTAGTAGAGTTAATGCTTGGATCCAACCAGAAACCCAGGCAGTCAATCCTGGGGCTCATCCTAAAACTGGTAATGTTGCATCCCTGACTATTCCTAAGCCTGAAAGAGTCAGAATGTGGATCCAGCCTGAAAGAGAAATGAGACCTGGCATCATTTATAAAACTAATATAACCACATCATTTGCTTCTGAAATTGAACCAGATGGAACAATTAGTCATTTTGATTCCTGGGCTAACCATGTAACATTTTTACCAATAGAAACTGTTCCTTCCCTAGATGAGCATTTTGCAGCTTTGTCAACTGAAATAGCTGCAGTAGAAAGCCAAGGTCAAATAAATCCTGTCCAACCCAGTGAAATCACAAATATTATCTTTCTTACAGTTTCAAGCACACAGCTTCCTGGAGGAGCTGGTTACCTGAACTTTGGCAACAAATTACAAATTACCAATTCAAAAGGAAGTCCTAATGTCCCATCTAGTTCTCTCAACCCACTTTTTCCATCTTTTTCCTTTATTGTTCCTTGTTTTTTCCCATTTTCATGTTCTTTGTCCCTTACTTGTTCAGTCTTTTCTTCTTGCACATTTTCTTCACCATGTACTTTTCCTTCTTGCTCAGTTCTTCCTATTGTGGGTCTCTCTCCTGTTCCTCCCTTAGCTGCTTCTGATAGTTCTCTCCAGAAACCATCTTCCTCGAAAGTTATTGAAGACACCATTCTTTCCCATACTTTTTCATCCTTTCATGCTGCTCCAGCCACTCTTTTAACAAAGCAACCATCTCTGATGCCTGGATTTCAATTGGAAACCAAGTCTAATCAGCCTGAACAAGATCTTCCTAAGTATTCTGAACTCAATATTTCCCTTGCTGAGTGTCGCCTGGGTGTGGTCTGGAAAGAAAGTCTCCAGGCTTTCTCGCTCTTCAAGACAGCTGTTATTTCTCATGAGATCACAGAGTGTGGATTACGCCCTGGCCTTGTTCCACACTGTCCCAACTGCTGGGAGGCTGAAGTGGGTGAATTCCCTTGGATGGTTTCTGTGCAACTCTCTTTCTCCCATTTTTGTGCTGGTTCTATACTGAATGAACAATGGATTCTCACTACAGCTAGGTGTGCAAATTTCATAAAAAACTCAGAAGCACTGGCCCATGTCCAGGTGGGGCTTATCGATCTTCAAGACCCTGCTCAAGCTCAAACTATAGGCATTCATCGTGCCATGCCCTACCTGGGCCCTAGAGGACCTCTGGGGCCTGGTCTAATCTTCTTGAAGCAACCATTACATTTTCAACCCCTGGTTCTTCCTATCTGCCTGGAGGAGAGCCTAGAGCAAGAGAAAAATATACAACTGTATGACTGCTGGCTACCCAGTTGGTCCCTCATGAGAGGAAGTCCTGGAATTTTGCAAAAAAGGCACCTGAGCATCCTGCAAGCCATCACATGTGCCCAGTTTTGGCCCAAACTGAATGAATTTACTTTCTGTGTGGCAGCCAAGAAAGCTATGGGGGAGGCTGGCTGTAAGGGTGACCTGGGGGCACCTCTTGTGTGTCATCTGCAACAAAAAGACACATGGGTGCAGGTGGGAATTTTGACTCACTTTGATGAACACTGCACAAAGCCCTACGTCTTCAGCCAAGTGAGCCCTTTCCTTTTCTGGCTCCAGGGAGTTACACGACCTAGCCAAGCACCCTGGTCCAAGCAAGGGCCCATGACCACCTCTGCTTCAGTCTCCCTTTCAGTCTCTACCTCTACGAATGCCTCAGCTTTTACTTCCACACCTGCTTCTGTCCGGCCACATTTCATCTCTCTGCCACAGCCTCAGACTTTGGCAGATCGAATTTCTCTGAGATATGCCATGCCTTGGCAGGCCATGATCATCAGTTGTGGCAGTCAAATTTGTAGTGGTTCCATTGTTAGCAGCTCTTGGGTACTCACTGCGGCCCACTGTGTCAGGAATATGAATCCTGAAGACACAGCTGTAATATTGGGTCTGAGGCACCCTGGGGCACCTCTGAGAGTTGTTAAGATCTCTACCATTCTTCTGCATGAGAGATTTCGATTGGTGAGTAGGGCAGCAAGAAACGATCTAGCATTGCTGCTCCTTCAAGAGGTCCAGACTCCCATTCAGATTTTAGCACCGCTAGGTCATCTGAAGAATCTGAACAGCTCAGAATGCTGGCTGTCTGGGCCACGAATTCTTAAGCCAGGAGAGACAGATGAAAATCCAGAAATATTACAGATGAAGGTGATAGGAGCTTCAAGCTGTGCCCACCTTTACCCTGATATAGGCAGTTCTATTGTGTGCTTCATTACACAAGACAAAGACGCTGACACAAATGTGGAACCAGTGAGTCCAGGCAGTGCTGTCATGTGCAGACCAATGTCTAGGAATGGAAGCTGGAGACAGATAGGCCTCACTAGTCTGAAGGCACTGGCTACCATTGTGAGCCCCCACTTCTCATGGATATTATCCACTTCATCAAAGGCAGGACATCCATTAAACCATGCACTCATGCCTTGGATGGAAAAGCCTAAGTCCTCTAGTCTTGTAAAACAGCCAACCACCCTGCCATTTTGTTCAACAATAATTGTTATACTACAAAGGCTTTCATAACTCATTGCAAAAATAAGGCAGGGCTAATCTATTCAAACTATTCATAATAAAAATGTTAAACAATGTTAAAAAAAATTAAGACCCTATGCAACCTAGGAATATATGTCATGAATAAAC

>Squirrel, Urocitellus parryii

ATGCATCCCGGTTTTAATCAAAAAGATGGAACAGAATCAGTCAAAGGGGATATAGGAGTTGTTCTCTTGAAGTACCCTATTATGAGGAAAGAAATTCCACTTTCTCACACTTATACCATCTTCTGGAGGAACTGCTATAACTGCCTGCACAGACTTTGCAGAGTATACCAATATGAGAGATTTTCCGTACAGTCACAGAGAAGATTAGAAACTTTCCCTCTGAACAGACAGCGACGTAATCCACCTTTAGTATTTTTTGGCCCAAAAAAGAGAGACTCTTTTCTTCACAGTATACAACTATATTTGCAAAGTAGACAACTCTCTTTGTCTAGAAAATTCCTAGTGGCACAAACTTGGATCTCTCCTATTGTCAAAAGGTGGGAACCTTCTTACATAGCAGATTCAGGGAATACCCAATTAACTGATGCTTTTGAATCTTTGGTTATATCTGGACCCCAGAGCACCAAAAGAGCTGATATTCTAATCCCTTGGAAACTAGATCATATAGATGCAATGAAATATCAACATGAAACTTTGACTGATTTAGTGATACTCTGGGATAAACTTTTAGATGATGTAACTGCAGTTCACACTCTGCCATTAGTATATACTCTCAAATCCTCAATTCCTTATTTAAGTGACATTGACGGATACCTAGTATCTACTGAGATTAATGCTGCCTACTCTCATGTTAAATCTAGTATGTTTCCTAATTATAGTAAATTTGTAGCTAGACCCTGGCTAGAAATTACCCAGGATTTGGGGCCTTGGACATATTCTGTGCCAGTTAAAACAGAAAGAATATTGTGGAGTCAATATACTGAAGAGAATACTAGAACTCAAATATACCATGTACCTGAAACAGTAAACACTGATGCTAATCCAGAAAAATATAACAGGCATTTATTAGTCTCCTCACTAGTTAATACAATTGAATTACAAACTCATCCCATACTTAATGAAGATGGATCCTGGTATCCTACAGTAACCCAGACCTTTGAACTACCATTTCAGTCAGTTTTGGATATATATGGATCCCAAGAATCTGTAGAAAAGGCAATTAGACACTGGATTAATCCTGCATCTATGTCAACTTCTTCTGGAGATGATCATATTAGGGATTTGACCCTGTATAAATCTAGCCAGATCAGATCCTCCAGTAAAATGGATAGAATTAGTCTACTGAGTAAGCATGATACTATAATATTAGAACACCAGATGCATACTGATGGTAAAACATGGCTCTCCATCCGTCCTCTTACTAATGCAATTAAGCCTTTAATTCACTCTAAAGTTGATGTGAACAGACCCTGGAATCACCCCAAATCAGAGAGAATCAATTCAGAAACCCAAATAGGAAAATCCTTGACTCAGTTAAATGCTGATATAATCACACTGTGGTTACAAACTAAAATTGAAAAAATAAGGCCTTGGATTCAGCCTTACTTTAAAATAAGCAGCTCTAAAACCCAGACTGAAGAAGGTGAAAACAAACATACAAAAGCAGGTACAATTAAATTATGGACCAAAACAAAAATTGAAAACGTCAAAAACCCCAGGAACCAACATGAGTCTGAAACAGCTAGATCACAAAATGATCATGTGAAACTGGGGATCCAACCAGACTTTAAAACTCTCCAATCCTGGACACATTCTGACATTGATATTGTTAGACCATCGATTCGATATGATGTTGAAAGTACTCAACTTGAAACATTCATTTTCAAGCCTTGGATACAATATAAGGTTAATGGAGTCATACCCAAGACAAAGCCTGGAGTTTTGTCAGAAGTTTTAGTCAGAGTTTGGCTCCAGTCACAAACAAATACAGTAAAGATATGGAGCCAATTCAAATCTCAAACTAATGATGTGAAATCTTGGGATCATATACAAATGAATACATTAAAAAACATGCTGGAGACAGTATTTCAAAGGGTTCACCATTTGACCCCAACTGAAGATGCTATATCTAGAACTTGGTCTCAAACAAAGAATGTTAATGTCAAACTTTGGTTCCAGACTCCAGTGGAGACAGTCTCACAGTGGACAGTAATTCAAACAGAATACAACTTGATACAGCCTGAAACTGAAGTAGTCAGTCCCTGGATCCAACCTGTGATGGATAAGTTAAACTGGATACAACATAAAGGTTATTCAGTTTGGTCGTCTAATAAACTTGTAAGGGAAAATATTGGACTCTGGTCCCAGTCTGAAGTTGAAGTATTAAGACCTTGGTTTCATACAGACATTAACATAGTACAGACATGGATGAAAAATAAATCCAGTATTTTAACAGGAGGGACCCAAACTGAATCTCCAGAGGAAAATTTCTGGAGACAGTCTGAAACTGATAAAACCATACAATGGACAGAGTATGAAACACTAATAGTAAATTATTGGAAAGATGTGTTGTCTGGTATAGTCACACCCTGGATTAATACAGAACTTGGGGAACTAAAGTCCCAGATACAGTCTAATTCTGAAAAAGTCCCAGTGTGGAAACATGCTGAATCTCCAATTCTAAAAGTCCTAACACTGTCTGAAACTGGTACATTCAAAACATGGCCTCAGTATGAAACCTTCAGAGAATGGGTAGTCAATAATCTTCCAGCACTACATCAGTGGACAAAGACTGTAGATGCTGAAGAACAACTATGGTCTGAAGATGAATTGCGAGCACTAAATACCTTGACAGGGTCTAAAACAGATAATGTTAAATCATGGAATAAGGGTAAGCTTCCAGCAATAATCTTACAGACACATCCTATGTCTCATAAAATTGTACCCTGGACTTGGGCTATAGACAAAATAGTAAAACCATGGCCAGAAACTATAGTTCCAACAGTCACAAAATTGCAAAGGGGTGATTCTCCATTCATAAATCTTTGGGAAAAAACTTTAACTGATAAAATTACTTTATCATTGCCTAAGGATGAATATCCAGCAGGGAATATTTGCTCATTACCTTTGTATATGTCACCATGGGCCCAGAATCAACCTATAAATATACGGGCAAAGCCTGTAGCTAAGATAATCACACCACAATGGCTTCTGGATAAATATCAAACAATGAATCTGTGGCCAGTGCCTTTGGCTCATTCACATATACCACCATGGCCCCAGCCTGTATCTACAATAGAAAATCTATGGCCAGTGACTTTGTCTGACACAATTATACTTCCATGGCGCCAGGCTAAATATTCACTGGTAATTAAAGGGACTCAACCTGTATCTGAAGTAATCCCACCTTTGTGGACCCAGGTTGAATCTCTACCAGAATACCTATGGATAAAATCAGTTTCTAATATACTTAGACCACTGTGGCCCCAGGATAAAATTTCAACAGGAAATTCATGGCCAGAAACTTCATTTGATACAATTACATCACCTTTGCCTCTGGATGAGTCTCCACCAGAAAAACTATGGACTCTCCTTGTATCTAATGCAGTCACACTCCAATTGCCACAGCTGGAGACATCAAGAATAAATCTGTGGACACTGCTGATTTCTGATATTTCCATACCAATTTGGCCCCAGATTAAACATAAACCAGAAAATATATGGACACTGTCTTCACCTGATAGAGTTATTACTCTGTGGCCCCAGGTTGAATATCCACCTGTTAATGAGTGGACACTTTTTATATCTGATGAAATATCACATTGGCTGCAGGATGAATCATCACCAGTGAATCTCTTTAAAACTTATGTGTCTGATAGAACTATACCACTTTGGTCCCAAGATAAATTGTCACCAGGATATCTATGGCCATGGACTTTTTCTAAAACAGTTACCACATCAGAGTTCCAGGATAAACTGCCAATAGTAAATCTATGGACACAGCTTGTGTCTGAAACAATTATACCACCACAGCTCCAGGTTGATTATACACCAGCAAATGTATGGATATTGCCTTTGTCTGACACAATTATGACTCCATGGTCTCAGGCAGAATATCTACCAGTTAATCAGTGGACATTTTTTATATCTGATGAAATTATATCACATTGGCTGCAGGATGAATCATCACCAGTGAATCTCTTTAAAACATATGTGTCTTATAGAACTACACTACTTTGGCCCCAGGATGAATCTCCACTAAGATATCTATGGCCATGGACTTTTTCTACAACAGTCACCACATCAGAATTTCAGGATGAATTGACAACAGTAAATCAATGGACACAGCTTGTATCTGATACAATTATATCACCATTGCCCCAGGTTAATTATATACCAGAAAATGTATGGACACTATCTTTACCTAATGCAACTAGGTCTACATGGTTCCAGGCTGAATATGCACCAAGTAATCAATGGACAAAATTTATATCTGATGAACTCAGACCTTATTGGCTGGAGGATGAATCATCACCAGTAAATCTCTTTAAAATGTCTGTGTCTGATACAGTTTCATCACTTTGGCCCCTGAATGAATCTCCACTAGGATATTGGCCATGGACTTTTACTAAAACAATCATAAAATCAAAGTTCCAAGATGATTCACCAACAATAAATCTATGGAAACAACTTGTATCAACAATTGTACCACCAACATCCCAGGTTCAATACCCACTAGCAACTGTATTGCCACTGCCTTTGCTTGACACAATTCTGCCTCCATGGCCTCGGTTTGAGTACCCAATAGTTAGTCTCTGGACACTGCAGGCATCAGATACAATCATGCCATCTTGGCCTAAGACTGAATTTTCACCAGGAACTGTATTGACATTGTCTTTGTCTAATACAATCACCCCACTTTGGTCCCAGGCTGAATTTCCAGCAGAAACCTCATGGCCAAACACTTTGTCTGAAATATTCACTGCATCTAAACCCCAGATTGAAAAATCTCAAGCAAATGAATTGACACTGCTGGTGTCTGATACGATCATACCATCATGGCCCCAGACTAAATCTCTGGCAGGAAATCTGTGGACACTGTCTATGGCTGATGAAATCACACCACATTGGCCTCAGACCGAATATCCACCATTCAATCTATGGGCAATGCTGCCATTTGATACAGTTACACATCCTGGGTTCCAAAATGAATCTTCATCATTAATTCTATGGACAATGCCTGTTTCTGATACAATAGCACAACATAGGTCCCAGGCTCAATCTTTGCACATAAATCTGAGGACATTGTCTTTATCTTATACAATGACAGCATCTTGGCCCCAGGCTGAATTCCCAGGAGGAATTTTATGGCCACAGACATTGTCTGATACAATGGAAATATCCAAGGCACAGGCTGAATCTCGAGCAAATTTGAGGACATTGCTGGTATCTGATACAATTATATTACCATGGCCCAAAGTATCTGGAGATTCACCAATAAATCTAAGGACATTACCTGTTTCTGATAAAACTACTTCACTTTGGCTTCATACTGAATATCCACAAGTTAGTCTAAGGACCCTGCTTGTCTTTAAAACAGTCACACCATTATGGCCCCAAACTGAATCTCCAGCTAGAAATTTATGGTCACAGATTTTGTCTAAAAAGGTCTCAGCAACTTGGATTGAAGCCAAATCTCCTCCAATAAATCCATCACCTGTGCCTGTATCCATAGCAATCATAACACCACGGCCCCAGGCTGAAATTTCAGCAGGAACTATATGGGCACAGCTTGTATCTGATACAGTGATATCATTATGGCCCCAGGCTGAATCAATGGTAGAATATGCATGGGAACAGCCTGTATCTGATATGCTCATCTCATTATGGACCCAGGCTGAATCTACAGTAGTATATCTCTGGCCAATGCCTTTGAATGATACAATCACACCACTGTGGTCCCAGGGTGAATCTCCAACACTAAAGCTATGGGTAAATCATGTATCTGATGCAGTCATACTGCCATGGTTCCAGGCTAAGTCTACAGCAGTAAATCTGCAGCCACAGCTTTTGTCTGATACCATACTACCTTGGTCACATAATGAATCTCCACTAGTAAATCTATGGACACAGGCTGTAACTGATACAATCATATCACCATGGATTGAGAATGAATCTTCAGTAAGCAATCTGTTAGAGTCTGAAACTGATACAGTCACACTTCAATGGCCCCATATTGAATCTTTACCAGTCAATGTACAGTCAGAAACAATGTATATTGCAACACCACTTTGGCTCCAGGGTGAATATCCAGCAGTACATCCATGGACACATTTTCTGTCTGATAGAATCATATCAGCCTGGCTCCGTGCTGAATCTTCAATCATCAGTCTATGGATATTGACTATATCTGCCACAGATACCCCAAAATGGATCCAGGGAAAAGTTCCAACAGGAAATTTTTGGCCAAAATCTTTTTCTGATAGAGCCACACCATTGTGGCTACAGACTGAAACTCCACTAGTAAATCTATGGCCAGGGCTGGTATCCCACATGATAACATTATCTTGGCCCCTAGCTGATTTTCTAGCAATACATACTTCAACACTCTCTATATCTGAAACAATTACACCTCCATGGTCACAGAATGAATCACCACTAGAAAATCTATGGACATTACCTATGTCCAATATACTTAAACCACCATGGCTCCAGGCTGAATATTCAACAGCAAGTCCAAAGTTACCTTTGACATCTGAACCAAATAGATCATCACGGATCCAGGCTGAAAATTCAGTATCAATTCCCCTGACAGAGTTTACTGCTGAAATATTCAGGCCATGGACTCAGTCTAAATTTTTAACTGTAGAACCTGGCTTTCAGACTTTTTCTGATGTACTCACACTTTGGTTCCAGGCTGAATCTCCATCAGTAAGACATTGGACACAGGCAATATCAGATGCATTTGCATTGTGGAACCAAGCTACTTCACCTGTAATATATCTCTGGACAAGGTATGAGATTGATACATTCAAAAAATGGAGTAATTCTCAATTCCCAAGAGTAAAGCTCCCAACACCTCCTGTATCTGATATATTAAGTAAAGCCAAAACTGAAGCATCAATTCCCTGGACAAAACTTGAAACAGACATTGTCTCTCATTTGACCCAGACTAATACTGAAGTCATAAATTCTAAGATACAAGATCTAGAAAATAGAGTGTTACGATTAAGTCATGTGTTTTCATTAAAACCGAGGACACAATTTGAATCAGATACAGTCAGAACATGGATCCAAGCTCAGTCTCCAACATTAATTCCTTGGACACAGACTGAAAAGGACACAATCACTTCATTAACTCAAGCCCTAAGACCAGCATTGAATGTCTTAACAGTATTTGTGACTGGTACAGATATGCTGTGGAACATCATAGAAGTTATAAGACTAAAGGACAGGGAAAAGTTGACAACAGAATTAGGTTTACAATGGACACAAAATAAGTCCACAGCAATGAATGCTTTTATACTGCCTATGTTAAATACAGTCACTCTTTTCACACAGGCTGTTAGCTCAGTAGAAAATCACTGGACACAGCTTGAAGCTCACAAAGTGACTCCACAGACTTATGTTAAATCTCTTAAAAAATATGCCTTGACAGAATCTGATTCTGAAACAGTGACATCATGGAACCCAGATGAATTTTCAGAAGTAAAGACTCTATCAGAGGCTGATACACTCACATTGTGGCAACAAACTGTATCTAAAACAGTAATTCTTTGGATAGACACTATAGCCAAAACAGGGAAATTGTGGACACACAGTCAATCTTCAGCATTAAATCCTTGGACACAGTTTGTCGCAAATAAAACTACAACGTGGATACAGACAGAACCTCCAGCTAGAAATCACTGGGTACCACCCATCTTTAATAATATCACACTAAGGACACAGGCCAAATCTCCATTAGTAAATACGTGGATCCAGGAAGAAACAATAGATCCTCTGATAGCGTTGGAATCTCCAACAGCAAGTGTCCATACAGAATTTGAAACTCTAGCCCCAACTCCTTGGTCAAAGTCCAGGAGTAGTAGAATCACACCATGGGTCTATACAGAAAATCACTTGACAACTAAAAGAGTCATATTGTGTACACGGTCTGAATCTACAAAATTTAATTCTTTTTTACAAAAGGGAAATAAAGTAATAGCATGCTCTCAAACTGAATTTTCTAGAATAAATCCTCAAAAGAGGGCTAAAGTACCTAAAGTTATACCTTGGACACAGGCTGAATCTCCATCAATAAGTCCCTGGACAGGGGCCACAAAGTCAAAAAGCATACCATGGACCCAGACTGAATATCTACAAGTAGATTCCTGGGAAGATGCCACAGTTTCAAAAGTTGTTCCATGGATCCAGACTGAATATTTTCCAACAAAACCCTGGATTGAAGCAATAGATTCAGAAGTTATAACCTGGTCTGAGGCTAAATTTCCAACAGTGGGTCCATGGATACAGCCTATGGGTTCTACTGTCATATTTTCGGCAAAGGGAATATATTCAATGGTAAACACATGGGCACAGACTTCATCCACCACATTAGGAACTCATTCTGAATCAGAAGCAGTAAATACTCATATACAACCTATATTTGAAACACTTATACCACTGAGCCAGGATGAATCTAGAATAGTAAATTCCCGGGTACAATCTTTAGCTGAAAAAGTTACAACATGGACCCAAATTGAAACTCTAGCAGTCAAGGCTTGGTCAGAGGCTACAGTTTCCAAAATCACAACTTGGAAAAAAAGAGAGTCTTTATCTGGTAAATCTTATACACAGATTGAAAATGACACAGGGATATTTTCAAGAATGCTAACACAGGGGGCCACAAAACCTCAGAGATCAACTGAAATTAATGCATTTAGGATTTTGATGCAATTTCAAACTGATGCTACTCAACACCTGAATAATGAATCACTTCTGGGGACACATCCTGAAATTGAAAAAGTCAAAACTTGGTACTTAGGGCCTGAGTTTGGAACTCTGTCATACTGGATAGTGCAACCTGAATCTTTTGTTAGCAAAGATGGTTTCAAAATTCAAACAGAAAGCATAAAACCATGGAAACAGTCAGACATTGAAATGCTCGGTAACTTTACCTCATTGAGATCTAATAATATAGAGTCCTTGGTCCAACCACAAAATATTATAGTTATATCACAGATCCAAAAGAAAACTAGCACAGTCAGTCCCTGGGCACAACCTAAAATGAATGCAAACAGAACATTAACCCAAGATGAATCTCCAGTAGTCAGGCTCTGGACTCAGACTGTGCCTAATATTGTCACACTGTTATCTGAGAGTGCAATGCAAACAGCAAAACATTTGGCCATGCTTGACATTAATAATGTCATAACTTTGTTCCAGACTCAAAATGACATAATAAAACGTCGGACCCAAATTGATTCTCAGATAGTTACTTCATGGGTTCAGCCAGAAATGCAAGTAGCTCACCCCTGGAATGAGCCTAAAATAGATATAGTCAGACCCTGGTCTCAGCTTGATGGTTATGCAGACCAACCATGGACCCACACTGAAGAAAATACAGTTGGACTCCAGACCTATTCTGAAACTGATAAAATACAATTTCAGACCCAGCCTGAATCTCAAGCAGTTAGGATCTGGCCAGAAGCAGATAAACCAACACTTTGGTCCTTGACTCGATATGATGCAATTAGACCTTTATCCCAAATAAAATTTCAAATATTACATTCAGGTGAAGTTGGTATAACTAAACTTTGGACTCAGAATGAAGTTGCTACTTTCAGCCCATGGACCTCGTCTGATACTCCAGAAATCCTTCCTTGGATCCAGCCTAATAGAGTAATTTCATATTTGTTTCAGTATCAGAAAGATTCATTCAGAACCCAGAACCAGGCTGAAAGTAAAGCAGTCCAAACTTGGACTGAGACAACCTTGATTAAAGTTGATACAACAAAACCTTGGATTCAAAGTAATACAACTAAACCCTGGATTCAAACTGATTCCTCCAAAATGAGTTCCTGGATTCAGACTGATGTTGGTATAGTCAGGTCTTGGACTCAGAAAAGAGCTGCTTCACATGATGCAGGGACCTACCCTGAAATCCAGGCATTCACACCCTGGGACAAACTGCAAGATGATACAGTCAGATCTTTGTTTAACATTCAAATGAATAAAGAAAGATCATTGACCTATTTAGAATCTCAAATATTAACTCAACCTGAAGTTGGTACAAAACATTCCTGGTTTCGGCCTAAAACGGAAGCAGTCAAGAGCTGGACACAATCTGAAACTTTCATAGTCAAATACCGGTCCCAATTTGTATATGAAATAGTCAAACCATTGTCTTCACTTGGAAGAAGAACTCTCAAATCTTGGATCCAATCTGAAACCCAACCAGATATATCATCCTTTTCCATTACTAACACTGATAAAGTCAGTACCTGGGTCCAGTCTGAAACAAAAATTCTAATACCTGGCACATATTATAAAGCTGATGTAATTGATTCATTCACTGTTTCTGATGTTGAGTCAGGTGAAGAGACCCTATTAAAGAGTCATTTTGGCATTTGGCCTAAAATTGTACCATTTTTACCAGTAGAAACAATTCCTTCCCTAGATAAGGGAGAAAATATATCTTTGTCAACTATAGCTACTATAGAAAACCAAGATAAAAACAATTCTCTACAACCCAGCCATACCACAAACACTGTCCTTCTTACCCTTTTAAGTGGTTGGATTCTTGGAAGATTTGGCTACCAGAGTGGTGGCAGCAATGTACAAATTACTCAGGCAATTGGAAACAATACACAGATTACCCAGGCAAGCGGAAACAATACACAGAATACCAAGGCAAGTGGAAACAATACACAGAATACCCAGGCAAGTGGAAATAATACACAGAATACCCAGGCAAGTGGAAGCAATACACAGATTACCCAGGCAAACGGTGACCTGGGGGCACCCCTGGTGTGTCATCTACAACAAAAGAACACATGGGTGCAGGTAGGAATCTTGAGTCGTTTTGATGAACGTTGCACAAAGCCCTATGTCTTCAGTCAAGTGAGCCCATTCATTTTCTGGCTCCAAGGAGTTACAAGACCCAGCTTTGCACCCTGGTCCCAGCAAGGACCCATGACTACTTCTGCTTCCATTGCCATTTCAATCTCCACCACTAGGAATGGTTCAGCCTTAACTTCCACTACTGCTTCTGTTCAGCCAAACTTTGTCTCTCTGCCACAGCCTCAGACTTTGGCAGACCGAATTTCTCTGAGATATACTATGCCTTGGCAAGCAATAATCATAAACTGTGGCAATCAAATTTGCAGTGGCTCCATTATTAGCAATTCTTGGGTTCTCACTGCTGCGCATTGTGTCAGGAACATGAATCCTGAAGATACTGTGGTGATACTGGGCCTTAGGTATCCTGGGGCACCTCTGAGAGTTTTGAAAGTGTCTAACATTCTACTGCATGAGAGGTTCCGGTTGGTGAGTGGGATAGCAAGAAATGATATAGCATTGCTGCTCCTTCAAGGAACTCCAACTTCCATTCAGACCTTAGCACCCTTGGGCCATGTAAAAAATTTGAACAGTACAGAATGTTGGCTTTCGGGGCCACGAATTCTTAAACCAGGAGAGACAGATGAGCATCCAGAAATATTACAGATGCACATGATGCAAGCTTCAAATTGCGTCTCCCTCTACCGTGACTTAGGTAGTTCTATTGTATGCTTCTATACTCGATCCAAAAATGCTGAAACAAATGCGGATCCAGTGAGTCCAGGAAGTGCTGTTATGTGCAGACCAATATCTGGTGGTAAATGGAAACAAATAGGCTTGACCAGTCTCAAATCTCTAGCTACCATCGTGAGCCCACATTTGTCCTGGATCTTAACCACTTCAGCAAAATCAGGACATCCCTTAAATCAGGAAATTATACCTTTAGTGGAATTGCCCAAATCTTCTTGTCCCCTAAAACAGACAAATATATTGTTACCTTTTTTGATAATGATTATTGTACTCCAGAGGTTTTTGGAGTTCAGAGATTAG

>Tree Shrew, Tupaia belangeri chinensis (BK059442)

TGATAGAGTTAATACTGGTGTTAAACCAGAAAACTATAACCTACATCCATGGGACTCTTTAAGTGTTAGTAAAATTGAAATAGAAACTCANNNTAACCACGATGGATCCCAAAACTTTATAGAAACTCATACTTTAAAACCATGGTTTTATCCAGTATTTAATATTTTTGATTCTCAAGAACCTATAGAAAATACAGATGAACACTGGATTCTCCCAGAATCTAAGTCAACTCAATTTTGGACTTCCTCTACATTTCGTATGTCTTTACTTTGGTCTCAATCTGTAAGTAACACTATTAGATCTTGGACCCAAGACAAAGTCAATAAAATCAAACCCTCACAACAAATAAATAAAATTACTTTCTTGAGTAAACATGAGGCTATTATACTAAAACCACAGGTCCAGACTGAAGCTACATGGCTGTTGATCTATTCTATTACTAATGTAAATAAGCCATATATCCACTCTAAAGTTGATATCATGAGGACCTGGATTCAATCTGAAACAGTCATAATCCAAACCTGGACCCATCCAGAAAGCCAAACAGGAAAACCCTTGTCTCAACTCAAAGCTGAGAAAGTCATTCCATGGTTACAAGCTGTATCTAAAAGAATTATACCCTGGATCCATCCTAAATTTCAAATAATCAGACCCAGAATCCAAACTGAAGAAGGTAAAGACAAAATTTTGACCTATCCAAAAGTAGGAATGATTAGATTTCGGACTCAGTCTGAAGTTGGTAGCATCCAACCATGGATAAAACTTGAAACATTTACCTTTAGATCCTCAATAGAATCTAAAGTTTATAAAGACATACACTGGACAAAACCTGAAGCTGACACCATCAGATTTTGGTTCCATACACTAACTAATATAATAATAACCTGGAGCCAACCTGAATCTCAGATGATCCTTTTCTTGTCTGAGACTAAACCTGCTAAACTTAGATCTTGGTTCCATAGACAAATAACTACATTCAAACCTTGGATAGAGACAGAATTTCAAATAACCCACCTAAAGACCCTATTTAAAGGTGATATTACCAGATCAATAATTCAGAGTGAGACTGACACTGTCAAATTTACTCAAATGGAGACAGTTACACAATGGACAGAGCAGTTAACTCAAACAGTTTACTATTGGATACACTCTATAACTGAAACAGACAGACCCTGGAACCTGTATGTGGCTGATGAATTAAGTGCTTGGAGTAAACATAACGCTTATACAATTAGGCCCTGGAATAAACATGAAAGTGATAAAGTGATATTCTGGACTCAGCTTGAAACTAGTACATTAAGATCCTGGATTCAGGCAGATGTTCGTGTAATCAACCCTTGGGTCCAACATGAAGCTAGCACATTAACAGAATGGACTCAGCATCATCTAGATTTAAATCCCTGGATACAATATGAAACTGCTCCAGTTACACTGTGGATGCAGGTGGAAACTGCAGAAGAAAATCCCTGGATACAATCCAGAACTGGCACAGTTACACTATGGACTCATGCAGAAACTACAATAGTAAATAATTGGACAGAAATGTTAACTAATACAGTCACAACACGGACAAGGACTAAATTTCAAGAACTAAATCCCTGGATACAGTCTATTAGCTCTTGGAACCAAGATAAAGTCAATATGAAAGAAATTGAGATGGCCACACTGTGGGCTCAGGCTGATTCTCTAGCAGTGAATCTACAGACACAATTTGTATCTGATACAGTCATACTGATACAGGCAGAATCTGCAGTACTAAATCCCTTCACAGTGTCTAAAATTGATACAGTCATGCTCTGGACCCAGGCTGAACTGCCAACAACAAAATCCTTGAAAAAATTTGTCAGTAATACTGTAAGATCATGGTTGCATTCTCAGTCTCCAGCAATAAACACCTGTGTTCTAACTATATTTGATCCATTCTTATGGTGGACCCAGTCTGATTCTCCAGCAGTATACCCCTATTTTCAGTCTGAAATGGATATAGCCACACTATGGACCCAGTTTAAATCTCTAGAAGCAATTCCTTATGAACAGTTTAAAACTGATACAGTTATACCAGGGACACTCATTAAAATGCCAGCAGTAAATTTATGGACACATCCTATGTCTAATACTGTCACAGTCTGTGCCCAGGCTAAATCTTTATTGAATCCTTGGACACAGTCTGGAACTGATATAATCATAACATGGCCCCAGGCTGAAATTACAGATGTGAGTTCCCACAGGTGCTCACCTGTAGCTGATACAATCATACAATGGACTCAGAATGAACATCCAGCAGTATATCCCAGGACTGAAGCAATGGCTAATGCATTTACATCACAGGCCCAGACTGATGTTTTATCAGGAAATCCTCAGGCACAATCTGATACAGTCACAGAGTGGGCCATGGCTGATTCTCCAGAAATAAATCTATGGACTCAATATGTATCTAATGCAGTCACTTTGTCATTCCTGACTGGATCATTAGTAGTACATCAGACAAAGCCTATGTCTGATAAAATCACACTGTGGCCCCAGATTGGATATCCAATAGTAAATCAACTGACACAGTATGTATCCAACACAATCATCTGGTGGCCACAGGCTAGTTTGTTAGCAGGAAATCTACAGACACACACTGTATCTGATGCGCTTTGGCCCAAAACTGGATCTCCTATAGCAAATCTATGGACACAGCATGTATTTAACACAGTAACATTGTGGCCTAAAACTGAAACTCCAGAAGTAAATCTGTGGACAAAATCTGTATCTGATACAGTCACATCTTGGCCTCACTCTAGATCTCTAATAGTAAATGTATCGACACAGAATAAATTCAACACAATCACACTATGGTCCCAGGATGAATATTCAGCAGTCAGCCTATGGAAAGAGCATGAATCCAGGACAATCACCTCTTGGTCTCAGTATGGCTCTCCAGAAATTAACCTAGAGACTCAGCTTGTATTCAGCATAATTACATTGTGGCCCAAGGATGAATCTCTAACTATAAATCGATGGACACAGAATATACTCAACACAATAACTCAAGGATCTCAGACTGGATCTCCCTTAGTAAATCTATGGACACAGCATGTATCTAACACAGTCAAGCAATGGCTTCAGGATGATTCTCCAAAAGTAAATCTATGGGCACAGCTTGTATCTGATACAGTTAAACTATGGTCTCATGATGGATCATCAAGAATAAATCTATGGATACAGCTTATGTCTTATGTAGTCACATCAACACCAAGGTCCCAGAGTGAATCTCCTACGGTAATTCCATGGATACAGCCTGTGTCTGATAAAATCACATCACAGTGGCCCCAGGCTGAATCAATAGCAATAATTTCTTGGAGAGAGCCCATCATCAATATATTCACATTGTGGTCTCAATATGAATTTCTAAAAGTAAAGCCTTGGATTAAGTCTGTACCTGATACACTCATACCATGGACTCATATTCTGTCTCCATCAACGAAGCACTGGACACAGGCTATATCCTCCATAGTCACACTATGGAACCAAACTGAGTCACCTCTAGTAAATCTCTGGACAGACTATAAAACATTCAGACAGTGGACCAAGGCTGAATCCTCCAAAACAAATCACTGGATTCAACTACTACCTGATACAGTAATATCATACCAAACTGAATTTCTGGCCATTTCATGGACAAAGCTTGAAACAGATGCTACACAGTGGACCCAGGCTAATTCTGAAATCATCAGTGCCTGGACACAGACTATAGTTGATAAAGTGACACCTTTGGTCCAGGGTATTTTTTTAGTGGTAAATCCCTGGACACAGTTTGAAACCAGTGGAGTTCCAATGTGGATAAAAGCTGTGTCTCCAATAGTAATTCCCTGGACAAAATATGAAAATGGCCCAGTCATATCATGGACCCAATCTCTATTGCCAGCAATAAGTACCTTAACAGGGGATTTGCCTGGTAGAGGTAAACTGTGGAACATGTTTGAAATTCTGTCGTTAAAGTCCTGGACACAGTCTTCAACAAAAATAATTATACAGTGGACTAAAAATGAGTCTCCTGAAGTAAATCTATTAACACAACCTGTAACAGATATAGTCATTCTTTTCACTCATGCTGTTAATTCTGCAGTAAAGCACCGGACACAGCCTGACACTAACATGATCATAACATGGACCCAGGCTAAATCTCTAGAAGTAAATGCATGGACCAAAATTATTTCTAAAACAGTCACACCATGGACTCAGATTGAATTTTCAGAAGTAAAGATTTGGCTACAGCCTGGAACTATTATAGATAGACTGTCACACCAGGATGTATATCCAACAGTAATCCCCAAAACAGAGGCTATAGCTAAGATAATCACACCATGGACAGAAATGGAATCTCCAGTACCAATTTCCTGGACACAGTCTGTAGCTGATACAGTCAAAACATGGACAAAGATAGAATTTCCAAGAGTAAATCCTCAGATGCAGTCTTTACCTAATACAGTTAGACTAACGATTCAGACTAAATCTCCAATAGTAAATACTTGGATTCAGGCCAAATTTGCAAATGTAAGCCCCAGCATACAGACCGAATTTTCAATATCAAATACCCAGATACAGCCTGTAGCTTATACAATCATACCATGTGCACAGACTGCATGTCCATTATTATCTTCTTTCCTGAAAACAGAAAATTATATAAACACTGAATGGACTCAGGAAGAATCTCCATCAGTAATTCCCTGGATGCAGATTGTAGCTACAAAAATCATACCCTGGACCCAGGCTGTGTCCCCACAAGTAAATCCTTGGACACAACCTATAACTTCCATAGTTACACAGGAAATTCAAGCTGAATATCTGGCATTACATCTCCAGCCTGTTGTTCATAAAATAAAATACTGGAGACATGTATCACCATGTCCAGTCACACCACAGTCCCCTTCTGAATCTCTATCAGAAATTACCTGGATAGAGCTAGTTTCTGATATACTCACTTCATTGACCAAGGATGAATCTTTAGTAGTAAAAGCCCAGATATTGGATGTATCTAAAACAGCAACGGCATGGACCCAGGCTCAATCTCCAACAGACATTGATTGGACATGGGCATTCACCCCATGGAGCCACATTTTATCTGTAGAAGTAATCCCTTGGGAAAAGGCCATGGCTTCCACAGTCACACAATGGACCCAGTCTGTATCTCCAGTAGTCATTCCCTGGACACAGGTTGTATCTTATATAATCACTCCATGGAAACAGACTCTATCTCCAGCAATAGTAACCAAGACACAGGCTTTGGCTTCCACTATGCCTTCAAGCTCTATCTCCAGCCATCATTTCTTGGTCATATGGACTCGGGCTCTACCTCCAGCAGTAATTCCTTGGGCACATTCTGTGGCTTCCAAATTCACACCATGGACCAATATTCAAGTTTTAGCAGCAATTCTCTGGACACAAGCTATGTCTGATACTGTAATTCGACAGATCCACTCCCTATCTCTATCTGTAAACCTCTGGACAGAAACCTTATATGATACAGTCACTCCATTGACACATGCTCTATCTCTCATTACAATTTCCTGGACACATGCTGTATCTTACACAGTCACACCATGGACCCAAGCTCTATTTCCAGCAGTTATTCCCTGGACATATGCTGTGACATCTACACTAATGACTCAAACTCCATCTATACCAGTAAATACTAGGACAAAGATTTTGACTTCTAAAATCATACCTTGGACACAGACATTATCTCAAGCAGTAAATCCTAGAAAACAGGCTAAAGCATCCACCATCAAACCATGGACCCAAGCTCTATCTTCAGTTATAAATTCCAAGACACAGAGTATAGCTTCCACATTTACAATATGGATGCAGGCTCCTACTTCTTCAATAATTCCCTGGACATATGCGGTAGTTTCCACAGTTCCATCATTGATCCAGGCTAAATCTACAGAAATCAGTCTCTGGACCCAGAATGTAGCTACTATAGTTACTACATGGGCTCTAACTCCAGCCATGACTCCCTGGGCAAATGCTGTTGTTTCCTTAGTCCCACCATGGACCCAGGCTCCAAATCTTTGGACAGTATCTATATCTGATACAGTTATATCATGGACCCAGGCTGTATCTCCTGGAGTAATTATGTCTTTAGAGCTTATATCTGATATAATTACACTGTGGATGCAATCTGAATCCCCTTCAATAATTCCCTGGACCCAGTCTGTATCTGACACACTCGCTTCATGGATACCTGATTATTCTTCAGCAGTAATGTTCTGGACAGACATGGCAGCCTCCACAATCACTCTGTGGACCCAAGATATCTCTCCAACAGTATATTTGGAGCCATATACAATAGCTTCCACAGTGTTTCCTTGGACTCAATTTCTACCTTCAACAATAAAGACTGAGGGACAATCTGTGATTGAATCAGTCACACAGTGGTCCCAGGCTGAGTTTTCAGGAGTCAACCCCTGGACAGAGGCTATAACTTCCTCAGTCACATTGTGGGCAAATACAGAGTCTCTAACAGTAATTTCTGAGACTGCAGATTCCTCAGTTACACCTTGGAAACAGACTTACTCTCCAGGTGTAAATTCCTATACACAGAGTGAAATTAATATAGAAACATTTTTAACAGAGCTGCTTAAAACTGAAATTAAGAAATTCTGGCCATTGCCTGAATCTTTGATATTAAGGGTTTCACTGCAGTTTCAAAGTGATAGTAACCAACTCTTGATTAAAACTAAAAATCAAGTATATCATCTGGGAACACATTCTGAAATTAAAAATGTTAATACATGGATTTTTCCTGAATCTGGAACCCTCATATCCTATAAAGGACCTATGTCTCAAGTAATTAGACCCTGGCCTCAGTCTGGAGCTCAAATGAACAGAATTTTGTTAACAACTCAGTCAATCATTCCCAGAACCCATACTGAAGTTTGGACCTGGACTCAACGAAGAATCATTACAAATCTACTCCTGGCCCATTCTGACACCCAGACAATGAGATCTTGGATCACACTGAAAACTGATATAGATATGTCTTGGTTTCACATTCAGCTGAATGAAAGCAGACCAATATCTGAATCTCAAATATTGAATCCCTGGATCCAGCCTGAAGTTAACATAGTCCATGCCTTGATCCAGCCTAAAACCCAAGTAGTCACACTGCAGGCTGATTCTGAAAATTTTATCATCAATTTTCTGACTCAGATTGTGTCTCGGGTAGCTGAATCATGGACTTTGCTTGAGGAAAGAGCATTGGAACCTTGGATGATACCTGCAACCCAGACTAAGGATGATAACTCATCATTTATTATTCCTGAACTTGAAAACGTTAGAACCCAGATCCAGCCTAAAACAGAAATAGCAATATCTAGAACCCATTATAAAGCTAATGTAATTGTATCATTTGTTCCTCCTGATGCCAAATCAGATGGGAAAACTCTATTGAGTCATTTTGACTCCTTGTCTAAACCTTTGTCCTTTTTACAAATAGAAACTATTGCTTCCCCAGATCAGTATTTTATAACTTTGTCAACTGATATAACTGCCATAGAAAACCAAGATAAAATAAATTTCCTACAACCTAGCCAGCACACAAACATTCTTTTTACCCTTTTAAGCACTTGGATTCCTGAAAGTGTTAGTTACCAGAATTTTGCCAGCAAATTACAAAGTATCAAAACAAAAGGACGTCATTTTGTCCCCTCTGCATTTCTCAGTTCTCTTGCTCCAGACTTTTCTCTTCTTATTTCTTATTCTGTCCCTTTCCTATGTACAGTGTTCTCTTCCTGTTCAGACTTTTCTTCTTACATATTTCCTTCATCCTGTATTTTCCCAACTTGCTCCTTCTTTTCACCTGTGGACTTCTCATATTTTCTTCTACCCTTACCCTTTTCTGATAGTTCTTTTCAGGGAATATCTTCCTCTAAATTAATTGATGAGGCCATTCTTTCTCATACTTTTACATCCCTGCATGATGCTCCAGCCATATTTTTAACAAAACATCCTGATATGCTAGGATCTCAATCTGGATCCACATCTATACATCAGCCTGAACAAGAAATTCACAATAACTCAGAACTCAATATTTCCTTGGCTGAGTGTCACCTGGGTATGGCATGGAAAGAGAATCTTCAGGCTTTTTGGCTTTTCAAGACAGCTGTTATTTCTCATGAAACCACAGAATGTGGATTACGCCCTGGCCTTGTCCCCCACTGTCCCAACTGTTGGGAGGCAGAAGTAGGTGAATTTCCCTGGATGGTTTCTATACAACTCTCTTTCTCCCATTTCTGTGCTGGCTCTATATTGAATGTACAATGGATCCTTACAACAGCTAGAGGTGCCAATTTCATAAAAAACTCAGAAGCTCTGGCTCTGGTTCAAGTGGGCATTACAGATCTTCAGGACCCTGTCCAAGCTCAGACTGTAAGCATTCACCATGCTATGCCCTACTTAGGTCCCAAAGGACCTCTGGGGCCTGGACTGATATTCCTGAAGCAGCCTCTACGTTTTCAACCACTGGTACTTCCTATCTGCCTGGAAGAAAGTCTAGGGCAAGAGAGAAATATACAACTATATGACTGTTGGCTACCTAGCTGGTCCCTCATGAGAGGAAGTCCTGGAATTCTGCAGAAAAGGCATCTAAGCATCCTACAAGTCAGTGCTTGTGCCCAGTTTTGGCCCAAGCTGAATGAATTTACTTTCTGTGTGGAAGCCAAGAAGGCTATGGGGGAGGCTGGCTGTAAGGGTGACCTGGGGGCACCTTTAGTGTGCCATTTACAGCAAAAGGACATATGGGTACAGGTGGGAATTTTGAGTCACTTTGATGAACACTGCATAAAGCCTTATGTCTTCAGCCAAGTGAGCCCTTTCATTTTCTGGCTCCAGGGAGTTACAAGGCCCAGCCATGCACCCTGGTCCCAGCAAGGGTCTATGACTACTTCTGCTTCCATCTCCCTTTCAGTCTCTGCATCTACAAATACCTCGGCTTTTACATCCATTCCCACTTCTATTCAGCCACATTTCATTTCTCTGCCACAACCTCAGACTTTGGCAGATAGGATTTCTCTACGATATGCTATGCCTTGGCAGGTCTTGATTATCAATTGTGGCAGTCAAATCTGCAGCGGTTCAATGATTAGCAGTTCATGGGTTCTCACTGCTGCCCACTGTGTCAGAAACATGAATCCTGAAGATACTGCTGTAGTACTGGGCCTGAAGCACCCTGGGGCATCTCTGAGAATTGTTAAGGTATCTACTATTCTACTTCATGAGAGATTCCGATTGGTGAATGGGATAGCAAGAAATGACTTAGCATTGCTTCTGCTTCAAGAAGTCCAGACTCCCATTCAGCTTTTAGCACCCTTGGGCCACATAAGAAATCTGAATAGCTCAGAATGCTGGCTTTCTGGACCACGGATTCTTATATCAGGAGAGACAGATGAAAATCCAGAAATGTTAAAGATACAGATGATGGATACTTCAAGCTGTGCCCATCTCTTTCCTGACATAGGCAGTTCTACAGTTTGTTTCATAACTCAGGTTAAAGGCTCTGACACAAATGTGGAGCCAGTGAGTCCAGGAAGTGCTGTTATGTGCAGGCTGATATCTGGCAATGGCAGTTGGAGACAGATAGGGTTCACTAGTCTAAAGACTTTAGCTACCATCGTGGGTCCACATTTTTCCTGGATTTTATCTACTTCAGTGAAAGCAGGCTATCCTCTAAACCAGGCTCTCACGCCTTGGGTAGAAAAACCCAAATTCTCTAGTCTCCATAAACAGTCAAACACACTGCCATTTTCATCAGTGATGATTATTGCAGCCCAGAGTCTATTGTAAACTACAGACTACAGTGAGTAGTGCTAATCTATTCACATTATCACATCATGATCAAAGTGA

>Wallaby, Macropus eugenii (BK059512)

TGGGTGTGGGATGCGGCCGGGCTTTGCAGCTCGGTGCCCCAACTGCTGGGAGGCAGAGGAAGGTGAGTTCCCCTGGGTGGTGTCACTGCAGTTCTCTCTGTCCCACTTCTGTTCGGGCTCCATCCTGAATGAGTGGTGGGTCCTAACCACCGCCAGCTGTGCCAACATCATACGAAATTCAGAAAGCCTGGCCCGCGTGCAGGCGGGCGTCAACAACCTTGAAGACCAGGTTCGGGCCCAACTTGTGGGTATCCACCGGGCCCTGCCATACTTTGGGATAGAAGGGCCCATGGGCTTGGGTCTCATCCTCCTCCAGGAGCCCTTGCACTTCCAGCCCAGGGTCCTGGCTGTGTGCCTAGAGGAGTCCCCGGAAAAGCCATTAGCAGAGTCTCAACTCCATCTCTATGACTGCTGGATCCCTGGCTGGACCTTGATCAAAGGGAACCTGGTCACAATGCAGAAGCAGCGACTGGATGTGGTTGAGGTTAGCAACTGTGCCCATTATTGGCCCATCAAGAACTTGAAGGCTTTCTGTGTGCAAGCCAAGAAGGTGATAGGCCAGAGCAGCTGCAAGGCCCTGGTGAATCGAATTTCACTGCGCTTTGCCATGCCATGGCAAGCTCTCATCGTGACCTGTGACAACACCATTTGCAGTGGGTCCATCCTCAGCCCTTCCTGGGTCCTCACCTCAGCCCACTGTATCCGGGACAT

>Walrus, Odobenus rosmarus

ATGGCTGAAACCTGCCAGGGCGTTATTCTGAGTCAGTGGTGGATCCTCTCCACAGCCAGCTGTCTGAATAAACTGAAACATTTGGACTCTGACATTTCAGCAGTCATTGACCAAGAAGATATCTTACTTGGCCATAAAATATGCCTGCACCCCAGTTTTGATCCACAAGTTGGAATGGATACAGTCAGAGGAGACATAGGAGTCGTCCTCCTACAGTACCCTATCAGGGTGGGGGGAATACCCCTTTATCACACTTATAACATCTTCCGGAAGAGCTGTTATAACTGCCAGTACAGACACTGCAGGGTATACCAATATCAGATTAATAAAAATGAATTCTGGACTCTTTCCACGCTAAATGCAGATGGATCTCAGTATCCTACAGTAACTCTTACGTTGGAACCCTGGTTTCAGTCAGTCTTAAATTTAGTTGGATCCCAAGAACTTACAGAAAAGACTAATGAATCCTGGATTATCCCTGAATCTACATCAGCTCAACTGTGGACTTCTTCAGCACTTAATATGCCTTTTACTTGGAATGAGTCTCCATTATTATATTCCTTGAAAAAGGCTGAAACTCATACATTGACAGCATGGACTCTGACAGAATCTTTAGCAGGAAATTCCTGGAGACAGTATGAAACTGATAGTGCCTCAATGTGGACCAAAAAAGGAAATCTAGAAGTAAATCCCTGGGCACAATCTGAAACTGATACAGTCACAACTTGGACCCAGGCAGAAACTCCAGGTGTAAATTTGTGGCCACAACCTGTAGCTGAAATAGTTCCATCATGGACCATAGCTGAATCTCCAGCAGTAAATACCTGGACAGAGATTGAGACTGTATTTGATGGTGTTATACCATGGACCCAGGTTGAATCTCCAGCAGTAAATCCTTGGACACAATCTATAGCTGATGTAGACACACTGTGGACCCTGACTGATTCTATAGCACTAAATTCTTGGACAGAGCCTATATCTGATAATGTTATACAGTGGACCCAGAGTGAACGTCCAGCCATAAATCAGTGGACACAAACTGTGTTAGATACAGTCACATCATTTATTGAGGATGAATTTCATGCAGTAGAGACCTGGACAGACCCTTTGACTCATGCCACACTGTGGACCCAGACTGAAACTCCAGGAGTAAATCCCTGGTCAGAGACTGTAGCTTTCACTGTCACACCATGGACCCAGACAAAATCACCAGTAATAAATCCACTGATAGAGGCTATAGCTGCCACACTATTACCATGGAATCAGGCTGAATCTGCGGCAGTAAATCCATGGATAGATGCTGTAGGTTTCACAGTAACACTATTGACTCAGGATCAGTCTCCATCAGTGAAAACCTGGACAGAGGTTGTGGCTTTCACAACTGCACCACTGACTCAGGCTGAATCCCTAGTAATAAAGTCTGTGTCACAGGGAGTATCTGATACAATCTTACTGTGGAACCAAGCTGAATCACCTCTAGTCTATCCCTGGACACAGTCTGAAACTGATGCAATCACACAGTGGACTCAGGGTGAATCTCTAAAAGTAAATCCTTGGACACAACCTTTGGCTGAAACAATCACACCATGGACCCCTTTTGAGTCCCCAGTAGTAAATCCCTGGACAGATGCTGCAAATTCTGAAATCCTAAATCCCTCGACACAGGCTATAGTTGATATAGTCACACTCTGGACCCAGGCTAAATCTCCTGCAGTGAATCCCTGGACACAGTTTGAAATTGACACAGTCACACCTTGGACCCAGGCTGAACTTGCTACAGTAAATCCCTGGACCCAGCCCAAAACTAATCTCGTGACACTGCAGACACATGGTAGATATCCAGCATTAAATCCCTGGGCACAGCCAGGACCTGACCCATTTACATCATGGTCTCAGGTTGAGCCTCCAGCAGTGAATCCATGGACACAATCTGAAACTGACACAATCACAACATGGACCCAGGCTGAATCTCCTGTAGTAAATCTCTGGGCACAACCTGAAAATGTTACAGTCACACCTTGGATCCAGGATGAATCTCTTGCAGTAAATACCTGGACACAGACTGAAAATAACACAGTCACACCATGGACTCAGAATGAATCACCAGAAGAAAATACCTGGACAGAGGCTGTTTCTGAAACAGCCATACCATGGACCATGGGGTTTTTTCCAACCATGAAGCCCTGGTTAGAGACTAAATTTGATAAAGTCACACCAGGCACCAAATCTCAATTTTCAGAAGTAAAACTTTGGACACAGCCATTCCCCAAAACATTGGACACTGAAACTGGTACAGTCAAAAGGTGGACTCAGTCTGAATCTCCACCCTTAATTCCCAGGATAGAAGCTATAGCTTCCATAGTCCCATTATCGACCCAGGCTGAATCTCTAGCTGTAAATCAGTGGACACAACCTGTAGCTTATAGAGTCACAGAATGGACACCGACTAAATCTCCATCAGTAAATACTTGGACTCAGGTTCAATTTCCGGCAGTAAATCAGACTGGACACAGTCTGAATNNTGTGCCCCACTGTCCCAACTGCTGGGAGGCAGAAGTGGGTGAATTTCCTTGGATGGTTTCCGTGCAACTCTCTTTCTCCCATTTCTGTGCTGGCTCTGTACTGAATGAACAGTGGATCCTTACCACAGCTAGATGTGCAAATTTCATNNNNNNNNNNNNNNNNNNNNNNNNNNNNNNNNNNNNNNNNNNNNNNNNNNNNNNNNNNNNNNNNNNNNNNNNNNNNNNNNNNNNNNNNNNNNNNNNNNNNNNNNNNNNNNNNNNNNNNNNNNNNNNNNNNNNNNNNNNNNNNNNNNNNNNNNNNNNNNNNNNNNNNNNNNNNNNNNNNNNNNNNNNNNNNNNNNNNNNNNNNNNNNNNNNNNNNNNNNNNNNNNNNNNNNNNNNNNNNNNNNNNNNNNNNNNNNNNNNNNNNNNNNNNNNNNNNNNNNNNNNNNNNNNNNNNNNNNTACTGCAAGTCAGCACATGTGCCCAGTTTTGGCCCAAGCTGAATGAATTCACTTTCTGTGTGGAAGCCAAGCAAGCTCTTGGCGAGGCTGGCTGTAAGGGTGACTTAGGGGCATCTCTGGTATGCCATCTACAACAAAAGGACACATGGGTGCAGGTGGGAATTTTGAGTCACTTCGATGAACATTGCACAAAGCCCTACGTCTTCAGCCAAGTCAGCCCTTTCCTTTTTTGGCTTCAGGGAGTCACACGGCCCAGCCATGCACCAGGGTCCCAGCAAGGGGCCATGACTATCTCTGCTTCCATCTCCCTTTCAGTCTCTACCTCTACAAACAACTCAGCTTTTACTGACACTCCTGCTTCTGTTCGGCCACAGTTCATCTCTCTGCNNNNNNNNNNNNCTTTAGCAGATCGAATTTCTCTAGGATATGCCATGCCTTGTCAAGCCAGGATGATCAGCTCTGGCTGCCAATTTTGCAGTGGTTCCCTTGTTAGCAGCTCTTGGGTTCTCACCTCTGCCCACTGTGTCAGGAACATGAATTCTGAAGACACTGCTGTAATACTAGGCCTGAAGCACCCTGGAGCACCTCTGAGAGTTGTTAAGGTGTCTAACATTCTATTGCATGAGAGATTTCGGTTGGTGAGTGGGGCAGCAAGAAATGATCTAGCACTGCTGCTCCTTCAAGAGGTCCAGACTCCCATTCAGCTCCTAGCACCATTGGGACATCTGAAGAACCTCAATAGCTCAGAAGGATGGCTTTCTGGGCCAAGAGTTCCCAAAGCAGGAGAAACAGATGAGAATCCAGAAATGTTACAGATGCAAGTAATGGGAGCTTCAGGCTGTGCCCACCTCTACCCTGACATTGGCAGTTCTATTATCTGCTTCATTACTCAGGAGAAAGGCTCTGATGTGAATGTGGAACCTGTGACTCCTGGCAGTGCTGTTATGTGCAGACCAGTGTCTGGAAATGGCAGCTGGAGACAGATAGGCTTTACCAGTCTAAAGGCACTTGCTACCATTGTCAGCCCACACAATTCCTGGATATTTTCCACATCAGCAAAAACAGGTCATCCCCTAAACCAGGCACTCATGCCTTGGGTTGAAAAGCCAAAGTCATCTAGTCTTCATAAACAGGCAACACTACCACTTTCATCATTAATGATTCTTGTAATGCGAGTCTATTGTAAACCAGTGGTTATAATGACCAGTACCAATCTGGTCACACTGTGATAAAAAAAAAAAGAGATCAGAACTTCCAATTAAAACAAAACAGTTGAATTAAGATGCT

>Wombat, Vombatus ursinis

CCAAGATCGACACCCCGCCTCCCCACCACACATACATTGCCCGTGCACACACACGTGCACGTACACCTGCACACATGCAGACAGACTTTCCATCTGCCTGCAGATGGAATTCAGAAAGCCTGGCCCGGGTCCAGGCAGGGGTCATCAACCTAGAAGACCAGGTTCGGGCCCAGCTTGTGGGCATCTGCCGGGCCCTGCCCCACCCTTTGATAGGTGTGCACATGGGCCTGGGCCTAGTCCTCCTCCAGGAACCCCTGCGCTTCCAGCCCAGAGCCCTGGCTGTGTGCCTCGAGGAGTCCCCAGAGAAGCCTCAGCTGCATCTCTTTGACTGCTGGGTCCCTGGCTGGACCTTGATCAAGGGAAACCTGGTCACAATGCAGAAGCGGCAACTGAATATGGTTGAGGTCAGCAACTGTGCCCAGTATTGGCCCATCAAGAACTCGCTGGCCTTCTGTGTGGAGGCCAAGAAAGTGATGGGCCAGAGCAGCTGCAAGGGAGACCTGGGATCCCCACTGATGTGCCGCCCAAAGCTGCACCTGGAGGAGACCCCCTGGGTACAGATGGGCATCCTCACCGCTTTCAATGAGGACTGCGTTCGGCCTTACGTCTTCAGTCGCATCGGCCCCTTCAGCCTTTGGCTCAAGGCCTCCACGAGGCCCCAGCACCCCCCCTGGGCCAGGCCTGTCCACAGGCCCACCCTCTCTTCCCTGCCCAAGCCTGAAGCCCTGGTAAATCGGATTTCGCTCCGCTTTGCAATGCCCTGGCAAGCTCTGATTGTGACCTGTGACAGGACCATGTGCAGCAGCTCCATCCTCAGCCCTTCCTGGGTCCTCACCTCAGCCCACTGCGTCCGGGACGTGAGATCAGAGAACATGGCAGTACTCCTGGGGCTGCCACAGCCTGGGGGCAACATGACAGCTGCACGGGTGTCCAGTGTTGTCCTGCATGAGCAGTACCAGGTGGTGAATGGGGTCCCTTGGAATGATTTGGCTCTCATCCTCCTGCAGAAGCCCCTAGGCCCTACCCAGCCCCTGGCCCCTGTGGGCCATGTGGAGGACATGCACAAAGCTGAGTGCTGGCTCATGGGGGCCCGTGAACTCCGAGAGGGTGAGAGGGACCAGTACCCACAAGTCCTCCAAGTTCAGGTGAAAGATGCTTTGAGCTGTGCGCACCTCTTCCCTGGCATCAAGAGCTCGGTGCTCT

>Human, Homo sapiens (model)

﻿AACTCCTGGATACAAAATGAAGCTATTATGTAAACACCATGGATGCAGGGAGAGTCTCAAGAATTAAATCCCTGGACACAATCTGAAACTCACACAGTCCCACTGTGTGAAACTCCAACAGTAAACTATTGGACAAATATGTCAGCAGATATAGTCACAACATGGACAAAGGCTGAATTTCAAGGATTAACATGCTGGAGACAGTCTGAAACTGATCCGGTCATACTGTGGACTATTGCTGAATCTCCAGCAGTGGATCTCTGGACACGTTTTGTATCTGATAAAGTCACACGTTGGAATCAAGGTGAATTTCCAGCCTTAATTCCCTTGACAGAGCCTATAGCTAAGACAGTCACACCATGGACCCAGGATAGATTCCCAGCAATGAATCCCTGGGCAAAAGCTATAGCTGAAACTGTCATACAATTGACCCTTGATGAAATGCCAACAATAAATCCTTGGACAAAATTGCAGCCTGAATATCTAGAAGGAAACAACTGGCTCCCATCTGAATTTAATTCAGTCTCATTGTGGACCACAGCTTATTTTCCAGCAGTCCCTGGGCACATGGTATATTACTATCACAGACCCATGCTGAACATCCACCAGTATCTTGTAGTCAACATGGATTCCAGGTAAATCTCCAGCAATTAATCCCTGAAGAAAGGCTATCACTGATATGGTCCTACCATGGAATGAGGGTGAATTTATAGAAGGAAATGCCTGGACACAGTCTGAAACTTACATAGTCACACAGGGAATCCTGAATGACTCTCCGACACTAATTTCATGGACACAGACAATAGCTGATGGAGACAGGCTGTGGACCAAGCCTGAATTTCCAGCCATAAATCCCTGGACAAACTAGTGGGTTTAGTACTATACCATGGATTCAGACTGAGTCTCCAACAGTAAATTCGTGCACAGATGCTGTAAATTCCAAAGTCACAACATGGGCCTAGGCTGTATCTCCAGTAGTAATTCCATGGACACAGCCTATATCTTCCATAGTCATACCTTGGACCCGGGTTGAATGTCCATCGGTAAATACCTGGACAACATCCATAGCTGATACCATCACACTATGGAAAGAGGCTGAATTAACAACAGAATATCCATAAATACTGCCCTGGGCTGCTACTGTCACACTATGGGCACATTCTCAATCTCCAGCAGGAAATCTTTGGACAAAGCCTACGAATGACACAGACACACCATGGCCTCAGGCTGAATCTCCAGCAGTAGTTTGTTGGACAGAACCTATAGGTGATACTACACAACCCTTAATCCAATATGAAAATCAAGCACTATTTTGTGGACACATCATGAAATTGAAAACATCAATGAATGGGCCTTGCCTGAATTTGGAACCCTTATATCCTTGGTAGTGCCTCTGCACTATCAAGCAGCAAAACTATGCTCCCAACCTGAAGCTCTAACTAGCAGAAGTTTGTTTAAAACGGCAACAGAAAAAAAATTAAACCTTGGGCTCAGCCAGAATTTCAAACACTGAGCACATTTACTCCCTTTGGACCTGGTAAAATAGAATCCTGGCTAAACACAGAACTACAACATTTATAACATGGATCCAATCTGAAACTGATGTCTTCTTCCTGTGTACCCAGTCTGAAGTAGGTACAATGAGATCCCAGAACATTTCTGAAGCTGATACAGTAAAACTATGGATCCAGACTGAAGCAGGCACCATCCAGCCCTGGACTAGAGCTAAAACTAATACTATCAGACCTTTGACTCATGGTGAATTTCAAGCAGTCAGACTCTGGACCCTGGCCTTGTCTGATACACTGTTACATGTATGAGTCTGGTACACTATTGAATCAGGCTTGACCATGTCTGACATTAATACTCTGAGTAATTGGTTTCAGACCCAAAAGCATGTAAGAAGAAATGGTACTCAACCTTATTCTCAAACAGTTACTACCTGGATGAAGCCAGAGAGAATGGTAAATAACCCACCCATGGAACCAATCTGAAAAGAATGCAGTTAGACCCTGGACACAGTCTGAATGTGATGTTATTCAACCGTGGACCTATGCTGAAATCAATACAGTCAGACACTGGACACATTCTGAATTTGATAAAATAGAACAATGGACTGAGCCTGAATCTCAAGAAATTAGGACCTGGCCTGAGGCGGAATGTTGACATGTTGGTCCCCAACTCAAAACTATGCAGTTTGGCCCTGGACCCAACTTGAATCTCAAATGGCACACTCCTGGACCCAGAATCAAGTTAGCATAATTACTCCTGGACTCAGCATGTACCTGCTACTATCAGATCATGGACTTATACTCTGAAATTCATCCCTGGACCCACCCTGAAGCCAATCCAGTGATAAGATACTGGTTCCAGACTCAAATGAATTCAATAAGATCCTGGAACCAATCTGAAACTGAAGTATTCCAAATTTGGACTGTAAGCCAAGGAATAAAGCCCTGAAACATGACTGAAATTGATACAGTCACATCTCACTTACAGACTCAATGTGATACAGTTAGACCCTGGATTCATCCTGAAAGTCTCTCCCTGGTCCAAACTGAAGTTGGTATATTTTGGCCCTGGACTCAGCAAAGAGCTGCTACATACCGAACCAATGCTGAGAACCAAGCAGTGAGACCCTGGATCAAGCAAGAAACTGATATAGTCAGATCTTCATTTAATATTCAAATGAATAAAGGCAGATCATGGGCTTATTTAAAATCTCATTCCCTGGATCCAGCCTGAAGTTGATATAATTCACGCTTTAATCCGCCAAAGCAGACATGCACTGGATCCAGTCTGAAGCTGATATTATTGAATGCTTTGCTGTTTTTAAAGCTGGTAAAGTGAGAACCTGGATCCAACCTGAAACAGAAATGCTAAGACCCAGAACCCATTATAAGGCTGATATAATTGTATCATTTTCTCCTCCTGAAATTGAGCTGAATGAAGAAACACTATTAACGAGTCACTTTGGCTCCTTGTCTAAACGTGTACCCTTTTTGGCAGTAAAAACTGTTTCTTTCCCAGATCACTATTTTATCACTTTGTTAACTGAGATACCTGTCACAGAAAGCCAAGATAAATCATTTCTCTCCAACCAAGCGAGCTTATAAGCATTTGCTTCCTAGAAGATTTGTTTACCAGCACTACGGCAGGAAATTAAAAATTATCAAGATAAAAGAAAGCCCTGATGTCCTAGGTACCTCTCTTATCTCTCTTTGTTCCTCCTTTTTCTTTTTTCTGTCTTGTTCTCTTCCATCTCCACATACACTGTGTTCTTCTTGTTCAATCTTTTCTTCTTGTGCATTCCCTTCATTTTGCATTTTCCCATCTTTCTCAGTTATTTTTCCTCTGGTCTCTTCTCCTGTTCTCCTATCCATAGCCTCTTCTGCTAATCCTCTTCAGAAAATATCTTTCTTAACATTTACTGAATAGTGCATTCTTTCCCATTCTTTCTCATCCTTGCATGCTGCTCCAGCCACACTTTTAACTTCTCCTGATGCCTGGATCTCAATCTGGACCCAAGCCTAGACAACAACCTCTTAGGCATTTAGAACTCAATGTTTCCCTGGTTGAGTGTCAACTAGCTGTGATATGGAAAGAAAGTTTCCAGACTTTCTGGCTCTTCAAGACAGCTGTTATTTCTCATGAAAACACAGAGTGTGGATTATGCCCTGGCCATGTAACCCTCTGTCCCAGCTGCTGGGAGGCAGGAATTGGTGAATTCCCTTGACAGTTTCTCTACAACTGTCTTTCTCCCATTTCTGTGCTGGCTCCATACTAAATGAACAGTGTATCCTTACCACAGCTAGATGTGCCTATTTTATAAAAAGCTCAGGAGCCCTGGCCCTAGTCCAGTGGGGCTTAGTCATCTTCTGGAATCTGCCCAAGCTGACTGTGAGCATTGACCATGCCATACCCTACCTAGCTCCCAAGGGACCTCTAGGACCTGGGTTGATCTTCCTGAAGCAGCCACTACATTTTCAACCCCTGGTGTTTCCTCTATGCTTGGAGGAAAGTCTGGAACAGGAGAAATTTACACAACTATATGACTATTGGCTATCTAGCTGGTCCCTTATGAGAGGAAGTTCTGGAATTCTGCAAAAAGGGCACCTGAGCAACCAACAAGTCAGCCCTTGTGCCCAATTGTGCCCCAAGCTGAATGAATTCACTTTTTGTGTAGAGGCCAAGACAGCTGTTGGGGAGGCTGGCTCGTGACTTGGGAGCACCTTTCGTATGCCATCTACAGCAAAAGGACACTTGGGTGCAAGTGGGAATCTTGAGTCACTTTGAGGAGCATTGCACAAAGCCCTGTGTCTTCAGCCAAGTGCACCCTTTTCTTTCTGGCTCCTGGGAGTGCCATGGCCTAGCCATGCACCCTGGCACCACCAGGGGCCCATGACTACCTCTGCTTCCATGTCCCTTTCAGTCTCTGCTTCTAAAGATGCCTTGACTTTTATCTCCGCTCCCACTTCCATTTGGCCACACTTCATCTCTCTGCCACAGCCTCAGACTTCAGCAGATCAGATTTCTCTGCAATATGCCATGCCTTGGCAGGCTGGGATCATCAGCTGTGGCAGTCAGGTCTACAGCGGGTCCATAGTTAGCAGCTCATGGGTTCTCATAGCTGCCCACTGTGTCAGGAACATGAGTCCTGAAGACACTGCTGTGATACTGGGCCTGAGGCATCCTGGGGCACAACTGAGAGTTGTGAAGGTGTCTACCATTCTGCTGCATGAGAGATTCTGGTTGGTGAGTGGAGCAGCAAGAAATATCCTGGCATTGCTACTCCTCCAAGATGTCCAGACTCCCATTCGGCTCTCAGCACTCCGGCTGTCTGAAGAATCTGAATAGTCCAGAAGGCTGGCTCTCTGGGGCACAAATTATTACACCAGGAGAGACTGATGAGAATGCAGAAATGTTAAAGCACAAGTGATGGAAGCTTCCAGCTGTGCCCACCTGTAACCTGACATAGACAGTTCCATTGTTTGCTTCATTACTCAGGTCAAAGACATTAATGCAAATGTGGAGTCAGTGAGTCCAGAAAATGTCATTATGCGCAGACCAATATCTGGCAATGGCACTAGGGGACAAATATGCTTTACCAGTCTCAAAGCCCTAGCTACTACAGTGAGTCCACACTTCTCCTGTATCTTATCTGCTTCAGCAAAAGAAGGCCACCACTAAATCAGGCCGTTGTGGCTTGGGTGGAAACTCCTAAGTCCTCTACTCTCCTTAAAGAGCCAACCACACTGCCACTTTCCTCAATAATAATTACTGCAGCTCTGAGACTTTGGTAGCCTAGTGACTATAACTATTGATGCTACAGTCTGGTCACAGTATGATAAAACACCAAAACAACAAAAACAAAAATATTGACTTAAGCCTTCTAAGA

>Chimpanzee, Pan troglodytes (model)

﻿AATTCCTGGGTACAAAATGAAGCTACTATATTAACACCATGCATTCAGGGAAAGTCTTGAGAATTAAATCCCTGACATAATCTGAAACTTCCACAGTGCCACTGTGGACCCAGACTGAAACTCCAACAGTAAACTCTTGGACAAATATGTTATCAGATACAGTCACAGTATGGACAAAGGCTGAATTTCAAGGATTAACATCCACCCATCTATTGAGGAAAACTGATCCAATCATACTGTGGACTATTGCCAAATCTCCAGCAGTGAATCTCTGGACACATTCTGTATGTGATGAAGTCAAATTTTGGAACCAAGAAGAACTTCCAGCCTTATTGCCTTTGACAGAGCCTATAGCTAAGACAGTCCCACAATGGATCCAGGATAGATTTCCAGTAATGAACCCCTGGGCAAAAACTATAGCTGAAACTGTCATACCATTGACCCTTGATGAAATGACACCAGTAAGTCCTTGGACAAAATTACAGCCTGAATATCTAGAAGGAAATACTTGGCTCCCATCTGAATTTAATCCAGTCTCATTGTGGACCATGGCTTATCTGCCAGCATAATTCCCTGGGAACAAGATATATTCCAACCATGGACCCACGCTGAACACCCACCAGTATCTTGGACTCAATCATTGATTCCGGGTAAAACAGCAGCAATGAATCCCTGGAGAGATGTTACAGCTGATATGGTCCTACCATGGAATCAGGCTGAATTTATAGTAGGAAATGCCTGGACATAGTCTGAAACTTATACAGTCACACAGGGGATCCTGAATGGTTCTCCAGCATGATATTCTTGGACACAACAAATAGCTGATGTAGACAAGCTGTGGACCAAGCCTGAATTTCCCACAATAAATCCCTGGACAAAGTCTGTAGGTGATAGTACGATACAATAGATTCAGGATGATCTCCAATGGTAAATCTGTGCACAGAGGCTGCAACTTCCAAAGTCACACCATGGGCCCAGGCTGTATCTCCAGCAGTAATTCCATGGACACAGCCTATACCTTCCATAATCGTACCTTGGACCCAGGTTGAATGTCCATCAGTAAATACCTGGACAATGTCCATAGCCAATACCATCACACTATGGAACCAGGCTGAATTAATAGCGGGATATCCATGGATACTGCCATGGGCTGCTACTATCACACTATGCACAGTCTGAATCTGCAGCAGGAAATCTTTGGACACAGCCTATATATGACACAGACACTCCATGGCGTAAGGCTGAATCTTCAGCAGTAGTGTACTGGACAGAATCTGTAGCTGATACACACACACCATGAACTCAATAAAAATCAAGAGTCTATTTTATGGACACATCATGAAATTGAATGTATCAGTGAATGGACTTTGCCTGAATTTGGAACACTTATATCCTGGATAGTGCCTGGGCCTCCAGCAGCAAAACCATGGTCCCAACCTGAAGCTCTAGTTATAGAACTTTGTTTAAAACTGGAACAGAAAAAATAAAACCTTGGGTTCACCAGAATTTCAAACACTGAGCACATTTACTCCCTTTGGACCTGGTAAAATAGAATCCTGGGCCAAACAGAGAACTACAACTTTAATAACATGGATCCAATCCAAAAGGGATGCCTTCTTCCCATATACACAATCTGAAGTAGGTACAATAAGATCCACAACAATTTCTGAAGCCGTTATAGTAAAATATGGATCCAGACTGAAGCAGGCACAATCCACCCTTGGACTCAAGCTAGAACTAATACAATCAGACCTTTGACTCATGCTGAATTTCAAGCAGTCAGACTCTGGACCCTGTCCTTGTCTGGTACGCTACTGTATCAGACTGAAATACAGGCAGCAAAATGCTTGACCATGCCTGATATTAATATTCTGAGTGCTTGGCTTCAGACCCAAAATTATGTAAGAAGAAATGGTACTCAACCTTACTCTCAAACAGTTACTACCTGGATGCAGCCATAATCCACCCATGGAACCAATCTGAAAAGAATGCAGTCAGACCCTGGACCCAGTCTGAAGGTGATGTTATCCAACCTTGGACCTATGCTGAAACCAATACTGTCAGACTCTGGACCCATTCTGAAACTGATAAAATAAAACAATGGACTGAGCCTGAATCTCAAGCAAGTAGGACCTGGCCTGAGACAGCTATGTTGATATGTTTGTCCCCAAAACGATGCAGTTTGACCCTGGACCCAACTTGAATCTCAAGCGTCACACTCCTGGACCCACAATCAAATTAGCATAAATTACTCTTGGATTTAGCATGCACCTGCTGCAATCAGACCATGGACTTACTCTGGAATTCATCCCTGGACCCACCATGAAACCAATAGAGTGATAAGATACTGGTTCCAGACTCAAATAAATTCAATGAGATACTGGAACCAACCTGAAACTGAAGTATTCCAAATTTGGACTGTAAGCCAAGGAATAAAACCCTGTAACATGACTGAAATTGATACAGTCACATCTTGCTGACAGCCTCAATGTGATACAGTTAAATCCTGAATTCATCCTGAAAGTCAGCCACTTTCTCCCTGGCCCCAGACTGAAGTTGGTATATTTTGTCCCTGGACTCAGCAAAGAGCTGCTACATATCAAACATGGACCCACCCTGAAACCCAAGCAGTGAGACCCTGGAACAAGCAGGAAACTGATATAGTCAGATCTTTGTTTTACATTCAGGTGCATAAAGGCAGCTCATAGGCTTATTCAAAATCTCAAATACTCAGTTCCTCGGTCCAGCCTGACATTGATATAATTCACAATTTTATTCACAGACACAGACTGGATCCAGTCTGAAGCTGATATTATTGAATCCTTTGTGGATTTTAAAGGTGGCAAAGTGAGAACCTGGATCTAGCCTGAAACAAAAATACTAAGACCCAGAACCCATTATAAGGCTAATATAATTGCATCATTTTCTCCTCCTGAAATTGAGCTGAATGGAGAAACACTATTAATGAGTCATTTTGTCTCCTTGTCTAAACGTGTAACCTTTTTGCCAGTAAAAACAGTTTCTTTCCCAGATCAGTATTTAATAGCTTTGTTAACTGAGATAACTGCCACAGAAAGCCAGGATAAAATCAATTCTCTCCAACCAAGCCAGCTTACAAGCATTTGGCTTCCTGGAAGAGTTGTTTACCAGCACTATGGAAGGAAATTAAAAATTATCAAGATAAAAGAAAGCCCTGAAGTCCCAGCTACCTCTCTTGTCTCTCTTTCTTCCTCCTTTTTCTTTCTTCTTTCTTGTTCTTTTCCATCTCCATGTACACTGTGTTCTTCTTAAGTCTTTTCTTCTTGTACATTCCCTTCATTCTGCGTTTTCCCATCTTGCTCAGTTTTTTCTCCTCTGGTCTTCTCTCCTATTCTTCTTTCCATAGCCTTTTCTGCTAATCCTCTTCAGAAAATATCTTCCTCAACATTTACTGAAGAGTCTATTCTTTCTCAATCTTTTTCATCCTTGCATGCTGCTCCAGCCACACTTTTAATAAAACACCCTCTCCTGATGCCTGGATCTCAATCTGGACCCAAGCCTGTACAACAACCTCTTAGGCATTCAGAACGCAATGTTTCCCTGGATGAGTGTCAACTAGCTGTGATCTGGAAAGAGAATTTACAGGCTTACTGGCTCTTCAAGACAGGTGTTATTTCTCGTTTATTCCTTCTGCAGAGTGTGGGTTACGCCCTGGCCATGTCCCCCTCTTTCCCAACTGCTGGGAGGCAAAAATTGGTGAATTTCCTTGGATAGTTTCCTGCAACTGTCTTTCTCTCATTTCTGTGCTGGCTCCATACTAAATGAACAGTGGATCCTTACCACAGCTAGATATGGCAATTTTATAAAAAGCTCAGGAGCGCTGGCCCTGGTCCAAATGGGGTGTAGTCATCTTCTGGATTCTGCCTAAGCTTAGACAGTCAGGATTGACAGTGCCATACCCTACCTAGGTCTCAAGGGACCCCTGGGTTGATCTTCCCAAGGCAGACACAACATTTTCAACCCCCTGGTCCTTCCTGTATGCCCAGAGGAAAGTCTGGAATGGAGTAAATTTATACAACTATGTGACTATTAGCTACCTAGTTGGTCCCTTATGAGGGAAAGTCCTGGAATTCAGCAAAAAAAGCACCCAAGCATTCTACAAATCAGCACTTGTGTCCAATTTTGCCCCAAGTTGAATGAATTCACTTTTTGTATGGTGGCCAAGAAAGCTATGTGGGAGGCTGGCTCTAAGGGTGACCTGAGAGCACCTTTGGTGTGCCAGCAACAGCAAAAGGACACTTGGGTGCAAGTGGGAATCTTGTTACTTTGAGGAGCATTGCACAAAGCCCTGTGTCTTCAGCCAAGTGCACCCTTTCCTTTTCTGGCTCCAGGGAGTGACATGGCCTAGCCATGCACCCTGGCACCATCAAGGACACATGACTACCTCTGCTTCCATGTCTCTTTCAGTCTCTACCTCTACAAATGCCTTGGCTTTTACCTCCACTGCTAGTGCTTTCCGGCCACACTTCATCTTTCTGCCACAGCCTCAGACTTTGGCAGATCGGATTGCTCTGCAATATGCTATGCCTTGGCAAGCTGTGATCATCAGCCATGGAAGTGATTTCTGCTGTGGTTCCATAGTTAGCCACTGTTGGATTCTCACAGCTGCCCACTGTGTCAGGAACATGAATCCTGAAAACACTGCTGTGATTCTGGGTCTGAGGCACACTGGCACACCACTGAGAATTGTGAAGGTGTCTATCATGCTATTTCATGAGAGATTCCGGTTGTTGAGTGGGGTAGAAAGAAATGATCAGGCTTTGCTACTCCTCCAAGATGTCTAGACTCCCTTTCGGCTCTTAGCACAGTTGGGCTATCTGAAAAACCTGAATAATTCAGAATGCTGGCTGTCTGGGCCACAAATTATTATACCAGGAGAGACCGATGAGAATCCAGAAATGTTAAAGATGCAAGTGATGGAAGCTTCCAGCTGTGCCCACCTGTACCCTGGCATAGGCAGTTCCATTGTTTGCTTCATTACTCAGACCAAAGACTCTAATGCAAATGAGTCAGTGAGTTCAGGAAGTGTCATTATGTGCAGACCAATATCTGGCAATGGCAGTTGGAGACAAATAGGCTTCACCAGTCTCAAAGCCCTAGCTACTACAATAAGTCAACACTTCTCCTGGATCTTATCTACTTCAGCAAAGGAAGGCTACCCACTAAACCAGGCCCATGTGCTTTGGGTGAAAACTCCTAAGTCCTCTAGCCTCCTTAAACAGCCAAGCACGCTGACACTTTCTTCAATAACAATTGCTGCAGCCCTGAGACTTTGGTAGCCTAGTGACTATAACTAGTGATGCTACAGTCTGGTCACAGTGTGATAAAACACCAGAGCAACAACAACAAAATGTTGACTTAAGCCTTCTAA

>Rhesus, Macaca mulatta (model)

﻿AACCCCTGGGTACAAAATGAAACTACTATATTAACAGCATGGATGCAGGGAGAGTCTCAGTAATTAAATTCCTGGACACAATCTGAGACTCACACAGTCCCACTGTGGAGCCAGACTGAAACTCCAACAGTAAACTATTGGACCAAGATGTTAGCAGATATAGTCACCACCTCGACAAAGTCTGAATTTCAAGGATTAACATCCTGGAGACAGTCTGAAACTGATCCAGTCATGCTGTGGACTATTGCCAAATTTCCAGCAGTGAATCTCTGGACACATTCTGTATCTGATAAAGTCACACCCATGGACTATGGCTTATTTGCCAGCAGTAAATTACTGGATATAGTTTGATACTAAAACAGTAACACTGTGAACCCAGGTAGAGTCCCCAGCAATACCTTCCTGGAAACAGTTGGAAACTGATATAATTATACTAGGGACCTATGCTGAATCACTCGTGATGAATTCATGGATATATTCTCCTACATCGGATACGGTCACAATGTGTATCCAGGCTGAATCACTATTATTACATCCTTGTACACACTCTAGGAGTAAGTCACTGAGCACATGATATATTCCTACCATGGACCCACACTGAACATCCACCAGTATCTTGGACTCAACCATGGATTCCAGGTGAAACTCCAGCAATGAATCATTGGAGAGAGGCTACAGCTGATACGGTCCTACCAAGGAATCAGGCTGAATTTATAGAAGGAAATGCCTGGACACAGTATGAAACTTATGCAATCACACAGGGGATCCTGAATGATTCTCCAGCACAAGATTCATAGACACAGAAAATAGCTGATGCAGACAAGCTGTGGACCAAGCCTGAATTTCTAGCAATACATCCTTGGACAAAGTCTGTAGTTGATAGTACTATACAATGGATTCAGGATGAAGTCTCCAACAGTAAATCCATGCACAGAGGCTGTTAATTCCATAGTCACACCATGGGCCCAGGCTGTATCTCCAGCAGTAATTCCATGGACACAGTCTATACCTTCCATAATCATACCTTGGACCCAGGTTGAACGTCCATCAGTAAATACTTGGACAATGTCCACAGCCGATACCATCACACTATGGAAAGGGGCTGAAAAAACTGCAGGATATCCATGGATACTGCCTTAGGCTGCTACTATCACACTATGGGCACGGTCTGAATCTCCAGCAGGAAATCTTTGGACACAGCCTGTGTATGACACAGATACACCATGGCCTCATGCTGAATCTCCAGCAGTAGTTTACTGAACAGAACCTGTAGCTGATATACACAACCTTTGATCTGACATGAAAATCAAGCATCTAATTTATGGACACATCATGAAATTGAAAATATCAATGAATGGACCTTGCCTGAATTTGGAACACTTATATCCTGGATAGTTCCTATGCCTCGAGCAGCAAAACTATGGTCCCAACCTGAAACTCTAGTTGGCAGAACTTCGTTTAAAACTGGAACAGAAAAAGTAAAACCTTGGACTCAGCCAGAATTTCAAACACTGAGCACATTTACTCACTTTGGACCTGGTAAAATAGAATCCTGGGCCAAACATAGAACTACAACATTTATATCATGGATCAAATCCAAAACTGATGCCTTCTTCCCATGTAACCAATCTGATGAAGGTGCAATGATATCTCAGACCATTTCTGAAGCTGATACAGTAAAACTATGGATCTGGACTGAAGCAGGCAAAATCCACCGCTAGACTCAAGCTAAAACTATTACAATCAGTCCTTTGACTCATACTGAATTTCAAGCAGTCAGAACCCGGGCCCTGTCCTTATCTGATACACTATTGTATCAGGCTGAAATGCAGGCAGCAAAATGTCTGACCATGCATGACATTAATACTCTGAGTGCTTGGTTTCAGACCCAAAACTATATAAGAAGAAATACTACTCTACCTTATCGTCAGTTACTACCTGGATGCAGCCAGAATGCCAAAAAATCCAGCCATGGAACCAATCTGAAAAGAATGAAGTCAGACCCTGGACCCAATCTCAAAGTGATGTTATCCAACCTTGGATCTATGCTGAAACCAATACAGTCAGACACTGGACCCATTCTGAAACTGATAAAGTAAAACAATGGACTAAGCCTGAATCTCCAGTAATTAGGACCTGGCCTGAGGCCAGGTTGACACATTCATACCCAAAATGATGCAGTTTGGTCCTGGACCCAACTTGAATCTCAAATGATACATTCCTGGACCCAGAATCAAGTTAGCATAAATTACTCTTGTATTCAGCATGGACCTGCTACAATCAGACCATGGACTTACTCTGAAATTCGTCCCCGGAACTACCCTGAAATAAAACCCTGAAATGTGACTGAAATTGATACAGTTATATCTTGCTTACAGACTCAATGTTATATAGTTAGACCCAGGATTCATCCTGAAAGTCAGTCGCTCTTATCCCTGGCCCCAGACTGAAGTTGGTATATTTTTGCCCTGGACTCAGCAAAGAGCTGCTACATATCCAACCTGGACCCACCCTTAACCCAAGCAGTGAGACACTGGATCAAACAGGAAACTGATATCGTCAGATCTTCATTTTACATTCAAATGAATAAAGGCAGCTCATGGGCTTATTCAAAATCTCCAATTGTCAGTACCTCAATCCAGCCTGAAGTTAATATAATTCACCCTTTTATCCACAAAGAGACACTGGATGCAGTCTGACGTTGATATTATTGAATCCTTTGCTATTTTTAAAGCTGCAAAGTAAGAACCTGGATCCAGCCTGAAACAGAAATACTAAAACCCAGAACCCATTATAAGGCAGACATAATTGCATCATTTTCTCCTCCTGAAATTGAGCCAAATGGAGAAACACTATTAACGATCATTTTGGCTTCTTGTTTAAACATGTACCATTTTTGCCAGTAAAAACTGTGCCTTTCCCAGATCAGTATTTTATAGCTTTGTTAAGTGAGATAACTGTCACAGAAAGCCAAGATAAAATCAATTCTCTCCAACCAAGCCAGCTTACAAGCAATTGGTTTCCTGGAAGAGTTGTTTACCAGCATTATGGTGGGAAATTAAAAATTATCAAGGTTAAAGAGAACCCTGATATCTCCTATACCTCTCTTGTCTCTCTTTCTTCTTCCTTTTTCTTTGTTCTTTCTTATTCTCTTCCATCTGCATGTACACTGTGTTCTTCTTATTCAGTCTTTTGTTCTTGTTCATTTCCTTTATTCTGCATTTTCCCAGCTTGCTCAGTTGTTTCTCCTCTAGTCTCCTCTCCTATTCTCCTATCCATACGCTCTTCTGCTAATCCTCTTCAGAAAATATGTTCCTCAACATTTACTGAAGAGTCCATTCTTTCTCATTCTTTTTCATCCTTGCATGCTGCCACAGCCACACTTTTAACAAAACAACCTCTCCGGATGCCTGGATCTCAATCTGGACCCAAGCCTGAATGACAACTTCTTAGGCATTCAGATCTCAATGTTTCCCTGGTTGAGCGTCAACTAGCTGTAATCTGGAAAGAAAGTTTCCAGGTTTTCTGGCTCTTCAAGACAGGTGTATATTCCTTCCGCAGATTGTGGATTACACCCTGGCCATGTCCCCCTCTGTCCCAACCGCTGGGAGGCAGAAGTTGGTAAACTCCCTTGGATAGTCTCTGTGCAACTGTCTTTCTCCCATTTCTGTGCTGGCTCCATACTAAATGAACAGTGGTTCCTTACCACAGCTATATGTGCCAATTTTATGAAAGTCTGAAGAAACACAGATAGAGACAGTGTCTTGCTCTGTTGCCCAGGCTGGAGTGCAGTGGCACTCCAGTACTGATTCACAGTAGCCTCAAATTCTCAAGAATAAAGGATTCTCCCACAGCTGCCTCCTGAGTAAATGGGACCACAGGCACACACCACTGCATCTGGCTAATTTATACTACTATGTGACTATTGGCTAGTTAGTTAGTCCCTTATGAAGAAAAGTGCTGGAATTCTACAAAAAAGCACCTGAGCATCCTACAAGTCAGCACTTGTGCCCAATTTTGCCCCAAGCTGAATGAATTCCTTTATTTGTGGAGGTCAAGAAAGCTATGCAGGAAGATGGCTGGTGACCTGGGAGCACCTTTGGTGTGCCATCTACAGCAAAAGGACACTTGGGTGCAGGAGGGAATATTGAGTAACTTTGAGGAGAGTTCCACAAAGCCCTATGTCAGTCAAGTGCATGCTTTCGTTTTCAGGCTCCAGGGAGTGACACAGCTTAGCCATGCACCCTGGCACCAGCAACGACCCATGACTACCTTGCTTTCATGTCTCTTTCAGTCTCTTCGTCTACAAATGCCTCGGTTTTTACCTACACTCCCAAATCTATTCAGCCACACTTCATCTCTCTGCCACAGCCTCAGACTTCAGCAGATCAGATTTCTCTGCGATATGCCATGCCTTGACAGGCTGTGATCATCAGCTGTGGTAGTTAGGTCTGCAGTAGTTCCATAGTTAGCAGCTCTTGGGTTCTCACAGCTGCCCACTGCATCAGGAACATGAATCCTGAAGACACTGCTGTGATTCTGGGCCTGAGGCACTCTGGGGCACCACTGAGAGTTGTGAAAGTGTTTACCATTCTACAGCATTAGAGGTTCTGCTTAGTGAGTAGGGCAGCAAGGAATGACCCAGCATTGCTACTCCTCCAAGATGTCCAGACTCCCATTTGGCTCTTAGCACCCTTGGGCTATCTAAAGAACCTGAATAGTTCAGACTGCTGACTCACTGGGCCACAAATTATTACACCAGGAGAGATTGATAAGAATTCCAAAATGTTAAAGATGCAAGTGATGGAAGCTTCCAGCTGTGCCCACCTTGTCCCTGGCATAGGCAGTTCCATTGTTTGCTTCATTACTGAGGCCAAAGACTCTAATGCAAATGTGGAGTCAGTGAGTCCAGGGAGTGCAGTTATGTGCAGACCAATATCTGGCAATGGCAGTTGGAGACAAATAGGTTTCCCCAGTCTCAAAACCCTGGCTAGTACAGTGAGTCTGCCCTTCTGGATCTTATCTACTTCGGCAAAAAAAGGCCACACACTAAACCAGGGCCTTATGCTTTGGGTGGAAACTCCTAAGTCCTGTAGTCTCCTTAAACAGCCAACCACACTGCCACTTGCTTCAATAATAATTACTGCAGCACTGAGACTTTGGTAGCCTAGTGACTATAACTAGTGATGCTATAGTCTGGCCACAGTATGATAAAACACCAGAGGAACCAAAACAAAAATATTGACTTAAGCCTTCTAA
